# Liquid-jet photoemission spectroscopy as a structural tool: site-specific acid–base chemistry of vitamin C†

**DOI:** 10.1039/d4cp01521e

**Published:** 2024-06-28

**Authors:** Lukáš Tomaník, Michele Pugini, Karen Mudryk, Stephan Thürmer, Dominik Stemer, Bruno Credidio, Florian Trinter, Bernd Winter, Petr Slavíček

**Affiliations:** a Department of Physical Chemistry, University of Chemistry and Technology Technická 5 16628 Prague Czech Republic Petr.Slavicek@vscht.cz; b Molecular Physics, Fritz-Haber-Institut der Max-Planck-Gesellschaft Faradayweg 4-6 14195 Berlin Germany winter@fhi-berlin.mpg.de; c Department of Chemistry, Graduate School of Science, Kyoto University Kitashirakawa-Oiwakecho, Sakyo-Ku 606-8502 Kyoto Japan

## Abstract

Liquid-jet photoemission spectroscopy (LJ-PES) directly probes the electronic structure of solutes and solvents. It also emerges as a novel tool to explore chemical structure in aqueous solutions, yet the scope of the approach has to be examined. Here, we present a pH-dependent liquid-jet photoelectron spectroscopic investigation of ascorbic acid (vitamin C). We combine core-level photoelectron spectroscopy and *ab initio* calculations, allowing us to site-specifically explore the acid–base chemistry of the biomolecule. For the first time, we demonstrate the capability of the method to simultaneously assign two deprotonation sites within the molecule. We show that a large change in chemical shift appears even for atoms distant several bonds from the chemically modified group. Furthermore, we present a highly efficient and accurate computational protocol based on a single structure using the maximum-overlap method for modeling core-level photoelectron spectra in aqueous environments. This work poses a broader question: to what extent can LJ-PES complement established structural techniques such as nuclear magnetic resonance? Answering this question is highly relevant in view of the large number of incorrect molecular structures published.

## Abstract 2

Liquid-jet photoemission spectroscopy directly probes specific molecular structure of solutes.

## Graphical abstract

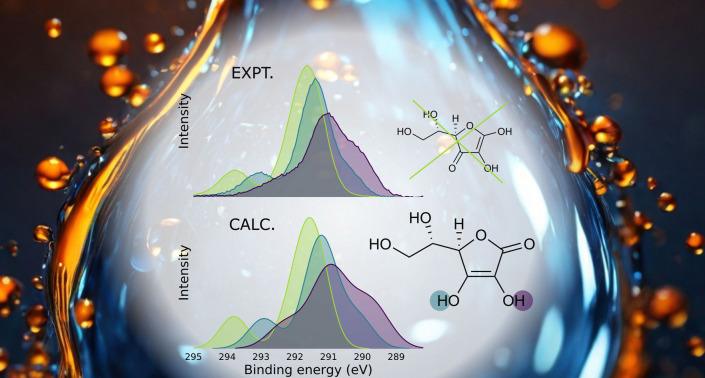

## 1 Introduction

Photoemission (PE) spectroscopy from the liquid phase was made possible *via* the liquid-microjet (LJ) technique,^1^ and has since become an increasingly applied tool for studying aqueous solutions as it directly probes the liquid's electronic structure.^2–4^ A growing body of experimental data in the field made us ask: can liquid-jet photoemission spectroscopy (LJ-PES) be used as a general structural tool in liquid-phase (and interface) chemistry? We are inspired by the ESCA (Electron Spectroscopy for Chemical Analysis) technique used for gaseous as well as solid-state samples and associated interfaces,^5^ where one determines chemical shifts in binding energies (BEs) of core-level electrons. As the core-level electrons are localized on a single atom, the observed shifts provide information on the specific chemical environment of the ionized atom. This approach can also be extended to liquids, where the PE spectrum, expressed in terms of BEs of the electrons, provides us with the full set of electronic-structure data on the studied solutes.^6^ For example, if we analyze all the aqueous-phase LJ-PES data up to May 2020, according to the list of publications in ref. 7, we obtain the following values for C 1s core-level BEs: 289.5–290.5 eV for the –CH_2_– group, 291–291.5 eV for the C–OH group, 293–293.7 eV for the –COO^−^ group, and 293.9–295 eV for the –COOH group. This demonstrates that core-level LJ-PES can be sensitive enough to reveal a specific chemical environment of individual atoms in the molecule. The technique has additional advantages, *e.g.*, the surface sensitivity of photoemission has been used to probe air–liquid interfaces^8,9^ or to study electrode–electrolyte interfaces.^10–12^

Analogous to PE spectroscopy in the solid state, recent developments now enable the accurate determination of electron BEs in the liquid (typically aqueous) phase.^13^ In addition, Auger electrons emitted after subsequent decay of the core hole can reveal the liquid structure and associated electron (and nuclear) dynamics;^14–16^ we will not discuss this emission channel in the present work. One of the limiting factors for a wider-community application of LJ-PES as a chemical analysis tool can be the need for suitable X-ray sources, usually limited to synchrotron-based facilities. However, recent developments of laboratory-based sources relying on high-harmonic generation (HHG) promise to significantly ease access to table-top X-ray generation, possibly vastly increasing the number of studies in the near future.^17–20^

Utilizing chemical shifts revealed by photoelectrons from solution to gain structural insight appears to provide a promising complementary route to powerful structural analysis techniques such as nuclear magnetic resonance (NMR).^21^ It took many years for NMR to be fully developed and established as a conventional structural tool. Yet, even the well-established NMR has witnessed surprisingly frequent structural misassignments. This is especially the case for natural products, an important group of compounds in drug development. There are hundreds of reportedly misassigned structures of even medium-sized molecules.^22–25^ The present work should contribute to the development of LJ-PES as a complementary tool for chemical (structural) analysis for molecules solvated in liquids. It could extend the portfolio of techniques to analyze the structure of liquids from the perspective of individual atoms, *i.e.*, useful to decipher molecular structure and speciation.

LJ-PES has been successfully applied, *e.g.*, to study interactions of hydroxide ions (OH^−^) with water,^26^ different charge states of amino acids,^27^ or specific solvation structures of biomolecules.^28,29^ The technique could even trace minor conformational changes following solvation.^30^ Recently, we have demonstrated that when combined with theoretical modeling, LJ-PES is able to reveal acid–base transformations in complex cases.^31^ For example, in the case of the glucose molecule, we have shown that the specific deprotonation site out of five candidates in the molecule can be unequivocally assigned using C 1s core-ionization spectra when comparing with calculated spectra modeled for each hypothetical deprotonation center. Core-electron BEs contain site-specific information on the chemical environment, reflecting the molecular structure of the ionized site. We identified the real deprotonation center based on spectral shape and peak positions, *i.e.*, only one of the modeled spectra matched the measured one. This proved the ability of the combined theoretical-experimental approach to site-specifically probe the acid–base chemistry of a molecule in an aqueous solution. The immediate follow-up question is how general such an approach is and whether the technique is also applicable in the case of multiple subsequent deprotonation events.

**Fig. 1:**
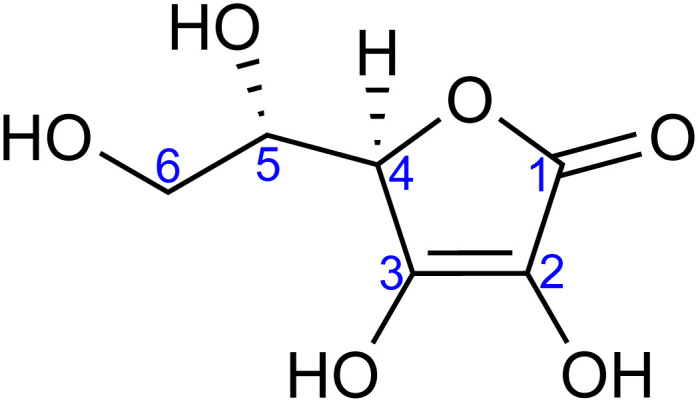
Molecular structure of l-ascorbic acid (vitamin C) in its fully protonated form. The numbering of carbon atoms, as used in this work, is indicated.

With that in mind, the current study focuses on the acid–base equilibria of vitamin C (l-ascorbic acid) revealed from LJ-PES. This vital water-soluble micronutrient has attracted much attention since the first pioneering studies of Albert Szent-Györgyi in the 1920s.^32^ Vitamin C's molecular structure, as shown in Fig. 1, is derived from glucose and exhibits versatile properties, enabling participation in numerous biochemical reactions, notably as an antioxidant.^33,34^ Its dual acidic and reducing nature allows it to donate protons and electrons, playing a pivotal role in cellular redox reactions, protecting biomolecules from oxidative stress. It also aids in iron absorption, collagen synthesis, neurotransmitter production, immune function, and regeneration of other antioxidants such as vitamin E.^34–36^

**Fig. 2:**
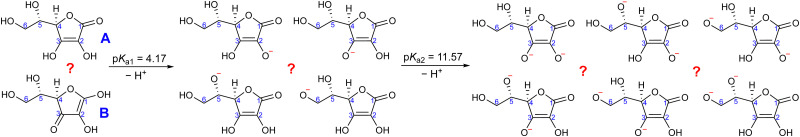
Molecular structures of vitamin C in its fully protonated, singly deprotonated, and doubly deprotonated form. Two fully protonated tautomers, four hypothetical single deprotonation sites, and six hypothetical double deprotonation sites of the A tautomer are shown.

Ascorbic acid as the molecular basis of vitamin C was identified by Szent-Györgyi between 1928 and 1932. In 1933, Walter Norman Haworth correctly deduced vitamin C's constitution based on chemical reactivity. The concepts of carbohydrate chemistry were well developed in the 1930s, and no alternative explanations, such as linear aldehyde structures, were considered. The story of vitamin C represents one of the final achievements of the classical era of structural elucidation before the field started to be dominated by various spectroscopies.^37^ The molecular structure of ascorbic acid can be considered in the form of different tautomers; see, *e.g.*, structures A and B in Fig. 2, with the A form assigned as the correct one in aqueous solution by ^13^C NMR peak positions and splitting.^38^ Vitamin C's molecule exhibits four hydroxyl groups, thus representing a polyprotic system with multiple possible deprotonation sites. The first acidity constant is p*K*_a1_ = 4.17,^39^ and thus ascorbic acid is predominantly singly deprotonated at pH = 7, see Fig. 2. The deprotonation site is reported to be located at the –OH group at carbon site C3 based on pH-dependent chemical shifts in ^13^C NMR.^38^ With the second acidity constant being p*K*_a2_ = 11.57,^39^ ascorbic acid is doubly deprotonated in strongly alkaline solutions (Fig. 2) with the second deprotonation taking place at C2, again determined by ^13^C NMR.^38^

Here, we apply our combined experimental–theoretical approach using LJ-PES and *ab initio* modeling to probe the acid–base structure of vitamin C in an aqueous solution. We performed pH-dependent measurements of solutions at pH values of 2, 7, and 13 in order to be well below or above the respective p*K*_a1_ and p*K*_a2_ values. This means that we investigate a different acid–base structure in each solution – fully protonated at pH = 2, singly deprotonated at pH = 7, and doubly deprotonated at pH = 13 (Fig. 2). We show the versatility of core-level LJ-PES as a valuable tool to explore tautomeric forms and acid–base molecular structures as we site-specifically probe not only the single deprotonation, as in the case of our previous work on glucose, but also double deprotonation for the first time. As the accumulated empirical data do not allow for a theory-free determination of the chemical structure, we rely on *ab initio* theory. It is vital to employ a fast, reliable, and stable computational protocol to screen through a larger number of molecular structures. Therefore, the methodological aspects of the computations are explored. We present an efficient computational approach to model the spectra from a single molecular geometry based on the maximum-overlap method and compare the protocol to calculations from an ensemble of structures.

## 2 Methods

### 2.1 Experiments

LJ-PES measurements were performed at the P04 soft-X-ray beamline at PETRA III^40^ (DESY, Hamburg, Germany) using the EASI setup.^41^ A vacuum LJ of ∼26 μm diameter was ejected with a velocity of ∼25 m s^−1^*via* a fused-silica nozzle. A high-performance liquid chromatography (HPLC) pump was used for solution delivery with a constant flow rate of 0.8 ml min^−1^. The temperature of the ascorbic-acid aqueous solution after injection was estimated to be in the range of 279–283 K in the laminar region of the LJ, which typically extends for 5–10 mm from the capillary into vacuum.^1,2^ This laminar region is brought into overlap with the X-ray beam and the photoelectron detection axis. The liquid subsequently freezes and is collected by a liquid-nitrogen-cooled trap at the far end of the interaction chamber. A small metallic tube, placed into the main polyether ether ketone (PEEK) liquid-delivery line, enables electric grounding of the liquid or application of a defined bias voltage.

Circularly polarized light from the beamline, with a focal size of 180 μm in the horizontal direction (parallel to the liquid jet) and 35 μm in the vertical direction (perpendicular to the liquid jet), intersected the jet perpendicular to the flow of the solution. The small focal size allowed for the spatial overlap with the liquid jet. Photoelectrons were detected in a backward-scattering detection geometry, corresponding to an angle of 130° with respect to the light propagation direction, *i.e.*, near magic angle,^41^ thus minimizing any differential sensitivity to the photoelectron angular distributions. A single photon energy of 400.88 ± 0.02 eV was used for measuring all the C 1s PE spectra from the aqueous-phase ascorbic acid in this study, which corresponds to a (unbiased) kinetic energy of about 105 eV for C 1s electrons. C 1s spectra obtained at 850 eV photon energy, corresponding to a slightly increased probing depth into solution, from approximately 20 Å (∼7 solution layers) to 30 Å (∼10 layers),^42^ exhibit very similar shapes; see the ESI† for details. Calibration of the photon energy was done by measuring well-known absorption features of gas-phase N_2_,^43^ right before the solution spectra were measured. In all cases, the energy resolution of the P04 beamline was better than 250 meV. The energy resolution of the hemispherical analyzer was 100 meV, yielding a total energy resolution better than 270 meV. Experimental C 1s electron BEs reported in the present study refer to an absolute energy scale (against the vacuum level). This was determined as the energy distance between the position of the spectral low-energy cutoff and the position of the C 1s peak (both measured from a liquid jet biased with −64 V, thus accelerating the photoelectrons) and from the accurately known photon energy; this procedure of determining absolute electron BEs from liquids and (aqueous) solutions has been detailed in ref. 13. We estimate the resulting error for all stated BE values to be ±0.1 eV, which is a combination of fit errors and the accuracy of our measurements.

Commercially available l-ascorbic acid, C_6_H_8_O_6_ (Sigma-Aldrich, >99% purity), was dissolved in MilliQ (18.2 MΩ cm^−1^) water to prepare 0.5 M solutions. Ascorbic-acid aqueous solutions of 0.5 M concentration without prior pH adjustment were found to exhibit a pH of 2, which is well below the first acidity constant, p*K*_a1_ = 4.17,^39^ and all solute molecules are fully protonated. A small amount of NaCl (yielding 50 mM concentration) was added to ensure sufficient solution conductivity and minimize sample charging due to photoionization and the streaming potential.^2^ The conductivity is readily maintained for pH-adjusted solutions in the following. In order to shift the population to dominantly singly and doubly deprotonated ascorbic acid, the solution pH was adjusted to 7 and 13, respectively. This was done by addition of NaOH pellets into the aqueous solutions under constant magnetic stirring. The solution pH was monitored with a previously calibrated pH meter (VWR, pHenomenal 1100L).

### 2.2 Computational strategies

The interpretation of the liquid-phase PE spectra relies on electronic-structure calculations, which requires solving two issues: accurate calculation of BEs and proper treatment of solvation.^44^ Different flavors of density functional theory (DFT) are typically used to model the valence PE spectrum, *e.g.*, by using a combination of DFT with time-dependent density functional theory^45^ or utilizing the ionization-potential theorem combined with optimally tuned range-separated hybrid functionals.^46,47^ Correlated *ab initio* calculations, *e.g.*, using equation-of-motion coupled clusters (EOM-CC) theory typically provide more accurate results but at a higher computational price.^48–50^ An alternative strategy employs Green's function formalism.^51,52^ The calculations are more complicated for core-electronic levels, *i.e.*, aiming at highly excited states of the ions. Here, the core-valence separation can be used in conjunction with EOM-CC,^53,54^ representing the state-of-the-art approach. Alternatively, the maximum-overlap method^55^ (MOM) can be used. MOM is based on modifying the convergence criteria to not only minimize eigenvalues but also to maximize overlap with the orbitals from the previous step. In this way, the approach prevents the variational collapse of the wavefunction and keeps the hole in the desired orbital. This represents an efficient and accurate approach as MOM can be combined with any electronic-structure method and includes full electronic relaxation. Based on our previous experience,^31^ we aim to utilize MOM in this way to describe core-level ionization in this study.

The most efficient approach to cover the solvation effects is using the dielectric continuum model.^56^ It is mandatory to use its non-equilibrium form to separate the fast electronic and slow nuclear response.^57,58^ The specific solvent effects can be conveniently added within cluster-continuum models^59,60^ by including a few closest solvent molecules into the quantum-mechanical calculation explicitly, while the rest is represented by the dielectric continuum. However, when dealing with systems exhibiting highly concentrated charges, *e.g.*, containing –O^−^ groups, a large number of explicit solvent molecules may be required to achieve convergence.^61,62^ Alternatively, the QM/MM (quantum mechanics/molecular mechanics) or fragmentation techniques can be used.^63,64^

### 2.3 Calculations

#### 2.3.1 Photoelectron spectra from a single optimized structure

We modeled the PE spectra for two fully protonated tautomers and for all hypothetical singly and doubly deprotonated forms of the A tautomer of ascorbic acid to compare them to the experimental data. In total, we used two fully protonated (A and B tautomers), four singly deprotonated (on the hydroxyl groups of C2, C3, C5, and C6), and six doubly deprotonated forms, as shown in Fig. 2. The optimizations were performed using the hybrid functional based on the B3LYP functional with the Coulomb-attenuating method, together denoted as CAM-B3LYP,^65^ and Pople's basis set 6-31+G*. This choice is based on our previous experience and testing.^31^ All optimized structures were confirmed to be real minima by the absence of imaginary vibrational frequencies. To mimic the solvent polarization effects, the polarizable continuum model^66,67^ (PCM) was used in the optimization. Default PCM parameters, *i.e.*, universal force field (UFF) atomic radii^68^ and an electrostatic scaling factor *α* = 1.1, were used. Furthermore, each singly deprotonated molecule contained six water molecules placed around the deprotonated hydroxyl group (–O^−^), and each doubly deprotonated molecule contained 12 water molecules around the two –O^−^ groups. This is known as the cluster-continuum approach, where a few explicit solvent molecules are placed around the molecule to describe specific short-range interactions, such as hydrogen bonds, while the rest of the solvent is represented by the PCM.^59,60^ The optimizations were performed using Gaussian 09, revision D.01.^69^ Cartesian coordinates of all optimized structures can be found in the ESI.†

The core-level BEs were calculated for optimized structures using the maximum-overlap method^55^ in its improved form, called initial maximum-overlap method,^70^ which yields better convergence. Here, we used the CAM-B3LYP functional with the aug-cc-pVTZ basis set^71,72^ for hydrogen atoms and the core-enhanced aug-cc-pCVTZ basis set^73^ for carbon and oxygen atoms. From our experience, this combination provides a very good accuracy (as we also demonstrate below in the present case) for an acceptable computational price. The solvent effects were included by non-equilibrium PCM,^56,57^ while partitioning the solvent response to slow (nuclear) and fast (electronic) responses to describe the rapid nature of the photoionization process. The same PCM parameters (UFF atomic radii, *α* = 1.1) as in the optimization described above were used. The calculations were performed in the Q-Chem package, version 6.0.^74^ The sample input is shown in the ESI.†

PE spectra were constructed from the calculated BEs as follows. Each energy value was broadened into a Gaussian function of the same intensity and width, and the Gaussian functions were summed to create the spectrum. The width of the Gaussian functions was set empirically for each protonation state to reproduce the experimentally observed spectral widths. Specifically, the standard-deviation value was set to 0.42, 0.45, and 0.52 eV for fully protonated, singly deprotonated, and doubly deprotonated molecules, respectively.

#### 2.3.2 Photoelectron spectra from an ensemble of sampled structures

To compare the modeled spectra based on a single optimized geometry, we also modeled spectra based on an ensemble of structures, an approach called the nuclear-ensemble method (NEM).^75–77^ It is based on a projection of the ground-state nuclear density onto the ionized state. Within this approach, the probability of ejecting an electron with kinetic energy *E*_*k*_ from ionization with photon energy *E* can be expressed as1
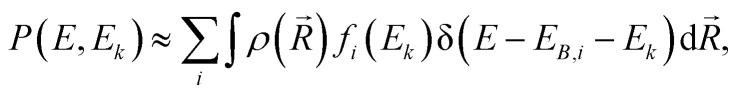
where *E*_*B*,*i*_ is the binding energy of *i*-th electron in a molecule of a geometry 
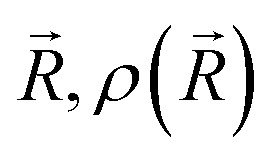
 is the ground-state nuclear density and the electronic factor *f*_*i*_(*E*_*k*_) is assumed in our study to be constant for all kinetic energies.^78^ Moreover, it has been shown that the number of sampled structures to represent 
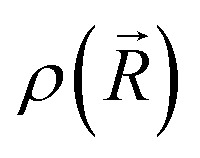
 can be significantly lowered even to a single representative structure.^79^

We used NEM here only for the fully protonated form (tautomer A), the singly deprotonated form at C3, and the doubly deprotonated form at C2 and C3. The geometries were sampled from a quantum mechanics/molecular mechanics simulation of a droplet containing the ascorbic-acid molecule in the respective protonation state and 1000 water molecules. The quantum part was applied to the ascorbic acid and was described at the B3LYP/6-31+G* level of theory with Grimme's D2 dispersion correction.^80^ The MM part modeling the water molecules was described by the TIP3P model.^81^ The temperature was kept constant at 280 K (close to the experimental conditions) during the simulation using the Nosé–Hoover thermostat.^82,83^ With this approach, we ignore nuclear quantum effects; however, they are usually not of significance for modeling PE spectra in the liquid phase.^84^ The initial droplet structure was created by the Packmol code,^85^ and the QM/MM simulation was performed with our ABIN code^86^ connected to Terachem, version 1.93.^87,88^ The total simulation length was 50 ps, with a time step of 0.5 fs. The first 10 ps were discarded as equilibrating period. From the remaining 40 ps, 100 geometries were sampled with equidistant steps of 400 fs. Geometries which contained the same number of explicit water molecules as in the single optimized geometry calculations were extracted, *i.e.*, 0, 6, and 12 water molecules for fully protonated, singly deprotonated, and doubly deprotonated forms, respectively. These water molecules were again chosen to be the closest to the deprotonated –O^−^ sites. The sampled geometries can be found in the ESI.†

The extracted 100 geometries for each protonated form were used as input for core-level binding-energy calculations employing exactly the same methodology as described for the single-geometry calculations. The resulting spectrum was again constructed from individual Gaussian functions with the same widths as detailed above.

## 3 Results and discussion

### 3.1 Core-level C 1s ionization for a site-specific probing of acid–base structure

Our goal is to demonstrate the potential of LJ-PES for elucidating structural information. For this, we focus on the well-described example molecule of vitamin C and show that the chemical insight obtained from LJ-PES can be as good as the one achieved from NMR studies, with LJ-PES having the additional capabilities described in the introduction.

**Fig. 3:**
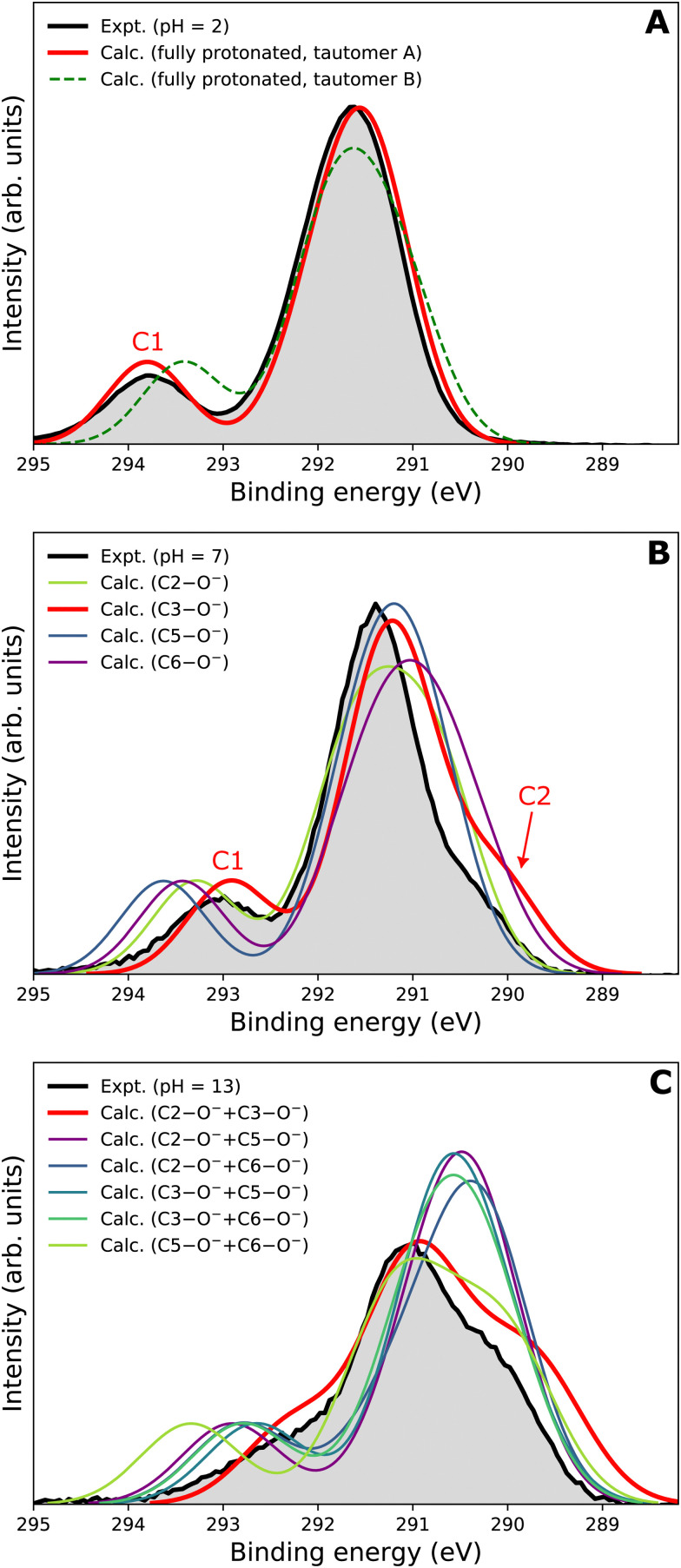
C 1s PE spectra of vitamin C at (A) pH = 2 (fully protonated), (B) pH = 7 (singly deprotonated), and (C) pH = 13 (doubly deprotonated); experimental spectra in black and calculated spectra in color as indicated in the legend. In panel A, spectra for two tautomers are modeled. In panels B and C, spectra for all possible deprotonation sites of tautomer A are modeled. The calculated spectrum with the best match is plotted in red in each panel. A quantification of the similarity between calculated and experimental spectra using KL (Kullback–Leibler) divergence can be found in the ESI† in Table S1.

Experimental and theoretical PE spectra for all three protonation states are presented in Fig. 3, panels A–C. Experimental spectra measured using a precise photon energy of 400.88 ± 0.02 eV are plotted in black and theoretical spectra are colored as indicated. We first focus on C 1s core-level PE spectra for pH = 2 (panel A) and pH = 7 (panel B), where the fully protonated and singly deprotonated forms of ascorbic acid are expected to be present.^39^ The results for pH = 13 (panel C), extending our approach to double deprotonation for the first time, are discussed later.

For a fully protonated molecule of vitamin C, we consider two tautomers (A and B, Fig. 2). The A form has been identified by NMR^38^ to be present in aqueous solution, and we show here that LJ-PES is capable of confirming this conclusion. The experimental PE spectrum for pH = 2 exhibits a main peak centered at BE = 291.6 eV and a smaller peak at BE = 293.8 eV. The experiment can be compared to our calculated spectra for the (fully protonated) A tautomer (red line) and the B tautomer (green dashed line). The modeled spectrum of the A tautomer agrees excellently with the experiment both in shape and position. From the theory, we can assign the smaller peak to ionization of the C1 site (see Fig. 1 and Table 1) while the main peak is from the combined signal of the other five carbon sites in the molecule. The main peak's position is in line with the previously published C 1s data for various systems containing the C–OH group ranging 291–291.5 eV, as mentioned in the Introduction, including the main peak of glucose C–OH centered at 291.5 eV.^31^ On the other hand, the modeled spectrum for the B tautomer exhibits discrepancies when compared to the experiment. With the positions of the smaller and main peaks at 293.4 eV and 291.6 eV, respectively, the energy separation between them is too small. Moreover, as the main peak is slightly broader, the relative intensities of the main and smaller peaks differ from the experiment. Therefore, we confirm that the A tautomer prevails in solution, and subsequent deprotonations will be considered for this structure.

**Table 1:** Carbon 1s binding energies (in eV) of individual atoms or atom groups extracted from experimental and calculated spectra at different pH values

Carbon atoms	Experiment	Calculation
	pH = 2	
C1	293.8	293.8
C2–C6	291.6	291.6
	pH = 7	
C1	293.0	292.9
C2	290.4	290.1
C3–C6	291.4	291.2
	pH = 13	
C1	292.4	292.3
C2–C3	290.1	289.8
C4–C6	291.1	291.0

Let us now focus on a singly deprotonated form of vitamin C, with the deprotonation site assigned to the C3–OH site by NMR.^38^ The pH = 7 PE spectrum (panel B, black line) exhibits similar main and C1 peaks, but their positions have clearly changed with respect to pH = 2, with their maxima at BE = 291.4 eV and BE = 293.0 eV, respectively. Moreover, a shoulder at BE = 290.4 eV appears, originating from the C2 1s electron according to our calculations (Table 1). These observations indicate that vitamin C exists in a different form at pH = 7 compared to pH = 2; the good match with theory indicates that, indeed, a singly deprotonated form is present, as discussed in the following. The ascorbic-acid molecule has four –OH groups (at carbons C2, C3, C5, and C6) that could, in principle, deprotonate at relatively low pH (Fig. 2). Therefore, we modeled spectra for all four hypothetical acid–base forms using the cluster-continuum solvation model; results are shown in panel B of Fig. 3 for C2 (green), C3 (red), C5 (blue), and C6 (purple). When comparing the calculated spectra to the experiment, only one modeled deprotonation site (C3–O^−^) reproduces the experimentally observed features, with the additional shoulder at ≈290 eV being the most distinct one. Our calculation predicts positions of the main and the smaller peaks of 291.2 eV and 292.9 eV, respectively, *i.e.*, an energetic distance of 1.7 eV, very close to the experimental energetic distance of 1.6 eV. Thus, we can safely assign the deprotonation center to the C3–OH site, consistent with the published NMR data.^38^

**Fig. 4:**
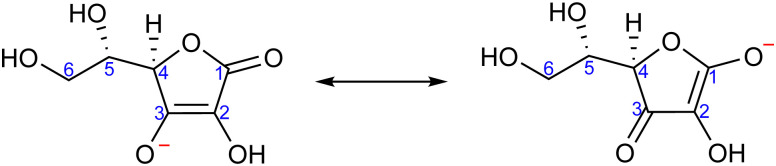
Resonance structures of the ascorbic-acid molecule singly deprotonated at the C3–OH site.

Interestingly, the high-BE peak originating from C1 is shifted by 0.8 eV upon the deprotonation at C3–OH, thus being influenced by an atom that is three chemical bonds away from the deprotonation site. A similar observation can be made for the C2 site, for which the chemical shift results in a distinguishable feature in the PE spectrum of the singly deprotonated molecule. This is well reproduced by our calculations and can be explained by two resonant structures,^38^ resulting in the –O^−^ group attached to either C3 or C1, as shown in Fig. 4. Importantly, analogous shifts for C1 and C2 signals are also observed in ^13^C NMR pH-dependent spectra.^38^ This demonstrates that site-specific changes affect not only the observable LJ-PES chemical shift of the site itself but also of other atoms at a distance of a few chemical bond lengths.

### 3.2 Extending the structural assignment to double deprotonation

Next, we investigate whether our approach is also capable of probing two different acid–base sites selectively at the same time. As for the single deprotonation, this question has not escaped the attention of NMR spectroscopists, concluding that the deprotonation takes place at C2–OH and C3–OH.^38^

Here, we investigate the PES spectrum for pH = 13, where double deprotonation of vitamin C is expected. The experimental spectrum (black) in panel C of Fig. 3 consists of a broad band with a maximum at BE = 291.1 eV, with a shoulder at BE = 290.1 eV, and another broad shoulder at BE = 292.4 eV. The spectrum is quite different from the one at pH = 7, and, in combination with our calculations (below), we find that the signal indeed comes from a doubly deprotonated molecule.

To interpret the experimental data, we again modeled all possible combinations of deprotonation for two sites to compare them with the experiment. We employed the cluster-continuum solvation model with 12 explicit water molecules placed around the deprotonated groups, as detailed in the Methods section. Calculated spectra for a total of six hypothetical combinations of double deprotonations (Fig. 2) are shown in panel C of Fig. 3. We observe that only one combination (C2–O^−^ + C3–O^−^) reproduces all the experimental features. It exhibits the lowest energy separation between the main peak and the high-BE shoulder, similar to the broad high-BE shoulder observed in the experiment. That spectrum also reproduces the low-BE shoulder with a similar height ratio. Also, the main peak's calculated position of 291.0 eV is in very good agreement with the measured spectrum (Table 1). Thus, our approach directly enables us to uniquely assign the two deprotonation centers in the molecule to C2–O^−^ and C3–O^−^. Our conclusion is in accordance with previously published results from NMR.^38^ To our knowledge, this is the very first time LJ-PES has been used to site-specifically probe double deprotonation acid–base sites. Although the double deprotonation of vitamin C has been previously probed by NMR, we highlight that LJ-PES augmented with theory can be used to derive the same structural information in this case, and in addition, promises to provide a plethora of complementary information, *e.g.*, on electronic structure, electronic decay channels, speciation or intermolecular interactions.^28,29,89^

### 3.3 Fast and reliable *ab initio* interpretation of the experimental data

**Fig. 5:**
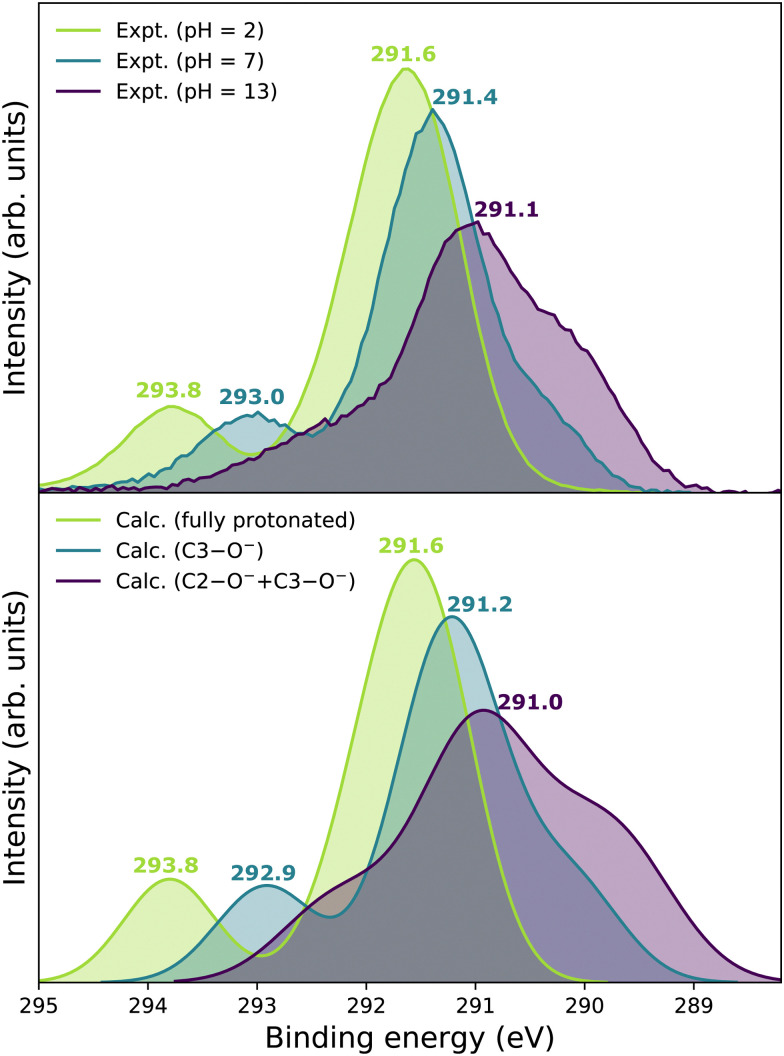
Upper panel: Summary of experimental C 1s PE spectra of vitamin C measured at pH = 2, pH = 7, and pH = 13. Lower panel: Summary of calculated C 1s LJ-PE spectra of vitamin C in its fully protonated, singly deprotonated, and doubly deprotonated form.

We now focus on the accuracy of our calculations. The so-far presented spectra were modeled using the maximum-overlap method based on a single structure for each protonation state. Therefore, the computations are inexpensive and relatively straightforward. Yet, they appear to be surprisingly accurate, as summarized in Table 1 and Fig. 5. All the peak positions are reproduced with an accuracy of 0.2 eV compared to the experiment, which has its own uncertainty of ≈0.1 eV. The small inaccuracies in our computations are attributed to multiple approximations, as described in the Calculations section, inevitably accompanying any theoretical effort to describe complex disordered systems of solvated molecules. We can compare our results to the typical accuracy of the state-of-the-art approach based on the equation-of-motion coupled clusters with single and double excitations (EOM-CCSD). With a small set of various gas-phase molecules, the reported mean average error (MAE) for calculated C 1s BEs was 0.68 eV, or 0.24 eV after shifting the values to eliminate systematic errors.^90^ For aqueous-phase glycine, the error was 0.05–0.22 eV when employing equilibrium averaging with an explicit solvent model based on EOM-CCSD with an effective fragment potential and solvent polarization correction.^90^ Our results thus indicate that the computational framework based on the maximum-overlap method combined with a suitably selected DFT approach is a powerful and efficient method for modeling core-level ionization. Although one could argue that the accuracy is based on an empirical combination of the CAM-B3LYP functional with a sufficiently large basis set and on possible cancellation of errors, we recommend this approach for modeling core-level electron ionization when aiming for high precision for a relatively low computational price. Before enough experimental data are collected to extract general trends, the above combination offers a pragmatic tool for the interpretation of the experimental data.

The spectra modeled in this work using a single optimized geometry are accurate; however, this approach is not generally applicable to all systems as a single minimal structure might be an insufficient representation of the overall distribution. A more robust technique, such as the nuclear-ensemble method,^75–77^ can be used to sample an ensemble of initial structures from molecular dynamics. To provide a comparison, we also employed NEM to model the spectra. Specifically, we sampled 100 geometries from 40 ps long QM/MM dynamics and used them as input to our calculations for constructing the PE spectra.

**Fig. 6:**
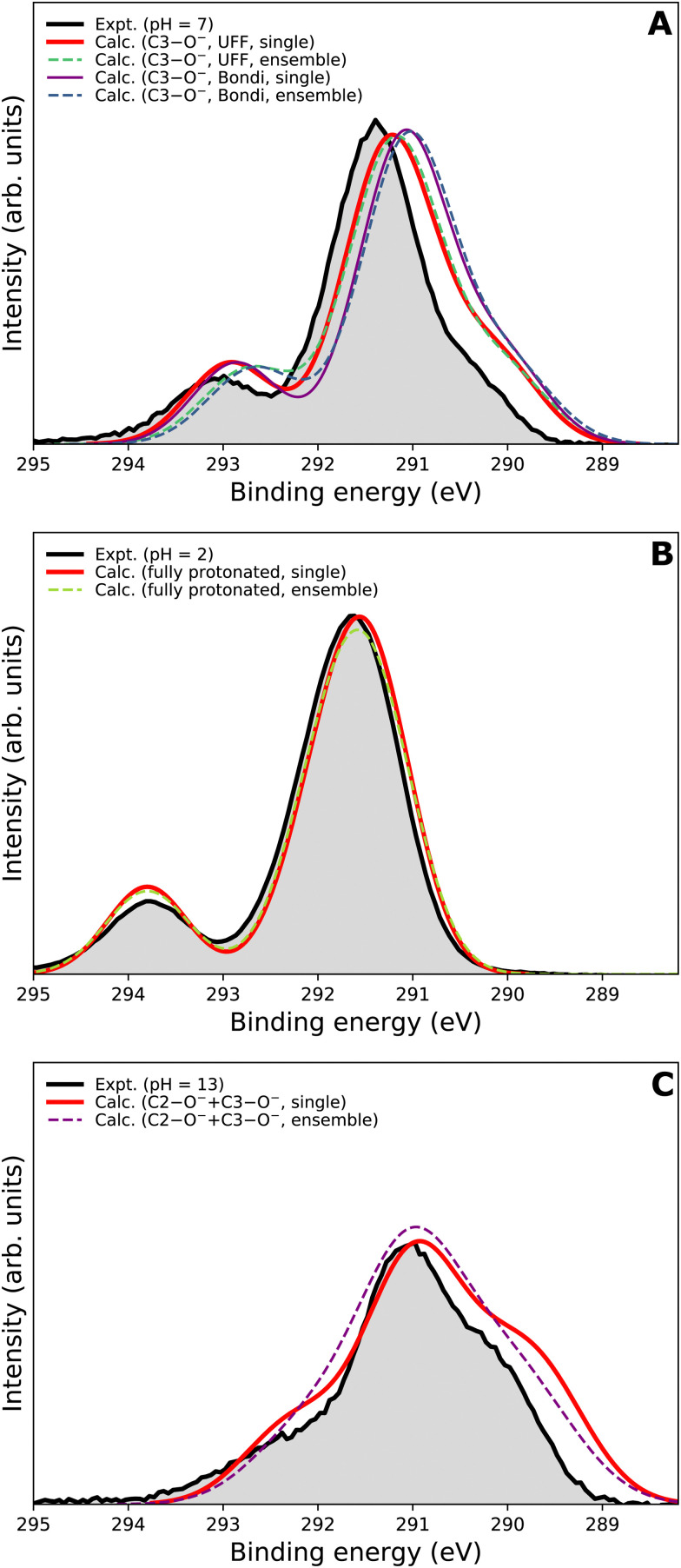
Comparison of different theoretical approaches to model C 1s PE spectra of vitamin C, and to the experimental PE spectra (black lines in each panel). Spectra from a single geometry optimized to its energetic minimum (‘single’) and from an ensemble of 100 structures sampled from molecular dynamics (‘ensemble’) are presented. (A) Calculated for single deprotonation/measured at pH = 7. Two PCM cavity radii specifications (‘UFF’ and ‘Bondi’) were tested for this case. (B) Calculated for full protonation/measured at pH = 2. (C) Calculated for double deprotonation/measured at pH = 13.

We first focus on the singly deprotonated form of vitamin C. Panel A of Fig. 6 shows the calculated spectra from a single structure (denoted as ‘single’) as well as from an ensemble of 100 structures from molecular dynamics (denoted as ‘ensemble’). In addition, we also compare two PCM cavity radii specifications (UFF^68^ and Bondi^91^) each to provide insight into how much those parameters influence the overall modeled spectrum. The differences in the four modeled spectra in panel A of Fig. 6 are not large overall but clearly follow an interesting pattern, which is also distinctive for the main peak and for the small high-BE peak. For the main peak, a difference in position of ≈0.15 eV is caused by changing the cavity specification. This is, from our experience, a typical behavior. Interestingly, the main peak's position remains the same regardless of using a single structure or an ensemble of structures. The single-geometry approximation is thus reasonable in the present case. This is distinct from the behavior of the high-BE peak. A difference of ≈0.25 eV is seen between the single-structure and ensemble models, irrespective of the PCM cavity model. The ensemble model provides a slightly less accurate (compared to the experiment) peak position in this particular case. While the nuclear-ensemble approach is an approximation, we attribute the worse agreement with the experiment to the insufficiency of our molecular dynamics used for the sampling. Particularly, we used the QM/MM method with a QM zone containing the ascorbic-acid molecule and an MM zone containing solvating water molecules. Thus, we interpret the discrepancy as an insufficient description of the explicit solvent structure around the vitamin C molecule. This claim is further supported by the spectrum of the fully protonated form in panel B of Fig. 6, where no explicit water molecules from QM/MM dynamics were used and where the ‘single’ and ‘ensemble’ spectra match in both peak positions. In fact, both are virtually identical, confirming that the single geometry is a good representation of the system distribution. Consequently, a single-structure spectrum based on optimization to the energetic minimum can provide a more reliable result in some cases, such as for the singly deprotonated form discussed above.

In panel C of Fig. 6, the results for the doubly deprotonated form of ascorbic acid are shown. Although the position of the maximum is very similar, the spectral shape is slightly different. Specifically, the spectrum obtained from an ensemble of structures lacks the low-BE and, to some extent, also the high-BE shoulder. We again attribute this to the insufficiently described solvation structure by the QM/MM molecular dynamics used for the sampling. Thus, the single-geometry spectrum again reproduces the experimental spectral features more closely. In summary, a single microhydrated structure optimized on a sufficiently high level of theory provides a more accurate spectrum than an ensemble of structures sampled from molecular dynamics with a lower level of theory.

We emphasize that the computational price for the ‘ensemble’ spectra in the presented case is about one hundred times higher than that of the spectra from a single geometry. Thus, we find that the single geometry is an inexpensive yet accurate approach to modeling LJ-PE spectra of carbon core-level electrons.

## 4 Conclusions

We have reported the pH-dependent C 1s liquid-jet PE spectra of vitamin C in water. By performing measurements at pH = 2, pH = 7, and pH = 13, we obtained data for three different acid–base forms. The peaks’ origin was interpreted by assigning contributions of specific carbon atoms using electronic-structure calculations.

We have demonstrated the applicability of a combined experimental–theoretical approach based on LJ-PES to probe tautomeric and acid–base molecular structure in aqueous solutions, using vitamin C as a prototype. A dominant tautomeric form of a fully protonated molecule has been determined. For the singly deprotonated molecule, we unequivocally assigned the deprotonation center to be C3–OH. Moreover, for the first time, the method has been extended to a double-deprotonation case, demonstrating the ability to selectively probe two acid–base centers simultaneously. This enabled us to identify the two deprotonation sites, C2–OH and C3–OH, in the double-deprotonated form of vitamin C in a highly alkaline solution.

These results are consistent with previous findings based on NMR spectroscopy. The two approaches thus provide comparable structural information. As NMR sometimes suffers from structural misassignments,^22–25^ there is room for the development of complementary structure-sensitive techniques. Furthermore, LJ-PES provides additional advantages as it simultaneously captures the full electronic structure and reflects molecular interactions, *e.g.*, with the neighboring solvent molecules.^28^ Note also that a single LJ-PES measurement can probe the structure from the perspective of different atomic species (*e.g.*, carbon and nitrogen) that can be helpful in the elucidation of complex structures. In the present case, probing the oxygen sites would be of little use due to the signal overlap with the aqueous solvent's oxygen. The LJ-PES technique can be conveniently expanded to studying air–water interfaces by varying the photon energy and thus the probing depth,^92^ exploring, *e.g.*, specific acid–base behavior and speciation at the interface, relevant for atmospheric chemistry.

An interesting phenomenon observed in the present study is that deprotonation affects BEs of core-level electrons of an atom that is a few chemical bonds away from the deprotonation site. This may be surprising, considering the enormous ability of water to screen the electrostatic effects.^93^ Our observation is promising for a possible application of multivariate analysis techniques to the measured data to reconstruct the full molecular structure from LJ-PES.

We have presented an efficient computational approach to model C 1s photoelectron spectra in aqueous solution. It is based on a single optimized geometry and employs the maximum-overlap method with the CAM-B3LYP functional. With this, we achieved an accuracy of 0.2 eV for the modeled BEs. The computational protocol is inexpensive and relatively straightforward. Therefore, we recommend this approach as the first choice for calculating core-level BEs in aqueous solutions.

The high accuracy of the computational protocol is in line with our previous studies. Note that the calculated BEs are close to the experiment on an absolute scale. This finding is somewhat surprising in the present case for two reasons. First, the deprotonated forms of the ascorbic acid contain an –O^−^ group with a highly concentrated charge. For calculations of the valence region, such a situation would usually require the inclusion of explicit solvent molecules in a large zone around the site.^50^ Second, the same computational treatment is required when dealing with multiply charged anions.^62^ In the present case, correct core-level energies are reproduced even with a minimalistic model assuming just a dielectric approximation of the environment and a few explicitly treated solvent molecules.

The present study further outlines the capabilities of liquid-phase photoemission spectroscopy for studying chemical and biochemical phenomena. In a broader sense, we attempt to bring LJ-PES's large potential to the awareness of experimental chemists. Both X-ray photoemission spectroscopy and nuclear magnetic resonance started to be developed in the 1950s, yet the former technique became almost exclusively a domain of solid-state physicists. We believe that LJ-PES could have played a more important role than NMR in an alternative history, and it is worth pursuing the paths it offers. The selectivity of the technique enables specific investigation of even complex systems,^28^ and a combination of information from different spectral regions provides full insight into the molecular structure in disordered systems. The need to interpret these data with the help of demanding electronic-structure calculations is one of the hurdles to a broader application of the technology. The present study focuses exclusively on the photoelectrons, yet further information can be retrieved from Auger and other second-order electrons (*e.g.*, electrons stemming from intermolecular Coulombic decay^89^). The latter spectra, however, have been so far only rarely used for structural determination, even though they contain important additional information.^29^ More studies are, therefore, needed to extract empirical trends to make the approach theory-free, *i.e.*, enable chemical and structural assignment on the basis of documented energies and spectral shapes.

The chemistry and acid–base properties of vitamin C are well-known. We used this system as a prototype with a large potential space of protonation states. We recall here that vitamin C was a favorite molecule of Linus Pauling, especially in his later years.^94,95^ While his views on the health effects of vitamin C were highly controversial, he is an undisputed titan of structural chemistry and the nature of a chemical bond.^96,97^ This work can be considered a homage to his lifelong achievements.

## Author contributions

Lukáš Tomaník: conceptualization, methodology, investigation, writing – original draft, writing – review & editing, visualization. Michele Pugini: methodology, validation, formal analysis, investigation, data curation, writing – review & editing. Karen Mudryk: methodology, formal analysis, investigation, writing – review & editing. Stephan Thürmer: software, formal analysis, writing – review & editing. Dominik Stemer: investigation. Bruno Credidio: investigation. Florian Trinter: investigation. Bernd Winter: conceptualization, resources, writing – original draft, writing – review & editing, supervision, project administration, funding acquisition. Petr Slavíček: conceptualization, resources, writing – original draft, writing – Review & editing, supervision, project administration, funding acquisition.

## Data availability

Cartesian coordinates of optimized structures, sample input for calculating core-level ionization energies, and Cartesian coordinates of structures sampled from QM/MM dynamics are available in the ESI.† The raw data relevant to this work has been deposited at DOI: 10.5281/zenodo.10706227.

## Conflicts of interest

There are no conflicts to declare.

## Supplementary Material

CP-026-D4CP01521E-s001

## References

[cit1] Faubel M., Steiner B., Toennies J. P. (1997). J. Chem. Phys..

[cit2] Winter B., Faubel M. (2006). Chem. Rev..

[cit3] Winter B. (2009). Nucl. Instrum. Methods Phys. Res., Sect. A.

[cit4] Seidel R., Winter B., Bradforth S. E. (2016). Annu. Rev. Phys. Chem..

[cit5] Siegbahn K. , ESCA. Atomic, Molecular and Solid State Structure Studied by Means of Electron Spectroscopy, Almqvist & Wiksells Uppsala, Uppsala, 1967

[cit6] Signorell R., Winter B. (2022). Phys. Chem. Chem. Phys..

[cit7] Dupuy R., Richter C., Winter B., Meijer G., Schlögl R., Bluhm H. (2021). J. Chem. Phys..

[cit8] Perrine K. A., Parry K. M., Stern A. C., Spyk M. H. C. V., Makowski M. J., Freites J. A., Winter B., Tobias D. J., Hemminger J. C. (2017). Proc. Natl. Acad. Sci. U. S. A..

[cit9] Ghosal S., Hemminger J. C., Bluhm H., Mun B. S., Hebenstreit E. L., Ketteler G., Ogletree D. F., Requejo F. G., Salmeron M. (2005). Science.

[cit10] Wu C. H., Weatherup R. S., Salmeron M. B. (2015). Phys. Chem. Chem. Phys..

[cit11] Malmgren S., Ciosek K., Hahlin M., Gustafsson T., Gorgoi M., Rensmo H., Edström K. (2013). Electrochim. Acta.

[cit12] Reuss T., Nair Lalithambika S. S., David C., Döring F., Jooss C., Risch M., Techert S. (2023). Acc. Chem. Res..

[cit13] Thürmer S., Malerz S., Trinter F., Hergenhahn U., Lee C., Neumark D. M., Meijer G., Winter B., Wilkinson I. (2021). Chem. Sci..

[cit14] Winter B., Hergenhahn U., Faubel M., Björneholm O., Hertel I. V. (2007). J. Chem. Phys..

[cit15] Winter B., Aziz E. F., Ottosson N., Faubel M., Kosugi N., Hertel I. V. (2008). J. Am. Chem. Soc..

[cit16] Hollas D., Pohl M. N., Seidel R., Aziz E. F., Slavček P., Winter B. (2017). Sci. Rep..

[cit17] Li J., Lu J., Chew A., Han S., Li J., Wu Y., Wang H., Ghimire S., Chang Z. (2020). Nat. Commun..

[cit18] Barreau L., Ross A. D., Garg S., Kraus P. M., Neumark D. M., Leone S. R. (2020). Sci. Rep..

[cit19] Nishitani J., West C. W., Suzuki T. (2017). Struct. Dyn..

[cit20] Yin Z., Chang Y.-P., Balčiūnas T., Shakya Y., Djorović A., Gaulier G., Fazio G., Santra R., Inhester L., Wolf J.-P., Wörner H. J. (2023). Nature.

[cit21] Ernst R. R. , Bodenhausen G. and Wokaun A., Principles of Nuclear Magnetic Resonance in One and Two Dimensions, Oxford University Press, 1990

[cit22] Yoo H.-D., Nam S.-J., Chin Y.-W., Kim M.-S. (2016). Arch. Pharm. Res..

[cit23] Shen S.-M., Appendino G., Guo Y.-W. (2022). Nat. Prod. Rep..

[cit24] Nicolaou K. C., Snyder S. A. (2005). Angew. Chem., Int. Ed..

[cit25] Suyama T. L., Gerwick W. H., McPhail K. L. (2011). Bioorg. Med. Chem..

[cit26] Aziz E. F., Ottosson N., Faubel M., Hertel I. V., Winter B. (2008). Nature.

[cit27] Ottosson N., Børve K. J., SpÅngberg D., Bergersen H., Sæthre L. J., Faubel M., Pokapanich W., Öhrwall G., Björneholm O., Winter B. (2011). J. Am. Chem. Soc..

[cit28] He L., Tomaník L., Malerz S., Trinter F., Trippel S., Belina M., Slavíček P., Winter B., Küpper J. (2023). J. Phys. Chem. Lett..

[cit29] Mudryk K., Lee C., Tomaník L., Malerz S., Trinter F., Hergenhahn U., Neumark D. M., Slavíček P., Bradforth S., Winter B. (2024). J. Am. Chem. Soc..

[cit30] Thürmer S., Seidel R., Winter B., Ončák M., Slavíček P. (2011). J. Phys. Chem. A.

[cit31] Malerz S., Mudryk K., Tomaník L., Stemer D., Hergenhahn U., Buttersack T., Trinter F., Seidel R., Quevedo W., Goy C., Wilkinson I., Thürmer S., Slavíček P., Winter B. (2021). J. Phys. Chem. A.

[cit32] Szent-Györgyi A. , Nobel Lecture, Nobel Foundation, 1937, https://www.nobelprize.org

[cit33] Institute of Medicine, Dietary Reference Intakes for Vitamin C, Vitamin E, Selenium, and Carotenoids, The National Academies Press, Washington, DC, 2000 25077263

[cit34] Davies M. , Partridge D. and Austin J., Vitamin C: Its Chemistry and Biochemistry, Royal Society of Chemistry, 2007

[cit35] Ems T. , St Lucia K. and Huecker M. R., Biochemistry, Iron Absorption, StatPearls Publishing, Treasure Island (FL), 2022 28846259

[cit36] Martıénez-Navarrete N., Camacho M., Martıénez-Lahuerta J., Martıénez-Monzó J., Fito P. (2002). Food Res. Int..

[cit37] Hirst E. L. , in The Structure and Synthesis of Vitamin C (Ascorbic Acid) and its Analogues, ed. L. Zechmeister, Y. Asahina, C. Dhéré, K. Freudenberg, C. R. Harington, E. L. Hirst, F. Kuffner, H. Rudy, E. Spath, G. Tóth and G. Zemplén, Springer, Vienna, Vienna, 1939, pp. 132–159

[cit38] Berger S. (1977). Tetrahedron.

[cit39] Birch T. W., Harris L. J. (1933). Biochem. J..

[cit40] Viefhaus J., Scholz F., Deinert S., Glaser L., Ilchen M., Seltmann J., Walter P., Siewert F. (2013). Nucl. Instrum. Methods Phys. Res., Sect. A.

[cit41] Malerz S., Haak H., Trinter F., Stephansen A. B., Kolbeck C., Pohl M., Hergenhahn U., Meijer G., Winter B. (2022). Rev. Sci. Instrum..

[cit42] Nguyen-Truong H. T. (2018). J. Phys.: Condens. Matter.

[cit43] Sodhi R. N., Brion C. (1984). J. Electron Spectrosc. Relat. Phenom..

[cit44] Pluhařová E., Slavíček P., Jungwirth P. (2015). Acc. Chem. Res..

[cit45] Runge E., Gross E. K. U. (1984). Phys. Rev. Lett..

[cit46] Muchová E., Slavček P. (2018). J. Phys.: Condens. Matter.

[cit47] Salzner U., Baer R. (2009). J. Chem. Phys..

[cit48] Krylov A. I. (2008). Annu. Rev. Phys. Chem..

[cit49] Feng X., Epifanovsky E., Gauss J., Krylov A. I. (2019). J. Chem. Phys..

[cit50] Ghosh D., Roy A., Seidel R., Winter B., Bradforth S., Krylov A. I. (2012). J. Phys. Chem. B.

[cit51] Aryasetiawan F., Gunnarsson O. (1998). Rep. Prog. Phys..

[cit52] Govoni M., Galli G. (2015). J. Chem. Theory Comput..

[cit53] Vidal M. L., Feng X., Epifanovsky E., Krylov A. I., Coriani S. (2019). J. Chem. Theory Comput..

[cit54] Vidal M. L., Krylov A. I., Coriani S. (2020). Phys. Chem. Chem. Phys..

[cit55] Gilbert A. T. B., Besley N. A., Gill P. M. W. (2008). J. Phys. Chem. A.

[cit56] Herbert J. M. (2021). Wiley Interdiscip. Rev.: Comput. Mol. Sci..

[cit57] Cammi R., Tomasi J. (1995). Int. J. Quantum Chem..

[cit58] Jagoda-Cwiklik B., Slavíček P., Cwiklik L., Nolting D., Winter B., Jungwirth P. (2008). J. Phys. Chem. A.

[cit59] Pliego J. R., Riveros J. M. (2001). J. Phys. Chem. A.

[cit60] Bryantsev V. S., Diallo M. S., Goddard III W. A. (2008). J. Phys. Chem. B.

[cit61] Tomaník L., Muchová E., Slavíček P. (2020). Phys. Chem. Chem. Phys..

[cit62] Pluhařová E., Ončák M., Seidel R., Schroeder C., Schroeder W., Winter B., Bradforth S. E., Jungwirth P., Slavíček P. (2012). J. Phys. Chem. B.

[cit63] Gordon M. S., Fedorov D. G., Pruitt S. R., Slipchenko L. V. (2012). Chem. Rev..

[cit64] Herbert J. M. (2019). J. Chem. Phys..

[cit65] Yanai T., Tew D. P., Handy N. C. (2004). Chem. Phys. Lett..

[cit66] Mennucci B., Tomasi J. (1997). J. Chem. Phys..

[cit67] Cancès E., Mennucci B., Tomasi J. (1997). J. Chem. Phys..

[cit68] Rappe A. K., Casewit C. J., Colwell K. S., Goddard W. A. I., Skiff W. M. (1992). J. Am. Chem. Soc..

[cit69] Frisch M. J. , Trucks G. W., Schlegel H. B., Scuseria G. E., Robb M. A., Cheeseman J. R., Scalmani G., Barone V., Mennucci B., Petersson G. A., Nakatsuji H., Caricato M., Li X., Hratchian H. P., Izmaylov A. F., Bloino J., Zheng G., Sonnenberg J. L., Hada M., Ehara M., Toyota K., Fukuda R., Hasegawa J., Ishida M., Nakajima T., Honda Y., Kitao O., Nakai H., Vreven T., Montgomery, Jr. J. A., Peralta J. E., Ogliaro F., Bearpark M., Heyd J. J., Brothers E., Kudin K. N., Staroverov V. N., Kobayashi R., Normand J., Raghavachari K., Rendell A., Burant J. C., Iyengar S. S., Tomasi J., Cossi M., Rega N., Millam J. M., Klene M., Knox J. E., Cross J. B., Bakken V., Adamo C., Jaramillo J., Gomperts R., Stratmann R. E., Yazyev O., Austin A. J., Cammi R., Pomelli C., Ochterski J. W., Martin R. L., Morokuma K., Zakrzewski V. G., Voth G. A., Salvador P., Dannenberg J. J., Dapprich S., Daniels A. D., Farkas Ö., Foresman J. B., Ortiz J. V., Cioslowski J. and Fox D. J., Gaussian 09 Revision D.01, Gaussian Inc., Wallingford CT, 2009

[cit70] Barca G. M. J., Gilbert A. T. B., Gill P. M. W. (2018). J. Chem. Theory Comput..

[cit71] Dunning T. H. (1989). J. Chem. Phys..

[cit72] Kendall R. A., Dunning T. H., Harrison R. J. (1992). J. Chem. Phys..

[cit73] Woon D. E., Dunning T. H. (1995). J. Chem. Phys..

[cit74] Epifanovsky E., Gilbert A. T. B., Feng X., Lee J., Mao Y., Mardirossian N., Pokhilko P., White A. F., Coons M. P., Dempwolff A. L., Gan Z., Hait D., Horn P. R., Jacobson L. D., Kaliman I., Kussmann J., Lange A. W., Lao K. U., Levine D. S., Liu J., McKenzie S. C., Morrison A. F., Nanda K. D., Plasser F., Rehn D. R., Vidal M. L., You Z.-Q., Zhu Y., Alam B., Albrecht B. J., Aldossary A., Alguire E., Andersen J. H., Athavale V., Barton D., Begam K., Behn A., Bellonzi N., Bernard Y. A., Berquist E. J., Burton H. G. A., Carreras A., Carter-Fenk K., Chakraborty R., Chien A. D., Closser K. D., Cofer-Shabica V., Dasgupta S., de Wergifosse M., Deng J., Diedenhofen M., Do H., Ehlert S., Fang P.-T., Fatehi S., Feng Q., Friedhoff T., Gayvert J., Ge Q., Gidofalvi G., Goldey M., Gomes J., González-Espinoza C. E., Gulania S., Gunina A. O., Hanson-Heine M. W. D., Harbach P. H. P., Hauser A., Herbst M. F., Hernández Vera M., Hodecker M., Holden Z. C., Houck S., Huang X., Hui K., Huynh B. C., Ivanov M., Jász Á., Ji H., Jiang H., Kaduk B., Kähler S., Khistyaev K., Kim J., Kis G., Klunzinger P., Koczor-Benda Z., Koh J. H., Kosenkov D., Koulias L., Kowalczyk T., Krauter C. M., Kue K., Kunitsa A., Kus T., Ladjánszki I., Landau A., Lawler K. V., Lefrancois D., Lehtola S., Li R. R., Li Y.-P., Liang J., Liebenthal M., Lin H.-H., Lin Y.-S., Liu F., Liu K.-Y., Loipersberger M., Luenser A., Manjanath A., Manohar P., Mansoor E., Manzer S. F., Mao S.-P., Marenich A. V., Markovich T., Mason S., Maurer S. A., McLaughlin P. F., Menger M. F. S. J., Mewes J.-M., Mewes S. A., Morgante P., Mullinax J. W., Oosterbaan K. J., Paran G., Paul A. C., Paul S. K., Pavošević F., Pei Z., Prager S., Proynov E. I., Rák Á., Ramos-Cordoba E., Rana B., Rask A. E., Rettig A., Richard R. M., Rob F., Rossomme E., Scheele T., Scheurer M., Schneider M., Sergueev N., Sharada S. M., Skomorowski W., Small D. W., Stein C. J., Su Y.-C., Sundstrom E. J., Tao Z., Thirman J., Tornai G. J., Tsuchimochi T., Tubman N. M., Veccham S. P., Vydrov O., Wenzel J., Witte J., Yamada A., Yao K., Yeganeh S., Yost S. R., Zech A., Zhang I. Y., Zhang X., Zhang Y., Zuev D., Aspuru-Guzik A., Bell A. T., Besley N. A., Bravaya K. B., Brooks B. R., Casanova D., Chai J.-D., Coriani S., Cramer C. J., Cserey G., DePrince I., Eugene A., DiStasio J., Robert A., Dreuw A., Dunietz B. D., Furlani T. R., Goddard I., William A., Hammes-Schiffer S., Head-Gordon T., Hehre W. J., Hsu C.-P., Jagau T.-C., Jung Y., Klamt A., Kong J., Lambrecht D. S., Liang W., Mayhall N. J., McCurdy C. W., Neaton J. B., Ochsenfeld C., Parkhill J. A., Peverati R., Rassolov V. A., Shao Y., Slipchenko L. V., Stauch T., Steele R. P., Subotnik J. E., Thom A. J. W., Tkatchenko A., Truhlar D. G., Van Voorhis T., Wesolowski T. A., Whaley K. B., Woodcock I., Lee H., Zimmerman P. M., Faraji S., Gill P. M. W., Head-Gordon M., Herbert J. M., Krylov A. I. (2021). J. Chem. Phys..

[cit75] Crespo-Otero R., Barbatti M. (2012). Theor. Chem. Acc..

[cit76] Sršeň Š., Sita J., Slavíček P., Ladányi V., Heger D. (2020). J. Chem. Theory Comput..

[cit77] Prlj A., Marsili E., Hutton L., Hollas D., Shchepanovska D., Glowacki D. R., Slavíček P., Curchod B. F. E. (2022). ACS Earth Space Chem..

[cit78] Dierking C. W., Zurheide F., Zeuch T., Med J., Parez S., Slavíček P. (2017). J. Chem. Phys..

[cit79] Sršeň Š., Slavíček P. (2021). J. Chem. Theory Comput..

[cit80] Grimme S. (2006). J. Comput. Chem..

[cit81] Jorgensen W. L., Chandrasekhar J., Madura J. D., Impey R. W., Klein M. L. (1983). J. Chem. Phys..

[cit82] Nosé S. (1984). J. Chem. Phys..

[cit83] Hoover W. G. (1985). Phys. Rev. A: At., Mol., Opt. Phys..

[cit84] Rubešová M., Jurásková V., Slavíček P. (2017). J. Comput. Chem..

[cit85] Martínez L., Andrade R., Birgin E. G., Martínez J. M. (2009). J. Comput. Chem..

[cit86] Hollas D. , Suchan J., Svoboda O., Ončák M. and Slavíček P., ABIN, v1.0, 2020

[cit87] Ufimtsev I. S., Martinez T. J. (2009). J. Chem. Theory Comput..

[cit88] Titov A. V., Ufimtsev I. S., Luehr N., Martinez T. J. (2013). J. Chem. Theory Comput..

[cit89] Jahnke T., Hergenhahn U., Winter B., Dörner R., Frühling U., Demekhin P. V., Gokhberg K., Cederbaum L. S., Ehresmann A., Knie A., Dreuw A. (2020). Chem. Rev..

[cit90] Sadybekov A., Krylov A. I. (2017). J. Chem. Phys..

[cit91] Bondi A. (1964). J. Phys. Chem..

[cit92] Thürmer S., Seidel R., Faubel M., Eberhardt W., Hemminger J. C., Bradforth S. E., Winter B. (2013). Phys. Rev. Lett..

[cit93] Pohl M. N., Muchová E., Seidel R., Ali H., Sršeň Š., Wilkinson I., Winter B., Slavíček P. (2019). Chem. Sci..

[cit94] Pauling L. (1968). Science.

[cit95] Pauling L. , How to Live Longer and Feel Better, Avon, 1987

[cit96] Pauling L. , Nobel Lecture, Nobel Foundation, 1954, https://www.nobelprize.org

[cit97] Pauling L. , The Nature of the Chemical Bond, Cornell University Press, Ithaca, NY, 1960

