# Supplementary material for: Liquid-jet photoemission spectroscopy as a structural tool: site-specific acid–base chemistry of vitamin C
Source: Phys Chem Chem Phys. 2024 Jun 28;26(29):19673–84. doi: 10.1039/d4cp01521e (PMC11267885; doi:10.1039/d4cp01521e)
Supplement: CP-026-D4CP01521E-s001 [file CP-026-D4CP01521E-s001.pdf]

Electronic Supplementary Material (ESI) for Physical Chemistry Chemical Physics.
This journal is © the Owner Societies 2024

Electronic Supplementary Information: Liquid-Jet Photoemission Spectroscopy as a Structural Tool: Site-Specific Acid-Base Chemistry of Vitamin C

Lukáš Tomaník,[a‡] Michele Pugini,[b] Karen Mudryk,[b] Stephan Thürmer,[c] Dominik Stemer,[b] Bruno Credidio,[b] Florian Trinter,[b] Bernd Winter,[b§] and Petr Slavíček[a¶]

1 Comparison of spectra measured at 400.88 eV and 850 eV photon energy

In Fig. S1, we compare liquid-jet photoemission spectra measured at 400.88 eV photon energy presented in the main text with additional measurements using 850 eV photon energy. The respective kinetic energies of emitted photoelectrons are approx. 105 eV and 455 eV (plus 64 eV from bias), translating into probing of 3–4 and 6 solution layers, respectively. The spectral shapes measured at two different photon energies are very similar, providing essentially identical fitted peak positions. The small discrepancies are attributed to experimental differences in the two measurement campaigns, such as alignment and settings.

2 Evaluation of the agreement between experimental and theoretical spectra using Kullback–Leibler (KL) divergence

We provide a quantitative evaluation of the agreement between our modeled and measured liquid-jet photoelectron spectra of vitamin C solutions at different pH values. We use Kullback–Leibler (KL) divergence, measuring how much is one probability distribution different form another. Specifically, we determine how well is the experimental spectrum represented by a calculated one. The lower the KL divergence value, the better represented the spectrum is, with the KL divergence of 0 corresponding to two identical spectra. The calculated values are presented in Tab. S1, confirming our assignment in the main text, *i.e.*, the calculated spectra shown in red in Fig. 3 having the best agreement with the experiment. However, in comparing the experimental spectrum at pH = 7 with modeled spectra for the deprotonation at C2–O^- and C3–O^-, we got a lower KL divergence value for C2–O^- (0.040 vs. 0.071). Nevertheless, we argue that C3–O^- is the real deprotonation center based on examining the similarity in spectral features: the magnitude of separation between the main and the smaller peak and the appearance of the low-energy shoulder. We show in Tab. S1 in parentheses for those two cases how the results change in favor of C3–O^- if we shift the experimental spectrum by −0.2 eV to match the position of the main peak with those two calculated spectra.

Table S1 KL divergence values for comparing the agreement between an experimental spectrum at the indicated pH value and the respective calculated spectrum. Values in parentheses are contained using an experimental spectrum at pH = 7 shifted by −0.2 eV.

Calculated spectrum	KL divergence
pH = 2	
A tautomer	0.014
B tautomer	0.059
pH = 7	
C2–O^-	0.040 (0.073)
C3–O^-	0.071 (0.016)
C5–O^-	0.109
C6–O^-	0.130
pH = 13	
C2–O^- + C3–O^-	0.070
C2–O^- + C5–O^-	0.161
C2–O^- + C6–O^-	0.119
C3–O^- + C5–O^-	0.108
C3–O^- + C6–O^-	0.094
C5–O^- + C6–O^-	0.123

3 Cartesian coordinates of optimized structures

3.1 Fully protonated molecule of vitamin C

A tautomer

C	1.833065	0.670477	0.707193
C	0.915838	-0.103747	-0.199674
C	-0.142393	-0.527446	0.501520
C	0.011848	-0.077940	1.874804
O	1.169465	0.625502	1.985642
O	1.133648	-0.317669	-1.502978
O	-1.200485	-1.257628	0.080709
O	-0.734449	-0.269994	2.812072
C	2.049954	2.135344	0.308542
C	3.016700	2.822966	1.261162
O	3.323867	4.099451	0.721887
O	0.817423	2.817154	0.193479
H	-1.796575	-1.407220	0.835359
H	2.797826	0.163969	0.817201
H	2.559015	2.918376	2.254106
H	3.923980	2.213324	1.356227
H	3.905842	4.568354	1.337646
H	2.492619	2.155374	-0.692935
H	0.440798	2.947805	1.078745
H	2.003936	0.002835	-1.789838

B tautomer

O	1.878940	0.590637	0.781638
C	0.991198	-0.124044	-0.118663
C	-0.166194	-0.573402	0.766009
C	0.152485	-0.135366	2.079718
C	1.337755	0.526879	2.007657

[a] *Department of Physical Chemistry, University of Chemistry and Technology, Technická 5, 16628 Prague, Czech Republic.*
[b] *Molecular Physics, Fritz-Haber-Institut der Max-Planck-Gesellschaft, Faradayweg 4-6, 14195 Berlin, Germany.*
[c] *Department of Chemistry, Graduate School of Science, Kyoto University, Kitashirakawa-Oiwakecho, Sakyo-Ku, 606-8502 Kyoto, Japan.*
‡ Current address: Molecular Physics, Fritz-Haber-Institut der Max-Planck-Gesellschaft, Faradayweg 4-6, 14195 Berlin, Germany.
§ winter@fhi-berlin.mpg.de
¶ Petr.Slavicek@vscht.cz

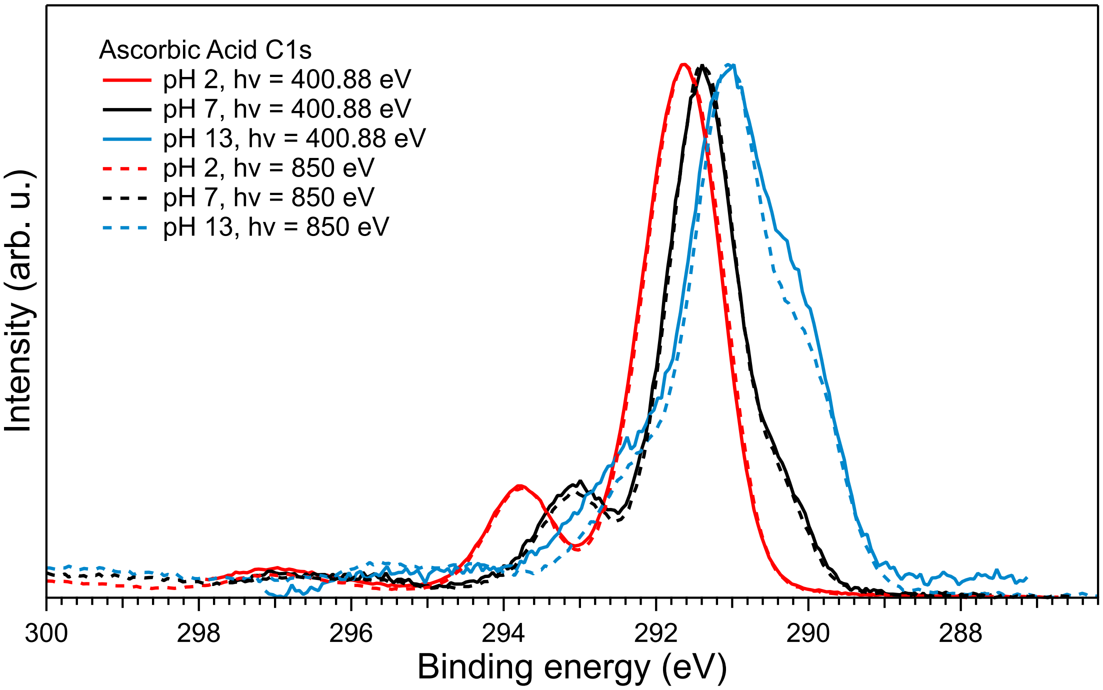

Fig. S1 Comparison of C 1s liquid-jet photoemission spectra of aqueous solutions of vitamin C (ascorbic acid) measured using two different photon energies, 400.88 eV and 850 eV. Results for solutions pH values of 2, 7, and 13 are shown.

C	0.570195	0.794845	-1.260077
O	-0.099885	1.939160	-0.762650
O	-1.159107	-1.194055	0.383904
O	-0.592567	-0.321155	3.204582
O	1.995918	1.104385	2.990715
C	1.742088	1.147612	-2.162276
O	1.230366	1.836078	-3.293547
H	-1.387190	-0.824963	2.958354
H	1.539774	-0.993303	-0.490759
H	2.461053	1.774468	-1.618181
H	2.250879	0.224559	-2.466480
H	1.967822	2.085874	-3.869228
H	-0.173679	0.246519	-1.846717
H	0.544829	2.526232	-0.336017
H	2.822057	1.507285	2.666294

3.2 Singly deprotonated molecule of vitamin C with six explicit water molecules

Deprotonated at C2–O^{-}

O	1.844723	-0.286673	0.575607
C	0.650470	-0.226850	-0.221966
C	-0.443520	-0.482616	0.767605
C	0.051710	-0.642734	2.011771
C	1.514811	-0.521557	1.879217
C	0.593265	1.138796	-0.919843
O	0.649051	2.194543	0.026480
O	-1.757352	-0.509257	0.440683
O	-0.557426	-0.872713	3.165756
O	2.381108	-0.606555	2.728369
C	1.678086	1.264921	-1.977234
O	1.450613	2.467196	-2.696815
H	0.712700	-1.020642	-0.975242
H	2.666898	1.276107	-1.500518
H	1.627179	0.396862	-2.646150
H	2.152384	2.576963	-3.354938
H	-0.378969	1.239377	-1.412818
H	1.527971	2.207566	0.440169
H	-1.894942	-0.554158	-0.518592
H	-1.021795	0.618684	3.811167
O	-1.369803	1.507260	4.106930
H	-0.807525	1.793067	4.842445
H	-1.317423	2.610540	2.636084
O	-1.319656	3.188487	1.843665
H	-0.676932	2.792730	1.224081
H	-3.191315	1.150481	4.464638
O	-4.076216	0.732176	4.462953
H	-3.935938	-0.065010	3.906147
H	-2.250738	-1.255037	3.004375
O	-3.221526	-1.417435	2.877293
H	-3.350388	-1.229220	1.934195
H	-0.492588	-2.806939	3.452553
O	-0.171390	-3.636595	3.860302
H	0.469017	-3.290684	4.510836

H	0.527703	-1.164366	4.583697
O	1.201657	-1.516986	5.212734
H	2.021383	-1.344843	4.719316

Deprotonated at C3–O$^-$

C	1.330033	0.016732	1.892220
C	1.825876	-0.384786	0.685862
C	0.748788	-0.831888	-0.155158
O	-0.436635	-0.702261	0.534031
C	-0.173701	-0.146958	1.834593
O	3.134941	-0.409610	0.273260
O	0.751311	-1.275980	-1.290767
C	-0.948766	1.163658	1.983968
O	-0.639180	2.051953	0.912967
O	1.944364	0.476888	2.934476
C	-2.444614	0.924009	2.090912
O	-3.071984	2.163732	2.386960
O	3.742323	1.981881	1.345920
H	3.521150	0.481208	0.487891
H	-0.512065	-0.868056	2.585802
H	-2.827733	0.508790	1.149587
H	-2.630183	0.195826	2.890344
H	-4.030134	2.029203	2.424341
H	-0.603790	1.668144	2.890358
H	-0.941188	1.654903	0.078829
H	3.072914	2.613251	0.987808
H	3.312717	1.615795	2.147338
H	0.849755	3.031677	0.646629
O	1.662515	3.540252	0.425608
H	1.570567	4.396781	0.868638
H	1.521489	2.169516	3.672201
O	1.313237	3.020410	4.107978
H	1.176156	2.790239	5.051936
H	1.522594	-0.126800	4.531887
O	1.396545	-0.389112	5.477973
H	0.660180	-1.018491	5.492435
H	3.463497	-0.745743	3.095383
O	4.140513	-1.388722	2.803780
H	4.057529	-1.342476	1.833035
H	1.623006	2.200789	7.344756
O	0.925625	2.000622	6.703161
H	1.096598	1.079514	6.386603

Deprotonated at C5–O$^-$

O	1.982086	0.978682	0.890931
C	1.405661	-0.113566	0.143700
C	0.399305	-0.696189	1.092453
C	0.361978	0.037959	2.208587
C	1.355584	1.095306	2.082788
C	0.787026	0.419471	-1.169244
O	-0.168665	1.391465	-0.927966
O	-0.398133	-1.742633	0.805471
O	-0.413982	-0.112845	3.305206
O	1.624570	1.958924	2.894684
C	1.927782	0.874897	-2.082562
O	1.398317	1.242100	-3.363002
H	-0.213517	0.607843	3.928022
H	2.214798	-0.820571	-0.069286
H	2.447342	1.735183	-1.646280
H	2.647683	0.059237	-2.204676
H	2.020532	0.969339	-4.052385
H	0.337638	-0.463994	-1.663343
H	-0.070908	-2.250892	0.044332
H	-1.135797	2.191761	0.539341
O	-1.944540	2.412803	1.049735
H	-2.493608	1.622711	0.877542
H	0.536415	2.883412	-0.675195
O	0.883064	3.824621	-0.617463
H	1.683663	3.782573	-0.074181
H	1.164896	3.074800	-3.513363
O	0.876990	4.003201	-3.378781
H	0.991425	4.136305	-2.409850
H	-1.080519	2.167538	-2.197862
O	-1.591874	2.764013	-2.807930
H	-0.891389	3.342162	-3.178061
H	-2.715152	3.663513	-0.107556
O	-3.024062	4.180677	-0.881766
H	-2.563702	3.746031	-1.636829
H	-1.645921	0.502621	-0.688443
O	-2.498869	0.067674	-0.412095
H	-2.223957	-0.746249	0.039460

Deprotonated at C6–O$^-$

O	1.841074	0.232596	0.874856
C	0.815771	-0.008675	-0.106698
C	-0.393680	-0.385339	0.705990
C	-0.077399	-0.303381	2.007029
C	1.313824	0.088286	2.114677
C	0.596217	1.219925	-0.985211
O	0.327148	2.320887	-0.137035
O	-1.567036	-0.703819	0.170339
O	-0.861265	-0.534011	3.092671
O	1.963190	0.260100	3.131067
C	1.746704	1.468046	-1.967853
O	1.336222	2.256150	-3.060682
H	-0.323042	-0.398775	3.891838
H	1.147695	-0.846261	-0.728016
H	2.576104	1.943547	-1.421257
H	2.110214	0.489709	-2.324615
H	-0.293827	0.996268	-1.587868
H	0.058626	3.082565	-0.699222
H	-1.522782	-0.801714	-0.824186
H	0.001399	1.439246	-3.738816
O	-0.660315	0.840267	-4.193852
H	-0.157516	0.518364	-4.962938
H	0.418093	3.517531	-2.583766
O	-0.116023	4.215188	-2.087339
H	-0.998746	4.223764	-2.485779
H	2.498959	3.299937	-3.507289
O	3.240725	3.894387	-3.850195
H	2.955451	4.807250	-3.699259
H	3.219811	3.598745	-7.048803

O	3.619208	3.006956	-6.394592
H	3.514771	3.454687	-5.516209
H	1.848106	1.423430	-4.548479
O	1.980645	0.934037	-5.398118
H	2.535917	1.538311	-5.934331
H	-1.199246	-0.404688	-3.140745
O	-1.461500	-1.094616	-2.470973
H	-2.269884	-1.513119	-2.803224

3.3 Doubly deprotonated molecule of vitamin C with 12 explicit water molecules

Deprotonated at C2–O$^-$ and C3–O$^-$

O	0.041558	-0.353340	1.953593
C	1.066057	0.648626	1.825276
C	1.343638	0.735095	0.343174
C	0.538606	-0.159436	-0.305178
C	-0.256181	-0.838143	0.702603
C	0.569242	1.943351	2.471599
O	-0.643035	2.381104	1.877712
O	2.267640	1.543654	-0.114416
O	0.416585	-0.447573	-1.608254
O	-1.088974	-1.725119	0.577269
C	0.440041	1.792513	3.979256
O	0.171679	3.074597	4.535266
O	2.456388	-4.009308	-0.151691
O	2.289889	-0.914382	-3.527011
O	4.951939	-0.252658	-3.006235
O	0.930258	-3.034986	-2.181145
O	-2.189494	-0.437165	-2.574731
O	2.057777	3.057802	-2.298016
O	3.694566	3.278360	1.362109
O	-0.348455	1.776433	-2.988343
O	6.086072	2.024111	1.089442
O	4.182651	-2.205707	1.134163
O	4.611474	0.167693	-0.325311
H	1.953666	0.291133	2.360405
H	-0.369381	1.090923	4.218436
H	1.377942	1.389926	4.382761
H	0.047968	2.981637	5.490977
H	1.302308	2.727738	2.264594
H	-1.298589	1.670402	1.968983
H	4.642596	0.570126	-3.442988
H	4.936046	-0.065585	-2.036935
H	2.128910	2.494716	-1.473319
H	2.262686	3.967856	-2.037670
H	3.105894	2.668593	0.835987
H	3.626673	4.150774	0.946681
H	6.422723	1.729563	1.948563
H	5.282470	2.572683	1.277590
H	4.318947	-1.400897	0.589864
H	3.577642	-2.790735	0.629467
H	3.746996	0.654433	-0.293554
H	5.245368	0.741502	0.167581
H	1.731503	-0.621248	-2.760213
H	3.225606	-0.902050	-3.223223
H	1.904244	-3.677788	-0.908156
H	2.914170	-4.797380	-0.478836
H	0.585684	-2.160690	-1.850893
H	1.531581	-2.738945	-2.891189
H	-1.320664	-0.598141	-2.133211
H	-2.070426	0.447293	-2.959012
H	0.004766	0.999462	-2.483428
H	0.346742	2.454656	-2.883671
H	2.737033	0.812045	-4.270153
O	3.303894	1.610907	-4.326936
H	2.929896	2.222154	-3.653667

Deprotonated at C2–O$^-$ and C5–O$^-$

C	1.892663	0.317759	0.960959
O	1.079259	-0.192794	0.009766
C	-0.159899	-0.634723	0.610590
C	0.000602	-0.267906	2.057085
C	1.217508	0.276772	2.267349
C	-1.365735	-0.048673	-0.143482
C	-1.241389	-0.451076	-1.616071
O	-2.517149	-0.313098	-2.256648
O	-0.885877	-0.606321	3.014373
O	1.793491	0.711399	3.385841
O	3.012338	0.722173	0.689563
O	-1.550274	1.319215	0.037468
O	1.555039	4.442794	1.385381
O	2.461585	4.292349	5.015646
O	0.253442	2.619917	4.392008
O	1.558009	-1.180306	5.515172
O	4.029275	-0.657126	4.198463
O	3.321897	2.814288	2.726019
H	-0.185807	-1.726375	0.504172
H	-0.503900	0.191426	-2.109939
H	-0.912015	-1.494008	-1.695669
H	-2.463556	-0.716317	-3.135568
H	-2.231503	-0.614568	0.252880
H	-1.813267	-0.259502	2.867751
H	2.771042	2.026096	2.999959
H	3.847965	2.458547	1.990380
H	2.210070	3.889851	1.877985
H	2.044336	5.212671	1.059856
H	3.030421	3.957978	4.298381
H	1.623373	3.799590	4.887043
H	0.794160	1.830046	4.093892
H	-0.256983	2.323411	5.160964
H	1.347866	-0.519007	4.822998
H	2.510327	-1.324258	5.361017
H	3.310246	-0.129192	3.769707
H	4.407255	-1.212752	3.501270

H	-2.198477	2.265982	-1.387154
O	-2.671994	2.510433	-2.217999
H	-2.878986	1.618621	-2.553186
H	-3.235739	1.305935	0.275128
O	-4.187790	1.126350	0.535663
H	-4.640140	1.981646	0.502698
H	-3.780152	0.606285	2.206447
O	-3.264290	0.580132	3.046407
H	-2.807470	1.458278	3.020869
H	-1.516450	2.205984	1.568618
O	-1.742913	2.731695	2.381903
H	-0.965891	2.721220	2.980475
H	-0.259565	2.381381	-0.419027
O	0.369026	3.086059	-0.735012
H	0.777022	3.507880	0.053130
H	1.478717	2.615895	-1.963907
O	2.113078	2.368348	-2.685129
H	1.850036	2.894248	-3.453604

Deprotonated at C2–O^{-} and C6–O^{-}

C	1.744470	0.670955	0.760540
O	0.989772	0.050420	-0.188838
C	-0.305677	-0.248282	0.354819
C	-0.211961	0.212331	1.776427
C	0.991186	0.775386	2.020441
C	-1.412284	0.430862	-0.448787
C	-1.623412	-0.199260	-1.830186
O	-2.912274	0.064699	-2.336636
O	-1.243063	0.100788	2.640130
O	1.491998	1.332024	3.115889
O	2.879407	1.036611	0.512083
O	-1.139212	1.820371	-0.502115
O	-4.576157	-1.586278	-5.700791
O	-4.095844	-2.333752	-3.019269
O	-3.205494	-1.526443	1.764091
O	-3.399485	2.605091	-1.888065
O	-2.906776	0.420988	-4.941362
O	-4.592285	-0.987966	-0.508478
H	-0.444108	-1.333526	0.293215
H	-0.843626	0.180128	-2.509053
H	-1.472851	-1.288018	-1.738116
H	-2.335411	0.263274	0.122028
H	-1.914552	2.268329	-0.907818
H	-1.975404	-0.473905	2.286266
H	-3.999675	-0.489084	-1.143711
H	-4.837415	-1.773534	-1.029435
H	-3.334595	1.623082	-2.112603
H	-4.203049	2.715573	-1.358877
H	-2.886594	0.333061	-3.933892
H	-3.140198	1.340724	-5.133937
H	-5.466157	-1.261295	-5.900792
H	-4.020383	-0.783072	-5.529420
H	-3.607848	-1.488610	-2.853801
H	-4.393919	-2.254968	-3.949822
H	-3.752140	-1.352265	0.949975
H	-3.827628	-1.748781	2.472861
H	1.162237	0.728509	4.730975
O	0.964408	0.698690	5.703125
H	0.151396	1.232051	5.757875
H	3.015119	2.964331	5.327600
O	3.219540	2.298717	6.024924
H	2.391625	1.771383	6.106470
H	3.233909	0.886295	3.418461
O	4.134119	0.811511	3.823084
H	3.994819	1.191905	4.719957
H	4.983385	2.160603	3.075883
O	5.226137	3.048130	2.704670
H	5.997223	3.351621	3.205903
H	2.215607	2.951604	3.315609
O	2.675449	3.749791	3.674594
H	3.574945	3.712370	3.282603
H	0.025875	2.285274	3.793321
O	-0.845837	2.455960	4.216753
H	-1.398449	1.759906	3.818214

Deprotonated at C3–O^{-} and C5–O^{-}

C	1.756268	0.380589	2.296165
C	2.248465	0.158201	0.883385
C	1.412511	-0.747417	0.301999
C	0.411201	-1.151243	1.255080
O	0.599002	-0.468390	2.422123
O	3.315575	0.742330	0.427789
O	1.460398	-1.361280	-0.922829
O	-0.487188	-1.973844	1.149450
C	1.418535	1.830164	2.670675
C	1.042236	1.890647	4.150064
O	1.145911	3.249187	4.605421
O	0.446943	2.402829	1.853320
O	2.950717	2.266119	-1.733381
O	3.986307	4.979670	1.730154
O	5.127509	2.408114	1.739997
O	3.506320	-3.340540	-0.644593
O	4.951981	-0.917756	-0.925224
O	3.034617	-0.255519	-2.831732
H	1.985798	-0.843241	-1.589347
H	2.512419	0.000994	2.994217
H	0.017289	1.528739	4.288211
H	1.724029	1.269580	4.742466
H	1.013782	3.260184	5.565087
H	2.379564	2.368241	2.575965
H	3.012180	0.717406	-2.697097
H	3.882372	-0.533090	-2.423280
H	3.162606	1.783060	-0.886381
H	3.609620	2.969234	-1.835923
H	4.370777	5.562669	1.059258
H	3.013676	4.950854	1.543078
H	4.458546	1.813608	1.335080
H	4.728464	3.304646	1.724188

H	2.664934	-2.851610	-0.740660
H	4.175950	-2.628952	-0.697220
H	4.444927	-0.286291	-0.344887
H	5.894287	-0.726060	-0.808002
H	-0.930122	3.219438	2.579441
O	-1.783991	3.658683	2.830252
H	-1.491105	4.531704	3.157650
H	0.451203	4.886339	4.040729
O	0.045748	5.676664	3.625690
H	0.442657	5.674476	2.729036
H	0.977871	3.890989	1.563081
O	1.293656	4.820721	1.299166
H	1.066950	4.855387	0.353511
H	0.490623	2.625024	0.096111
O	0.420233	3.004377	-0.818880
H	1.273104	2.793736	-1.255313
H	-1.085848	2.159231	-1.435026
O	-1.936670	1.669757	-1.480814
H	-2.106988	1.461918	-0.535525
H	-1.077818	1.480525	1.496541
O	-2.031492	1.295504	1.295336
H	-2.481440	2.060572	1.694766

Deprotonated at C3–O⁻ and C6–O⁻

C	0.441723	0.520084	1.215997
C	1.397631	0.347345	0.057986
C	0.649508	0.252864	-1.076702
C	-0.743994	0.383847	-0.734310
O	-0.866838	0.554455	0.621827
O	2.687867	0.299819	0.246573
O	1.009899	0.080308	-2.380750
O	-1.735732	0.366343	-1.446336
C	0.683074	1.789299	2.027759
C	-0.049847	1.819847	3.371899
O	0.524933	2.756172	4.240345
O	0.420864	2.950747	1.244382
O	4.980921	1.808014	-2.969366
O	3.912376	2.703361	-0.662793
O	4.323171	0.918710	2.532813
O	3.277863	-2.301885	1.134948
O	5.687102	2.961811	1.431261
O	3.648237	-0.498819	-2.225700
H	1.975940	-0.141822	-2.435254
H	0.488855	-0.352153	1.877244
H	-1.116937	2.036644	3.190544
H	-0.002413	0.807163	3.807416
H	1.755102	1.826515	2.235929
H	-0.514858	2.934144	0.985814
H	4.166731	0.247706	-2.608909
H	3.545407	-0.278281	-1.271056
H	4.608500	2.275967	-3.731234
H	4.624487	2.261458	-2.159515
H	3.432490	1.909621	-0.336923
H	4.595832	2.888289	0.024709
H	3.658589	0.692304	1.846729
H	3.839270	1.232105	3.337784
H	3.083004	-1.390865	0.822369
H	3.066002	-2.880817	0.388402
H	6.595889	2.695681	1.227991
H	5.271753	2.197607	1.914332
H	1.431196	3.940482	3.446788
O	1.961261	4.678990	3.027604
H	1.607884	4.716579	2.125054
H	-0.396797	4.007938	4.811231
O	-0.769326	4.760903	5.365405
H	-1.421902	5.221802	4.818550
H	0.421261	2.469125	5.906706
O	0.434591	2.488238	6.907128
H	-0.219647	3.169067	7.130829
H	2.637678	4.576873	6.350648
O	2.933413	3.753469	6.804788
H	2.088045	3.298559	7.023538
H	2.076587	2.113529	4.554628
O	2.990845	1.779765	4.776274
H	3.297093	2.434015	5.440609
H	0.829210	5.674122	5.562804
O	1.771180	5.941195	5.461886
H	1.954238	5.717480	4.522175

Deprotonated at C5–O⁻ and C6–O⁻

C	2.251020	-0.322709	0.109142
O	1.306723	-0.699275	-0.780486
C	0.095485	-1.054721	-0.076507
C	0.458903	-0.901894	1.372098
C	1.722383	-0.458118	1.451151
C	-1.079354	-0.145044	-0.472036
C	-1.449654	-0.334361	-1.952173
O	-2.778608	0.057013	-2.230161
O	-0.371632	-1.117728	2.387776
O	2.402738	-0.042438	2.556921
O	3.360044	0.062202	-0.220140
O	-0.883181	1.174309	-0.092053
O	-4.628065	-1.118464	-3.799848
O	-4.150643	-1.562344	-0.443584
O	-3.064908	-1.234565	2.015916
O	-2.770618	2.772185	-2.740671
O	-2.584030	0.930111	-4.831723
O	-5.518697	0.707894	-1.845977
H	3.324317	0.139338	2.301900
H	-0.127384	-2.096322	-0.329422
H	-0.736280	0.240488	-2.563529
H	-1.312671	-1.397851	-2.215162
H	-1.921835	-0.579941	0.087561
H	-1.320381	-1.248739	2.123012
H	-4.546467	0.747988	-1.735540
H	-5.605135	0.122267	-2.626566
H	-2.750821	1.856427	-2.346930
H	-2.753571	2.567401	-3.696771
H	-2.637655	0.505528	-3.934987

H	-3.342188	0.566710	-5.314189
H	-3.840574	-0.899222	-3.230987
H	-4.817724	-2.058137	-3.663305
H	-3.620455	-0.996002	-1.071393
H	-5.040906	-1.174681	-0.486321
H	-3.523558	-1.459945	1.160702
H	-3.529663	-1.702908	2.726565
H	0.040584	1.836662	1.134254
O	0.504303	2.415174	1.817858
H	1.179103	1.861441	2.243255
H	0.599325	4.193809	-0.635332
O	1.026032	4.628973	0.140233
H	0.953523	3.945288	0.838050
H	-0.361329	2.277264	-1.184108
O	-0.136013	3.044643	-1.797223
H	-0.994509	3.212007	-2.236606
H	-4.509102	3.411592	-2.480372
O	-5.477707	3.490315	-2.343649
H	-5.740555	2.560504	-2.183699
H	-2.244131	1.528937	0.793324
O	-3.043358	1.626033	1.399757
H	-3.181828	0.731905	1.764533
H	-0.869673	2.986237	2.932726
O	-1.755029	3.160292	3.316191
H	-2.343135	2.650477	2.711664

4 Sample input for calculating core-level ionization energies using the maximum overlap method within the Q-Chem 6.0 software

```
$molecule
0 1
 C     1.825823     0.674620     0.709209
 C     0.912154    -0.096275    -0.194739
 C    -0.136831    -0.526982     0.504213
 C     0.016518    -0.081475     1.875330
 O     1.168415     0.623924     1.985358
 O     1.134066    -0.303772    -1.493924
 O    -1.191973    -1.256956     0.089733
 O    -0.721961    -0.279053     2.806705
 C     2.048409     2.132593     0.306830
 C     3.010325     2.815911     1.258550
 O     3.344138     4.080424     0.714940
 O     0.825702     2.823450     0.179275
 H    -1.773955    -1.411373     0.845365
 H     2.789155     0.171351     0.809316
 H     2.541475     2.923753     2.240192
 H     3.902363     2.194827     1.372274
 H     3.894457     4.554854     1.343548
 H     2.499691     2.140598    -0.686906
 H     0.445142     2.959890     1.053561
 H     1.988809     0.038980    -1.777962
$end

$rem
METHOD CAM-B3LYP
BASIS General
MAX_SCF_CYCLES 129
solvent_method  pcm
PCM_PRINT      1
MEM_TOTAL  16000
$end

$basis
H 0
aug-cc-pVTZ
****
C 0
aug-cc-pCVTZ
****
O 0
aug-cc-pCVTZ
****
$end

$pcm
THEORY            IEFPCM
RADII             UFF
vdwScale          1.1
NonEquilibrium
$end

$solvent
Dielectric        78.39
OpticalDielectric 1.78
$end

@@@
$molecule
1 2
READ
$end
$rem
METHOD CAM-B3LYP
BASIS General
MAX_SCF_CYCLES 129
unrestricted TRUE
mom_start   1
MOM_METHOD IMOM
scf_guess read
solvent_method  pcm
PCM_PRINT      1
MEM_TOTAL  16000
$end

$basis
H 0
aug-cc-pVTZ
****
C 0
aug-cc-pCVTZ
****
O 0
aug-cc-pCVTZ
****
$end

$pcm
THEORY            IEFPCM
RADII             UFF
vdwScale          1.1
StateSpecific     Marcus
$end

$solvent
```

```
Dielectric        78.39
OpticalDielectric 1.78
$end

$occupied
 1:46
 1:6 8:46
$end
```

5 Cartesian coordinates of structures sampled from QM/MM dynamics

5.1 Fully protonated molecule of vitamin C

C	0.73270565764705897	0.14730178470588234	-0.23382298323529410
C	1.0468530576470589	0.98908504470588232	0.95052217076470602
C	0.11860095764705880	1.9784511847058823	0.97168117076470595
C	-0.76535520235294119	1.8918391847058824	-0.26580528823529409
O	-0.40052861235294124	0.73515877470588231	-0.86518568923529404
O	2.0949684576470586	0.68465971470588238	1.6853662707647059
O	7.0955757647058748E-002	3.0341725847058827	1.8400036707647058
O	-1.6416791423529413	2.6716788847058819	-0.64230069923529409
C	0.47019885764705882	-1.3606667152941176	0.18533310076470591
C	-0.15429394235294125	-2.0871713152941176	-0.97744688923529410
O	-0.31836965235294123	-3.4563091152941174	-0.55725131923529414
O	-0.36598474235294121	-1.4632259152941176	1.2975286707647060
H	-0.73559925235294121	3.5429686847058823	1.6522233707647058
H	1.6140822576470588	0.17004256470588230	-0.89665054923529408
H	-1.1266506423529412	-1.6117165152941177	-1.1727356292352942
H	0.45286405764705884	-2.0683396152941178	-1.9277706292352941
H	-1.0927678723529413	-3.7979292152941175	-1.0436887492352942
H	1.4429387576470589	-1.8156213152941179	0.33872569076470593
H	8.0803576470587224E-003	-0.99088341529411772	2.0688538707647059
H	2.1974245576470590	1.2921216647058822	2.4071749707647059

C	0.88214317505882356	0.17377727352941175	-0.24647416970588237
C	1.1184879750588235	1.0434523235294118	0.96098437029411776
C	0.10618187505882348	1.9701581835294117	1.0605659702941175
C	-0.72027704494117650	1.8596687835294117	-0.21141610970588237
O	-0.17044442494117651	0.87366748352941181	-0.94732792970588231
O	2.1186865750588235	0.75699184352941173	1.7918284702941176
O	-8.2585524941176436E-002	2.8365564835294119	2.0792905702941176
O	-1.5769318049411765	2.6649343835294115	-0.53464464970588232
C	0.43990497505882353	-1.3274668164705883	5.0243985294117645E-002
C	-0.25747702494117641	-2.0753571164705883	-1.0792375297058825
O	-0.29518242494117641	-3.4905279164705885	-0.64455669970588236
O	-0.53210322494117646	-1.3269045164705882	1.0465377702941177
H	-0.74509815494117648	3.5140715835294118	1.8918322702941175
H	1.7739962750588236	0.18163897352941175	-0.80709572970588228
H	-1.3226350909411764	-1.7024893164705881	-1.1800871297058824
H	0.26923987505882363	-2.1870128164705882	-2.0254354297058823
H	-1.0059060049411765	-3.7651588164705885	-1.2050080297058825
H	1.3501432750588234	-1.9368672164705882	0.38222292029411764
H	-0.32403540494117644	-0.89688281647058821	1.8883619702941175
H	2.1172837750588234	1.2529332235294117	2.6405587702941173

C	0.76646123147058831	0.10113704547058833	-0.20554990552941174
C	1.0400114314705882	0.90740785547058833	0.98548331447058823
C	0.19764123147058821	1.9667360954705884	1.0660307444705883
C	-0.67508914852941182	1.8822859954705882	-1.1810687529411758E-002
O	-0.36108366852941187	0.81206989547058828	-0.76558762552941173
O	2.0207937314705884	0.53954480247058834	1.8542652444705883
O	0.19524303147058819	2.9659288954705882	1.9537704444705883
O	-1.6138788985294119	2.6265205954705881	-0.36017079552941178
C	0.45512123147058814	-1.3789583045294118	6.0997790470588245E-002
C	-0.21774226852941192	-2.0479463045294120	-1.1352882555294117
O	-0.33042276852941188	-3.4559775045294119	-0.89313790552941175
O	-0.44002125852941187	-1.5269543045294118	1.1541230444705883
H	-0.45823720852941185	3.6343889954705881	1.6761839444705884
H	1.5564085314705880	0.17138795547058833	-1.0133391555294116
H	-1.1772774585294119	-1.6188882045294117	-1.3261760555294118
H	0.29290603147058825	-1.8568991045294116	-2.1440689555294115
H	-1.2508337735294119	-3.7217844045294122	-0.89572518552941183
H	1.3643262314705880	-1.9482711045294119	0.26207672447058822
H	8.0600231470588257E-002	-1.3755826045294115	2.0161293444705883
H	1.8690693314705882	1.0363397354705883	2.6648824444705883

C	0.78058292982352961	7.9318864117647048E-002	-0.12746648405882346
C	1.1014151298235295	0.98591657411764699	1.0242491959411764
C	0.18424712982352931	1.9863336641176470	0.97304057594117654
C	-0.81909626017647064	1.7089933641176469	-5.4085365058823484E-002
O	-0.38759958017647056	0.63062646411764711	-0.76649163405882337
O	2.1433962298235296	0.68060480411764712	1.7600544959411766
O	6.9831298235294437E-003	2.9713161641176473	1.8952759959411765
O	-1.8144461801764706	2.3684504641176471	-0.31381451405882349
C	0.45971342982352947	-1.4304546358823531	0.16192980594117654
C	-0.18721177017647062	-2.0098970358823527	-1.1663659040588235
O	-0.24261807017647063	-3.4615343358823529	-1.0102506040588235
O	-0.59206645017647064	-1.4074428358823530	1.1886532959411764
H	-0.32655213017647056	3.7902654641176472	1.4762790959411765
H	1.6183806298235295	0.22159942411764705	-0.75258908405882341
H	-1.2349894071764707	-1.5943265358823531	-1.2561124040588236
H	0.45304192982352931	-1.7287704358823532	-2.1161791040588236
H	-1.1431806901764705	-3.7909994358823531	-0.91612736405882345
H	1.3589571298235295	-1.9805829358823528	0.43573231594117651
H	-0.38125618017647056	-0.79781403588235311	1.9114545959411764
H	2.0885329298235296	1.1333170041176470	2.5837204959411766

C	0.91452563929411745	0.13421282411764701	-0.23194648911764706
C	1.0943051392941174	0.97849211411764703	1.0318326408823530
C	0.11274803929411759	1.8937703241176469	1.1154383408823529
C	-0.70181134070588258	1.7580566241176472	-0.14074598411764708
O	-0.19742125070588257	0.79968446411764704	-0.90028348911764700
O	2.1070901392941175	0.57885844411764709	1.7941363408823530
O	-0.14216986070588256	2.8474078241176470	2.0348067408823529
O	-1.6425938007058825	2.4118544241176472	-0.46665427911764712
C	0.47031513929411739	-1.2793233758823530	9.4322790882352930E-002
C	-0.14650056070588247	-1.9444826758823530	-1.0829481091176472
O	-0.49307275070588252	-3.3140110758823531	-0.73391251911764710
O	-0.36507447070588250	-1.3711957758823528	1.1859975408823529
H	-0.93721782070588255	3.3063837241176470	1.7864937408823529
H	1.7730402392941174	0.10659406411764705	-0.90401308911764700
H	-1.0551030307058826	-1.4148616758823529	-1.1926953591176470
H	0.40444733929411747	-1.8678071758823531	-2.0605802591176472
H	-1.1955067887058826	-3.6236330758823532	-1.3292485591176471
H	1.4695645392941175	-1.6572302758823532	0.32446519088235293
H	0.21226443929411754	-1.2640538758823530	1.9928623408823529
H	2.1939250392941174	1.2697278541176469	2.4565160408823528

C	0.83111574176470571	0.18601829941176468	-0.30568109658823522
C	1.0663871417647055	0.93649095941176463	0.96685410341176481
C	0.10207044176470581	1.8919457294117645	1.0383377034117649
C	-0.71461843823529425	1.8168006294117647	-0.14025113558823527
O	-0.20883673823529425	0.87363296941176460	-0.96785841658823524
O	2.0498012417647056	0.59796165941176471	1.7971773034117648
O	-0.11728125823529423	2.7888887294117648	2.0622768034117644
O	-1.6440892682352943	2.5839164294117647	-0.33892077658823527
C	0.53498944176470564	-1.2628241705882353	3.7841264411764747E-002
C	-0.22897202823529428	-1.9787948705882350	-1.0511732365882354
O	-0.43805620823529434	-3.3396718705882353	-0.63816815658823522
O	-0.39654688823529427	-1.3704191705882351	1.1807239034117647
H	-0.78912718823529426	3.4200359294117648	1.7731798034117647
H	1.7705111417647057	0.28429005941176466	-0.94121387658823519
H	-1.2539107582352942	-1.5443413705882354	-1.1489340965882353
H	0.22711404176470573	-1.9849163705882353	-2.0251347965882354
H	-0.79055041823529426	-3.8990135705882354	-1.2990552965882352
H	1.4963611417647056	-1.6715388705882352	0.22747994341176475
H	0.16281734176470564	-1.2622420705882353	1.9215442034117647
H	2.0626891417647055	1.0234258694117648	2.6265963034117648

C	0.79991060120000013	6.0371284882352994E-002	-0.17145288090588237
C	1.0882917012000002	0.85814900488235302	1.0925959259941176
C	0.21858070120000006	1.9082146648823530	1.0803533259941176
C	-0.56083588880000002	1.9251878648823531	-0.13655068900588235
O	-0.18510579880000000	0.86075725488235300	-0.93555264400588245
O	1.9955342011999999	0.42257278188235303	1.9851942259941176

O	-4.9037398799999909E-002	2.8977664648823529	1.9662329259941176
O	-1.3323371762000000	2.7994317648823528	-0.48497940400588235
C	0.32236330120000001	-1.3641225351176471	0.10076850599411766
C	-0.28449399879999993	-1.9783666351176472	-1.1381663340058823
O	-0.26900279879999989	-3.4384362351176470	-1.0820951240058823
O	-0.67154182880000002	-1.5067136351176471	1.2004604259941176
H	-0.57948371879999994	3.6421065648823530	1.5012191259941177
H	1.7113919011999998	-3.9599105117647027E-002	-0.74218811400588236
H	-1.3095509117999999	-1.6406834351176469	-1.3039491740058824
H	0.25878240119999996	-1.6811782351176472	-2.0494600740058821
H	-1.1534652888000001	-3.7254578351176466	-0.88243002400588244
H	1.1705107012000000	-1.8488358351176473	0.47469536599411766
H	-0.43817544879999992	-0.96881873511764693	1.9274081259941176
H	1.9579479011999998	0.92784481488235304	2.8129817259941174
C	0.82582406941176489	9.6719284705882286E-002	-0.15527748182352941
C	0.94008026941176470	0.88250721470588234	1.1026156341764706
C	0.13080026941176492	1.9954041347058822	1.1117049341764706
C	-0.68013469058823506	1.8538837347058821	-0.12500348082352941
O	-0.17582603058823509	0.82720471470588230	-0.85795134582352939
O	1.8413769694117648	0.43560806470588231	2.0672378341764706
O	-6.2017430588235056E-002	2.8851681347058822	2.1536167341764703
O	-1.5832801305882351	2.5471943347058823	-0.50720671582352939
C	0.30191106941176482	-1.4084043652941176	1.2243044176470583E-002
C	-0.19584553058823517	-1.9534481652941178	-1.2555767658235295
O	-0.24477823058823511	-3.4072483652941177	-1.2120702658235294
O	-0.61143383058823508	-1.4956559652941177	1.0758953341764705
H	-0.54109773058823507	3.6807012347058823	1.7811376341764706
H	1.7159378694117648	-2.5420595294117720E-002	-0.69574172582352944
H	-1.0999402505882352	-1.5979969652941177	-1.6056842658235293
H	0.52599266941176492	-1.6675954652941178	-2.0691797658235296
H	-1.0875693305882350	-3.6486209652941177	-0.82075933582352945
H	1.2366529694117647	-2.0261025652941180	0.25668439417647060
H	-0.20723663058823516	-1.1038734652941176	1.8602504341764705
H	1.6194244694117648	0.90241150470588227	2.9122981341764702
C	0.75012441488235337	0.10293562841176473	-0.25489613694117647
C	0.97193881488235334	0.80935074841176469	1.0820075030588234
C	0.27601401488235311	1.9067277684117649	1.1580764030588235
C	-0.59000480511764686	1.9732210684117648	-2.9468458941176459E-002
O	-0.27997958511764676	0.83460231841176469	-0.88508648694117653
O	1.8174326148823532	0.33061656541176476	1.9894276030588236
O	0.19757081488235317	2.7001303684117648	2.2397426030588239
O	-1.4367393081176467	2.8025711684117649	-0.27126370694117646
C	0.28603681488235311	-1.3608223315882353	-0.11365070694117646
C	-0.26844248511764679	-1.9842458315882352	-1.3731616969411766
O	-0.42173095511764680	-3.3879882315882353	-1.1762078969411764
O	-0.80386743511764680	-1.5497946315882354	0.76581727305882352
H	-0.15519838511764683	3.6059923658823517647	2.1773156030588239
H	1.6593045148823533	0.13575681841176473	-0.80715339694117638
H	-1.2342521951176468	-1.5157357315882352	-1.6282884969411766
H	0.41539851488235313	-1.8368276315882350	-2.2299250969411761
H	-1.1836053651176468	-3.5664904315882353	-0.64328490694117635
H	1.1944842148823533	-1.9077780315882351	0.28172050305882357
H	-0.61533106511764679	-1.1506638315882354	1.6957223030588235
H	2.0332161148823533	0.88606396841176471	2.7464867030588236
C	0.74900988899999987	0.16046090270588254	-0.20334516876470593
C	1.0587974889999998	0.85694214270588254	1.1085393912352941
C	0.20657858899999981	1.8851837127058826	1.2341527112352941
C	-0.73689454100000018	1.9212774127058825	0.10212547023529403
O	-0.35807908100000019	0.94389285270588252	-0.75400281876470598
O	2.0402873890000000	0.44694182670588256	1.9227188112352940
O	9.6937688999999772E-002	2.8009850127058824	2.2140392112352938
O	-1.6569728510000004	2.6617626127058824	-0.20027379876470597
C	0.37053098899999970	-1.3403824872941175	2.2863671235294036E-002
C	-0.20220671100000032	-1.9300685872941172	-1.2876186887647059
O	-9.6882811000000180E-002	-3.4384398872941175	-1.2681105887647059
O	-0.64683083100000027	-1.5616899872941175	1.0430363412352941
H	-0.55701002100000019	3.4614811127058824	1.8821893112352941
H	1.5775694889999996	0.17668222270588252	-0.94906818876470589
H	-1.2249903140000002	-1.6155351872941175	-1.4164089887647060
H	0.39228148899999971	-1.6488514872941176	-2.1402242887647063
H	-1.0121258510000002	-3.7806421872941178	-1.3106123887647059
H	1.3337694889999998	-1.8587235872941172	0.35227958123529401
H	-0.25759648100000021	-1.1794666872941175	1.8584682112352942

H	1.9786717889999998	0.81905167270588253	2.8312142112352938
C	0.73795154582352995	0.12976882700000003	-0.21453671117647061
C	0.97360174582352976	0.78590096700000012	1.1282452788235293
C	8.9365045823529776E-002	1.8288031070000001	1.2799731988235294
C	-0.70337478417647026	1.8753616069999999	5.7649128823529405E-002
O	-0.34407149417647021	0.87168662699999999	-0.82257660117647058
O	1.9893258458235297	0.45846138700000005	1.9646010988235294
O	-7.4037054176470329E-002	2.6536264070000000	2.3204599988235293
O	-1.4778631041764703	2.7872258070000000	-0.19587265117647057
C	0.36907584582352970	-1.3450678930000000	6.0049988235294227E-003
C	-0.29599261417647027	-1.8941863930000000	-1.2142616011764706
O	-0.38325478417647030	-3.3551576929999998	-1.1070233011764705
O	-0.54824937417647024	-1.4695025930000001	1.1036543688235294
H	-0.67293343417647022	3.3987886070000002	2.0270555988235293
H	1.6102577458235297	0.26555520800000004	-0.89799530117647053
H	-1.3253615231764702	-1.4560597930000001	-1.3007631011764704
H	0.14834704582352964	-1.7028980929999999	-2.2022832011764706
H	-9.2786654176470362E-002	-3.8323060930000001	-1.9323312011764704
H	1.2306529458235296	-2.0228073929999999	0.19336314082352940
H	-0.11217535417647029	-1.1837561929999998	1.9046971988235295
H	1.8286940458235297	0.81854501700000004	2.8516322988235294
C	0.73100992411764731	0.17200749176470592	-0.19947686117647062
C	1.0293026241176471	0.89141027176470589	1.0693594388235295
C	0.17565972411764719	1.9658105417647058	1.1190031688235293
C	-0.68755571588235276	1.9352047417647060	-0.10604870117647064
O	-0.33882687588235272	0.90083472176470591	-0.84275797117647078
O	1.8233379241176471	0.42424536176470595	2.0640735288235295
O	-8.7284758823527042E-003	2.8194648417647059	2.1770185288235293
O	-1.5919864158823529	2.6837681417647059	-0.37717284117647060
C	0.27272142411764722	-1.3041142582352940	5.2033088823529361E-002
C	-0.28351827588235290	-1.9902739582352940	-1.2089601711764708
O	-0.30522097588235275	-3.4309423582352943	-0.96136247117647078
O	-0.71583439588235276	-1.3517778582352942	1.1081706188235294
H	-0.82741817588235278	3.3771856417647061	2.1634397288235294
H	1.6575461241176470	0.23394175176470594	-0.78180707117647064
H	-1.3088449858823528	-1.6664131582352941	-1.4703118711764707
H	0.41884542411764714	-1.7078230582352942	-2.0420191711764706
H	-4.0488875882352726E-002	-3.9525288582352944	-1.7631809711764708
H	1.1299832241176471	-1.8714209582352939	0.31517894782352934
H	-0.32508997588235289	-0.98841345823529414	1.9065006288235293
H	1.7895357241176473	0.89254708176470587	2.9067580288235297
C	0.80833418941176483	6.8316596341176483E-002	-0.12684088111764710
C	0.99943018941176476	0.92077310634117648	1.0736349288235528
C	0.10122398941176458	1.9380450263411764	1.0236843688235529
C	-0.58135336058823539	1.9101049263411765	-0.24004216111764706
O	-0.19783631058823548	0.79996197634117649	-0.93609436111764710
O	1.8683033894117647	0.49714080854117648	2.0047880388235529
O	-0.14065051058823541	2.9471700263411762	2.0051976388235527
O	-1.4370794705882355	2.6808241263411761	-0.63923647111764703
C	0.23877258941176471	-1.3497592736588235	0.19322358788235292
C	-0.24137461058823528	-2.0674941736588233	-1.0462081611176470
O	-0.19052491058823540	-3.4844459736588238	-0.80532796111764715
O	-0.68269814058823541	-1.2833909736588236	1.2273429388235529
H	-0.39732776058823538	3.8166805263411767	1.6062327388823530
H	1.6854664894117648	-4.1997103658823587E-002	-0.79147556111764694
H	-1.2793916705882353	-1.8292051736588235	-1.3031435611176470
H	0.37203368941176462	-1.6055471736588236	-1.9381033611176470
H	-0.92532778058823539	-3.9171772736588233	-1.3076317611176471
H	1.1287879894117645	-1.8564447836588235	0.56696447888235291
H	-0.31680991058823538	-1.3403643736588235	2.1391856388823527
H	1.7126798894117647	0.94751456634117648	2.8481479388823527
C	0.66498693258823516	4.2943816882352770E-002	-0.10620475852941175
C	1.0055582325882353	0.97883827688235281	1.0150084914705881
C	0.30796433258823508	2.1216962768823526	0.86062267147058824
C	-0.50808488741176483	1.9813454768823529	-0.32177978852941180
O	-0.25204836741176484	0.81145969688235287	-0.94074338852941175
O	1.8013504325882350	0.60618771988235276	2.0233869114705882
O	0.30589063258823512	3.0270645768823528	1.8627367114705882
O	-1.2805868744117648	2.8150089768823530	-0.78618738852941183
C	1.4804132588235097E-002	-1.3412443231176472	0.25727976647058826
C	-0.38308615741176488	-2.1456927231176470	-0.95745448852941173
O	-0.30565696741176485	-3.5260285231176471	-0.55521167852941178

O	-1.1242100474117649	-1.2937473231176473	1.0836966214705881
H	-1.0978467411764781E-002	3.9002617768823526	1.5472071114705881
H	1.5677973325882351	-0.14794613311764726	-0.70954932852941177
H	-1.3681538244117648	-1.8187070231176472	-1.1802046885294120
H	0.27812303258823512	-1.9403249231176471	-1.8295153885294120
H	-0.71366946741176485	-4.0711156231176471	-1.2630873885294118
H	0.86257133258823493	-1.8591210231176474	0.68407162147058820
H	-0.86430259741176485	-1.0471377231176473	1.9979909114705880
H	1.5962026325882352	1.1363757268823527	2.8534336114705883

C	0.76532996676470577	2.2475701176470686E-002	-0.16546871423529408
C	0.85860116676470599	0.89990598117647069	1.1036894457647060
C	0.18758826676470597	1.9984051511764707	0.97941002576470593
C	-0.54073882323529410	1.9226741511764707	-0.26859655423529405
O	-9.2820933235294056E-002	0.82788271117647072	-0.98139811423529411
O	1.6664401667647060	0.53183598117647068	2.1334881857647057
O	0.22835666676470590	3.0814278511764703	1.8194368857647059
O	-1.4555439632352940	2.6597795511764710	-0.69381944423529407
C	0.22122406676470585	-1.3360717488235292	0.13652838776470594
C	-0.27983180323529411	-1.9612375488235292	-1.1655330142352940
O	-0.16676933323529419	-3.4069852488235295	-0.89541581423529415
O	-0.77534478323529410	-1.3529545488235293	1.1627595857647059
H	-0.70578173323529414	3.3438538511764708	1.9750735857647059
H	1.8149408667647060	-3.6856088823529309E-002	-0.54886550423529412
H	-1.2907651582352941	-1.6436658488235294	-1.4879507142352941
H	0.51409956676470592	-1.6506143488235294	-1.9509111142352942
H	-0.94898420323529409	-3.8998555488235294	-1.1524271142352940
H	1.0490366667647060	-1.8926676488235292	0.55524824576470588
H	-0.33906884323529407	-1.4375496488235291	2.0089453857647062
H	1.5139949667647057	1.1752100811764707	2.8644322857647060

C	0.71719746217647051	0.12021698882352927	-0.22359958523529408
C	0.98295126217647022	0.94351827882352923	1.0488989847647059
C	0.26334476217647040	2.0662122288235292	1.0472719347647059
C	-0.55327836782352957	2.0653701288235293	-0.20832245523529405
O	-0.27373333782352960	0.96045524882352928	-0.88391313523529413
O	1.7135230621764705	0.21524264882352928	1.9253901647647060
O	0.20980656217647042	3.0594189288235292	1.9545470647647061
O	-1.2925498028235296	2.9299050288235291	-0.67946325523529405
C	0.30469186217647048	-1.3688872711764706	0.11533621376470593
C	-0.30404347782352958	-2.1484108711764707	-1.0423399352352940
O	-0.10219983782352959	-3.6025888711764709	-0.97655473523529401
O	-0.69936854782352964	-1.3291802711764706	1.1630036647647060
H	-0.37624631782352957	3.7768613288235291	1.6932529647647061
H	1.5742465621764703	0.12397078882352930	-0.81059868523529410
H	-1.3666232298235297	-1.9300363711764708	-0.99745213523529419
H	0.14190666217647041	-1.7976768711764710	-1.9512002352352942
H	-0.93962527782352967	-4.0843910711764702	-1.1742568352352940
H	1.2300376621764704	-1.7929216711764711	0.53998171476470591
H	-0.18512693782352962	-1.1026488711764708	1.9674470647647060
H	1.8958040621764702	0.63753561882352927	2.7635034647647059

C	0.68515400664705894	7.7369054705882401E-002	-0.20296623792352944
C	1.1074129066470590	0.79691411470588236	1.0380604120764705
C	0.35093510664705874	1.9149647747058824	1.0702571820764706
C	-0.56224295335294117	1.9847872747058823	-0.11055707792352945
O	-0.35758153335294118	0.87231834470588243	-0.81933915792352940
O	2.1209871066470587	0.31671424470588239	1.7867890220764704
O	0.37127310664705893	2.8725571747058827	2.0777612220764707
O	-1.3001413763529412	2.8671125747058825	-0.45349671792352941
C	0.13470970664705884	-1.3266160252941175	0.13755042677647056
C	-0.36132268335294115	-2.0472755252941175	-1.1300867779235295
O	-0.17360989335294108	-3.4683856252941174	-1.1317028779235294
O	-0.94539995335294114	-1.3602219252941175	1.0841024720764705
H	-0.13941559335294107	3.6706856747058825	1.9519648220764705
H	1.4408075066470587	-5.3626055294117569E-002	-0.96056567792352932
H	-1.4096895533529412	-1.7358386252941176	-1.2798418779235294
H	0.15775510664705883	-1.5953631252941176	-1.9905804779235297
H	-1.1196310133529412	-3.7860963252941171	-1.0673486779235295
H	1.0009058066470586	-1.9113953252941176	0.51911757207647058
H	-0.66401559335294114	-1.1763694252941175	1.9650869220764704
H	2.0812974066470584	0.43411672270588242	2.7438626220764704

C	0.77537870647058860	0.11946178176470579	-0.32937027311764705
C	1.0667649064705884	0.87353081176470582	0.92675044688235297
C	0.43617450647058864	2.0252952517647058	0.98401716688235286
C	-0.59108330352941141	2.0161517517647058	-8.5798331117647050E-002
O	-0.27319956352941144	0.99916186176470578	-0.93496423311764709
O	2.0142827064705884	0.45310126176470578	1.7231811668823531
O	0.42337880647058856	2.8074365517647055	2.0858961668823532
O	-1.4744565735294115	2.8245814517647059	-0.20619351311764705
C	0.29978310647058848	-1.3528570482352942	-1.7028892117647049E-002
C	-0.49607536352941140	-2.0142418482352942	-1.1192377331176468
O	-0.24947775352941148	-3.4249417482352946	-0.91009325311764711
O	-0.47379439352941144	-1.4126387482352945	1.1464007668823530
H	-0.32259207352941144	3.4292935517647058	2.0008728668823532
H	1.6253776064705887	0.10689286176470580	-1.0480285331176469
H	-1.5106411735294114	-1.7941542482352943	-1.0248098531176471
H	-0.15415659352941136	-1.7147291482352944	-2.1425923331176469
H	-1.0956635535294115	-3.9413443482352939	-1.0490016331176470
H	1.2475895064705886	-1.8104111482352945	0.16712426688235296
H	9.0144506470588581E-002	-1.1560788482352944	1.9124527668823530
H	1.9812247064705883	1.0626997217647058	2.4726468668823531

C	0.75713837058823508	6.6007467647058837E-002	-0.19856142705882357
C	1.1398591705882350	0.96402800764705887	0.96781378294117659
C	0.39658737058823479	2.0403145076470590	0.96954118294117642
C	-0.62390161941176514	1.8699325076470588	-9.9699317058823581E-002
O	-0.42024325941176510	0.65998698764705876	-0.71639570705882361
O	2.1344211705882348	0.66529346764705877	1.8158025829411764
O	0.46424167058823484	3.0824972076470587	1.8417466829411766
O	-1.5216973094117652	2.6059751076470592	-0.41532702705882357
C	0.35538737058823489	-1.3791333923529412	0.12419028294117640
C	-0.38759395941176511	-2.0017817923529408	-1.0348432470588236
O	-0.39529685941176507	-3.3931812923529412	-0.85024746705882359
O	-0.59100677941176516	-1.4076125923529412	1.2613663829411765
H	-0.47836383941176508	3.3366283076470591	1.9576593829411766
H	1.4964455705882349	0.16108907764705882	-0.94958573705882365
H	-1.4551325294117652	-1.6510156923529411	-1.1665043170588236
H	8.6763370588234912E-002	-1.8631357923529410	-1.9830625170588234
H	-0.95760790941176510	-3.7558920923529415	-1.5238935170588235
H	1.2309291705882348	-2.0087846923529415	0.41905727294117640
H	-0.11734972941176514	-1.2658062923529412	2.0511066829411764
H	2.2129791705882349	1.1544451176470587	2.6129546829411763

C	0.76753652999999988	7.4243869999999823E-002	-0.18289495258823524
C	1.1539711299999997	0.98417824999999981	0.91217329741176478
C	0.34528342999999984	2.0936249699999996	0.98144605741176483
C	-0.68088759000000021	1.8697099699999997	-8.4696828588235240E-002
O	-0.44949352000000020	0.74377745999999978	-0.72848236258823518
O	2.1909831300000002	0.63860427999999980	1.6541775574117648
O	0.12987422999999976	3.0259111699999997	1.9231285574117647
O	-1.6690340900000002	2.5838803700000001	-0.28891407258823526
C	0.42595892999999974	-1.3828577300000002	0.19515888741176474
C	-0.36844005000000013	-2.0267571300000000	-1.0218641425882353
O	-0.17299577000000022	-3.4442971299999998	-0.96609102258823520
O	-0.41092912000000015	-1.3583068300000001	1.3594548574117646
H	-0.69043500000000013	3.5035280699999998	1.7011368574117647
H	1.5570319299999997	-5.9521200000000163E-002	-0.98627086258823515
H	-1.4118034000000002	-1.7129174300000003	-0.84613284258823518
H	9.5015729999999854E-002	-1.7408081300000002	-2.0040315425882351
H	-0.81163650000000009	-3.7919928299999999	-1.6172974425882354
H	1.4210771299999998	-1.8944870300000003	0.29238039741176475
H	0.16298362999999982	-1.3713992300000002	2.1154831574117647
H	2.3826494299999998	1.2695792999999997	2.3843470574117651

C	0.74743830041176507	0.12631414764705878	-0.25873211376470595
C	1.1314382004117651	0.90296013764705885	0.97479083623529406
C	0.34374310041176503	1.9934761176470588	1.0291652362352941
C	-0.68374947958823495	1.9176855176470586	-2.5600682764705941E-002
O	-0.45862467958823494	0.83088042764705872	-0.76609129376470597
O	2.1435083004117650	0.59244490764705882	1.8031019362352942
O	0.39439950041176508	2.9642894176470591	1.9122572362352941
O	-1.5294234665882349	2.7488459176470590	-0.31293526376470593
C	0.33347140041176515	-1.2887952823529412	3.9447692352940593E-003
C	-0.38547449958823488	-1.9847082823529412	-1.1897699637647059
O	-0.55026438958823498	-3.3609211823529410	-0.67650521376470596
O	-0.37919769958823490	-1.4942348823529412	1.2483094362352942
H	-0.44882193958823491	3.3239531176470591	2.1642445362352940
H	1.4800766004117651	0.14418576764705882	-1.0776653637647058
H	-1.2878905495882349	-1.6235612823529413	-1.6532089637647058
H	0.28551110041176497	-1.8631432823529412	-1.9816097637647059

H	-1.1361397995882350	-3.9296712823529409	-1.1936953637647059
H	1.1970146004117650	-1.9492428823529411	2.5261266235294056E-002
H	0.19106670041176499	-1.6496163823529411	2.0262699362352943
H	1.9247670004117650	0.90984631764705881	2.6912947362352941

C	0.67704255823529413	0.13025687894117638	-0.29929369000000006
C	1.2016741582352943	0.95026304894117641	0.82678797999999998
C	0.36560425823529430	1.9706027489411764	1.0884912000000000
C	-0.79200049176470577	1.8622710489411765	0.18007803999999999
O	-0.64450808176470575	0.79340726894117641	-0.58210259000000009
O	2.3811733582352943	0.61884071894117643	1.2657219999999998
O	0.55473255823529399	2.9530673489411763	1.9727727000000002
O	-1.7175362517647057	2.6521169489411767	7.9221509999999967E-002
C	0.41930835823529411	-1.3844453510588237	9.3281799999999776E-003
C	-0.37815684176470588	-2.0729350510588240	-1.1279550000000000
O	-0.38873804176470572	-3.4808006510588241	-0.90751343000000007
O	-0.35034094176470587	-1.5069111510588236	1.2202807999999998
H	-0.12629594176470582	3.6606189489411767	1.9520876999999999
H	1.3143088582352944	0.31501659694117634	-1.1736513000000000
H	-1.4166608417647057	-1.6462466510588236	-1.1189152999999998
H	7.0821058235294165E-002	-1.8576927510588235	-2.1230590999999999
H	-1.1704277317647058	-3.9574299510588231	-1.2622797000000001
H	1.4163796582352943	-1.8238975510588238	3.5712819999999978E-002
H	0.32498345823529418	-1.4666600510588237	1.8730921999999999
H	2.6874010582352938	1.3813116489411763	1.8225419000000000

C	0.53470149352941143	0.23052467799999993	-0.33542581823529410
C	1.2223136935294117	0.98459472199999987	0.78467089176470584
C	0.39515229352941161	2.0080392919999999	1.0903308717647058
C	-0.83264817647058831	1.8927013919999998	0.30568734176470586
O	-0.82356927647058831	0.78475086199999988	-0.44790915823529415
O	2.4530308935294114	0.70068523199999988	1.1786339717647059
O	0.71663439352941150	2.9840031919999999	2.0882433717647060
O	-1.7840274664705884	2.6422227919999997	0.33525967176470589
C	0.42442409352941168	-1.3146273079999999	-8.7229818235294146E-002
C	-0.33143930647058828	-2.0549383080000001	-1.1791137282352941
O	-8.0662906470588380E-002	-3.4935180080000001	-1.1887932282352942
O	-0.26622880647058844	-1.5377724079999999	1.0913115717647057
H	-0.10049530647058846	3.4876969919999996	2.2708311717647058
H	1.0176026935294116	0.44503800199999993	-1.3156680282352942
H	-1.3995043064705883	-1.9479310080000003	-1.0129520282352942
H	-0.17349240647058828	-1.8011225080000002	-2.2154720282352942
H	-0.97179159647058833	-4.0103476080000000	-1.3624050282352942
H	1.4696774935294117	-1.6576979080000003	-5.9963849235294152E-002
H	0.29203949352941172	-1.9175443080000001	1.7381505717647057
H	2.5718219935294115	1.1977712320000000	2.0182332717647058

C	0.56976117885294109	0.19658146576470584	-0.43239797529411761
C	1.2136232788529413	1.0181255677647059	0.65550325470588233
C	0.37093417885294122	1.9933120277647058	1.0663170447058823
C	-0.84634336114705888	1.8261553277647058	0.45624594470588237
O	-0.73607993114705883	0.82790683776470586	-0.48360491529411764
O	2.3979195788529410	0.61199556776470576	1.1035754647058824
O	0.67714897885294123	2.7978592277647056	2.1595007747058821
O	-1.9290113111470588	2.4079720277647061	0.54548106470588231
C	0.43826557885294126	-1.2918870722352942	-0.20261068529411763
C	-0.45486972114705893	-1.9597775722352944	-1.1674340252941175
O	-0.14768332114705873	-3.3866572722352943	-1.1285025252941177
O	8.7835878852941196E-002	-1.7188087722352943	1.1008855047058823
H	-4.9871211470589039E-003	3.4765847277647062	2.2582467747058823
H	1.0649396788529413	0.41986272776470579	-1.3844973252941177
H	-1.4840874316470589	-1.6821221722352941	-0.95806472529411768
H	-0.24869962114705890	-1.6277586722352941	-2.1536920252941174
H	-0.96866651114705882	-3.9093439722352938	-1.4349516252941177
H	1.4358553788529411	-1.7087771722352942	-0.38090693529411768
H	0.89481787885294128	-2.0466110722352946	1.3880120747058824
H	2.7001085788529409	1.2901015277647059	1.7240783747058823

C	0.58448027000000025	0.23271879752941171	-0.37624050041176466
C	1.1528481700000004	1.0185623920294118	0.75291348958823523
C	0.28584617000000012	1.8879686620294118	1.2604188395882352
C	-0.93545060999999985	1.7687080620294118	0.36161742658823531
O	-0.74386989999999986	0.73181584202941174	-0.47998005041176467
O	2.3796192700000001	0.65578732202941170	1.2202406995882353
O	0.49434057000000009	2.8226412620294119	2.1965735395882353
O	-1.9706608499999998	2.4316206620294119	0.47387212958823532
C	0.52756187000000021	-1.2741368379705882	-0.20838655041176468
C	-0.29475212999999978	-1.9521752379705881	-1.3434983604117647
O	-8.8073629999999792E-002	-3.2973409379705880	-1.1246633604117646
O	-8.7858029999999809E-002	-1.6695081379705883	1.0528710995882353
H	-0.33320122999999979	3.3803168620294120	2.3353195395882351
H	1.1163698700000002	0.36924790202941171	-1.3613997604117647
H	-1.3419017699999998	-1.6390880379705883	-1.2277910604117646
H	0.11685517000000023	-1.6118150379705882	-2.2710709604117647
H	-0.86215320999999978	-3.8553235379705879	-1.2607961604117646
H	1.5664646700000002	-1.6590902379705883	-0.23791350041176468
H	0.54561677000000008	-1.5514273379705883	1.7666552395882353
H	2.6226040700000000	1.3399606620294118	1.8915636395882354

C	0.70827883294117644	0.20599479935294113	-0.28260996405882349
C	1.1744079329411763	0.91337124135294112	0.89727044594117644
C	0.37230313294117634	1.9952808113529410	1.1701460759411764
C	-0.80191245705882375	1.6947953113529410	0.39611621494117649
O	-0.53642746705882383	0.81980003135294122	-0.61538754405882345
O	2.2396825329411763	0.44597564135294110	1.5578506559411764
O	0.43276843294117606	2.9119581113529414	2.1343206559411763
O	-1.9139815370588238	2.2257331113529411	0.60962057594117647
C	0.46872193294117626	-1.2709975886470590	-0.12305926405882350
C	-0.31059046705882376	-1.9504061886470587	-1.3244954440588235
O	-0.32668756705882362	-3.3917379886470589	-1.1292294440588235
O	-0.29171836705882370	-1.5046690886470588	1.0251517559411765
H	-0.29200596705882376	3.5542017113529414	2.0766148559411763
H	1.3481663329411764	0.28422695035294110	-1.2163187440588235
H	-1.3537451870588237	-1.5488333886470589	-1.3638409440588235
H	0.23728923294117621	-1.7018931886470590	-2.2498379440588234
H	-1.1545493470588237	-3.6828002886470586	-1.5623119440588236
H	1.4186143329411762	-1.7412675886470590	-4.6525374058823510E-002
H	0.34972573294117648	-1.4525396886470590	1.7295401559411765
H	2.4577455329411766	0.92449102135294114	2.3610262559411765

C	0.57857700882352914	0.26637002558823525	-0.41503276008823525
C	1.1052926088235293	0.91389418258823529	0.80076120991176469
C	0.27826550882352930	1.9661883025882352	1.0941702899117647
C	-0.86943591117647079	1.8560247025882353	0.23748500991176477
O	-0.64854915117647083	0.93368684258823531	-0.73691270008823517
O	2.2718240088235291	0.52000228258823533	1.2315028799117647
O	0.39681070882352909	2.7068942025882352	2.2569121999117647
O	-1.9314478111764708	2.4351830025882353	0.37459124141176475
C	0.32049660882352926	-1.2180572974117649	-2.3458900882352141E-003
C	-0.34312799117647086	-1.9718701974117647	-1.1534943000882352
O	6.1580808823529232E-002	-3.3505661974117644	-1.0013589000882352
O	-0.58758219117647070	-1.2836962974117649	1.0817759199117647
H	-3.8119791176470796E-002	3.5774597025882353	2.0623043999117647
H	1.3438426088235293	0.23963303558823526	-1.1981380000882351
H	-1.4205768411764708	-1.9991060974117647	-1.1105411000882353
H	-1.6679117647089292E-004	-1.5881280974117646	-2.1195325000882352
H	-0.51768339117647089	-4.0039120974117646	-1.4021470000882352
H	1.3054591088235292	-1.7550598974117646	0.23835329991176477
H	-0.15300269117647081	-1.1249640974117647	1.9295027999117649
H	2.4536447088235289	1.0173594525882352	2.0630183999117646

C	0.73815857235294091	0.27929134070588235	-0.42544126358823525
C	1.2258153723529412	1.0448548457058824	0.75101635641176478
C	0.30578037235294109	2.0302355057058823	1.0591097664117648
C	-0.86327503764705893	1.7383537057058824	0.22830569541176476
O	-0.55281112764705886	0.84657356570588238	-0.72614395358823525
O	2.3714414723529411	0.74766755570588228	1.4206641464117649
O	0.29001537235294106	2.9671487057058825	2.0149189464117647
O	-1.9753450476470589	2.1918711057058822	0.22897808841176476
C	0.42427067235294103	-1.2591540942941175	-0.12332058358823522
C	-0.29253727764705881	-2.1142748942941174	-1.2241211535882353
O	-0.17481722764705898	-3.5302575942941177	-0.95246315358823508
O	-0.31189472764705894	-1.4448324942941175	1.0906004664117648
H	-0.29523742764705885	3.6811158057058826	1.7723989464117649
H	1.3962382723529410	0.37601842370588234	-1.3178419535882351
H	-1.3848996476470590	-1.7860582942941177	-1.1888551535882352
H	-2.0683627647059000E-002	-1.9022349942941177	-2.2152617535882353
H	-0.88021900764705896	-3.8663181942941174	-0.39254344358823517
H	1.4873785723529411	-1.6308633942941175	0.11104635641176477
H	0.12063837235294117	-0.97466329429411758	1.8254041464117647
H	2.5199473723529415	1.3775651057058824	2.1169406464117646

C	0.68353190882352921	9.0410297647058879E-002	-0.29135718570588232
C	1.1655578088235292	1.0368920776470589	0.78807439429411763
C	0.36183980882352906	2.0742557376470590	0.88212311429411761
C	-0.78434294117647085	1.8097454376470590	-4.8677285705882339E-002
O	-0.57522509117647092	0.66536745764705885	-0.71910081570588236
O	2.4161630088235295	0.81649095764705892	1.3352806142941178
O	0.51269740882352899	3.1611700376470591	1.6813268142941178
O	-1.8841787011764710	2.3845657376470588	-4.9367565705882327E-002
C	0.36534100882352916	-1.3450194623529410	0.18224463129411766
C	-0.29101159117647080	-2.0554336623529412	-0.99787978570588243
O	-0.14820439117647077	-3.4735618623529412	-0.92279968570588233
O	-0.54716179117647079	-1.3675177623529411	1.2204599842941177
H	-0.36448149117647088	3.6200469376470590	1.9159498142941178
H	1.2636488088235289	3.4071457647058878E-002	-1.2419416857058823
H	-1.3483814711764710	-1.8075171623529411	-0.92076498570588228
H	0.14459930882352912	-1.7227506623529409	-1.9735107857058822
H	-0.97039160117647083	-3.9212155623529408	-0.84005958570588246
H	1.3632164088235292	-1.6956224623529410	0.34414690429411765
H	-7.2448391176470839E-002	-1.3973884623529411	2.0615659142941176
H	2.5350085088235295	1.4298046376470590	2.0608176142941175

C	0.64573598576470603	8.1653425294117649E-002	-0.29756749558823531
C	1.3215721857647060	1.0099090352941176	0.68685106441176469
C	0.46207728576470597	1.9978017352941178	0.97034111441176463
C	-0.74589437423529392	1.8348472352941176	9.8639104411764683E-002
O	-0.59817625423529397	0.71248123529411767	-0.59371735558823535
O	2.4787829857647061	0.61199641529411763	1.1295834644117646
O	0.48966585576470623	3.0383442352941179	1.8295210844117646
O	-1.7183957542352939	2.6374743352941179	7.5391674411764698E-002
C	0.28358878576470614	-1.3047921647058822	0.19711314941176469
C	-0.24042131423529400	-2.1052214647058825	-0.99018891558823530
O	-0.48567911423529386	-3.5259342647058824	-0.86259781558823545
O	-0.64220742423529398	-1.2261690647058823	1.2265743044117647
H	-0.34892821423529385	3.5079425352941178	1.6313575844117647
H	1.1430770857647059	1.4194365294117661E-002	-1.2903979155882355
H	-1.1794509642352939	-1.6323761647058823	-1.3090636155882354
H	0.58601108576470606	-2.0816991647058822	-1.7451882155882354
H	-1.4513585722352940	-3.5704522647058825	-0.75665121558823545
H	1.2110386857647062	-1.8490746647058822	0.49615867441176470
H	-0.18479141423529399	-1.0552770647058822	2.0854547844117648
H	2.7889932857647057	1.3047884352941177	1.7723510844117647

C	0.64540436599999995	8.7210031176470609E-002	-0.26937498864705878
C	1.1787250659999999	0.99211066117647051	0.80892902135294120
C	0.32091716599999964	1.9528818511764705	1.0828589013529413
C	-0.83485830400000027	1.7495285511764704	0.17032384835294123
O	-0.53267282400000027	0.82702863117647052	-0.74011784864705876
O	2.3707864659999993	0.66959081117647057	1.3905985113529413
O	0.41228676599999980	3.0835757511764710	1.8001330113529412
O	-1.8419350640000003	2.4136549511764707	0.25051671935294123
C	0.24222556599999967	-1.2907365488235294	0.18399772335294123
C	-5.0760034000000287E-002	-2.1494732488235293	-1.0588197886470587
O	-0.58874434400000031	-3.4243127488235294	-0.74564715864705877
O	-0.84331472400000029	-1.3208228488235294	1.0535209913529413
H	-0.41743703400000021	3.6019974511764707	1.5801645113529412
H	1.3473131660000000	0.18656905117647060	-1.0882667886470587
H	-0.87300122400000024	-1.6678788488235294	-1.7027405886470588
H	0.88901056599999984	-2.2312973488235293	-1.4984806886470587
H	-1.4239455760000004	-3.4796261488235292	-1.2175953886470587
H	1.1703866659999995	-1.6976818488235295	0.70833181135294121
H	-0.51004943400000036	-0.98536554882352956	1.9425013113529412
H	2.5507026660000003	1.2856726611764706	2.0591936113529412

C	0.70406860823529449	0.15090290882352939	-0.39958032300000002
C	1.2397159082352944	1.0210121788235293	0.74230524700000000
C	0.37541670823529438	2.0109020688235293	0.98655627699999993
C	-0.72408197176470557	1.8832358688235293	-3.3370922999999997E-002
O	-0.44874174176470560	0.92232768882352945	-0.88596065300000004
O	2.4446809082352945	0.88603365882352936	1.2419261470000000
O	0.56377070823529429	3.0495609688235294	1.8541913470000000
O	-1.7123714217647057	2.5788975688235296	-0.20320881300000002
C	0.32215520823529431	-1.2626727311764707	0.14029388799999998
C	-0.29919349176470567	-2.1685841311764706	-0.94474505300000000
O	-0.72463706176470566	-3.4802098311764706	-0.52145388300000006
O	-0.56465958176470565	-1.2240412311764706	1.2027299069999999
H	-0.30361819176470561	3.3026114688235295	2.2569026470000000

H	1.3327201082352942	-7.5183461176470623E-002	-1.2225969529999998
H	-1.1501401417647057	-1.5953580311764706	-1.3941575529999999
H	0.42513450823529442	-2.2758166311764705	-1.7730811529999999
H	-1.4802190617647055	-3.7236183311764708	-1.0467501529999998
H	1.2751895082352944	-1.6944726311764706	0.48656699699999995
H	-0.31086559176470563	-0.70814673117647065	1.9563746470000001
H	2.6910162082352942	1.5468960688235294	1.9596791470000001

C	0.64866689700000002	8.3082382352941186E-002	-0.35294213258823537
C	1.3450106970000000	0.96881795235294121	0.63311438741176473
C	0.52662309699999987	2.0366489723529413	0.94107843741176467
C	-0.67583517300000007	1.8769929723529413	9.1073694411764688E-002
O	-0.64429999300000007	0.78318847235294120	-0.69988651258823531
O	2.5514694970000003	0.50797679235294124	1.0654371774117648
O	0.59964819700000005	3.1438704723529414	1.6988551874117646
O	-1.6772729829999999	2.5557433723529410	0.11539529641176469
C	0.25541999699999995	-1.2961928276470589	0.22405588541176469
C	-0.38202111300000008	-2.0526076276470588	-0.94504281258823530
O	-0.59925788300000005	-3.4355790276470590	-0.44249593258823539
O	-0.70022960300000003	-1.1756402276470588	1.3269214874117647
H	-0.19841980299999995	3.7114011723529412	1.6708768874117648
H	1.2315010970000000	-4.3152667647058829E-002	-1.2593069125882352
H	-1.3577665420000000	-1.5767078276470587	-1.1795270125882353
H	0.19700679700000001	-2.0895144276470590	-1.8820631125882352
H	-1.1202431830000001	-3.9983279276470589	-1.0055440125882353
H	1.1872832969999998	-1.7564473276470589	0.56343456741176468
H	-0.14249390300000009	-0.83524562764705879	2.0285966874117647
H	2.9066035970000001	1.0742347623529414	1.7664194874117647

C	0.60434515047058834	7.5588339764705956E-002	-0.37707662029411770
C	1.2594966504705882	0.97733952876470598	0.66009225970588237
C	0.47796105047058846	2.0557590587647061	0.91722950970588235
C	-0.68850905952941166	1.9333379587647059	3.4408227705882297E-002
O	-0.57575045952941162	0.88565052876470596	-0.81813158029411770
O	2.4611338504705884	0.56196111876470600	1.1472946497058822
O	0.62286115047058854	3.0002769587647062	1.8472260497058821
O	-1.6573006895294116	2.6602513587647056	-2.5219653294117704E-002
C	0.22764775047058827	-1.3035596412352941	0.23223457970588229
C	-0.33543424952941159	-2.1369031412352939	-0.88925470029411768
O	-0.62567690952941157	-3.4716078412352944	-0.43995652029411769
O	-0.79092376952941157	-1.1511336412352939	1.2110183497058822
H	-3.3825549529411525E-002	3.7041826587647062	1.6658148497058822
H	1.2757883504705883	-9.7346021235294011E-002	-1.2457918502941179
H	-1.2862673495294117	-1.6286288412352941	-1.1699616502941177
H	0.37984635047058846	-2.2257215412352940	-1.7453445502941178
H	-1.3153922675294116	-3.8394468412352936	-1.0045813502941179
H	1.1980144504705883	-1.7070552412352940	0.57837793970588236
H	-0.35023184952941167	-0.79684648123529400	2.0059536497058823
H	2.6620892504705882	1.1154652787647059	1.9739983497058822

C	0.53319859005882342	0.15444319207058832	-0.42036340029411767
C	1.2327869900588233	0.94057116837058830	0.65619711970588235
C	0.50828099005882343	2.0877501383705885	0.87495243970588232
C	-0.74668518994117650	2.0619959383705884	4.1068282705882336E-002
O	-0.65894732994117655	0.94016203837058832	-0.72043117029411763
O	2.4157281900588234	0.54863319837058833	1.1196448097058824
O	0.77419929005882349	3.0707771383705884	1.8252205997058821
O	-1.7131577699411764	2.8371927383705886	0.14035706270588233
C	0.15913549005882355	-1.2657901616294116	9.2158108705882341E-002
C	-0.25853810994117654	-2.2575874616294116	-1.0368892002941177
O	-0.60286096994117655	-3.4369437616294114	-0.33057887029411764
O	-0.92139826994117646	-1.2090861616294117	1.0512630197058823
H	-7.0428109941176542E-002	3.4832252383705886	2.0649117997058823
H	1.1342157900588234	0.12950324237058833	-1.3232922002941176
H	-1.0558513799411764	-1.8790099616294118	-1.7084313002941176
H	0.68012379005882329	-2.3911499616294116	-1.6646333002941178
H	-1.4098019909411765	-3.8146865616294114	-0.66115380029411763
H	1.0044806900588235	-1.7189656616294118	0.58268747970588231
H	-0.65805501994117654	-0.59951383162941174	1.7531387997058823
H	2.6203258900588238	0.82270188837058833	2.0215603997058822

C	0.46474268729411805	0.14468836029411783	-0.32188365352941173
C	1.1338219872941182	0.97133385529411786	0.71877072647058826
C	0.38878988729411801	2.0148304752941177	1.0402026764705883
C	-0.73265361270588203	1.9732209752941179	0.21754028747058823
O	-0.73344849270588197	0.86848117529411784	-0.57320278352941179

O	2.3094739872941181	0.57313690529411776	1.2026988864705883
O	0.86168868729411807	2.9584972752941177	1.8965492864705884
O	-1.6237032327058820	2.8017689752941175	0.25064757847058827
C	0.14655208729411795	-1.3402500247058822	5.7234123470588247E-002
C	-0.30228521270588193	-2.1056116247058823	-1.2052256135294117
O	-0.29385651270588209	-3.5626986247058818	-1.0067324135294116
O	-0.87438763270588193	-1.4547678247058822	1.0634616664705883
H	0.13795668729411803	3.4458148752941176	2.2871215864705885
H	1.1281656872941181	0.20782740029411784	-1.2034268135294117
H	-1.2646404067058821	-1.7293129247058823	-1.5299826135294117
H	0.48656258729411794	-1.9594789247058821	-1.9347828135294116
H	-1.2327791827058820	-3.8075803247058824	-0.95899011352941177
H	1.1321566872941178	-1.7585501247058821	0.34130176647058824
H	-0.61118387270588204	-1.0613746247058822	1.9081162864705883
H	2.5251903872941179	0.97661961529411778	2.1026709864705881
C	0.65552235064705888	8.5838461764705970E-002	-0.37788765065882357
C	1.2016215506470589	0.93888292176470600	0.68403995934117634
C	0.39638095064705881	2.0042001917647059	0.98493725934117637
C	-0.72794243935294112	1.8689516917647060	4.3618220541176443E-002
O	-0.53636435935294113	0.81567784176470592	-0.77057002065882363
O	2.3571628506470588	0.59359829176470602	1.2747800193411765
O	0.65160685064705870	3.0903140917647063	1.8247027193411764
O	-1.7961031193529411	2.4488893917647059	3.8107966341176441E-002
C	0.25905805064705878	-1.2882665082352940	7.2867462341176442E-002
C	-0.28140344935294115	-2.0324590082352940	-1.1225447806588236
O	-0.54406783935294112	-3.3806374082352941	-0.90378748065882364
O	-0.66935948935294109	-1.3261817082352940	1.1068457193411765
H	-9.0643549352941166E-002	3.1559351917647063	2.5120093193411765
H	1.3167765506470590	1.4845981764705973E-002	-1.2095620806588234
H	-1.2033554093529411	-1.5009015082352941	-1.4389774806588236
H	0.49092375064705895	-1.9627492082352940	-1.8494078806588234
H	-1.4798132503529411	-3.5259387082352940	-0.86917127065882360
H	1.1525047506470587	-1.7668255082352937	0.42231243934117646
H	-0.21623514935294108	-1.0323319082352942	1.9073006193411766
H	2.4323878506470589	1.1736840517647060	2.0637736193411764
C	0.68097796294117630	0.16702011964705879	-0.43099742302941174
C	1.1640509629411764	0.96243982764705882	0.74108190697058829
C	0.30172906294117641	1.9836565976470588	0.97010334697058820
C	-0.79505002705882355	1.7814055976470589	4.7342972970588280E-002
O	-0.45913983705882355	0.83579466764705879	-0.82627702302941164
O	2.2349964629411767	0.62201054764705876	1.4250572369705883
O	0.40303816294117634	2.9316317976470589	1.8722638369705882
O	-1.9119113170588236	2.3387186976470589	8.1708067470588286E-002
C	0.49875746294117640	-1.3307122023529412	-1.4830478029411717E-002
C	-0.18471973705882361	-2.0639217023529408	-1.1262346630294118
O	-0.41847153705882345	-3.4609767023529412	-0.88862923302941166
O	-0.36269273705882354	-1.2863820023529413	1.0951066369705882
H	-0.44729233705882354	3.3621914976470588	2.0356732369705886
H	1.4216935629411764	0.18401716564705881	-1.2393982630294118
H	-1.2180461370588236	-1.6309215023529413	-1.3068451630294118
H	0.31679156294117639	-1.8832762023529412	-2.0763397630294116
H	-1.2247115370588235	-3.5126962023529411	-0.35878523302941173
H	1.4763895629411763	-1.8528404023529415	0.20744656697058828
H	5.1391962941176539E-002	-1.8498143023529414	1.8018000369705882
H	2.2196887629411766	0.93437916764705875	2.3483716369705880
C	0.75599256235294110	0.22723985788235287	-0.44787973764705880
C	1.1994216623529410	0.94108368188235292	0.72156465235294120
C	0.23830676235294113	1.8157599218823530	1.1600759423529412
C	-0.84412097764705896	1.6715465218823529	0.26860477235294117
O	-0.49662903764705901	0.80489852188235278	-0.79450378764705876
O	2.3898803623529412	0.67200152188235285	1.2682647223529411
O	0.42868036235294116	2.6695718218823528	2.2072391223529415
O	-1.9921885276470590	2.1724942218823529	0.32751591235294120
C	0.41495156235294117	-1.2307144781176471	-0.12720747764705884
C	-0.31635503764705897	-1.9721671781176469	-1.2963903776470589
O	-0.50869725764705898	-3.3483901781176475	-0.84428017764705887
O	-0.52951621764705892	-1.2680594781176471	0.95905760235294124
H	-8.6454537647058904E-002	3.4753378218823525	1.9288368223529411
H	1.4072613623529411	0.32488055388235287	-1.3365505776470590
H	-1.2215956576470588	-1.5373521781176471	-1.6579038776470589
H	0.36831766235294117	-2.0818181781176475	-2.1484704776470589
H	-1.2072550476470589	-3.3363127781176471	-0.18797305764705882
H	1.3468911623529412	-1.7343371781176471	6.8442111352941176E-002

H	9.7616623529410695E-003	-1.4676569781176472	1.8159643223529411
H	2.6093562623529407	1.3045722218823530	1.9513680223529410
C	0.62042281117647025	0.21125659517647061	-0.40848643294117642
C	1.1468697111764705	0.91239232517647062	0.83022463705882354
C	0.33650191117647044	1.9291405851764707	1.1348363470588236
C	-0.77194216882352962	1.8692847851764707	0.10817563705882355
O	-0.47348156882352965	0.94086332517647064	-0.81906423294117636
O	2.2532275111764699	0.41072030717647062	1.4346822670588235
O	0.44751001117647027	2.8617256851764710	2.1012081670588234
O	-1.8421429488235297	2.4467002851764708	8.2152587058823556E-002
C	0.38997971117647023	-1.2631164148235294	-0.10840782294117646
C	-0.23220918882352959	-2.0824974148235293	-1.2779089329411764
O	-0.44494646882352962	-3.4140849148235297	-0.88754193294117645
O	-0.66360694882352966	-1.3252041148235294	0.87626371705882344
H	-0.29086578882352976	3.4753343851764704	2.0525761670588234
H	1.2986355111764702	0.11991572517647059	-1.2764430329411764
H	-1.1299822588235298	-1.4954798148235293	-1.5264685329411765
H	0.46217161117647043	-2.0597515148235290	-2.0731245329411765
H	-1.1061414488235297	-3.5371998148235297	-0.24267407294117643
H	1.3054820111764702	-1.7512241148235292	0.24790124205882355
H	-0.36964098882352969	-1.2973257148235293	1.8358732670588236
H	2.2793855111764705	0.76628179517647066	2.3188379670588235
C	0.72755953532352935	0.17784320694117650	-0.45786108588235286
C	1.2846839353235295	0.99153475694117654	0.65507046411764713
C	0.36970843532352959	1.8699123069411765	1.0962158741176471
C	-0.75679844467647051	1.8143203069411764	0.15415611411764713
O	-0.48147209467647045	0.87027213694117656	-0.78956298588235285
O	2.4015128353235293	0.54329321694117649	1.2228352841176471
O	0.56306133532352942	2.7094814069411766	2.1183587141176470
O	-1.8239440646764704	2.4055593069411763	0.17784980411764711
C	0.31617603532352945	-1.2490475930588236	-5.6024365882352878E-002
C	-0.30659986467647049	-1.9829056930588234	-1.1310673858823530
O	-0.34445207467647054	-3.3524116930588237	-0.88995868588235283
O	-0.58967382467647045	-1.1565977930588236	1.0349944841176471
H	-0.26918566467647054	3.0604837069411763	2.4301732141176471
H	1.3029834353235294	0.28775269894117650	-1.3859965858823529
H	-1.3233890641764705	-1.5559702930588235	-1.2400783858823528
H	0.21480943532352947	-1.8394775930588234	-2.0653778858823526
H	-1.2849798856764705	-3.5940423930588237	-0.87372658588235297
H	1.2328457353235294	-1.8059114930588236	0.20465060411764713
H	-0.12063736467647046	-0.97365689305882364	1.8911823141176471
H	2.6554839353235296	0.91422091694117658	2.0900751141176470
C	0.63379053254117701	0.21447833929411769	-0.53112178300588231
C	1.3072057325411770	0.87191446329411770	0.64839009699411765
C	0.49090903254117690	1.8514895532941176	1.1208921969941177
C	-0.73622869745882313	1.8559460532941177	0.26560627199411768
O	-0.52678637745882306	1.0206265532941177	-0.78038719300588233
O	2.4607436325411767	0.32420761129411768	1.0405413169941176
O	0.66689013254117691	2.5967115532941176	2.2306670069941177
O	-1.6833485174588230	2.5739115532941175	0.29158286309411768
C	0.11896603254117699	-1.2280747467058823	-4.7834923005882324E-002
C	-0.54218237745882314	-1.9765661467058822	-1.1247544930058824
O	-0.49330010745882313	-3.4030772467058825	-0.73247619300588229
O	-0.63184028745882315	-1.0376602467058824	1.1450952769941176
H	0.60664653254117695	3.5081574532941175	2.0302842069941174
H	1.1756503325411771	4.0258393294117678E-002	-1.4457367930058822
H	-1.5562010974588232	-1.6436498467058822	-1.3847489930058823
H	0.10055483254117692	-1.9174945467058824	-2.0205044930058826
H	-1.3914693306588231	-3.6511782467058822	-0.70549437300588225
H	1.0704087325411769	-1.8209501467058824	0.12119736699411765
H	-0.18267386745882308	-1.5389314467058823	1.8207128069941176
H	2.6601432325411771	0.60377798329411769	1.8909832069941177
C	0.52598617200000031	0.19038383988235286	-0.59420314000000007
C	1.3263276720000003	0.87889763988235292	0.53342086999999994
C	0.59808827200000025	1.9473693198823527	1.0101337400000001
C	-0.68450852799999984	1.9198611198823530	0.31574279999999999
O	-0.63501210799999974	0.96695656988235290	-0.69332982999999992
O	2.5149240720000003	0.36988369088235284	0.98844381999999997
O	0.89926027200000003	2.8147021198823530	2.0321013699999999
O	-1.6653835679999998	2.6536439198823527	0.53036266999999992
C	0.22535867200000026	-1.2771749801176471	-0.12648923000000001
C	-0.50454635799999981	-2.1247217801176475	-1.1482659300000000

O	-0.50379727799999974	-3.4782584801176473	-0.71549383000000000
O	-0.47083641799999976	-1.2127280801176470	1.1157084500000001
H	0.13610777200000013	3.4407879198823528	2.0260998699999999
H	1.0385131720000003	0.29477712088235286	-1.5916721300000001
H	-1.5299910879999998	-1.6822425801176473	-1.3216454300000000
H	0.15772267200000023	-2.1454032801176472	-1.9812077299999999
H	-1.4282134019999997	-3.5567340801176472	-0.37970634000000003
H	1.0941769720000003	-1.8345565801176473	0.17061073999999996
H	0.11996807200000026	-1.3752193801176471	1.8943977699999999
H	2.6055515720000004	0.80061245988235286	1.8679279700000000

C	0.61002334788235291	0.16117761352941179	-0.38310993300000001
C	1.1995553478823531	0.89481185352941184	0.71643890700000001
C	0.35727364788235305	1.8821157035294118	1.1037722070000000
C	-0.81019475211764691	1.8996269035294118	0.26532781700000002
O	-0.60649729211764691	0.87693191352941180	-0.62130897299999988
O	2.4191921478823533	0.64863609352941176	1.3126045770000001
O	0.60097174788235308	2.8116960035294118	2.1113798269999999
O	-1.8047265521176470	2.6365552035294115	0.32505563800000004
C	0.33641214788235296	-1.3248913964705882	8.6394216999999995E-002
C	-0.16380495211764701	-2.0892845964705886	-1.2282278730000000
O	-0.57806961211764696	-3.4362433964705881	-0.96539277300000004
O	-0.66405504211764699	-1.3017544964705883	1.0409980270000001
H	-0.20940375211764684	3.2854302035294118	2.2936374270000002
H	1.1790783478823530	0.32130238352941182	-1.3451842729999999
H	-0.97346735211764690	-1.5875970964705883	-1.7398772729999998
H	0.62649564788235312	-2.1692332964705878	-2.0355907730000000
H	-1.5187830761176468	-3.5092795964705878	-0.93691677299999987
H	1.2707048478823533	-1.7417652964705883	0.42532492410000000
H	-0.42193005211764700	-1.8507516964705883	1.7966757270000000
H	2.3980946478823535	1.0385562435294118	2.2280502269999998

C	0.58013931170588262	0.15303384611764692	-0.34450451941176469
C	1.1941590117058827	0.90464436611764687	0.77326056058823522
C	0.43464071170588237	2.0440118961176470	1.0317011605882354
C	-0.74306140829411749	1.9071592961176469	0.12873708058823527
O	-0.62741357829411748	0.82787619611764696	-0.64603652941176470
O	2.2632273117058821	0.36578078211764692	1.3959615705882353
O	0.54257311170588252	3.0949279961176468	1.8374402705882353
O	-1.7270621182941175	2.6603894961176469	5.2749710588235244E-002
C	0.33544421170588246	-1.3545720038823530	0.12718552058823526
C	-0.20912188829411749	-2.2293036038823533	-1.0629401294117646
O	-0.59149824829411757	-3.5451128038823532	-0.78027872941176479
O	-0.62514625829411752	-1.3334374038823531	1.1375331205882353
H	-6.6314488294117568E-002	3.7856433961176466	1.5808619705882354
H	1.1661763117058823	0.10922155611764695	-1.2704509294117647
H	-0.97662025829411758	-1.5788881038823530	-1.5523232294117646
H	0.58842551170588231	-2.2074177038823533	-1.8741374294117648
H	-1.5385472472941175	-3.6039572038823531	-0.53475946941176478
H	1.3118539117058823	-1.6405037038823529	0.53927883058823523
H	-0.19121248829411752	-1.5340139038823530	1.9884049705882354
H	2.4028504117058826	0.65106642611764687	2.2869047705882353

C	0.73534680017647069	0.20724514399999994	-0.35094855647058820
C	1.1412127001764705	0.91926700399999994	0.89805433352941177
C	0.27679010017647054	1.8981951040000000	1.1405808135294118
C	-0.75347175982352954	1.8707450040000000	0.19297927352941174
O	-0.44124864982352952	0.98494924399999995	-0.78153713647058831
O	2.2465068001764705	0.45324017599999994	1.4682520635294118
O	0.29244410017647038	2.7150663040000000	2.2215253635294117
O	-1.8550727398235294	2.4667726040000000	0.15684750352941174
C	0.31090720017647056	-1.2910056960000000	-6.5310656470588258E-002
C	-6.9227799823529557E-002	-2.0318011960000000	-1.3246220364705883
O	-0.52400190982352957	-3.4178537960000002	-0.99133943647058831
O	-0.76065628982352951	-1.2910878960000001	0.84789134352941176
H	-0.52786111982352957	3.1409926040000000	2.3579428635294120
H	1.4114539001764705	0.26419318399999991	-1.1882432364705882
H	-0.83209819982352951	-1.4850980960000000	-1.9012727364705881
H	0.71543740017647028	-2.1891851959999999	-2.0609735364705881
H	-1.3664605328235295	-3.2146344960000000	-0.61982622647058827
H	1.2933978001764705	-1.6572314960000001	0.39191764452941175
H	-0.24257159982352960	-1.3749664960000001	1.6777226635294118
H	2.4296107001764704	0.94569787399999994	2.2750790635294118

C	0.72837479882352962	0.19002310529411759	-0.42823911000000003
C	1.1466975988235295	0.91193026529411758	0.80429675000000000
C	0.20733919882352958	1.8819398352941177	1.1206771899999999
C	-0.82997982117647051	1.7896709352941176	9.8002369999999950E-002
O	-0.56599575117647050	0.86653117529411761	-0.83697126000000011
O	2.2720092988235296	0.61699407529411765	1.3654379300000001
O	0.36346729882352946	2.8113889352941177	2.0703455399999999
O	-1.8758394811764705	2.3918391352941173	0.21441898999999998
C	0.29716579882352945	-1.2736363647058824	-0.10755480000000001
C	-0.16424980117647059	-2.0008828647058827	-1.3447896600000000
O	-0.35487410117647045	-3.3280098647058827	-0.95847806000000002
O	-0.79534618117647049	-1.2690922647058824	0.85893081999999998
H	-0.38875920117647045	3.0617245352941174	2.5851716399999995
H	1.4335139988235297	0.25211295529411759	-1.2321587600000001
H	-1.0435616111764705	-1.4906849647058824	-1.8139352599999998
H	0.67392579882352965	-2.0525514647058820	-2.0371598600000000
H	-1.1038878411764705	-3.3592971647058825	-0.35799446000000001
H	1.1875130988235294	-1.7693372647058825	0.41365867299999998
H	-0.40071989117647044	-1.3524520647058824	1.7680314399999999
H	2.3420131988235293	1.0582938752941176	2.2688981400000001

C	0.67320648294117680	0.19585887205882357	-0.34191232882352940
C	1.2542874829411765	0.94684681205882359	0.81362141117647058
C	0.36157008294117654	1.9026633402058823	1.1317471611764707
C	-0.76961780705882343	1.7351207020588237	0.19639842117647061
O	-0.46979178705882341	0.87591663205882353	-0.76523904882352944
O	2.4161436829411764	0.56466990705882358	1.3135129811764705
O	0.33480618294117659	2.8557044020588238	2.0912307511764707
O	-1.8023722470588235	2.4200481020588236	0.28465367117647061
C	0.28741668294117662	-1.2016404979411766	0.10533640117647058
C	-0.11877391705882334	-2.0055043979411762	-1.2360641488235293
O	-0.53155441705882345	-3.3451639979411762	-0.93354504882352929
O	-0.81006664705882336	-1.2194844979411763	1.0079849211764706
H	-0.58164772705882339	3.0753744020588236	2.1602924511764705
H	1.3764683829411766	0.19309655205882353	-1.2107977488235293
H	-0.94703541705882333	-1.5361833979411763	-1.7171990488235294
H	0.80873218294117644	-2.0778515979411765	-1.7980042488235293
H	-1.4817711970588234	-3.3794713979411761	-1.1020165488235294
H	1.0814902829411768	-1.7679837979411763	0.49997675417647058
H	-0.55958634705882337	-1.1322794979411763	1.9503180511764706
H	2.6968906829411763	1.1238840520588236	2.1199161511764704

C	0.66215779882352965	0.11922002399999998	-0.33764772352941175
C	1.1563336988235293	0.85578018400000000	0.87692504647058822
C	0.21258459882352954	1.8384389839999999	1.1837867364705883
C	-0.83577463117647055	1.8199226840000000	0.15750463647058827
O	-0.63182672117647054	0.86854902400000000	-0.72854545352941169
O	2.2527429988235297	0.45340608599999999	1.4361878864705884
O	0.42359459882352946	2.7183863840000000	2.1889550464705883
O	-1.7457379211764705	2.6307054839999999	0.10706830647058824
C	0.42585098882352944	-1.3760648160000000	-0.15463148352941170
C	-0.22222480117647048	-1.9610227160000000	-1.4150311535294118
O	-0.51524590117647051	-3.3227319160000000	-1.1147394535294119
O	-0.44886944117647054	-1.5917028160000002	0.93882824647058827
H	-0.33260130117647058	3.3961671839999998	2.2698578464705883
H	1.3963227988235296	0.34231907399999995	-1.0904538535294117
H	-1.1300148511764705	-1.4390879160000001	-1.6568818535294114
H	0.50762729882352953	-2.0203210160000000	-2.2355949535294117
H	-1.1749191211764705	-3.3319639159999999	-0.42558782352941171
H	1.4010519988235295	-1.8657034160000001	0.10119025647058827
H	4.6367398823529582E-002	-1.6151615160000001	1.8008880464705883
H	2.4808619988235296	0.86256178399999994	2.2926563464705882

C	0.82241052388235270	0.15252285811764699	-0.32277096117647058
C	1.1641210238823527	0.87309827811764706	0.96000977882352956
C	0.28619922388235297	1.7989144281176470	1.1451419288235294
C	-0.76678814611764712	1.7449565281176469	0.15050574882352946
O	-0.38210537611764717	0.85011411811764703	-0.77408356117647048
O	2.3126207238823531	0.54059098011764695	1.5655704388235294
O	0.36463852388235285	2.8167047281176472	2.0602401388235294
O	-1.8168559161176472	2.3967312281176469	0.10719384882352945
C	0.46295842388235298	-1.3279922718823529	-0.14100151117647058
C	-0.29626757611764720	-1.9411232718823528	-1.3114531611764706
O	-0.52089281611764715	-3.2878804718823531	-1.0386927611764705
O	-0.23178067611764708	-1.5392908718823528	1.1016656788235295
H	-0.51105896611764712	3.3192499281176469	2.0380165388235292
H	1.5249039238823530	0.27947852811764701	-1.1390115611764704
H	-1.2264753161176472	-1.3443377718823530	-1.4454422611764706

H	0.23510182388235279	-1.8469933718823528	-2.2485387611764707
H	-1.4207294021176471	-3.4847435718823530	-0.70734956117647041
H	1.4090946238823530	-1.8022339718823530	-0.14921775117647057
H	0.32413612388235280	-1.9755586718823528	1.7544048388235296
H	2.5220803238823533	1.1954881281176470	2.2777926388235294

C	0.88300163094117634	0.17489121294117654	-0.35198611470588231
C	1.3547162309411762	0.99495103294117659	0.78801141529411767
C	0.29032463094117622	1.8058068229411766	1.1664903052941176
C	-0.84323612905882372	1.7679707229411765	0.17718993529411767
O	-0.34468876905882362	0.84999115294117655	-0.73484599470588230
O	2.4910021309411761	0.70769681294117659	1.4273385052941177
O	0.15767303094117624	2.5279376229411765	2.3074796052941178
O	-1.9268425690588238	2.2903165229411764	4.6187255294117657E-002
C	0.49902003094117631	-1.3271747770588234	-8.5026624705882348E-002
C	-0.26891306905882373	-1.8727767770588233	-1.2523933947058823
O	-0.53138380905882376	-3.2986607770588234	-1.1539012947058822
O	-0.34497396905882383	-1.3659656770588235	1.0207097752941177
H	-0.69592229905882375	2.9911788229411762	2.2961583052941177
H	1.5881708309411762	0.23481531294117652	-1.2505966947058822
H	-1.1975900490588236	-1.3294811770588235	-1.4361853947058822
H	0.30874813094117637	-1.7060469770588236	-2.1643295947058823
H	-1.4191059850588237	-3.4454498770588238	-0.80029999470588220
H	1.4081720309411763	-1.9347032770588235	2.1917285294117683E-002
H	9.6264330941176368E-002	-1.1345158770588235	1.8476767052941176
H	2.5236857309411764	1.2360885729411766	2.2652393052941178

C	0.76708436482352949	0.22624023882352950	-0.49379624482352941
C	1.3686757648235297	1.0473928688235294	0.63281526517647047
C	0.34464156482352970	1.8364744088235296	1.1120205551764706
C	-0.81159018517647041	1.7271081088235296	0.14340416517647056
O	-0.47498728517647038	0.88341209882352956	-0.82402918482352949
O	2.5093868648235294	0.64817326882352955	1.2414132051764706
O	0.23270466482352958	2.5456930088235294	2.2646940151764707
O	-1.9024289751764702	2.2420047088235293	0.23020529717647054
C	0.45389156482352955	-1.2572916911764707	-3.3317054823529479E-002
C	-0.34682433517647038	-1.9595252911764702	-1.1790716848235294
O	-0.65089512517647041	-3.3284351911764705	-0.86245148482352940
O	-0.41389286517647039	-1.3016212911764704	1.0241540251764705
H	-0.12920753517647032	3.4578686088235298	2.0712359151764708
H	1.3159049648235295	0.13544341882352950	-1.4238418848235295
H	-1.1847609151764704	-1.3627729911764703	-1.5172445848235294
H	0.33147236482352960	-2.0879581911764702	-2.0679794848235296
H	-1.4091748971764704	-3.4522060911764703	-0.31821083482352946
H	1.3710916648235296	-1.7899190911764702	0.14579007517647055
H	7.5508648235296238E-003	-1.2787096911764704	1.9092436151764705
H	2.7942483648235292	1.3210289788235294	1.8704298151764704

C	0.76058667000000035	0.24365971034705886	-0.39324953035294119
C	1.1926003700000001	0.97061884034705881	0.82331581964705880
C	0.27830657000000025	1.8238992103470588	1.2711988896470587
C	-0.79075233999999972	1.7689309103470590	0.23619542164705878
O	-0.49304904999999977	0.89008396034705894	-0.79445023035294127
O	2.3398700700000004	0.47368044444705887	1.3654530696470588
O	0.15738957000000031	2.6438081103470590	2.3111985696470585
O	-1.8995766799999996	2.2849989103470589	0.38150957364705879
C	0.42979997000000036	-1.2941456896529411	-0.10266236035294124
C	-0.14507382999999963	-1.9700501896529410	-1.3408313303529411
O	-0.51646130999999973	-3.3056195896529412	-1.0110817303529411
O	-0.50836063999999970	-1.4089792896529412	0.88448460964705877
H	-0.46279001999999969	3.3168661103470591	1.9755392696470588
H	1.5317266700000003	0.29941612034705889	-1.2054329303529412
H	-0.96639964999999972	-1.3817622896529411	-1.6298013303529413
H	0.54538227000000017	-2.1359084896529410	-2.1083073303529414
H	-1.4531986399999997	-3.2194967896529412	-0.66307845035294122
H	1.3976136700000001	-1.7133721896529410	0.19935197764705878
H	1.1743970000000381E-002	-1.6155078896529411	1.6949101696470588
H	2.7869149700000007	1.0372610203470589	2.0384895696470586

C	0.77526596235294121	0.22397636317647063	-0.40242255000000005
C	1.2327168623529412	0.93478168317647059	0.85023843999999993
C	0.20057786235294128	1.6922389431764706	1.3444845200000000
C	-0.90275334764705861	1.6029018431764706	0.38024572000000001
O	-0.49899395764705867	0.84918443317647063	-0.68348355000000005
O	2.4094797623529409	0.51221782917647063	1.3762572199999998
O	0.15032516235294135	2.5342474431764708	2.3950389200000002
O	-1.9680913376470586	2.2107140431764707	0.36632591999999997
C	0.36493636235294136	-1.2563896568235293	3.4163899999999969E-002
C	-1.8516376470585350E-003	-1.9522232568235292	-1.3114075800000000
O	-0.67921292764705865	-3.2275554568235294	-1.2456090799999999
O	-0.72023704764705865	-1.1498074568235293	0.93955361999999998
H	-0.53578111764705860	3.2206282431764706	2.2291741200000001
H	1.4303819623529412	0.14223937317647062	-1.2915089800000001
H	-0.59074953764705862	-1.2183907568235293	-1.8497107800000001
H	0.93290596235294143	-2.1098610568235294	-1.8954164799999997
H	-1.5989189876470586	-3.0089025568235295	-1.2359233800000000
H	1.2303153623529413	-1.6810704568235293	0.50091079999999999
H	-0.39804572764705859	-1.2116787568235294	1.8797900200000002
H	2.6688030623529411	1.0650044631764706	2.1430414199999999

C	0.79068942717647084	0.23470432388823534	-0.37232873235294117
C	1.1632119271764709	0.88598607388823536	0.92358145764705890
C	0.14762162717647054	1.6789181538882352	1.3603107764705589
C	-0.91923852282352914	1.5947439538882353	0.36438903764705888
O	-0.45367505282352905	0.91753811388823525	-0.76528588235294115
O	2.3501077271764705	0.60331023388823535	1.4948417776470588
O	7.8687927176470884E-002	2.4555875538882352	2.4915998776470585
O	-2.0244600228235292	2.1361798538882355	0.37124160764705882
C	0.43591682717647084	-1.2528335461117648	-0.23173016235294117
C	-0.10313917282352913	-1.9435307461117648	-1.4577904223529412
O	-0.52386527282352913	-3.2326990461117648	-1.0542783223529411
O	-0.46783768282352911	-1.3133110461117647	0.84132589764705890
H	-0.27777737282352910	3.3324305538882353	2.3192928776470585
H	1.5421164271764709	0.42294640778823533	-1.1765003223529411
H	-0.91331211282352909	-1.3396339461117648	-1.8822228223529411
H	0.64219632717647079	-2.0165536461117646	-2.2866222223529413
H	-1.4672430048235290	-3.1637832461117648	-0.93982452235294112
H	1.3719635271764707	-1.7307054461117648	-5.0505072352941160E-002
H	-0.25210727282352918	-2.1320825461117647	1.2915909776470589
H	2.4470015271764707	1.0923021138882354	2.3507579776470586

C	0.73409682941176491	0.20150160982352941	-0.45163180394117652
C	1.2352261294117650	0.95632649982352946	0.78777910605882351
C	0.23342772941176482	1.8600101198235293	1.1825513360588236
C	-0.91045462058823512	1.6359537198235294	0.21975489305882348
O	-0.50884563058823518	0.83552935982352938	-0.76764522394117651
O	2.3074248294117652	0.46890497582352941	1.3989128360588237
O	0.13287912941176483	2.5905862198235297	2.3261512360588235
O	-2.0759513205882349	2.0979832198235293	0.28708883605882352
C	0.41073432941176491	-1.2542157801764706	-0.12139574394117653
C	-9.8760470588235227E-002	-1.9864328801764708	-1.3166449639411764
O	-0.51568796058823518	-3.2880548801764706	-0.86971076394117641
O	-0.65694915058823511	-1.3169185801764707	0.85550500605882351
H	-3.2685570588235180E-002	3.5161204198235296	2.0783334360588235
H	1.3461866294117650	0.32698991682352940	-1.3380101639411763
H	-0.94586579058823517	-1.4732282801764707	-1.8217651639411767
H	0.67739452941176492	-2.0590113801764702	-2.0633061639411765
H	-1.3321696205882352	-3.1120442801764705	-0.38596669394117655
H	1.2712091294117649	-1.7644200801764705	0.25018767605882347
H	-0.37035337058823514	-1.5265593801764707	1.7794580360588235
H	2.6826310294117652	1.1997574598235294	1.9672756360588233

C	0.56994120823529393	0.18370665235294109	-0.40177679700000002
C	1.0627577082352939	1.0216588423529411	0.69740967300000001
C	7.0753308235294021E-002	1.8814919523529410	1.1199540630000000
C	-1.0581514817647060	1.6482198523529412	0.23919016599999998
O	-0.70516440176470596	0.79459984235294101	-0.73528174699999993
O	2.2267719082352939	0.79964858235294112	1.2459428530000001
O	0.14961570823529402	2.7632670523529410	2.1492081530000000
O	-2.1373683417647058	2.1851898523529409	0.29082414899999998
C	0.35231010823529396	-1.2874503476470589	3.4650713000000000E-002
C	0.13348870823529402	-2.0166012476470589	-1.2187742470000000
O	-0.25739929176470588	-3.3330097476470590	-1.0284651469999999
O	-0.77771564176470598	-1.4816291476470589	0.80475900300000003
H	-0.51610347176470595	3.4569119523529408	1.9904861530000000
H	1.2634463082352938	0.21802755235294108	-1.2119068470000001
H	-0.54616508176470602	-1.4562330476470589	-1.8369844469999999
H	1.0633607082352938	-2.0074872476470591	-1.8121254470000001
H	-0.89437796176470596	-3.3703113476470592	-0.32711024700000002
H	1.2890788082352942	-1.6291873476470591	0.45580293299999997
H	-0.56956311176470598	-1.4796190476470592	1.7602662529999999
H	2.2283173082352938	1.3560183423529411	2.0153696530000000

C	0.65535897929411768	0.17529510147058830	-0.31036428964705887
C	1.1467195792941176	0.90590334147058837	0.89443291035294115
C	0.33147117929411762	1.9386350514705883	1.1930451103529411
C	-0.89322265070588247	1.6771756514705882	0.32691455435294114
O	-0.64092786070588237	0.81042098147058828	-0.70895838964705882
O	2.3506642792941177	0.62162147647058830	1.4136311303529412
O	0.36691307929411754	2.9969732514705885	2.0075595303529412
O	-1.9748714707058825	2.1906078514705882	0.53664533035294115
C	0.28905157929411751	-1.2920975485294117	-7.5949349647058850E-002
C	-0.11966672070588236	-2.0880821485294114	-1.3436108696470588
O	-0.43086728070588243	-3.4324957485294116	-0.96950726964705880
O	-0.82569837070588237	-1.3452897485294115	0.81846365035294111
H	-0.23717131070588238	3.6907124514705885	1.6540371303529411
H	1.2734324792941174	0.37870228147058832	-1.2010206696470589
H	-0.92841428070588239	-1.6627975485294115	-1.9153335696470588
H	0.77855397929411740	-2.1696600485294115	-1.9677481696470589
H	-1.1413251887058824	-3.3956246485294117	-0.35223676964705886
H	1.2260007792941177	-1.7478150485294117	0.28396515853250615
H	-0.50940218070588239	-1.5528142485294114	1.6952163303529411
H	2.3511308792941175	1.0228387614705883	2.2908725303529409

C	0.72151559705882362	0.21830194611764714	-0.42902279058823528
C	1.0757282970588233	0.94570873611764705	0.85076888941176465
C	0.14159959705882352	1.8865801461176472	1.1143112694117647
C	-0.94318968294117655	1.7333631461176471	0.17906628941176470
O	-0.54590497294117646	0.88773685611764708	-0.81566525058823536
O	2.1653668970588233	0.68552935611764709	1.6122862494117647
O	0.11507159705882342	2.7790921461176472	2.1812998494117646
O	-1.9908502529411765	2.3412582461176470	0.14246145941176469
C	0.52800969705882350	-1.3180748538823530	-0.11129505058823530
C	-8.1789102941176584E-002	-2.0793433538823529	-1.2724243505882353
O	-0.14207380294117655	-3.4494372538823530	-1.0208875505882353
O	-0.43800962294117651	-1.5206989538823530	0.92353154941176463
H	-0.43391862294117656	3.5286031461176468	1.8862603494117647
H	1.4455174970588234	0.43135265211764712	-1.2569593505882353
H	-1.1173100929411766	-1.6660752538823531	-1.4011468505882352
H	0.43174239705882345	-1.8062935538823530	-2.1537750505882354
H	-0.93150542294117655	-3.5976031538823530	-0.42880966058823528
H	1.5136982970588233	-1.6963178538823529	0.29576581941176466
H	-0.13664910294117649	-1.3286188538823529	1.8294475494117648
H	2.3000295970588231	1.3773137861176470	2.3255442494117649

C	0.65480258941176461	0.21105603535294126	-0.42793747705882357
C	1.2444540894117646	0.89946903535294132	0.84217016294117641
C	0.33537648941176457	1.7632730053529411	1.3207159329411764
C	-0.83622321058823545	1.5820339053529411	0.55663568294117649
O	-0.64418615058823536	0.82968851535294119	-0.51431013705882356
O	2.4697781894117643	0.58540641435294127	1.3236824329411765
O	0.40456208941176452	2.5770691053529413	2.3999597329411761
O	-1.9409590005882356	2.1666709053529414	0.77631888294117646
C	0.44806548941176461	-1.3160631946470587	-0.36194374705882359
C	-0.29556402058823539	-1.7821903946470585	-1.6198415670588235
O	-0.40129255058823543	-3.2379915946470588	-1.4068126670588235
O	-0.43285805058823545	-1.6062613946470585	0.73779542294117650
H	-0.33182645058823546	3.2514552053529413	2.4570268329411764
H	1.2712077894117646	0.37928333535294123	-1.2870043670588236
H	-1.3183909305882353	-1.3734926946470587	-1.7515818670588235
H	0.34639018941176469	-1.6602459946470587	-2.4546453670588235
H	-0.97333655058823543	-3.2691601946470588	-0.59022788705882356
H	1.3729074894117648	-1.7666284946470587	-0.27559724705882360
H	8.9368989411764632E-002	-1.8352244946470586	1.5556178329411765
H	2.7628359894117649	1.1973755653529412	2.0778597329411763

C	0.60644742764705861	0.25178325941176471	-0.53286010823529417
C	1.2826024276470589	0.84237448941176474	0.67878440176470589
C	0.42636022764705861	1.6859415194117648	1.2961975017647058
C	-0.82863258235294124	1.7663231494117646	0.53371912176470593
O	-0.70768046235294124	0.93596512941176468	-0.57131409823529411
O	2.4751432276470586	0.41505803941176472	1.0819414517647059
O	0.63546052764705863	2.4487418494117645	2.3923548017647058
O	-1.8596789223529413	2.4203898494117646	0.79252844176470583
C	0.34049382764705860	-1.2455803505882352	-0.30806775823529409
C	-0.32375660235294135	-1.8597191505882353	-1.5224363982352942
O	-0.42526596235294134	-3.2595545505882355	-1.1689444982352941
O	-0.35814692235294132	-1.4758699505882356	0.91951695176470583
H	-0.17968797235294121	2.9022711494117646	2.5856505017647060
H	1.1051194276470588	0.55426171941176472	-1.4787484982352941
H	-1.3425935523529413	-1.5250748505882354	-1.7967783982352943
H	0.29739502764705872	-1.6778400505882356	-2.3768902982352942
H	-1.1435791423529413	-3.1794712505882354	-0.52465311823529415
H	1.3524759276470588	-1.7268634505882354	-0.22410592823529407
H	0.11565482764705881	-2.2206199505882354	1.3348378517647059
H	2.5837071276470587	0.77761651441176471	1.9485368017647058

C	0.61405907323529396	0.27224000294117634	-0.51535538897647060
C	1.3149803732352940	0.97129461294117636	0.69689912102352936
C	0.43776477323529384	1.8568236629411763	1.1889848410235295
C	-0.84774460676470609	1.7238583629411763	0.40231670362352945
O	-0.71717751676470609	0.83544643294117638	-0.61242565897647072
O	2.5153204732352941	0.60670789294117633	1.2262148710235294
O	0.35991137323529387	2.6904183629411764	2.2906674410235297
O	-1.8262803967647061	2.4057038629411762	0.68015686102352935
C	0.44647037323529393	-1.2382080370588238	-0.31368276897647057
C	-0.33716382676470613	-1.9519356370588239	-1.4141478589764707
O	-0.36350305676470607	-3.3725682370588235	-1.0314547589764707
O	-0.31063422676470620	-1.5221856370588236	0.82985370102352940
H	-0.43969722676470613	3.3306216629411765	2.0870732410235293
H	1.2256121732352940	0.38462220294117633	-1.4796834589764707
H	-1.3973944417647062	-1.6248365370588236	-1.4606852589764707
H	0.18728847323529396	-1.9317168370588236	-2.3619912589764702
H	-0.86181178676470616	-3.4362861370588238	-0.21274036897647058
H	1.4655665732352938	-1.6381471370588236	-0.35624665897647062
H	0.17615767323529385	-1.5030187370588235	1.6676277410235294
H	2.6723341732352939	1.2595452529411764	1.9064142410235294

C	0.64725943647058837	0.32282358705882358	-0.63175286516470586
C	1.1905196364705881	1.0379294670588235	0.64036168483529410
C	0.26127143647058815	1.7534424870588237	1.2319394448352943
C	-0.81906807352941169	1.8775441870588234	0.24441263263529414
O	-0.59083855352941173	1.0060441970588236	-0.82353470516470595
O	2.4053374364705880	0.74098679705882353	1.1015907448352942
O	0.33426773647058816	2.5859796870588236	2.3511818948352943
O	-1.8130812835294117	2.5921427870588234	0.29157589483529417
C	0.38557963647058835	-1.1357370129411763	-0.27044946516470592
C	-0.28764006352941185	-1.9714621129411767	-1.3266203051647059
O	-0.26967946352941174	-3.3939799129411767	-0.98319820516470591
O	-0.39058086352941168	-1.3549235129411765	0.90270850483529408
H	-0.38125166352941164	2.5234946870588235	3.0188413948352943
H	1.3117868364705882	0.40032240705882360	-1.4901326051647059
H	-1.2307694635294117	-1.5960446129411765	-1.6738053051647059
H	0.32852703647058834	-1.8590539129411767	-2.1963186051647057
H	-1.0816397635294117	-3.5295092129411767	-0.38680013516470590
H	1.4151250364705883	-1.6323206129411765	-0.14783053516470587
H	0.16821033647058825	-1.0768601129411763	1.7301545948352941
H	2.4893059364705881	1.2656221370588234	1.9222776948352942

C	0.61185046764705886	0.34915509811764711	-0.53986125448235289
C	1.0333607676470589	0.91263133811764707	0.80599302551764707
C	0.26110306764705893	1.9714232581176470	1.1446051855176471
C	-0.92927182235294115	1.8573652581176470	0.19952851751764705
O	-0.65701549235294110	0.92821406011764707	-0.81329193448235304
O	2.1116920676470587	0.39425215811764708	1.4396343655176471
O	0.39080196764705888	2.9570497581176474	2.0548551655176470
O	-2.0260182023529412	2.3921170581176470	0.27140249971764707
C	0.41716406764705893	-1.1942648418823527	-0.28549160448235295
C	-0.27289293235294121	-1.9985593418823528	-1.4122728344823530
O	-0.28671953235294123	-3.3915601418823527	-1.0554674344823529
O	-0.38983633235294124	-1.4169828418823527	0.84610508551764707
H	0.23606806764705879	2.6845717581176469	2.9475238655176472
H	1.3457400676470588	0.49462174811764709	-1.4026232344823530
H	-1.2316677923529411	-1.6048332418823530	-1.5244174344823529
H	0.42628876764705881	-1.7985308418823527	-2.2402603344823526
H	-1.0406472023529412	-3.5366702418823528	-0.43596164448235292
H	1.4327291676470590	-1.6022913418823528	-0.22419534448235295
H	0.11984536764705878	-1.1307711418823527	1.6163085655176470
H	2.3625600676470588	0.91960525511764712	2.2551870655176471

C	0.68548337794117598	0.27954365758823518	-0.53634001647058827
C	1.0973545779411762	1.0596217735882352	0.64821218352941168
C	0.14941137794117609	1.9758352675882351	0.99237067352941177
C	-0.96546268205882391	1.8192038575882350	-2.1785016470588336E-002

O	-0.52189850205882393	0.99194627258823520	-0.99259871647058828
O	2.1120654779411758	0.61717552758823513	1.4392974835294117
O	0.19443937794117616	2.8724472675882353	2.0037974835294117
O	-2.0595606820588239	2.3625609675882351	5.1662923529411681E-002
C	0.49234517794117605	-1.2472744324117651	-0.27240170647058831
C	-0.40215892205882398	-1.9292837324117649	-1.2806323164705884
O	-0.32464752205882386	-3.3801341324117646	-1.0167613164705884
O	-0.22154342205882394	-1.4495523324117650	0.94790109352941165
H	0.44241417794117610	2.4514051675882351	2.8192158835294117
H	1.4530420779411759	0.44472386758823512	-1.3386678164705883
H	-1.4104551170588240	-1.4745199324117650	-1.0411120164705883
H	-2.8456022058823915E-002	-1.7997958324117649	-2.2731818164705886
H	-0.69237275205882398	-3.5939032324117650	-0.12897698647058831
H	1.5085814779411761	-1.6882239324117649	-0.24729178647058830
H	0.30085867794117616	-1.0676814324117649	1.7006469835294116
H	2.6256330779411758	1.4200339075882351	1.6797622835294117

C	0.62537920058823526	9.7586364294117711E-002	-0.35735522823529403
C	1.0098519005882354	1.0343992112941176	0.81200532176470586
C	0.10896520058823533	2.0721861142941176	0.98548697176470590
C	-0.94066605941176473	1.7772028442941177	4.4627221764705904E-002
O	-0.65364418941176472	0.75265715429411773	-0.77651904823529416
O	2.0777823005882352	0.66806106429411771	1.4578806517647058
O	-8.2073299411764777E-002	3.0117880142941176	1.9115208517647058
O	-2.0205128894117648	2.3118355142941178	-8.5082838235294056E-002
C	0.47709600058823520	-1.3716414857058825	-6.4523382352940639E-003
C	-0.18884979941176483	-2.1325395857058824	-1.1918128482352941
O	-0.25502289941176470	-3.5484195857058825	-0.83225544823529418
O	-0.38641456941176477	-1.4477483857058826	1.0906314917647060
H	0.23836650058823516	3.8391596142941178	1.5817503517647058
H	1.3544854005882354	0.27522710429411767	-1.1765594482352941
H	-1.0954109794117648	-1.6510925857058822	-1.3914838482352940
H	0.51128490058823517	-2.0074198857058825	-2.0418102482352940
H	-0.78061671941176480	-3.6812414857058822	-2.4571568235294106E-002
H	1.5149290005882352	-1.8499259857058825	0.22420429176470591
H	0.18117300058823527	-1.4872323857058825	1.8624622517647058
H	2.2478632005882351	1.2009321742941177	2.2351950517647059

C	0.63629340411764712	0.16944146988235309	-0.33156292470588233
C	0.99930200411764725	1.0709438598823531	0.82815640529411760
C	-3.3914595882352572E-002	1.9504687598823529	1.0517414952941175
C	-1.0218045258823527	1.7219653398823529	-1.6546404705882367E-002
O	-0.63280517588235263	0.63134534988235314	-0.79703587647058227
O	2.1797301041176476	0.90412497188235308	1.4084457152941177
O	-0.13677329588235265	2.8969408598823532	2.0331578152941177
O	-2.0743867458823528	2.3365650598823531	-0.18615135470588234
C	0.49611930411764726	-1.3463067401176469	5.5742035294117642E-002
C	-2.7562795882352553E-002	-2.0047213401176469	-1.1513871847058823
O	-6.6694795882352720E-002	-3.4597198401176468	-1.0715686847058823
O	-0.34192739588235255	-1.6369350401176468	1.0764663452941177
H	-0.70800586588235270	3.6429257598823530	1.7929019152941177
H	1.4147475041176474	0.21986874988235305	-1.0781934847058823
H	-0.97589663588235265	-1.6483160401176469	-1.4957388847058823
H	0.68109590411764742	-1.7635482401176468	-1.9303626847058823
H	-0.38751639588235265	-3.6850429401176465	-0.18806423470588235
H	1.5355505041176474	-1.6715843401176467	0.27229328429411764
H	0.15338130411764728	-1.8136543401176470	1.9156812152941176
H	2.4835513041176474	1.6271992098823529	1.9943924152941177

C	0.68808176647058827	0.26778768305882350	-0.35924200294117636
C	0.85447216647058832	1.0691689030588234	0.88642429705882364
C	-0.22182723352941180	1.8604571330588233	1.0914727270588236
C	-1.1126394535294117	1.6034683830588234	-2.7654432941176366E-002
O	-0.66154970352941167	0.70809242305882347	-0.89878527294117638
O	1.9275570664705883	0.91128959105882346	1.6181005270588238
O	-0.36379933352941163	2.7419064930588233	2.1138792270588236
O	-2.1862889735294120	2.2052034930588236	-0.28115007294117644
C	0.60724006647058815	-1.2335576069411767	3.7682147058823612E-002
C	0.22209886647058830	-2.0409936069411767	-1.1708639729411763
O	0.10611796647058824	-3.4334791069411765	-0.82843927294117636
O	-0.36279573352941163	-1.4909679069411768	1.0324470870588236
H	-0.56057918352941172	3.5966895930588234	1.7367643270588236
H	1.3580527664705881	0.50056135305882354	-1.1772750729411765
H	-0.70623377352941175	-1.6964765069411767	-1.6896010729411763
H	1.1048380664705881	-2.0701888069411765	-1.8746393729411763
H	-0.69274534352941175	-3.4989615069411770	-0.20911979294117644
H	1.5884562664705881	-1.5542253069411767	0.27443729705882358
H	7.7066647058821047E-004	-1.4694730069411768	1.9175045270588238
H	1.8457575664705881	1.3900319130588235	2.4806936270588236

C	0.70204893588235273	0.24632857764705895	-0.37271246411764697
C	0.95728383588235277	1.1133807576470589	0.85144319588235295
C	-0.16911486411764720	1.9093223376470589	1.0905244158823530
C	-1.0914313941176472	1.6246860376470589	4.0701958823529671E-003
O	-0.53749470411764722	0.72661389764705897	-0.86853513411764705
O	2.0457542358823524	0.85841704764705895	1.6176503658823531
O	-0.55408721411764728	2.7321389376470591	2.0986843658823529
O	-2.1593351341176472	2.2040640376470590	-0.18197278411764700
C	0.61370333588235293	-1.2470244623529412	7.1493525882352971E-002
C	0.16965243588235279	-2.1107267623529413	-1.1715063341176470
O	-0.13552576411764727	-3.4979871623529410	-0.91232673411764686
O	-0.38333256411764727	-1.3265257623529412	1.0617640558823529
H	-0.45321227411764731	3.6533163376470590	1.7479515658823530
H	1.5173606358823528	0.53462986764705900	-1.0480943341176470
H	-0.60785192411764732	-1.6101675623529410	-1.6989720341176471
H	1.0566751358823525	-2.1478148623529414	-1.8299423341176471
H	-0.97109271411764730	-3.6626512623529406	-0.45951953411764707
H	1.6207174358823526	-1.5582602623529411	0.46823733688235297
H	0.19841003588235262	-1.1670752623529410	1.8803864658823530
H	1.9933319358823527	1.5497255676470589	2.3544631658823527

C	0.69211851352941145	0.19525915905882341	-0.34398641941176461
C	0.91317581352941146	0.99899400005882344	0.93252867058823541
C	-8.0069586470588261E-002	1.9312893390588235	0.97759754058823534
C	-1.0304060264705883	1.7129071190588234	-0.11912263941176465
O	-0.52185701647058835	0.77556227905882347	-0.94106114941176466
O	2.0022262135294113	0.84548336405882341	1.7127485505882354
O	-0.31499728647058833	2.8291161390588235	2.0264505505882355
O	-2.1435445064705885	2.2296427390588232	-0.31621535941176465
C	0.48910321352941155	-1.2969416609411764	2.5599210588235333E-002
C	0.11713221352941172	-2.0802032609411767	-1.1748448494117647
O	8.0790213529411625E-002	-3.4349667609411765	-0.70252724941176470
O	-0.66789943647058836	-1.4006800609411765	0.88612192058823536
H	-0.35042868647058834	3.6899703390588234	1.5955242505882352
H	1.5275785135294118	0.20786654905882340	-0.97988464941176479
H	-0.85721005647058834	-1.7727246609411766	-1.5007998494117647
H	0.84552791352941159	-1.9328374609411765	-1.9259525494117649
H	-0.70124000647058837	-3.4977371609411763	-0.15217597941176469
H	1.4378253135294117	-1.7164936609411767	0.42489180158823536
H	-0.43520848647058830	-1.4988549609411765	1.8442074505882353
H	2.0131878135294112	1.5181125590588236	2.3969432505882353

C	0.75771953176470586	0.30807884017647053	-0.31749798999999995
C	0.94428523176470591	1.0722923301764706	0.92678093000000006
C	-0.13861836823529416	1.8461225901764706	1.1862050300000000
C	-1.1216267482352942	1.7065326901764706	9.4258750000000002E-002
O	-0.45142656823529426	0.91078311017647051	-0.91682617999999994
O	2.0127453176470560	0.77882209717647055	1.6945691199999999
O	-0.40060306823529412	2.5016360901764703	2.4054263200000001
O	-2.2349753282352944	2.1675385901764708	-7.4760129999999980E-002
C	0.54640853176470561	-1.2347557098235296	-0.11911816000000003
C	0.18182563176470579	-1.9972363098235295	-1.3929663800000001
O	-4.0629568235294178E-002	-3.3421158098235293	-0.99873627999999992
O	-0.50188261823529412	-1.4305782098235293	0.88221151000000009
H	-0.54034905823529422	3.4461948901764705	2.2143704200000003
H	1.5855896317647060	0.48392574017647055	-0.98118418000000007
H	-0.70652209823529422	-1.7236476098235296	-1.9038428800000002
H	1.0063641317647058	-2.0703508098235295	-2.1259017800000000
H	-0.89830469823529413	-3.4232425098235297	-0.57298811999999999
H	1.5242713317647056	-1.6442605098235297	0.27326872000000002
H	-0.13676516823529417	-1.6935969098235293	1.7464623200000000
H	1.9700265317647059	1.3060249301764706	2.5561943200000004

C	0.71107993705882322	0.35364228529411762	-0.30522232058823529
C	0.93273153705882317	1.0444456952941177	0.99029320941176469
C	-9.8295862941176759E-002	1.8229091052941175	1.3120034794117648
C	-1.0860467329411767	1.6134316152941177	0.19694073941176474
O	-0.52837109294117679	0.90211922529411759	-0.78392596058823527
O	1.9811712370588230	0.61214725529411762	1.6882459394117646
O	-0.19160236294117672	2.6335801052941177	2.4144409394117647
O	-2.2658603529411767	2.0136146052941175	3.5591589411764712E-002
C	0.55126103705882312	-1.1492478947058824	-0.17449229058823523

C	0.22910843705882322	-1.9390188947058824	-1.4316409605882354
O	0.19647223705882322	-3.3142047947058826	-1.1323510605882352
O	-0.59694257294117681	-1.5158969947058825	0.73445629941176471
H	-0.81142341294117681	3.3960735052941180	2.2003178394117646
H	1.4824154370588234	0.35879906529411759	-1.1496834605882351
H	-0.79152465294117680	-1.5703021947058822	-1.7350430605882354
H	0.92766373705882321	-1.7435411947058823	-2.1430600605882355
H	-0.64183655294117681	-3.5185504947058819	-0.71687086058823535
H	1.3912464370588231	-1.5195418947058823	0.36218569161176473
H	-0.36886636294117681	-1.3322966947058823	1.6575991394117646
H	2.1416496370588232	1.1574881852941177	2.4678488394117646

C	0.76180017294117630	0.27327968276470588	-0.29610041000000004
C	0.88938107294117663	1.1125044427647057	0.90684269999999989
C	-0.18737972705882355	1.8653223727647059	1.1169403800000000
C	-1.1034755770588234	1.5709328327647059	-2.1467090000000022E-002
O	-0.46333072705882339	0.73121043576470590	-0.91374387000000001
O	1.9314829729411767	0.96184387276470584	1.6288431299999999
O	-0.56153770705882344	2.6191349727647060	2.2354781300000002
O	-2.2451503770588235	1.9569434727647059	-0.25998395000000002
C	0.62835767294117661	-1.2658214272352941	3.4977060000000004E-002
C	0.24724907294117648	-1.9468002272352940	-1.2786618700000001
O	-2.1116327058823492E-002	-3.3758369272352944	-0.97447687000000016
O	-0.45986702705882343	-1.4577595272352941	0.96725843999999994
H	-0.67669393705882352	3.5478658727647057	2.0540696299999999
H	1.4930660729411767	0.40574843276470585	-1.0782998700000002
H	-0.59073060705882352	-1.4951299272352940	-1.8148307699999999
H	1.1494138729411765	-2.1132351272352938	-1.8906895700000002
H	-0.79146889705882351	-3.3902032272352942	-0.41615520000000000
H	1.5349094729411765	-1.6832212272352940	0.35717056599999997
H	-0.12580572705882354	-1.8154328272352942	1.7848148300000000
H	1.9618218729411765	1.5708400427647060	2.3796279299999998

C	0.70630895411764683	0.20422445794117650	-0.14007043588235296
C	0.71621285411764712	0.96373718794117647	1.1332025941176471
C	-0.32781024588235308	1.8648142779411765	1.1854391841176470
C	-1.1219898658823531	1.6816514779411764	-2.8714415882352962E-002
O	-0.49233012588235303	0.74982512994117645	-0.78304057588235287
O	1.8115142541176470	0.68091434094117642	1.8525092241176471
O	-0.56978803588235305	2.6959483779411766	2.2284676241176471
O	-2.1724444058823531	2.1907157779411763	-0.33020071588235295
C	0.52657475411764687	-1.2776795220588237	-3.4888725882352922E-002
C	0.38508495411764687	-1.9593276220588236	-1.3597121758823529
O	0.30251935411764697	-3.3875675220588239	-1.1268508758823528
O	-0.68614725588235304	-1.4924626220588237	0.64877487411764712
H	-0.98417809588235305	3.5176996779411764	1.9305961241176470
H	1.6187452541176468	0.44319884794117648	-0.71583917588235280
H	-0.48421015588235305	-1.6147635220588237	-1.9251978758823527
H	1.2547379541176469	-1.6827314220588234	-1.9414906758823527
H	-0.48280014588235309	-3.5781973220588239	-0.59298397588235296
H	1.3290339541176468	-1.6138710220588237	0.55760652411764711
H	-0.69732384588235308	-1.6368500220588236	1.5724349241176472
H	1.9032746541176471	1.2347238079411764	2.6972741241176470

C	0.62771357647058834	0.30914444045294109	-0.32894338647058829
C	0.85388217647058817	1.0515728804529412	0.92560741352941167
C	-0.21216742352941154	1.8310261904529410	1.1815361235294117
C	-1.1861322435294117	1.6260622404529412	6.4648553529411679E-002
O	-0.63407754352941159	0.81380718575294109	-0.81566037647058831
O	1.9450237764705884	0.89972707745294112	1.6068898235294118
O	-0.33477379352941161	2.6147330904529409	2.3114781235294117
O	-2.2217474535294115	2.2678432904529413	-0.18789344647058825
C	0.41932277647058847	-1.2077965095470589	3.9545083529411718E-002
C	0.37350967647058830	-2.0210291095470589	-1.2378680764705883
O	0.39390807647058845	-3.4352180095470586	-0.90081607647058837
O	-0.90569802352941164	-1.3655538095470590	0.57917098352941165
H	-0.63016612352941159	3.5200611904529411	2.1430468235294118
H	1.3277673764705884	0.44305198045294109	-1.1292426764705883
H	-0.50084299352941164	-1.6986827095470587	-1.7164846764705881
H	1.2039324764705885	-1.9290628095470588	-1.9337164764705881
H	-0.51945431352941163	-3.7196866095470589	-0.60129773647058826
H	1.2315446764705882	-1.5318819095470588	0.75947629352941171
H	-0.78784122352941166	-1.7771076095470586	1.4530437235294118
H	1.9832730764705884	1.5648926504529412	2.3118716235294117

C	0.75576642647058834	0.18449909371764694	-0.23075825352941182
C	0.94405992647058867	1.0813843537176471	0.95129796647058829
C	-0.16885907352941154	1.8919221237176469	1.0846943564705882
C	-1.0550598535294116	1.6732745737176469	-1.7638813529411768E-002
O	-0.38567628352941152	0.78206330051764694	-0.87528798352941184
O	2.0339737264705882	0.97259239371764694	1.7277833164705882
O	-0.31205188352941149	2.7303977237176471	2.1429841164705881
O	-2.1290346135294116	2.2094168237176470	-0.31097068352941182
C	0.39196402647058837	-1.2688306762823531	0.10620282647058824
C	0.16377872647058855	-2.0259607762823530	-1.1957239835294118
O	0.26524922647058857	-3.4288140762823534	-1.0305218835294117
O	-0.74826515352941148	-1.2755662762823530	0.89713898647058821
H	-0.92551044352941148	3.4734227237176469	1.9685560164705882
H	1.6499582264705885	0.33649972371764697	-0.93361868352941180
H	-0.79234039352941155	-1.8682850762823531	-1.6032790835294117
H	0.87559692647058829	-1.7050397762823533	-2.0007782835294119
H	-0.56354951352941152	-3.7629761762823528	-0.68007993352941176
H	1.2226500264705884	-1.6898737862823533	0.71732482647058826
H	-0.52097540352941152	-1.3997881762823532	1.8605625164705883
H	2.1059144264705889	1.6774008437176469	2.4340520164705879

C	0.67340563294117639	0.20107799676470595	-0.37930877941176466
C	0.96361293294117667	1.0853320267647060	0.86102295058823530
C	-9.9726067058823586E-002	1.9614635767647060	0.96821774058823529
C	-1.0660851570588235	1.7201691667647059	-6.6666949411764709E-002
O	-0.56348402705882350	0.74119449176470598	-0.87669957941176468
O	2.0065549329411763	1.1413387067647061	1.6442652205882353
O	-0.36805238705882359	2.7408943767647056	2.0579795205882352
O	-2.1652698570588234	2.2176815767647060	-0.29528675941176474
C	0.45910723294117650	-1.2249198232352940	9.1499530588235289E-002
C	0.14508953294117655	-2.0426026232352941	-1.0633088794117647
O	0.42009883294117656	-3.4801640232352944	-0.73519217941176462
O	-0.51588188705882354	-1.3653909232352941	1.1007550605882352
H	-0.75063019705882350	3.6143754767647058	1.7938079205882353
H	1.4495593329411767	0.38942226676470593	-1.1539019794117646
H	-0.86821788705882352	-1.9943970232352939	-1.4475542794117646
H	0.70483513294117639	-1.8343997232352942	-2.0445266794117649
H	-0.42491609705882349	-3.8710755232352940	-0.45510187941176472
H	1.4301362329411766	-1.5888877232352940	0.49348372058823531
H	-0.16631726705882355	-1.5976958232352940	1.9417849205882354
H	1.8667341329411764	1.8062123567647059	2.3392194205882353

C	0.60641673176470601	0.21532461447058826	-0.28524445829411760
C	0.77803753176470614	1.0914709244705882	0.95942816170588230
C	-0.24559836823529402	1.9763669944705882	1.0471711817058824
C	-1.1132922882352940	1.7201642044705883	-6.0269448294117650E-002
O	-0.62191073823529408	0.75259858947058833	-0.87083739829411766
O	1.7951357317647061	0.72695102347058826	1.7629782017058824
O	-0.42309162823529400	2.8112363944705883	2.1209345017058823
O	-2.1650134382352939	2.2318929944705883	-0.44919002829411769
C	0.45791023176470591	-1.2301113055294119	5.3303962705882363E-002
C	0.32852403176470601	-2.0873335055294118	-1.2119592982941176
O	0.33355283176470607	-3.5037370055294121	-0.91270419829411764
O	-0.71370779823529407	-1.4741186055294118	0.88398201170588231
H	-0.58735233823529398	3.7265225944705880	1.9022588017058824
H	1.3980830317647059	0.30890100447058833	-1.0533527982941175
H	-0.62260535823529406	-1.7298635055294116	-1.6302728982941175
H	1.2286709317647058	-1.9000654055294119	-1.9034125982941175
H	-0.43375909823529402	-3.6362000055294117	-0.35281369829411768
H	1.4281993317647059	-1.5842545055294118	0.49718829170588241
H	-0.47639773823529408	-1.3646035055294119	1.8187393017058824
H	1.6539919317647060	1.3718535344705884	2.5304225017058823

C	0.71446202176470552	0.24511387411764712	-0.23409230588235297
C	0.87780812176470557	1.0321923941176472	1.0160607141176472
C	-0.27829697823529442	1.7201658641176472	1.0771422741176471
C	-1.0351872082352944	1.6797529341176471	-0.11680559588235295
O	-0.37363897823529446	0.86444693411764706	-0.95890356588235293
O	1.8326251217647054	0.79603671411764709	1.9163887341176471
O	-0.66921959823529442	2.5814613641176472	2.1365206341176473
O	-2.0454848582352945	2.2855687641176470	-0.45982334588235296
C	0.42389082176470549	-1.2110083358823529	0.12378446411764704
C	0.31497982176470551	-2.0093537358823528	-1.1707228658823530
O	0.19208462176470564	-3.4280763358823525	-1.0000507658823530
O	-0.87164411823529442	-1.2178432358823530	0.73633615411764697
H	-0.72112385823529446	3.4869989641176473	1.8255333341176470
H	1.6417870217647057	0.29830154411764714	-0.79656486588235287

H	-0.51021416823529442	-1.6518286358823531	-1.7331991658823531
H	1.0980545217647057	-1.8042723358823527	-1.8844786658823529
H	-0.59088230823529442	-3.6676567358823529	-0.47712516588235299
H	1.1539068217647055	-1.5324343358823529	0.85468325411764701
H	-0.69439507823529445	-1.3810380358823529	1.6423861341176469
H	1.9080144217647057	1.5153644041176473	2.5736070341176474

C	0.81547340117647060	0.22570716529411772	-8.2681861058823580E-002
C	0.74512950117647048	1.0256116452941177	1.1867287389411765
C	-0.32498389882352963	1.8395288052941177	1.1588793389411765
C	-0.99848641882352951	1.6092594352941179	-0.10081307105882359
O	-0.21713809882352963	0.82432019529411771	-0.91837176105882357
O	1.7174807011764703	0.85391117529411775	2.0518063389411765
O	-0.81094041882352952	2.7608714052941177	2.0319512389411765
O	-2.0383787488235297	2.0673215052941178	-0.51703774105882361
C	0.29838060117647047	-1.2519705947058823	0.13247899694117640
C	0.40874650117647038	-2.0432128947058823	-1.2379101610588237
O	0.16406750117647051	-3.4730138947058826	-1.0535563610588237
O	-1.0059776888235294	-1.1695765947058823	0.63468937894117639
H	-0.71948486882352958	3.6525706052941178	1.7073077389411764
H	1.7924923011764706	0.23859452529411773	-0.60062071105882364
H	-0.37543019882352957	-1.7237213947058820	-1.9111320610588236
H	1.3556350011764704	-1.9301042947058824	-1.7272899610588237
H	-0.80658516882352960	-3.5060967947058819	-0.75442808105882364
H	1.0881988011764703	-1.6869894947058821	0.80917705894117642
H	-0.92850501882352954	-1.2253575947058823	1.6331510389411763
H	1.6801242011764703	1.4437069752941176	2.8796106389411764

C	0.76206767823529398	0.26027593576470587	-6.7277379999999970E-002
C	0.63040997823529410	1.0407799057647058	1.1960798900000000
C	-0.36461332176470584	1.9397219657647060	1.1128153400000000
C	-0.99073856176470587	1.6728996657647057	-0.20649471999999999
O	-0.22594532176470583	0.77145878576470583	-0.90174770999999998
O	1.3076561782352940	0.65304471576470591	2.2475874899999999
O	-0.94457253176470590	2.7551707657647060	2.1026339899999997
O	-2.0807773417647057	2.0396447657647059	-0.67305358999999998
C	0.52306737823529414	-1.2686676342352943	-2.9214999999996327E-004
C	0.51629487823529430	-2.0396584342352941	-1.3122952100000000
O	8.4585178235293990E-002	-3.3944085342352941	-1.0328483099999999
O	-0.79189358176470592	-1.5036838342352938	0.53524667000000004
H	-0.71775012176470598	3.6935495657647062	2.0007494900000000
H	1.7330496782352942	0.45876326776470588	-0.58085834999999997
H	-0.19413102176470587	-1.6891423342352940	-2.1088771099999999
H	1.4755953782352942	-2.0724407342352942	-1.8116327100000000
H	-0.72230452176470594	-3.3173078342352942	-0.49973562999999999
H	1.3080767782352940	-1.8123934342352941	0.71536434000000004
H	-0.63281862176470582	-1.4942361342352939	1.4843352900000000
H	1.0937731782352940	1.2033709057647060	3.0196752899999999

C	0.73491906647058780	0.19123564823529410	-0.10987073888235289
C	0.65869796647058809	1.0125151482352941	1.1590612511176470
C	-0.30969703352941202	1.9750490282352942	1.0717238011176471
C	-0.90159413352941198	1.8525923282352941	-0.26182787888235287
O	-0.20387503352941194	0.92017529823529409	-0.99923854888235297
O	1.5587930664705878	0.67944492823529412	2.0768054511176470
O	-0.79022074352941196	2.6407926282352943	2.1796513511176472
O	-1.9154970235294120	2.3471156282352941	-0.69674341888235292
C	0.38326166647058812	-1.2972047717647059	6.2211422117647096E-002
C	0.29889796647058797	-2.0005158717647058	-1.2744839488823529
O	0.25336526647058788	-3.4586193717647058	-1.1439154488823529
O	-0.91850653352941203	-1.4516179717647060	0.60597447111764713
H	-0.84954336352941207	3.6370359282352944	1.9752530511176472
H	1.8246709664705878	0.29946223823529411	-0.45781107888235290
H	-0.61831083352941207	-1.6528140717647057	-1.7815814488823529
H	1.2129402664705879	-1.9187232717647060	-1.8866397488823530
H	-0.41830153352941202	-3.7759234717647061	-0.51856853888235299
H	1.1838563664705881	-1.6429622717647059	0.77782553111764707
H	-0.94532629352941200	-1.7517301717647060	1.5271103511176471
H	1.5174945664705879	1.2635587982352940	2.8599117511176471

C	0.81839782058823496	0.18927087823529412	-0.13283102094117655
C	0.77318432058823494	1.0050535682352941	1.1722019570588234
C	-0.30356437941176506	1.7775486682352941	1.1547396570588233
C	-0.93803113941176519	1.6470525682352943	-0.18052945294117656
O	-0.18690437941176508	0.80604237823529412	-0.91663281294117649
O	1.6677503205882347	0.82304947823529417	2.1299601570588234
O	-0.84257012941176512	2.5203743682352941	2.1556227570588233
O	-1.9755853294117651	2.0780005682352942	-0.63625806294117648
C	0.33158192058823466	-1.2730566317647058	5.9011155058823442E-002
C	0.31801202058823486	-1.9296227317647059	-1.3130084429411766
O	0.16222412058823488	-3.3618452317647058	-1.0562328429411767
O	-0.94642710941176511	-1.2315506317647058	0.65398804705882352
H	-0.80619673941176517	3.5116459682352943	2.0479716570588233
H	1.7229494205882350	0.26308107823529414	-0.77802931294117661
H	-0.42034537941176509	-1.5421091317647058	-1.9572872429411765
H	1.3018639205882347	-1.8490873317647056	-1.8045083429411766
H	-0.67633927941176508	-3.4338479317647059	-0.59817785294117654
H	1.0522560205882348	-1.8241209317647056	0.66383018705882346
H	-0.91349817941176514	-1.6426560317647056	1.4926679570588235
H	1.2832751205882349	1.1527072282352941	2.9938811570588233

C	0.83218531105882354	0.20420707529411763	-0.18334541529411758
C	0.79945851105882326	1.0071253052941176	1.0678461147058824
C	-0.14949388894117654	1.9740499252941175	1.0034491147058824
C	-0.82916925894117655	1.7267255252941176	-0.28539315529411757
O	-0.20578318894117653	0.78632302529411757	-0.99406671529411761
O	1.6344035110588235	0.52854401529411765	2.0083738147058825
O	-0.63583028894117644	2.7056112252941178	2.0216542147058827
O	-1.8484751369411765	2.2635621252941176	-0.69408556529411758
C	0.46278951105882360	-1.2601052747058823	0.16739116470588244
C	0.26026281105882365	-2.0324015747058821	-1.1289835852941177
O	-0.15910598894117656	-3.4101965747058820	-0.98023742529411761
O	-0.79810954894117658	-1.3738446747058823	0.81462747470588237
H	-0.80008347894117660	3.6400398252941177	1.7444903147058823
H	1.7335107110588233	0.20303087529411767	-0.80417095529411764
H	-0.52209718894117652	-1.4739437747058823	-1.6331004852941176
H	1.1113018110588233	-2.0629697747058824	-1.7574776852941176
H	-0.88576420894117658	-3.4257572747058820	-0.36697122529411752
H	1.2286597110588233	-1.6660776747058825	0.79435436470588239
H	-0.74772818894117665	-1.4094806747058823	1.7887609147058825
H	1.6181659110588233	1.1209785552941176	2.7793237147058827

C	0.88010155411764690	0.21851636882352943	-0.18695021894117647
C	0.84866765411764722	1.0497939788235295	1.0186975790588235
C	-0.19238504588235283	1.8853222388235293	0.99543737905882346
C	-0.88171301588235285	1.7158138388235293	-0.26387123094117648
O	-0.22176794588235293	0.74848593882352943	-0.98823204094117645
O	1.7951355541176472	0.77718548882352945	1.8940956790588235
O	-0.60452608588235290	2.5610527388235296	2.1553415790588235
O	-1.9437921258823530	2.2067364388235298	-0.71867721094117643
C	0.54425805411764716	-1.2966704611764706	8.0332049058823518E-002
C	0.19944715411764702	-1.9727583611764705	-1.2354954209411766
O	-0.20676404588235298	-3.3651003611764709	-0.97574835094117640
O	-0.71225900588235291	-1.2361118611764705	0.86561619905882359
H	-0.76408394588235284	3.5072790388235298	1.9642558790588236
H	1.8082555541176470	0.24179305882352942	-0.77747638094117644
H	-0.66814008588235285	-1.4381717611764706	-1.6584942209411766
H	0.98844095411764710	-2.1690595611764705	-1.9537390209411765
H	-0.86887517588235286	-3.4341067611764702	-0.21509224694117648
H	1.3431325541176473	-1.7489842611764705	0.65633720905882353
H	-0.49537024588235279	-1.4268704611764707	1.7659487790588235
H	1.5988963541176473	1.2792298388235295	2.7104786790588236

C	0.85184136235294106	0.16587544000000004	-0.17159043958823533
C	0.84060656235294107	1.0312783400000001	1.0599507554117646
C	-0.23428053764705892	1.9142977999999999	1.0147775554117646
C	-0.92572296764705886	1.6137785999999998	-0.20630495158823534
O	-0.16159633764705883	0.85432294000000009	-1.0185374745882352
O	1.6768437623529413	0.81127082000000006	2.0617363554117647
O	-0.66863958764705889	2.6979167999999998	2.0622025554117647
O	-2.0107317676470591	2.0413127000000002	-0.57008587458823534
C	0.42582406235294123	-1.3189560999999999	3.7128145411764679E-002
C	0.18100326235294117	-1.9875642000000000	-1.2714820445882353
O	-2.7159437647058970E-002	-3.3685439000000001	-0.99447909458823536
O	-0.77980931764705896	-1.4444400000000002	0.70418483541176469
H	-0.45307823764705901	3.6766030000000001	1.9900537554117648
H	1.8053298623529412	0.24376616000000007	-0.70140203458823536
H	-0.77284634764705895	-1.5202298000000001	-1.5879071445882353
H	0.95416826235294105	-1.9301317000000000	-2.0604753445882356
H	-0.70175259764705888	-3.4805568999999998	-0.34776955458823533
H	1.3381076623529411	-1.7536705000000001	0.51072559541176465
H	-0.77655170764705894	-1.3939246999999999	1.6568786554117647

H	1.8731587623529411	1.6673610000000001	2.4624722554117646

C	0.81663682588235309	0.21622409705882351	-0.18968334488235292
C	0.81986912588235317	1.0651886670588235	1.0676232421176473
C	-0.20715477411764693	1.9593953970588234	1.0908450421176472
C	-0.98690633411764683	1.6363023970588233	-0.13884476688235292
O	-0.31707177411764675	0.76278941705882353	-0.90488181788235289
O	1.7091354258823532	0.66584921705882349	1.9897872421176472
O	-0.66211777411764683	2.6944160970588236	2.1679088421176469
O	-2.1791745241176468	2.0391278970588234	-0.34157789788235293
C	0.47462422588235320	-1.2902742029411765	6.7165932117647070E-002
C	0.31093312588235311	-1.9832569029411764	-1.3132184578823529
O	0.18654812588235314	-3.4124148029411763	-1.1992226578823528
O	-0.63186928411764687	-1.4763303029411765	0.86564964211764706
H	-1.0001908441176468	3.6307989970588235	1.9163151421176472
H	1.7112959258823532	0.43134053705882353	-0.72727567788235292
H	-0.57839983411764684	-1.5661489029411766	-1.7480927578823529
H	1.1526652258823531	-1.8067020029411762	-1.9402580578823527
H	-0.61882286411764686	-3.5663056029411764	-0.66223964788235290
H	1.3686386258823533	-1.7387767029411765	0.50205816211764709
H	-0.26571977411764691	-1.4497230029411763	1.7834807421176471
H	1.7359717258823530	1.3528094170588236	2.6600352421176470

C	0.72923252999999999	0.26259782117647068	-0.29029629000000001
C	0.81957833000000013	1.0913669311764707	0.94059550599999997
C	-0.29009187000000014	1.8249019711764705	1.0571510060000000
C	-1.1379716000000000	1.6510973711764705	-0.15135377999999999
O	-0.45183317000000001	0.79176424117647071	-0.95352841399999999
O	1.8875332299999998	1.0331410111764707	1.7813926060000000
O	-0.61901955000000008	2.5061176711764706	2.1760137060000000
O	-2.2024680800000001	2.2107462711764705	-0.46066548399999996
C	0.52105632999999996	-1.2618829288235291	7.3512065999999987E-002
C	0.33396333000000000	-2.0840583288235290	-1.2072718640000000
O	8.0164429999999953E-002	-3.4711449288235294	-0.88541874400000009
O	-0.66992487000000001	-1.3934305288235294	0.80206140599999998
H	-0.46056458000000000	3.4488207711764707	2.1257661060000004
H	1.5766339299999999	0.42989664117647064	-0.97969014400000010
H	-0.50722477000000010	-1.6140355288235293	-1.7682111940000000
H	1.1387583299999999	-1.9348044288235293	-1.9075304940000000
H	-0.74782195000000007	-3.4910940288235293	-0.35252599400000001
H	1.3530594300000001	-1.6330660288235292	0.67879456600000010
H	-0.40943737000000002	-1.5656063288235291	1.7124297060000000
H	1.8727521299999998	1.7239679711764708	2.4828257060000003

C	0.62381187235294111	0.15250483800000009	-0.18777750099999999
C	0.84970377235294126	1.1073443980000000	0.95556895499999994
C	-0.15346682764705877	2.0059412679999999	1.0285031550000001
C	-1.0556854076470588	1.7287805679999999	-0.13038051400000000
O	-0.50704402764705891	0.72247537200000012	-0.86158362499999996
O	1.9130910723529413	0.96275290800000013	1.7259749550000001
O	-0.40719162764705885	2.8309974680000001	2.0781939550000001
O	-2.1309353376470588	2.2378833679999999	-0.41126562499999997
C	0.25920397235294113	-1.3236050320000001	0.16487857500000000
C	0.39987997235294115	-2.0904727319999998	-1.1394143950000000
O	3.4695072352941114E-002	-3.4506896320000000	-0.92220053499999999
O	-1.0078677276470587	-1.5537466319999997	0.73015973500000009
H	-0.70279641764705880	3.6836775680000002	1.7694914550000000
H	1.5057739723529411	0.19778986800000009	-0.86515775499999992
H	-0.23313572764705892	-1.6520963319999997	-1.9660851449999999
H	1.4170533723529413	-2.1019005319999997	-1.4943579450000000
H	-0.80508997764705881	-3.4576367319999997	-0.47454774499999997
H	1.1257013723529410	-1.6441088319999997	0.78003925499999993
H	-1.0252915676470589	-1.1597054320000000	1.5933225550000001
H	2.0640743723529411	1.6812542680000000	2.3774246550000000

C	0.65700115294117656	0.11117992999999993	-0.16983955717647059
C	0.81162565294117672	1.0101239199999998	1.0022392048235294
C	-0.15828754705882342	1.9508363499999999	0.97212130482352932
C	-0.88065757705882330	1.8575681500000001	-0.30825989017647060
O	-0.34844264705882333	0.74831813999999997	-1.0610958851764707
O	1.8531477529411766	0.80141717999999995	1.8066340048235294
O	-0.29543294705882328	2.9567476500000001	1.9205094048235294
O	-1.7648544370588233	2.6184248500000002	-0.72426113517647062
C	0.28532665294117665	-1.3360772500000002	0.19656364482352939
C	0.22127485294117655	-2.2399287499999998	-1.0091711651764705
O	-5.9117147058823427E-002	-3.6033733499999996	-0.81654721517647055
O	-1.0233748770588234	-1.3037320500000003	0.76534830482352945
H	-0.64097313705882331	3.8367674500000000	1.5519847048235293
H	1.5793770529411766	0.14598142999999991	-0.75219347517647062
H	-0.55707184705882329	-1.8583409500000001	-1.5816101951764705
H	1.1272888529411766	-1.9915645500000001	-1.5899564951764706
H	-0.80682980705882335	-3.7043481500000000	-0.20246556017647060
H	0.99948155294117647	-1.8017312500000000	0.91183620482352934
H	-0.97012480705882331	-0.95860655000000017	1.6704793048235294
H	1.7697516529411768	1.4460576599999999	2.5384185048235293

C	0.68103551764705861	3.6330853764705906E-002	-0.16953265787882363
C	0.84892801764705883	1.0195578037647059	0.97987175436117635
C	-7.0114182352941201E-002	2.0104320237647060	0.99878535436117644
C	-0.91376381235294124	1.7906724237647058	-0.16396382653882360
O	-0.42282558235294121	0.76066277976470587	-0.89837278563882372
O	1.8506196176470588	0.83681461376470589	1.8498263543611766
O	-0.25560978235294129	2.9127693237647061	1.9244922543611764
O	-1.8778979723529412	2.4682796237647060	-0.55398791563882366
C	0.24326251764705886	-1.3470956762352939	0.13949111436117639
C	5.0399517647058856E-002	-2.1490776762352941	-1.1204995756388236
O	0.16078811764705869	-3.5665663762352939	-0.83464574563882366
O	-1.0524577723529411	-1.2294173762352942	0.75773547436117639
H	-0.36699558235294116	3.8182138237647059	1.5506682543611765
H	1.5071046176470588	0.18274376376470591	-0.89041737563882362
H	-0.86458957235294120	-1.9578931762352938	-1.6526331456388235
H	0.90694741764705888	-1.8132878762352940	-1.7828212456388235
H	-0.42483108235294131	-3.7731388762352940	-0.13399628663882363
H	0.98853761764705883	-1.8459321762352938	0.78138714436117640
H	-1.0857404623529412	-1.4244994762352938	1.7013584543611764
H	1.8084397176470586	1.5867671837647057	2.4818587543611765

C	0.76309240470588202	0.12959982588235297	-0.27420137076470585
C	1.0013734047058822	1.0451836058823529	0.95481677923529418
C	-1.0478595294117765E-002	2.0031681258823530	0.94540747923529422
C	-0.90996643529411780	1.7742778258823528	-0.16975631776470584
O	-0.35610369529411767	0.80770357588235298	-0.94842395076470576
O	1.8923116047058821	0.86915636588235290	1.8917615792352942
O	-0.39474279529411782	2.8306146258823528	1.9608326792352941
O	-1.9334076452941178	2.3583627258823530	-0.48792679076470585
C	0.24436630470588216	-1.2759843741176471	9.5645979235294151E-002
C	0.12364440470588223	-2.1308869741176473	-1.1277754207647057
O	2.1631047058823238E-003	-3.5368944741176476	-0.78091388076470580
O	-0.96367790529411779	-1.1908323741176470	0.85353158923529415
H	-0.73616898529411778	3.6839599258823528	1.5067527792352942
H	1.5327120047058824	9.0739915882352973E-002	-1.0569383607647058
H	-0.75514503529411781	-1.7983358741176472	-1.6014591207647058
H	0.99482670470588230	-2.0352582741176475	-1.8004630207647059
H	-0.49479884529411777	-3.6245741741176474	3.9109369235294164E-002
H	1.1067750047058822	-1.6540252741176469	0.67208535923529422
H	-0.79780541529411775	-1.5803054741176472	1.7332112792352943
H	1.7723165047058824	1.4752978358823530	2.6489730792352941

C	0.70939623823529385	0.18204727397647064	-0.29639874382352943
C	0.89350393823529406	0.94481824397647063	0.93241447617647055
C	4.3942338235293921E-002	1.9439475339764707	1.0094474761764705
C	-0.83953825176470620	1.9472074339764707	-0.11240803882352943
O	-0.36545916176470616	0.97494661397647064	-0.95467276382352950
O	1.7386173382352939	0.56786515637647061	1.9133109761764706
O	-7.4579561764706259E-002	2.7940888339764705	1.9990147761764707
O	-1.7653252617647062	2.6842855339764711	-0.43175169382352940
C	0.22187123823529387	-1.2617914660235294	2.7529046176470590E-002
C	1.6002938235293751E-002	-2.1812591660235290	-1.2042941238235294
O	7.6707438235293912E-002	-3.5654176660235297	-0.77400453382352952
O	-1.0526101917647062	-1.2458678660235294	0.70788527617647068
H	-0.38233245176470620	3.7585886339764709	1.7770887761764704
H	1.6078153382352938	0.16986800397647067	-0.95009538382352932
H	-1.0263781417647062	-1.9496715660235295	-1.6015041238235295
H	0.69052603823529402	-2.0627729660235294	-2.0407103238235296
H	-0.49215982176470618	-3.7008825660235294	-8.5107382352941907E-004
H	0.96938473823529381	-1.7890057660235292	0.66078624617647064
H	-0.83342898176470614	-1.2546634660235294	1.6690884761764706
H	1.8898093382352938	1.3336840439764708	2.4766307761764708

C	0.68211131294117666	0.20745193176470589	-0.27285967352941187
C	0.83934531294117654	0.97897869176470587	0.97631152647058816
C	-5.6001587058823299E-002	2.0020062317647058	1.0178676264705881

C	-0.94219661705882340	1.8584723317647058	-0.14326228352941187
O	-0.49834402705882341	0.83007991176470586	-0.94323016352941191
O	1.6502472129411767	0.44775100176470584	1.8395110264705881
O	-0.27933368705882344	2.6992001317647061	2.1717759264705880
O	-1.8805600970588234	2.5117671317647057	-0.55164481352941186
C	0.35828601294117668	-1.2798740682352940	2.9876514705881284E-003
C	0.17986791294117666	-2.0922970682352942	-1.2495289735294119
O	0.16854491294117668	-3.4781115682352945	-0.84014729352941186
O	-0.83858766705882337	-1.3903650682352942	0.65200668647058813
H	-0.62860320705882344	3.6163360317647060	2.0665661264705881
H	1.4947677129411765	0.40465308176470582	-0.98381696352941184
H	-0.81854676705882345	-1.8926536682352941	-1.6902908735294118
H	1.0446903129411764	-1.9013016682352941	-1.9242242735294119
H	-0.47568704705882336	-3.5220933682352946	-0.12802125852941187
H	1.2514898129411767	-1.6204923682352941	0.57302360647058814
H	-0.66198223705882342	-1.3320883682352942	1.6280327264705881
H	1.6660701129411766	0.76826594176470586	2.7737560264705881

C	0.73139366117647064	0.24898793652941176	-0.25186637482352936
C	0.74777676117647029	0.97733549652941176	1.0665426651764705
C	-0.22399723882352940	1.9259041565294117	1.1456402651764706
C	-1.0306956188235294	1.7859457565294119	-6.0252019823529353E-002
O	-0.41451579882352951	0.87831680652941180	-0.93336362482352930
O	1.7008595611764705	0.66970517552941178	1.8847653651764706
O	-0.47693364882352951	2.6902058565294120	2.2246374651764707
O	-2.0478133788235295	2.3537445565294117	-0.47570738482352931
C	0.43884086117647048	-1.2511895434705882	-7.1619578235293524E-003
C	0.17827326117647058	-2.0210526434705880	-1.3369305348235294
O	0.14046476117647044	-3.4245424434705880	-1.1065674348235295
O	-0.72919522882352950	-1.5736185434705883	0.73861574517647066
H	-0.50076639882352947	3.6480354565294117	2.1184420651764708
H	1.6604001611764705	0.37092330652941174	-0.86655381482352933
H	-0.79196043882352951	-1.6957280434705879	-1.7667244348235294
H	0.97578946117647059	-1.9591310434705882	-2.0896682348235291
H	-0.35792073882352948	-3.6238422434705879	-0.28384775482352931
H	1.3034081611764705	-1.6505044434705880	0.49380862517647062
H	-0.94952948882352950	-0.70399574347058824	1.1766141651764706
H	1.6839841611764705	1.1506898865294117	2.7526568651764709

C	0.71583817529411764	0.18731775447058829	-0.20948130882352942
C	0.81287007529411781	1.0268354744705883	1.0722834611764707
C	-0.14308492470588230	1.9528180744705881	1.1001609611764707
C	-0.99063477470588235	1.7881171744705884	-0.11748282882352942
O	-0.44601976470588234	0.79825643447058825	-0.90287383882352945
O	1.8074740752941176	0.69862344447058833	1.9175437611764707
O	-0.50376541470588243	2.8382234744705883	2.0205058611764706
O	-2.0834389947058822	2.2546039744705881	-0.43529100882352939
C	0.41776047529411753	-1.2450013255294117	7.2140691176470581E-002
C	0.16991977529411750	-2.0915563255294116	-1.2762545388235293
O	0.24971507529411752	-3.4880071255294118	-0.95435083882352945
O	-0.79975374470588234	-1.4822008255294117	0.75351415117647058
H	-0.43631772470588237	3.8112089744705884	1.8078101611764708
H	1.6306125752941174	0.36212999847058824	-0.77527452882352943
H	-0.79185695470588235	-1.7830724255294115	-1.6983664388235293
H	0.96327447529411758	-1.9165988255294115	-1.9220148388235294
H	-0.57259240470588235	-3.7116979255294114	-0.45256887882352942
H	1.3048369752941178	-1.7081814255294114	0.59460041117647056
H	-0.69666686470588235	-1.3612132255294118	1.6947432611764706
H	1.6925703752941175	1.2767371444705882	2.7407692611764705

C	0.57435845882352932	0.16997893647058837	-0.28235056235294120
C	0.89995585882352946	1.0047289647058884	0.95419494764705881
C	-3.7274741176470672E-002	1.9681892464705881	0.99878698764705875
C	-0.91516230117647068	1.9084764464705883	-0.11514062235294119
O	-0.53683026117647070	0.83923065647058825	-0.90615786235294116
O	1.8332500588235292	0.50576163647058836	1.8428032876470588
O	-0.21701344117647081	2.7241506464705885	2.1110100876470588
O	-1.7703469011764708	2.7389409464705885	-0.36288280235294118
C	0.21096125882352923	-1.2320720535294116	2.6683377647058810E-002
C	0.15170975882352922	-2.1394099535294115	-1.2288181123529411
O	-2.9540241176470694E-002	-3.5248178535294117	-0.87140568235294125
O	-0.95992488117647068	-1.3524344535294115	0.78432104764705879
H	-0.44133131117647073	3.6561504464705883	1.8774319876470589
H	1.4377120588235295	0.24923162647058833	-0.95980121235294125
H	-0.61407308117647075	-1.8383822535294114	-1.9540309123529409
H	1.1533318588235293	-2.1067904535294115	-1.6787669123529412
H	-0.73978215117647073	-3.5709325535294112	-0.23587704235294121
H	1.0377665588235292	-1.6239963535294115	0.58024878764705878
H	-0.95474256117647072	-0.76512555352941158	1.5492198876470589
H	2.0719510588235295	1.1890523964705884	2.4736862876470589

C	0.62359575352941166	0.15040752470588242	-0.24123662458823530
C	0.85814105352941183	1.0123531247058826	0.92028175541176460
C	-7.9987446470588086E-002	2.0390751547058823	0.93629196541176474
C	-0.90603049647058809	1.8623145547058826	-0.23913192458823529
O	-0.34635514647058807	0.82839521470588240	-0.97264809458823542
O	1.7252281535294118	0.67268149470588245	1.8584257054117646
O	-0.32476314647058824	2.8051162547058826	2.0483152054117646
O	-1.8250920364705880	2.4487408547058824	-0.70963284458823539
C	0.21352105352941186	-1.2808361452941175	0.11804148341176469
C	0.14023225352941182	-2.1179402452941174	-1.1424193945882353
O	-1.0641746470588132E-002	-3.4719575452941172	-0.73785567458823520
O	-1.0237844664705882	-1.4017292452941175	0.69808399541176480
H	-0.12087274647058810	3.7893759547058825	1.9048444054117646
H	1.4707462535294118	4.8245284705882407E-002	-0.92214829458823533
H	-0.73184529647058814	-1.7077818452941176	-1.6840710945882353
H	1.0535468535294119	-2.0093896452941173	-1.7309172945882354
H	-0.71564154647058809	-3.6670707452941178	-0.10422327458823530
H	1.0155995535294120	-1.6493654452941175	0.78494648541176471
H	-1.0550686264705882	-0.93056624529411758	1.5890260054117646
H	1.8736488535294118	1.3390960147058824	2.5449587054117648

C	0.51205282294117671	0.15692952705882346	-0.35805659862352945
C	0.87127902294117643	0.89433206705882351	0.88354008137647055
C	8.6710922941176438E-002	1.9512984370588236	1.0026277613764705
C	-0.87117226705882345	1.9761629370588234	-0.20950724862352946
O	-0.53446367705882358	0.86787075705882344	-1.0055288486235294
O	1.9032009229411764	0.49846500705882346	1.6906292513764707
O	4.0704322941176541E-002	2.8673480370588234	2.0277059513764706
O	-1.7017979770588234	2.7877279370588233	-0.49110317862352937
C	0.17423132294117649	-1.2417332629411766	9.3946406976470553E-002
C	-1.2946877058823469E-002	-2.2480243629411767	-1.0970541486235295
O	-0.25876237705882343	-3.5985480629411768	-0.68752389862352936
O	-0.97653607705882350	-1.2156197629411765	0.85522900137647062
H	0.18929852294117655	3.7818028370588235	1.6400577513764707
H	1.4350021229411767	0.18772299705882350	-0.90443534862352948
H	-0.82013888705882354	-1.8307885629411764	-1.7589743486235294
H	0.87885162294117647	-2.2611874629411766	-1.6952130486235295
H	-0.91551346705882353	-3.5737590629411766	1.3660462376470561E-002
H	0.98759052294117655	-1.7010006629411767	0.77895929137647058
H	-0.67930925705882350	-1.3161656629411764	1.7780083513764706
H	1.7538753229411763	0.84614893705882355	2.6221904513764707

C	0.58060869705882334	8.1250289588235214E-002	-0.24643458041176475
C	1.0107621970588236	0.96217815958823527	0.95257967958823531
C	0.15384149705882355	1.9746942495882354	1.0328976495882352
C	-0.81217769294117648	1.9006694495882352	-5.4984330411764717E-002
O	-0.64968131294117648	0.69337113658823524	-0.96668682041176470
O	2.0218703970588234	0.50996325958823530	1.7226483395882353
O	0.25252339705882343	2.9776945495882354	1.9928294395882351
O	-1.7528418529411764	2.6655647495882353	-0.35562821041176473
C	0.24363569705882360	-1.3812296504117647	8.5906336588235280E-002
C	-1.8175102941176524E-002	-2.1911972504117645	-1.1548637604117649
O	-0.41656010294117651	-3.5558806504117650	-0.88846986041176468
O	-0.87002398294117655	-1.3769805504117647	0.92552848958823530
H	-3.0969202941176555E-002	3.8271069495882353	1.6903842395882351
H	1.3133463970588233	0.24902605958823526	-1.0397938604117647
H	-0.71949680294117646	-1.6997326504117645	-1.7896324604117648
H	0.89311799705882366	-2.1307742504117648	-1.8372086604117648
H	-1.1997802229411765	-3.5057238504117643	-0.33908963041176471
H	1.1032519970588237	-1.8522309504117644	0.59931658958823530
H	-0.55403230294117645	-1.6932892504117647	1.8315673395882353
H	2.0774527970588235	1.1202642395882352	2.5010581395882356

5.2 Singly deprotonated (at C3–O^-) molecule of vitamin C with six explicit water molecules

C	-1.5882393694736843	0.27128790199999997	-0.75256523242105278
C	-2.0235995694736841	1.3056893409999999	5.4611377578947351E-002
C	-1.3758164694736843	1.1848836910000000	1.3385340675789472
O	-0.40365892947368415	0.21333694100000000	1.3058986675789472
C	-0.49181517947368419	-0.48918437900000000	8.2055675789473592E-003
O	-2.8463999694736843	2.3180159810000003	-0.25642263242105262
O	-1.5063782694736843	1.8732341809999999	2.3701603675789475
C	0.84789258052631578	-0.43934277899999996	-0.62209392242105266
O	1.4340122305263159	0.89018004100000003	-0.58887223242105269
O	-1.9730077694736843	-0.11607975900000000	-1.8877494324210529
C	1.8430588305263158	-1.4453460190000000	5.8517187578947361E-002
O	3.0528495305263159	-1.4001966189999999	-0.70559398242105265
H	-3.7620426694736842	2.0476758810000000	-0.28657331242105261
H	-0.86723296947368422	-1.5063086189999999	0.19688414657894737
H	2.0636757305263158	-1.0601824190000000	1.0783628675789474
H	1.4510834305263158	-2.4447046190000004	0.11899145757894736
H	3.7484625305263157	-1.6140468189999999	-7.4049612421052624E-002
H	0.81760687052631575	-0.68279941899999996	-1.7197303324210527

H	1.5795494305263158	1.0938874909999998	0.36348498457894735	H	1.7506857784210523	1.0760705991052633	0.22951042063157895
O	-6.5647419473684160E-002	0.61259006100000002	-3.7639774324210524	O	-3.6185101578947498E-002	0.88815313910526317	-3.8821186283684210
H	-0.83093586947368414	0.45623871599999999	-3.1823324324210525	H	-0.79725555157894745	0.55349721910526317	-3.3396049283684208
H	-0.28594084947368414	-2.2755459000000033E-002	-4.4140095324210522	H	-0.47906912157894743	1.2074893891052632	-4.6905247283684215
O	-3.3052330694736840	1.7179183810000000	-3.6459271324210523	O	-3.1134448215789474	2.3045282391052631	-3.4949044283684207
H	-2.7138774694736840	1.8205913810000001	-4.3860680324210524	H	-2.3612395215789475	2.4321154391052628	-4.0793173283684210
H	-2.8867261694736843	1.0992776810000000	-3.0574569324210525	H	-2.7141766215789476	1.6858333391052631	-2.8767153283684208
O	-3.3021624694736844	-2.3528033190000004	-2.6025246324210523	O	-2.8980875215789474	-0.87840616089473689	-4.8472656283684215
H	-2.4628865694736843	-2.8622733189999998	-2.5715872324210527	H	-2.2359244215789476	-1.3530539608947370	-5.4272730283684210
H	-3.2248409694736844	-1.8090974190000002	-1.7669752324210528	H	-2.5104162215789474	-1.0366468608947368	-3.9578143283684208
O	-5.2144160694736845	-0.14383307899999997	-2.8607387324210523	O	-3.3767548215789476	-2.4130548608947366	-2.2057222283684208
H	-4.4872733694736846	-0.67363751899999991	-2.5451011324210526	H	-2.3886949215789475	-2.4960211608947365	-2.2186125283684208
H	-4.7200670694736839	0.62370832100000007	-3.1778546324210524	H	-3.5335591215789472	-1.9131013608947367	-1.4193676283684211
O	-3.2503034694736841	-1.1475277189999999	-5.1858763324210528	O	-5.4035376215789475	-0.38389354089473682	-2.8339507283684209
H	-2.8786591694736843	-1.9128190189999998	-5.7119811324210525	H	-4.9856940215789471	-1.2363723608947368	-2.8084128283684211
H	-3.1775985694736844	-1.5158619190000000	-4.2711359324210525	H	-4.7458710215789477	0.17520612910526312	-2.4191068283684207
O	-4.0606001694736840	-1.2928664190000001	0.26751021057894736	O	0.56201528842105253	3.4318791391052628	-0.80281942836842113
H	-3.6205723694736842	-1.0534107189999999	1.1166562275789473	H	-0.18636535157894749	3.4631498391052631	-0.18307627836842105
H	-4.8655108694736846	-0.69455791899999997	0.37944598757894737	H	0.54016232842105250	2.5247254391052634	-1.1658115283684212

C	-1.4799685442105264	0.43210166157894742	-0.77608944636842103	C	-1.5084025457894736	0.23665101684210532	-0.83715626315789471
C	-1.9504537442105265	1.4427232515789474	2.8573063631578965E-002	C	-1.9091598457894736	1.3029343818421053	-8.5414631578947242E-003
C	-1.3225811442105264	1.2946877415789475	1.3221870636315789	C	-1.3168107457894738	1.1782690618421052	1.2883004968421052
O	-0.44782495421052626	0.22808875157894742	1.3469322636315790	O	-0.55302268578947378	-2.5075938157894662E-002	1.3274685968421054
C	-0.50352365421052625	-0.50630010842105255	-1.8930236368421033E-002	C	-0.57522712578947377	-0.55701267815789479	2.4704096842105255E-002
O	-2.9775734442105262	2.2742172515789472	-0.32502088636842108	O	-2.6394121457894735	2.4243042818421050	-0.28292803315789472
O	-1.4033259442105264	1.9965749515789475	2.3285180636315790	O	-1.3356037457894736	1.8962480818421055	2.2861399968421052
C	0.90506742578947375	-0.46464286842105257	-0.59445560636842099	C	0.81099215421052617	-0.42184467815789467	-0.53018919315789470
O	1.5136658557894735	0.83073958157894734	-0.59963460636842103	O	1.4122440542105263	0.91622507184210522	-0.38728949315789474
O	-1.8945640442105263	0.18150622157894741	-1.9634602363684210	O	-1.7259175457894738	-0.13235444815789471	-2.0219378031578947
C	1.7697263557894736	-1.4651632484210526	0.14565105863157896	C	1.7651092542105262	-1.4209183181578946	0.13621286384210526
O	2.9821657557894734	-1.6804684484210526	-0.63712617636842106	O	2.9269809542105265	-1.6250480181578946	-0.71521497315789473
H	-3.7617328442105262	1.7311245515789475	-0.51723385636842101	H	-3.5796922457894738	2.2555546818421051	-0.40889741315789474
H	-0.77106484421052623	-1.5220324484210526	0.24963928863157897	H	-0.83218995578947375	-1.6490698181578947	6.2843111842105268E-002
H	1.9524989557894736	-1.0063148484210525	1.1285642036315791	H	2.0808154542105264	-1.1140715181578946	1.1385320568421053
H	1.3086498557894737	-2.4578407484210527	0.27393502463157898	H	1.2088403542105264	-2.3997679181578948	0.11183713484210527
H	3.7482401557894738	-1.6761243484210526	-2.8342856368421043E-002	H	3.6614667542105264	-1.4003117181578946	-0.13925037315789476
H	0.92307804578947372	-0.72979034842105261	-1.6652667363684210	H	0.77541985421052617	-0.68721590815789479	-1.5975139031578947
H	1.4095207557894736	1.0969134515789474	0.30156061363157899	H	1.3335697542105263	1.2225043818421053	0.55288055684210524
O	-2.9505076442105262	2.0516012515789472	-3.5988972363684208	O	-0.27591603578947371	0.64037614184210534	-4.1697724031578947
H	-2.4475803442105262	1.8415241515789476	-4.4128194363684212	H	-0.72437305578947375	0.50691181184210532	-3.3251818031578950
H	-2.6229015442105266	1.3459329515789475	-2.9778736363684208	H	-0.14140889578947374	-0.32645098815789469	-4.3123638031578952
O	-9.6763334210526269E-002	1.0599152915789474	-3.9047565363684207	O	-2.8267635457894738	2.1714690818421052	-3.7374899031578948
H	-0.62226368421052625	0.79506068157894738	-3.1678814363684209	H	-2.1693657457894737	2.2484581818421052	-4.5190423031578950
H	-0.54051255421052624	0.54728620157894747	-4.6808048363684209	H	-2.4231400457894736	1.4092379818421055	-3.2747566031578947
O	-3.2665809442105265	-2.2986269484210524	-2.4081356363684208	O	-3.4381503457894738	-2.6467190181578948	-2.0648659031578949
H	-2.4311787442105266	-2.7774975484210525	-2.2442431363684214	H	-2.4497918457894738	-2.6007909181578950	-2.0101022031578948
H	-3.2091963442105262	-1.6716166484210526	-1.6805608363684210	H	-3.7359943457894738	-2.0430007181578946	-1.3087440031578947
O	-2.8171592442105262	-0.80852784842105252	-5.0576674363684209	O	-3.4207698457894735	-0.90688461815789467	-4.4093827031578945
H	-2.1858670442105264	-1.1795204484210526	-5.6942785363684205	H	-2.5987296457894735	-0.85758111815789473	-5.0046332031578951
H	-2.4682690442105262	-1.2044783484210526	-4.2398386363684208	H	-3.1084403457894738	-1.5772979181578946	-3.7174595031578948
O	-5.4111804442105260	-0.39110469842105261	-3.0413609363684211	O	-0.67966949578947378	-3.0659033181578947	-2.0107607031578949
H	-4.5232840442105262	-0.59608206842105260	-3.3231200363684215	H	-0.13588583578947372	-3.8335083181578948	-1.9066946031578949
H	-5.4514088442105262	0.57923843157894739	-3.0695734363684206	H	-0.10317983578947375	-2.5240649181578947	-2.6158327031578947
O	-3.9784482442105262	-1.2583863484210525	5.2731683631578957E-002	O	-5.5758774457894740	-0.18139962815789468	-2.6573840031578948
H	-3.9546785442105263	-0.88891194842105259	0.93495681363157901	H	-5.3078679457894742	-1.0657145181578946	-2.4810636031578950
H	-4.8568964442105260	-0.88847514842105246	-0.27707964636842103	H	-4.7746272457894738	2.0542431842105302E-002	-3.1568539031578950

C	-1.4897295215789477	0.40444012610526314	-0.80915752836842114	C	-1.5213009000000000	0.43173651765263166	-0.72726803289473685
C	-1.8893099215789475	1.3632538491052633	9.1856441631578939E-002	C	-1.8952019000000000	1.4179158293526317	0.16287813210526314
C	-1.2575621215789476	1.2722920291052633	1.3029989716315789	C	-1.2393095000000001	1.2243064193526316	1.3543691071052633
O	-0.46489524157894746	0.10603491910526314	1.3211445716315788	O	-0.46003201999999993	0.14425127935263166	1.3944835071052633
C	-0.54243206157894752	-0.47424243089473683	2.2752321631578948E-002	C	-0.56799555000000002	-0.48893349064736830	3.7850352105263149E-002
O	-2.6981953215789471	2.4385695391052629	-0.24085163836842111	O	-2.4204053999999999	2.6716417993526318	-0.19650236289473685
O	-1.3027016215789475	2.0654564391052634	2.2574279716315790	O	-1.3148797000000001	1.9690392993526316	2.3977484071052633
C	0.87499909842105250	-0.46847885089473684	-0.62067428836842109	C	0.82828595999999999	-0.44225036064736839	-0.59584497289473681
O	1.4738974784210523	0.79897592910526316	-0.67900610836842112	O	1.4300177000000001	0.82514161935263164	-0.41490441289473678
O	-1.7555436215789475	0.22702773910526314	-2.0203388283684207	O	-1.8532150999999999	0.36486067335263167	-1.9553353928947370
C	1.6735344784210524	-1.6125042608947369	5.7131181631578931E-002	C	1.6703707000000001	-1.5439312006473684	-9.8598228947368582E-003
O	3.0560817784210528	-1.5939830608947370	-0.37048683836842106	O	2.9455840000000002	-1.7363864006473684	-0.67820945289473678
H	-3.5913090215789474	2.1369517391052630	-0.31940562836842107	H	-3.2689328000000000	2.4617394993526318	-0.61657589289473680
H	-1.0521121215789475	-1.4029062608947369	8.9995091631578955E-002	H	-0.91175576000000003	-1.5422464006473684	0.17097877210526316
H	1.6732565784210525	-1.3835771608947369	1.0860347516315789	H	1.8203959000000001	-1.3755724006473684	1.1089415371052631
H	1.1718996784210525	-2.5781736608947368	-0.21057078836842108	H	1.0881525000000001	-2.4850008006473683	-0.16871557289473685
H	3.6145954784210526	-1.6454565608947369	0.45404985163157896	H	3.5497000000000001	-2.2325801006473682	-0.14916241289473683
H	0.75484022842105258	-0.72975066089473684	-1.6424099283684210	H	0.64976807000000003	-0.59269560064736848	-1.6707855928947368

H	1.4707538000000000	0.92896381935263173	0.55591410710526312	H	1.5050631115789472	0.59297855494736851	0.89013009157894740
O	-3.0700831999999996	2.1948700993526318	-3.6142854928947368	O	-2.9204007884210528	2.1802361349473682	-3.9126033184210525
H	-2.3404157999999997	2.3003079993526319	-4.1814322928947369	H	-2.2501609884210527	1.8980774349473686	-4.6036989184210526
H	-2.6733407999999996	1.4878807993526315	-3.0313183928947369	H	-2.6587364884210527	1.7821518349473686	-3.0672593184210530
O	-0.10762335999999997	0.57174535935263160	-4.3115870928947375	O	0.38561092157894727	0.69064142494736847	-4.0070224184210526
H	-0.58004979000000001	0.44307164255263165	-3.4007126928947367	H	-0.35170132842105273	0.60746050494736847	-3.3458263184210528
H	-0.74864525999999998	0.21796889935263167	-4.9402189928947369	H	-9.0587358421052755E-002	0.96251499494736836	-4.7909137184210522
O	-3.3914706999999997	-2.4353329006473681	-2.4463291928947366	O	0.69980575157894720	3.2079580349473686	-1.2104510184210526
H	-2.4548880999999998	-2.6398671006473680	-2.1873858928947367	H	-0.10785246842105273	3.0775153349473685	-1.7429601184210526
H	-3.6287984999999998	-1.6543694006473684	-1.8787700928947366	H	0.84889673157894729	2.2760300349473686	-0.95099531842105267
O	-4.2361373000000002	-0.67063980064736839	-0.50019268289473684	O	-2.9872415884210528	-2.7257100650526316	-2.7572438184210530
H	-3.7897423999999997	-0.81500710064736848	0.35020898710526316	H	-2.0948311884210526	-3.0870696650526317	-2.4445519184210527
H	-5.1903033999999995	-0.43226893064736832	-0.28362716289473688	H	-3.3960682884210529	-2.2331717650526315	-1.9652540184210527
O	-5.5705115999999997	0.12671679935263164	-3.1339440928947369	O	-5.4646662884210526	-0.12019143505263158	-3.5711382184210527
H	-5.3992578000000000	-0.31962655064736839	-2.2609901928947367	H	-4.5117625884210524	-0.28498199505263150	-3.5226371184210530
H	-4.9232988999999998	0.86417948935263167	-2.9456082928947369	H	-5.7543454884210528	0.45366117194736844	-2.8174985184210528
O	0.18175818000000002	3.5699128993526319	-1.0089934928947368	O	-3.1610360884210529	-1.1301198650526314	-4.9983843184210528
H	-0.72220525000000002	3.4486057993526318	-0.94813569289473687	H	-2.2730726884210530	-1.2273716650526314	-5.4410205184210527
H	0.41010453000000008	2.6626684993526317	-0.81718942289473684	H	-3.1267190884210527	-1.5524368650526315	-4.1429503184210521

C	-1.5387973889473685	0.47417714273684208	-0.77409115894736846	C	-1.4896711036842107	0.40601756131578959	-0.89670482094736848
C	-1.8344035889473687	1.4384887937368420	0.22774163105263157	C	-1.9602963036842105	1.3778920423157897	4.3625435052631549E-002
C	-1.2346437889473685	1.0940483037368423	1.4317149610526316	C	-1.4290598036842106	1.0716800963157897	1.2778394790526315
O	-0.60922465894736866	-0.14856828626315788	1.3461541610526315	O	-0.82426060368421061	-0.17428206768421045	1.2693374790526315
C	-0.66119481894736865	-0.54831359626315790	-2.1352658947368416E-002	C	-0.63518790368421074	-0.58714701768421040	-6.4933940947368443E-002
O	-2.3648601889473686	2.6976565937368422	-3.4804508947368418E-002	O	-2.4974808036842107	2.5943055823157892	-0.29217391094736844
O	-1.0313770889473686	1.7785292937368422	2.4467883610526315	O	-1.4235994036842108	1.7674542823157897	2.3394602790526315
C	0.74902409105263135	-0.48535462626315795	-0.56197873894736849	C	0.84948607631578943	-0.44918983768421045	-0.52038671094736844
O	1.3546339110526315	0.81863995373684206	-0.31048902894736841	O	1.2444297963157893	0.83735024231578958	-0.10358739094736845
O	-1.8143237889473687	0.34416198373684209	-1.9676880389473683	O	-1.5422415036842108	0.34963356931578959	-2.1220953209473681
C	1.6197270110526314	-1.6486025062631580	-7.0560178947368421E-002	C	1.7715900963157893	-1.5490830176842103	-6.1666350947368456E-002
O	2.9180190110526314	-1.5418676062631578	-0.70769925894736851	O	3.0964718963157893	-1.3606646176842103	-0.55187466094736848
H	-3.2904305889473688	2.5624607937368422	-0.38180432894736843	H	-3.5046043036842103	2.4649577823157895	-0.15323150094736843
H	-1.0322841889473686	-1.5239309062631579	-0.24515091894736840	H	-0.87257370368421072	-1.6366661176842106	-0.12403864094736845
H	1.7939222110526314	-1.5676621062631577	1.0022925510526315	H	1.8695860963157893	-1.4738400176842104	1.0050349390526316
H	1.1963594110526314	-2.6301134062631579	-0.28694324894736839	H	1.3276740963157894	-2.4964826176842108	-0.40777963094736841
H	3.6316142110526313	-1.3926869062631577	-9.7703338947368421E-002	H	3.7200036963157896	-1.6234368176842102	0.16939474105263155
H	0.60272802105263135	-0.57960814626315793	-1.6220903389473684	H	0.89222948631578936	-0.33245591768421040	-1.6236719209473685
H	1.5455122110526314	0.85854523373684211	0.62766408105263150	H	1.4075041963157893	0.81395602231578956	0.81745244905263148
O	-0.12982102894736863	0.46811352873684209	-4.2472447389473684	O	0.40842035631578932	0.81164221231578959	-3.9676639209473685
H	-0.62356323894736865	0.26720293373684212	-3.4167579389473683	H	-0.19234497368421066	0.46010145031578958	-3.3090059209473681
H	-0.74169714894736871	0.21056016373684208	-4.9338156389473689	H	-0.17048220368421063	0.82339437231578960	-4.7816309209473689
O	-2.9954775889473688	2.1537101937368419	-3.7946178389473686	O	-2.8057122036842106	2.1596702823157896	-3.7948360209473684
H	-2.3208064889473690	2.0182289937368423	-4.4312447389473686	H	-2.1330666036842105	1.9722321823157896	-4.4371201209473687
H	-2.6593280889473689	1.5580967937368420	-3.1273718389473686	H	-2.6495577036842106	1.5134153823157896	-3.0562235209473685
O	-3.2757407889473686	-2.7677025062631579	-2.6352959389473685	O	-3.1240807036842106	-2.8001106176842106	-2.8350822209473683
H	-2.3083572889473687	-2.9451639062631578	-2.6005371389473684	H	-2.1884670036842104	-3.0575422176842104	-2.7290762209473685
H	-3.3986415889473687	-1.9757440062631577	-2.0540898389473683	H	-3.3225328036842106	-2.1550528176842105	-2.0789091209473685
O	-4.1868192889473681	-1.1136962062631581	-0.59515990894736848	O	0.87162599631578930	3.2405662823157892	-1.6292141209473685
H	-3.6000896889473686	-0.95238070626315785	0.17422067905263158	H	0.28585903731578932	2.8877779823157894	-2.2600596209473682
H	-5.0147712889473688	-0.65026330626315787	-0.35275792894736840	H	0.95955754631578927	2.5199737823157893	-1.0122096209473685
O	0.12590920105263137	3.2663836937368420	-1.4709377389473686	O	-3.0710602036842105	-1.1388524176842103	-4.9671980209473690
H	-0.80367512894736870	3.3979465937368420	-1.1188857389473685	H	-2.1441367036842105	-0.99716791768421031	-5.1672046209473690
H	0.42805086105263135	2.4258578937368420	-1.0755732389473684	H	-3.0473544036842104	-1.8772261176842104	-4.2863654209473685
O	-0.85958365894736866	-3.4081340062631580	-1.8721670389473684	O	-5.5606125036842107	-0.38387917768421037	-3.5906803209473681
H	-0.27508568894736862	-4.1734223062631575	-1.6096878389473686	H	-4.6549859036842109	-0.33104745768421034	-3.2982903209473684
H	-0.28458626894736860	-2.8146583062631576	-2.4300687389473685	H	-5.9249580036842113	0.54067137231578966	-3.5164537209473683

C	-1.5136985884210525	0.48753322494736845	-0.86238731842105265	C	-1.3824705568421054	0.37519687044210526	-0.92310621352631572
C	-1.8747459884210529	1.4575433349473685	0.13817368157894735	C	-1.8902672568421053	1.3563592266421054	-5.5526663526315800E-002
C	-1.2188917884210526	1.1540477749473685	1.3231468815789473	C	-1.4981383568421054	1.0008510966421054	1.2676801064736842
O	-0.69899710842105278	-9.9021825052631574E-002	1.2788243815789473	O	-0.59781367684210529	-6.3921183357894762E-002	1.2529954064736841
C	-0.66202940842105273	-0.54593878505263160	-0.11302621842105262	C	-0.50686889684210534	-0.48270543335789473	-7.9328893526315780E-002
O	-2.3254934884210527	2.7859525349473686	-7.4403558421052646E-002	O	-2.8688494568421055	2.2877072466421051	-0.35040359352631578
O	-1.0002859884210529	1.7769376349473684	2.4188038815789472	O	-1.8330295568421053	1.5425494466421052	2.3001008064736843
C	0.81232868157894722	-0.47414882505263151	-0.54900969842105263	C	1.0081329731578945	-0.34725401335789474	-0.44688307352631579
O	1.4903154115789472	0.69158346494736844	-9.6870548421052666E-002	O	1.6404942431578946	0.86634564664210523	-0.13179582352631580
O	-1.7761017884210526	0.48024750094736846	-2.0741637184210528	O	-1.5416732568421054	0.18095889664210524	-2.1684035935263157
C	1.5989640115789472	-1.7461650650526317	-5.2015738421052643E-002	C	1.7912541431578946	-1.5150328533578947	0.11084083347368420
O	2.9342083115789470	-1.8030553650526313	-0.59402977842105265	O	3.1321099431578947	-1.6241034533578949	-0.43434939352631580
H	-3.2261453884210529	2.6821121349473684	-0.40028328842105265	H	-3.7941648568421051	2.0225739466421055	-0.16965203352631580
H	-1.0301293884210527	-1.5355745650526316	-0.19817092842105263	H	-0.73927239684210533	-1.5209862533578948	-0.31275033352631582
H	1.7194737115789471	-1.6740177650526316	1.0908133815789474	H	1.8127344431578947	-1.4329321533578947	1.2260302064736841
H	1.0618868315789474	-2.7069172650526316	-0.31652603842105265	H	1.2815784431578947	-2.4909689533578945	-9.8207743526315783E-002
H	3.3330326115789473	-0.98764936505263146	-0.18097564842105265	H	3.7309308431578945	-1.0198203533578947	-5.7559113526315792E-002
H	0.87124624157894726	-0.53644733505263154	-1.6280298184210527	H	0.99499929315789470	-0.40898834335789480	-1.5744684935263158

H	1.2603139431578947	1.2741706166421052	0.64478760647368416	H	1.1490858168421054	1.1987628532631578	0.42785656294736846
O	0.37890925315789464	0.71957173664210528	-3.9876740935263153	O	0.63619276684210535	0.79986363326315790	-4.0114989670526313
H	-0.23115790684210535	0.46585248564210524	-3.2650980935263156	H	-0.16916498315789472	0.45212599026315786	-3.5148291670526315
H	-0.26511741684210532	0.81372412664210525	-4.6816838935263156	H	0.39646718684210530	0.42603544926315789	-4.9137803670526319
O	-2.6999838568421053	2.0166151466421054	-3.9313698935263157	O	-3.2348877831578946	1.6413939532631578	-3.8186233670526315
H	-1.9635059568421054	1.8579747466421053	-4.5368637935263161	H	-2.6976046831578948	1.7411182532631579	-4.7007087670526317
H	-2.4833525568421058	1.3948121466421053	-3.2011806935263158	H	-2.6126752831578948	1.0696374732631577	-3.2918225670526313
O	-2.9939903568421053	-1.5850558533578947	-5.0522680935263162	O	0.33544930684210533	3.2629277532631580	-1.8242052670526314
H	-2.0822835568421052	-1.3652307533578947	-5.2857482935263160	H	0.19002913684210529	2.9546302532631579	-2.7896605670526315
H	-2.7643744568421056	-1.8554999533578946	-4.1218458935263165	H	0.49519910684210533	2.3880101532631581	-1.4065683670526314
O	-3.0168436568421058	-2.7944194533578948	-2.5251246935263154	O	-3.4210768831578946	-2.5837146467368424	-2.5582330670526314
H	-2.1876355568421051	-2.9921520533578949	-2.2393789935263158	H	-2.4858885831578945	-2.6808259467368423	-2.4299148670526316
H	-3.4257507568421053	-2.4056204533578951	-1.6980131935263159	H	-3.5822326831578946	-1.9164065467368421	-1.8775036670526315
O	0.90316011315789468	3.1260378466421050	-1.6689801935263158	O	-0.96558940315789465	-3.2047593467368420	-2.0351737670526314
H	0.59565992315789473	2.5960122466421049	-2.4445655935263155	H	-0.42936429315789471	-4.0340189467368424	-2.1058784670526314
H	1.0533789731578946	2.3631828466421050	-1.0575367935263158	H	-0.56751557315789469	-2.7080556467368417	-2.7787147670526315
O	-0.31984995684210538	-3.2188207533578952	-1.9230382935263159	O	-5.6616051831578948	-0.13052763673684209	-2.7504974670526314
H	0.30886958315789464	-3.9440989533578943	-1.8457211935263158	H	-4.7723017831578955	-0.55451259673684217	-2.5040725670526314
H	-2.5250176842105343E-002	-2.8612407533578947	-2.7507995935263154	H	-5.4840379831578954	0.73605762326315793	-2.9901665670526314

C	-1.3537576294736842	0.47960723414210527	-0.92792329436842103	C	-1.3785807278947368	0.42959793332105267	-0.81243816738421049
C	-2.0373485294736846	1.3093998441421053	-7.2242563684210143E-003	C	-1.9531404278947369	1.3509543533210526	-1.5945907384210506E-002
C	-1.5277103294736842	1.1033876141421053	1.3157197556315789	C	-1.5587693278947368	1.1830004633210527	1.3268442726157894
O	-0.60917004947368425	6.4818214142105257E-002	1.3120293556315790	O	-0.57713364789473687	0.25672376732105262	1.4822852726157894
C	-0.47954437947368428	-0.43536437585789473	-3.5672861368421016E-002	C	-0.54469700789473685	-0.48317459667894741	0.15498153561578948
O	-2.8519304294736845	2.4098810841421052	-0.35108455436842101	O	-2.7979608278947365	2.4168981533210525	-0.28393373738421057
O	-1.8127935294736841	1.6363466841421053	2.3912352556315790	O	-2.0918817278947368	1.7526991533210527	2.3045274726157894
C	0.95769116052631575	-0.47585072585789473	-0.50614495436842111	C	0.85687442210526310	-0.58983956667894732	-0.45313454738421044
O	1.6604434705263158	0.77572606414210532	-0.46941816436842104	O	1.4533976721052633	0.80680354332105275	-0.66574417738421043
O	-1.4304058294736841	0.36687110544210527	-2.1738683443684210	O	-1.4532845278947368	0.28398618622105265	-2.0577257273842107
C	1.7588222705263159	-1.5920869158578947	0.10467409663157898	C	1.9161935721052632	-1.6065365466789474	0.10969700861578949
O	3.0140311705263154	-1.6805793158578948	-0.56914155436842107	O	3.1144520721052631	-1.7559863466789472	-0.62132936738421052
H	-3.7130004294736842	2.3128106841421050	0.14808835963157899	H	-3.6760136278947368	2.1578698533210527	-0.42262818738421049
H	-0.84605052947368431	-1.4443333158578946	-0.17090413436842100	H	-0.94002880789473686	-1.4214011466789473	0.28344740261578949
H	1.8883242705263159	-1.4754970158578946	1.1876538556315790	H	2.1891853721052632	-1.3262063466789473	1.1203921026157895
H	1.2093560705263158	-2.5731161158578946	5.2225732631578987E-002	H	1.4417014721052632	-2.6307491466789474	0.13318965391578949
H	3.6609825705263157	-1.0832876158578946	-0.15836391436842101	H	3.7892734721052634	-1.1569116466789473	-0.29818696738421047
H	0.74867861052631568	-0.71589031585789464	-1.5907087443684211	H	0.68205513210526314	-0.92383494667894728	-1.5346095273842106
H	1.7633820705263159	1.0171571841421052	0.44882836563157896	H	1.5283574721052633	1.2561068833210527	0.25031159261578950
O	-2.8938061294736843	1.7161150841421053	-3.9084397443684207	O	-2.9598476278947365	1.7654749533210528	-3.5697982273842106
H	-2.0314429294736844	1.6570383841421052	-4.4324022443684212	H	-2.3375259278947369	1.9165185533210527	-4.3412697273842102
H	-2.5940603294736846	1.2178733641421053	-3.1121339443684208	H	-2.4361518278947369	1.3206059533210528	-2.9451066273842108
O	0.55464527052631574	0.92532763414210528	-4.0100741443684216	O	0.64615920210526312	0.87778974332105264	-3.7842430273842105
H	-0.10326798947368428	0.56715316414210526	-3.4630391443684210	H	5.6218946052631917E-003	0.42200696332105264	-3.1640273273842108
H	7.0065079526315729E-002	1.0008806941421053	-4.8245801443684213	H	0.23674784210526320	0.73581930332105272	-4.6529454273842106
O	0.55690800052631573	3.1720241841421055	-1.9133111443684210	O	0.14963209210526318	3.1202017533210524	-1.7484393273842107
H	-0.25678561947368428	2.7469496841421055	-2.2506741443684208	H	-2.7336554947368086E-003	2.8920754533210524	-2.7056736273842108
H	1.0341778705263158	2.3874953841421052	-1.5627469443684210	H	0.33323334210526318	2.2335372533210527	-1.3821806273842105
O	-5.3722644294736845	-0.54922503585789473	-2.7608159443684208	O	-3.3385308278947368	-2.2770780466789474	-2.5838013273842106
H	-4.4817183294736846	-0.16515430585789476	-2.7106515443684209	H	-2.4054975278947368	-2.2628812466789476	-2.4435818273842105
H	-5.8322024294736847	-9.4562765857894748E-002	-3.5130969443684208	H	-3.6971927278947367	-2.0718932466789477	-1.7109749273842105
O	-3.2411532294736845	-2.8930313158578946	-2.5243346443684209	O	-0.77471667789473686	-3.1802014466789474	-1.7871392273842106
H	-2.3294467294736845	-3.0167882158578947	-2.2226639443684206	H	-0.39031733789473683	-3.9646758466789471	-2.1197001273842107
H	-3.6034480294736846	-2.5316854158578947	-1.6994310443684211	H	-0.47489471789473681	-2.5058325466789477	-2.4229778273842104
O	-3.1507738294736845	-1.2995252158578947	-5.1212851443684215	O	-3.6353379278947369	-0.95288014667894738	-5.2052380273842109
H	-2.2080063294736845	-1.2235647158578946	-5.4506116443684212	H	-2.7185548278947369	-0.87551674667894730	-5.5917347273842104
H	-3.0072069294736843	-1.9024034158578949	-4.3547623443684209	H	-3.3981843278947368	-1.1899447466789472	-4.2515646273842105

C	-1.3313521831578947	0.40453335526315787	-0.94492046705263144	C	-1.3705215263157893	0.48655098345789460	-0.87942811142105259
C	-1.8987065831578946	1.3248682832631578	-6.5389437052631560E-002	C	-1.9278709263157894	1.3466701334578948	3.4832538578947386E-002
C	-1.4947585831578947	1.0849787132631579	1.2615616329473684	C	-1.3845060263157893	1.1925749334578948	1.3271322885789472
O	-0.59233290315789466	-3.6997673684213694E-004	1.2838936329473685	O	-0.52740202631578925	8.1096247757894660E-002	1.3108346885789475
C	-0.54808659315789476	-0.57267354673684212	-9.2278637052631562E-002	C	-0.55941553631578933	-0.46889864654210534	-3.4486114210526275E-003
O	-2.7639852831578948	2.3683743532631576	-0.43462144705263156	O	-2.8565662263157892	2.3737105334578947	-0.22768646142105264
O	-1.8690169831578947	1.6291035532631579	2.3132846329473686	O	-1.6077601263157892	1.8006798334578948	2.3778850885789473
C	0.91930224684210526	-0.51281170673684218	-0.54200414705263156	C	0.84939588368421071	-0.59295127654210533	-0.49209013142105262
O	1.4658400168421053	0.89769519326315783	-0.46529680705263160	O	1.6030233736842108	0.64391182345789466	-0.58511017142105259
O	-1.3895387831578947	0.32598379926315790	-2.2231729670526317	O	-1.4476252263157894	0.35061895345789462	-2.1390843114210529
C	1.7579915168421054	-1.4705422467368421	0.24897286294736842	C	1.7261030736842107	-1.4969687665421052	0.27473053857894736
O	3.1080887168421052	-1.6219390467368422	-0.36807376705263156	O	2.8898908736842110	-1.7622741665421053	-0.46432553142105260
H	-3.6991566831578946	2.0505499532631579	-0.25234651705263156	H	-3.4299802263157892	1.9720755334578948	-0.93309761142105263
H	-0.86403610315789470	-1.6298402467368420	-0.13832483705263157	H	-0.98817073631578933	-1.5350019665421053	0.12140501557894738
H	1.8685034168421053	-1.1685978467368421	1.3157794329473684	H	1.8477043736842107	-1.0362763665421053	1.2893649885789475
H	1.4383472168421054	-2.5104808467368418	0.22646375294736842	H	1.2026462736842107	-2.4387314665421056	0.42158602857894739
H	3.7641806168421055	-1.1035171467368421	0.12481918694736843	H	3.5213312736842108	-1.1101669665421052	-0.21483414142105262
H	0.97963111684210524	-0.69407744673684202	-1.6762026670526315	H	0.79639118368421069	-0.82138642654210536	-1.5447078114210524

H	1.6633322736842107	1.0147670734578946	0.32604171857894737
O	-3.1252284263157892	1.6801126334578946	-3.9280888114210524
H	-2.5949743263157892	1.6493080334578947	-4.7628715114210527
H	-2.6030927263157890	1.1748461334578948	-3.2872701114210527
O	0.81311761368421065	0.34164148345789469	-3.8668052114210529
H	0.12920649368421069	0.60161406345789459	-3.2373992114210526
H	0.30271302368421071	0.32545604345789469	-4.7059012114210521
O	-2.8675598515789286E-002	2.9205735334578944	-1.8353416114210526
H	0.69371106368421065	2.7780986334578945	-2.4967492114210526
H	-0.68219245631578929	2.2314388334578945	-2.0549026114210527
O	-3.5299868263157892	-2.4183869665421054	-2.3990238114210527
H	-2.7081851263157892	-2.7232411665421057	-1.9871668114210528
H	-3.9177260263157891	-1.9803489665421055	-1.6508754114210524
O	-2.5000513263157891	4.0599022334578949	-3.2082687114210526
H	-2.9059570263157890	3.1949984334578945	-3.5041830114210528
H	-2.4347153263157892	4.0243751334578945	-2.2334158114210529
O	-3.4625232263157892	-1.1001202665421053	-4.9888721114210519
H	-2.6125996263157889	-1.0695786665421052	-5.4513655114210522
H	-3.3746105263157893	-1.7778796665421053	-4.2256326114210525

C	-1.4003385315789474	0.46006591842105271	-0.87191142384210518
C	-1.9033741315789474	1.3473308184210528	5.5041702157894765E-002
C	-1.3911508315789474	1.1500145484210527	1.3380887061578948
O	-0.49013529157894736	0.10812458842105266	1.3168140061578948
C	-0.53231787157894739	-0.46831932157894740	3.8241159157894763E-002
O	-2.6470845315789475	2.5010067184210527	-0.21911318384210526
O	-1.6731714315789472	1.7116072184210527	2.3855730061578946
C	0.86150262842105274	-0.63052931157894732	-0.53533483384210523
O	1.5310848684210527	0.70127513842105260	-0.69877239384210521
O	-1.5135321315789474	0.29086705842105265	-2.1295195938421054
C	1.7619979684210527	-1.5635402815789472	0.23746653615789476
O	3.0200136684210523	-1.6844156815789473	-0.40819506384210530
H	-3.5666057315789477	2.3287162184210528	-0.35428897384210523
H	-0.95243443157894725	-1.4806519815789472	0.15288829615789476
H	1.8347954684210528	-1.2995959815789473	1.2839301061578947
H	1.3749000684210526	-2.5998805815789474	0.16047936715789476
H	3.4233010684210523	-0.77573613157894739	-0.43326124384210524
H	0.72518210842105257	-1.0920786815789472	-1.5200606938421053
H	1.5373670684210528	0.99573972842105263	0.20193451915789476
O	-3.0755912315789473	1.8241394184210526	-3.7422575938421052
H	-2.4584006315789475	1.6048206184210527	-4.5249213938421056
H	-2.7101483315789476	1.3290483184210526	-2.9519217938421054
O	0.55958894842105256	0.81315885842105262	-4.0097644938421055
H	-9.6795861578947373E-002	0.43723769842105265	-3.4224826938421051
H	4.4307342105263936E-004	1.1115002284210527	-4.7466194938421049
O	0.26810314842105265	2.8506213184210529	-2.0649761938421052
H	8.7063516421052645E-002	2.7558407184210529	-3.0438623938421054
H	0.50476366842105258	1.9409114184210527	-1.7078372938421054
O	-3.3967901315789475	-2.6217605815789473	-2.3877497938421053
H	-2.4669844315789473	-2.2574236815789472	-2.3225937938421053
H	-3.9451336315789471	-2.0327917815789474	-1.8278787938421053
O	-3.6473727315789475	-0.80909329157894738	-5.0649525938421052
H	-3.2169959315789476	-1.5680515815789473	-5.3973293938421056
H	-3.2522866315789476	-0.71636567157894737	-4.1588069938421057
O	0.48984909842105262	-2.3218115815789471	-3.7197191938421055
H	1.1037064684210527	-2.9280710815789472	-4.2015450938421051
H	0.29329894842105264	-1.7264936815789473	-4.4445369938421049

C	-1.3785639321052634	0.34059248447368418	-0.96138795473684213
C	-2.0360711321052634	1.2233672984736841	-9.8846964736842041E-002
C	-1.4741071321052632	1.1621874684736841	1.2015124352631579
O	-0.49052396210526317	0.22895316047368414	1.2356785452631578
C	-0.49903310210526319	-0.48110489152631586	-4.2057854736842065E-002
O	-2.8319575321052635	2.3539230784736844	-0.45123791473684205
O	-1.7005004321052630	1.8078614784736842	2.2395316452631580
C	0.91500092789473675	-0.53914313152631588	-0.48275191473684198
O	1.5349076678947369	0.71357180847368418	-0.50956483473684200
O	-1.4790782321052633	0.17035875947368415	-2.2260373547368419
C	1.7513891678947369	-1.5328571215263158	0.34437062526315798
O	3.1298124678947365	-1.6211634215263158	2.4129615263157933E-002
H	-3.7712930321052633	2.0601688784736840	-0.76524471473684208
H	-0.86382620210526317	-1.4492979215263158	0.14928323926315795
H	1.7010054678947366	-1.2363128215263157	1.3840457452631578
H	1.3487454678947368	-2.5390388215263155	0.25198567126315796
H	3.4741524678947369	-0.75147979152631583	-0.20997228473684207
H	1.0802164878947367	-0.96773092152631579	-1.4924125547368421

H	1.5897245678947369	1.0571444284736842	0.40897682526315793
O	0.58546474789473679	0.79044387847368414	-3.9038074547368415
H	1.4863199894736792E-002	0.37118288847368414	-3.1717790547368421
H	-5.4903452105263206E-002	0.64861850847368419	-4.7051706547368415
O	-2.9926646321052632	1.8744450784736841	-3.7746938547368418
H	-2.3043824321052635	1.7374752784736842	-4.4946822547368424
H	-2.6410286321052632	1.2865107784736842	-3.1346184547368421
O	-3.5306177321052634	-0.94657192152631586	-4.7215459547368424
H	-2.7160531321052632	-0.62231097152631587	-5.1254424547368416
H	-3.1571495321052634	-0.72648214152631585	-3.8490747547368418
O	-4.0405035321052631	-2.7152956215263160	-2.0615395547368420
H	-3.2411107321052635	-2.1857052215263160	-2.1402321547368417
H	-4.1605842321052631	-2.4025390215263158	-1.1258211547368422
O	0.49848905789473680	2.7972273784736843	-1.8061208547368419
H	0.14069806189473680	2.5910452784736844	-2.7304807547368419
H	1.0597139478947368	2.0046223784736843	-1.5398370547368421
O	0.69709568789473675	-2.4392287215263160	-3.6200472547368419
H	1.0499540378947367	-3.0324720215263157	-4.2801510547368418
H	0.16420409789473678	-1.8436719215263158	-4.2435214547368423

C	-1.4111318468421052	0.31132961342105264	-0.85155551200000001
C	-1.9949770468421053	1.3300843844210526	-9.5218862000000015E-002
C	-1.3556160468421052	1.3687569844210528	1.1804412280000001
O	-0.56391913684210526	0.22907037142105263	1.2890971879999999
C	-0.52290698684210524	-0.50542905557894735	4.2475257999999988E-002
O	-2.9414260468421052	2.2672998844210528	-0.43580237199999999
O	-1.5369993468421053	2.1151412844210524	2.1572481880000001
C	0.96169428315789474	-0.54168157557894747	-0.45560094200000006
O	1.6000978531578949	0.70325810442105263	-0.54032456200000012
O	-1.6988185468421053	-6.1796275578947346E-002	-2.0406841120000001
C	1.7945756531578947	-1.5343122155789473	0.31113116799999996
O	3.1416642531578947	-1.6227309155789473	-5.7369402000000014E-002
H	-3.7083071468421052	1.8272835844210527	-0.85228311200000006
H	-0.82775638684210529	-1.5627326155789474	0.12685303100000000
H	1.5998225531578947	-1.3344908155789472	1.4106472880000001
H	1.4426686531578947	-2.5466014155789471	0.15588136300000000
H	3.4906240531578949	-0.73332041557894723	-0.16219789200000001
H	1.0194636831578949	-0.83718721557894726	-1.5148945119999999
H	1.5112475531578948	1.1280583044210526	0.33215656799999999
O	-3.1415460468421053	1.8947086844210528	-3.6457692120000003
H	-2.7261562468421054	1.8973152844210528	-4.5681342120000004
H	-2.6312670468421051	1.1763820044210527	-3.2143028120000001
O	0.52991135315789484	0.65886424442105263	-4.0630583119999999
H	-9.6640686842105208E-002	0.47129791442105262	-3.3164344120000000
H	-7.9056596842105212E-002	0.52860997442105262	-4.8179401120000005
O	-3.9982631468421048	-2.3150624155789474	-2.0205869120000002
H	-3.0588120468421054	-2.0101976155789472	-1.9165561120000001
H	-4.3351344468421047	-2.0219839155789474	-1.1394028119999999
O	-3.4770792468421052	-0.76226555157894728	-4.8579207120000003
H	-2.5311376468421054	-0.62414474557894728	-5.1812342120000006
H	-3.3116910468421050	-1.3333820155789473	-4.1164078120000003
O	0.57923934315789471	-2.4160891155789472	-3.5093468120000000
H	0.77314832315789472	-3.2011074155789472	-4.0805644120000002
H	6.7100466357894781E-002	-1.8371472155789474	-4.0908833119999999
O	0.87279669315789477	2.8549706844210525	-1.9794785119999998
H	0.88760299315789482	2.4428256844210527	-2.8507948120000002
H	0.88040920315789473	1.9386031844210527	-1.5499441119999999

C	-1.5192227347368421	0.21985834368421045	-0.94417316021052633
C	-1.9874747347368422	1.2852959636842103	-0.17125578021052634
C	-1.3943490347368419	1.3746720336842104	1.1344940797894736
O	-0.45191790473684201	0.33466906368421045	1.3137038397894736
C	-0.50163119473684203	-0.43623415631578955	5.0483219789473649E-002
O	-2.9182171347368420	2.2697282036842106	-0.48815191021052634
O	-1.6927655347368420	2.0829822036842103	2.1268966397894737
C	0.93561681526315799	-0.63989369631578952	-0.47350306021052641
O	1.5676606652631579	0.63043285368421043	-0.70247281021052643
O	-1.8082651347368419	-0.17287148631578952	-2.1240698602105264
C	1.8021104652631581	-1.4445948963157895	0.51788661978947359
O	3.1327779652631582	-1.5571975963157894	-3.1312202105263642E-003
H	-3.7698960347368420	1.9004522036842104	-0.74629486021052638
H	-0.82186231473684201	-1.4105235963157896	0.30820366378947361
H	1.8225750652631580	-1.0469826963157896	1.5204758397894735
H	1.4245073652631579	-2.4989327963157892	0.49248450978947367
H	3.3295656652631580	-0.72429539631578965	-0.51948878021052636
H	0.91095968526315796	-1.1515238963157897	-1.4376884602105264

H	1.9398280652631581	0.98495934368421045	0.14560148978947363	H	1.6220794110526313	1.0835771633157893	1.0306629663157985E-002
O	0.20101849626315799	0.85411312368421044	-3.8159968602105261	O	-3.2075424889473685	1.7983649133157895	-3.5466332503368423
H	-0.37401366473684194	0.36053140368421044	-3.2018090602105262	H	-2.5043893889473683	1.9531139133157895	-4.1899076503368420
H	-0.26660645473684202	0.73887539368421051	-4.6021626602105261	H	-2.7980807889473684	1.2209093833157896	-2.9142107503368422
O	-3.1036093347368419	2.0977308036842102	-3.4357372602105265	O	-3.5906306889473685	-2.0664707866842105	-1.7843783503368418
H	-2.6956137347368418	1.9643077036842105	-4.3230497602105267	H	-2.8688784889473684	-1.4152375866842106	-1.8869412503368419
H	-2.6857009347368419	1.3616191436842104	-2.8774728602105264	H	-3.9791975889473687	-1.8178246866842107	-0.87629875033684201
O	-3.7017023347368418	-2.2707562963157892	-2.0869390602105264	O	0.49611614105263147	1.2102530833157896	-3.6763535503368421
H	-3.0309665347368422	-1.7039760963157897	-1.8184045602105265	H	-0.19210640894736858	0.63536656331578945	-3.2497142503368424
H	-4.1793827347368424	-2.3291774963157899	-1.2536897602105264	H	0.17316586405263143	1.3219633833157896	-4.6494943503368420
O	-3.3433026347368422	-0.89188999631578958	-4.6334009602105262	O	-3.5978023889473683	-0.89961978668421061	-4.4964666503368420
H	-2.4286913347368420	-1.1490951963157896	-4.9709095602105267	H	-2.6801453889473685	-1.1715036866842106	-4.7056560503368416
H	-3.2808074347368419	-1.2094899963157895	-3.6811722602105266	H	-3.5081275889473686	-0.98166058668421052	-3.5366986503368421
O	0.41400223526315799	2.9069400036842108	-1.9582392602105263	O	-4.9676198947368577E-002	3.3662286133157893	-1.8527436503368422
H	2.2498455263157996E-002	2.4314799036842105	-2.7348070602105263	H	-0.19645930894736857	3.1125422133157894	-2.7719201503368422
H	0.72582207526315801	2.1005141036842105	-1.4863790602105265	H	-0.42756112894736853	2.6087332133157894	-1.4950947503368419
O	-0.17896099473684202	-3.5882457963157899	-1.6590641602105265	O	0.85954629105263147	-2.1400013866842107	-3.7590825503368426
H	0.32334689526315796	-4.3224260963157901	-1.9081121602105262	H	1.0889976910526316	-2.7820459866842104	-4.4771901503368419
H	0.15991686826315801	-2.9043199963157900	-2.2953978602105263	H	0.21157086205263143	-1.5647981866842104	-4.1937494503368420

C	-1.4853544294736840	0.29693081584210523	-0.91033450872105270	C	-1.4050546257894736	0.39520175631578941	-1.0260204281157894
C	-2.1530721294736841	1.2069897858421053	-4.5592748721052623E-002	C	-2.0293065257894738	1.2762715963157896	-0.16491601811578943
C	-1.3028952294736840	1.3415483558421053	1.0956304312789475	C	-1.4581636257894737	1.1620165863157894	1.0949665618842106
O	-0.39502292947368411	0.41139926484210526	1.1646150712789474	O	-0.52125897578947378	0.17920093631578943	1.1327334918842107
C	-0.39816355947368409	-0.38206911415789480	-1.2630558721052620E-002	C	-0.49893917578947378	-0.54484271368421056	-0.10809703811578947
O	-3.2010018294736842	2.0932110158421056	-0.24607828872105264	O	-2.8099030257894735	2.4219567863157896	-0.53026399811578939
O	-1.4385129294736840	2.1618159158421051	2.0472250912789476	O	-1.6197532257894738	1.8906859863157894	2.1431716718842106
C	0.98746945052631596	-0.53943338415789466	-0.53469511872105258	C	0.99522132421052623	-0.71286721368421058	-0.48885244811578943
O	1.6374773705263161	0.74704049584210530	-0.67504820872105264	O	1.4290390742105263	0.66139769631578949	-0.73278952811578946
O	-1.8375688294736841	-0.13641714415789474	-2.0561364087210525	O	-1.5783879257894737	0.26024512631578944	-2.3093330281157893
C	1.7582900705263160	-1.5348214841578947	0.40911394327894740	C	1.7265578742105263	-1.5070831136842107	0.56267009188421058
O	3.2073019705263159	-1.6916153841578949	0.14222345127894739	O	3.1229270742105264	-1.6566987136842104	0.28650268008421054
H	-3.8197071294736840	1.7913160158421053	-0.96947420872105272	H	-3.5504471257894736	2.0821273863157894	-1.1402632281157894
H	-0.73182868947368407	-1.3756367841578947	0.37391859127894739	H	-0.96636462578947391	-1.4646996136842105	7.3751871884210563E-002
H	1.5757404705263161	-1.2065680841578947	1.4709038912789474	H	1.7031235742105264	-1.0414791136842108	1.5786844718842106
H	1.3590769705263159	-2.4754089841578946	0.35238966497894736	H	1.3157307742105262	-2.5182579136842103	0.57295031188421053
H	3.5236675705263161	-0.85206658415789471	-0.26933681872105264	H	3.5321235742105261	-0.78192071368421068	0.36632383788421052
H	0.80082694052631598	-0.94432098415789478	-1.5220046087210526	H	1.0490758142105263	-1.2122791136842106	-1.4488005281157894
H	1.9132768705263161	1.0881062658421052	0.18531134127894738	H	1.5637797742105264	1.1110243663157893	0.13758125188421055
O	-3.1606253294736839	2.1565086158421050	-3.2327215087210526	O	-3.1435654257894736	2.0964308863157894	-3.6055031281157897
H	-2.5928699294736841	2.2744000158421054	-4.0477245087210525	H	-2.4949354257894738	2.2429661863157895	-4.3543334281157895
H	-2.8009378294736837	1.2396033358421052	-2.9759818087210528	H	-2.7127816257894737	1.3883806063157893	-3.1357750281157895
O	-3.8355248294736839	-2.0287507841578947	-1.8830034087210525	O	0.51281869421052617	1.1477522163157894	-3.8418549281157892
H	-2.8912104294736838	-1.7747803841578946	-1.7396408087210529	H	-9.1560225789473801E-002	0.64401292631578944	-3.2866602281157897
H	-4.1337697294736842	-1.9388606841578948	-0.93334340872105270	H	-0.12656706578947380	1.2210838063157894	-4.5590864281157888
O	-3.4987738294736839	-0.71531048415789478	-4.4619034087210530	O	-3.5590645257894735	-2.0237212136842104	-1.8136505281157895
H	-2.5914771294736840	-0.79765398415789468	-4.8444625087210529	H	-3.0259590257894735	-1.2574879136842108	-1.9366227281157895
H	-3.3300330294736842	-0.84712228415789470	-3.4937938087210529	H	-3.8551617257894737	-1.9205701136842106	-0.90809762811578953
O	0.40534470052631599	1.1708844158421052	-3.8060611087210523	O	-0.34517217578947379	3.4738393863157895	-1.9212965281157894
H	0.47021667052631599	0.28394447584210525	-3.3752599087210529	H	0.18504871221052621	3.0920462863157896	-2.6489966281157891
H	-1.8710799473684037E-002	0.94838892584210521	-4.6432902087210524	H	-0.83931440578947381	2.6450130863157897	-1.6529758281157894
O	-0.14056103947368406	3.2469036158421054	-1.7646645087210526	O	-3.4727491257894738	-0.99534581368421049	-4.5240460281157890
H	0.13189686402631595	2.9465077158421051	-2.6735231087210529	H	-2.5956047257894737	-0.88185231368421058	-4.9339731281157890
H	-0.62287256947368408	2.4249539158421052	-1.5721842087210525	H	-3.2582870257894738	-1.4037531136842105	-3.6796797281157891
O	-0.40036386947368408	-3.4968833841578948	-1.3749471087210527	O	0.97288442421052623	-2.0026886136842106	-3.8268838281157889
H	0.10922962852631596	-4.3250397841578945	-1.3811219087210524	H	1.1544423742105263	-2.4655364136842106	-4.6669100281157894
H	-0.14440136947368407	-3.1312193841578946	-2.2212885087210528	H	0.31270586421052621	-1.4286930136842106	-4.1730071281157892

C	-1.4478555889473688	0.33975447331578945	-0.90106475033684208	C	-1.4186091963157894	0.27580807021052622	-1.0425134426315790
C	-1.9144262889473684	1.4153593433157896	-0.15985606033684202	C	-2.0200356963157891	1.2817078602105263	-0.28153428263157904
C	-1.3236842889473688	1.3492067733157895	1.1502555296631580	C	-1.4829564963157895	1.2904378102105261	1.0124066073684210
O	-0.53983829894736857	0.26068168331578950	1.2432582396631580	O	-0.63848297631578943	0.20139039021052618	1.2194756973684211
C	-0.47349505894736854	-0.45638276668421052	-2.8360940336842055E-002	C	-0.49473139631578944	-0.49064578978947382	-8.4168252631579010E-002
O	-2.6868309889473685	2.4931852133157895	-0.64192765033684207	O	-2.9541508963157890	2.2428758102105260	-0.72083995263157896
O	-1.5394846889473688	2.0636110133157892	2.2137458496631579	O	-1.6853481963157895	2.0049441102105261	2.0409238573684210
C	0.95466943105263147	-0.77912388668421051	-0.51360246033684209	C	1.0241192436842106	-0.62778568978947380	-0.42036927263157903
O	1.6384092110526316	0.46870825031578950	-0.75297405033684206	O	1.6908653036842105	0.60267570621052624	-0.64272655263157907
O	-1.8412787889473683	5.0354433315789482E-002	-2.0708687503368424	O	-1.6114178963157895	-0.10393602978947380	-2.2513202426315790
C	1.6732279110526314	-1.5473699866842106	0.58185505966315798	C	1.7127733036842105	-1.4009248897894737	0.71866387736842097
O	3.1183923110526317	-1.6500379866842105	0.41981874166315797	O	3.1866620036842104	-1.6285332897894738	0.42071142736842099
H	-3.4245396889473683	2.0806556133157894	-1.1634691503368420	H	-3.5034564963157893	1.9840044102105263	-1.4839558426315791
H	-0.94285698894736847	-1.4080984866842106	0.28217637966315795	H	-0.97497939631578934	-1.4476597897894738	9.2680787368420975E-002
H	1.4696015110526313	-1.1169986866842105	1.5809178496631580	H	1.5432718036842106	-0.92981818978947373	1.6745058573684208
H	1.2388890610526313	-2.5128184866842105	0.43853622406315795	H	1.2689772036842106	-2.3887569897894738	0.79708587736842107
H	3.4412972110526314	-0.97895738668421051	-0.21896434033684203	H	3.5145365036842104	-0.79364528978947368	9.4452337368420985E-002
H	0.97772461105263142	-1.1553062866842105	-1.4697823503368419	H	1.1468882736842105	-1.2482934897894737	-1.2963689426315790

H	1.6960750036842105	1.1761552702105262	0.15289045736842100
O	0.19494298218421058	1.2921367202105261	-3.6932354426315790
H	-0.19129557631578942	0.61386081221052624	-3.0714964426315792
H	-0.21801999631578939	1.0291694502105262	-4.5688069426315785
O	-2.9332539963157895	2.0363881102105261	-3.7697280426315793
H	-2.4619638963157895	2.0871823102105260	-4.6219379426315790
H	-2.3953814963157898	1.3793935402105262	-3.3478670426315791
O	-3.9018384963157899	-2.1888371897894738	-1.9714775426315789
H	-2.9451277963157896	-1.9754967897894740	-1.9599021426315790
H	-4.1895340963157892	-2.1144540897894739	-0.98354884263157905
O	-3.8657999963157899	-0.84859118978947379	-4.2836211426315787
H	-2.9782440963157892	-0.53715298978947379	-4.1068152426315789
H	-3.9628888963157900	-1.6001633897894738	-3.7230230426315791
O	-0.57892353631578941	-3.3447855897894736	-1.9132725426315789
H	0.15621130368421060	-3.9530258897894734	-1.8226751426315793
H	-0.16229057631578941	-2.6005627897894739	-2.3396611426315790
O	-0.18977963631578942	3.4551849102105261	-1.8750641426315791
H	0.23725979268421060	2.7336264102105261	-2.4434448426315791
H	-1.1380149963157895	3.1529480102105261	-1.9655095426315792

H	1.6714584352631581	1.1635568647894736	0.39534587073684213
O	-2.7927930647368422	2.0750428547894737	-3.6834992592631579
H	-2.2565596647368418	2.0171491547894735	-4.4698250592631581
H	-2.6003857647368420	1.1839675947894737	-3.3345249592631578
O	-3.3035653647368424	-0.77015924521052637	-4.3144349592631581
H	-2.4709478647368419	-1.0496282452105266	-4.7974708592631581
H	-3.0686050647368424	-1.0552484452105264	-3.4093517592631577
O	0.85465573526315797	1.6578581547894735	-3.5437066592631576
H	0.47390839426315801	0.89584971478947362	-3.1347735592631576
H	0.18866110526315799	2.0088976547894735	-4.1647588592631584
O	-3.4869172647368423	-2.4544511452105264	-1.8743869592631579
H	-2.6492539647368423	-2.3324780452105260	-1.4741524592631579
H	-4.0383422647368423	-2.2655981452105260	-1.1162206592631578
O	-6.2327454736842036E-002	3.5410242547894737	-2.0052976592631575
H	0.47591633626315799	2.9925870547894737	-2.5806171592631575
H	-0.84325326473684203	2.9794994547894738	-1.9790169592631581
O	1.2721570852631578	-1.6546987452105266	-3.8233061592631574
H	1.8686505352631579	-2.0999291452105262	-4.5171557592631579
H	0.54641278526315795	-1.2674258452105265	-4.3854279592631578

C	-1.3808052615789475	0.18486570600000007	-0.90564039769999993
C	-1.9092869615789474	1.3320925460000002	-0.28720453769999998
C	-1.4647200615789475	1.3988392760000001	1.0533597023000001
O	-0.54816763157894743	0.39645744200000010	1.2605922823000002
C	-0.42978642157894742	-0.44940704399999998	7.7817522300000025E-002
O	-2.9535594615789473	2.0969529360000001	-0.86812299769999990
O	-1.7511711615789474	2.2422806360000003	1.9300965023000001
C	1.0330390684210524	-0.50595630399999991	-0.35318947769999998
O	1.7149914384210525	0.75935745600000004	-0.25783931770000001
O	-1.6510203615789474	-0.25557972399999995	-2.0292983976999999
C	1.7644833384210525	-1.5755516640000000	0.45309831230000003
O	3.1576894384210528	-1.4822848639999999	0.19055739530000004
H	-3.4794839615789472	1.4898276360000000	-1.4415635977000001
H	-0.81453206157894753	-1.4872692639999998	0.28853288730000004
H	1.5301991384210525	-1.5659953639999997	1.5048496023000002
H	1.4052166384210525	-2.4987066640000002	3.2217772300000030E-002
H	3.3152622384210524	-0.53896373399999997	0.27848263060000006
H	1.0761609084210526	-0.77944356399999992	-1.4221857977000001
H	1.3854911384210526	1.2384845560000000	0.49543991230000006
O	-3.7193043615789474	-2.2684765640000002	-1.9376599977000000
H	-2.8665475615789475	-1.8202542640000001	-1.8488602976999999
H	-4.0503619615789477	-2.2290658639999998	-0.98213129769999996
O	-2.8915288615789474	1.9794934360000001	-3.7151830976999998
H	-2.6025415615789473	2.0287396360000001	-4.6743333976999999
H	-2.3969488615789474	1.1825732060000000	-3.4827496976999996
O	0.37004437842105253	1.1921937060000001	-3.8552913976999998
H	0.12230251242105254	0.28083277600000012	-3.7878022976999994
H	-7.9577061578947478E-002	1.5046975360000001	-4.6701186977000004
O	-3.6377027615789475	-0.73872256399999991	-4.1869021977000003
H	-2.8105898615789475	-0.82215326399999999	-4.6983733977000002
H	-3.5049376615789476	-1.4470865639999999	-3.5702299976999998
O	-0.47479185157894743	-3.3287999639999999	-1.8392159976999998
H	0.12974865742105254	-4.0503356639999994	-1.9205057976999997
H	-0.12666375157894746	-2.7517760639999995	-2.5546978976999997
O	-0.23457559157894745	3.3021660360000000	-2.1908575977000000
H	-6.1533961578947444E-002	2.5865295360000005	-2.8042124977000000
H	-0.99545976157894733	2.8733895360000004	-1.6927901976999999

C	-1.4138376905263157	0.17149059521052631	-1.0125163500526315
C	-2.0379066905263157	1.1394342052105264	-0.29794216005263152
C	-1.5070903905263155	1.2609256952105263	0.98840393994736842
O	-0.61058769052631579	0.25214972621052634	1.2013591899473683
C	-0.44392459052631594	-0.50731500478947367	-1.5273360052631579E-002
O	-2.8951074905263159	2.0938442252105260	-0.90996885005263151
O	-1.7866700905263158	2.1006536252105263	1.8649928499473685
C	0.99562392947368417	-0.54722752478947378	-0.42922121005263159
O	1.5204194894736842	0.76198536521052629	-0.67984945005263153
O	-1.4842477905263158	-0.12347433478947367	-2.2497990500526317
C	1.7941003094736843	-1.3331455747894736	0.60194692994736843
O	3.2056132094736842	-1.4691778747894737	0.43319439994736841
H	-3.6421765905263159	1.5169849252105263	-1.2626070500526316
H	-0.76083429052631579	-1.5455265747894735	0.17605876994736841
H	1.5964776094736841	-0.99224197478947374	1.6054200499473685
H	1.3640214894736842	-2.3759685747894737	0.60788260994736842
H	3.6886237094736845	-0.67836047478947359	0.63353989994736837
H	1.1254115394736841	-1.1002117747894737	-1.3650106500526316
H	1.2920920194736842	1.3751813252105263	0.10938949094736841
O	-3.2132468905263156	-1.1227901747894737	-4.1040107500526313
H	-2.4345836905263156	-0.75434087478947354	-3.6562603500526318
H	-2.9894032905263157	-2.0174557747894739	-3.7895028500526320
O	-3.0576743905263158	1.7078574252105263	-3.7953871500526316
H	-2.6802693905263157	1.8733941252105264	-4.6825048500526316
H	-2.6186286905263159	0.84127691521052639	-3.5325239500526320
O	0.63084301047368418	1.1251137852105264	-3.8232694500526319
H	7.2105669473684131E-002	0.39296299621052633	-3.4315790500526320
H	0.16252897947368417	1.4804048252105264	-4.6308096500526315
O	-3.3593286905263158	-2.4439256747894738	-1.6947462500526316
H	-2.6192992905263157	-1.8172412747894737	-1.7360178500526315
H	-3.7687609905263155	-2.3397198747894739	-0.77434038005263151
O	7.4464794736841533E-003	3.2809719252105261	-2.1031243500526320
H	0.26143971947368416	2.8997184252105264	-3.0030342500526319
H	-0.68882319052631591	2.6325906252105260	-1.8430225500526316
O	1.3756026994736841	-2.0755553747894737	-3.8242504500526322
H	1.6748415094736844	-2.5493274747894739	-4.6361524500526317
H	0.59275152947368415	-1.5799399747894736	-4.0265384500526311

C	-1.3679846647368421	0.26250049478947357	-1.0029434592631579
C	-2.0901771647368417	1.1870308847894735	-0.21354949926315786
C	-1.5299031647368420	1.2949193147894735	1.1081539907368421
O	-0.55034386473684205	0.36270282378947361	1.2373765907368421
C	-0.49133213473684201	-0.44200873521052636	-1.6399209263157855E-002
O	-2.8346888647368420	2.1805411547894735	-0.72031330926315795
O	-1.7134881647368421	2.2005112547894736	1.9605828407368422
C	0.97698143526315795	-0.60220954521052650	-0.38815947926315791
O	1.6582200352631580	0.69250886478947360	-0.52999366926315794
O	-1.4358817647368420	-1.8639715210526431E-002	-2.2518104592631576
C	1.6822618352631580	-1.5827925452105263	0.56121561073684212
O	3.1195586352631581	-1.5234126452105266	0.38021284073684214
H	-3.4357725647368422	1.8138918547894736	-1.3673410592631579
H	-0.89908376473684193	-1.4098212452105265	0.18928918673684214
H	1.4657260352631580	-1.3568985452105264	1.6249408407368420
H	1.2961233752631580	-2.5623858452105264	0.40551031073684213
H	3.3566696352631578	-0.63407844521052636	2.8004420736842145E-002
H	1.1216566952631579	-1.0259162452105264	-1.4001223592631580

C	-1.3622986973684208	8.4837042105263272E-002	-0.99326448149999991
C	-1.9820605973684211	1.1907434921052633	-0.40233055149999991
C	-1.5723245973684210	1.2859187421052634	0.95463982849999995
O	-0.67393409736842103	0.35017480210526331	1.2259826185000000
C	-0.52274969736842103	-0.54635561789473663	9.9494374000000024E-002
O	-2.8233674973684213	2.1321645221052634	-0.94351908149999997
O	-1.9158671973684211	2.0526249221052630	1.8322662185000000
C	0.93649453263157900	-0.55083053789473668	-0.34195204149999997
O	1.5529920526315790	0.70280841210526335	-0.53077225150000007
O	-1.4389347973684212	-0.37324558789473666	-2.1684503815000000
C	1.7719583026315791	-1.3214651778947366	0.70428851849999996
O	3.1906635026315788	-1.5574688778947368	0.15705887550000003
H	-3.6020399973684207	1.6973651221052632	-1.3547842815000000
H	-0.81564149736842106	-1.6048324778947367	0.31987580850000003
H	1.8225241026315788	-0.73361527789473668	1.6162829185000001
H	1.4801108026315790	-2.3458448778947369	0.83400061849999996
H	3.6544347026315789	-0.69441057789473670	7.2363334500000015E-002
H	0.98464522263157894	-1.0487641778947367	-1.3303492815000000

H	1.3153954526315790	1.2801961321052633	0.24916923850000000	H	1.1658484452631579	1.2961474789473684	0.41187418873684212
O	-2.7492163973684209	1.6059839221052634	-3.8326583814999999	O	0.35807746526315803	-2.0591859310526317	-3.5601673712631579
H	-2.1298886973684210	1.8020291221052633	-4.6173845814999996	H	0.13629712526315801	-1.8420983310526315	-4.4806246712631577
H	-2.1980084973684213	0.99811935210526337	-3.2298043814999997	H	-0.21476500473684201	-1.3394925310526316	-3.1467088712631579
O	-3.1014055973684211	-1.2375896778947366	-4.3630329815000000	O	0.57015667826315797	1.1558996789473683	-3.7690046712631577
H	-2.3998991973684212	-0.98729597789473655	-5.0376355815000000	H	-3.2196944736841981E-002	0.54918248894736843	-3.3784855712631581
H	-2.6247250973684211	-1.2138909778947367	-3.5084531814999997	H	0.18162921526315801	1.1837616889473683	-4.6904568712631578
O	0.74209362263157896	1.0960335821052634	-3.9673621814999995	O	-3.1487265247368419	-0.66174893105263155	-4.3435034712631575
H	0.20665105263157901	0.59087019210526326	-3.3435652815000001	H	-2.3248961247368420	-0.60358873105263167	-4.7587320712631573
H	0.11925128263157903	1.2782318721052632	-4.6038966815000002	H	-2.8262530247368418	-0.84574033105263158	-3.4697787712631580
O	-3.2641281973684211	-2.5972481778947367	-1.8557351814999998	O	-2.8468881247368421	-2.8549174310526317	-1.7442530712631579
H	-2.2778772973684212	-2.6476510778947371	-1.9977567814999997	H	-1.8841351247368419	-2.8670375310526319	-1.7961056712631580
H	-3.3743165973684213	-2.1228677778947369	-1.0247066814999999	H	-2.9671349247368419	-2.0428707310526319	-1.2834919712631581
O	0.77888729263157896	-1.9791143778947367	-3.6807950814999999	O	-2.8509239247368421	2.1674827689473686	-3.7931555712631582
H	1.3019399426315790	-2.0775896778947369	-4.4707469814999996	H	-2.1574059247368420	2.1631576689473682	-4.4802393712631572
H	-5.2024697368420969E-002	-1.5825761778947367	-4.0096668815000003	H	-2.3647811247368420	1.9343086689473683	-3.0233867712631581
O	1.4270812631579033E-002	3.0871496221052630	-2.1805877814999999	O	0.16784133526315803	3.2014820689473682	-1.8819755712631578
H	0.53201137163157897	2.5264511221052630	-2.7672903815000001	H	0.13884404526315802	2.3529247689473682	-2.3021420712631580
H	-0.72020589736842100	2.4542998221052632	-1.9474945815000000	H	0.28775116526315803	3.7004009689473683	-2.6958426712631578

C	-1.3474375357894739	0.11088879947368407	-1.0400246271052631	C	-1.3702682563157897	0.20592063615789469	-1.0370966042105265
C	-2.0585797357894742	1.1306576494736842	-0.42647573710526315	C	-2.1843774563157896	1.1500593351578947	-0.33185206421052638
C	-1.5934389357894738	1.3285568894736841	0.87389329289473672	C	-1.5636017563157898	1.2617982651578947	0.97168454578947361
O	-0.62688623578947389	0.37665889947368408	1.1504921228947369	O	-0.64133275631578968	0.21073461615789471	1.1824909957894736
C	-0.44914053578947388	-0.56622052052631577	5.6951383894736821E-002	C	-0.46896555631578962	-0.48574419484210529	-4.4955494210526387E-002
O	-2.9289756357894738	1.9898711794736841	-0.97915572710526311	O	-3.0960061563157897	2.0789604651578948	-0.78767522421052638
O	-1.8453351357894738	2.2768520794736840	1.7180056728947368	O	-1.5752608563157899	2.0821471651578949	1.8530950957894736
C	1.0555956842105261	-0.55803432052631585	-0.34828235710526317	C	1.0367304036842104	-0.57510638484210530	-0.39781601421052637
O	1.6447633642105259	0.69832154947368408	-0.32182263710526315	O	1.5541525536842102	0.71149312515789465	-0.41164610421052639
O	-1.5359410357894738	-0.45775643052631593	-2.1786801271052632	O	-1.4168283563157895	-0.10277992484210532	-2.2782083042105263
C	1.8569895642105263	-1.4046441205263158	0.62331810289473677	C	1.7360934436842101	-1.3911048348421053	0.63726248578947364
O	3.2769599642105258	-1.2322357205263159	0.49184856289473683	O	3.1541110436842104	-1.5117962348421055	0.25048626578947364
H	-3.5512997357894740	1.3331551894736842	-1.4169185271052631	H	-3.7418811563157894	1.6612133651578946	-1.4120180042105264
H	-0.87105733578947386	-1.5007294205263157	0.23740946689473683	H	-0.79295975631578963	-1.5236275348421053	2.6537505789473628E-002
H	1.5612694242105261	-1.0087706205263158	1.6279822728947368	H	1.6312908436842104	-0.93199473484210527	1.6660180957894735
H	1.5274624842105262	-2.4608493205263158	0.52102886289473682	H	1.3582850436842104	-2.4126896348421050	0.75532114578947362
H	3.4911634642105263	-0.33360500052631586	0.40226599289473680	H	3.6211715436842100	-0.69061130484210531	0.33113100578947363
H	1.1465049942105261	-0.90659232052631578	-1.4076535271052630	H	1.2442286736842103	-0.82818233484210524	-1.4231959042105264
H	1.2473829142105262	1.1844755594736840	0.41581753289473683	H	1.5154185136842102	1.0913101451578946	0.45043657578947360
O	-3.4258682357894736	-0.88917882052631581	-4.1006505271052633	O	-2.8261306563157897	1.6452890651578946	-3.7064457042105263
H	-2.6087418357894738	-0.81109772052631590	-4.7041963271052634	H	-2.2043475563157897	1.8731156651578946	-4.4149419042105267
H	-2.8945097357894740	-0.84522552052631594	-3.2778851271052631	H	-2.2042577563157897	0.99800971515789472	-3.2746582042105263
O	-3.1415363357894739	-2.8329069205263160	-1.8368300271052631	O	9.1683636842103677E-003	-2.1886381348421051	-3.3696077042105266
H	-2.2857474357894740	-2.3598691205263158	-1.9795700271052632	H	-4.6844286315789629E-002	-1.9410419348421055	-4.2789090042105267
H	-3.6491915357894742	-2.4264772205263156	-1.1031316271052631	H	-0.56723615631578961	-1.5363056348421054	-2.9045902042105265
O	-2.8889139357894740	1.8250833794736843	-3.8952239271052633	O	0.87891057368421033	0.73790244515789472	-3.5575555042105265
H	-2.1014674357894738	1.9197678794736841	-4.5804598271052628	H	7.8325473684210389E-002	0.34721275515789468	-3.1574092042105266
H	-2.5776430357894742	1.0535188994736842	-3.4220189271052632	H	0.66178413368421030	0.92670265515789474	-4.4937488042105267
O	0.59391868221052613	1.1493411094736841	-3.7544754271052629	O	-3.2324917563157896	-1.0507817348421054	-4.3127590042105268
H	6.5199284210526121E-002	0.31333611947368412	-3.7599575271052630	H	-2.3700960563157896	-0.81591903484210537	-4.7038239042105268
H	0.22396430421052616	1.5465111794736841	-4.5899047271052629	H	-2.9402701563157896	-0.96316503484210536	-3.3906201042105266
O	0.94458838421052616	-2.0163481205263158	-3.5485797271052633	O	0.40094245368421033	2.9124067651578947	-1.9418908042105265
H	0.96019828421052611	-2.5553148205263159	-4.3442363271052633	H	0.41272599368421037	2.0058755651578948	-1.5903923042105264
H	4.6911804210526098E-002	-1.5893428205263160	-3.5995436271052630	H	0.28981742368421032	2.7290595651578951	-2.8750736042105265
O	-1.0451252357894738	-0.43185907052631595	-5.4439975271052630	O	-2.7566371563157896	-3.2429166348421052	-1.6890591042105265
H	-0.97871473578947377	-1.3043984205263159	-5.9546936271052635	H	-1.7792276563157898	-3.0923429348421050	-1.6692706042105265
H	-0.88025753578947386	9.2063129473684113E-002	-6.2370824271052632	H	-3.1972269563157898	-2.5179365348421050	-1.1494790042105265

C	-1.3084614247368420	0.11718648894736838	-1.0270364712631579	C	-1.4412249242105259	3.8683308242105335E-002	-1.0288673413684211
C	-2.0405255247368421	1.1520502689473684	-0.39303699126315783	C	-2.0698346242105261	1.0763299082421054	-0.35501079136842106
C	-1.5391547247368420	1.3345668589473685	0.92423027873684216	C	-1.6133764242105264	1.2290398482421054	1.0185758286315789
O	-0.63589452473684194	0.30497312894736839	1.2027085287368420	O	-0.59537102421052623	0.28451734164210535	1.2571514586315788
C	-0.43067442473684192	-0.45186541105263167	3.3831118736842108E-002	C	-0.46752853421052620	-0.52189633175789463	1.6372494631578957E-002
O	-3.0995475247368418	1.8097701689473684	-0.94481587126315791	O	-3.0055085242105264	1.9905864982421053	-0.82094357136842100
O	-1.7966617247368419	2.1813734689473683	1.7735568287368420	O	-2.0469898242105264	1.9156048982421052	1.9092076586315789
C	1.0450190752631581	-0.41851943105263167	-0.40868167126315791	C	1.0465998257894737	-0.48220278175789472	-0.37489517136842104
O	1.5622059952631582	0.88124890894736829	-0.34911559126315783	O	1.6106020757894739	0.83743016824210526	-0.41057916136842110
O	-1.3794934247368420	-0.31739748105263166	-2.2048481712631580	O	-1.6939924242105264	-0.31587443175789470	-2.2370552413684210
C	1.8193567752631581	-1.3955169310526316	0.57662131873684208	C	1.8812203757894737	-1.3294026017578948	0.56382368863157895
O	3.2262339752631579	-1.4903498310526317	0.34018870873684215	O	3.2295240757894739	-1.3635094017578948	0.35125133863157892
H	-3.6949766247368419	1.1318810689473684	-1.1821535712631579	H	-3.3965490242105263	1.5343972982421052	-1.6579437413684210
H	-0.77028832473684195	-1.4899124310526317	0.15534284273684212	H	-0.67251112421052606	-1.5527769017578947	0.31192291863157895
H	1.7446821752631581	-0.91911543105263160	1.5742949287368420	H	1.6993643757894739	-0.94554210175789466	1.5716247586315790
H	1.4009276352631579	-2.4305825310526319	0.63325622873684206	H	1.4202248257894738	-2.3297773017578947	0.55531748863157893
H	3.5758950752631580	-0.58667933105263159	0.30171723873684209	H	3.6001811757894737	-0.48207345175789468	0.30335659863157899
H	1.1555090952631581	-0.70925903105263155	-1.4179338712631580	H	1.1146754657894737	-0.79474460175789474	-1.4362684413684210

H	1.4004942557894737	1.2112106382421053	0.46295922863157896
O	0.16814978578947382	-2.1508794017578947	-3.2061004413684207
H	0.31466215578947387	-1.5958201017578948	-3.9835547413684207
H	-0.65363172421052618	-1.6890218017578946	-2.8271619413684208
O	-3.0010311242105261	-2.7610900017578945	-2.0449522413684207
H	-2.3423145242105261	-2.1024189017578947	-2.1187427413684210
H	-3.4453422242105263	-2.5063347017578943	-1.2348233413684211
O	-2.8903772242105261	1.6544613982421053	-4.0341903413684213
H	-2.1764977242105261	1.9258269982421052	-4.5930366413684212
H	-2.5719596242105265	0.89110527824210539	-3.5295337413684207
O	-3.4373385242105261	-1.1736617017578947	-4.6007771413684209
H	-2.7647897242105262	-0.94237180175789481	-5.2568348413684207
H	-2.8780482242105263	-1.2798295017578947	-3.7942746413684207
O	0.80737945578947379	0.87844975824210536	-3.7830063413684210
H	8.8583915789473833E-002	0.42687540824210535	-3.2921567413684207
H	0.35777272578947383	1.0292751082421052	-4.6393382413684208
O	0.27215993578947384	3.0573402982421056	-2.0366001413684209
H	-4.3685084210526193E-002	2.2394001982421052	-1.7398552413684210
H	0.64482264578947390	2.7353207982421055	-2.8279371413684209

C	-1.3366700110526315	0.10685427168421052	-1.0056590700894736
C	-2.0497962110526315	1.0948895256842106	-0.38814376008947371
C	-1.6110286110526317	1.1987509456842105	0.97192473991052641
O	-0.73470951105263149	0.19470262168421051	1.2315393999105262
C	-0.49093221105263141	-0.57633453431578952	-3.1031418389473660E-002
O	-2.9627156110526314	1.9260603456842105	-0.95827033008947360
O	-1.8386856110526315	2.0318558456842104	1.9046522999105262
C	1.0514268289473685	-0.50819391431578942	-0.35836514008947362
O	1.5115117589473686	0.81470434568421046	-0.62493186008947366
O	-1.3575516110526316	-0.33804076431578950	-2.2276500000894734
C	1.7236931889473683	-1.2669737543157895	0.76323653991052631
O	3.1571802889473686	-1.3883117543157895	0.57819866991052637
H	-3.1735786110526316	1.5254808456842106	-1.8088769000894738
H	-0.79484801105263136	-1.5819849543157893	0.21455870991052634
H	1.4228893789473687	-0.73971215431578941	1.6831861999105262
H	1.3532592989473686	-2.3078691543157897	0.73479609991052641
H	3.4974095889473684	-0.51154365431578941	0.40819399991052630
H	1.2071835989473687	-1.1142100543157893	-1.2630885000894738
H	1.4259620789473686	1.4398759456842105	0.17573031991052634
O	0.12829277894736857	-2.4042177543157894	-3.2283859000894735
H	-0.15111005105263142	-2.1717953543157895	-4.1264396000894736
H	-0.51843491105263151	-1.7966607543157895	-2.8003038000894733
O	0.62115455894736860	0.80899486568421053	-3.8594195000894733
H	8.7318389473685087E-003	0.41432339568421050	-3.2327951000894735
H	0.32328279894736855	0.51752712568421044	-4.7577318000894735
O	-2.8637785110526317	-2.8074810543157898	-2.0959240000894734
H	-2.4059517110526314	-1.9915401543157893	-2.3996771000894737
H	-3.2270545110526316	-2.3965652543157896	-1.2739720000894736
O	-2.3490383110526314	1.7105523456842107	-4.0086660000894740
H	-1.9041397110526317	1.8380754456842106	-4.8493993000894733
H	-1.7226738110526316	1.0790467556842105	-3.5696847000894736
O	-3.5348830110526316	-1.3315689543157894	-4.6408196000894737
H	-3.4115021110526316	-2.2911507543157899	-4.5454829000894739
H	-2.6403117110526315	-0.94371575431578947	-4.3500787000894734
O	0.45496424894736853	2.9468691456842104	-2.1294803000894733
H	0.47454651194736852	2.0762926456842106	-1.7175408000894739
H	0.81622903894736853	2.7042662456842104	-3.0554623000894736

C	-1.4001087047368423	3.6919683163157968E-002	-0.94346350199999995
C	-2.2242516047368421	0.99131754316315801	-0.32005332200000003
C	-1.6095098047368421	1.1756345931631580	1.0364410880000001
O	-0.56463630473684234	0.34824958306315795	1.2214529980000000
C	-0.41881582473684220	-0.49782870683684205	0.11905655599999998
O	-3.1897274047368422	1.7575401831631579	-0.91171771200000007
O	-1.8957040047368423	1.9866145831631579	1.9161876979999999
C	1.0420949252631577	-0.44607740683684199	-0.39425171200000003
O	1.4278469352631578	0.90570948316315802	-0.62775547200000004
O	-1.3550133047368422	-0.36166276683684201	-2.1553831019999996
C	1.9404616952631577	-1.1894257168368421	0.71086284799999999
O	3.2634906952631577	-1.3369040168368420	0.30089956800000001
H	-3.8038326047368418	1.1286917631631579	-1.3914928020000001
H	-0.59235750473684234	-1.5551586168368421	0.43551493799999996
H	1.9583946952631577	-0.56426020683684208	1.5110376980000000
H	1.5156909352631578	-2.2018404168368422	0.88224851799999993
H	3.6033303952631579	-0.60414412683684193	-0.20082996200000003
H	1.0689345852631578	-1.0096451168368421	-1.3753965020000001

H	1.2337122052631577	1.4362696831631578	0.18664217799999999
O	-9.1085804736842246E-002	-2.5395237168368423	-3.0806888019999996
H	3.1364505263157705E-002	-2.2735565168368419	-3.9644327019999999
H	-0.56233010473684231	-1.7840959168368422	-2.7028354019999998
O	-2.4214176047368423	1.5477383831631579	-3.8491303019999998
H	-1.8488453047368423	1.4085128831631579	-4.6234883020000002
H	-2.2006869047368420	0.76144649316315793	-3.3235691020000000
O	0.57663300926315775	0.90384839316315801	-3.7687887019999997
H	-0.16482229473684229	0.42137775716315795	-3.3390838019999998
H	0.20102442526315778	1.3468493831631578	-4.5514735019999994
O	-2.8888939047368423	-2.9147579168368423	-1.9162150019999999
H	-2.4235485047368424	-2.1797956168368420	-2.2818060019999997
H	-3.0424221047368420	-2.5106664168368420	-1.0411604020000000
O	0.37582841526315774	3.2740807831631580	-2.1093382020000000
H	0.43789952526315779	2.3044917831631579	-2.1822117020000000
H	-0.26474388473684229	3.4804784831631581	-2.8281843019999999
O	-1.2113414047368423	-0.95672351683684220	-5.3109929019999997
H	-0.79358950473684220	-1.5473185168368422	-5.9222019019999994
H	-1.1115689047368422	-0.14352506683684202	-5.8339502019999996

C	-1.4824569615789476	4.6870000526315875E-002	-0.95116321756842104
C	-2.2464228615789472	0.96933015052631588	-0.22434232756842107
C	-1.6596036615789473	1.3204211805263160	1.0184100524315789
O	-0.66932836157894748	0.39140358052631585	1.2290474124315789
C	-0.41600997157894748	-0.45931631947368412	-5.5379425684210504E-003
O	-3.4918234615789472	1.4461735805263158	-0.60053338756842112
O	-1.9452249615789472	2.1770208805263156	1.8386086124315790
C	1.0334981884210523	-0.42074116947368412	-0.34179537756842104
O	1.5497570384210526	0.87757335052631591	-0.62246733756842110
O	-1.6339823615789477	-0.37109295947368415	-2.1876638875684207
C	1.9753582384210526	-1.1820806194736841	0.70220483243157894
O	3.2079398384210527	-1.5258355194736841	4.1223091231578947E-002
H	-3.6607024615789472	1.0625733305263159	-1.4359808875684210
H	-0.55079156157894749	-1.4867587194736842	0.36067982243157892
H	2.1986272384210523	-0.55018546947368407	1.5794200124315789
H	1.5063196384210524	-2.0683796194736841	0.96364192243157887
H	3.7350331384210524	-0.73667051947368423	-0.26072141756842104
H	1.1783657684210525	-0.95612351947368412	-1.2822335875684210
H	1.3714475384210525	1.4658183805263159	0.17920361243157895
O	-0.18218477157894752	-2.3421008194736839	-3.1651389875684210
H	-0.53508863157894748	-2.1274718194736844	-4.0359590875684210
H	-0.89263706157894751	-1.8283191194736841	-2.6456129875684207
O	-2.5872151615789476	1.6279001805263158	-3.7845571875684207
H	-2.0168110615789474	1.7026107805263160	-4.5907784875684206
H	-2.1853877615789474	0.99941766052631587	-3.1980930875684210
O	0.50491850842105246	0.77853299052631586	-3.5506239875684207
H	-0.22710808157894746	0.26369815752631587	-3.1247649875684207
H	9.2345188421052493E-002	1.3404182805263158	-4.2053494875684212
O	-2.9862099615789472	-3.2162149194736838	-1.9642845875684209
H	-2.6585414615789476	-2.2845103194736840	-2.0898480875684209
H	-3.2943762615789476	-3.1401646194736843	-1.0150053875684211
O	-1.2947689615789475	-1.0454561194736842	-5.1844444875684212
H	-1.2513599615789475	-1.7434150194736839	-5.8701680875684206
H	-0.95864876157894752	-0.32804064947368416	-5.6842711875684211
O	-5.1657102615789476	-0.49568359947368412	-2.6582052875684208
H	-4.6523394615789471	-1.0718526194736842	-3.1609909875684208
H	-5.1352221615789473	0.31440068952631589	-3.1523476875684207

C	-1.5244134521052630	9.5153142105263433E-003	-0.93796224336842104
C	-2.1972198521052633	0.95921516421052644	-0.22691807336842104
C	-1.6343249521052632	1.1547590642105263	1.0510916366315790
O	-0.48587316210526316	0.37008181421052633	1.1743962366315790
C	-0.40347319210526317	-0.46378940578947364	-3.9313569368421029E-002
O	-3.3918994521052634	1.5816341742105262	-0.53141665336842103
O	-1.9646871521052629	1.8930762742105263	2.0086022366315790
C	1.0827191378947367	-0.37344260578947369	-0.52188035336842109
O	1.4756790478947368	1.0175893242105263	-0.64207639336842104
O	-1.7258355521052631	-0.44991341578947369	-2.1063599633684214
C	1.9474905478947369	-1.1027015257894737	0.55617837663157899
O	3.2587225478947368	-1.3890124257894738	0.10052194663157897
H	-3.8154389521052634	1.0545912742105263	-1.2849161633684210
H	-0.52428059210526312	-1.5246843257894738	0.15792178663157896
H	1.9303561478947369	-0.59212084578947355	1.5471288366315790
H	1.5690673478947368	-2.1230309257894735	0.76653200663157894
H	3.8483671478947366	-0.65135197578947368	0.16515334663157896
H	1.2189188478947368	-0.84536752578947372	-1.4878465633684210

H	1.3361255378947368	1.4749525742105263	0.25116356663157896	H	1.2737891800000001	1.4934976236315789	0.28892843999999995
O	-0.24279618210526316	-2.3643962257894735	-3.0559839633684214	O	0.59988781000000002	0.56305432363157881	-3.7661700099999997
H	-0.35168135210526313	-2.0818654257894735	-3.9794974633684213	H	-3.9667580000000036E-002	-6.6385656368421198E-002	-3.3095827099999999
H	-0.77614495210526302	-1.6766435257894736	-2.5964203633684213	H	0.12266580999999999	0.70594083363157889	-4.5247823100000000
O	-2.4842453521052632	1.4849263742105263	-3.8924964633684214	O	-3.1512232800000000	-2.5341607763684211	-2.1741256099999999
H	-1.8949776521052633	1.4061780742105263	-4.6827041633684212	H	-2.4602188799999998	-1.9376522763684210	-2.0085308099999999
H	-2.3094038521052633	0.65162262421052630	-3.3801898633684213	H	-3.5799354800000001	-2.3592079763684213	-1.3106992099999999
O	-3.1346058521052633	-2.8101066257894738	-2.1419912633684213	O	0.11793712000000001	-2.5303195763684210	-3.4068668099999999
H	-2.6201304521052635	-2.0263245257894735	-2.4031568633684213	H	-0.17900560999999998	-2.1002971763684211	-4.2802596099999999
H	-3.5771521521052634	-2.4712935257894739	-1.3279523633684209	H	-0.35316640999999999	-1.9514669763684211	-2.7505496099999998
O	0.28860373189473681	0.63537439421052633	-3.7891130633684211	O	-2.7740391799999999	1.8601808236315789	-3.7116692100000002
H	-0.44070437210526320	0.20397925521052634	-3.3035251633684211	H	-2.2495035799999998	1.8883502236315788	-4.6417947100000001
H	-2.3975721052632015E-003	0.68655191421052630	-4.7536008633684208	H	-2.6440828800000000	0.92190812363157892	-3.4141911100000000
O	-5.3034168521052631	-0.84220892578947382	-2.7596392633684212	O	-4.9926582800000006	-7.6209256368421185E-002	-2.8397407100000001
H	-4.5409067521052631	-1.0757519257894737	-3.2942562633684211	H	-4.2420252800000000	-0.13709071636842113	-3.4386355100000001
H	-5.7200768521052634	-0.13552491578947368	-3.2442488633684210	H	-5.6409545800000007	0.42841343863157882	-3.3686987099999999
O	-3.6680948521052636	-1.4920592257894738	-4.8626711633684208	O	-3.2524612799999999	-0.82514267636842109	-5.0203775100000003
H	-4.0477787521052635	-2.2934231257894737	-5.2218951633684210	H	-3.4561719799999997	-1.8011101763684212	-4.9978959099999996
H	-2.7244513521052633	-1.6594491257894737	-4.9074535633684215	H	-2.3378661799999998	-0.95135467636842108	-5.1919958099999999

C	-1.3993532589473685	-3.2218382315789440E-002	-0.96247103242105259	C	-1.4136113189473685	0.10466334052631576	-0.92349786684210522
C	-2.1323112589473681	0.90550731768421056	-0.24902840242105262	C	-2.0990740189473680	1.1078217605263156	-0.33865113684210529
C	-1.5077227589473685	1.2845890576842105	0.98650359757894734	C	-1.4931861189473685	1.4254693405263157	0.95671622315789473
O	-0.59953321894736855	0.26494805168421054	1.2512799975789475	O	-0.58402541894736837	0.46513897052631575	1.2480325231578948
C	-0.44887337894736840	-0.58133705231578947	8.1912817578947347E-002	C	-0.48894153894736841	-0.43252520947368422	7.9662243157894677E-002
O	-3.2280871589473681	1.5825813276842104	-0.78066711242105258	O	-3.1725701189473683	1.7925042405263159	-0.88238995684210531
O	-1.6290627589473685	2.2729517276842106	1.7339542975789475	O	-1.6372213189473686	2.3299213405263157	1.7464619231578948
C	1.0743099210526315	-0.49942797231578950	-0.32896137242105267	C	0.98618877105263159	-0.51080972947368419	-0.36945470684210530
O	1.2552553010526315	0.89675797768421051	-0.56236453242105267	O	1.6273970810526315	0.76411696052631584	-0.40489063684210530
O	-1.5385882589473685	-0.38152885231578948	-2.1977985024210529	O	-1.5072527189473683	-0.41680716947368424	-2.0858078768421056
C	1.9906381410526315	-1.0743941723157895	0.68387309757894732	C	1.7980252810526316	-1.3977057594736841	0.60689297315789470
O	3.2517436410526317	-1.3336188723157893	7.1445897578947357E-002	O	3.2263029810526316	-1.4543825594736841	0.16725482315789469
H	-3.7525043589473679	0.97910317768421051	-1.2659941024210526	H	-3.6661651189473679	1.1812173705263156	-1.4156066768421052
H	-0.74784155894736859	-1.6168743723157895	0.32163750757894738	H	-0.84945181894736832	-1.3559451594736842	0.50492704315789472
H	2.1174484410526317	-0.32122885231578946	1.4883436975789475	H	1.8844993810526316	-0.90571795947368428	1.6270683231578946
H	1.5776012410526314	-2.0914912723157895	0.88230752757894737	H	1.3925679810526317	-2.4232438594736840	0.67622375315789474
H	3.5672535410526316	-0.46420588231578941	-0.14145944642105265	H	3.5380366810526316	-0.58768723947368429	-0.27657599684210532
H	1.2451058110526316	-1.0430427723157896	-1.2629249024210527	H	0.94736567105263170	-0.78239365947368433	-1.4177762768421052
H	0.90452193105263157	1.2529298176842105	0.25041096757894737	H	1.5111156810526316	1.0963649805263156	0.50141130315789473
O	0.48077539105263156	0.58431146768421049	-3.8505619024210529	O	1.9616291052631629E-002	-2.2429850594736842	-3.4193421768421053
H	-0.27496701894736847	0.34508682668421053	-3.2423022024210528	H	2.6728861052631614E-002	-1.8290503594736842	-4.2988688768421053
H	-0.14582397894736843	0.70179724768421048	-4.6479728024210525	H	-0.45666495894736842	-1.5500273594736842	-3.0076251768421054
O	3.8898021052631571E-002	-2.4237340723157894	-3.1009740024210530	O	-3.1448992189473683	-2.7052703594736842	-2.3609702768421053
H	4.1460141052631572E-002	-2.0634626723157896	-4.0025747024210530	H	-2.5706061189473681	-1.8829597594736840	-2.4426352768421054
H	-0.48906683894736841	-1.7812113723157896	-2.6857084024210529	H	-3.2452950189473682	-2.7834079594736840	-1.3969415768421052
O	-3.1568557589473683	-2.7378673723157894	-2.1630177024210528	O	0.55426703005263167	0.64451977052631571	-3.7211190768421054
H	-2.7832430589473685	-1.8677624723157895	-2.3699812024210529	H	-0.22181913894736832	0.27298332452631574	-3.2402387768421055
H	-3.3865724589473682	-2.6161961723157896	-1.2273188024210526	H	0.12465680105263161	0.82998481052631568	-4.5727626768421050
O	-2.7499137589473683	1.6649578276842105	-3.9081126024210531	O	-2.5991960189473682	1.7975922405263156	-3.8370264768421052
H	-2.1499280589473684	1.3716150276842105	-4.6325753024210528	H	-2.1326005189473682	1.6983960405263159	-4.7101471768421055
H	-2.4074572589473684	1.1221577376842107	-3.1854510024210527	H	-2.2202288189473682	1.0921128405263159	-3.2862082768421055
O	-0.24489743894736843	2.9156160276842105	-2.1522680024210530	O	6.4947361052631603E-002	-3.5109410594736841	-0.86623766684210524
H	-4.6565368947368457E-002	2.0747990276842105	-1.7076285024210527	H	0.97518965105263167	-3.8053098594736841	-0.83933637684210527
H	0.15266389105263156	2.7345145276842104	-2.9860416024210528	H	-8.5119988947368330E-002	-3.0392643594736843	-1.7605558768421052
O	-3.5019002589473684	-1.1000258723157894	-4.8366309024210521	O	-5.1435208189473682	-0.27474056947368419	-2.7958253768421053
H	-3.4831052589473681	-1.8189791723157893	-5.5229245024210520	H	-4.3754766189473679	-0.80992715947368421	-3.0233132768421052
H	-2.5785166589473683	-0.91256247231578946	-4.9822419024210527	H	-5.3963805189473684	6.4064730526315727E-002	-3.6149060768421051

C	-1.3500955800000001	4.0275213631578821E-002	-0.88663377000000010	C	-1.5388431910526317	0.12520081473684208	-0.82864690052631573
C	-2.1700248799999997	0.98977071363157876	-0.29370228000000004	C	-2.0390722910526318	1.2565390247368420	-0.26768481052631576
C	-1.6593258800000001	1.2425998936315787	1.0003272900000000	C	-1.4274339910526317	1.4622657147368421	1.0057436494736842
O	-0.60113768000000012	0.41154180463157880	1.3093865900000001	O	-0.47025648105263163	0.44370899473684211	1.2424658494736842
C	-0.39944604000000000	-0.46403277636842116	0.17541126999999998	C	-0.48558901105263164	-0.43816187526315797	0.10740556947368421
O	-3.2433211799999997	1.7017876236315790	-0.81841339000000002	O	-3.1012823910526315	2.0422767147368424	-0.66264017052631574
O	-2.1069006799999999	1.9836856236315790	1.8882983900000001	O	-1.5688239910526318	2.3677285147368421	1.8480063494736840
C	1.0467598700000000	-0.43932179636842122	-0.32295990000000002	C	0.96699675894736836	-0.61147969526315793	-0.47814363052631581
O	1.4056196699999999	0.96377996363157892	-0.53683728000000008	O	1.6020133089473685	0.69626618473684210	-0.63806701052631576
O	-1.4108140800000000	-0.36186666636842119	-2.1284443099999999	O	-1.7213538910526316	-0.50287738526315795	-1.9739323505263158
C	1.9835624199999999	-1.2922845763684212	0.54294260999999999	C	1.8796608089473683	-1.3871271852631579	0.52831448947368420
O	3.3212650200000002	-1.3589774763684210	2.9925899999999617E-003	O	3.2276124089473681	-1.6061249852631578	7.0486489473684216E-002
H	-3.5994889799999998	1.2067184636315789	-1.5718773099999999	H	-3.8238738910526315	1.4648495147368421	-1.0698145505263159
H	-0.64900778000000003	-1.4898955763684212	0.45805670999999992	H	-0.84156189105263168	-1.4005037852631579	0.55886868947368429
H	1.9123423199999998	-0.96421177636842104	1.5509584900000000	H	2.0093004089473681	-0.90802218526315781	1.5009760494736841
H	1.5075260200000000	-2.3229864763684209	0.57045814999999989	H	1.4774068489473684	-2.4041346852631582	0.67521223947368425
H	3.7458773200000000	-0.48908922636842117	0.14437111999999996	H	3.4191365089473686	-0.74582028526315791	-0.42034690052631574
H	0.99282093999999987	-0.85099057636842113	-1.3732634099999999	H	0.86652445894736840	-1.1504221852631580	-1.3862200505263158

H	1.5694395089473683	1.2958387747368421	0.18801699947368419	H	1.4425415905263159	1.0752227492105264	0.73487020626315791
O	-3.0371875910526320	-2.9580648852631581	-2.4666562505263157	O	-2.7597760094736841	1.6970824892105263	-3.7725820137368422
H	-2.3420981910526315	-2.2494337852631583	-2.3587796505263157	H	-2.0327896094736837	1.8696764892105264	-4.4530158137368421
H	-3.4775349910526314	-2.7131467852631577	-1.6583262505263159	H	-2.3192004094736838	0.97217767921052634	-3.2984267137368422
O	2.4438248947368402E-002	-2.1848778852631581	-3.4682538505263159	O	-2.3256189473684075E-002	-2.0211254107894741	-3.4191508137368420
H	-0.11794032105263164	-1.7655843852631581	-4.4164229505263153	H	-0.49170712947368406	-1.8993297107894735	-4.2431686137368425
H	-0.72758219105263167	-1.8415506852631578	-2.8768870505263155	H	-0.54430666947368400	-1.3890837107894736	-2.9085934137368423
O	-2.3095082910526319	1.7667408147368420	-3.5994735505263158	O	-2.9475587094736837	-2.9699816107894739	-2.4210432137368421
H	-1.8024759910526316	1.6888472147368421	-4.4015043505263156	H	-2.3293844094736840	-2.1796723107894738	-2.5252178137368420
H	-2.2932417910526315	0.89932594473684213	-3.2049428505263156	H	-3.0760821094736839	-2.8059290107894737	-1.5060500137368422
O	0.78788917894736832	0.57761904473684211	-3.9014582505263156	O	0.97328123052631588	0.85887239921052627	-3.7247474137368419
H	0.50042239584736836	-0.31858553526315792	-3.6023199505263155	H	0.48714554052631592	2.6144919210526307E-002	-3.5410018137368420
H	0.16537103894736838	0.71471743473684213	-4.6599803505263155	H	0.49743186052631594	1.1101935692105263	-4.5193420137368419
O	-5.4688119910526316	-0.32433022526315791	-2.8558466505263156	O	-5.2252575094736837	-0.91592621078947367	-2.7775142137368420
H	-4.5100186910526316	-0.24220199526315789	-2.7355744505263155	H	-4.4114014094736840	-1.4722991107894736	-2.6547280137368423
H	-5.6961264910526310	0.23419916573684207	-3.6079528505263156	H	-5.0212573094736843	-0.51084186078947358	-3.6361849137368423
O	-3.3515961910526313	-0.70459248526315799	-5.0002542505263152	O	-3.5429497094736839	-0.81261211078947371	-4.9661728137368417
H	-3.9576019910526314	-1.4114337852631580	-5.4319188505263156	H	-3.9547683094736836	-0.78641701078947368	-5.8182399137368419
H	-2.4026291910526316	-0.92919808526315784	-5.1623605505263157	H	-2.6071619094736840	-0.98549111078947371	-5.1115743137368419
C	-1.5187912257894738	5.1443177368421023E-002	-0.83954042784210525	C	-1.3370260794736841	2.1665593157894764E-002	-0.91316299684210522
C	-2.1169179257894735	1.1683395173684210	-0.20218312084210521	C	-2.0572560794736843	1.0920145531578946	-0.29681983684210522
C	-1.4915808257894738	1.4142601773684209	1.0723130521578947	C	-1.6325521794736841	1.3625753431578946	0.98446136315789479
O	-0.47946649578947370	0.47197027736842101	1.2599511521578948	O	-0.54204568947368414	0.53805001315789480	1.2373541631578946
C	-0.40110798578947376	-0.40779951263157899	0.14640161215789477	C	-0.41132700947368417	-0.40696510684210518	0.14981060315789474
O	-3.1770360257894734	1.9056252773684212	-0.57170437784210526	O	-3.3086164794736845	1.7032183431578947	-0.61910024684210530
O	-1.7619661257894739	2.2361645773684211	1.9242026521578948	O	-1.9867362794736840	2.2164871431578947	1.7757946631578947
C	1.0463137442105264	-0.46971834263157897	-0.41972942784210521	C	1.0356064705263157	-0.40032631684210518	-0.35927563684210528
O	1.6646212742105262	0.77741979736842104	-0.61303667784210525	O	1.5601101205263159	0.93556051315789479	-0.47735181684210526
O	-1.9025110257894740	-0.44384124263157898	-1.9983177478421053	O	-1.5486996794736843	-0.52681731684210520	-2.0839705368421053
C	1.8935234742105262	-1.3895586226315788	0.47898015215789480	C	1.8730202205263158	-1.3086379568421054	0.57554526315789478
O	3.1135656742105264	-1.6588415226315791	-8.0295175842105215E-002	O	3.1357284205263163	-1.5932092568421052	2.5454293157894742E-002
H	-3.6758215257894733	1.3971021773684211	-1.2111203478421053	H	-3.5738629794736845	1.3220824431578946	-1.4874209368421054
H	-0.72551072578947373	-1.3991720226315789	0.47366211215789478	H	-0.56727693947368418	-1.4898372568421052	0.51075533315789479
H	1.9190017742105263	-0.95459712263157892	1.4871016521578948	H	1.8192211205263158	-0.89566575684210525	1.6387725631578949
H	1.4029853742105263	-2.3743858226315790	0.64079046215789481	H	1.3821258205263158	-2.3023847568421054	0.62557008315789475
H	3.3744929742105265	-0.76954752263157900	-0.31826676784210522	H	3.4696560205263163	-0.73530630684210518	-0.30987280684210528
H	0.97279732421052634	-0.87814652263157889	-1.4285076478421053	H	1.0030223805263159	-0.85040715684210511	-1.3771647368421052
H	1.8634082742105262	1.3232832773684211	0.19929887215789477	H	1.6869088205263159	1.3179032431578948	0.40062122315789472
O	-0.20986653578947373	-1.8549156226315788	-3.6050368478421051	O	-2.6708437794736843	1.1770290131578947	-3.8745261368421056
H	-0.55787031578947377	-1.7317865226315790	-4.5470203478421052	H	-1.8399453794736840	1.2411490431578946	-4.3549635368421056
H	-0.88204782578947372	-1.3823557226315790	-3.0389161478421052	H	-2.4483542794736843	0.52355060315789481	-3.1882063368421054
O	-3.1572718257894734	-3.0349144226315787	-2.2085255478421049	O	-0.19088074947368416	-2.5114546568421052	-3.7279591368421054
H	-2.6459273257894735	-2.1834870226315788	-2.2060609478421052	H	-0.35825405947368416	-2.1717481568421055	-4.6101009368421053
H	-3.5587427257894735	-2.9838397226315787	-1.3253294478421052	H	-0.66542731947368416	-1.9079824568421055	-3.1277472368421053
O	-2.5820368257894737	1.8795441773684209	-3.6399328478421049	O	-3.1665493794736843	-2.9454122568421055	-2.2066092368421053
H	-2.0706324257894737	1.5531221773684210	-4.3874350478421054	H	-2.5915027794736840	-2.1520542568421055	-2.3973609368421052
H	-2.5825058257894735	1.0954444273684212	-3.0460074478421051	H	-3.1583016794736842	-2.9398537568421053	-1.2495149368421052
O	-5.4080910257894734	-0.46757184263157892	-2.9920469478421050	O	1.0582651605263158	0.84247803157894782	-3.9112904368421053
H	-4.5691365257894736	-0.67396691263157893	-3.4118523478421050	H	0.51549660052631585	0.10858301215789476	-3.5731167368421053
H	-5.5855856257894736	0.38297664936842102	-3.3733500478421048	H	0.85931865052631584	0.81374407315789477	-4.8609306368421050
O	-0.11884942578947372	-3.3890705226315788	-1.2964327478421052	O	-8.6715899473684133E-002	-3.3361471568421055	-1.1287646368421052
H	0.52352666421052629	-4.1721107226315794	-1.3783537478421053	H	0.66866345052631582	-3.9320562568421056	-1.1752291368421051
H	-0.16989080578947374	-2.9935486226315788	-2.1393460478421051	H	-0.19858181947368414	-2.9476899568421051	-2.0438364368421054
O	0.95663138421052629	1.0293204473684210	-4.1747063478421049	O	-5.1590478794736843	-1.0635957568421053	-2.7211213368421054
H	0.62997283421052619	0.16877412736842104	-3.8980123478421049	H	-4.3706893794736841	-1.5152105568421053	-2.5696786368421054
H	0.50119229421052625	1.2151615273684211	-5.0425541478421048	H	-4.9529687794736841	-0.96526765684210514	-3.6902518368421053
C	-1.4674478094736840	0.18354992421052635	-0.94971719373684216	C	-1.3866269131578948	9.4478664526315759E-002	-1.0332994626842105
C	-2.0578432094736838	1.1985701192105263	-0.26933167373684208	C	-2.1055671131578948	1.0914742025263158	-0.45248340268421050
C	-1.4594371094736842	1.4535204892105265	0.96289143626315787	C	-1.5843942131578947	1.2726811325263159	0.90732591731578949
O	-0.50114725947368410	0.46160226921052633	1.1636762862631578	O	-0.63172647315789465	0.27956515252631575	1.2057489173157896
C	-0.44127974947368404	-0.36888694078947365	1.9298042263157907E-002	C	-0.44130184315789467	-0.48761972747368421	-8.9903426842105338E-003
O	-3.1953792094736837	1.8355136892105264	-0.64133392373684217	O	-3.2690402131578948	1.7190242325263159	-0.83633866268421053
O	-1.6115952094736841	2.3825451892105263	1.7370126862631579	O	-2.0106856131578947	2.1230840325263158	1.7669051173157895
C	1.0464108005263160	-0.46972447078947371	-0.40371168373684208	C	1.0883635968421053	-0.40687010747368424	-0.33314895268421052
O	1.6757105905263159	0.82469146921052627	-0.17399521373684207	O	1.5320902868421054	0.93760879252631579	-0.17657588168421054
O	-1.7742640094736839	-0.23592920078947371	-2.1347266137368419	O	-1.6549137131578948	-0.43955458747368426	-2.2358325826842109
C	1.7559745905263160	-1.5092245107894735	0.49262169626315794	C	1.8814372868421052	-1.2794706674736842	0.64552697731578945
O	3.0908342905263160	-1.8162008107894738	4.7088900263157908E-002	O	3.2522590868421051	-1.4065823674736841	0.24743597731578945
H	-3.5100172094736837	1.5101699892105265	-1.5475663137368421	H	-3.5042487131578945	1.3505420325263158	-1.7132063826842105
H	-0.66381510947368416	-1.3304604107894735	0.41801972626315792	H	-0.68905263315789467	-1.5583761674736842	0.17444953731578947
H	1.8026009905263158	-1.1068468107894736	1.5952625862631578	H	1.7860475868421053	-0.94639456747368433	1.6065658173157895
H	1.1482075505263158	-2.3991593107894738	0.48845381626315793	H	1.5140214868421054	-2.3402927674736840	0.57106944731578946
H	3.5792850905263163	-0.96158101078947356	-7.5128352736842097E-002	H	3.5793333868421051	-0.50190866747368423	0.29562885731578947
H	1.1406603905263160	-0.72737241078947368	-1.4636844137368421	H	1.2106990368421053	-0.69469554747368423	-1.3902396826842105

H	1.4333056868421052	1.1933069325263159	0.75945878731578953	H	1.3813925521052630	1.1706261010526315	0.73362642236842102
O	-2.5279455131578947	1.4730932325263157	-3.8930115826842107	O	-0.64052166789473697	-2.3937392689473684	-3.7546346876315786
H	-1.8301427131578949	1.6238225325263158	-4.6283432826842104	H	-0.69381449789473693	-1.8680006689473685	-4.5903147876315789
H	-2.1918400131578948	0.62986687252631579	-3.4075553826842109	H	-1.0498186478947371	-1.7520140689473684	-3.0976095876315788
O	0.57116044684210532	0.47705958252631575	-3.7585308826842105	O	0.67397857210526313	0.13314332305263157	-3.7837932876315787
H	-0.15626870315789468	0.11698301052631577	-3.1920283826842106	H	3.3935689105263019E-002	-0.12320150894736842	-3.1166400876315787
H	0.31493169784210534	9.0576394526315773E-002	-4.5804305826842109	H	0.39529838210526302	-0.40716376894736844	-4.5525419876315789
O	-2.7689576131578946	-3.0402739674736843	-1.8400632826842105	O	-2.7764773478947369	1.4674424310526317	-4.0651812876315789
H	-2.2638171131578946	-2.2444367674736840	-1.9574149826842104	H	-1.9174083478947370	1.4077678310526316	-4.3024384876315791
H	-2.8704296131578948	-2.9895702674736842	-0.86626334268421057	H	-2.7817890478947369	0.66146412105263153	-3.5766327876315787
O	-0.89229611315789470	-2.6361767674736840	-3.9524655826842108	O	-3.4706339478947368	-3.2250980689473683	-1.9234020876315789
H	-0.86867781315789461	-2.0759198674736843	-4.7132746826842107	H	-2.5095439478947368	-3.0049672689473685	-1.9272071876315791
H	-1.3378984131578948	-2.0984188674736841	-3.2978503826842109	H	-3.7937335478947367	-2.7522015689473682	-1.1311890876315789
O	-5.1337794131578942	-1.4345118674736841	-2.5891243826842105	O	-4.9391240478947376	-0.74822035894736838	-2.7655019876315787
H	-4.1961621131578939	-1.4261727674736842	-2.3540182826842107	H	-4.3151128478947376	-1.4875605689473683	-2.5788723876315789
H	-5.0820977131578946	-1.7560269674736841	-3.5088125826842109	H	-4.4887929478947370	-0.39078122894736839	-3.5605285876315786
O	0.15554523884210533	-3.3043733674736839	-1.3679001826842105	O	-0.24402554789473696	-3.4529950689473683	-1.1567943876315789
H	0.91739688684210541	-3.9157629674736838	-1.4964183826842106	H	0.49133425210526305	-4.1383376689473685	-0.96932649763157896
H	-5.4102153157894656E-002	-3.1257048674736843	-2.2639657826842106	H	0.11953596410526302	-3.1258371689473683	-1.9815792876315790
C	-1.4943707910526316	-2.3983268421052625E-002	-0.93938623578947367	C	-1.4374147978947369	3.0805199473684236E-002	-1.0323278905263158
C	-2.1484179910526313	1.0275487515789474	-0.38183070578947365	C	-2.0829304978947372	1.0882938994736842	-0.38267282052631579
C	-1.6241846910526316	1.3750044015789473	0.90376085421052632	C	-1.6827268978947370	1.2574858394736841	0.99450547947368417
O	-0.71778294105263152	0.32306709157894736	1.3088662542105263	O	-0.48990735789473694	0.41922687947368420	1.1749532794736843
C	-0.51560811105263149	-0.50971678842105261	0.19375251421052633	C	-0.40385285789473696	-0.43769765052631571	3.7851494736842461E-003
O	-3.1707997910526311	1.8011481015789474	-0.89110414578947361	O	-3.3556829978947369	1.5811724394736841	-0.78901405052631579
O	-1.8172704910526316	2.2775283015789474	1.7690152542105262	O	-1.9683848978947369	1.9298580394736842	1.9216012794736845
C	0.97104553894736845	-0.46600081842105268	-0.26344696578947369	C	1.0729545021052631	-0.46164484052631571	-0.37744016052631579
O	1.5592431089473684	0.85606727157894735	-0.27756309578947364	O	1.5899413021052631	0.91383555947368422	-0.31874737052631574
O	-1.5542010910526316	-0.49333645842105267	-2.0931914457894738	O	-1.6652837978947370	-0.41683188052631570	-2.1615851205263157
C	1.8199902089473685	-1.3785501984210526	0.63507383421052632	C	1.8673877021052629	-1.3203270605263158	0.62138145947368417
O	3.2375561089473686	-1.2670573984210527	0.26146177421052630	O	3.2942121021052628	-1.3001014605263159	0.18301116947368426
H	-3.1560234910526312	1.6604410015789473	-1.8984270457894736	H	-3.5734388978947371	1.3064151394736840	-1.6657136205263157
H	-0.63816315105263155	-1.5212256984210526	0.49792686421052634	H	-0.71289931789473693	-1.4513958605263158	0.29026574947368422
H	1.8201244089473685	-1.0025803984210526	1.6769533542105262	H	1.8131732021052631	-0.82772609052631574	1.6720545794736843
H	1.5028939089473685	-2.4323603984210522	0.52528058421052637	H	1.6063323021052631	-2.3657841605263159	0.63598625947368426
H	3.3965652089473686	-0.51668749842105266	-0.30458991578947364	H	3.5282763021052630	-0.39428054052631578	4.6661794736842510E-003
H	1.0749540389473686	-0.91178629842105274	-1.3312614457894738	H	1.3285380021052631	-0.77209289052631569	-1.3395175205263157
H	1.4544500089473684	1.2024803015789474	0.60870971421052633	H	1.2717069021052629	1.2207894394736842	0.56480796947368417
O	0.62109781894736849	0.26114902157894737	-3.4214169457894736	O	-2.6632235978947372	1.5335503394736842	-3.8389857205263156
H	-0.18831202105263153	0.13839948457894738	-2.8270273457894737	H	-2.2076645978947371	1.3545977394736841	-4.6738630205263156
H	0.37431856894736848	-0.25644476842105263	-4.1728491457894741	H	-2.1492622978947371	0.91242707947368429	-3.3336636205263157
O	-3.2593669910526315	-2.9287774984210526	-2.0045223457894736	O	0.62540241210526304	0.53813998947368424	-3.7808533205263157
H	-2.6483380910526315	-2.2142665984210526	-1.8735684457894737	H	-9.9482167894736920E-002	1.4730289473684238E-002	-3.3361229205263156
H	-3.3219306910526312	-3.2393944984210523	-1.1021900257894737	H	0.39488418210526305	0.27506519947368424	-4.7002826205263153
O	-0.86107479105263152	-2.7554946984210522	-3.9143694457894735	O	-1.1901496978947370	-2.5248602605263160	-4.2184217205263153
H	-0.89312799105263152	-2.2608233984210524	-4.7637089457894737	H	-1.0051364978947370	-1.7702439605263158	-4.8174844205263154
H	-1.2491892910526314	-2.0808272984210525	-3.3706271457894736	H	-1.4725214978947370	-2.0993773605263160	-3.4060618205263156
O	-2.8005727910526312	1.3626347015789474	-4.0798206457894732	O	-5.2347281978947366	-0.79759549052631573	-2.8677465205263157
H	-2.1558792910526314	1.2108245015789474	-4.7751646457894736	H	-4.2653939978947362	-0.78426606052631576	-2.8121314205263155
H	-2.5533597910526313	0.57366085157894742	-3.5591000457894739	H	-5.5357445978947366	-0.39564452052631571	-3.7080735205263156
O	-4.8486014910526318	-0.97110799842105266	-2.9631498457894736	O	0.62067044210526301	2.8220532394736844	-2.1958020205263158
H	-4.1708620910526317	-1.5132742984210525	-2.4696450457894739	H	1.1295666021052631	2.0810491394736843	-1.7272373205263156
H	-4.4236012910526314	-0.85012517842105262	-3.8166177457894737	H	0.27070510210526305	2.2449631394736840	-2.9442550205263158
O	9.0925550947368497E-002	-3.5053072984210525	-1.3068153457894738	O	-0.17153252789473691	-3.6220279605263159	-1.6207299205263157
H	0.78006073894736849	-4.1934351984210521	-1.4703860457894737	H	0.24726968210526307	-4.4843587605263160	-1.3959207205263158
H	-5.2974641052631499E-002	-3.1070993984210524	-2.1980623457894737	H	0.15883395210526308	-3.4012179605263158	-2.5586245205263158
C	-1.5007075478947369	3.7475461052631581E-002	-0.95064828763157894	C	-1.5311285784210527	8.6145644242105232E-002	-1.0378331256315789
C	-2.1906755478947368	1.1191403010526315	-0.29529935763157900	C	-2.2241927784210525	1.0067766625421053	-0.32127872563157894
C	-1.6835306478947369	1.3069755310526316	1.0058755123684211	C	-1.6801806784210527	1.1771699425421054	0.97597229436842103
O	-0.56077798789473698	0.49040217105263156	1.2217488123684210	O	-0.62917618842105272	0.30546310254210524	1.0972978743684212
C	-0.48792092789473701	-0.46096536894736845	8.2169492368421054E-002	C	-0.42356888842105272	-0.49725806745789475	-9.4119277631578957E-002
O	-3.3760655478947368	1.7039579310526316	-0.82785368763157896	O	-3.2794577784210523	1.7913897425421053	-0.81147543563157898
O	-1.9120006478947371	2.1162152310526317	1.8888221123684210	O	-1.9478688784210527	1.9670526425421053	1.8641085743684211
C	1.0212452421052631	-0.49607362894736845	-0.29369733763157896	C	1.0943411215789474	-0.49245567745789470	-0.34481998563157895
O	1.5850005521052630	0.79104526105263151	-0.14892163763157895	O	1.6548758215789472	0.85896374254210517	-0.21689104563157896
O	-1.5668223478947370	-0.40161320894736846	-2.1515594876315789	O	-1.6652956784210526	-0.31465010745789473	-2.2520380256315793
C	1.8698608521052629	-1.4909959689473684	0.46608717236842101	C	1.9341182215789472	-1.3997299574578947	0.59879286436842094
O	3.2855782521052630	-1.3561210689473684	9.9107742368421042E-002	O	3.3169277215789474	-1.1999734574578946	0.27118082436842106
H	-3.1096838478947366	2.0062321310526317	-1.7133992876315789	H	-3.3766338784210523	1.5801524425421054	-1.7277727256315789
H	-0.75243460789473704	-1.4806077689473685	0.43919162236842102	H	-0.68076706842105272	-1.5299703574578947	8.0081083368421041E-002
H	1.7516521521052630	-1.3789219689473684	1.5375846123684209	H	1.8108360215789472	-0.93521455745789484	1.5533858743684210
H	1.7273341521052630	-2.5294386689473685	0.17908343236842106	H	1.7181211215789474	-2.4823082574578947	0.61863560436842102
H	3.3760527521052630	-0.42801037894736843	3.2842437368421053E-002	H	3.4086124215789475	-0.28739706745789478	0.44460964436842104
H	1.1425031521052629	-0.71932208894736838	-1.3047602876315789	H	1.2179575215789473	-0.79986125745789483	-1.3612959256315789

H	1.2824804215789474	1.1657048425421053	0.66345963436842104
O	0.69062357157894727	0.38574385254210525	-3.5056691256315791
H	-0.17677267742105271	0.14784208554210523	-3.1339159256315789
H	0.94594212157894719	-0.29447585745789473	-4.0956496256315784
O	-1.7162540784210527	-2.7136361574578949	-4.0041914256315785
H	-1.7241835784210526	-2.1414142574578947	-4.8313652256315791
H	-1.7773452784210528	-2.0046461574578949	-3.3452276256315789
O	-3.0335363784210525	1.5913465425421054	-4.1083591256315790
H	-2.4862538784210524	1.8346647425421052	-4.8735203256315787
H	-2.4588437784210524	0.97801085254210518	-3.5960767256315793
O	-5.0180214784210531	-0.60392131745789479	-2.4963641256315792
H	-4.1370614784210531	-0.59975665745789486	-2.1966807256315790
H	-4.9600111784210528	-0.81782955745789476	-3.5042461256315791
O	0.43231094157894723	2.6225742425421052	-1.9194174256315788
H	1.0312272215789473	1.9901088425421054	-1.5371760256315790
H	0.38846196157894730	2.2634521425421052	-2.8343812256315792
O	-1.2302049784210527	-1.0357592574578947	-6.1370133256315791
H	-0.98891026842105267	-0.81826998745789481	-7.0267725256315785
H	-1.3433987784210528	-0.12576516745789479	-5.7600522256315783

C	-1.4322942178947369	5.6231147789473690E-002	-0.96808718589473675
C	-2.1348431178947367	1.0244065017894737	-0.30805556589473682
C	-1.7005139178947368	1.2313148517894739	1.0203197841052631
O	-0.65264646789473679	0.37161022178947367	1.2741396141052630
C	-0.42648651789473685	-0.42682029821052636	4.5193098105263174E-002
O	-3.1506510178947367	1.7825543517894737	-0.90610505589473678
O	-2.1089878178947368	1.9021495517894738	1.9948626141052630
C	1.0648015821052632	-0.39957015821052633	-0.36202930589473681
O	1.6482633821052632	0.85697167178947364	-2.9193315894736824E-002
O	-1.6605605178947369	-0.33630092821052632	-2.1645001858947368
C	1.9664548821052632	-1.4401133482105264	0.44678105410526320
O	3.3380022821052631	-1.1831919482105264	0.28669114410526320
H	-3.2848923178947369	1.3653720517894739	-1.7697424858947368
H	-0.74298672789473685	-1.4819381482105265	0.20602034410526315
H	1.7031600821052633	-1.3840133482105261	1.5031935141052630
H	1.7261177821052631	-2.4014599482105261	4.5265802105263174E-002
H	3.4697433821052632	-0.17534499821052629	0.36607544410526316
H	1.2860709821052632	-0.50975447821052633	-1.4320877858947370
H	1.0922482821052633	1.1478972517894737	0.75125847410526314
O	0.46663688210526316	0.54395050178947368	-3.6707004858947370
H	-0.11741165789473684	1.9687539789473685E-002	-3.0380404858947370
H	0.31217930210526312	0.16249282578947369	-4.5496224858947363
O	-1.4072885178947367	-2.4920228482105262	-3.9915601858947372
H	-1.4319977178947367	-2.1051464482105260	-4.8746933858947363
H	-1.6902867178947369	-1.8087847482105261	-3.4199934858947367
O	-2.7902696178947366	1.4438510517894736	-4.1041070858947366
H	-2.1311219178947369	1.6628124517894736	-4.7651121858947363
H	-2.5775824178947366	0.52250632178947365	-3.8653148858947368
O	-4.9072775178947365	-1.0849361482105264	-2.6698748858947368
H	-3.9031511178947369	-1.0860977482105265	-2.4740284858947370
H	-5.0585228178947368	-1.1176825482105262	-3.5921714858947369
O	0.50206359210526319	2.7997410517894736	-1.7395815858947370
H	1.0788017821052631	2.4056816517894739	-1.0756200858947369
H	0.46532179210526314	2.1397225517894740	-2.4902864858947371
O	-0.20924483789473683	-3.6076693482105262	-1.2471234858947369
H	0.55887270210526319	-4.2277754482105268	-1.3104300858947369
H	-0.19526729789473685	-3.2802637482105261	-2.1797732858947367

C	-1.3186388326315790	2.4814632631578987E-002	-1.0863947410526316
C	-2.1165844326315790	0.93402711263157900	-0.47448830105263151
C	-1.6650688326315790	1.1543165926315790	0.89137689894736849
O	-0.62308616263157912	0.38666869263157899	1.2109432589473683
C	-0.36768063263157907	-0.41107311736842100	-1.1387921052631561E-002
O	-3.2364789326315790	1.5794514526315790	-0.84919244105263159
O	-2.1306510326315791	1.8970427526315792	1.7551747589473683
C	1.0563838673684209	-0.27542991736842104	-0.37123502105263151
O	1.5553981673684210	1.0451504726315790	-4.7025331052631569E-002
O	-1.3015590326315789	-0.65940748736842103	-2.1538978410526313
C	1.8309031673684211	-1.3284460473684212	0.48873398894736841
O	3.2465659673684208	-1.1117764473684209	0.49607984894736845
H	-3.5017710326315790	1.1946765526315790	-1.7065619410526316
H	-0.58147468263157909	-1.4021211473684212	0.29305755894736840
H	1.5331338673684209	-1.3416800473684209	1.5586953589473684
H	1.7888737673684210	-2.3597703473684213	6.2940418947368432E-002
H	3.3808295673684210	-0.17037131736842104	0.45823739894736848
H	1.2931432673684211	-0.36083153736842100	-1.3761824410526318

H	1.1577619673684210	1.2047591526315791	0.86112648894736843
O	-1.5088715326315789	-2.7456747473684211	-3.9352405410526310
H	-1.1053253326315791	-2.1319310473684210	-4.6141414410526318
H	-1.6297728326315790	-2.0311023473684213	-3.2006577410526313
O	0.72701984736842096	0.60555577263157900	-3.7025266410526316
H	-2.3639661315790066E-002	9.2087836631578990E-002	-3.3666159410526313
H	1.0227812673684211	-5.8509897368421021E-002	-4.3100882410526316
O	-2.6808738326315793	1.7527721526315792	-3.8682726410526311
H	-2.2709168326315794	1.7637510526315792	-4.7404338410526314
H	-2.2476488326315791	0.99939868263157894	-3.4058273410526314
O	-0.39848839263157909	-3.4907700473684211	-1.3389688410526317
H	0.42450968736842093	-4.0538680473684208	-1.3373690410526315
H	-0.64870183263157910	-3.3173501473684213	-2.2373371410526315
O	-3.7472067326315792	-3.1340510473684211	-1.8245026410526315
H	-2.8290015326315792	-2.9395753473684212	-1.5825929410526316
H	-4.1507489326315792	-2.8460820473684212	-0.97927684105263146
O	-4.7245474326315788	-0.92974264736842105	-2.9642153410526313
H	-4.2085175326315793	-1.5720311473684210	-2.5845242410526312
H	-4.6318742326315796	-1.1376879473684212	-3.8991896410526317

C	-1.4339734531578947	3.1408913894736545E-003	-0.98872286363157902
C	-2.1661480531578952	0.97112605138947372	-0.36158687363157893
C	-1.7004154531578948	1.1409092413894737	0.98283028636842107
O	-0.57256768315789486	0.30314926498947364	1.2112230363684211
C	-0.35116567315789482	-0.47599823861052637	1.6962423368421034E-002
O	-3.2748648531578950	1.5816637113894736	-0.88198314363157893
O	-2.1707565531578950	1.7718803113894737	1.9458292363684211
C	1.1101974468421052	-0.35802290861052632	-0.37866061363157894
O	1.5298378468421052	0.98135533138947362	-0.20994565363157897
O	-1.5579896531578947	-0.41941290861052638	-2.1586919636315791
C	1.9594817468421053	-1.3587731886105263	0.48892873636842105
O	3.3002874468421051	-0.87347218861052633	0.47313362636842105
H	-3.5038503531578948	1.0759753813894737	-1.6786492636315788
H	-0.58661849315789483	-1.4831804886105264	0.30329353636842105
H	1.5661282468421054	-1.3736267886105262	1.5552939363684211
H	2.0421819468421050	-2.2945930886105264	6.1738883684210322E-003
H	3.2570041468421049	0.13004659138947366	0.49637909636842104
H	1.3204213468421053	-0.62326938861052628	-1.4611909636315790
H	1.2328100468421053	1.3011024113894736	0.63938353636842105
O	-1.7450503531578947	-2.5652368886105261	-3.7744567636315791
H	-1.6752871531578948	-1.9719182886105262	-4.4747282636315795
H	-1.8774530531578948	-1.8887953886105264	-3.0439021636315791
O	0.64601429684210521	0.71190741138947367	-3.8272606636315789
H	-2.2663416157894811E-002	0.21113632838947366	-3.3348262636315789
H	0.54702152684210514	0.37470520138947366	-4.7365913636315797
O	-2.9553069531578950	1.6660247113894737	-3.9006768636315790
H	-2.3869040531578949	1.6895081113894737	-4.6888790636315791
H	-2.3231931531578951	1.2615244613894738	-3.3009983636315789
O	-0.53826942315789483	-3.5279308886105265	-1.4488553636315789
H	0.29845209684210516	-4.0359761886105261	-1.5758512636315789
H	-0.72551948315789483	-3.1942108886105265	-2.3880145636315788
O	0.42316036684210517	2.9036828113894737	-1.8549197636315788
H	1.2423999468421052	2.4372708113894737	-1.6214588636315790
H	0.21794735684210520	2.2572587113894738	-2.4988829636315790
O	-3.9311056531578950	-3.1479699886105261	-1.6835969636315788
H	-3.0534108531578950	-3.4179960886105265	-1.4617256636315790
H	-4.2445840531578947	-2.9239053886105264	-0.78235590363157892

C	-1.2979064157894737	0.15162762684210529	-1.0428532227368421
C	-2.1518662157894735	1.0223154968421053	-0.33101392273684216
C	-1.7224301157894737	1.1164820468421053	0.99829042726315786
O	-0.71056066578947352	0.17482482684210529	1.2612778772631579
C	-0.40872254578947353	-0.52118807315789462	-3.5709997368421204E-003
O	-3.1643393157894733	1.8349381268421052	-0.78716661273684219
O	-1.9859071157894739	1.8843888268421052	1.9360956772631579
C	1.0874123442105263	-0.39518902315789467	-0.34394484273684212
O	1.5435090842105263	0.92831798684210520	-0.15019467273684212
O	-1.1900878157894736	-8.3036263157894696E-002	-2.2649655227368419
C	1.8846006842105263	-1.4721945731578947	0.53139138726315793
O	3.2639884842105267	-1.1354358731578948	0.46439977726315784
H	-3.4640576157894736	1.6856043268421053	-1.6977782227368421
H	-0.63925725578947357	-1.5576450731578948	9.2523619263157886E-002
H	1.5548108842105264	-1.5037397731578948	1.5281644772631580
H	1.7717446842105262	-2.4593723731578949	6.5508254263157872E-002
H	3.2964762842105264	-0.12713413315789468	0.36489083726315785
H	1.0456856442105265	-0.69223987315789470	-1.3646752227368422

H	1.2869069842105263	1.1486757668421053	0.74362090726315788
O	-2.5848377157894733	1.3862763268421052	-4.0093177227368422
H	-1.9951189157894735	1.3931699268421052	-4.8113667227368424
H	-2.1218766157894735	0.66008840684210535	-3.4913394227368419
O	1.1194814242105264	0.65549888684210522	-3.8653145227368420
H	0.42445762421052646	0.46400405684210533	-3.2811611227368420
H	1.0182284242105264	-4.3691553157894691E-002	-4.4568562227368425
O	-1.4381978157894737	-2.3241669731578947	-4.0230773227368424
H	-0.99163471578947349	-1.7548424731578947	-4.6963240227368424
H	-1.6736833157894737	-1.6627611731578948	-3.4089995227368419
O	0.14789104521052643	2.6738373268421052	-2.1333529227368420
H	0.55682355421052643	2.1582944268421054	-1.4289525227368423
H	0.22059642921052641	2.0993567268421054	-2.8907047227368419
O	-0.39994718578947352	-3.3205081731578949	-1.5302205227368422
H	0.42172313421052643	-3.8077901731578945	-1.6624913227368421
H	-0.58779264578947354	-3.1035892731578949	-2.4422408227368417
O	-2.1730476157894736	4.0745309268421055	-3.8188415227368417
H	-2.3821064157894734	3.1317484268421052	-3.6291528227368421
H	-1.9583454157894737	4.4768671268421061	-2.9502866227368418

C	-1.3367145689473683	7.6641036210526314E-002	-1.0639697402631578
C	-2.2105033689473683	0.92601220621052627	-0.42718789026315784
C	-1.8752239689473684	0.86894279621052628	0.97412531973684213
O	-0.64693857894736839	0.19689428821052632	1.1613984597368421
C	-0.34472756894736833	-0.51836930378947366	-5.0663016263157890E-002
O	-3.3186108689473683	1.6203972062105263	-0.90442993026315788
O	-2.4615323689473683	1.3990076062105263	1.9485245597368421
C	1.1818796310526316	-0.23312647378947365	-0.34473459026315789
O	1.5933509310526317	1.0580046662105262	2.6660080736842104E-002
O	-1.3259844689473683	-9.6153463789473703E-002	-2.3493553402631582
C	1.9496622310526317	-1.2894857937894737	0.50481062973684210
O	3.3771057310526316	-1.0143678937894736	0.57896317973684208
H	-3.3893836689473682	1.3862961062105263	-1.8171840402631578
H	-0.42806316894736834	-1.6237274937894737	9.8523459736842109E-002
H	1.5016145310526317	-1.3623735937894736	1.5213764597368422
H	1.8332464310526315	-2.2913965937894738	0.10495518973684212
H	3.4578970310526316	-8.2303263789473668E-002	0.71280154973684207
H	1.3445199310526317	-0.38239514378947365	-1.4228188402631579
H	1.0984061510526317	1.3615031062105263	0.74820449973684211
O	-1.6242573689473685	-2.3026081937894740	-4.0081219402631580
H	-1.0410677689473684	-1.9195411937894737	-4.6372587402631575
H	-1.7891708689473684	-1.4562129937894737	-3.4742735402631579
O	0.81846363105263165	0.78810053621052623	-3.9794075402631579
H	0.13388844105263165	0.31399063521052634	-3.4717462402631578
H	0.63128986105263163	0.37187546921052633	-4.8466475402631577
O	-2.6221796689473682	1.4690019062105264	-4.3613379402631578
H	-1.9389978689473684	1.6674275062105264	-5.0722412402631578
H	-2.0933700689473684	0.92371801621052629	-3.7537212402631579
O	2.5805511052631652E-002	2.6136184062105263	-2.2500752402631581
H	0.72924001105263159	2.2972695062105259	-1.6133933402631577
H	0.31409351105263167	2.0771621062105261	-2.9659810402631579
O	-0.70060487894736834	-3.5075469937894739	-1.5593000402631578
H	0.13906818605263166	-4.0240323937894740	-1.6973979402631578
H	-1.1424861689473684	-3.3573805937894741	-2.4563798402631578
O	-5.1003396689473686	-1.1373327937894737	-3.2382859402631579
H	-4.3159781689473684	-1.5984390937894737	-2.9309405402631579
H	-4.8767930689473680	-1.1167793937894737	-4.2151688402631580

C	-1.2784365810526317	9.9922526421052621E-002	-1.1097541754584210
C	-2.1936642810526314	0.91124835642105251	-0.45334012545842106
C	-1.8471999810526316	1.0900823064210525	0.88593764454157897
O	-0.71378487105263144	0.32123652442105260	1.2022564245415790
C	-0.37430889105263154	-0.46894069357894735	-2.0205763458421039E-002
O	-3.3711958810526315	1.3980653764210527	-1.0462654754584211
O	-2.3366014810526314	1.7147847764210526	1.8270649245415791
C	1.1230689289473685	-0.39369866357894739	-0.20107477545842103
O	1.6491927189473683	0.95496342642105270	-0.12666361545842103
O	-1.2376382810526316	-0.23216258357894737	-2.3609964754584212
C	1.9268549189473685	-1.2270245235789474	0.73796330454157899
O	3.3099177189473687	-0.93548452357894751	0.54827516454157899
H	-3.4289500810526312	0.88273922642105251	-1.8299009754584210
H	-0.65302725105263149	-1.5205832235789474	4.5602836251578960E-002
H	1.6137314189473684	-1.0660628235789475	1.7666766245415790
H	1.6870101189473683	-2.2506473235789470	0.47974583454157893
H	3.4839602189473684	3.2953486421052613E-002	0.30736361454157901
H	1.3310018189473685	-0.73906132357894738	-1.2769348754584211

H	1.3100697189473685	1.4276696764210526	0.62424988454157904
O	-1.4722244810526317	-2.5490873235789473	-3.8501047754584210
H	-1.0390772810526316	-2.0926472235789473	-4.6366739754584216
H	-1.6360428810526315	-1.8081472235789475	-3.1940889754584210
O	0.98750438894736847	0.91830209642105265	-3.8915731754584209
H	0.29554903094736851	0.61127551642105260	-3.2427224754584212
H	0.93641013894736846	0.27804001342105261	-4.5707364754584212
O	-2.7037645810526314	1.8510777764210526	-4.2771042754584219
H	-2.2093073810526316	1.8667645764210525	-5.1435172754584215
H	-2.2801600810526312	1.0567246264210526	-3.7981166754584210
O	-3.7292145810526316	-2.6998847235789474	-1.6775153754584211
H	-2.7495185810526315	-2.6468133235789471	-1.5985595754584210
H	-4.0395027810526312	-2.5691184235789470	-0.79437706545842102
O	-0.56902641105263152	-3.7214712235789471	-1.5410771754584209
H	0.37901402894736846	-4.0358563235789475	-1.4862735754584211
H	-0.65188837105263153	-3.2973586235789472	-2.4177193754584212
O	-4.9192486810526317	-1.0026152235789474	-3.4035298754584211
H	-4.1816419810526311	-1.4104250235789475	-2.9732596754584208
H	-4.5976401810526317	-1.0567050235789475	-4.2991346754584212

C	-1.2837912431578948	2.5927501052631613E-002	-1.1904727494736842
C	-2.1829619431578950	0.78058874105263154	-0.47213637947368420
C	-1.7349709431578950	0.88371115105263165	0.88462764052631582
O	-0.72667669315789485	7.8882910526316130E-003	1.1143268505263157
C	-0.39452371315789481	-0.60702520894736844	-0.15706432947368426
O	-3.3702374431578948	1.4330850010526315	-0.92259239947368421
O	-2.1971586431578949	1.6094631010526315	1.8161742505263159
C	1.1556660368421052	-0.21249292894736838	-0.27855308947368423
O	1.4625142568421052	1.1635812910526315	-7.7860269473684240E-002
O	-1.1040132431578948	-0.32107272894736838	-2.4242777494736840
C	1.8734125568421052	-1.1630495989473684	0.67532211052631574
O	3.2902048568421050	-0.96339369894736837	0.69435593052631572
H	-3.6521986431578948	1.1310489510526316	-1.8450730494736842
H	-0.58682778315789486	-1.6811138989473684	-0.13071194947368425
H	1.4679642568421052	-1.0573089989473685	1.6746226505263158
H	1.8346900568421052	-2.2099828989473682	0.37588630052631578
H	3.5255951568421051	-2.1512789473683847E-003	0.77879554052631572
H	1.5078311568421052	-0.33354578894736836	-1.2799347494736841
H	1.1154819568421050	1.5158430010526316	0.76456544052631581
O	1.2105524268421051	0.60668921105263163	-3.6976596494736844
H	0.45385477684210518	0.32890329105263161	-3.1065797494736844
H	0.96031769684210522	-1.4642088947368403E-002	-4.4360942494736850
O	-2.3648160431578948	1.5894717010526316	-4.1338617494736845
H	-1.8672061431578948	1.7351954010526316	-4.9529338494736850
H	-1.8091009431578948	0.96904485105263150	-3.6743432494736843
O	-1.9316926431578949	-2.4071664989473684	-4.0770648494736843
H	-1.5507607431578949	-1.7531063989473685	-4.7464089494736843
H	-1.8816979431578948	-1.9196781989473684	-3.2561265494736844
O	-0.41397770315789484	-3.6169684989473683	-1.5810017494736843
H	0.49689223684210515	-3.9797753989473681	-1.5776279494736842
H	-0.45215150315789487	-3.3054618989473683	-2.4720859494736844
O	0.38137592684210520	2.8184117010526317	-2.2002703494736844
H	0.80280919684210517	2.2678913010526318	-1.5303706494736842
H	0.60535611684210511	2.1706025010526315	-2.9728239494736841
O	-3.6070998431578949	-3.3149848989473680	-1.7159331494736842
H	-2.6363180431578948	-3.2362348989473682	-1.7476152494736843
H	-3.7944048431578952	-3.5236603989473680	-0.82281477947368420

C	-1.3584524121052632	4.8014921578947378E-002	-1.1334986984210527
C	-2.2250384121052633	0.82886239157894737	-0.41963118842105263
C	-1.9754307121052630	0.67687888157894738	0.94637809157894737
O	-0.88365751210526322	-0.12697359842105260	1.1510174015789472
C	-0.36067535210526314	-0.60089154842105263	-9.3214652421052632E-002
O	-3.3861196121052632	1.3732423715789475	-0.89745392842105265
O	-2.5756545121052632	1.1192778515789474	1.9121651015789474
C	1.0978752378947370	-0.22995730842105261	-0.21217545842105262
O	1.2372570578947368	1.1786383715789475	3.6068915578947362E-002
O	-1.3622149121052631	-0.22631758842105260	-2.4046468984210527
C	2.0908865878947367	-1.0610649284210525	0.59983714157894741
O	3.4900169878947369	-0.89121052842105264	0.38263482157894735
H	-3.2564109121052631	1.7297845715789473	-1.7985104984210527
H	-0.47205613210526320	-1.7504579284210524	-0.17866648842105262
H	1.7847366878947368	-0.93335242842105270	1.6179683015789472
H	1.9497413878947367	-2.1200603284210526	0.29097749157894737
H	3.7258583878947369	2.8669011578947384E-002	0.51637754157894733
H	1.3707567878947369	-0.43527485842105262	-1.2919001984210527

H	1.1085813578947368	1.3921926715789472	0.97627320157894737	H	0.95672653210526337	1.2990451357894739	0.87015288268421054
O	-1.7612817121052631	-2.4301429284210530	-3.9518644984210525	O	-2.7161169778947363	2.0547499857894738	-3.8316463573157891
H	-1.1205146121052632	-2.0932738284210526	-4.6301764984210525	H	-2.0591111778947364	2.1569635857894736	-4.6390549573157900
H	-1.6052262121052632	-1.6980659284210527	-3.3427951984210527	H	-2.1025127778947366	1.4647422857894736	-3.2770207573157895
O	-2.5829851121052632	1.6770537715789473	-3.9691867984210525	O	-1.1628498778947365	-2.1181574142105264	-4.0809386573157900
H	-2.1396429121052631	1.5168647715789474	-4.8637233984210528	H	-0.44534601789473655	-1.9077502142105263	-4.7354000573157897
H	-2.3596311121052631	0.81656305157894737	-3.5188244984210524	H	-1.1881004778947364	-1.3001654142105261	-3.5794266573157891
O	1.0954714978947369	0.56528114157894738	-3.8810067984210526	O	1.3743669221052635	0.30172639798947376	-3.9901316573157892
H	0.74701444789473681	0.16077233957894738	-3.0730303984210523	H	0.50623748210526343	8.2291957894737866E-003	-3.5698836573157893
H	0.62109569789473684	9.5985256578947381E-002	-4.5608919984210523	H	1.2260002221052635	0.21481415978947377	-4.9608513573157893
O	0.31566453189473681	2.6504442715789471	-1.9345228984210527	O	0.44977434210526346	3.1421215857894738	-2.2685194573157892
H	0.57949656789473680	2.1052955715789472	-1.1476627984210528	H	0.71431822210526352	2.5241714857894735	-1.5389021573157895
H	0.19248297289473681	2.0642906715789473	-2.6716427984210527	H	0.41477024210526348	2.5191646857894736	-2.9859181573157891
O	-3.9286467121052633	-2.9771151284210529	-1.7384821984210528	O	-4.2591339778947370	-1.7371690142105263	-2.0311667573157894
H	-2.9608175121052631	-2.7740216284210528	-1.9253511984210527	H	-3.3380448778947365	-1.8672856142105263	-1.9907440573157895
H	-4.0045862121052629	-2.6627549284210525	-0.80705134842105264	H	-4.4053715778947371	-1.9234558142105262	-1.0754885573157895
O	-5.3423653121052634	-1.2579014284210528	-3.6324217984210527	O	-3.1602285778947365	-1.1112443142105262	-5.6894638573157899
H	-4.5385786121052636	-1.4334480284210525	-3.1245512984210526	H	-2.8837212778947365	-1.7484732142105264	-6.3491378573157897
H	-4.9845770121052633	-1.2174072284210524	-4.5053799984210521	H	-2.4434681778947365	-1.3089714142105264	-5.0642041573157899
C	-1.3433151499999998	7.5070385263157902E-003	-1.0737509978947368	C	-1.2624663715789473	0.15157662684210532	-1.2106046544736841
C	-2.2994882499999996	0.62226607852631577	-0.24176998789473680	C	-2.1849237715789469	0.82166850684210535	-0.40889592447368422
C	-1.8516113500000000	0.57328240852631573	1.0947019021052631	C	-1.9232865715789471	0.63701371684210528	0.97591231552631574
O	-0.72510502999999982	-0.24383001147368422	1.0930476021052631	O	-0.78963547157894731	-0.12701203315789467	1.1030364455263157
C	-0.31371702999999984	-0.59614995147368421	-0.17623653789473681	C	-0.38041710157894726	-0.62768188315789464	-0.17451708447368419
O	-3.3933563499999995	1.4124452185263159	-0.60550183789473677	O	-3.1976835715789473	1.6526917368421055	-0.80809510447368427
O	-2.3786338499999999	1.0044676085263158	2.1743249021052633	O	-2.4578382715789475	1.2223860868421053	1.9230601455263159
C	1.1718483500000001	-0.12965374147368419	-0.35890950789473680	C	1.0970186584210526	-0.34706438315789467	-0.29037471447368418
O	1.3807862500000001	1.2453498185263159	-3.2184147894736823E-002	O	1.2886351284210527	1.0233649368421052	-4.3022089473684202E-002
O	-1.2756949499999999	-7.6097331473684193E-002	-2.3255385978947367	O	-1.0199849715789473	8.0825326842105305E-002	-2.4307393544736842
C	2.0289944499999999	-1.0697080814736841	0.43159124210526317	C	1.9539892284210527	-1.1417033631578946	0.63412714552631577
O	3.3831312499999999	-0.77604970147368424	0.56900012210526318	O	3.3629188284210532	-0.79793536315789459	0.42657477552631579
H	-3.4956832499999999	1.3598499185263158	-1.6360162978947368	H	-3.5057219715789474	1.4794721368421055	-1.7278249544736841
H	-0.27272260999999981	-1.6664820814736843	-0.34710743789473680	H	-0.57301613157894726	-1.7221870631578948	-0.21439736447368421
H	1.5264001500000002	-1.2401175814736842	1.3943688021052631	H	1.6545574284210527	-1.0021417631578946	1.6437228455263158
H	1.9900709500000002	-1.9609577814736843	-8.2348957894736813E-002	H	1.8971394284210528	-2.2217382631578948	0.41549945552631578
H	3.5098797500000005	0.16279654652631578	0.66172120210526320	H	3.5435271284210526	0.17088223684210532	0.57698679552631571
H	1.4652478500000001	-9.2752491473684195E-002	-1.3890015978947370	H	1.4107472284210527	-0.53460219315789470	-1.3008521544736842
H	0.89296882000000022	1.4638341185263157	0.84961013210526315	H	1.0864411484210528	1.2821849968421053	0.91040345552631574
O	0.80871845000000020	0.41679540852631580	-4.0011572978947374	O	-1.0421812715789474	-2.1930854631578947	-3.9629734544736843
H	0.15394402650000016	5.4189258526315787E-002	-3.3615432978947366	H	-0.45717525157894723	-2.1950143631578949	-4.7133553544736841
H	0.41731339000000017	-9.5016401473684231E-002	-4.7166584978947368	H	-0.91812217157894738	-1.2915405631578945	-3.5553678544736842
O	-1.5874815499999999	-2.5549037814736844	-3.5426743978947366	O	-2.2146312715789476	2.0984119368421053	-3.9443466544736840
H	-0.85307084999999994	-2.4342948814736838	-4.1490941978947369	H	-1.6130329715789473	2.2894529368421055	-4.6857807544736838
H	-1.4272043499999998	-1.7299824814736842	-3.0021046978947368	H	-1.6628883715789473	1.3896999368421055	-3.4564088544736840
O	-2.6933111499999995	1.7875944185263157	-3.8519025978947368	O	1.1704395984210527	1.0157297768421052	-3.9413109544736842
H	-2.1721432499999995	1.7674631185263159	-4.7026881978947372	H	0.42916129842105272	0.68450089684210536	-3.3882226544736840
H	-2.1894249500000003	1.0673733685263158	-3.4140952978947365	H	1.0236123384210527	0.50323526684210529	-4.7297534544736841
O	0.19275349400000016	2.7582511185263154	-1.9977543978947370	O	-3.9129921715789475	-2.0453440631578950	-2.0005680544736841
H	0.73632260000000016	2.3201618185263158	-1.2523043978947368	H	-2.9565408715789472	-1.9510227631578947	-1.9413180544736841
H	1.0136100000000176E-002	1.9706292185263159	-2.5004096978947365	H	-4.2241743715789477	-2.0195126631578946	-1.0925065544736841
O	-3.9956050500000000	-2.7099863814736844	-1.4907643978947369	O	0.19290191742105273	3.1180794368421054	-2.2278069544736843
H	-3.0652767499999998	-2.5661608814736843	-1.2598357978947370	H	1.0037302684210527	2.7587160368421051	-1.7386152544736841
H	-4.3766750500000002	-2.4632376814736840	-0.62898464789473674	H	0.29201452642105274	2.7177396368421052	-3.0788119544736841
O	-3.5998487499999996	-1.1711944814736843	-5.7830899978947370	O	-3.1256830715789476	-0.86225036315789472	-5.6677530544736845
H	-3.4176629499999995	-1.6656357814736842	-6.5722695978947367	H	-3.1479583715789472	-1.2842266631578947	-6.5520835544736844
H	-2.9179583500000001	-1.6175936814736842	-5.1415188978947368	H	-2.3285508715789476	-1.3003722631578944	-5.3091654544736837
C	-1.2647412778947364	0.17813628578947377	-1.1527487573157895	C	-1.2809886289473682	7.2479131578946365E-003	-1.0953788624210525
C	-2.1868002778947364	0.83487246578947372	-0.35195431731578947	C	-2.2298213289473683	0.74214676315789463	-0.41751830242105265
C	-1.8711408778947365	0.70699775578947377	1.0162433426842106	C	-1.8848880289473684	0.71957026315789463	0.94584815757894747
O	-0.79032087789473637	-0.13996473421052624	1.1409839426842106	O	-0.74885270894736822	-4.8977586842105347E-002	1.2115556375789474
C	-0.35345924789473654	-0.50204384421052628	-0.24538285731578946	C	-0.30842207894736828	-0.61635224684210543	-0.16136050242105263
O	-3.3348671778947363	1.4488334857894736	-0.80000171731578951	O	-3.2160658289473685	1.5712761931578947	-0.86670747242105262
O	-2.4120574778947366	1.0964097557894736	2.0662727426842107	O	-2.4931581289473685	1.1788216231578947	1.8943437375789474
C	1.1136444521052635	-0.15396472421052626	-0.33177181731578947	C	1.0757695910526317	-0.15296553684210534	-0.32426760242105263
O	1.3766684221052634	1.2318145757894738	8.9794316842105454E-003	O	1.3909879710526318	1.2071390531578947	4.9522546578947377E-002
O	-1.2433117778947365	0.16475294578947375	-2.4527384573157893	O	-1.1751927289473683	-9.7448906842105332E-002	-2.3881481624210528
C	1.9724979221052634	-1.0981510142105262	0.51743412268421052	C	1.9645995710526318	-1.1538187068421053	0.56275527757894739
O	3.3994682221052637	-0.97933141421052616	0.37894416268421055	O	3.3823265710526313	-0.94702040684210531	0.47772646757894738
H	-3.5252047778947366	1.0783210057894737	-1.7165898573157894	H	-3.4895398289473682	1.4146368931578945	-1.8004300624210525
H	-0.47056096789473650	-1.5874631142105264	-0.41342014731578947	H	-0.40357767894736829	-1.6702038068421055	-5.9932985421052618E-002
H	1.6639443221052634	-1.0190050142105260	1.6420159426842105	H	1.6599182710526317	-1.1042650068421054	1.6591424375789474
H	1.8018089221052636	-2.1134191142105263	0.28638176268421051	H	1.8406115710526318	-2.1922465068421051	0.24897373757894736
H	3.6960540221052636	-0.20603327421052620	0.92370615268421052	H	3.5081148710526313	-1.2108266842105364E-002	0.50791340757894743
H	1.4716519221052635	-0.23980716421052628	-1.3865065573157895	H	1.4695825710526318	-0.24296571684210538	-1.3856999624210526

H	0.93859598105263176	1.3975339931578945	0.94166250757894743	H	0.70624608526315780	1.3375455252631578	0.67941158989473682
O	-2.4614408289473686	1.7838771931578945	-3.8791037624210527	O	-0.91331367473684222	-2.7484448747368417	-3.6985680101052631
H	-1.7665506289473683	2.0642210931578946	-4.4744821624210527	H	-0.97224833473684225	-2.3803020747368420	-4.6067761101052627
H	-1.9824561289473681	1.0899837831578947	-3.3866569624210530	H	-0.97013256473684217	-1.8812144747368420	-3.2272117101052631
O	-1.1482878289473681	-2.4483751068421054	-3.8656499624210530	O	-2.4272947447368418	1.6418537252631578	-4.0771898101052626
H	-0.84565472894736815	-2.0560844068421051	-4.7183634624210526	H	-1.9662711447368422	1.5542098252631578	-4.9439684101052626
H	-1.1047005289473681	-1.6059145068421055	-3.3172193624210529	H	-1.9095082447368423	0.93829426526315785	-3.5977437101052629
O	1.0009064110526318	0.93028233315789466	-3.8300196624210527	O	1.2067605552631577	8.2545065263157885E-002	-3.9574296101052631
H	0.44413078105263171	0.31639031615789465	-3.3260140624210526	H	0.52243321526315789	-0.14714893473684210	-3.2614909101052634
H	0.83484423105263172	0.54905868315789463	-4.7598547624210532	H	0.87144607526315787	-0.42667230473684209	-4.7343840101052628
O	-4.2597721289473682	-2.0765673068421053	-2.0361264624210529	O	-0.22069622473684217	3.0117131252631579	-2.6354686101052631
H	-3.3201319289473683	-1.7391263068421055	-2.0127971624210526	H	0.63400326526315776	3.1655805252631581	-3.0199295101052632
H	-4.3742196289473680	-2.1674274068421053	-1.0620912624210526	H	-0.37119786473684219	2.0865216252631580	-2.8136548101052630
O	-0.20087135894736827	2.9027087931578950	-1.9882919624210527	O	-1.1193844447368422	-4.0185170747368426	-1.2914192101052631
H	0.47393692105263174	2.4482716931578947	-1.4296589624210525	H	-0.40068467473684216	-4.6731635747368427	-1.4491262101052631
H	2.5560861052631723E-002	2.6093351931578948	-2.8693812624210526	H	-0.93371090473684215	-3.4009399747368421	-2.0333530101052633
O	-0.83664393894736822	-3.5747394068421050	-1.1985858624210526	O	-5.0047386447368423	-1.1726563747368421	-2.0127249101052631
H	-3.5146908947368272E-002	-4.1780523068421056	-1.2209812624210525	H	-4.1160529447368424	-1.6071686747368421	-2.0671891101052631
H	-0.82690216894736823	-3.0925938068421051	-2.0920604624210526	H	-5.1053052447368428	-1.2072752747368423	-1.0195600101052631

C	-1.3399767821052631	-9.1464008000000013E-002	-1.1515220826315788	C	-1.4423359826315787	-9.1453710263157864E-002	-1.1184625110526316
C	-2.2348434821052630	0.65664639199999997	-0.41685225263157888	C	-2.2873119826315791	0.71345910973684212	-0.36336001105263160
C	-1.8584471821052631	0.60696385200000003	0.92673091736842106	C	-1.8548661826315789	0.71561040973684209	1.0059603589473685
O	-0.77771495210526320	-0.19271359800000001	1.0737657173684210	O	-0.70882446263157883	-7.1750970263157868E-002	1.1401217889473685
C	-0.35345028210526314	-0.68182293799999993	-0.24096688263157892	C	-0.36943257263157880	-0.68755989026315789	-0.17064277105263156
O	-3.3946994821052630	1.2554393719999999	-0.85511110263157897	O	-3.3751165826315792	1.4370935797368420	-0.75311816105263152
O	-2.4012530821052631	1.2435750719999998	1.8936173173684210	O	-2.2951683826315792	1.2752879797368419	2.0225258889473685
C	1.1819702178947369	-0.26414935800000006	-0.39762751263157892	C	1.1230683173684213	-0.35145860026315784	-0.41580883105263161
O	1.3113353178947369	1.1586885419999999	-0.17474777263157892	O	1.3352259173684211	1.0995733097368421	-0.26638515105263161
O	-1.2710263821052632	-0.27613409799999999	-2.4397525826315789	O	-1.4394153826315788	-0.40972890026315789	-2.3990351110526316
C	2.0496040178947368	-1.0629931280000000	0.63265657736842107	C	2.0100881173684209	-1.0685062202631579	0.63567008894736843
O	3.3904705178947370	-0.66077585799999994	0.45977040736842112	O	3.4448568173684211	-0.78940395026315791	0.49899045894736838
H	-3.0941156821052629	1.9787231719999998	-1.4518870826315791	H	-3.2099541826315789	2.0159121797368420	-1.5247823110526315
H	-0.49866432210526318	-1.7810735280000003	-0.23504321263157893	H	-0.46404334263157887	-1.7608881202631579	-5.2796721052631577E-002
H	1.5791750178947368	-0.92153402800000006	1.6462623173684210	H	1.6713441173684211	-0.84165239026315786	1.6809605889473684
H	1.9271967178947369	-2.1590240280000002	0.49346759736842111	H	1.8647379173684213	-2.0895173202631581	0.53770373894736845
H	3.4376881178947367	0.18699815400000000	0.97257590736842103	H	3.4858406173684209	0.20696994473684210	0.32152432894736838
H	1.5087044178947369	-0.37629415799999999	-1.4258756826315790	H	1.4375834173684212	-0.55250062026315783	-1.4210232110526315
H	0.83804728789473681	1.3809441720000000	0.69053940736842101	H	1.0737238173684212	1.2505141797368420	0.64195754894736845
O	-1.1132066821052631	-2.5322494280000001	-3.6393267826315787	O	-0.90052424263157882	-2.7768754202631580	-3.6389301110526318
H	-0.94690098210526330	-2.1433364280000000	-4.5466415826315796	H	-1.0134950526315789	-2.4872154202631580	-4.5193780110526314
H	-1.3335775821052631	-1.6773535280000000	-3.1598794826315788	H	-1.1289850426315788	-1.9031622202631580	-3.1994399110526319
O	0.80090436789473685	0.60456716200000005	-3.8728242826315786	O	-2.4158207826315792	1.7452250797368420	-3.7893273110526318
H	-1.6129832105263156E-002	0.33906276199999996	-3.4094984826315788	H	-1.8749372826315789	1.7252836797368420	-4.6118967110526317
H	0.82429770789473678	-0.10643649800000002	-4.5098978826315790	H	-2.0597719826315788	1.0223220297368421	-3.2418462110526316
O	-2.3211033821052629	1.6563367720000000	-4.1385953826315793	O	0.84906265736842124	0.11047779993684212	-4.0554840110526316
H	-1.6632845821052631	1.7081521719999999	-4.9176844826315795	H	0.13093987736842119	-0.15922608026315788	-3.4354029110526318
H	-1.9553459821052632	0.86084745200000001	-3.6804686826315787	H	0.53517476736842118	-0.50020578026315787	-4.8061246110526321
O	-4.4744675821052633	-1.8517441280000000	-2.0078531826315786	O	-0.15510319663157884	2.9131096797368419	-2.3639372110526318
H	-3.7380691821052632	-1.1895578280000001	-1.7816615826315789	H	0.45418317736842118	2.6851798797368422	-1.6262113110526315
H	-4.7465300821052629	-2.0620142280000002	-1.0815428826315789	H	0.10967665736842117	2.2030597797368419	-3.0224224110526317
O	-0.11695657210526315	3.0609362719999997	-2.5137781826315786	O	-5.3921742826315793	-1.4972091202631579	-1.8752309110526315
H	0.74367531789473684	2.7715291719999997	-2.1728353826315787	H	-4.5422450826315792	-1.1204616202631579	-2.0906554110526319
H	-0.21670409210526315	2.4539859719999999	-3.1929272826315787	H	-5.3234673826315788	-1.9318284202631579	-1.0100198110526315
O	-3.7224409821052631	-1.1452874280000001	-5.6886658826315788	O	-1.1943889826315788	-4.1418554202631581	-1.4985750110526315
H	-3.7651634821052631	-1.0984719280000002	-6.6258633826315796	H	-0.31736158263157882	-4.5379714202631583	-1.3267558110526316
H	-2.7634598821052632	-1.1965378280000001	-5.4152018826315791	H	-0.96334759263157876	-3.5957802202631579	-2.3384538110526316

C	-1.3012822447368422	-0.10271569473684211	-1.1827550101052631	C	-1.2904326857894737	-4.8830428315789487E-002	-1.2047808826315789
C	-2.1031845447368420	0.74594577526315797	-0.45287446010526317	C	-2.0977734857894736	0.78651870168421056	-0.50222808263157903
C	-1.8400002447368422	0.57419851526315790	0.90738286989473682	C	-1.7968878857894737	0.72132172168421049	0.85442009736842106
O	-0.74879368473684216	-0.25686759473684212	1.1780061898947369	O	-0.78747178578947374	-0.10453962831578950	1.0929986173684212
C	-0.32209485473684218	-0.73549965473684220	-0.15329165010526313	C	-0.39170999578947363	-0.71089211831578947	-0.16436314263157895
O	-3.0762828447368422	1.5923749252631578	-0.85268281010526314	O	-3.2772586857894739	1.4072137816842105	-0.89269232263157905
O	-2.3529848447368420	1.1315965252631579	1.9225141898947369	O	-2.4087088857894736	1.2414576816842104	1.7997035173684210
C	1.1653683552631577	-0.29372786473684215	-0.35417241010526318	C	1.0537304142105264	-0.45291143831578945	-0.32912611263157893
O	1.1993893552631578	1.0973802452631578	-0.14877433010526314	O	1.3560687142105263	0.90622191168421051	-0.51036025263157891
O	-1.2158003447368422	-0.30673063473684209	-2.4513170101052633	O	-1.2438633857894736	-0.40980048831578947	-2.4041972826315789
C	2.0009729552631579	-0.92589047473684216	0.66885033989473686	C	1.8682310142105265	-0.95498901831578964	0.90691913736842111
O	3.3103845552631581	-0.46226944473684212	0.65857086989473679	O	3.2962960142105260	-0.85193811831578947	0.58365087736842103
H	-3.3414252447368420	1.3885844252631578	-1.7766810101052632	H	-3.1203784857894736	2.0974641816842103	-1.5623405826315788
H	-0.39698207473684216	-1.8556252747368422	-6.7609368105263135E-002	H	-0.58305374578947367	-1.7668238183157896	-3.6454422631578942E-002
H	1.5274954552631577	-0.84623357473684213	1.6549929898947369	H	1.6792391142105265	-0.33903438831578953	1.8349440173684211
H	2.0442832552631578	-2.0262847747368418	0.49834768989473682	H	1.7515589142105263	-2.0090169183157895	1.1192070173684212
H	3.2811969552631579	0.54111212526315788	0.70600773989473686	H	3.3807514142105264	0.12357711968421051	0.31282463736842103
H	1.4634939552631578	-0.59689307473684217	-1.4339264101052631	H	1.5858366142105265	-0.97131281831578953	-1.2017839826315788

H	1.0258268142105265	1.3363140816842105	0.30365914736842103
O	-0.86333584578947364	-2.6857918183157894	-3.8103721826315793
H	-0.58043604578947372	-2.2028312183157897	-4.6406451826315784
H	-0.99414177578947371	-1.8479510183157895	-3.2790015826315790
O	0.75409614421052629	0.76633896168421045	-3.9176628826315789
H	0.20597362421052631	0.28759972868421052	-3.2596236826315792
H	0.80861744421052628	6.1147181684210489E-002	-4.5674606826315785
O	-2.6085301857894736	1.6870215816842105	-3.8980572826315791
H	-2.1126956857894736	1.5716593816842104	-4.7533490826315790
H	-2.3611042857894740	0.90029480168421050	-3.4635228826315791
O	-1.0606591857894736	-3.9047633183157897	-1.4649232826315788
H	-0.20311483578947367	-4.3351870183157892	-1.2295406826315789
H	-0.83343008578947364	-3.4115209183157895	-2.2359849826315790
O	0.31830991421052635	3.0713886816842106	-1.9156457826315789
H	0.51544710421052631	2.1016936816842104	-1.9200924826315788
H	0.83039625421052632	3.4575643816842105	-2.6364140826315792
O	-0.18865997578947366	-0.83924001831578954	-5.7883435826315788
H	-0.21471745578947365	-1.1738309183157896	-6.6856640826315790
H	-0.43816572578947366	9.7911941684210496E-002	-5.9399850826315790

C	-1.3649990936842105	-5.6488025789473650E-002	-1.1216784084210527
C	-2.2997982936842103	0.68483525421052627	-0.37379040842105260
C	-1.9032841936842104	0.69560452421052632	0.97322081157894735
O	-0.85720791368421034	-0.12162846578947367	1.1886609915789472
C	-0.39932473368421045	-0.66583519578947370	-0.12304059842105265
O	-3.3206267936842107	1.4331951542105263	-0.82301326842105271
O	-2.3592636936842104	1.2389665942105264	2.0292048915789476
C	1.0616469063157894	-0.41033373578947363	-0.34906806842105265
O	1.4001333063157895	0.96474967421052638	-0.57605435842105268
O	-1.2324039936842106	-0.10212854578947367	-2.3851007084210525
C	1.8989059063157896	-1.0062034457894737	0.74848093157894735
O	3.2606870063157896	-0.92662284578947363	0.48512215157894739
H	-2.9609945936842106	2.1827318542105263	-1.3791821084210527
H	-0.53488545368421037	-1.7440025457894737	-0.20624937842105262
H	1.7206300063157896	-0.54594473578947367	1.6946211915789473
H	1.6690341063157894	-2.0527363457894738	0.87649652157894731
H	3.6027269063157896	-3.6887757894736861E-003	0.37787610157894735
H	1.2088488063157894	-0.96974514578947368	-1.3064141084210528
H	1.4101758063157894	1.4052747542105264	0.26990782157894738
O	-0.71289940368421034	-2.5191238457894736	-3.4968029084210528
H	-0.18121239368421044	-2.2979505457894733	-4.2838727084210522
H	-0.95848544368421040	-1.6047468457894736	-3.1382469084210527
O	-2.5650757936842106	1.5402939542105263	-3.9810763084210527
H	-1.8560027936842105	1.4447294542105262	-4.6669673084210519
H	-2.1719450936842106	0.94862982421052633	-3.3518140084210528
O	1.1444827063157894	0.16126669421052633	-3.6350168084210526
H	0.48084452631578961	-5.5012125789473676E-002	-2.9376917084210525
H	0.61017395631578963	-0.15138484578947370	-4.4407403084210522
O	0.37048776631578956	3.1748406542105263	-1.7040950084210527
H	0.63340585631578961	2.2530244542105260	-1.5346281084210527
H	1.1492545063157895	3.6998716542105266	-1.4314808084210526
O	-0.16916199368421042	-1.0022368457894737	-5.7500406084210525
H	0.10103427631578958	-0.74853389578947371	-6.6717236084210523
H	-1.0598396936842105	-0.51334665578947369	-5.5930366084210519
O	-1.0545778936842105	-3.7691343457894737	-1.1166059084210527
H	-0.26268583368421045	-4.2932597457894737	-0.89395636842105264
H	-0.96632941368421044	-3.4498136457894732	-2.0449882084210529

C	-1.2706240142105263	6.1254436157894748E-002	-1.1074238036842106
C	-2.1895382142105264	0.91954359615789483	-0.42886469368421054
C	-1.9412879142105262	0.68209465615789477	0.95081351631578948
O	-0.78523977421052626	-6.9768733842105274E-002	1.2025870963157894
C	-0.33022697421052627	-0.60341895384210520	-6.4021873684210501E-002
O	-3.4504671142105265	1.3861694161578946	-0.76448222368421048
O	-2.5926458142105266	1.1043800761578948	1.8998433963157895
C	1.1345547857894738	-0.44171726384210519	-0.37411316368421055
O	1.4811886857894736	0.91590162615789483	-0.42453993368421050
O	-1.1065954142105263	-0.12383779384210522	-2.3371683036842104
C	2.0117045857894738	-1.0167557838421053	0.72364596631578948
O	3.3667065857894736	-0.82150228384210522	0.51927479631578943
H	-3.3638467142105264	1.9438824161578947	-1.5535327036842106
H	-0.45697779421052631	-1.6529239838421053	-9.0929313684210505E-002
H	1.6377522857894737	-0.64152287384210527	1.7082067963157894
H	1.8961626857894738	-2.1449977838421050	0.71553999631578946
H	3.5541563857894736	0.13101919915789476	0.35601395631578947
H	1.3738684857894736	-0.98618098384210529	-1.2619976036842107

H	1.0313552557894736	1.3583810161578946	0.33114809631578945
O	-0.64967933421052626	-2.4845001838421052	-3.3729561036842104
H	-0.56841036421052626	-1.9578471838421054	-4.1614648036842103
H	-0.92121671421052631	-1.7914781838421054	-2.7419972036842104
O	-2.2837053142105264	1.8733913161578946	-3.9661634036842104
H	-1.8679583142105263	1.9761636161578946	-4.8861448036842106
H	-1.8332824142105264	1.0455980161578948	-3.6245558036842103
O	1.3587151857894737	0.35947216615789479	-3.6365645036842102
H	0.62494961578947372	0.40273981615789478	-2.9672769036842106
H	0.98156423578947372	-0.18215136384210526	-4.3936766036842103
O	0.26500360578947368	3.0148674161578950	-1.8722497036842107
H	0.62862523578947371	2.1183841161578947	-1.6891945036842106
H	1.1483077857894737	3.3318081161578950	-2.0539613036842104
O	-1.1845062142105263	-4.0904703838421055	-1.2124125036842106
H	-0.32124952421052627	-4.3613175838421050	-0.80185802368421055
H	-0.85775390421052633	-3.3965123838421052	-1.8327756036842107
O	-0.26568886421052629	-0.90384318384210516	-5.8397289036842102
H	8.6881403689473696E-002	-1.4844409838421053	-6.5514732036842105
H	-0.67180562421052625	-0.24078195384210524	-6.4091363036842104

C	-1.2754054457894737	-3.1036635789473632E-002	-1.1135930709842108
C	-2.3065518457894738	0.72129883421052643	-0.46471186098421058
C	-1.9860693457894738	0.71039512421052642	0.91843150901578940
O	-0.83835624578947354	-8.0424125789473611E-002	1.1601222990157893
C	-0.40563187578947357	-0.67349983578947370	-4.9889900984210644E-002
O	-3.4265620457894737	1.2925399342105264	-1.0187734709842107
O	-2.5318350457894740	1.2141302742105264	1.9228140290157896
C	1.1133447442105264	-0.43459845578947359	-0.27588930098421061
O	1.3532033542105264	0.97326800421052639	-0.26048729098421064
O	-1.0257802457894736	-0.12278947578947363	-2.3258147709842105
C	2.0446186542105265	-1.0604531357894735	0.69066187901578946
O	3.3930982542105261	-0.73261603578947354	0.25026474771578938
H	-3.2810676457894736	2.1847415642105261	-1.4266934709842105
H	-0.60526770578947364	-1.7130502357894735	3.5607890157893785E-003
H	1.8481116542105265	-0.68346001578947368	1.7521544290157895
H	2.0417946542105261	-2.1607734357894737	0.59119962901578937
H	3.5538822542105262	0.19516473421052638	0.36492318901578935
H	1.4539948542105263	-0.88330403578947347	-1.2383033709842106
H	0.88047902421052637	1.2844669542105265	0.52002400901578938
O	-0.45774441578947356	-2.5102143357894739	-3.3657735709842109
H	-0.26611524578947365	-2.0824790357894738	-4.2675237709842104
H	-0.84567574578947369	-1.7876906357894737	-2.8827342709842108
O	1.2967795542105265	0.46726942421052636	-3.8105560709842110
H	0.39052421421052635	0.39367728621052633	-3.4610820709842107
H	1.1824402542105263	-7.0683725789473628E-002	-4.5816241709842105
O	-2.2009507457894739	2.0975290642105264	-3.9108261709842105
H	-1.5317256457894737	2.4052389642105263	-4.5399436709842105
H	-1.7885828457894737	1.4823057642105264	-3.3136918709842109
O	0.28201903421052638	3.1171933642105261	-1.8680477709842109
H	0.50311727421052632	2.1813332642105263	-1.6264140709842105
H	1.1210246542105264	3.3597698642105263	-2.2558035709842108
O	-4.5448878457894741	-1.1953399357894736	-1.8607676709842109
H	-3.9795389457894741	-0.43878547578947363	-1.9778931709842107
H	-4.3314833457894739	-1.3644851357894736	-0.93510247098421073
O	-0.43329954578947361	-0.89448663578947363	-5.6034686709842099
H	0.17244140981052636	-1.1250194357894736	-6.3477869709842105
H	-1.2019060457894737	-0.49876609578947367	-5.9590792709842102

C	-1.3324109242105262	2.6777879473684180E-002	-1.1568728426315789
C	-2.2355583242105261	0.76355059947368420	-0.49025338263157892
C	-2.0313424242105267	0.78125794947368421	0.93094984736842101
O	-0.83994582421052622	4.3791989473684179E-002	1.1808421473684210
C	-0.36790616421052624	-0.58185194052631584	-6.8138522631578963E-002
O	-3.3747966242105258	1.3103868394736842	-0.96397574263157892
O	-2.5195530242105262	1.2403130794736841	1.9292716573684210
C	1.1054094057894739	-0.30227343052631578	-0.34532947263157898
O	1.5001627757894738	1.0677595194736842	-0.19804331263157895
O	-1.1057115242105262	-0.10083619052631582	-2.3786305426315790
C	1.9305101757894738	-1.1730234605263157	0.65496075736842108
O	3.3546967757894741	-0.89361546052631580	0.62866871736842100
H	-3.2062759242105265	1.9107983394736843	-1.7357961426315789
H	-0.56251815421052631	-1.6845378605263157	-4.6804192631578950E-002
H	1.5448329757894737	-0.93695056052631576	1.6441035573684211
H	1.8378229757894737	-2.2389530605263159	0.40689575736842104
H	3.5897598757894738	2.7111489473684164E-002	0.59855157736842102
H	1.4161734757894737	-0.55326306052631580	-1.3354998426315789

H	1.2966504757894737	1.2935573394736841	0.74509997736842104
O	-1.9617240242105261	2.0125262394736843	-3.8526836426315794
H	-1.2329672242105263	2.2489837394736840	-4.4514936426315792
H	-1.6800592242105263	1.2161775494736842	-3.3314054426315791
O	-0.33684002421052628	-2.5767644605263160	-3.3888906426315790
H	-0.56729459421052630	-2.3009560605263162	-4.3559927426315790
H	-0.55970700421052622	-1.7678658605263158	-2.9619682426315790
O	1.3601872757894737	0.44541227947368417	-3.9065799426315793
H	0.51623665578947375	9.2980629473684184E-002	-3.5079342426315789
H	1.3447304757894738	7.3913539473684176E-002	-4.8407067426315793
O	-0.47682278421052626	-0.90535196052631584	-5.6329563426315792
H	-4.0596354210526264E-002	-1.4143632605263157	-6.2924055426315793
H	-1.4632882242105263	-0.90074746052631571	-5.8871585426315791
O	-5.0382461242105263	-0.97869256052631570	-1.6181080426315790
H	-4.4612289242105261	-0.24642357052631586	-1.5894246426315790
H	-4.8216429242105256	-1.3296105605263158	-0.70326401263157889
O	-0.75333482421052622	-4.0006272605263158	-1.1578485426315790
H	0.17492190978947375	-4.0895598605263164	-0.76517327263157897
H	-0.53794001421052628	-3.5023733605263159	-1.9960957426315789

C	-1.2189283326315790	0.11509431368421047	-1.1808727463157895
C	-2.2286785326315792	0.77688459368421048	-0.51789326631578958
C	-2.0619988326315788	0.66062333368421045	0.87770220368421059
O	-0.89833903263157899	-9.9339486315789538E-002	1.1133769236842106
C	-0.35247843263157908	-0.58615873631578963	-0.18671613631578948
O	-3.3203233326315793	1.4102946336842106	-1.0257666463157895
O	-2.6863921326315792	1.1041440036842105	1.8762758536842103
C	1.1798313773684210	-0.32395851631578959	-0.32807039631578944
O	1.5292080673684210	1.0806058936842105	-0.10910460631578950
O	-0.95711613263157913	0.12507366368421047	-2.4146684463157895
C	1.8564273673684211	-1.1586852663157894	0.73431077368421049
O	3.3564488673684210	-1.0254395663157896	0.70884615368421056
H	-3.1475203326315793	2.0002517336842103	-1.8129399463157896
H	-0.63974575263157907	-1.6571562663157893	-0.21281582631578949
H	1.5889815673684209	-0.92740026631578942	1.7592255536842105
H	1.7171366673684210	-2.2400356663157899	0.52445102368421048
H	3.5088735673684210	-7.6513886315789537E-002	0.79765810368421053
H	1.5810027673684210	-0.56272040631578957	-1.3109042463157896
H	1.1936105973684210	1.3844358936842105	0.70790567368421053
O	-1.8388699326315792	2.1886437336842106	-3.8315086463157897
H	-1.4963158326315791	2.1695971336842104	-4.7157898463157890
H	-1.4496106326315790	1.3063663636842104	-3.4986372463157895
O	1.3077896673684211	0.69652182368421045	-3.7635187463157891
H	0.62950609736842100	0.32617706268421048	-3.1856860463157894
H	0.94772824736842098	0.26648366368421050	-4.6023897463157892
O	2.6807587368420982E-002	-2.5377989663157896	-3.4522130463157894
H	4.9738477368420964E-002	-2.1309898663157898	-4.3231005463157892
H	-0.15484498263157903	-1.7121540663157895	-2.9978789463157893
O	0.41219102736842095	3.2954155336842104	-1.8086377463157894
H	1.1964311873684210	2.8344349336842103	-1.5116587463157896
H	0.31116694946842099	2.9526009336842103	-2.6765015463157895
O	-0.41943352263157907	-0.50722286631578961	-5.4991626463157894
H	-0.34645445263157904	-0.50927146631578957	-6.4585056463157891
H	-1.2207945326315790	-1.0391542663157896	-5.4425628463157896
O	-4.9182911326315795	-0.73679286631578944	-1.4309103463157895
H	-4.0255055326315796	-0.30093182631578957	-1.4843199463157895
H	-4.7896808326315794	-1.1521064663157894	-0.56387485631578960

C	-1.2013974757894734	0.15335010052631581	-1.2770251436842106
C	-2.1250420757894739	0.84872260052631576	-0.52280640368421061
C	-1.8598656757894736	0.74800172052631586	0.90292559631578939
O	-0.69265697578947361	-3.4721749473684171E-002	1.0703027363157895
C	-0.29351900578947354	-0.48521045947368419	-0.25345468368421054
O	-3.2756226757894735	1.4367042805263159	-0.94832114368421050
O	-2.5202369757894738	1.1905292005263157	1.8681217563157895
C	1.2151194342105265	-0.25791970947368420	-0.27760333368421053
O	1.5013113242105265	1.1252544105263158	-0.12785869368421060
O	-1.0113276757894736	6.0604270526315784E-002	-2.4978675436842104
C	1.8212736242105265	-1.2023400194736842	0.80127799631578944
O	3.2558876242105264	-1.1311111194736840	0.92612641631578940
H	-3.2352948757894735	1.4464019805263160	-1.9270841436842105
H	-0.53482490578947350	-1.5144632194736842	-0.34943516368421057
H	1.4140010242105265	-0.87065351947368419	1.8193349563157895
H	1.4768306242105265	-2.2194000194736843	0.56500139631578938
H	3.4144525242105264	-0.20257395947368423	0.79329435631578937
H	1.6749922242105264	-0.50708244947368430	-1.2363928436842107

H	0.97591991421052648	1.4159076605263159	0.67146388631578935
O	-2.0037673757894732	2.1871548805263159	-3.8896773436842107
H	-1.4637079757894735	2.0833550805263159	-4.7187181436842103
H	-1.6349067757894735	1.4086227705263159	-3.3998900436842105
O	1.4934426242105265	0.59744173052631577	-3.5379428436842106
H	0.79912272421052655	0.40190012552631582	-2.9185114436842108
H	1.1739968142105264	0.11373943052631580	-4.3647548436842110
O	0.43060175321052652	-2.3560498194736845	-3.3112018436842106
H	4.2057784210526472E-002	-2.8845068194736845	-4.0051845436842104
H	7.7355884210526471E-002	-1.4794888194736842	-3.5427815436842107
O	1.0484271642105265	2.9911046805263157	-2.0338748436842105
H	1.2646514042105266	2.2212037805263156	-1.4543206436842107
H	0.82450031421052650	2.6770255805263155	-2.8563908436842107
O	-0.44232925578947346	-0.66458801947368418	-5.5814860436842109
H	-0.28090752578947353	-0.54953461947368409	-6.5641419436842110
H	-1.2423105757894735	-1.1671348194736841	-5.6023924436842103
O	-0.86068657578947350	-3.9589986194736841	-1.2529994436842107
H	-1.6104465789473499E-002	-4.4174372194736842	-0.98943454368421069
H	-0.55667369578947357	-3.2741483194736842	-1.8467528436842104

C	-1.1999001836842105	5.6289032999999988E-002	-1.3280433968421053
C	-2.2101978836842107	0.66849708299999999	-0.55984522684210536
C	-1.9313738836842105	0.53452067599999997	0.78310944315789466
O	-0.87848698368421041	-0.28544648700000008	0.99983478315789465
C	-0.33712222368421052	-0.70537634700000007	-0.30686423684210529
O	-3.3250858836842103	1.2688586730000000	-1.0684719968421053
O	-2.5734414836842103	1.0820253929999999	1.7303852031578948
C	1.2248891663157895	-0.43189002700000001	-0.26676671684210529
O	1.4345712163157895	0.95834025300000003	-0.43353747684210531
O	-0.93696028368421058	0.18162648299999995	-2.5426706968421051
C	1.8248556163157894	-0.99573234700000013	1.0542707731578946
O	3.2829689163157894	-0.92361334700000008	1.0013623531578948
H	-3.1311708836842107	2.1390917529999998	-1.5330185968421053
H	-0.63528852368421052	-1.7624223470000000	-0.39626930684210526
H	1.4235141163157894	-0.36043471700000007	1.8855880031578949
H	1.6343650163157895	-2.1243031470000000	1.0872039531578948
H	3.4666587163157896	1.4828512999999988E-002	0.71110331315789477
H	1.7273585163157894	-0.91774524700000004	-1.0499004968421053
H	1.1398469363157895	1.6028861530000000	0.23253032315789471
O	1.3475218163157896	0.79822561299999994	-3.5759103968421053
H	0.59846146631578945	0.60408287299999996	-2.9367960968421052
H	1.1123363563157895	0.15403041299999998	-4.2776185968421050
O	-2.2757669836842105	2.1600423529999997	-3.7546724968421050
H	-1.7386664836842103	2.4026316530000003	-4.4787440968421048
H	-1.6488472836842105	1.4612553529999999	-3.3833004968421050
O	0.61239022631578943	-2.6549044469999998	-3.6250310968421053
H	0.95998544631578953	-3.0698695469999997	-4.4120342968421049
H	0.24138192931578950	-1.7594271470000000	-3.9213210968421048
O	0.87122180631578949	3.3950433530000002	-2.1369532968421052
H	1.7122462163157894	3.1082881530000002	-1.7825780968421050
H	0.49900215631578948	2.5195889530000004	-2.5246213968421052
O	-0.36947216368421054	-0.85576194699999997	-5.5696466968421054
H	-0.29936552368421054	-0.90011324699999995	-6.5192445968421051
H	-1.1214157836842105	-1.4463512470000000	-5.5023410968421054
O	-4.8864502836842103	-0.98628904700000009	-1.4350332968421051
H	-4.1422912836842105	-0.36576298699999998	-1.4764460968421051
H	-4.8275889836842101	-1.2559563470000001	-0.48156629684210528

C	-1.2660527663157894	-9.6723241052631692E-002	-1.2554822548947369
C	-2.0734817663157892	0.74879608894736838	-0.47741798489473680
C	-1.7527021663157896	0.65916531894736829	0.93216002510526308
O	-0.79754046631578945	-0.31814800105263169	1.1140649351052632
C	-0.29555370631578942	-0.72783371105263162	-0.18617653489473679
O	-3.2320317663157891	1.4648727889473685	-0.87067895489473668
O	-2.2469368663157891	1.2378507389473683	1.9167781451052632
C	1.1740538736842105	-0.39872089105263159	-0.24417762489473677
O	1.4384754736842105	1.0131221089473683	-0.50629113489473676
O	-1.3318937663157895	-0.27269863105263165	-2.4982298548947366
C	1.8330811336842106	-0.86879021105263154	1.0307376951052631
O	3.2851234336842108	-0.93286701105263159	0.76789406510526326
H	-3.0964557663157892	2.0531261889473682	-1.6031467548947367
H	-0.52047196631578951	-1.7832675110526315	-0.18463839489473682
H	1.5866939336842105	-0.28353522105263168	1.9165144451052631
H	1.5395646336842104	-1.9436371110526316	1.2554068451052631
H	3.5712149336842107	-0.11651527105263165	0.41887272810526321
H	1.5931416336842106	-0.96696101105263166	-1.0882639548947368

H	0.59177195368421054	1.5327645889473684	-0.43792543489473684	H	0.93760135894736840	1.2951006945789474	0.70759863157894731
O	-2.0931886663157893	2.0287963889473684	-3.8734866548947364	O	1.5137237289473684	0.61632633457894737	-3.9026926784210532
H	-1.4513955663157896	2.1219823889473681	-4.5969076548947365	H	0.76032534894736836	0.36601944757894733	-3.3144089784210529
H	-1.9015444663157897	1.1356063589473684	-3.4656355548947366	H	1.2198067189473685	0.24430174457894729	-4.6970211784210525
O	1.1462742536842105	1.0722932789473683	-3.6294549548947366	O	-2.1314678710526316	2.3328633345789473	-3.9499310784210526
H	0.28648228368421053	0.93404111894736830	-3.2038974548947365	H	-1.5108960710526316	2.2935371345789473	-4.6671086784210525
H	1.0078707836842105	0.42565293514736835	-4.3482751548947371	H	-1.8152625710526316	1.5277886345789473	-3.4263996784210526
O	0.80890384368421053	-2.7822553110526318	-3.4605785548947368	O	5.9068518947368365E-002	-1.0635070654210528	-5.6090741784210527
H	1.0444552636842106	-3.3401110110526320	-4.2655994548947369	H	-0.14193723105263167	-0.89819616542105285	-6.5576028784210525
H	0.61687379368421058	-2.0042868110526317	-3.9823896548947371	H	-0.65683807105263159	-0.62236746542105270	-5.0577658784210522
O	-4.5317153663157894	-1.1460671110526315	-1.0548399548947367	O	0.46053103594736838	-2.8400734654210527	-3.5016224784210528
H	-4.1781673663157894	-0.30541917105263167	-1.3015559548947369	H	0.63879139894736836	-3.7026233654210525	-3.9717517784210528
H	-4.5240371663157894	-1.1386331110526315	-7.4859804894736814E-002	H	0.46998821394736834	-2.3337362654210527	-4.3073216784210526
O	-0.66174106631578944	-3.8512524110526316	-1.3582998548947367	O	1.3665815489473685	3.5798349345789475	-1.7806400784210525
H	0.17269430368421052	-4.2300975110526320	-1.0141186548947367	H	2.3035222289473687	3.5835303345789473	-1.8996931784210525
H	-0.32282704631578946	-3.1498155110526320	-1.9688790548947366	H	1.2443915289473684	2.6272119345789475	-1.5833963784210525
O	0.16841781368421055	-0.73449941105263150	-5.4819088548947370	O	-0.82505967105263167	-3.9799535654210523	-1.2187064784210526
H	0.24778438368421052	-0.55717053105263159	-6.4293824548947365	H	1.1537778947368349E-002	-4.3492965654210529	-0.97605457842105259
H	-0.57406756631578948	-1.3049567110526317	-5.4554644548947371	H	-0.53750852105263158	-3.4976357654210526	-2.0333407784210529

C	-1.3315392268421054	0.11207100105263160	-1.1601109789473683	C	-1.1463254131578946	9.4331037368421106E-002	-1.2459647552631579
C	-2.1439528268421055	0.87929676105263166	-0.31419274894736837	C	-2.1726596131578946	0.66981882736842113	-0.45691223526315794
C	-1.8365032268421053	0.64269954105263161	1.0172312010526317	C	-1.9178238131578946	0.44739835736842110	0.91361603473684205
O	-0.77887712684210531	-0.26118664894736837	1.1316897210526315	O	-0.80879311315789471	-0.34978995263157892	1.0539540747368421
C	-0.38259492684210533	-0.69697525894736856	-0.19529067894736840	C	-0.29854210315789459	-0.65679899263157893	-0.26569970526315789
O	-3.3768260268421053	1.4277055410526316	-0.77318897894736827	O	-3.3590432131578947	1.3688926473684211	-0.87826125526315790
O	-2.4194203268421055	1.0190053910526315	2.0570411210526318	O	-2.5735243131578946	0.87363241736842112	1.8489916447368422
C	1.1221865731578946	-0.41416804894736842	-0.40370870894736832	C	1.1375697168421055	-0.16271147263157892	-0.24274890526315784
O	1.4188290731578947	0.95146091105263153	-0.42939364894736831	O	1.2775865568421054	1.1976194873684212	-3.8083285263157907E-002
O	-1.2302864268421052	3.6212831052631567E-002	-2.4384004789473686	O	-0.91828031315789471	-1.1380526315789008E-003	-2.4460449552631580
C	1.9500140731578948	-1.1055685589473685	0.70880440105263165	C	1.8606028868421054	-0.93431595263157896	0.78498389473684216
O	3.3221474731578944	-0.73086105894736852	0.60945938105263164	O	3.2896274868421056	-0.83268425263157875	0.66727209473684213
H	-3.2169569268421054	2.0712434410526317	-1.5295414789473685	H	-3.3403084131578944	1.5659463473684212	-1.8688681552631579
H	-0.47225964684210536	-1.7687117589473684	-0.18776027894736835	H	-0.34940677315789453	-1.7469206526315788	-0.52657820526315791
H	1.5645263731578947	-0.72433815894736853	1.6465277210526317	H	1.4873890868421054	-0.63996853263157893	1.7867534447368421
H	1.8787282731578947	-2.2051413589473681	0.57864812105263164	H	1.6649987868421054	-1.9789924526315787	0.64861625473684215
H	3.3743289731578945	0.19439125105263161	0.53739528105263168	H	3.4820308868421055	7.9994027368421095E-002	0.56727269473684205
H	1.4528862731578946	-0.77330685894736839	-1.3075754789473684	H	1.5501708868421054	-0.42907228263157887	-1.2102935552631580
H	1.1055696031578945	1.3461710410526315	0.45236651105263165	H	1.1347307868421055	1.4347594473684211	0.90799487473684215
O	-2.3500373268421053	1.8657425410526316	-3.9413050789473685	O	-2.1232419131578948	2.0230297473684211	-4.0893159552631575
H	-1.8327884268421053	1.6999668410526316	-4.7418689789473678	H	-1.3430291131578946	2.2383248473684212	-4.6218041552631579
H	-1.8515216268421051	1.2842954410526315	-3.3293603789473680	H	-1.6414975131578946	1.3015817473684212	-3.5876730552631577
O	1.1830552031578947	0.57240772105263160	-3.8183592789473684	O	1.6108212868421055	1.4207367368421098E-002	-3.9148128552631576
H	0.54141392315789461	0.33421180505263159	-3.1719307789473685	H	0.87664423684210546	-7.3199432631578909E-002	-3.2560641552631577
H	0.93253422315789458	-0.10552837894736838	-4.5133388789473683	H	1.1989971768421055	-0.47885037263157887	-4.6540508552631579
O	-0.18611615684210536	-1.1986659589473685	-5.5495008789473683	O	0.66715078684210538	-3.1119117526315789	-3.5933085552631581
H	-5.2523236842105359E-002	-1.1207335589473684	-6.5140918789473679	H	0.74801521684210548	-3.7959191526315785	-4.2911895552631583
H	-0.94927402684210538	-0.66919307894736835	-5.3500157789473679	H	0.54069725684210546	-2.3272894526315788	-4.1940122552631580
O	-4.8422464268421059	-1.0513428589473686	-1.2748771789473683	O	-0.96988451315789459	-3.9236019526315791	-1.7046823552631578
H	-4.2128805268421052	-0.37427621894736846	-1.3719163789473683	H	-0.17682434315789458	-4.4143349526315792	-1.3067843552631579
H	-5.0070291268421059	-1.2359486589473685	-0.30772840894736841	H	-0.49361162315789459	-3.3886511526315788	-2.3851536552631578
O	0.83207661315789472	-2.9803868589473681	-3.3317734789473685	O	-0.10941552315789453	-1.4534611526315788	-5.5059598552631579
H	0.90859994315789461	-3.7477435589473682	-3.9631640789473686	H	0.19325563684210545	-1.2551499526315788	-6.4083230552631578
H	0.64631579315789467	-2.2839866589473683	-3.9793557789473679	H	-0.84726421315789469	-1.9894041526315789	-5.7292455552631578
O	-0.94988752684210520	-3.9151845589473684	-1.3749043789473683	O	1.4114168868421053	3.5180659473684210	-1.3547506552631579
H	-8.6914106842105376E-002	-4.2386759589473684	-1.0589089789473685	H	2.3115610868421057	3.4944706473684213	-1.7664590552631578
H	-0.67147959684210545	-3.2899581589473681	-2.0482784789473687	H	1.1820848768421055	2.5871927473684213	-1.1434600552631580

C	-1.1626232710526316	0.10142723457894731	-1.2924444784210525	C	-1.1105803568421053	2.8749371263157825E-002	-1.2855436984210524
C	-2.1237459710526316	0.79984402457894732	-0.52414887842105262	C	-2.1180364568421051	0.70341752126315782	-0.56268734842105239
C	-1.9734787710526316	0.47885654757894730	0.82812555157894741	C	-2.0052810568421049	0.51224431126315784	0.78191132157894749
O	-0.87918487105263154	-0.37940380542105273	0.97303838157894740	O	-0.89968135684210526	-0.29860538873684217	1.0779962215789476
C	-0.31752370105263161	-0.66602106542105277	-0.28390097842105261	C	-0.30418419684210518	-0.64268466873684227	-0.26026751842105250
O	-3.2501706710526315	1.4336541345789473	-0.93879937842105265	O	-3.2167824568421053	1.3555176712631578	-1.1222916984210525
O	-2.5339515710526315	0.87743540457894731	1.8756435215789475	O	-2.7095804568421049	0.86357262126315781	1.7142381015789476
C	1.1883011789473683	-0.37096819542105269	-0.23072304842105268	C	1.1624503731578948	-0.24636188873684220	-0.21120329842105251
O	1.4049259789473685	1.0869754045789473	-0.15380150842105261	O	1.3270908031578950	1.1429231912631579	-2.9667778421052504E-002
O	-0.97061837105263171	2.3763834578947318E-002	-2.5321547784210527	O	-1.0486964568421051	-8.4359698736842209E-002	-2.5536543984210525
C	1.8553622289473684	-1.0409626654210526	0.85611045157894727	C	1.8847121431578948	-0.97747542873684212	0.90032651157894761
O	3.2072066289473682	-0.68562176542105280	0.88733467157894741	O	3.3124086431578950	-0.66445405873684227	0.79076484157894744
H	-3.0959154710526318	2.0568609345789475	-1.6782858784210524	H	-3.0153674568421049	1.5820973712631579	-2.0653060984210527
H	-0.42537999105263169	-1.7650400654210527	-0.50398547842105268	H	-0.34222483684210514	-1.7047538287368422	-0.42024900842105245
H	1.4122278989473682	-0.74895436542105265	1.7900741215789475	H	1.4652105431578950	-0.75355272873684220	1.8796026015789475
H	1.7915401289473685	-2.1216098654210525	0.78128470157894736	H	1.7072762431578949	-2.0334046287368421	0.91019302157894755
H	3.2704374289473686	0.27432384457894732	0.61850385157894738	H	3.3762606431578948	0.27213119726315782	0.84472471157894757
H	1.6649898289473684	-0.64966026542105282	-1.1794694784210527	H	1.5818086431578946	-0.50796040873684223	-1.2213478984210524

H	0.95319705315789482	1.4529594712631577	0.83246141157894749	H	0.48386846105263159	1.4784034161052630	0.79923059631578952
O	-1.9651600568421053	1.8762470712631578	-4.1332786984210523	O	0.61679800105263161	-2.3164082838947366	-3.0709847336842104
H	-1.2309722568421053	1.9570660712631578	-4.8430844984210530	H	0.61265666105263161	-2.2541794838947369	-4.0217933336842098
H	-1.5492457568421050	1.1161943212631578	-3.6450713984210528	H	-0.10098507894736838	-1.7230028838947367	-2.8057453336842104
O	1.6530386431578949	0.45073815126315780	-4.0109083984210523	O	1.3732986710526316	0.52643335610526321	-4.0332613336842105
H	0.92771897315789487	0.70205845126315780	-3.5387814984210526	H	0.65899877105263160	0.44602521610526324	-3.3398194336842106
H	1.1838409331578950	0.22280063526315780	-4.8180907984210526	H	0.94560096105263158	-2.1096113894736812E-002	-4.7304683336842102
O	0.51282423315789483	-2.8049301287368422	-3.4055526984210527	O	-2.0182844289473683	2.1187226161052632	-4.1811253336842098
H	0.36387868915789479	-3.3367667287368423	-4.2018471984210528	H	-1.5852497289473684	1.9839844161052631	-5.0456306336842101
H	0.28266377315789482	-1.9126049287368421	-3.7114360984210526	H	-1.8758729289473681	1.2711388661052632	-3.7165616336842109
O	-8.1911466842105207E-002	-0.81460742873684211	-5.5361218984210527	O	-0.18739480894736840	-1.2095018838947369	-5.7758858336842103
H	-6.5298716842105198E-002	-1.0414450287368422	-6.5049497984210527	H	0.41480640105263161	-1.1849914838947369	-6.5332286336842103
H	-0.98708405684210521	-1.0766255287368423	-5.3158318984210524	H	-0.77954852894736837	-1.9108122838947370	-6.0573387336842099
O	-1.1456828568421051	-3.5907820287368422	-1.3676871984210524	O	-1.1251556289473683	-3.9633763838947371	-1.3822247336842106
H	-0.36084206684210518	-3.9695942287368422	-0.95408179842105234	H	-0.51078998894736838	-4.3340189838947367	-0.73670113368421053
H	-0.73477785684210517	-3.2116948287368423	-2.1824224984210527	H	-0.49887315894736839	-3.7067276838947367	-2.0945452336842107
O	-4.7490973568421051	-1.0195079287368423	-1.1750252984210525	O	-3.1022103289473684	-0.10380448389473679	-6.4630320336842102
H	-4.1398051568421055	-0.25536856873684222	-1.2921918984210525	H	-3.0559219289473685	-1.0607884838947368	-6.7861798336842103
H	-4.5310349568421051	-1.3683484287368421	-0.25642236842105254	H	-2.2980320289473681	-0.24797824389473683	-5.8689432336842104

C	-1.1673551357894734	4.2232692736842103E-002	-1.2648167878947367	C	-1.2361543794736840	0.15174560684210536	-1.2726318903157896
C	-2.2737344357894735	0.64471771273684220	-0.58253911789473678	C	-2.2188476794736842	0.73187005684210538	-0.53592713031578953
C	-1.9627537357894735	0.71934359273684212	0.79171685210526332	C	-1.9990406794736837	0.63877578684210534	0.85102219968421045
O	-0.92294413578947343	-0.16577337726315788	1.0722931621052632	O	-0.95334297947368385	-0.23008719315789466	1.0108120396842106
C	-0.33532247578947344	-0.59072990726315777	-0.13775152789473671	C	-0.36772121947368391	-0.62952888315789468	-0.26734520031578940
O	-3.3862533357894735	1.1708506027368422	-1.1978755878947367	O	-3.2781776794736843	1.3890831468421054	-1.0267997903157895
O	-2.6437711357894735	1.2894865627368421	1.6743474121052633	O	-2.5140274794736843	1.2114116468421054	1.8314212096842104
C	1.1764554742105267	-0.26656274726315793	-0.14968290789473676	C	1.1370424105263159	-0.23183947315789460	-0.23392677031578940
O	1.3950662642105267	1.1381955827368422	1.3278702105263274E-002	O	1.2715843505263160	1.0989747268421053	0.11967265968421056
O	-0.89721673578947336	-4.5746917263157882E-002	-2.4818652878947365	O	-1.0930966794736838	9.7361396842105374E-002	-2.5564960903157896
C	1.9988795642105266	-0.99269307726315792	0.91899302210526324	C	1.9632498205263160	-1.1990199831578947	0.67765913968421054
O	3.3896308642105266	-0.67526404726315792	0.90173961210526321	O	3.3515383205263158	-0.89345988315789460	0.75887696968421059
H	-3.2270236357894735	1.2548834527368422	-2.1793493878947365	H	-2.9446311794736841	2.1708235168421055	-1.5112216903157896
H	-0.48454757578947344	-1.6652953772631578	-0.26981101789473672	H	-0.48610091947368400	-1.7455844831578948	-0.30447595031578939
H	1.6035571642105266	-0.88385507726315793	1.9240054121052634	H	1.4893963205263161	-1.1530162831578947	1.6993536096842106
H	2.0438468642105265	-2.0290650772631582	0.72784307210526333	H	1.9453300205263160	-2.2241294831578946	0.33987976568421058
H	3.4011515642105268	0.23898748073684212	0.71303459210526321	H	3.4349048205263157	5.7491316842105400E-002	0.60447237968421064
H	1.5436488642105266	-0.53860719726315787	-1.1384213878947369	H	1.4545459205263160	-0.44630886315789459	-1.2815005903157894
H	0.74868571421052654	1.3548951227368422	0.66486117210526330	H	1.0435488905263159	1.2054373268421055	1.0971551296842106
O	-2.1090235357894733	1.7632422227368423	-4.1185348878947368	O	-2.3267420794736839	2.0936360168421055	-4.0458906903157894
H	-1.4464491357894733	1.9817775227368422	-4.7940338878947371	H	-1.8252979794736841	2.0920771168421055	-4.8529908903157892
H	-1.6774081357894732	0.95733280273684218	-3.6684575878947365	H	-1.8135626794736839	1.4000960368421054	-3.5665316903157893
O	0.40359836031578954	-2.4674939772631581	-3.0961030878947366	O	0.50945594052631604	-2.1742755831578946	-3.3324338903157895
H	0.21515402421052657	-2.9291079772631581	-3.9368344878947363	H	0.62472034052631598	-1.9129731831578949	-4.2472842903157897
H	4.6463054210526544E-002	-1.5620194772631579	-3.2863161878947365	H	2.2859305263160379E-003	-1.3755816831578946	-3.1089077903157896
O	1.5448031642105266	0.68723937273684210	-3.8945954878947369	O	1.4761705205263160	0.81498942684210540	-3.9527758903157899
H	0.90733307421052656	0.35687928473684211	-3.2282620878947368	H	0.73726441052631597	0.90271751684210533	-3.3236234903157893
H	1.0788663242105265	0.48201497273684213	-4.6982391878947363	H	1.0616504405263161	0.49949758584210541	-4.7793631903157898
O	-1.1022613357894734	-3.6757405772631579	-1.1518571878947368	O	-2.8281579794736840	3.6154246842105386E-002	-6.3552256903157893
H	-0.27194088578947345	-4.0533517772631580	-0.74551778789473666	H	-2.7571218794736843	-0.91538478315789473	-6.5696536903157892
H	-0.70852893578947329	-3.0069807772631578	-1.6964011878947365	H	-2.0671292794736842	0.19899619684210540	-5.8328113903157899
O	-0.23864031578947348	-0.98784477726315778	-5.6655807878947364	O	-0.34505781947368391	-0.89341128315789464	-5.7721842903157894
H	0.18639908421052656	-1.0822727772631577	-6.5519397878947370	H	1.1321880526316053E-002	-0.90351738315789465	-6.6691835903157894
H	-0.87933563578947349	-1.6920392772631578	-5.7091069878947369	H	-0.95416587947368381	-1.6653833831578948	-5.7024172903157897
O	1.3915794642105266	3.5413191227368421	-1.5702978878947369	O	-4.8105790794736842	-1.4540461831578946	-4.2914432903157893
H	2.3571531642105263	3.7312249227368421	-1.7959085878947367	H	-4.1560908794736839	-1.3797560831578948	-3.5511984903157896
H	1.4188803642105265	2.6006547227368420	-1.3603773878947369	H	-5.0587243794736843	-0.52798992315789461	-4.4912184903157897

C	-1.1968380289473683	-0.15076609389473677	-1.2692543336842106	C	-1.1883208789473685	1.5547694210526319E-002	-1.2598408291578946
C	-2.2245262289473682	0.48353396610526322	-0.68509553368421050	C	-2.2208396789473683	0.72334091421052626	-0.56705166915789484
C	-2.0576342289473684	0.36125619010526322	0.69916715631578952	C	-1.9971241789473686	0.62051086421052637	0.84490201084210526
O	-0.97207502894736841	-0.44069345389473674	1.1079404763157896	O	-0.88423637894736862	-0.18494857578947366	1.0131059008421053
C	-0.32461535894736837	-0.75596778389473673	-0.14859272368421050	C	-0.39916825894736846	-0.65234584578947363	-0.23258406915789476
O	-3.2414240289473684	1.2136085361052633	-1.3218575336842107	O	-3.2730893789473683	1.4779278542105265	-1.0170718291578946
O	-2.6904908289473681	0.86543608610526324	1.6084431663157894	O	-2.5842373789473685	1.0770858342105263	1.8183796708421054
C	1.0964538710526317	-0.15641402389473680	-0.13665488368421047	C	1.0857846310526316	-0.33390225578947363	-0.26799325915789474
O	1.1022085310526317	1.3420059161052631	8.6274996315789554E-002	O	1.3292627210526315	1.0206460342105264	0.17411395484210523
O	-0.88757642894736832	-0.25235883389473673	-2.5283061336842105	O	-0.88627597894736843	2.0035524210526345E-002	-2.5200906291578948
C	1.9524965710526316	-0.82557778389473691	0.90936894631578957	C	1.9726196210526314	-1.2110323457894738	0.62829022084210528
O	3.4049862710526315	-0.69335848389473687	0.77131310631578953	O	3.3220717210526316	-0.72335014578947365	0.58394813084210528
H	-2.8506338289473683	1.9217305161052631	-1.8280130336842104	H	-2.9936907789473683	2.3680102542105264	-1.2548460291578947
H	-0.19880289894736838	-1.8862662838947371	-0.24031340368421050	H	-0.54426725894736849	-1.7419055457894737	-0.22926654915789474
H	1.6376475710526317	-0.44102873389473679	1.8837406663157896	H	1.5669571210526314	-1.1730239457894736	1.6412778708421052
H	1.7193855710526318	-1.8745567838947368	0.98506951631578954	H	1.9828483210526315	-2.2874629457894740	0.35652422084210522
H	3.7149389710526317	0.17717752610526322	0.46724898631578954	H	3.3231506210526316	0.24196106421052632	0.54928841084210522
H	1.5326310710526316	-0.36616389389473675	-1.1597100336842106	H	1.4296631210526314	-0.34811021578947365	-1.3251157291578948

H	0.95889227105263153	1.0910157842105264	1.0640302008421052
O	1.4215743210526315	0.59596510421052629	-3.8063044291578945
H	0.62060853105263147	0.44661299431052631	-3.3450796291578948
H	1.1616549710526316	0.19657326421052632	-4.6630469291578951
O	-2.3965719789473683	2.0001295542105262	-4.0897861291578952
H	-1.7924080789473684	2.1479266542105262	-4.8754434291578947
H	-1.8363694789473686	1.2531087042105264	-3.7279919291578949
O	0.28813431105263154	-2.3803627457894736	-3.4733507291578949
H	0.36948674105263152	-2.7577098457894738	-4.4038138291578948
H	-0.27527785894736850	-1.5987114457894740	-3.5948697291578946
O	-0.87904587894736852	-3.9167649457894740	-1.6094615291578946
H	-0.16465885894736848	-4.4239828457894737	-1.1141925291578947
H	-0.31248157894736844	-3.3129056457894737	-2.2052315291578948
O	3.0212073210526316	-2.0013765457894737	-2.6400752291578948
H	2.0366388210526316	-2.0365063457894736	-2.8984466291578945
H	3.3774028210526317	-2.6736234457894739	-3.2548255291578947
O	-0.31755026894736843	-0.97167434578947376	-6.0496680291578953
H	0.26025122105263154	-1.1778879457894735	-6.8029033291578953
H	-0.84006937894736855	-1.8291525457894737	-6.0452050291578949

C	-1.1574746600000001	7.8868878947368326E-002	-1.2889621252631578
C	-2.1267072600000003	0.81466682894736842	-0.59708980526315791
C	-1.8787620600000001	0.69960400894736829	0.81931491473684215
O	-0.88971266000000027	-0.26309466105263168	1.0309619847368421
C	-0.33402251000000011	-0.65138812105263166	-0.25157141526315785
O	-3.2727036600000003	1.3785093189473683	-1.0654763252631578
O	-2.4294979600000000	1.2612182689473683	1.7777999747368420
C	1.1264587199999998	-0.30281572105263166	-0.20382847526315784
O	1.3706991399999999	1.0878086489473682	3.9868694736842125E-002
O	-1.0185973600000002	-3.2841461052631638E-002	-2.5179119252631579
C	1.8161652399999999	-1.1069021210526317	0.84048732473684218
O	3.2918286399999999	-0.83192312105263155	0.74466732473684205
H	-3.1388962600000001	1.9070782789473681	-1.9043840252631581
H	-0.50200967000000007	-1.6807238210526316	-0.37702666526315787
H	1.4762196399999998	-0.90828142105263165	1.8767972747368422
H	1.6954473399999999	-2.2206832210526319	0.85406719473684212
H	3.3634858399999996	9.8605108947368347E-002	0.41488344473684213
H	1.5706587399999998	-0.59444222105263156	-1.1696546252631579
H	1.0374207599999998	1.2667365489473683	0.97705725473684213
O	1.3169611499999998	0.67811690894736831	-3.5244284252631579
H	0.48370666999999989	0.46313622094736834	-3.0513104252631580
H	1.1712692199999999	0.18901367894736831	-4.3459069252631579
O	-2.4710345600000001	1.8401680789473684	-4.1658447252631579
H	-2.0187677600000002	2.0341175789473684	-4.9729655252631577
H	-1.7656071600000001	1.2442288789473683	-3.7984215252631581
O	0.62811213999999982	-2.4319755210526317	-3.3301329252631580
H	0.68548638999999989	-2.8927778210526318	-4.1819526252631576
H	-0.12330694000000009	-1.7984555210526318	-3.5015016252631579
O	-4.3413953599999999	-1.1524463210526315	-1.3380312252631579
H	-4.1244604599999999	-0.19781587105263165	-1.3480902252631579
H	-4.6470741599999998	-1.2524984210526315	-0.38685052526315789
O	-0.41691468000000015	-1.0761912210526319	-5.9300384252631577
H	0.11263939999999989	-0.83129092105263169	-6.6999523252631583
H	-0.92354416000000006	-1.8094089210526318	-6.3077878252631585
O	-0.81239206000000008	-4.0085238210526315	-1.5873684252631579
H	-0.28658927000000006	-4.7756628210526317	-1.3261129252631578
H	-0.19754383000000014	-3.6419980210526322	-2.2202038252631580

C	-1.1616903026315790	0.23001786421052633	-1.2129472515789472
C	-2.2144753026315791	0.80053581421052633	-0.52674329157894739
C	-1.9732773026315789	0.74259000421052634	0.88448309842105277
O	-0.88691520263157897	-0.15542020578947369	1.1516560584210527
C	-0.36550574263157892	-0.53260287578947374	-0.20245676157894726
O	-3.2649740026315790	1.5112513742105262	-0.95697205157894727
O	-2.4852523026315789	1.2670607442105264	1.8895495484210527
C	1.1106156473684210	-0.28644918578947365	-0.26328800157894733
O	1.5273556973684210	1.0121744442105265	0.14195775842105268
O	-0.96775600263157902	0.12944504421052633	-2.4552263515789474
C	1.8550146973684210	-1.3771684757894738	0.61886901842105269
O	3.2444955973684211	-0.93443037578947374	0.86863932842105274
H	-2.9934762026315789	1.9953026242105265	-1.7593916515789474
H	-0.59177605263157895	-1.5962340757894737	-0.32688532157894729
H	1.3066310073684211	-1.5757843757894734	1.5741627484210527
H	1.8194243973684210	-2.2365046757894733	-4.0121141578947306E-002
H	3.3240847973684211	5.4368794210526317E-002	0.79567520842105277
H	1.4853176973684210	-0.31495254578947363	-1.2288177515789473

H	1.2321588773684211	1.2668000842105265	1.0478568084210527
O	-2.5158060026315789	1.7469040242105263	-4.0112285515789479
H	-1.8776483026315791	1.9634215242105264	-4.7595793515789477
H	-2.0445314026315788	1.0083474742105263	-3.5896268515789473
O	1.5112993973684210	1.1230486042105263	-3.5332382515789473
H	0.65274292736842110	0.79734589421052637	-3.2501089515789476
H	1.4563248973684211	0.94360594421052635	-4.4943947515789473
O	0.91751153736842106	-2.3332972757894734	-3.4725204515789474
H	1.0527584173684210	-2.8276587757894736	-4.3283088515789476
H	7.3349457368421023E-002	-1.7840756757894733	-3.5934224515789475
O	-4.1856813026315782	-1.3879365757894737	-1.5165012515789473
H	-3.5300846026315789	-0.67770617578947367	-1.4371769515789472
H	-4.2631004026315784	-1.7062051757894734	-0.51251845157894738
O	-0.57089099263157905	-0.70968077578947375	-5.8842916515789474
H	0.16065573736842104	-0.89233777578947360	-6.4668650515789476
H	-0.99443910263157909	-1.5692846757894734	-5.9313697515789476
O	-0.31923776263157894	-3.8794346757894735	-1.4800561515789472
H	0.24015246736842105	-4.6645477757894742	-1.4043511515789473
H	0.15950069736842104	-3.3634321757894736	-2.1855255515789476

C	-1.1429350810526315	0.21973353131578943	-1.2948742791052630
C	-2.0666077810526318	0.98795316131578947	-0.59674545910526322
C	-1.8987734810526316	0.78160281631578943	0.74839656089473683
O	-0.89351348105263151	-0.15852712868421059	0.94409393089473681
C	-0.24921229105263143	-0.50960506868421063	-0.32245960910526317
O	-3.1391448810526317	1.5650442613157893	-1.0875602791052630
O	-2.4407888810526313	1.2691313313157895	1.7675004208947369
C	1.2209719589473687	-0.27902496868421056	-0.13254531910526315
O	1.4291890189473686	1.0838300913157894	0.29925751089473684
O	-1.0867387810526314	5.1235071315789416E-002	-2.5719771791052630
C	1.8106690189473684	-1.3605277686842108	0.77930327089473683
O	3.1811922189473689	-0.94943776868421059	0.96630299089473681
H	-3.0180185810526314	1.7238273413157894	-2.0198589791052632
H	-0.50644829105263145	-1.5730638686842107	-0.42741582910526321
H	1.2546575489473684	-1.5301328686842104	1.6781656208947369
H	1.8331619189473685	-2.2998676686842106	0.27562666389473683
H	3.1772363189473687	4.6677261315789420E-002	1.0545580408947368
H	1.7407773189473685	-0.32674186868421051	-1.0692715791052632
H	0.79432620894736850	1.2578941113157893	1.0095035008947368
O	0.51842244894736855	-2.0170346686842104	-3.4199875791052632
H	0.45308100894736852	-2.3762373686842104	-4.3189352791052631
H	-0.21702195105263145	-1.3673425686842107	-3.3604022791052630
O	-2.3670833810526313	2.0126630313157894	-4.3033201791052633
H	-1.4819633810526314	2.1662739313157893	-4.7191072791052635
H	-2.2344284810526318	1.1473260913157894	-3.8423201791052630
O	1.6382415189473685	1.4585006813157895	-3.3664754791052629
H	0.84567448894736852	1.8729733313157895	-2.9708570791052629
H	1.1956507389473685	0.72888736631578943	-3.8259667791052632
O	-4.3741818810526318	-1.2604720686842106	-1.6669308791052631
H	-3.7555070810526319	-0.53659356868421049	-1.7416988791052630
H	-4.4176612810526317	-1.2587055686842106	-0.74003590910526318
O	-0.69016409105263143	-3.7094359686842102	-1.5580666791052631
H	-7.9202941052631481E-002	-4.4702942686842109	-1.3464976791052632
H	-0.10341280105263145	-3.2107414686842106	-2.1142403791052633
O	-0.87309368105263152	-0.31559806868421059	-6.2117680791052630
H	-0.22391161105263147	-0.34033616868421057	-6.9334474791052632
H	-1.1802866810526316	-1.2378902686842106	-6.2497517791052628

C	-1.2014268694736843	0.13559711878947356	-1.2269390452631579
C	-2.1975634694736841	0.77381503078947356	-0.51507410526315778
C	-2.0065409694736842	0.67077475978947354	0.90402204473684211
O	-0.84815915947368414	-3.8195171210526402E-002	1.0780849547368421
C	-0.32205012947368417	-0.53672848121052641	-0.15514648526315783
O	-3.3274044694736844	1.4567832287894737	-0.99678774526315794
O	-2.6820064694736843	1.1525387387894737	1.8524693547368420
C	1.1675439305263158	-0.25660963121052638	-0.21330507526315784
O	1.4326251305263158	1.0758398587894735	0.31392029473684213
O	-0.97866386947368422	0.18144525878947360	-2.4607905452631580
C	1.9612878305263157	-1.3527714812105263	0.56206510473684212
O	3.3047659305263157	-0.88711918121052635	0.77598937473684226
H	-2.9745257694736842	2.1661495187894735	-1.5329242452631580
H	-0.62723617947368415	-1.6180633812105265	-0.14245623526315782
H	1.5252534305263159	-1.6572098812105263	1.5300083547368422
H	2.0306692305263159	-2.1847274812105266	-0.17662351526315784
H	3.4441085305263157	5.9786638789473545E-002	0.56654870473684216
H	1.4195192305263158	-0.27656939121052637	-1.2756856452631580

H	0.87980411052631580	1.1352639287894735	1.1126244547368420
O	1.2499469305263158	1.3086098287894736	-3.8066050452631579
H	0.61083271052631583	1.2497847787894736	-3.0667725452631580
H	0.73896912052631580	0.82094207878947356	-4.5052838452631576
O	-2.7038301694736844	2.2025177187894736	-4.0131190452631582
H	-2.2093010694736841	2.3027795187894737	-4.8443820452631581
H	-2.2507274694736843	1.4045591187894737	-3.7255206452631580
O	0.35147168052631583	-2.2979268812105262	-3.4432966452631577
H	0.59109907052631583	-2.5969254812105262	-4.3658655452631576
H	-0.32332296947368422	-1.6462764812105264	-3.6309000452631577
O	-4.3193615694736840	-1.1928510812105263	-1.4765018452631580
H	-3.9148800694736838	-0.29707846121052639	-1.4030689452631580
H	-4.4044813694736842	-1.4527827812105265	-0.50797870526315791
O	2.8992013305263158	-1.6695043812105266	-2.8657509452631578
H	1.9277222305263157	-1.7804343812105263	-2.9473283452631578
H	3.1692758305263160	-2.1942598812105265	-3.6037523452631577
O	-1.0117310694736843	-0.48442068121052650	-6.3406772452631577
H	-0.25806943947368421	-0.40599178121052648	-7.0049699452631575
H	-1.4164283694736841	-1.3766838812105262	-6.6393361452631581

C	-1.1938274405263156	0.21150079310526326	-1.2863461184210527
C	-2.1815147405263158	0.74245562310526325	-0.52555755842105267
C	-1.9996967405263157	0.52762646410526326	0.92567940157894735
O	-0.86607211052631583	-0.33584130689473668	1.0556766815789473
C	-0.31838659052631579	-0.61990651689473664	-0.31273028842105260
O	-3.2890631405263160	1.5877265331052632	-0.80767443842105269
O	-2.5504640405263159	1.0365538731052633	1.8914687815789473
C	1.1379669594736843	-0.32672906689473669	-0.27839226842105258
O	1.4929716594736844	0.99768639310526330	0.15874263157894739
O	-0.97427135052631575	0.25880634310526329	-2.5392853184210522
C	1.8805680594736844	-1.2719219168947367	0.66182614157894737
O	3.2917798594736842	-0.94478891689473676	0.83653983157894740
H	-3.0591439405263161	2.2557701831052634	-1.4779156184210527
H	-0.47469890052631580	-1.6815414168947369	-0.44531860842105259
H	1.3881951594736843	-1.0471476168947367	1.6625509815789474
H	1.7779482594736844	-2.2728363168947370	0.37915376157894742
H	3.5496872594736839	-3.4596026894736731E-002	0.63159341157894733
H	1.5641312594736843	-0.36335060689473675	-1.3152347184210527
H	0.82389051947368419	1.2805335031052634	0.78522331157894731
O	0.11775488647368425	-1.9983567168947369	-3.3594461184210522
H	-0.35780303052631579	-2.4635695168947365	-4.0365665184210524
H	-0.47989095052631581	-1.2315762168947366	-3.1299164184210526
O	-2.4912315405263157	2.2315734831052634	-3.8996736184210525
H	-2.0939328405263158	2.2009984831052636	-4.7529965184210523
H	-1.8824134405263158	1.4968655531052633	-3.4875174184210525
O	1.2572028594736844	1.6729896831052633	-3.4048551184210525
H	1.2486469594736844	2.1278542831052634	-2.5530849184210522
H	0.42235364947368426	1.1884126331052633	-3.4116229184210525
O	-4.7842202405263157	-0.66962131689473670	-1.5104127184210527
H	-4.2303227405263160	0.16144292310526326	-1.3438596184210527
H	-4.5399560405263157	-1.1686474168947367	-0.68184792842105268
O	2.7540347594736843	-1.8756219168947366	-2.7181700184210524
H	1.7702879594736842	-1.9312602168947366	-2.8036517184210523
H	3.0709326594736841	-2.4891008168947364	-3.3968039184210523
O	-1.1988705405263156	-0.65610591689473674	-6.1594807184210527
H	-0.24325390052631576	-0.79846271689473669	-5.9169256184210521
H	-1.6277551405263158	-1.5058909168947368	-6.3594275184210520

C	-1.2100130442105264	0.15935187184210514	-1.2657840923157895
C	-2.0296286442105265	0.91087107184210514	-0.43826843231578949
C	-1.7968198442105263	0.68635713684210520	0.93253488768421056
O	-0.73328904421052621	-0.22769927815789481	1.0184236776842106
C	-0.31576403421052623	-0.57232551815789479	-0.32762306231578947
O	-3.1951163442105264	1.5256309618421051	-0.87490073231578946
O	-2.3329261442105262	1.0818637618421052	1.9557616076842106
C	1.1741116557894737	-0.21149118815789480	-0.29229690231578948
O	1.4319507557894737	1.1190217118421051	0.14651956768421051
O	-1.1527307442105263	0.14699917184210509	-2.5383238923157898
C	1.7564679557894736	-1.3186691181578949	0.59442042768421055
O	3.2133118557894735	-0.92085571815789491	0.94433702768421057
H	-3.0143337442105262	2.0392809818421052	-1.7453826923157894
H	-0.46587066421052625	-1.6608240181578950	-0.27405726231578947
H	1.3352502557894737	-1.4784305181578947	1.6095517076842105
H	1.8639817557894736	-2.2348876181578947	4.4426391684210524E-002
H	3.1471424557894738	3.7142891842105175E-002	0.93640199768421051
H	1.5580510557894736	-0.22524531815789484	-1.3068872923157895

H	0.76622450578947376	1.1439087318421051	0.88114706768421058
O	0.96252758578947373	1.6292400418421051	-3.4480015923157898
H	0.97249993578947380	2.2354333818421050	-2.6750134923157898
H	0.26283446578947373	1.0095561018421051	-3.1876860923157895
O	-2.5898522442105265	2.0451846818421053	-3.9418984923157896
H	-1.9382539442105262	2.2752146818421051	-4.5850507923157888
H	-1.9784059442105262	1.4803379018421050	-3.3904482923157895
O	0.23318902578947376	-2.2304234181578950	-3.2530831923157897
H	0.23267779578947373	-2.7189092181578949	-4.0549313923157895
H	-0.41859875421052628	-1.5093511181578947	-3.4655307923157896
O	-4.7731058442105256	-0.55155781815789484	-1.6329503923157895
H	-4.2830798442105262	0.32531230184210513	-1.6156372923157893
H	-4.3996792442105255	-0.99749061815789486	-0.84355279231578939
O	-0.78406237421052627	-0.77335671815789486	-6.3097361923157894
H	9.0899123789473735E-002	-0.60095971815789495	-5.9156400923157895
H	-0.80979944421052630	-1.7673823181578947	-6.3455998923157892
O	-1.2834689442105263	-3.8804648181578951	-1.6024682923157894
H	-1.3002391442105263	-4.7949833181578949	-1.9565956923157894
H	-0.40335149421052630	-3.5172305181578949	-1.8830234923157894

C	-1.2705279415789474	6.1402099105263175E-002	-1.2147555595421053
C	-2.0568844415789473	0.94512612910526328	-0.44938792954210527
C	-1.9413301415789475	0.63255054610526318	0.96044191045789473
O	-0.92402520157894752	-0.41201807089473685	1.0718474404578946
C	-0.39876663157894748	-0.71201721089473680	-0.25008367954210525
O	-3.0951355415789474	1.7729858891052630	-0.89833834954210523
O	-2.4886079415789473	1.0790309691052631	1.9057582404578945
C	1.0889695584210526	-0.33312596089473678	-0.24750240954210523
O	1.3326014584210526	1.0093310091052632	6.8582350757894747E-002
O	-1.2962503415789475	-0.12693950089473682	-2.5069666595421052
C	1.9320976584210525	-1.2824645108947368	0.58312245045789479
O	3.2892784584210526	-0.74950661089473669	0.66962737045789478
H	-2.8131710415789475	2.7136945891052631	-0.80921548954210520
H	-0.39803983157894746	-1.7975089108947371	-0.42484577954210523
H	1.5777956584210526	-1.4115054108947369	1.5899529404578949
H	1.8873268584210525	-2.2350842108947369	3.3123731457894746E-002
H	3.4176943584210524	0.17540980910526321	0.37556500045789476
H	1.3035642584210525	-0.53935041089473679	-1.3021847595421052
H	0.85341078842105256	1.2099897691052632	0.84525918045789472
O	0.37066626842105249	-2.2435371108947368	-3.0817307595421055
H	0.29871378842105251	-2.5538628108947368	-4.0275115595421056
H	-0.30136636157894747	-1.5267221108947369	-3.0146143595421053
O	0.96376394842105251	1.3719817591052632	-3.3832544595421052
H	0.93319536842105255	2.1037941891052632	-2.8086182595421052
H	0.11792139842105251	0.91726551910526322	-3.2427904595421055
O	-2.5178554415789476	1.9886612891052632	-4.2856917595421056
H	-1.9111937415789475	1.8902766891052631	-5.0296334595421053
H	-2.2311981415789477	1.2333868691052632	-3.6727215595421052
O	-1.2303749415789476	-3.9906498108947369	-1.8271610595421053
H	-0.50012230157894755	-4.3774766108947363	-1.3182274595421051
H	-0.67765053157894750	-3.3314068108947370	-2.2549308595421054
O	-3.0774487415789475	-3.7027339108947372	-3.9806138595421050
H	-3.2599743415789475	-2.8213549108947369	-3.6355596595421056
H	-2.5537373415789473	-4.0846872108947361	-3.2433271595421052
O	-4.9709659415789478	-1.2580978108947369	-4.0744927595421050
H	-4.7190988415789477	-0.62998521089473669	-3.3579381595421052
H	-5.2217840415789478	-0.59040311089473685	-4.7132554595421050

C	-1.1826997257894738	0.19738929684210538	-1.1506459187894738
C	-2.0202437257894736	1.0093066768421055	-0.43227335878947371
C	-1.8992578257894737	0.69176894684210533	1.0239803312105265
O	-0.89710658578947367	-0.26530995315789463	1.2247820812105263
C	-0.45241656578947365	-0.66300927315789471	-0.15096626878947370
O	-3.0952237257894737	1.6946676268421053	-0.90378687878947372
O	-2.5226586257894739	1.1458127768421054	1.9725215812105263
C	1.0760776742105262	-0.44413363315789467	-0.32369864878947369
O	1.3519254742105262	0.94438215684210536	-9.9829288789473697E-002
O	-1.0179698257894736	-2.2970413157894609E-002	-2.4405393187894737
C	1.8840567742105263	-1.2440832731578946	0.72989920121052632
O	3.3174958742105263	-0.91398057315789472	0.61507911121052627
H	-2.8325588257894738	2.3577708268421054	-1.5271638187894736
H	-0.72701532578947370	-1.7160088731578949	-0.30707074878947371
H	1.5254101742105264	-0.91927357315789471	1.7688708812105263
H	1.7857539742105264	-2.2862022731578948	0.59337916121052625
H	3.5406205742105263	-0.15476959315789462	8.7024748210526304E-002
H	1.3393791742105263	-0.69759907315789471	-1.3603424187894737

H	0.82643106421052626	1.2862421968421054	0.68077957121052635	H	1.1216338263157895	1.2584470573684212	0.84250576957894729
O	0.54728946421052627	-1.9585313731578946	-3.5101770187894736	O	0.17694169631578940	-1.9842416526315787	-3.4814433304210524
H	2.3866543010526351E-002	-2.4539951731578946	-4.1460764187894732	H	0.13533061331578938	-1.7813589526315787	-4.4749174304210522
H	1.5418356210526351E-002	-1.1442205731578947	-3.2601160187894735	H	-0.50649741368421064	-1.3929277526315789	-3.1333748304210522
O	1.1062499742105263	1.4375972568421054	-3.2667754187894738	O	-2.4965030736842109	1.7967875473684212	-4.3004776304210521
H	0.93277491421052627	1.7050001268421053	-2.3631770187894738	H	-1.9468173736842105	2.0666292473684211	-5.0772381304210521
H	0.21122804421052635	0.88807357684210531	-3.3686109187894737	H	-2.0833569736842108	0.98154564736842120	-3.9020801304210524
O	-2.3359602257894738	1.6944931268421053	-4.0108887187894728	O	0.93881216631578934	1.7413328473684213	-3.3913443304210524
H	-1.7271883257894738	2.0102924268421054	-4.7136060187894735	H	0.42280535631578936	2.2086558473684210	-2.7379676304210525
H	-2.1013311257894736	0.83356327684210529	-3.6565610187894739	H	0.40797672631578935	0.89083713736842118	-3.3071315304210525
O	-1.3293999257894737	-3.6378642731578945	-1.6070063187894736	O	-1.1114332736842105	-3.8788974526315787	-1.9340277304210527
H	-0.80331034578947369	-4.4035196731578949	-1.2793228187894736	H	-0.29102236368421064	-4.2956568526315788	-1.6847814304210527
H	-0.64103797578947375	-3.2424770731578945	-2.2378234187894739	H	-0.78957318368421059	-3.1659017526315791	-2.5212885304210526
O	-4.9594539257894734	-3.8637473157894675E-002	-1.7905941187894736	O	-3.5599484736842109	3.8410178473684211	-2.8126912304210525
H	-4.2491947257894740	0.56713570884210540	-1.4229420187894737	H	-3.0791095736842107	3.1601115473684209	-3.3604368304210523
H	-4.9002881257894737	-0.67416607315789467	-1.0540262187894738	H	-2.7767043736842107	4.2892723473684216	-2.4096592304210525
O	-4.4631599257894736	-1.5566992731578946	-3.8693606187894738	O	-5.0902595736842100	-1.6927033526315787	-4.0553166304210526
H	-4.5977057257894733	-0.81019827315789472	-3.2482174187894737	H	-5.2653987736842103	-0.84107185263157880	-3.5939833304210524
H	-4.7713535257894737	-1.1236001731578946	-4.6839901187894730	H	-4.2702306736842104	-1.4687765526315788	-4.4886237304210521

C	-1.3393267963157893	4.3259744736842132E-002	-1.1348922463684210	C	-1.2348394821052633	0.27965343236842111	-1.2114000608578948
C	-2.1535042963157895	0.85408753473684218	-0.39046064636842104	C	-2.1476203821052633	0.85543756236842117	-0.37567891085789479
C	-1.8595048963157894	0.80147108473684214	1.0320526636315790	C	-1.9653950821052635	0.52657903736842104	0.96740323914210513
O	-0.85739222631578937	-9.9437575263157885E-002	1.2591535536315790	O	-0.89721023210526329	-0.40239692763157886	1.0426679391421052
C	-0.46005408631578937	-0.67761414526315789	-2.5845509368421046E-002	C	-0.43917747210526326	-0.65636956763157883	-0.28858172985789476
O	-3.1254626963157892	1.7618957547368421	-0.83829321636842113	O	-3.1502470821052633	1.7200475323684210	-0.71364712085789483
O	-2.3706714963157896	1.3961620047368422	1.9924105536315790	O	-2.5644426821052630	0.80636067236842113	2.0009069391421055
C	1.0328379036842106	-0.37886847526315781	-0.25148517636842105	C	1.0936722178947367	-0.35702765763157895	-0.31778592085789481
O	1.2511404036842106	0.99989411473684209	-4.3447680368421046E-002	O	1.3374980178947367	1.0213973323684211	-0.18875054555789481
O	-1.2187271963157895	-0.16135778526315792	-2.3677371463684214	O	-1.1417247421052634	0.20288868236842106	-2.4485075608578946
C	1.9788402036842108	-1.2122080452631578	0.58052012363157890	C	1.9596638178947365	-1.1234307676315789	0.79032873914210522
O	3.3155479036842106	-0.88921464526315797	0.23169438363157896	O	3.4671550178947368	-0.81271906763157897	0.62337902914210519
H	-2.7336046963157896	1.8934068547368421	-1.7183226463684209	H	-3.0617006821052630	1.9824790323684212	-1.7035179608578948
H	-0.66547154631578942	-1.7869046452631578	-3.5457848368421044E-002	H	-0.57498452210526330	-1.7782677676315788	-0.45989112085789474
H	1.7936615036842105	-1.0776443452631579	1.6500468536315791	H	1.8209765178947366	-0.88285556763157902	1.9177859391421053
H	1.8431789036842106	-2.2776444452631579	0.38636706363157897	H	1.6901372178947367	-2.2122817676315791	0.60412041914210513
H	3.2968936036842105	8.0299764736842139E-002	0.14903609363157894	H	3.3987901178947371	0.12854260236842108	0.38884168914210515
H	1.2474697036842106	-0.49407267526315790	-1.3526456463684209	H	1.4609107178947367	-0.62963936763157902	-1.2820925608578948
H	1.0241498036842105	1.2244899247368422	0.87730647363157899	H	0.94853871789473676	1.3316025723684211	0.65441955914210514
O	0.52954618368421058	-2.1605293452631580	-3.3016977463684212	O	-2.6929403821052631	1.5922150323684210	-4.2555060608578952
H	0.32155743368421064	-2.5929852452631579	-4.0999756463684207	H	-2.2652138821052632	1.6936249323684212	-5.1241459608578950
H	-4.0000638015789389E-002	-1.3232475452631578	-3.2877665463684211	H	-2.0698760821052633	1.0649767523684210	-3.7150419608578948
O	-2.6751930963157893	1.7312881547368422	-3.9188023463684214	O	0.22382748789473669	-1.8671755676315791	-3.6399206608578947
H	-2.0310180963157891	1.9169495547368420	-4.6244141463684212	H	0.11957617789473671	-2.0262628676315790	-4.5869100608578952
H	-2.2234800963157895	0.97890035473684212	-3.5490680463684212	H	-0.30065812210526333	-1.0781171676315788	-3.4056034608578947
O	0.95298960368421060	1.6544978547368421	-3.0517866463684213	O	0.66778705789473669	1.9279059323684211	-3.6017293608578949
H	0.88646949368421057	2.2928307547368423	-2.3337165463684211	H	0.50723178789473677	2.4604050323684210	-2.8480763608578945
H	0.11138738368421061	1.1445162647368421	-2.8648084463684214	H	0.17964990789473670	1.1603372623684209	-3.3171029608578948
O	-1.3577677963157895	-3.6661158452631577	-1.7923519463684210	O	-1.3685065821052633	-3.5700219676315790	-2.1923477608578947
H	-0.49423388631578935	-4.0679666452631578	-1.5174685463684210	H	-0.63688526210526331	-4.2081742676315788	-1.9917301608578948
H	-1.0352834763157894	-2.9631588452631576	-2.2923583463684212	H	-0.92454449210526324	-2.8011801676315788	-2.5676115608578947
O	-4.6711734963157889	-1.6532088452631579	-4.0133560463684210	O	2.8232174178947367	-1.7488972676315790	-3.2324779608578948
H	-5.0112828963157892	-1.0721236452631580	-3.3189668463684212	H	1.8665737178947366	-1.9408636676315791	-3.3214055608578947
H	-4.1338033963157894	-1.0221380452631579	-4.4431996463684209	H	3.1706549178947370	-2.3600047676315787	-3.9306122608578948
O	-0.51664039631578940	-0.91091714526315792	-6.3926168463684210	O	-3.5428749821052632	4.0010297323684210	-2.9631080608578948
H	0.43830930368421062	-1.1629911452631578	-6.4057992463684208	H	-3.1625956821052630	3.3541457323684210	-3.5570951608578949
H	-1.0174286563157893	-1.7061248452631577	-6.0546225463684209	H	-2.7340996821052630	4.4446542323684213	-2.5775607608578945

C	-1.3137044736842105	0.10704039736842114	-1.1790869304210527	C	-1.2261176194736845	0.24842032652631579	-1.1828643280526314
C	-2.1882215736842108	0.87834244736842115	-0.40397010042105264	C	-2.1781638194736845	0.96552286652631580	-0.40917572805263164
C	-1.8881904736842106	0.72429883736842116	1.0086290695789473	C	-2.1053631194736844	0.49204939252631580	0.93812457194736842
O	-0.82080205368421066	-0.30122875263157883	1.1108411695789473	O	-1.0115682694736845	-0.36747974347368423	1.1139726719473686
C	-0.37398400368421064	-0.65173985263157874	-0.22448392042105264	C	-0.42696654947368440	-0.57399259347368425	-0.21315017805263159
O	-3.2177614736842108	1.6929765473684213	-0.77283230042105266	O	-3.0319270194736845	1.9990620065263158	-0.71329555805263167
O	-2.4370625736842109	1.0970934473684211	2.0275761695789476	O	-2.8877723194736844	0.67525060652631574	1.8895243719473684
C	1.1867247263157894	-0.32611214263157889	-0.35385524042105265	C	1.1002224805263157	-0.28191892347368425	-0.25713231805263159
O	1.2986493263157894	1.0523397773684211	-0.10756563242105263	O	1.4577677805263156	1.0475961165263157	-6.7414050526316133E-003
O	-1.2304437736842104	2.9438157368421147E-002	-2.4472294304210527	O	-0.90361053947368442	0.26671940652631576	-2.4056511280526314
C	1.9433110263157896	-1.1620562526315787	0.65955075957894738	C	1.9589815805263155	-1.1826115934736843	0.66130225194736836
O	3.3098114263157892	-0.81012535263157881	0.80365326957894734	O	3.4024313805263158	-0.99093489347368435	0.48113087194736837
H	-3.1159685736842109	1.7452639473684213	-1.7334559304210526	H	-3.4441746194736842	1.9055715065263157	-1.5782531280526315
H	-0.47651726368421066	-1.7670356526315787	-0.29348274042105260	H	-0.50292834947368448	-1.6502462934736841	-0.42309668805263162
H	1.4011909263157896	-1.0042948526315787	1.5922199695789474	H	1.7390009805263156	-1.1424558934736844	1.7571612719473686
H	1.8854972263157894	-2.1844462526315791	0.29308305957894737	H	1.8774995805263155	-2.2445261934736842	0.35972103194736837
H	3.4770528263157892	0.15326644736842115	0.59198021957894742	H	3.5602768805263154	-3.3413773473684238E-002	0.37934377194736840
H	1.4387849263157895	-0.53146795263157887	-1.4140772304210527	H	1.4262614805263156	-0.42907760347368423	-1.3152001280526315

H	1.1961500805263157	1.2964652765263158	0.92427977194736843	H	0.96692124473684216	1.0501325140526316	1.1486551666315790
O	-2.5267549194736842	1.4631716065263158	-4.3072754280526322	O	1.0347961747368422	1.8274264840526317	-3.3850322333684209
H	-1.9728155194736845	1.5086455065263158	-5.1248855280526318	H	1.0109980747368421	2.5073381840526316	-2.6467012333684212
H	-1.8625441194736845	0.96057667652631573	-3.7130344280526315	H	0.38912579473684211	1.2187669040526314	-3.0145507333684209
O	0.91611018052631565	2.0617924065263158	-3.5556527280526313	O	-2.6545414252631581	1.5328966840526315	-4.4675265333684209
H	1.2207892805263156	2.6304197065263155	-2.8472472280526313	H	-1.9909110252631579	1.4095296840526315	-5.1823200333684207
H	0.13219189052631558	1.5854621065263157	-3.1520271280526315	H	-2.2157631252631580	1.0149084440526317	-3.7658477333684210
O	0.15217617052631560	-2.3158274934736842	-3.4687871280526315	O	0.18007025473684210	-2.0679363159473687	-3.7622555333684211
H	-0.12622213047368441	-2.7784438934736841	-4.2981348280526319	H	-2.2816927263157896E-002	-2.6382382159473687	-4.5499372333684205
H	-5.5556817473684419E-002	-1.3841546934736844	-3.6577344280526316	H	-0.19034991526315789	-1.2347545159473685	-3.9607361333684210
O	-1.3641986194736844	-3.4835383934736841	-1.4970648280526315	O	2.5989562747368420	-1.6884326159473684	-3.0165013333684212
H	-0.70255231947368435	-4.2296175934736837	-1.4079457280526315	H	1.6279621747368422	-1.7334965159473685	-3.1995415333684210
H	-1.0182637094736844	-2.9881190934736841	-2.2804562280526315	H	2.8984165747368422	-2.3696770159473686	-3.6950149333684212
O	2.8224413805263158	-2.0092389934736841	-2.9817553280526314	O	-1.1697986252631578	-4.0141643159473688	-1.4273801333684211
H	1.8641308805263157	-2.1005669934736844	-3.1773097280526317	H	-0.51816049526315788	-4.7181832159473682	-1.5468548333684211
H	3.1591784805263154	-2.7254765934736844	-3.5889806280526315	H	-0.74576775526315786	-3.2970811159473685	-1.9272500333684210
O	-3.3424643194736845	3.7273002065263157	-3.1497504280526316	O	-3.4952204252631578	3.9356730840526315	-3.1360600333684210
H	-3.1874933194736843	2.9674127065263156	-3.7474752280526316	H	-3.0941925252631579	3.1662924840526316	-3.6888816333684211
H	-2.4711379194736844	3.7301352065263158	-2.7304950280526317	H	-2.7431748252631580	4.2764334840526317	-2.5876108333684211

C	-1.1308744505263157	0.14977439789473684	-1.2227864714210526	C	-1.2648816515789472	0.22694536831578943	-1.1330690572105262
C	-2.1596369505263158	0.84030405789473683	-0.55904329142105258	C	-2.2679916515789476	0.80906792731578947	-0.37521545721052629
C	-2.1292377505263160	0.61978509789473679	0.80880483857894736	C	-2.0489892515789476	0.63003612731578951	0.99418554278947358
O	-0.99467146052631583	-0.19265207210526314	1.0955420285789474	O	-0.96561095157894727	-0.23299633268421055	1.1885324427894737
C	-0.35745087052631580	-0.56404830210526313	-0.13042415642105262	C	-0.38365183157894728	-0.61258711268421062	-9.3959110210526331E-002
O	-3.1268616505263158	1.6274373978947367	-1.0278781814210527	O	-3.3781978515789475	1.5150583473157895	-0.78712588721052634
O	-2.9250326505263158	0.98780422789473687	1.7032505285789474	O	-2.6139910515789473	1.1264840673157894	1.9732562427894738
C	1.1276304494736842	-0.27769916210526313	-0.15697438942105263	C	1.1174668484210528	-0.33507215268421059	-0.23039076721052631
O	1.4482941494736843	1.0390541778947369	6.4456888578947366E-002	O	1.3767829484210528	1.1207409773157895	0.13919945278947368
O	-0.92020338052631578	6.8206178947368690E-003	-2.4867548714210526	O	-0.97927545157894724	0.20253875731578944	-2.3343309572105264
C	1.9782726494736842	-1.1215287021052633	0.82736010857894737	C	2.0246992484210526	-1.2320785526842104	0.53981399278947362
O	3.4231687494736840	-0.89448870210526310	0.57060345857894745	O	3.4152309484210526	-1.0526154526842104	5.7930372789473675E-002
H	-3.2415829505263156	1.4241275978947368	-1.9585104714210526	H	-3.0717497515789476	2.0094729473157895	-1.5395546572105263
H	-0.41342913052631580	-1.6033511021052633	-0.28762417142105262	H	-0.50867953157894719	-1.6451833526842106	-0.35128439721052629
H	1.7420403494736842	-0.81408680210526307	1.8608116285789473	H	1.9402191484210527	-0.95306905268421049	1.6111777427894738
H	1.7960890494736841	-2.2281501021052632	0.75000946857894746	H	1.8522695484210527	-2.3246548526842106	0.35862900278947363
H	3.4067038494736841	3.3846497894736871E-002	0.45764758857894733	H	3.5668277484210527	-0.14465228268421054	0.32081741278947362
H	1.4843611494736841	-0.52929402210526311	-1.1429366714210525	H	1.2775543484210528	-0.35591394268421056	-1.3040620572105261
H	0.99242084947368414	1.4963448978947369	0.83444613857894745	H	0.91196818842105265	1.2484785673157894	0.96545014278947361
O	0.18684209947368416	-2.3113518021052633	-3.3535934714210525	O	-2.4726940515789475	1.7039627473157894	-4.0921174572105263
H	-0.14953295952631585	-2.3253366021052631	-4.3004108714210529	H	-2.0332955515789473	1.4537935473157895	-4.9363215572105261
H	-0.15069984752631585	-1.4936025021052632	-3.0534122714210525	H	-2.0463654515789473	1.1565758073157895	-3.4173456572105265
O	0.74726707947368420	1.9146343978947367	-3.6735994714210527	O	1.0837454484210527	1.8037305473157894	-3.3510161572105264
H	0.68365953947368419	2.5855570978947369	-2.9682171714210526	H	0.83939040842105284	2.3065804473157896	-2.5331548572105262
H	0.23640601947368414	1.1537117878947369	-3.2867971714210524	H	0.39092785842105277	1.1469553173157894	-3.3771662572105265
O	-2.4478536505263158	1.6157603978947370	-4.2994807714210532	O	3.8365089421052739E-002	-2.5224838526842106	-3.4308988572105266
H	-1.8904356505263158	1.5671634978947369	-5.0838086714210533	H	-2.6002238578947262E-002	-2.9122160526842107	-4.4097647572105263
H	-2.0007671505263160	1.0056553978947369	-3.6543492714210526	H	-0.34888583157894726	-1.6267697526842104	-3.6120930572105263
O	-1.2179044505263159	-3.6517722021052634	-1.6075649714210525	O	1.0410337484210528	3.3945314473157895	-1.2083899572105263
H	-0.53804067052631588	-4.2862412021052636	-1.5376321714210526	H	1.7946479484210527	3.8988346473157893	-0.82336256721052636
H	-0.76901622052631580	-3.0377123021052634	-2.2435747714210525	H	1.2380165484210528	2.4580013473157893	-0.89796124721052639
O	2.9626878494736841	-1.7032841021052634	-3.3375302714210525	O	2.6431470484210524	-1.6254417526842104	-3.1374427572105263
H	1.9750098494736843	-1.7001795021052635	-3.2758330714210526	H	1.7714559484210528	-2.0177103526842104	-3.1268488572105264
H	3.0890067494736844	-2.4686209021052634	-4.0209307714210532	H	3.1299334484210526	-2.1093599526842106	-3.8580596572105263
O	3.2253753494736843	0.83946026789473682	-4.2370314714210533	O	-1.1014186515789472	-4.0198237526842107	-1.6263257572105263
H	2.8786022494736843	-1.7496582105263170E-002	-3.9175609714210524	H	-0.39037209157894726	-4.6760427526842108	-1.5860954572105261
H	2.3499990494736842	1.3000421378947369	-4.1901892714210529	H	-0.72350337157894717	-3.4579724526842104	-2.3275808572105263

C	-1.1875137252631578	0.19466777305263155	-1.1479834333684211	C	-1.3220649394736843	0.19787985810526310	-1.1786730381578947
C	-2.1787016252631579	0.82320345405263151	-0.38814934336842100	C	-2.3066508394736842	0.76084906510526307	-0.36565646815789477
C	-2.1004331252631578	0.47719075405263156	0.93295076663157905	C	-1.8949561394736842	0.65098921510526309	1.0490337618421053
O	-1.0147574752631576	-0.44284624594736843	1.1526872666315788	O	-0.89519869947368413	-0.31879800489473686	1.1076550618421053
C	-0.37988391526315790	-0.60260452594736846	-0.18169909336842102	C	-0.45020836947368414	-0.57680582489473686	-0.20067689815789475
O	-3.2523861252631576	1.6197669840526316	-0.78976824336842111	O	-3.3873079394736840	1.5696386451052633	-0.66948742815789475
O	-2.8226800252631579	1.0050753340526315	1.7856940666315790	O	-2.3159646394736844	1.2269708651052631	2.1080565618421057
C	1.0780291747368422	-0.21655617594736842	-0.26558418336842099	C	1.1041762605263159	-0.26670314489473690	-0.30804396815789475
O	1.4098098747368422	1.1073673740526315	0.26779225663157902	O	1.4304881605263158	0.99711203510526314	0.22428774184210526
O	-1.0216035252631579	0.20919532805263152	-2.3922280333684212	O	-1.1632265394736843	2.2385525105263093E-002	-2.3784096381578945
C	1.9625642747368421	-1.1804708159473685	0.49239578663157896	C	1.9841375605263161	-1.2778153548947371	0.37194366184210520
O	3.3896757747368422	-0.83377071594736851	0.36113225663157900	O	3.3742180605263159	-1.0115924548947368	0.12387954384210524
H	-2.8658448252631579	2.0959331840526314	-1.5502486333684211	H	-3.2081368394736844	2.4810832451052631	-0.89536578815789480
H	-0.44683707526315791	-1.6823232159473687	-0.38496769336842102	H	-0.60306519947368409	-1.6287512548947367	-0.43910411815789480
H	1.6761978747368422	-1.2338803159473684	1.5745529666315790	H	1.8807230605263157	-1.2688695548947369	1.5014485618421052
H	1.8845722747368421	-2.1656648159473684	1.0532410631578989E-002	H	1.8159786605263157	-2.3233351548947367	7.1524979842105235E-002
H	3.5297999747368420	3.5144314052631520E-002	0.69537354663157891	H	3.4044804605263157	-1.5580404894736888E-002	9.0221646842105235E-002
H	1.3730709747368421	-0.25956018594736852	-1.3211378333684212	H	1.3767391605263160	-0.25185360489473696	-1.4006248381578947

H	1.1758387605263159	1.0331963051052631	1.1879906618421052	H	1.6178520605263156	1.2496519103684212	0.89066100289473671
O	0.64765508052631582	1.6191217451052631	-3.4694748381578946	O	-2.0948235394736843	1.9789212103684211	-4.0109335971052635
H	0.75998837052631585	2.2113714451052631	-2.6854593381578944	H	-1.4519192394736844	2.0149906103684208	-4.7478424971052631
H	-0.10531066947368414	0.97120736510526307	-3.2117635381578946	H	-1.6550327394736843	1.3856974103684210	-3.3796300971052631
O	6.0410975263158612E-003	-2.2081489548947366	-3.3337303381578947	O	0.12635948052631571	-1.9703928896315790	-3.7438305971052630
H	1.0502437526315862E-002	-2.5836420548947370	-4.2407707381578952	H	-0.14104631947368429	-1.6327768896315791	-4.5785227971052631
H	-0.56539474947368418	-1.4110794548947370	-3.3964005381578946	H	-0.28698337947368430	-1.3540776896315789	-3.1253111971052632
O	-2.4731008394736844	2.0620695451052633	-3.8898712381578946	O	0.73869196052631569	2.2656875103684211	-3.9709243971052630
H	-1.8385497394736841	2.0505406451052632	-4.6237969381578949	H	0.95636846052631563	2.8613929103684210	-3.2332959971052633
H	-2.0149897394736844	1.5405893451052632	-3.2589985381578948	H	0.34944911152631575	1.5282598103684211	-3.4780715971052629
O	-1.1265717394736843	-3.8530641548947369	-1.5897272381578949	O	-3.2639885394736843	-0.23453685963157900	-6.1907405971052629
H	-0.43704945947368412	-4.4961492548947364	-1.6769751381578948	H	-3.1093566394736842	-1.1023627896315791	-6.5148304971052635
H	-0.83655134947368415	-3.0974509548947369	-2.1896896381578945	H	-2.3839524394736840	-0.12124700963157897	-5.7871896971052630
O	2.5688450605263156	-1.2768754548947370	-3.5465048381578947	O	-4.7992128394736842	1.8033870103684211	-4.6374297971052627
H	1.6098372605263158	-1.5018386548947369	-3.4568777381578943	H	-4.5843976394736838	1.4451536103684210	-5.5166680971052635
H	2.8908030605263160	-2.0721878548947368	-4.0472903381578949	H	-3.9296430394736843	1.8242207103684209	-4.2151305971052633
O	-3.4276796394736841	-0.15611601489473692	-6.2949595381578947	O	2.8132971605263157	-1.3238860896315789	-3.6670580971052629
H	-3.5494949394736843	-1.1005068548947370	-6.3368383381578948	H	1.8839614605263157	-1.7449849896315790	-3.6960009971052630
H	-2.4930132394736844	-0.16539160489473692	-5.9298446381578946	H	3.2704256605263158	-2.0979693896315790	-4.1492710971052631

C	-1.3535172247368423	0.28762266721052632	-1.0599824250789474	C	-1.1632865947368423	0.37045450905263161	-1.1238755684210526
C	-2.2044758247368419	1.0325452472105263	-0.26120374507894739	C	-2.1052577947368420	0.98247554005263171	-0.31734481842105267
C	-2.0390520247368427	0.72042126721052635	1.1265416749210526	C	-1.9419604947368421	0.63276275005263161	1.0162286315789473
O	-0.81935152473684236	-0.10087147278947364	1.2025389749210527	O	-0.86295909473684218	-0.31369163994736843	1.1527695315789475
C	-0.46441111473684227	-0.57123438278947358	-0.10754229507894736	C	-0.36190714473684227	-0.64922069994736842	-0.23368956842105265
O	-3.3439083247368426	1.6041775572105264	-0.85364973507894737	O	-3.0523684947368421	1.9508877100526316	-0.66751528842105268
O	-2.5867451247368427	1.1204240772105263	2.1691187749210523	O	-2.5905901947368424	1.0003311200526317	2.0454935315789475
C	1.0593727652631577	-0.46050684278947362	-0.31728999507894734	C	1.1085955852631577	-0.36192249994736841	-0.38251233842105264
O	1.5280426752631577	0.92324813721052634	-6.5346238578947349E-002	O	1.3375805052631577	1.0048007000526316	5.7405051578947347E-002
O	-1.1369529247368422	0.28229752021052634	-2.2728210250789473	O	-1.0899965947368422	0.42255741005263159	-2.3632056684210525
C	2.0039019752631577	-1.3040311427894737	0.55887020492105255	C	1.8441343052631578	-1.3316691899473683	0.53705877157894732
O	3.3677334752631580	-1.0906865427894739	0.12149536492105265	O	3.2556393052631578	-1.0285683899473683	0.42823073157894731
H	-2.9183944247368423	2.2946952572105261	-1.3839001250789473	H	-2.9786219947368422	2.1321530100526314	-1.6380391684210527
H	-0.76011745473684222	-1.6555041427894737	-0.11216153107894736	H	-0.56035192473684226	-1.7366986899473684	-0.31422679842105261
H	1.7341317752631578	-1.0034320427894738	1.5313047749210527	H	1.5544613052631577	-1.2449241899473684	1.6027822315789473
H	1.9299222752631577	-2.3777710427894738	0.41913963492105266	H	1.7066362052631578	-2.3785558899473687	0.25650186157894733
H	3.3467178752631579	-0.16124874278947368	-0.15096708907894735	H	3.4068017052631578	-8.2094499947368393E-002	0.45375741157894733
H	1.2241286752631577	-0.72330062278947360	-1.4025738250789472	H	1.5266807052631577	-0.45361327994736839	-1.4460012684210526
H	1.4329744752631577	1.1831552472105262	0.85842862492105265	H	0.96677070526315778	1.0845362200526316	0.93618273157894727
O	-0.15604711473684230	-2.0517552427894739	-3.1676462250789474	O	-2.4332643947368422	1.9907178100526317	-4.0294360684210524
H	3.4706835263157737E-002	-1.8780152427894738	-4.1528597250789474	H	-1.6579774947368422	2.3646775100526316	-4.3506903684210529
H	-0.39182598473684227	-1.1501774427894738	-2.9632975250789477	H	-2.0363380947368421	1.2996971700526316	-3.4887679684210529
O	-2.3762351247368425	2.0232165572105263	-4.1715889250789475	O	-0.72369926473684221	-2.1047496899473686	-3.5353520684210524
H	-1.7380833247368423	1.9111270572105263	-4.9408761250789475	H	-0.57444755473684228	-1.8085105899473681	-4.4582749684210530
H	-2.0539138247368420	1.3193180572105263	-3.5089517250789477	H	-1.0696509947368422	-1.3159632899473683	-3.1326468684210527
O	0.87755994526315773	1.6251692572105263	-3.7149731250789477	O	0.87555610526315775	2.2436805100526316	-4.0259245684210523
H	0.98672322526315770	2.2449460572105262	-2.9635360250789473	H	0.69870828526315776	2.8453620100526313	-3.2847538684210527
H	0.29001192526315772	0.92299747721052638	-3.3564875250789474	H	0.69514399526315773	1.4121619100526317	-3.5685154684210527
O	1.4449120752631577	3.2298646572105261	-1.5517049250789472	O	1.1006866652631577	3.2998322100526316	-1.2399178684210526
H	2.3365032752631576	3.5530886572105262	-1.8027545250789474	H	1.7737796052631578	3.9530754100526315	-0.88182525842105264
H	1.5615998752631577	2.2862775572105263	-1.2921872250789472	H	1.3310055052631578	2.4240850100526314	-0.85439244842105266
O	2.7088211752631581	-1.3729959427894738	-3.4321017250789474	O	2.7308268052631579	-1.2451321899473684	-3.1843925684210528
H	1.7673729752631577	-1.5290333427894738	-3.1757409250789475	H	1.9002127052631577	-1.7651662899473683	-3.3540861684210528
H	2.8612331752631581	-2.1654593427894739	-3.9891937250789473	H	3.3557856052631578	-1.8603536899473683	-3.5714909684210525
O	-3.5010369247368427	-0.34065009278947367	-5.9748427250789478	O	-0.78172592473684221	2.2667107100526316	-6.3802719684210523
H	-3.3868617247368427	-1.0883512427894737	-6.5784025250789480	H	-1.0168063947368422	1.7504902100526316	-7.1455711684210526
H	-2.5630888247368420	-0.29203990278947367	-5.5724102250789471	H	-0.12576379473684224	1.7997957100526316	-5.8406736684210525

C	-1.3601556394736842	0.24289880336842101	-1.1220065771052632	C	-1.3044379410526314	0.26542231260526317	-1.0971034385105263
C	-2.2625419394736843	0.93535611036842103	-0.38758848710526317	C	-2.0338806410526313	1.0171188066052632	-0.21010309551052633
C	-1.9662706394736842	0.85780334036842099	1.0027067028947370	C	-1.8713100410526313	0.59358685660526311	1.1347698114894738
O	-0.88418993947368429	-0.16795593963157895	1.1923378028947369	O	-0.82278484105263150	-0.37397655339473684	1.2243419114894738
C	-0.45268610947368432	-0.46139163963157892	-0.14822419910526316	C	-0.43058211105263144	-0.59542240339473684	-0.14746152851052632
O	-3.1992086394736843	1.7923323103684210	-0.93473901710526319	O	-2.9760962410526313	2.0723214066052633	-0.39137970851052634
O	-2.4993764394736839	1.3631596103684209	2.0310376028947368	O	-2.4231199410526316	0.95498143660526313	2.1872437114894736
C	1.0525760105263158	-0.30158322963157902	-0.29254080710526315	C	1.0446590389473687	-0.27358516339473682	-0.28398143851052632
O	1.5269327605263157	1.0055442003684210	-2.4303597105263169E-002	O	1.4328047589473685	1.0055745566052632	0.17319677148947366
O	-1.2252646394736844	6.9931530368421035E-002	-2.3762419971052631	O	-1.2407917410526315	0.18413498510526319	-2.3445176885105266
C	1.8064955605263158	-1.3359095896315789	0.58438904289473681	C	1.8875458589473686	-1.3767943933947369	0.37449672148947366
O	3.2659955605263158	-1.4621840896315790	0.34510940289473679	O	3.2372119589473685	-1.1619904933947369	3.4993321489473678E-002
H	-2.9695380394736843	2.1390942103684210	-1.7554346971052630	H	-3.0168711410526314	2.3272853066052632	-1.3438686885105262
H	-0.61516976947368418	-1.5415148896315789	-0.25966959010526314	H	-0.63758897105263146	-1.6764943933947369	-0.44447000851052632
H	1.6448189605263157	-1.0515171896315789	1.6939584028947370	H	1.8047908589473685	-1.3452818933947368	1.4616267114894737
H	1.4352478605263157	-2.3334665896315792	0.51132176289473685	H	1.6588654589473686	-2.4448777933947370	4.7419241489473657E-002
H	3.7097432605263161	-0.55355493963157898	0.35209364289473677	H	3.2161461589473683	-0.18910723339473678	-0.12480482981052633
H	1.3747397605263156	-0.44669392963157895	-1.3028663971052630	H	1.3082171589473686	-0.16882665339473679	-1.3361089885105262

H	1.1672223589473685	1.1859313066052632	1.0857112114894738
O	-2.2973232410526316	1.9029933066052631	-4.1351553885105261
H	-1.5574696410526314	2.0736204066052633	-4.7531716885105260
H	-1.9553140410526315	1.1595523066052631	-3.5510384885105264
O	-0.23447375105263146	-2.2062939933947368	-3.4228130885105266
H	-0.71838510105263143	-1.9931655933947368	-4.2091905885105261
H	-0.37248629105263142	-1.3196940933947368	-2.9203176885105266
O	0.68592796894736852	2.2182104066052633	-4.0563109885105266
H	0.63880919894736854	3.0068320066052632	-3.5300590885105265
H	0.24757461894736851	1.5778808066052632	-3.4985733885105263
O	-5.0129488410526317	1.4895030066052632	-4.7561887885105261
H	-4.1630613410526314	1.5396781066052632	-4.3582042885105263
H	-4.5649727410526317	1.0878761266052632	-5.5696444885105256
O	-0.98118054105263153	-4.3952963933947364	-1.8797950885105263
H	-0.13088656105263147	-4.9041304933947369	-1.6746443885105262
H	-0.70524263105263152	-3.6254759933947369	-2.3158510885105263
O	2.5333312589473684	-1.5184849933947369	-3.5653048885105263
H	1.7365714589473684	-2.0408491933947368	-3.5586762885105263
H	3.1768338589473686	-2.1883404933947368	-3.7257907885105266

C	-1.3012918094736841	0.24849412747368427	-1.1181535658421053
C	-2.0860692094736839	1.0458283744736843	-0.29049813584210527
C	-1.9046371094736843	0.69647081447368420	1.1052062341578948
O	-0.87651240947368403	-0.30372938552631573	1.1868901341578946
C	-0.48019050947368402	-0.64536069552631581	-0.20024627284210525
O	-2.8415416094736843	2.1673747444736842	-0.61613583584210518
O	-2.5240524094736845	1.1135764344736843	2.1131456341578949
C	1.0149093105263161	-0.43932190552631573	-0.32746290584210525
O	1.2720229405263159	0.92021970447368417	0.10021659415789475
O	-1.1619897094736840	0.30224937647368427	-2.3546547658421053
C	1.8606211905263159	-1.3117586555263159	0.52625785415789472
O	3.2426693905263155	-1.2523925555263158	0.15447071415789473
H	-2.6082080094736844	2.5166300444736840	-1.4964539658421054
H	-0.77903410947368412	-1.7018060555263157	-0.36968989584210521
H	1.7352441905263158	-1.0415768555263158	1.6025510341578946
H	1.7156897905263160	-2.3794725555263159	0.40263454415789479
H	3.5920327905263161	-0.32336229552631574	-2.4674167842105257E-002
H	1.1546497905263160	-0.50549997552631565	-1.3814253658421054
H	0.97568750052631592	0.89343731447368424	0.98802213415789486
O	0.22770809552631593	-1.7764543555263157	-3.3780226658421051
H	0.11759129052631592	-1.4461594555263158	-4.2966647658421060
H	-0.37419588947368410	-1.1278467555263159	-2.9764503658421053
O	-2.3847251094736839	2.2401615444736840	-3.9966430658421053
H	-1.9925663094736838	2.4194740444736844	-4.8693287658421056
H	-1.7519229094736841	1.5552840444736842	-3.7499053658421051
O	0.95880013052631596	1.8520783444736841	-3.8238503658421052
H	0.83698115052631594	2.5298201444736841	-3.1295600658421052
H	0.35744086552631593	1.1333453144736843	-3.4795852658421049
O	-3.1163646094736839	2.3015914473684257E-002	-5.8080748658421060
H	-3.0640798094736841	-0.80580705552631571	-6.2831178658421054
H	-2.2254751094736838	4.0223114473684263E-002	-5.4761728658421056
O	1.2517578105263159	3.3165217444736843	-1.2875446658421053
H	2.2308127905263158	3.4484263444736842	-1.1626706658421053
H	1.0605373405263159	2.5387713444736844	-0.71299024584210524
O	-1.0873276094736841	-3.7822262555263162	-2.1474628658421051
H	-0.25077545947368407	-4.1885559555263159	-1.7652341658421054
H	-0.76301740947368413	-3.2061585555263159	-2.8572557658421052

C	-1.3306337063157894	0.32441101894736835	-1.0295928237368421
C	-2.0658277063157895	1.1859536189473683	-0.24649096373684209
C	-2.0007274063157894	0.75764307894736838	1.1256997662631578
O	-1.0392291063157895	-0.24739247105263160	1.2643482662631578
C	-0.52218628631578945	-0.58795422105263162	-0.12577156773684209
O	-2.7741167063157892	2.3639018189473684	-0.53015232373684207
O	-2.6505127063157894	1.1581070189473683	2.0928452662631578
C	1.0324018036842104	-0.48338729105263156	-0.32042705373684205
O	1.4215737936842106	0.87466264894736845	7.9715376263157903E-002
O	-1.1001699063157895	0.28724143894736842	-2.2652961337368422
C	1.9055794936842105	-1.5003091810526317	0.42701336626315795
O	3.2452260936842108	-1.3271231810526316	-4.3729971736842087E-002
H	-2.6488672063157894	2.6171496189473684	-1.4400583337368422
H	-0.93937700631578935	-1.6279962810526316	-0.29248088373684211
H	1.8932998936842105	-1.3819143810526318	1.5266104662631579
H	1.6617555936842106	-2.5669805810526314	0.21684693626315793
H	3.4594793936842105	-0.33702811105263164	-0.10712672073684210
H	1.2719426436842105	-0.65437098105263158	-1.3428167337368422

H	1.1803890336842104	1.1453864189473684	1.0108640662631578
O	-2.1613560063157893	2.0209142189473686	-3.8751698337368423
H	-1.4601510063157894	1.9668230189473683	-4.5884412337368419
H	-1.9208652063157894	1.2396749189473684	-3.3099185337368420
O	0.85805426368421056	1.5445647189473684	-3.7360050337368422
H	0.83378254368421056	2.0887998189473684	-2.9326964337368420
H	0.30030646068421057	0.76802128894736843	-3.6215941337368420
O	0.69873192368421055	-2.3046700810526315	-3.4586904337368423
H	0.52690718368421052	-2.6792748810526317	-4.3760796337368424
H	-7.2533846315789408E-002	-1.7790133810526316	-3.3034906337368422
O	1.3990545936842105	3.0795986189473683	-1.5241561337368421
H	2.3700447936842104	3.0014160189473684	-1.8152147337368421
H	1.1978015236842106	2.3080621189473685	-0.96648965373684215
O	-4.8066364063157891	1.6365124189473683	-4.3159415337368419
H	-4.0324353063157892	1.7103494189473682	-3.7160707337368422
H	-4.4538642063157896	1.1424614189473683	-5.0985459337368422
O	-0.28029082631578939	-0.92913948105263156	-5.6890296337368422
H	0.61485201368421061	-1.1867211810526317	-5.8701091337368423
H	-0.75913890631578940	-1.6755388810526317	-6.0582502337368425

C	-1.3392617010526315	0.36980726315789475	-1.1082298573684211
C	-2.0347867010526315	1.1693009131578949	-0.25692694736842103
C	-1.8828032010526314	0.81229931315789472	1.1166003426315789
O	-0.95495000105263173	-0.28919644684210521	1.1599861426315790
C	-0.52024493105263159	-0.62844682684210529	-0.12743979536842104
O	-2.8422805010526315	2.2271129131578946	-0.59229009736842109
O	-2.3804966010526316	1.1424761631578948	2.1971574426315787
C	0.97596854894736840	-0.50786126684210531	-0.31106496736842104
O	1.4418679989473684	0.82369998315789472	-4.6497091368421034E-002
O	-1.1600899010526315	0.36708151315789472	-2.3647521573684211
C	1.8987208989473683	-1.4313162868421052	0.53230664263157901
O	3.2094846989473682	-1.1368171868421051	0.11516316263157897
H	-2.5783514010526316	2.4205710131578946	-1.5171265573684212
H	-0.76601570105263161	-1.7037172868421051	-0.16612194436842104
H	1.6956314989473684	-1.3285621868421051	1.6277058426315789
H	1.6775448989473685	-2.4729386868421055	0.28864796263157894
H	3.1453545989473684	-0.14348050684210525	-5.7190008368421037E-002
H	1.0631459989473684	-0.69072299684210525	-1.3913182573684211
H	1.3515614989473683	1.0007106031578947	0.90139014263157891
O	-2.3471685010526318	2.2955532131578944	-3.9867842573684209
H	-1.7740986010526316	2.1384749131578946	-4.7803763573684215
H	-1.9488888010526315	1.5422799131578948	-3.4102926573684211
O	0.92198282894736838	1.7570206131578949	-3.8128915573684212
H	0.85148905894736848	2.3686966131578946	-3.0745823573684210
H	0.30575367294736844	1.0596953131578948	-3.4601296573684213
O	0.30860434194736841	-2.0865876868421056	-3.0321928573684209
H	0.35218833594736842	-2.3636620868421052	-3.9630684573684212
H	-0.14562403105263155	-1.2049624868421052	-3.1370776573684211
O	1.3720839989473683	3.1356691131578946	-1.3393465573684211
H	2.3266749989473685	3.3422166131578948	-1.2653709573684211
H	1.3941916989473684	2.1973319131578948	-1.0262722773684210
O	-1.1505447010526317	-3.8347468868421055	-1.5553070573684211
H	-0.45401654105263162	-4.5172093868421053	-1.5496850573684211
H	-0.62030724105263157	-3.1010964868421054	-1.9953154573684211
O	-5.0635005010526317	1.5102089131578948	-4.1490207573684215
H	-4.1736767010526314	1.7700910131578949	-3.9118492573684209
H	-4.9469801010526320	1.4862285131578947	-5.0764545573684208

5.3 Doubly deprotonated molecule (at C2–O^- and C3–O^-) of vitamin C with 12 explicit water molecules

C	0.16276686E+01	-0.27426912E+01	0.22154128E+01
C	0.16597455E+01	-0.13846211E+01	0.27627721E+01
O	0.14797277E+01	-0.47190040E+00	0.17427947E+01
C	0.11465740E+01	-0.11998840E+01	0.52063880E+00
C	0.12301422E+01	-0.26319647E+01	0.89462792E+00
O	0.19336789E+01	-0.97235693E+00	0.39169536E+01
C	-0.25338970E+00	-0.65263002E+00	-0.13288306E+00
O	-0.13318476E+01	-0.78864023E+00	0.76298752E+00
O	0.95727779999999996	-3.5862769000000001	4.4487976999999998E-002
O	1.8820910000000000	-3.7502426999999998	2.9897098999999998
C	-0.90648718E-01	0.81909966E+00	-0.59176548E+00
O	-0.14217320E+01	0.12399407E+01	-0.97825952E+00
H	0.18849831E+01	-0.98133145E+00	-0.24962611E+00
H	0.26708035E+00	0.14558277E+01	0.16805353E+00
H	0.61894656E+00	0.89464299E+00	-0.14758717E+01
H	-0.15610569E+01	0.22209768E+01	-0.10466074E+01
H	-0.40614156E+00	-0.12936317E+01	-0.10033456E+01
H	-0.11138054E+01	-0.25409428E+00	0.15649613E+01
O	1.5626241999999999	-5.9636361999999998	1.3999174000000001
H	1.6296828000000001	-5.1899015000000004	2.0491117000000001
H	1.3505148000000000	-5.4680567000000000	0.61032598999999998
O	-0.79557593000000004	-3.8294424999999999	3.6793608999999998
H	0.19702864000000000	-3.9769169000000000	3.4658365999999998
H	-1.2179371999999999	-4.5031613000000004	3.0514964000000000
O	3.3387194999999998	-3.7571683000000000	-1.3365395000000000
H	2.7626265000000001	-3.9817434999999999	-0.58332527000000001

H	4.0124276999999999	-3.2305660999999999	-0.87534763999999998
O	4.6524824999999996	-3.7129458999999998	2.7123974000000000
H	4.8573091000000002	-4.6155201999999997	2.7570918000000000
H	3.6635468000000002	-3.7499994000000001	2.6817665000000002
O	-1.4402641000000000	-5.0627081000000000	2.7807315999999999E-002
H	-0.65658612000000005	-4.5168885999999997	0.14684738000000000
H	-1.3177926000000000	-5.7311481000000004	0.72696983000000004
O	3.9501331999999998	-6.8554487999999996	1.0599052000000000
H	3.6166369999999999	-7.5471326000000003	1.6325466000000000
H	3.2396501000000000	-6.2910010999999999	1.2496711000000000
O	2.9030382000000001	-5.8210338000000004	4.8903717999999996
H	2.6506406999999998	-5.1493400999999999	4.2702736999999997
H	2.2184889000000001	-6.4467615000000000	4.7054112000000003
O	1.6044168000000001	-3.5147906999999998	5.7986110000000002
H	2.2156056999999998	-4.3490099999999998	5.7800152999999996
H	1.5451611000000001	-3.3248527000000001	4.8594705999999999
O	0.82864923999999995	-7.3166490000000000	3.7177527000000001
H	0.61347399000000002	-6.6869762000000001	2.9996103999999999
H	1.5888534000000001	-7.7488856000000004	3.3058231999999999
O	-0.21437259000000000	-3.4704366000000002	-2.6724122000000001
H	3.5955790000000001E-002	-4.3586841999999999	-2.9260681000000002
H	3.7909361000000003E-002	-3.5891673000000002	-1.7433558000000000
O	-2.8227975000000001	-3.1078768999999999	1.0216556999999999
H	-2.3392127000000000	-3.8805603000000000	0.62313848000000005
H	-2.3026875000000002	-2.3552423999999998	0.74025090000000004
O	1.8514603999999999	-1.8309386999999999	-2.6734708999999999
H	2.3761727000000001	-2.2376046999999999	-2.0213399000000001
H	0.97784157000000005	-2.2975848000000001	-2.4921104000000001

C	0.15122821E+01	-0.28871651E+01	0.22007604E+01
C	0.14858721E+01	-0.15901309E+01	0.28065364E+01
O	0.10694795E+01	-0.59081960E+00	0.18820499E+01
C	0.88337540E+00	-0.13034403E+01	0.61075350E+00
C	0.97296904E+00	-0.27891703E+01	0.94192287E+00
O	0.18069858E+01	-0.11977420E+01	0.39639582E+01
C	-0.30006780E+00	-0.71421104E+00	-0.84760642E-01
O	-0.14446668E+01	-0.78522269E+00	0.81349452E+00
O	0.78235650000000001	-3.6946493999999999	3.2855254000000000E-002
O	1.9256932000000000	-3.9187463000000000	2.8786545000000001
C	-0.95591912E-01	0.73752013E+00	-0.49092499E+00
O	-0.13712562E+01	0.13249834E+01	-0.98466697E+00
H	0.17676420E+01	-0.98947378E+00	-0.10036895E-01
H	0.22386065E+00	0.13518824E+01	0.34931102E+00
H	0.66684478E+00	0.77179912E+00	-0.13112112E+01
H	-0.14482791E+01	0.21363766E+01	-0.45632927E+00
H	-0.57648200E+00	-0.12456385E+01	-0.10604625E+01
H	-0.11611478E+01	-0.55674763E+00	0.17313358E+01
O	1.2924069000000000	-6.0887735000000003	1.4939106000000000
H	1.5068912000000001	-5.2948494000000004	1.9966934999999999
H	1.0874708000000000	-5.5207696999999998	0.74845923000000003
O	3.2787356999999999	-3.5464810999999998	-1.2634500000000000
H	2.4834100000000001	-3.8126924999999998	-0.77252178000000005
H	3.8793780999999998	-2.9754339999999999	-0.67230816999999998
O	-0.86885732000000004	-4.0412077000000002	3.7791367000000000
H	9.5269800000000002E-002	-4.1585479999999997	3.7601285000000000
H	-1.1300140000000001	-4.7520126999999999	3.1769769999999999
O	-1.5251307000000001	-5.2705194000000004	7.1017888000000001E-002
H	-0.71019549000000004	-4.6907107000000003	2.7438665000000001E-002
H	-1.5140207999999999	-5.7008061000000003	0.96930963999999997
O	4.6451656999999997	-3.8329371999999999	2.9686275000000002
H	4.7949755999999999	-4.7767238000000001	3.0264850000000001
H	3.6119713999999999	-3.8683646999999999	2.8946288000000000
O	3.0993993999999998	-5.8827007000000000	4.4432675000000001
H	2.7544648000000000	-5.2203017999999997	3.8222255999999999
H	2.3217298000000000	-6.5222201000000002	4.3601884999999996
O	1.5174683000000000	-3.8571371000000001	5.7861364999999996
H	2.1142506000000001	-4.5682280000000004	5.9406203000000000
H	1.6904045999999999	-3.7087501000000000	4.8028963999999998
O	-3.1674911000000000E-002	-3.5318822999999999	-2.6953914000000001
H	0.10737134000000000	-4.3533075999999999	-3.2570627000000001
H	-3.4766851000000001E-002	-3.8207974999999998	-1.7182306000000001
O	1.0232007999999999	-7.5021101999999997	3.5919821999999999
H	0.82949346000000002	-7.0025444999999999	2.7929865999999999
H	1.5800547000000000	-8.2356055000000001	3.3187399000000002
O	-2.8098119000000001	-3.0801772000000001	1.3446636000000001
H	-2.6378457000000002	-3.9358510999999998	0.88138545000000001

H	-2.0229121000000001	-2.6045080999999999	1.2851475000000001
O	4.0134793999999996	-7.1225464000000001	1.2749097000000000
H	3.7142176999999998	-7.7603019000000000	0.57305181999999999
H	3.1389296999999998	-6.9332865000000004	1.6598280000000001
O	5.1194265000000003	-2.2073151000000002	0.60959173000000000
H	4.9589235000000000	-2.8359939999999999	1.2944019000000000
H	5.4833537000000003	-1.4301119000000000	1.0696840999999999

C	0.16882591E+01	-0.26991295E+01	0.22277372E+01
C	0.17701170E+01	-0.13913474E+01	0.27153125E+01
O	0.14083354E+01	-0.43179217E+00	0.17094152E+01
C	0.11074578E+01	-0.11623065E+01	0.56177524E+00
C	0.11001470E+01	-0.26412412E+01	0.97196453E+00
O	0.19406862E+01	-0.10103694E+01	0.38898224E+01
C	-0.18405623E+00	-0.66314047E+00	-0.41269318E-01
O	-0.12331474E+01	-0.79080591E+00	0.89798956E+00
O	0.85318287999999998	-3.5227976000000001	7.7953898999999993E-002
O	1.8828440000000000	-3.8256039999999998	2.9351696000000000
C	-0.10611843E+00	0.77580357E+00	-0.55961400E+00
O	-0.14108678E+01	0.11479511E+01	-0.11091600E+01
H	0.20123035E+01	-0.91491920E+00	-0.44517324E-01
H	0.32987967E+00	0.14231814E+01	0.22643891E+00
H	0.64603599E+00	0.78296868E+00	-0.13656862E+01
H	-0.15308759E+01	0.21020375E+01	-0.10295375E+01
H	-0.44802145E+00	-0.13964769E+01	-0.86897069E+00
H	-0.98895706E+00	-0.37588637E+00	0.17195824E+01
O	1.5177377000000001	-6.1755871000000004	1.3341236999999999
H	1.7176870000000000	-5.3579981999999999	1.8706186000000000
H	1.2293626000000000	-5.7277920000000000	0.53209574000000004
O	-0.78661544000000005	-4.1261178000000003	3.8860326999999999
H	1.4383119999999999E-002	-4.0299914000000001	3.4688789000000000
H	-1.0960550000000000	-4.8321528999999996	3.2826293999999998
O	3.2107228999999999	-3.2748187999999998	-1.3187913000000000
H	2.5141536000000002	-3.6961626000000001	-0.76392521000000002
H	3.9319445000000002	-3.0496213000000001	-0.63729197999999998
O	-1.5136769999999999	-5.1664801999999996	0.21126909999999999
H	-0.63172233000000000	-4.8171419999999996	0.36240791000000000
H	-1.7177819999999999	-5.5405540999999996	1.0716239000000001
O	4.5902069000000001	-3.9508000000000001	2.9126827000000000
H	4.9097682000000002	-4.8463503000000001	2.8752444000000001
H	3.5580362999999999	-3.9698381999999999	2.8340108000000002
O	3.0230260000000002	-5.9694023999999999	4.3988828000000000
H	2.8912287000000001	-5.2917800000000002	3.6704055000000002
H	2.2376654000000000	-6.5254402999999996	4.3442844000000003
O	1.7182282000000000	-3.7701093000000001	5.7106291999999996
H	2.1826235999999999	-4.6110075999999998	5.4986431000000001
H	1.6790487000000001	-3.3772812000000001	4.8108985000000004
O	4.7510006000000002	-2.1779750000000000	0.65461926000000004
H	4.6383049999999999	-2.8076555000000001	1.3796769000000000
H	5.0102425999999998	-1.3538912999999999	1.1952305000000001
O	0.20472219999999999	-3.4359882000000002	-2.7473972000000000
H	0.43263473000000002	-4.2292056999999996	-3.3459498999999999
H	5.1527554000000003E-002	-3.7067239000000001	-1.8463099999999999
O	-2.8057808000000000	-3.0485932999999998	1.4214648999999999
H	-2.2258589999999998	-3.6287973999999998	1.0043662000000000
H	-2.3246521000000002	-2.1904210000000002	1.4203395000000001
O	4.1108093999999999	-7.1931089000000004	1.0176955999999999
H	3.9090126999999999	-8.0416427000000006	1.5142933999999999
H	3.2546069000000002	-6.8175692999999997	0.83527655000000001
O	-1.1444728000000000	-1.3496338000000001	4.3413792000000004
H	-1.1586669000000001	-2.2969081999999998	4.1621430000000004
H	-0.22485540000000001	-1.1980510000000000	4.5920902000000003

C	0.14317077E+01	-0.29184453E+01	0.22998025E+01
C	0.15998344E+01	-0.16250058E+01	0.28623697E+01
O	0.14019559E+01	-0.61137177E+00	0.19207035E+01
C	0.10096264E+01	-0.12936437E+01	0.65063658E+00
C	0.10353921E+01	-0.28013863E+01	0.96598155E+00
O	0.18386699E+01	-0.13565399E+01	0.40586199E+01
C	-0.27334441E+00	-0.57009758E+00	0.66715958E-01
O	-0.12180299E+01	-0.37481197E+00	0.11272767E+01
O	0.76617864000000002	-3.7011414000000000	8.1937562000000005E-002
O	1.6376728000000000	-3.9727152000000001	3.0771929000000000
C	0.11433801E+00	0.80776745E+00	-0.52319309E+00
O	-0.11860391E+01	0.13521054E+01	-0.94281975E+00
H	0.17737695E+01	-0.10972253E+01	-0.15379971E+00

H	0.54892221E+00	0.15229968E+01	0.21060995E+00
H	0.79791190E+00	0.84325366E+00	-0.13958521E+01
H	-0.12607598E+01	0.23081684E+01	-0.62252210E+00
H	-0.72102917E+00	-0.11669920E+01	-0.71839136E+00
H	-0.88615373E+00	0.16864600E+00	0.18521782E+01
O	1.3839532999999999	-6.2765439000000001	1.5701848000000000
H	1.2580222999999999	-5.4311211999999998	2.0109667000000000
H	1.0509523999999999	-6.0738232999999999	0.68544441000000000
O	-1.6445436000000000	-5.0778353999999997	0.48354870999999999
H	-0.74909912999999995	-4.6911320999999999	0.44432161999999997
H	-1.6510857000000001	-5.7364050999999998	1.2010874000000000
O	-0.99244124000000000	-3.9459379000000001	4.0842220999999999
H	-5.3045223000000002E-002	-4.1803971999999998	3.7935286000000001
H	-1.4263317000000000	-4.5732789000000000	3.4510177999999998
O	4.6799413000000003	-4.1547196000000000	2.6827443000000000
H	4.8915649999999999	-5.0850340000000003	2.5490230999999999
H	3.6784948000000002	-4.0536231999999996	2.6412924000000002
O	3.1241663000000002	-3.4437142999999999	-1.4550304000000001
H	2.4529466000000002	-3.8140285000000000	-0.89417380999999996
H	3.8234094000000001	-3.1871299999999998	-0.84630640999999995
O	3.1865613000000002	-5.8572135999999997	4.5056786999999998
H	2.7667331000000002	-5.0855060999999999	4.0051188000000000
H	2.5550503999999998	-6.6289774000000001	4.4248120999999996
O	4.1627485999999996	-6.7399088999999999	1.3772922000000001
H	3.9814905999999999	-7.4987120000000003	0.83931246000000004
H	3.2496041999999998	-6.3220898999999999	1.3836575000000000
O	-9.3492591000000000E-002	-3.4220271000000002	-2.5197858000000002
H	0.43792269000000000	-4.1172253000000003	-2.8884211999999998
H	0.15208267000000000	-3.4720008999999998	-1.5645309999999999
O	1.6646247999999999	-3.8170169000000000	5.9335646999999998
H	1.9312540000000000	-4.7092179999999999	5.7484652000000001
H	1.4824790000000001	-3.5105365000000002	5.0156751000000002
O	-2.7409151000000000	-2.6074893000000001	1.5396056000000000
H	-2.2716205999999999	-3.3631338999999998	1.1677848000000000
H	-2.0298216999999998	-1.9239268000000000	1.5106702000000001
O	4.6815169000000001	-2.3929231999999998	0.51507486000000002
H	4.7140760000000004	-3.1287408000000001	1.1212806000000000
H	5.1978033999999997	-1.6661351000000000	0.98862766000000002
O	1.2558271000000001	-7.9284435999999996	3.8510038999999998
H	1.0825480000000001	-7.3987669000000000	3.0465713999999999
H	1.9896571999999999	-8.5839368000000000	3.5587715000000002

C	0.16885062E+01	-0.28885581E+01	0.24046318E+01
C	0.19585698E+01	-0.15592714E+01	0.28893593E+01
O	0.16377522E+01	-0.53169847E+00	0.19186227E+01
C	0.12204372E+01	-0.12173119E+01	0.69344733E+00
C	0.11747421E+01	-0.27053657E+01	0.11085448E+01
O	0.23242773E+01	-0.11673562E+01	0.40015140E+01
C	-0.15640267E+00	-0.63213244E+00	0.11128775E+00
O	-0.12021475E+01	-0.46822004E+00	0.11578417E+01
O	0.71254795999999998	-3.6037606000000002	0.24291335999999999
O	1.7553243000000001	-4.0095780000000003	3.1037599999999999
C	0.10881777E+00	0.75790567E+00	-0.48306258E+00
O	-0.10394989E+01	0.14054206E+01	-0.10449081E+01
H	0.19669586E+01	-0.10275696E+01	-0.32206843E-01
H	0.61626990E+00	0.13816563E+01	0.32664705E+00
H	0.83122731E+00	0.68841199E+00	-0.13483653E+01
H	-0.13053746E+01	0.22111094E+01	-0.57931161E+00
H	-0.57476472E+00	-0.12357650E+01	-0.63696582E+00
H	-0.67685824E+00	-0.24058622E+00	0.19494109E+01
O	1.4416446000000001	-6.0945568000000003	1.5918520000000000
H	1.5937387999999999	-5.2068871000000003	2.1123725000000002
H	0.87519217999999999	-5.6355800000000000	0.96929522999999995
O	-1.7401911999999999	-4.8221125000000002	0.33891253999999998
H	-0.83081258999999996	-4.5092600000000003	0.55638122999999996
H	-1.8877400000000000	-5.3393690999999999	1.1884775999999999
O	-0.92895145000000001	-3.7646746000000002	4.1727914000000004
H	-6.2751599000000005E-002	-4.1367101000000002	3.9796925000000001
H	-1.4145652000000000	-4.2388808999999998	3.4789439000000000
O	3.1925218000000002	-3.4128207000000002	-1.3787639000000000
H	3.0214036000000002	-4.0572891000000002	-0.68355171000000003
H	3.9059276000000001	-2.8654842000000000	-0.90697338999999999
O	4.9916533999999997	-4.3278663000000002	2.3153223999999999
H	4.7051432000000002	-5.1675452000000002	1.8259950000000000
H	4.0981828000000000	-3.9806691000000001	2.5867580000000001
O	3.1942409999999999	-5.7773984000000000	4.6356415000000002

H	2.9723339000000002	-5.1727376999999999	3.9127022000000000
H	2.4749061999999999	-6.4075847000000001	4.5473352000000000
O	4.0472362000000004	-6.7416799999999997	0.79728977999999995
H	4.0804893000000000	-7.5474012999999998	0.28309067999999998
H	3.0996956999999998	-6.6246254999999996	1.0978730000000001
O	-0.15828558000000001	-3.2707948000000000	-2.5089038000000001
H	-0.60163745999999996	-4.0826279000000003	-2.7733607999999998
H	0.20379175999999999	-3.3803185999999998	-1.5787351000000001
O	1.7828170999999999	-3.9518767000000001	6.0743274999999999
H	2.2095349000000000	-4.7782410000000004	6.4423352999999999
H	2.0665496000000001	-4.1535207999999999	5.1609921999999999
O	-2.8462920000000000	-2.5798809999999999	1.5587530000000001
H	-2.3495021000000000	-3.3191367000000001	1.1159916999999999
H	-2.1445758000000001	-1.9131688000000000	1.4134503000000000
O	1.5797995000000000	-7.9760964000000003	3.5640985999999999
H	1.3792412999999999	-7.2992910000000002	2.8582437999999999
H	2.2910135999999999	-8.5046335000000006	3.2161141000000000
O	-0.92753795999999999	-1.0671839000000001	4.1856236999999998
H	-0.99989315000000001	-2.0437395000000000	4.2599251000000002
H	-5.1473802999999999E-002	-0.92140217999999996	4.4377256999999997

C	0.14610441E+01	-0.29089852E+01	0.24692164E+01
C	0.18245000E+01	-0.15621116E+01	0.28949443E+01
O	0.16846679E+01	-0.69724226E+00	0.17914133E+01
C	0.12334282E+01	-0.14137964E+01	0.62495238E+00
C	0.11439735E+01	-0.28645140E+01	0.11082466E+01
O	0.21742097E+01	-0.10106698E+01	0.39671633E+01
C	-0.84699926E-01	-0.77273834E+00	0.88820566E-01
O	-0.10968870E+01	-0.59582691E+00	0.10826778E+01
O	0.82696855999999996	-3.7862545000000001	0.25205404999999997
O	1.3427998000000001	-3.9579197000000002	3.2880571999999999
C	0.19793356E+00	0.64552791E+00	-0.49124170E+00
O	-0.10042176E+01	0.11082730E+01	-0.10735364E+01
H	0.20127217E+01	-0.13008736E+01	-0.99099361E-01
H	0.56317579E+00	0.12705724E+01	0.32216166E+00
H	0.91506138E+00	0.62305157E+00	-0.13090517E+01
H	-0.11512773E+01	0.20798846E+01	-0.94107853E+00
H	-0.38963965E+00	-0.14846258E+01	-0.76047897E+00
H	-0.70259042E+00	-0.74807667E+00	0.19547292E+01
O	1.6757152000000000	-6.0911033000000003	1.4047115999999999
H	1.4243631999999999	-5.4698212000000002	2.1372089999999999
H	1.3395226000000000	-5.6069825000000000	0.63913067999999995
O	-1.5110492000000000	-5.1465373000000000	0.61221188999999998
H	-0.56672020000000001	-5.0243764000000004	0.59653723999999997
H	-1.7349357000000001	-5.6089918000000001	1.4419736000000001
O	-1.1787504000000000	-3.9345414999999999	4.3842999999999996
H	-0.39223112999999998	-4.3048434999999996	3.8106575000000000
H	-1.8938514000000000	-3.9791069999999999	3.7074432000000002
O	3.4520476000000002	-3.8011851999999999	-1.1220467000000001
H	2.7153418999999999	-4.1393576000000003	-0.55607174999999998
H	4.2451597999999997	-3.7882175000000000	-0.52774555000000001
O	3.1619863000000001	-5.5109323999999997	4.4866440000000001
H	2.6299041999999999	-4.7400893999999996	4.1998021999999997
H	2.5024199000000000	-6.1888290000000001	4.4921331000000002
O	0.24005708000000001	-3.7129764999999999	-2.3222071000000000
H	0.62501808000000003	-4.5459838000000001	-2.4058628000000000
H	0.15928112999999999	-3.6507288999999998	-1.3040346000000000
O	-2.7090862000000002	-2.7616949000000002	1.0930419000000000
H	-2.1284149000000001	-3.6007254999999998	1.0176016999999999
H	-2.0161923000000002	-2.0003082999999999	1.1457284999999999
O	1.5348430000000000	-3.5864468999999999	6.0074291000000004
H	1.2738536000000000	-4.5011931000000001	6.1238203000000002
H	1.5290798000000001	-3.5289912000000001	5.0640359000000004
O	4.3545657999999996	-6.3487064999999996	0.54127902000000006
H	4.4762269999999997	-7.3263267000000001	0.36362137999999999
H	3.3472914999999999	-6.2708729999999999	0.55954678999999996
O	1.6251709999999999	-7.7451319999999999	3.5729722000000002
H	1.5202004000000000	-7.2157419999999997	2.7785619000000001
H	2.2660377000000000	-8.4117622999999995	3.2506317999999998
O	-0.61160988999999999	-1.3250565999999999	4.8277307000000000
H	-0.84358000000000000	-2.2953453000000001	4.5919847000000003
H	0.35264988000000003	-1.3940482999999999	4.8437276999999996
O	4.7279131999999997	-2.2117572000000001	0.78383636000000001
H	4.7406325000000002	-2.7827676000000001	1.5668118000000000
H	5.1781318000000001	-1.3931861000000001	1.1024300000000000

C	0.14028332E+01	-0.30092635E+01	0.24581465E+01
C	0.18723855E+01	-0.17268199E+01	0.28360600E+01
O	0.17617132E+01	-0.84125694E+00	0.18166192E+01
C	0.12249301E+01	-0.16007592E+01	0.62181759E+00
C	0.10106946E+01	-0.29791535E+01	0.11136972E+01
O	0.23446408E+01	-0.12563426E+01	0.38904146E+01
C	-0.11768025E+00	-0.94994282E+00	0.19535063E+00
O	-0.10278465E+01	-0.96025043E+00	0.12818282E+01
O	0.60871209000000004	-3.9239369000000002	0.31109645000000002
O	1.3276038999999999	-4.0270685000000004	3.3231970000000000
C	0.10559049E+00	0.57250503E+00	-0.21641651E+00
O	-0.10221341E+01	0.10963007E+01	-0.87965156E+00
H	0.20356575E+01	-0.15166379E+01	-0.14994861E+00
H	0.25511847E+00	0.11194532E+01	0.70883299E+00
H	0.10003751E+01	0.63854013E+00	-0.83542015E+00
H	-0.85477051E+00	0.20575853E+01	-0.94723619E+00
H	-0.63879343E+00	-0.16030491E+01	-0.51213780E+00
H	-0.70783127E+00	-0.37056034E+00	0.20099119E+01
O	1.7364733000000001	-6.0796559999999999	1.6099817999999999
H	1.6332405999999999	-5.4692442999999997	2.3788578000000000
H	1.1632754000000001	-5.6121233000000004	0.89801777999999999
O	-1.1349178000000000	-4.0373381000000004	4.2650762999999996
H	-0.29965195000000000	-4.0639830000000003	3.7995461000000001
H	-1.5072684000000001	-4.8829266000000002	3.9179963999999998
O	-1.7865618999999999	-5.2183628000000004	0.54937287999999995
H	-0.86730046000000005	-4.8768921000000001	0.55508199000000003
H	-1.9389525000000001	-5.5678903999999996	1.4912635000000001
O	3.3875247000000002	-3.6818844999999998	-1.0427675999999999
H	2.6716880000000001	-3.8805345000000000	-0.37626853999999998
H	3.9523337999999999	-3.0576496000000000	-0.61728081000000001
O	3.0770951000000002	-5.5517782000000002	4.7204810999999998
H	2.8782668999999999	-4.7599073000000001	4.0972853999999996
H	2.5560478000000000	-6.1959248999999996	4.3180337000000000
O	0.35089641999999999	-4.0369172999999998	-2.2388398999999999
H	1.0605902000000000	-4.6765106999999997	-2.2918658999999999
H	0.31576260000000000	-3.9234011000000000	-1.3114277000000001
O	1.5109699000000001	-3.6907404000000001	6.0414326999999997
H	1.9741413000000001	-4.5838326000000000	5.9510265000000002
H	1.3619829999999999	-3.5405407000000002	5.0599147999999996
O	-0.69582769000000000	-1.4430609000000001	4.8128612000000004
H	-0.79817294000000005	-2.3248761000000000	4.3978735999999996
H	0.24981537000000001	-1.4952953000000000	4.9366346999999999
O	-2.8724270000000001	-2.8974825000000002	0.81147230999999997
H	-2.4023772999999999	-3.7975989999999999	0.64234943000000000
H	-2.1308881000000000	-2.3083505999999998	1.0985328999999999
O	4.4557175999999998	-6.7206789999999996	1.0567572000000001
H	4.1948639999999999	-7.3544546999999998	0.38972510999999999
H	3.5658994000000002	-6.5792780999999998	1.3758893999999999
O	1.8064293000000000	-7.9836619999999998	3.6539142000000000
H	1.7398905000000000	-7.6054268000000000	2.7587464000000002
H	2.5936867000000001	-8.4912509000000007	3.4421574000000001
O	4.7563086999999999	-2.2179468999999998	0.83099933000000004
H	4.8448023999999998	-3.0530636000000002	1.3747761000000001
H	5.2184688000000001	-1.6071924000000000	1.4578070000000001

C	0.15686407E+01	-0.30190364E+01	0.23820805E+01
C	0.19021299E+01	-0.16693253E+01	0.28110732E+01
O	0.16956507E+01	-0.75938340E+00	0.18205836E+01
C	0.12183966E+01	-0.14227252E+01	0.65775658E+00
C	0.10432511E+01	-0.28396683E+01	0.11140341E+01
O	0.23303262E+01	-0.11959088E+01	0.38594107E+01
C	-0.75677386E-01	-0.77798649E+00	0.20415494E+00
O	-0.11107471E+01	-0.80519276E+00	0.12076072E+01
O	0.55871428999999995	-3.7078383000000001	0.32950257999999999
O	1.6117500000000000	-4.1509312999999999	3.1250667999999999
C	0.25712398E+00	0.61923769E+00	-0.31564204E+00
O	-0.84540418E+00	0.92375786E+00	-0.11958927E+01
H	0.19929017E+01	-0.14376617E+01	-0.11293606E+00
H	0.17307470E+00	0.13124297E+01	0.51232962E+00
H	0.11207653E+01	0.66984472E+00	-0.94244026E+00
H	-0.94441311E+00	0.19280768E+01	-0.12037783E+01
H	-0.42918678E+00	-0.13920353E+01	-0.63725819E+00
H	-0.65763113E+00	-0.73962653E+00	0.20418368E+01
O	2.0246374000000000	-6.4254832000000004	1.4233431999999999
H	1.7978750999999999	-5.7066502999999997	2.0706072000000000
H	1.6118812000000000	-6.1238264999999998	0.59788220000000003
O	-1.5483575000000001	-5.1830610999999998	0.62997143000000000
H	-0.79159740000000001	-4.5087278000000000	0.74420312000000000
H	-1.5955109000000001	-5.5451975999999998	1.5377075000000000
O	-0.77653930000000004	-4.4541902000000002	4.3143813000000000
H	0.10504980000000000	-4.4869881999999999	3.8707957999999998
H	-1.3638583000000000	-4.8591781999999997	3.6571305999999999
O	3.0651218999999998	-3.6753396999999999	-1.1879560000000000
H	2.3148599000000001	-4.2143487000000004	-0.90792490000000003
H	3.4535714999999998	-3.4230977999999999	-0.34102207000000001
O	3.3566142999999999	-5.6740832000000001	4.7465149999999996
H	2.8339075999999999	-4.9815851999999996	4.1817298999999997
H	2.8511091999999998	-6.4005520999999996	4.4418838999999997
O	0.29810567999999998	-3.6880765000000002	-2.3389859000000000
H	0.65666809999999998	-4.5595977000000003	-2.3977795000000000
H	0.12581279000000001	-3.7193901000000000	-1.3552119000000000
O	1.7553893000000000	-3.7898309000000001	5.8775085000000002
H	2.3335670999999998	-4.5652920000000003	5.9094122999999996
H	1.4785360999999999	-3.8668548000000000	4.9605338999999997
O	-0.62054739000000003	-1.5402473999999999	4.7403430000000002
H	-0.48497802000000001	-2.4967103000000002	4.5371755000000000
H	0.30565205000000001	-1.1894608000000000	4.7369605999999997
O	-2.9784565999999999	-2.7113931999999998	0.87923951000000000
H	-2.4128234000000002	-3.4812671000000002	0.77374982999999997
H	-2.2939136000000002	-2.0520149999999999	0.82236047000000001
O	4.6244709000000004	-6.7578379999999996	1.0162475000000000
H	4.5762352999999996	-7.6564721999999996	0.64722214000000000
H	3.6445409999999998	-6.6569200999999998	1.3087886000000000
O	4.7820647000000003	-2.1669002000000002	0.48458801000000001
H	4.6684511000000004	-2.7634257999999998	1.2719201000000000
H	5.6239594999999998	-1.7734999000000000	0.83471132000000003
O	1.6123710000000000	-7.9562540999999998	3.8242055000000001
H	1.4873599000000000	-7.5256233000000003	2.9503667999999998
H	2.3945983000000002	-8.5616374999999998	3.5339634000000002

C	0.14857108E+01	-0.28576461E+01	0.23987529E+01
C	0.17869774E+01	-0.15663546E+01	0.28639913E+01
O	0.16022816E+01	-0.64305760E+00	0.18402110E+01
C	0.12463888E+01	-0.13094361E+01	0.60965441E+00
C	0.11747409E+01	-0.27691922E+01	0.10114829E+01
O	0.20822816E+01	-0.11484053E+01	0.40142144E+01
C	-0.16222850E-01	-0.69175585E+00	0.79583253E-01
O	-0.11373806E+01	-0.80436948E+00	0.10225075E+01
O	0.91823957000000000	-3.6974032999999999	0.21031510000000000
O	1.4114176000000000	-3.9069348000000002	3.2281976999999999
C	0.20677906E+00	0.72688462E+00	-0.37996697E+00
O	-0.83346517E+00	0.10935782E+01	-0.11778605E+01
H	0.20489704E+01	-0.11178274E+01	-0.11028847E+00
H	0.25096591E+00	0.13443938E+01	0.54020081E+00
H	0.11381025E+01	0.73580222E+00	-0.92087575E+00
H	-0.11259002E+01	0.20059114E+01	-0.95092875E+00
H	-0.33546142E+00	-0.11930733E+01	-0.79563826E+00
H	-0.82299424E+00	-0.44586506E+00	0.18905115E+01
O	1.7647870000000001	-6.1400613999999996	1.4372175000000000
H	1.4461980000000001	-5.5642247999999999	2.1602665000000001
H	1.6691042000000000	-5.5620563000000001	0.63325224000000002
O	-1.3515995999999999	-5.0775059999999996	0.80331459999999999
H	-0.50933561000000005	-4.6631039000000003	0.85358924000000003
H	-1.3346138000000001	-5.7106224000000001	1.5949925000000000
O	-1.0153090000000000	-4.2648048000000003	4.3990530000000003
H	-0.18244812999999999	-4.2218787000000004	3.8865818999999999
H	-1.3808594000000001	-5.1069075000000002	3.9752399000000000
O	3.3555907000000000	-5.2009397999999996	4.4707781999999998
H	2.6181854000000002	-4.8237832000000003	3.9636828000000000
H	2.9032159000000002	-6.0376592999999996	4.5908870999999998
O	3.2345579999999998	-3.2971455999999999	-1.6694376000000000
H	2.5088265999999999	-3.5632104999999998	-1.0654881000000000
H	3.7332291999999998	-2.6065114999999999	-1.2130280000000000
O	-0.14499980000000001	-3.7637738000000001	-2.3371157999999999
H	6.8036315999999999E-002	-4.7161765999999998	-2.2135117000000002
H	0.15387543000000001	-3.5232770000000002	-1.4367635999999999
O	-2.7813390000000000	-2.8978307999999999	1.0048477000000000
H	-2.2022962000000001	-3.7544453000000000	0.99919073999999997
H	-2.0883459000000002	-2.2220282000000000	0.88395407000000004
O	1.9710318000000000	-3.4122232000000001	6.0337933000000001
H	2.4798019000000000	-4.1907322000000002	5.7975570999999997
H	1.7782688000000000	-3.0937445000000001	5.1162764999999997

O	4.6721883999999996	-2.1098835999999999	0.79724465000000000
H	4.7269148000000003	-2.9280512999999999	1.2947715000000000
H	5.1323319999999999	-1.4560556000000000	1.3754280999999999
O	1.2767694999999999	-7.8659138999999998	3.7202576000000001
H	1.4151088000000001	-7.2916347999999997	2.9774676000000002
H	2.0454895999999998	-8.4810262000000005	3.4921096000000000
O	4.5418228999999997	-6.9542088000000000	1.1667303000000000
H	4.5653639999999998	-7.7187507000000002	0.61719115000000002
H	3.5958874999999999	-6.7203556999999998	1.3129713999999999
O	-1.0158944999999999	-1.5467701000000000	4.4812516999999996
H	-1.1223356000000000	-2.5813022000000001	4.6468318999999996
H	-0.10668606000000000	-1.3908841000000001	4.6315625999999996

C	0.15031963E+01	-0.29591761E+01	0.25488906E+01
C	0.18237702E+01	-0.15893718E+01	0.30489554E+01
O	0.17986238E+01	-0.67182866E+00	0.20145519E+01
C	0.14925750E+01	-0.14194327E+01	0.78837526E+00
C	0.13409976E+01	-0.28341760E+01	0.11856383E+01
O	0.20964601E+01	-0.11331928E+01	0.41539814E+01
C	0.25237767E+00	-0.83555382E+00	0.51544332E-01
O	-0.95191510E+00	-0.86323664E+00	0.86578519E+00
O	1.1313553999999999	-3.7630322000000000	0.32298466999999997
O	1.3851580999999999	-4.0680835000000002	3.2286614000000000
C	0.55059806E+00	0.56298491E+00	-0.39075381E+00
O	-0.62022504E+00	0.11218903E+01	-0.10826748E+01
H	0.23872600E+01	-0.12192338E+01	0.83967331E-01
H	0.65228804E+00	0.11634454E+01	0.55144524E+00
H	0.13697366E+01	0.49428946E+00	-0.10644750E+01
H	-0.31402900E+00	0.20233467E+01	-0.11364950E+01
H	0.35065319E-01	-0.14373946E+01	-0.85830300E+00
H	-0.81177332E+00	-0.21011633E+00	0.15878995E+01
O	1.8437669999999999	-6.1908446000000001	1.5566936000000000
H	1.6918688000000000	-5.5063043000000000	2.2491720000000002
H	1.4590057000000001	-5.6226076999999997	0.81826403999999997
O	-1.2339173999999999	-5.1661902000000000	0.50302036000000006
H	-0.38232355000000001	-4.7660482999999996	0.70905059000000004
H	-1.4281117000000001	-5.6045138999999997	1.3324666000000001
O	3.4578397000000001	-5.1078679999999999	4.1134914000000000
H	2.7700255999999999	-4.7015744000000002	3.6262511000000002
H	3.2147529000000001	-6.0384846999999997	4.0522140000000002
O	-0.97549472000000004	-4.1491536000000000	4.4341013000000000
H	-0.16401779000000000	-4.1340271000000000	3.8568593000000000
H	-1.4623381000000000	-4.8631827999999997	4.0113481000000002
O	3.5337811000000001	-3.3030290000000000	-1.1877025999999999
H	2.6509966000000000	-3.5047742000000000	-0.86337671000000005
H	3.9090462000000001	-3.0975844000000001	-0.29068872000000001
O	-2.8116927000000000E-002	-3.9905336999999999	-2.1436434000000002
H	8.1974501000000005E-002	-4.9438551000000004	-2.2412033000000000
H	6.0913598999999999E-002	-3.9294563999999998	-1.2084937000000000
O	1.8903323999999999	-3.9118347000000000	6.0238315000000000
H	2.5403139000000001	-4.6889475999999997	5.8872543999999998
H	1.6023284000000000	-3.8149856000000000	5.0885793000000001
O	1.7062552000000000	-7.7091598000000001	3.7157979000000001
H	1.7183717999999999	-7.3793419000000000	2.7966017000000001
H	2.3046217000000002	-8.4591360000000009	3.7070934000000002
O	-2.7942849999999999	-3.0743117999999998	0.78584089999999995
H	-2.2665931000000001	-3.8846177000000002	0.56921261999999995
H	-2.1138938999999999	-2.3802021000000000	1.0370953000000001
O	4.4249342000000000	-7.1333036999999999	1.4967884000000000
H	4.2168633000000000	-7.5649125000000002	0.59796053999999998
H	3.5096316999999999	-6.8883928000000001	1.7949332000000000
O	-0.82555928999999995	-1.3711005999999999	4.9398932999999996
H	-0.99452445000000000	-2.2864341000000001	4.6916482999999998
H	0.15037563000000001	-1.4427542000000000	5.1408506999999997
O	5.6296897000000001	-4.4960880999999997	2.4769874000000001
H	5.5021613000000000	-4.9322844999999997	1.6137092000000000
H	4.9682307000000003	-5.0089388000000001	3.0536431999999998

C	0.15339471E+01	-0.30045858E+01	0.26130672E+01
C	0.17059147E+01	-0.17148470E+01	0.31114058E+01
O	0.16245936E+01	-0.78442501E+00	0.20237794E+01
C	0.14382958E+01	-0.14063494E+01	0.79388657E+00
C	0.14230631E+01	-0.29120177E+01	0.11797100E+01
O	0.19493885E+01	-0.11923206E+01	0.42239997E+01
C	0.22358298E+00	-0.89321787E+00	0.47378581E-01
O	-0.93221521E+00	-0.91293754E+00	0.93839320E+00
O	1.2227980000000001	-3.8479988999999999	0.36847990000000003
O	1.4219560000000000	-4.1464816000000004	3.3287105000000001
C	0.45690546E+00	0.55472457E+00	-0.45537762E+00
O	-0.69530555E+00	0.10744513E+01	-0.11144371E+01
H	0.23657517E+01	-0.11466632E+01	0.20532494E+00
H	0.84812385E+00	0.12439636E+01	0.33838266E+00
H	0.12441129E+01	0.51626608E+00	-0.12022512E+01
H	-0.87999560E+00	0.19093994E+01	-0.67134494E+00
H	-0.12647592E+00	-0.15374702E+01	-0.78725821E+00
H	-0.75774840E+00	-0.45356032E+00	0.18029163E+01
O	2.1187697000000001	-6.2146324999999996	1.5010128000000000
H	1.9169936000000001	-5.4288334999999996	2.0798003999999999
H	1.6354481000000001	-5.7970804999999999	0.78756919000000003
O	-1.1501423000000000	-5.2763831000000003	0.40352558999999999
H	-0.21615248000000001	-4.9131966000000000	0.45780704000000000
H	-1.3841654000000001	-5.3385721000000004	1.3394670000000000
O	-1.1653099000000000	-4.0451357999999997	4.2612652999999998
H	-0.26444526000000002	-4.1986480000000004	3.9455594999999999
H	-1.6423679000000000	-4.4540379999999997	3.5313452999999999
O	3.7880707999999998	-5.3319169000000004	4.1923066000000002
H	2.9866744999999999	-4.7224632000000000	4.0283540000000002
H	3.4604471000000001	-6.1284552999999997	4.5490496000000000
O	3.7045878000000001	-3.3232349000000001	-0.98764372000000000
H	2.9707593999999999	-3.5859344000000002	-0.46631756000000002
H	4.2496555999999996	-2.8010931999999999	-0.39478822000000002
O	0.15975634999999999	-3.8364338999999998	-2.2855037999999999
H	0.19166960999999999	-4.7657144000000002	-2.3470312000000000
H	0.39980453999999999	-3.7043702000000001	-1.3392986000000000
O	1.6966611000000000	-4.2093106000000002	6.1078261999999999
H	2.4868119000000002	-4.7439518999999999	5.9863530000000003
H	1.4961333999999999	-3.9771149000000001	5.2072732999999998
O	1.4191621999999999	-7.9349926999999996	3.7065153999999998
H	1.4252172999999999	-7.2097648999999997	3.0260452000000000
H	2.0869010000000001	-8.5040600000000008	3.3863132999999999
O	4.7058989999999996	-6.9192815000000003	1.3380985999999999
H	4.4985102000000001	-7.6435233000000000	0.69180819999999998
H	3.7844072000000000	-6.6085956000000001	1.5262035000000000
O	-2.7842620999999999	-3.1092871000000000	0.36871675999999998
H	-2.2480638000000002	-3.9439335000000000	0.33978461999999998
H	-2.0830457000000000	-2.4653290000000001	0.45491131000000001
O	-0.71520373999999998	-1.3498451000000000	4.7039907999999997
H	-0.86175323999999998	-2.3188941999999999	4.7373173000000000
H	0.22709304999999999	-1.3906970000000001	4.6086894000000003
O	5.6004379999999996	-4.6078703000000001	2.4011616000000000
H	5.5958177999999998	-5.3991107999999999	1.7328273999999999
H	4.9932192999999998	-4.9808062000000000	3.0538932000000001

C	0.16019092E+01	-0.27646708E+01	0.24436244E+01
C	0.18309432E+01	-0.15257748E+01	0.30079831E+01
O	0.16922494E+01	-0.52897412E+00	0.19884341E+01
C	0.16073505E+01	-0.12038975E+01	0.72114931E+00
C	0.16098950E+01	-0.26112330E+01	0.10426953E+01
O	0.20738336E+01	-0.11558369E+01	0.41814799E+01
C	0.40817646E+00	-0.71794903E+00	-0.95040229E-01
O	-0.77981619E+00	-0.83518769E+00	0.75549616E+00
O	1.4127433000000000	-3.5665174999999998	0.22444715000000001
O	1.5348474000000001	-3.8960708999999998	3.1819329000000001
C	0.44551483E+00	0.76101485E+00	-0.46719246E+00
O	-0.61540995E+00	0.96263101E+00	-0.15074602E+01
H	0.25500623E+01	-0.96751538E+00	0.21051363E+00
H	0.13840079E+00	0.13634368E+01	0.44706535E+00
H	0.14523772E+01	0.10474265E+01	-0.82976941E+00
H	-0.97087209E+00	0.18399246E+01	-0.14714635E+01
H	0.24400563E+00	-0.13600473E+01	-0.94278434E+00
H	-0.53578229E+00	-0.43599749E+00	0.15969685E+01
O	1.8528574000000000	-6.0614122000000004	1.5639571000000001
H	1.6291815000000001	-5.3820034000000003	2.2738996000000000
H	1.6970202999999999	-5.5190982000000002	0.78090731999999996
O	-0.83473523000000005	-5.2663266000000002	0.54462906000000000
H	5.6797981999999997E-002	-4.8656611999999999	0.68138935000000000
H	-0.96760758000000002	-5.6026004000000000	1.4003274000000001
O	3.6156366000000002	-5.6316290999999996	4.2177695000000002
H	2.9717840000000000	-5.1527240000000001	3.6134724000000000
H	2.9883101999999999	-6.2498202999999997	4.6103481000000004
O	-1.0640775000000000	-4.1844919000000003	4.3531434999999998
H	-0.18513336999999999	-4.1926430999999997	3.9803768000000002

H	-1.4681929000000000	-4.8457781999999998	3.7311052000000000
O	4.0943186000000003	-3.1311043000000001	-1.1696196000000001
H	3.2219006000000001	-3.2405648000000000	-0.79954440000000004
H	4.5534910999999996	-2.7633372000000000	-0.38707000000000003
O	-0.14805317000000001	-3.9450870000000000	-2.1989356000000000
H	0.15058864000000000	-4.8556653000000001	-1.9863948000000000
H	1.4257868000000000E-004	-3.6223334999999999	-1.2686814000000000
O	4.4814122000000003	-6.8029567000000002	1.0416314000000000
H	4.2619316999999999	-7.7074623999999998	1.1109062000000001
H	3.5831225999999998	-6.3183080000000000	0.97972568000000004
O	1.9182386000000000	-3.8041426000000000	6.0783250000000004
H	2.5599598000000001	-4.4599739999999999	6.3538186999999997
H	1.8629078999999999	-3.7146357999999999	5.1238960999999996
O	-2.5893663000000000	-2.7997725000000000	1.2242618000000001
H	-1.9420744999999999	-3.5009766999999998	1.2608385000000000
H	-1.9446547999999999	-2.1014130999999998	0.95212096000000002
O	1.2313921999999999	-7.7677171999999999	3.4636518000000001
H	1.4120763999999999	-7.2923160999999999	2.7132122000000001
H	2.0510096999999998	-8.3914784000000004	3.4047239000000000
O	-0.95241931999999996	-1.2280989000000000	4.3555786000000003
H	-1.1105465999999999	-2.1744854000000000	4.3369869999999997
H	5.2626903000000003E-002	-1.3142081000000001	4.2405635999999998
O	5.2608074000000000	-2.2628496999999999	1.1233378999999999
H	5.3394066000000002	-3.1260669999999999	1.5740765000000001
H	5.8231313000000000	-1.6279416000000000	1.6324988000000000

C	0.15436674E+01	-0.26734821E+01	0.25162954E+01
C	0.18725575E+01	-0.14143625E+01	0.30001479E+01
O	0.17986056E+01	-0.40560699E+00	0.19372802E+01
C	0.14997864E+01	-0.11137019E+01	0.67678568E+00
C	0.14699127E+01	-0.25518971E+01	0.11417748E+01
O	0.22018243E+01	-0.10127382E+01	0.41692889E+01
C	0.27092757E+00	-0.56289340E+00	-0.30280634E-01
O	-0.82453183E+00	-0.61899759E+00	0.83874675E+00
O	1.3580186000000001	-3.4510421999999998	0.21346752999999999
O	1.4707340000000000	-3.8286034000000000	3.2225852000000001
C	0.42314417E+00	0.86283363E+00	-0.54391832E+00
O	-0.77265433E+00	0.12933927E+01	-0.12090822E+01
H	0.24111545E+01	-0.10419076E+01	0.51040874E-01
H	0.66426891E+00	0.15959879E+01	0.24524679E+00
H	0.12883616E+01	0.88688501E+00	-0.11546211E+01
H	-0.72551390E+00	0.22942453E+01	-0.10078922E+01
H	0.94965069E-01	-0.12042905E+01	-0.91605177E+00
H	-0.77743351E+00	0.17864949E+00	0.14154999E+01
O	2.0730984000000001	-5.9823082000000003	1.7590005000000000
H	1.9122110000000001	-5.2047616999999997	2.3430491999999998
H	1.6023037000000000	-5.6457816999999997	0.96901663000000005
O	-0.97480365000000002	-5.0684649000000004	0.43451636999999999
H	-0.17674843000000001	-4.5371414000000003	0.60381488999999999
H	-1.1357221000000000	-5.4131988000000000	1.3181045000000000
O	-1.0494403999999999	-4.0791437999999998	4.2768842999999999
H	-9.7181428000000000E-002	-3.8492690999999999	3.9785742000000002
H	-1.2284383000000001	-4.8093810000000001	3.6980697000000000
O	3.6671648000000001	-5.2134704000000003	4.1956585000000004
H	2.8877427999999998	-4.7139835999999997	3.9293809000000000
H	3.4995875999999999	-6.0516693000000004	3.7589495999999998
O	4.0009570999999999	-3.1119675999999998	-1.0834851999999999
H	3.1961738999999998	-3.1617004999999998	-0.58636168000000000
H	4.5982798999999996	-3.1362733999999999	-0.32219004000000001
O	0.11882463000000000	-3.8245423999999999	-2.3261280000000002
H	0.36647344999999998	-4.7611711999999997	-2.4302134000000000
H	9.6162355000000005E-002	-3.7111926999999998	-1.3063895000000001
O	1.4869664000000000	-3.9605570000000001	5.9803721999999997
H	1.7753254000000001	-4.8762610999999998	5.7016217999999999
H	1.4951532000000001	-3.5661596000000002	5.1009178999999998
O	4.5083473999999999	-6.9435038000000002	1.4856760000000000
H	4.2280841999999996	-7.4149867000000000	0.68844910000000004
H	3.6940721999999999	-6.4298212000000001	1.6043137000000001
O	-2.5843908999999998	-2.5890306999999999	1.3192697000000000
H	-2.1511307000000000	-3.2947891999999999	0.86409988999999998
H	-1.9378280999999999	-1.8587753000000000	1.2617792999999999
O	1.3613862999999999	-7.7426640000000004	3.5442806000000000
H	1.3765383000000000	-7.1915187999999999	2.7944909000000000
H	1.8363985000000000	-8.5406864999999996	3.2286320000000002
O	-1.0495156000000001	-1.3739238000000000	4.1391121999999996
H	-1.0290940000000000	-2.3906342999999999	4.2434286999999999

H	-8.9498817999999994E-002	-1.2381206000000000	4.1687099999999999
O	5.0148315999999999	-2.4002227999999999	1.2704389000000000
H	5.3758504000000000	-3.0993949000000001	1.8418424000000000
H	5.4899996000000000	-1.6167654000000000	1.6211921000000000

C	0.15012376E+01	-0.26923015E+01	0.24924109E+01
C	0.17660620E+01	-0.13515910E+01	0.29226302E+01
O	0.19225876E+01	-0.45613624E+00	0.18865074E+01
C	0.14463008E+01	-0.10982164E+01	0.64473711E+00
C	0.13562997E+01	-0.25738431E+01	0.11077410E+01
O	0.18447682E+01	-0.91183169E+00	0.41370984E+01
C	0.14716005E+00	-0.37602346E+00	0.67879738E-01
O	-0.91527385E+00	-0.50110869E+00	0.10473032E+01
O	1.3886598999999999	-3.5413196999999998	0.21238604999999999
O	1.5603636999999999	-3.7285488000000000	3.3006837999999998
C	0.32035501E+00	0.10997451E+01	-0.22811500E+00
O	-0.73905918E+00	0.14165581E+01	-0.12021281E+01
H	0.22418194E+01	-0.84462053E+00	-0.92515317E-01
H	0.18086343E+00	0.17428738E+01	0.65586028E+00
H	0.12927268E+01	0.12395447E+01	-0.61444950E+00
H	-0.10447705E+01	0.23512287E+01	-0.11313958E+01
H	-0.15025539E+00	-0.90367486E+00	-0.89626612E+00
H	-0.59628161E+00	-0.44717058E+00	0.18921863E+01
O	1.9106894999999999	-5.8712771000000004	1.7029840000000001
H	1.6780200999999999	-5.2083795000000004	2.4030754999999999
H	1.6763885999999999	-5.3026958999999998	0.93947654999999997
O	-1.0206816000000001	-5.0704019999999996	0.26843869999999997
H	-0.13869509999999999	-4.7186262000000001	0.46483616000000000
H	-1.2434390000000000	-5.5118242999999998	1.1247788999999999
O	-1.0927975000000001	-4.2391813999999997	4.2218768999999998
H	-0.15709616000000001	-4.3299849000000004	3.7831237999999998
H	-1.6117192000000000	-4.8872990999999999	3.6628639000000001
O	3.6059823000000000	-5.2097188000000001	4.2729654000000004
H	2.9527749000000001	-4.6242840000000003	3.8478159000000001
H	3.1888226999999998	-6.0940966999999997	4.1340776999999997
O	3.7525738000000000	-3.3021389999999999	-1.1344966999999999
H	3.1121286000000001	-3.3935602999999999	-0.47285560999999998
H	4.4387620999999999	-2.7329780000000001	-0.63391565999999999
O	0.22858426000000001	-4.0177981999999997	-2.2143782999999999
H	0.44378169000000001	-4.9574762999999997	-2.1405698000000002
H	0.29794974000000002	-3.7230900000000000	-1.2808670000000000
O	1.6631073000000001	-4.0498491000000003	5.9578457000000000
H	2.3696720000000000	-4.6484648999999996	5.6905378000000004
H	1.3840399000000001	-3.8236227999999999	5.0469426999999998
O	4.3626389000000003	-6.8125765999999999	0.94454945999999995
H	4.0904154999999998	-7.7141491000000002	0.74140344000000002
H	3.5070063999999999	-6.5163067000000003	1.2169619000000000
O	1.5197601000000001	-7.6947972000000000	3.6694046000000000
H	1.5946590000000000	-7.0967165999999997	2.8576904999999999
H	2.0148693000000000	-8.4084073000000004	3.2818385999999999
O	5.0371800999999996	-2.2125669000000001	0.92905262000000000
H	5.1167495000000001	-3.0772647000000002	1.3954061000000000
H	5.5114752999999999	-1.6412059999999999	1.5098114000000000
O	-2.6463054000000001	-2.7470412000000000	0.92665050000000004
H	-2.1581144999999999	-3.5071229000000002	0.59977115999999997
H	-1.8950240000000000	-2.0883354000000001	1.0924630000000000
O	-0.86210040000000003	-1.4988277999999999	4.6346186999999999
H	-0.97388368999999997	-2.5225759999999999	4.6544713000000000
H	7.2038545999999995E-002	-1.4758148000000000	4.8973008000000000

C	0.15272479E+01	-0.26111224E+01	0.24286940E+01
C	0.19321046E+01	-0.13241917E+01	0.29272073E+01
O	0.18297018E+01	-0.34475325E+00	0.19797035E+01
C	0.14775802E+01	-0.93031821E+00	0.71889999E+00
C	0.13294007E+01	-0.24864976E+01	0.10825658E+01
O	0.22889765E+01	-0.98201691E+00	0.40469109E+01
C	0.17575745E+00	-0.39436555E+00	0.88096624E-01
O	-0.94207874E+00	-0.78717982E+00	0.91461773E+00
O	1.2254687000000000	-3.3980541999999998	0.17298530000000001
O	1.5362382000000001	-3.7220463000000001	3.1831589999999998
C	0.19890691E+00	0.11431093E+01	-0.16919237E+00
O	-0.90756652E+00	0.17003394E+01	-0.94927660E+00
H	0.23025272E+01	-0.73809589E+00	0.33563561E-01
H	0.19696410E+00	0.16853146E+01	0.77647955E+00
H	0.10881939E+01	0.12261338E+01	-0.85390558E+00
H	-0.10846680E+01	0.25194589E+01	-0.46921094E+00

H	0.14587750E-01	-0.90685295E+00	-0.86584702E+00
H	-0.53515648E+00	-0.87358406E+00	0.17945224E+01
O	1.8157395999999999	-5.9079573999999999	1.7693132000000000
H	1.7326657000000001	-5.2503498999999998	2.5186343000000000
H	1.7880902000000001	-5.2358212000000002	1.0946830999999999
O	-0.96881930999999999	-4.8029050000000000	0.53455951999999995
H	-6.3733308000000002E-002	-4.5303145999999996	0.42359811000000003
H	-0.97766989999999998	-5.3648870999999998	1.3430603999999999
O	-1.0590577999999999	-4.2751418000000001	4.2519985000000000
H	-0.11451172000000000	-4.1041026000000000	4.1310253000000001
H	-1.1394508000000001	-5.1286949999999996	3.8549093999999999
O	3.9082496000000000	-3.5937252000000002	-1.3217386000000000
H	3.1676704000000000	-3.4393582999999999	-0.72148332999999998
H	4.5895865000000002	-3.0063157000000000	-0.83602164000000001
O	3.7319040999999999	-5.2055270000000000	4.1541107000000004
H	2.9758148000000002	-4.5987210999999997	4.0052950999999997
H	3.3551422000000000	-5.9798413000000004	3.8914773999999999
O	8.2708639000000000E-002	-3.8350602000000000	-2.3503759999999998
H	0.43701847999999999	-4.7044132000000003	-2.6507453999999999
H	0.24907366000000000	-3.9021306999999998	-1.3550560000000000
O	1.6118767000000001	-3.8740896999999999	6.0105877999999997
H	2.1691449999999999	-4.6136825999999997	6.0166298999999999
H	1.6130941000000001	-3.6740439000000000	5.0110241000000002
O	4.3856636000000000	-6.8308241000000001	1.3044560999999999
H	4.2444376000000004	-7.6482424000000000	0.76959661000000001
H	3.5343068000000000	-6.3634861000000003	1.4637673000000000
O	-2.7206869000000000	-2.7565957000000001	0.91303676000000000
H	-2.3010698000000001	-3.5876025000000000	0.65395678999999995
H	-1.8969826000000001	-2.1750994000000001	0.81514710999999995
O	-0.72367835000000003	-1.6126948999999999	4.8197899000000000
H	-0.91408542999999998	-2.5634936000000001	4.4960975000000003
H	0.25406687000000000	-1.6717910000000000	4.8468001000000003
O	0.93799025000000003	-8.0301895999999999	3.4921579000000000
H	1.3370834000000000	-7.3976476000000000	2.8290611000000001
H	1.6679107000000000	-8.7486239000000001	3.5415763999999998
O	-1.5400840000000000	-6.4641887000000002	2.6968709999999998
H	-1.3662780999999999	-7.3765621000000001	3.0060292000000000
H	-2.5201788000000001	-6.4759630000000001	2.9926154000000000

C	0.15383664E+01	-0.26431354E+01	0.25435197E+01
C	0.18253982E+01	-0.13091504E+01	0.30155248E+01
O	0.15753079E+01	-0.29937709E+00	0.20052917E+01
C	0.13551075E+01	-0.10444360E+01	0.73772322E+00
C	0.14023812E+01	-0.25458709E+01	0.11366968E+01
O	0.20890824E+01	-0.87572364E+00	0.41734165E+01
C	0.79910181E-01	-0.49338954E+00	0.63685521E-02
O	-0.10900298E+01	-0.69663298E+00	0.78363701E+00
O	1.2638663999999999	-3.5369896000000001	0.28479732000000002
O	1.4421590000000000	-3.7654196999999998	3.3383967000000001
C	0.27146061E+00	0.10070107E+01	-0.20204517E+00
O	-0.85097717E+00	0.15323307E+01	-0.95119444E+00
H	0.22752098E+01	-0.91552083E+00	0.89002999E-01
H	0.43151950E+00	0.13977922E+01	0.79483317E+00
H	0.12098571E+01	0.10947962E+01	-0.81718706E+00
H	-0.10187253E+01	0.24472457E+01	-0.67844671E+00
H	-0.44763091E-01	-0.10270971E+01	-0.94835357E+00
H	-0.91541744E+00	-0.61502123E+00	0.17564737E+01
O	1.7510095000000001	-5.9333741000000000	1.7096183000000000
H	1.7376229000000001	-5.1926777000000001	2.4053320000000000
H	1.5682843000000000	-5.2965777000000003	0.93982966999999995
O	-0.95049262000000001	-5.1829917999999999	0.40053535000000001
H	-8.9816410999999999E-002	-4.7140534000000001	0.53715225000000000
H	-1.1355427000000000	-5.4357898000000002	1.3412762000000000
O	-0.92991970999999995	-4.0245429000000001	4.4214257000000003
H	-0.10005430000000000	-3.8367727999999999	3.9086682000000001
H	-1.3675358000000000	-4.5993709000000003	3.7882609000000000
O	3.7329257999999998	-5.1124872999999997	4.0394205999999997
H	3.0221868999999999	-4.3948137999999997	4.0216691000000004
H	3.2184211999999999	-5.9146701999999998	3.8060280999999998
O	-6.2269247999999999E-002	-3.7867931000000001	-2.0910332000000000
H	-2.9154333000000001E-002	-4.7520734999999998	-2.2771336000000000
H	0.45007093999999997	-3.6851101000000002	-1.2467714000000001
O	3.8133021000000000	-3.7379392000000000	-1.5363635000000000
H	3.0060099999999998	-4.0078693000000003	-1.0253414000000001
H	4.3787817999999996	-3.2771360000000000	-0.82689740000000000
O	1.6931229000000001	-4.0196611999999998	6.1250643000000000
H	2.3804631999999999	-4.8043325000000001	6.0730544000000002
H	1.4792543000000000	-4.0346380999999996	5.1590638000000002
O	4.3211196999999997	-6.6779614000000000	0.93852846000000001
H	4.1907696999999997	-7.4948075000000003	0.43037670000000000
H	3.3940530000000000	-6.4153608999999996	1.2204077000000000
O	-0.82279837000000000	-1.5116061000000001	4.6210117000000004
H	-1.0604363999999999	-2.4078281000000001	4.2816967000000004
H	7.4035803999999997E-002	-1.6337489000000001	4.9086949000000004
O	1.3301338000000000	-7.9147732000000000	3.5110879000000002
H	1.3622810999999999	-7.2628998999999999	2.8197942999999999
H	2.0438729000000002	-8.5931025999999999	3.1970926000000000
O	-1.3355642999999999	-6.1740133999999998	2.9593056000000000
H	-0.69731040000000000	-6.8638250999999997	3.1995784999999999
H	-2.1872571999999999	-6.5539895000000001	3.2595784999999999
O	5.8285863999999998	-4.6378271000000000	2.6093651000000002
H	5.5232543999999999	-5.4336358999999996	2.0757884000000000
H	4.9700563999999998	-4.5159098000000002	3.0263662000000000

C	0.15695037E+01	-0.26664354E+01	0.25835940E+01
C	0.17950055E+01	-0.13111260E+01	0.29763773E+01
O	0.17420916E+01	-0.39912410E+00	0.19521569E+01
C	0.15273337E+01	-0.11235507E+01	0.67660233E+00
C	0.15130291E+01	-0.26209812E+01	0.11734537E+01
O	0.18340964E+01	-0.79906026E+00	0.41519687E+01
C	0.21444302E+00	-0.63255061E+00	0.11112860E-02
O	-0.94752756E+00	-0.10215052E+01	0.71661289E+00
O	1.3235030999999999	-3.6274920000000002	0.34169643000000000
O	1.5479655999999999	-3.6564850000000000	3.4694680999999998
C	0.35762666E+00	0.96420709E+00	-0.34121033E-01
O	-0.71755060E+00	0.15346777E+01	-0.90649834E+00
H	0.23667551E+01	-0.10374159E+01	0.10535516E-01
H	0.26371447E+00	0.13397798E+01	0.10193263E+01
H	0.13347571E+01	0.12019791E+01	-0.46881904E+00
H	-0.10316166E+01	0.23912225E+01	-0.55137438E+00
H	0.84948358E-01	-0.11204087E+01	-0.10019725E+01
H	-0.10312885E+01	-0.52205130E+00	0.14853141E+01
O	1.8736140999999999	-5.8048264999999999	1.7202691999999999
H	2.0047882000000001	-5.1147860999999999	2.3813141999999998
H	1.5402263000000000	-5.2267030999999999	0.98293810000000004
O	-1.1210099000000000	-5.2390977000000003	0.35306440000000000
H	-0.45041236000000001	-4.5265649000000003	0.40973083999999999
H	-1.0317628999999999	-5.6153493000000001	1.2777567999999999
O	-1.0719197000000000	-4.3239549999999998	4.2732690000000000
H	-0.16432379000000000	-4.2849119000000000	3.9739447000000001
H	-1.4157573999999999	-5.0903299000000004	3.8496793999999999
O	3.6529964000000001	-5.4586147000000000	3.9578997000000000
H	3.1103906000000001	-4.6630013999999997	3.8984139000000000
H	3.3762929000000002	-5.9397985999999996	3.2066067000000000
O	3.6285693000000001	-4.0715903999999998	-1.2623886000000000
H	2.8782717000000000	-4.3446537999999997	-0.78326247000000004
H	4.0718236000000001	-3.4262687999999999	-0.66383709000000002
O	-7.9359369999999999E-002	-3.4948879000000002	-2.0497744000000000
H	2.8714673000000000E-002	-4.2941899000000001	-2.6520120000000000
H	0.36554818000000000	-3.7656809999999998	-1.2035708000000001
O	-0.90961276999999996	-1.3044730000000000	4.3835379999999997
H	-0.94131821000000004	-2.2353437000000000	4.0887754999999997
H	4.2680460000000003E-002	-1.3166960000000001	4.6672406000000004
O	4.2136903999999999	-6.7672898999999997	1.0706895000000001
H	4.1526787000000001	-7.6235910000000002	0.61309305000000003
H	3.3408809000000002	-6.4890540999999997	0.93544958000000000
O	1.7600739000000001	-3.9603587999999998	6.2094950000000004
H	2.1887797000000000	-4.8027397000000001	6.0471639000000004
H	1.1797854000000001	-3.8919489000000000	5.4554831000000004
O	1.0636692999999999	-8.0839797999999998	3.0457036000000000
H	1.4924767999999999	-7.3888175000000000	2.5168558999999999
H	1.7076112999999999	-8.8034683000000005	3.0438071999999998
O	-1.1219679000000000	-6.5235298999999998	2.6687256000000001
H	-0.32599983999999999	-7.1022150000000002	2.6396801000000001
H	-1.7870638000000001	-7.1559344999999999	3.0986563000000000
O	-2.9469959999999999	-3.2594077000000001	0.82414027000000001
H	-2.4334617999999999	-3.9169006000000000	0.30073191999999999
H	-2.2933549000000002	-2.5299073999999999	0.63563265999999996

C	0.16538927E+01	-0.26938123E+01	0.26080880E+01
C	0.18239274E+01	-0.13489238E+01	0.30164745E+01
O	0.17096949E+01	-0.46157986E+00	0.19835723E+01

C	0.13338591E+01	-0.12397239E+01	0.76681073E+00
C	0.13301804E+01	-0.27130987E+01	0.12408658E+01
O	0.19841294E+01	-0.93104698E+00	0.41739996E+01
C	0.27245421E-01	-0.71772742E+00	0.15564851E+00
O	-0.10654091E+01	-0.98319844E+00	0.91317449E+00
O	1.1120395000000001	-3.7198300999999998	0.39023318000000001
O	1.6229582000000000	-3.7284798000000001	3.5186166999999999
C	0.31841511E+00	0.78730522E+00	-0.66135676E-02
O	-0.78889505E+00	0.14244591E+01	-0.61044060E+00
H	0.21720666E+01	-0.11104968E+01	0.69145286E-01
H	0.61420317E+00	0.12494916E+01	0.93822754E+00
H	0.12791222E+01	0.85512891E+00	-0.55711450E+00
H	-0.93555248E+00	0.22132671E+01	-0.12172661E+00
H	-0.13028388E+00	-0.12465223E+01	-0.84785771E+00
H	-0.10353888E+01	-0.57400269E+00	0.17792690E+01
O	1.8995567000000000	-5.7919565999999998	1.7589096000000000
H	1.9224635000000001	-5.0740778999999998	2.3966446000000001
H	1.5408980000000001	-5.2674896000000002	0.98681189999999996
O	-1.0836763000000000	-4.2794628000000001	4.2585753999999998
H	-0.14862908000000000	-4.0798151000000002	3.9660386999999999
H	-1.2789889999999999	-5.0512800999999996	3.6797035000000000
O	3.5851850999999999	-5.6514176000000003	3.9032613999999999
H	2.8243681999999999	-5.1385053999999997	3.6224904000000002
H	3.2918987000000000	-6.4997074000000001	3.6041962999999999
O	-1.3424805000000000	-5.2336780999999997	0.30061580999999998
H	-0.52200528000000002	-4.6556924999999998	0.34825326000000001
H	-1.2992718000000001	-5.6271956000000003	1.2088926000000000
O	3.5008918000000002	-3.8287195000000001	-1.3524744000000000
H	2.8905468999999999	-4.2373713999999998	-0.73020978000000003
H	4.0745247000000004	-3.4174454000000001	-0.72812655999999998
O	0.14180553000000001	-3.5200456000000000	-2.0326792000000000
H	7.0477162999999995E-002	-4.4693465999999997	-2.0526857999999999
H	0.50983827000000004	-3.3898100000000002	-1.1173211999999999
O	4.2648989999999998	-6.5795171000000003	0.86969015000000005
H	3.8623449000000001	-7.1165735000000003	9.9596884999999996E-002
H	3.4911641000000002	-6.0242582999999996	1.0269752999999999
O	0.83055972000000000	-7.9718673999999998	2.9095515999999999
H	1.1856633000000001	-7.2080446000000000	2.4045302999999998
H	1.5063157000000000	-8.6412850999999993	2.6047094000000000
O	-0.99982705000000005	-1.2790340000000000	4.0431312000000004
H	-1.1332409000000001	-2.2632352000000000	3.9601454000000000
H	-0.14564224000000001	-1.2445476000000000	4.4129512999999996
O	1.8482611000000000	-4.0317474999999998	6.5496758000000002
H	2.4244378000000002	-4.7335599999999998	6.2531768000000003
H	1.4340605000000000	-3.9638211999999999	5.6836228999999996
O	-1.4382438000000000	-6.3670761999999996	2.7493813000000000
H	-0.64802152999999996	-6.8921527999999999	2.8708630999999998
H	-2.0576699000000001	-7.0098267999999999	3.1738062999999999
O	4.9232873000000001	-2.6082011000000001	0.45893873000000002
H	5.1354255000000002	-3.3260814999999999	1.0738637000000000
H	5.2241030000000004	-1.7820571999999999	0.99136590999999996

C	0.16740883E+01	-0.27412630E+01	0.27685966E+01
C	0.17498577E+01	-0.13782966E+01	0.31632061E+01
O	0.16081440E+01	-0.43889482E+00	0.21987974E+01
C	0.14516949E+01	-0.12058991E+01	0.92395379E+00
C	0.14694952E+01	-0.26463315E+01	0.13802597E+01
O	0.18813220E+01	-0.88918413E+00	0.43330184E+01
C	0.24368099E+00	-0.67794686E+00	0.18942350E+00
O	-0.91336767E+00	-0.74036462E+00	0.10323557E+01
O	1.3713291999999999	-3.5612512000000001	0.47732426999999999
O	1.7632867999999999	-3.7828023000000002	3.5281991000000001
C	0.59323465E+00	0.71243676E+00	-0.25424156E+00
O	-0.59612531E+00	0.12504333E+01	-0.85853381E+00
H	0.23324397E+01	-0.94423801E+00	0.31907882E+00
H	0.83255911E+00	0.13226612E+01	0.60199411E+00
H	0.14487721E+01	0.73349956E+00	-0.91582569E+00
H	-0.54439557E+00	0.22132774E+01	-0.76948857E+00
H	0.10559428E+00	-0.13001144E+01	-0.72914561E+00
H	-0.56268224E+00	-0.47630691E+00	0.19151931E+01
O	1.5512927999999999	-5.8616809999999999	1.8311706999999999
H	1.6495200999999999	-5.0646192000000001	2.3213892999999999
H	1.2365116000000000	-5.4535346000000002	0.97111723000000005
O	-0.88035348000000002	-4.9638556999999999	0.25933728000000000
H	-0.10911619000000000	-4.3933745999999996	0.49935014999999999
H	-0.91079268000000002	-5.4980308000000004	1.1149585000000000
O	3.6908333000000000	-5.5074905999999997	3.7881098000000000
H	2.9969260000000002	-4.9084992999999999	3.5895201000000001
H	3.1550265000000000	-6.2419446000000001	3.9370459000000002
O	-0.74082630999999999	-4.1212701000000003	4.2631747000000004
H	0.19956845000000001	-4.2168767999999996	4.0625140999999996
H	-1.0917003999999999	-4.7248745000000003	3.5782728000000001
O	3.9059815000000002	-6.0202238000000001	0.43473516000000001
H	3.3473975999999999	-6.4448936999999997	-0.19954479999999999
H	3.2698711000000000	-5.7606178999999997	1.0902225000000001
O	3.4431764999999999	-3.6045484000000001	-1.3612932000000000
H	2.5248020000000002	-3.4494519000000001	-0.92781807999999999
H	3.9275788000000000	-2.9182974000000002	-0.82242430000000000
O	1.9447696999999999	-3.8967413000000000	6.3393465000000004
H	2.4068372000000000	-4.7114200999999998	6.0828739000000001
H	1.7134841000000001	-3.5258221999999999	5.4644002000000000
O	0.37157666000000000	-3.7213797999999998	-2.2177422999999998
H	0.36622230000000001	-4.7544741000000004	-2.2737044000000002
H	0.34634615000000002	-3.6539712000000000	-1.2755854000000000
O	4.8061233000000003	-2.1445565000000002	0.55340849000000003
H	5.1109210999999997	-2.9465555999999999	0.98216535000000005
H	5.3445689999999999	-1.5135395000000000	1.0157754000000001
O	-1.1088846999999999	-1.2644259000000000	4.4787140000000001
H	-1.0255451000000000	-2.1901080999999998	4.3677761999999998
H	-0.23086603000000000	-1.0261735000000001	4.7251199000000002
O	1.0635256000000000	-8.3704912000000000	2.7697299000000002
H	1.2575837000000001	-7.5656194000000001	2.2928041000000001
H	1.7947090999999999	-8.9012141000000007	2.5388787000000002
O	5.7497001000000001	-4.6208280000000004	1.9782154999999999
H	5.2646515000000003	-5.1377039000000000	1.2727917000000000
H	5.2422617999999996	-4.8805870999999996	2.7581663000000001

C	0.14610383E+01	-0.26553712E+01	0.26244650E+01
C	0.15889860E+01	-0.13016143E+01	0.31056854E+01
O	0.15486521E+01	-0.40122772E+00	0.20853242E+01
C	0.14374531E+01	-0.11019323E+01	0.84285034E+00
C	0.14223195E+01	-0.25654978E+01	0.12507241E+01
O	0.17091365E+01	-0.83202728E+00	0.42404217E+01
C	0.21237646E+00	-0.49227309E+00	0.97787947E-01
O	-0.92626479E+00	-0.65189569E+00	0.10103601E+01
O	1.4948672000000001	-3.4919593000000000	0.34601745000000000
O	1.4199079999999999	-3.7212466000000002	3.4527656000000002
C	0.50933823E+00	0.96415845E+00	-0.26753499E+00
O	-0.63409340E+00	0.17028875E+01	-0.61852238E+00
H	0.23402108E+01	-0.98361316E+00	0.29868787E+00
H	0.91330681E+00	0.14373662E+01	0.64525521E+00
H	0.13152890E+01	0.10685968E+01	-0.97365742E+00
H	-0.38757985E+00	0.26451123E+01	-0.50896012E+00
H	0.47485319E-01	-0.12112228E+01	-0.78767020E+00
H	-0.57599570E+00	-0.30943253E+00	0.18532443E+01
O	1.1908388000000001	-5.9640488999999999	1.8344038000000000
H	1.3617732000000000	-5.3339242000000002	2.5520187000000001
H	1.3594927999999999	-5.4038662999999998	1.0783107000000001
O	-0.92272695000000005	-4.8692565999999999	0.21218946000000000
H	-0.16628889999999999	-4.3040741999999996	0.31702750000000002
H	-1.0462970000000000	-5.2621623000000000	1.1219536999999999
O	-1.1501304000000001	-3.9934273000000000	4.0996686000000002
H	-0.23912617999999999	-3.8351187000000002	3.7681860999999999
H	-1.3359699000000000	-4.8515176000000002	3.6423071000000000
O	3.4674178000000002	-5.5911670999999998	3.5490244999999998
H	3.0164900000000001	-4.7098111999999999	3.5694341000000001
H	2.7556278000000001	-6.1621645999999997	3.2508610000000000
O	4.0458159000000000	-6.1634808000000003	0.26404807000000002
H	3.6024466999999998	-6.7339567999999996	-0.41203943999999998
H	3.3378169000000000	-5.5726336999999999	0.59240908000000003
O	3.5532357000000001	-3.9508728000000000	-1.3121848000000000
H	2.6861228000000001	-3.6590945000000001	-0.97141396999999996
H	4.2043585999999999	-3.5872060000000001	-0.59801652000000005
O	0.62490581000000001	-3.5506327999999998	-2.1971432000000002
H	0.63024665000000002	-4.5178818999999999	-2.3410418000000002
H	0.46845430999999998	-3.5339391999999998	-1.1402620000000001
O	1.8433714000000001	-3.8444900999999998	6.2303271999999996
H	2.3868089000000001	-4.6031744999999997	6.1529496000000004
H	1.6133071000000001	-3.7012130000000001	5.2693156999999999
O	4.8180228999999999	-2.1568361999999999	0.55787169999999997
H	4.9834268000000002	-2.9963685000000000	1.0606412000000001
H	5.3361694000000002	-1.5742946000000000	1.1997515999999999

O	-1.6089424999999999	-6.2579589999999996	2.5942096000000001
H	-0.71437890999999998	-6.5256432999999996	2.4787918000000002
H	-1.8226528000000000	-7.1266762000000003	3.0661432000000000
O	5.4058982000000002	-4.6187227000000002	2.0032906000000001
H	5.0576654999999997	-5.2318528999999998	1.3010596999999999
H	4.7950426000000004	-4.9623448000000003	2.7299834000000001
O	-1.2358042000000000	-1.1075290000000000	4.3231592000000001
H	-1.2814175999999999	-2.0760489000000000	4.4660848000000000
H	-0.37051399000000002	-0.88526174000000002	4.6847371000000004

C	0.12613630E+01	-0.26039101E+01	0.26093650E+01
C	0.17490404E+01	-0.13097458E+01	0.30673537E+01
O	0.17470223E+01	-0.42329695E+00	0.20149058E+01
C	0.13487315E+01	-0.11251392E+01	0.79755902E+00
C	0.11637263E+01	-0.25844286E+01	0.12248818E+01
O	0.21031040E+01	-0.92990773E+00	0.42114791E+01
C	0.46665523E-01	-0.47926017E+00	0.29716970E+00
O	-0.98132425E+00	-0.63709821E+00	0.12586721E+01
O	1.0582471000000000	-3.5348193999999999	0.37658863999999997
O	1.2543339000000000	-3.7210426000000001	3.3694378000000000
C	0.35227559E+00	0.10407467E+01	0.18311533E+00
O	-0.59175006E+00	0.16141324E+01	-0.77085616E+00
H	0.21160111E+01	-0.97472541E+00	0.11871260E+00
H	0.14831475E+00	0.14262739E+01	0.11993734E+01
H	0.13743515E+01	0.13001879E+01	-0.19637937E+00
H	-0.50996814E+00	0.25350262E+01	-0.58467725E+00
H	-0.15659124E+00	-0.92118763E+00	-0.69154447E+00
H	-0.65294249E+00	-0.10999690E+01	0.20788618E+01
O	1.3420433000000001	-6.0945422000000002	1.6699413999999999
H	1.4366882000000001	-5.2077555000000002	2.1607726999999999
H	1.2348832000000001	-5.6259180999999998	0.82574544999999999
O	-1.3152756999999999	-4.1025755000000004	4.0306837000000000
H	-0.36808213000000001	-3.9735122000000000	3.7941775999999998
H	-1.5388035000000000	-4.8553845999999998	3.4073327999999998
O	-1.3988588000000000	-4.9463619000000003	3.5046829000000002E-002
H	-0.64775108000000003	-4.4030848000000002	0.31129675000000001
H	-1.5491518000000000	-5.5757510000000003	0.75732748000000005
O	3.1810174000000000	-5.5670045999999997	3.9016251999999998
H	2.6357689000000000	-4.8096956999999998	3.7869785000000000
H	2.7539136000000002	-6.1967961000000003	3.3368424999999999
O	3.5936012000000002	-3.7899308999999999	-1.2643958000000000
H	2.8267850999999999	-4.0220026000000004	-0.71318404999999996
H	4.2401922000000001	-3.2619615000000000	-0.68028025999999997
O	0.38442051999999999	-3.6340829000000001	-2.2120185000000001
H	0.59308548000000005	-4.5124715999999996	-2.4111231000000002
H	0.67754433999999997	-3.5719261000000002	-1.2891383999999999
O	1.7976174000000000	-3.5079973000000000	6.1401263999999998
H	1.9861131000000001	-4.4719930999999997	6.0595740999999999
H	1.5418056000000000	-3.4260128000000001	5.1676146000000003
O	-1.4507848000000001	-6.5233223999999996	2.3970870999999998
H	-0.50800065999999999	-6.5727621999999997	2.6154394000000001
H	-1.7939309000000001	-7.3135317000000004	2.8361980999999998
O	4.0819013000000002	-6.4864806000000002	8.5165570999999995E-002
H	4.0950690999999999	-7.4176871000000002	-0.13346864999999999
H	3.1419470000000000	-6.3724246999999998	0.16047041000000001
O	5.2180264999999997	-4.9208436000000004	2.1521417000000000
H	4.7130235999999996	-5.4796116000000001	1.5348067999999999
H	4.6157678999999998	-4.9812564999999998	2.9777399999999998
O	-1.2640255000000000	-1.2878485000000000	4.4102132999999997
H	-1.5458997999999999	-2.1926657999999999	4.2694596000000002
H	-0.76985040000000005	-1.5049716000000000	5.2126557000000000
O	0.96361244000000001	-8.2187620999999993	3.2732640000000002
H	1.1165479000000000	-7.4440445000000004	2.7149676999999999
H	1.7580754000000001	-8.7531788000000006	3.0608591000000001

C	0.15197156E+01	-0.26590324E+01	0.25955082E+01
C	0.18771681E+01	-0.13342367E+01	0.30128506E+01
O	0.18560128E+01	-0.38342602E+00	0.19252912E+01
C	0.14268350E+01	-0.11370708E+01	0.78516222E+00
C	0.12504204E+01	-0.25663672E+01	0.11947485E+01
O	0.21015297E+01	-0.80087398E+00	0.41743350E+01
C	0.76780219E-01	-0.52427338E+00	0.33302543E+00
O	-0.90943467E+00	-0.71644022E+00	0.13956525E+01
O	0.71335680999999995	-3.3843974999999999	0.36800052000000000
O	1.4078291000000001	-3.6192793999999999	3.4206048000000000
C	0.27988078E+00	0.10016313E+01	0.11024099E+00
O	-0.77729902E+00	0.18045519E+01	-0.31852530E+00
H	0.21752954E+01	-0.91275822E+00	0.20688925E-01
H	0.73294932E+00	0.14310873E+01	0.10408199E+01
H	0.10661365E+01	0.11245754E+01	-0.64353169E+00
H	-0.13011541E+01	0.20565865E+01	0.44078917E+00
H	-0.28403026E+00	-0.11294447E+01	-0.51645042E+00
H	-0.55522583E+00	-0.59525020E+00	0.22864534E+01
O	1.2696386000000000	-5.8438651000000004	1.8213571000000000
H	1.3807421000000000	-4.9335022000000004	2.1727531000000000
H	0.98504164000000005	-5.5873660000000003	0.98051147000000005
O	-1.1626097000000000	-4.2405438000000002	4.4366855000000003
H	-0.33059548000000000	-4.2677611999999998	3.8731518999999999
H	-1.7096633999999999	-4.8304713000000001	3.8985489000000002
O	-1.3516827000000000	-4.9461592000000003	-0.20255139999999999
H	-0.68867480000000003	-4.3422460999999997	0.16816536000000001
H	-1.3888218000000001	-5.4950862999999996	0.59917684999999998
O	3.2247145000000002	-5.6636053000000004	3.9748456000000001
H	2.5471409000000000	-4.9770352000000004	3.7640908999999998
H	3.2116821000000000	-6.2645001000000002	3.1700059999999999
O	0.75475312999999999	-3.5580438000000001	-2.2167007000000001
H	0.62115518000000003	-4.4873045999999999	-2.1707717999999998
H	0.62670490000000001	-3.3304260999999999	-1.2732441000000001
O	1.3482263999999999	-3.7110012999999999	6.1681489000000003
H	1.3238448000000000	-4.6223656999999996	6.3527325000000001
H	1.5632012000000000	-3.6449455999999998	5.2352654999999997
O	-1.5217814000000001	-6.2855477000000004	2.4694316000000001
H	-0.61639104000000000	-6.5617938999999996	2.5113774000000002
H	-1.9607532000000001	-7.0652188999999996	2.8845166000000000
O	3.6014607000000001	-3.5027370000000002	-1.6726057999999999
H	2.7808432000000001	-3.4984784000000002	-1.2432741000000000
H	4.1981811000000002	-3.0170802000000001	-1.0481412000000001
O	-1.1916281000000000	-1.3918592000000001	4.5378901999999997
H	-1.1541197000000001	-2.3284541999999999	4.4921265999999997
H	-0.27482273000000002	-1.2083298000000000	4.8754803000000004
O	3.8337520000000000	-6.5788751999999997	0.63345465000000001
H	3.7572505999999999	-7.3032845000000002	1.8064212000000000E-002
H	2.9134848000000000	-6.4401035000000002	0.83412790000000003
O	-3.2490464000000001	-2.5231283000000002	0.54527731999999995
H	-2.9130346999999999	-3.4609182000000001	0.65014828000000002
H	-2.4115712999999999	-2.0590820999999999	0.61199135999999998
O	1.1779044000000001	-8.2066499000000004	3.2489816999999999
H	1.3321954000000000	-7.4825825999999998	2.6507399000000000
H	1.9663845000000000	-8.7911145000000008	3.2143058000000000

C	0.13886724E+01	-0.27135741E+01	0.26316515E+01
C	0.17366044E+01	-0.13984677E+01	0.29761915E+01
O	0.17301576E+01	-0.49041809E+00	0.18939003E+01
C	0.12189317E+01	-0.12202131E+01	0.74371255E+00
C	0.11765098E+01	-0.26777111E+01	0.12239673E+01
O	0.19693629E+01	-0.90377187E+00	0.41154284E+01
C	-0.80770601E-01	-0.50258982E+00	0.37566291E+00
O	-0.11008893E+01	-0.83651480E+00	0.13744423E+01
O	1.0504073000000000	-3.6728209999999999	0.35267190999999998
O	1.3645569000000000	-3.7467684000000001	3.4940842999999999
C	0.70958078E-01	0.10175324E+01	0.26233139E+00
O	-0.95126391E+00	0.17760302E+01	-0.35295327E+00
H	0.19886651E+01	-0.96992358E+00	-0.17795624E-01
H	0.27207656E+00	0.14477384E+01	0.12653637E+01
H	0.91452322E+00	0.10847529E+01	-0.53264256E+00
H	-0.14917814E+01	0.21263549E+01	0.38973022E+00
H	-0.41637769E+00	-0.98327909E+00	-0.56882684E+00
H	-0.63824457E+00	-0.11660503E+01	0.21843428E+01
O	1.3235979000000000	-5.9766909000000004	2.0336048000000000
H	1.2708756999999999	-5.1579364999999999	2.5891663999999999
H	1.1833501000000000	-5.5069737999999999	1.2196397000000001
O	-1.5032626000000000	-4.6441467000000003	0.37643258000000002
H	-0.56642808000000000	-4.3511245000000001	0.29709723999999998
H	-1.5653125999999999	-5.0925324999999999	1.2729547000000001
O	-1.1593789000000001	-4.1560446000000004	4.4721102999999998
H	-0.19031824000000000	-4.1728332999999997	4.3007261999999997
H	-1.5135677000000001	-4.7358038000000002	3.8235364000000001
O	3.4172699999999998	-5.5342796999999999	4.1125325000000004
H	2.6705893000000001	-4.8777188999999996	4.0152039999999998
H	3.0580318000000002	-6.0891766000000001	3.4208542999999998
O	-1.5877545000000000	-6.0905335000000003	2.7547465000000000
H	-0.69768353999999999	-6.2631673000000001	2.4114545999999999

H	-1.9759407000000000	-6.9287368999999996	2.9367383999999999
O	0.64482952000000004	-3.5023721000000001	-2.3131718999999999
H	0.39458965000000001	-4.4256456000000002	-2.3516571000000002
H	0.92582907000000003	-3.3763725999999998	-1.3658600000000001
O	1.5330245000000000	-3.7409783000000001	6.1955666999999996
H	1.1680904000000001	-4.6507573999999998	6.2148059000000000
H	1.3346979000000001	-3.5094767000000000	5.2521328000000000
O	3.9253342999999998	-3.8523204000000000	-1.5601209000000000
H	3.1902689999999998	-3.6736276000000001	-0.99832056000000002
H	4.5955092999999998	-3.2863367000000001	-1.1321471999999999
O	-0.96089928000000002	-1.2999738999999999	4.5794493000000003
H	-1.0666043000000001	-2.2860117000000000	4.6164345999999998
H	3.5281541999999999E-002	-1.3435261999999999	4.8381543999999996
O	4.2315166000000000	-6.2819906999999997	0.10432892000000001
H	3.8746349000000002	-7.1468930999999998	0.19721818999999999
H	3.6088472999999999	-5.7095371999999998	-0.30983685000000000
O	4.9410594999999997	-2.0971848999999998	0.60769951000000000
H	4.9094065000000002	-2.8440078999999998	1.2343805999999999
H	5.3954101000000003	-1.3765509000000000	1.1755644000000001
O	5.1324715999999997	-4.4657463000000002	1.9442086999999999
H	4.7723364000000004	-5.1732468000000003	1.3765764000000000
H	4.9009362999999997	-4.7965064999999996	2.8069270999999998

C	0.12018723E+01	-0.27028177E+01	0.27700512E+01
C	0.15340874E+01	-0.13229461E+01	0.31183645E+01
O	0.16035483E+01	-0.45706122E+00	0.20256751E+01
C	0.13588297E+01	-0.12222267E+01	0.85286540E+00
C	0.12637269E+01	-0.26861965E+01	0.13621016E+01
O	0.17330945E+01	-0.86868883E+00	0.42531601E+01
C	0.30604097E-01	-0.67212110E+00	0.22188273E+00
O	-0.10689745E+01	-0.86824529E+00	0.11278156E+01
O	1.2206527000000000	-3.6246784000000001	0.52807327999999998
O	1.3763631000000001	-3.7133908000000000	3.6171069000000000
C	0.21651305E+00	0.87085077E+00	0.15263591E-01
O	-0.10352825E+01	0.15169299E+01	-0.15094866E+00
H	0.22309361E+01	-0.10851866E+01	0.14627787E+00
H	0.63876142E+00	0.12447986E+01	0.95968018E+00
H	0.88431849E+00	0.10755563E+01	-0.79388348E+00
H	-0.14642394E+01	0.14012463E+01	0.72563717E+00
H	-0.12997316E+00	-0.11772881E+01	-0.79835813E+00
H	-0.68154855E+00	-0.12492987E+01	0.19221258E+01
O	1.3198251999999999	-6.0366647999999996	2.1044972999999998
H	1.3272594000000000	-5.2276682000000001	2.7099826999999999
H	1.4615578000000000	-5.4725276999999997	1.2999118000000001
O	-1.4987136000000001	-4.2780239000000000	0.59372979000000004
H	-0.49788947000000000	-4.3426055999999997	0.64525220999999999
H	-1.7742359000000001	-5.0573980000000001	1.1044537999999999
O	-1.2387576000000000	-4.1309448000000000	4.2902522999999997
H	-0.36685181000000000	-3.8192170000000001	3.9803175000000001
H	-1.3072078000000000	-4.9004181000000004	3.7043862999999999
O	3.4975070000000001	-5.4266323999999999	3.8454996000000001
H	2.9833319000000000	-4.6340880999999996	3.9542630000000001
H	2.9262288999999999	-5.9469430000000001	3.2680655999999999
O	3.7184746999999998	-3.8943550999999998	-1.0326118000000000
H	2.7755478999999998	-3.8836032000000000	-0.70909409999999995
H	4.0901947999999999	-3.1750633000000001	-0.53491047999999997
O	0.99395120000000003	-3.3407510000000000	-2.3260602000000001
H	0.59481119999999998	-4.1442509999999997	-2.7763575000000000
H	1.0063248000000000	-3.4973898000000001	-1.3325231000000000
O	1.4141741000000001	-3.9723785999999999	6.4776233999999997
H	1.1163673000000001	-4.8862668999999999	6.6557418999999998
H	1.2834166000000000	-3.9566948000000002	5.4982479000000000
O	-1.3589792000000001	-1.5185998000000001	4.1286582000000003
H	-1.4228626000000000	-2.4812090000000002	4.0781055999999998
H	-0.52636897000000005	-1.4824406000000000	4.5909928999999998
O	4.1927975999999996	-6.2483646999999998	0.44175714999999999
H	3.5537603999999998	-7.0235890999999997	0.53444773000000001
H	3.6218156000000001	-5.5652381000000002	2.5393730999999999E-002
O	-1.5213563999999999	-6.4671605999999997	2.5504768000000002
H	-0.63504415999999997	-6.7752496999999998	2.4210802000000000
H	-1.8747562000000000	-7.1635232999999996	3.1660634999999999
O	5.4542707999999998	-4.3944539999999996	2.3160687000000002
H	5.4470251000000003	-5.0146446999999998	1.5579608000000000
H	4.7702011999999998	-4.8464084999999999	2.8699981000000001
O	1.0556057000000001	-8.5144359999999999	3.0608200999999999
H	1.1550252000000001	-7.5570325000000000	2.8968162999999998

H	1.9569302000000000	-8.8994312000000004	2.8627292999999998

C	0.16666564E+01	-0.26513497E+01	0.27121938E+01
C	0.17749742E+01	-0.13376307E+01	0.31531883E+01
O	0.18762317E+01	-0.43988274E+00	0.20862885E+01
C	0.16742683E+01	-0.11513200E+01	0.83581494E+00
C	0.15734573E+01	-0.26067304E+01	0.12813091E+01
O	0.17874210E+01	-0.92733780E+00	0.43401813E+01
C	0.32944260E+00	-0.61559478E+00	0.20575346E+00
O	-0.76903099E+00	-0.95713592E+00	0.11186037E+01
O	1.3434044000000001	-3.6193548000000000	0.50724234000000001
O	1.3918159000000001	-3.7139723999999998	3.4878567999999999
C	0.40587155E+00	0.85844793E+00	0.29293021E-01
O	-0.94134679E+00	0.14191742E+01	-0.37750676E+00
H	0.25408874E+01	-0.10722191E+01	0.21512708E+00
H	0.70831370E+00	0.14110067E+01	0.98776225E+00
H	0.10493699E+01	0.11034120E+01	-0.77464864E+00
H	-0.14278317E+01	0.15987537E+01	0.44783489E+00
H	0.22811986E+00	-0.11895843E+01	-0.75572555E+00
H	-0.38110445E+00	-0.15697274E+01	0.17683542E+01
O	1.5549459999999999	-6.0222651000000003	1.8789496000000001
H	1.1017425999999999	-5.3587799000000000	2.4387967000000002
H	1.8662685999999999	-5.3935849999999999	1.2737961000000000
O	-1.3294888000000000	-4.4545192000000000	0.67411695999999999
H	-0.35603750000000001	-4.1761528999999999	0.64721424999999999
H	-1.3049352000000001	-5.3144453000000000	1.0730010000000001
O	-1.2514611000000000	-4.3265345000000002	4.4296167999999998
H	-0.39155266000000000	-3.9908937999999998	4.0798411000000003
H	-1.3975611999999999	-5.0756769999999998	3.7907593999999998
O	3.4735073999999999	-5.5477483000000003	3.7475298000000001
H	2.8566951000000000	-4.8632476000000002	4.0294160999999997
H	2.9737182999999998	-5.8578424000000000	2.9920008999999999
O	1.6810052000000000	-4.1899329999999999	6.1755250000000004
H	1.2060268999999999	-4.9804566000000001	6.2799218000000003
H	1.6429147000000000	-4.0041190999999996	5.1842354000000004
O	3.7797485000000002	-3.4014353000000002	-1.0969807000000000
H	2.9169925999999999	-3.6405297999999999	-0.85920331999999999
H	4.2243943000000002	-3.1038277000000001	-0.26454349999999999
O	0.72178096999999997	-3.4840216000000002	-2.3313942000000001
H	0.69977305000000001	-4.4038775000000001	-2.6094390999999999
H	0.76570855000000004	-3.4737930000000001	-1.3331987000000001
O	-1.2212193000000000	-1.5942016999999999	4.2755247000000001
H	-1.4921331000000000	-2.5617683000000002	4.3418128999999999
H	-0.29097239000000003	-1.6384946000000000	4.4127150999999998
O	2.0714128000000001	-6.4814388000000003	-1.0115759000000000
H	1.8260400000000001	-7.4532636999999999	-0.93241244999999995
H	1.2316494000000000	-6.1210418999999998	-0.68071102000000006
O	5.4888678999999998	-4.5299686000000001	2.4443454999999998
H	5.7581464000000002	-5.2435425000000002	1.8233055000000000
H	4.8108127999999999	-4.9787431000000000	3.0182625000000001
O	1.1348868000000001	-8.2740687000000008	3.2363547000000001
H	1.3634721999999999	-7.5206460999999996	2.6037121000000001
H	1.9917507000000001	-8.7535878000000000	3.3907661000000000
O	-1.1520714999999999	-6.6124415000000001	2.7367273999999999
H	-0.34054705000000002	-7.1399596000000001	2.9201310000000000
H	-1.8185121000000000	-7.1747155999999999	3.2042242000000001

C	0.15110328E+01	-0.27867062E+01	0.24547457E+01
C	0.18417033E+01	-0.14646509E+01	0.29235541E+01
O	0.19512783E+01	-0.55279290E+00	0.19417986E+01
C	0.16548828E+01	-0.12277634E+01	0.69276432E+00
C	0.16195127E+01	-0.26909196E+01	0.10488402E+01
O	0.20638210E+01	-0.11283635E+01	0.41111476E+01
C	0.29298854E+00	-0.82817301E+00	0.12316357E+00
O	-0.80241325E+00	-0.96179254E+00	0.10157471E+01
O	1.4738083000000000	-3.6833179000000000	0.23357668000000001
O	1.3956195000000000	-3.8396756000000001	3.1649756999999998
C	0.32179459E+00	0.65112765E+00	-0.28589350E+00
O	-0.10383282E+01	0.11472787E+01	-0.48367240E+00
H	0.24163794E+01	-0.94110463E+00	-0.11429844E+00
H	0.80176340E+00	0.12252238E+01	0.50684327E+00
H	0.84197937E+00	0.81105124E+00	-0.12098770E+01
H	-0.13970939E+01	0.12314605E+01	0.37237569E+00
H	0.17329360E+00	-0.15031571E+01	-0.73126090E+00
H	-0.66701595E+00	-0.17285266E+01	0.15805069E+01
O	1.4074534999999999	-6.1129270000000000	1.5838649000000000

H	1.4673989000000001	-5.2064589000000003	1.9659076000000000
H	1.5196722000000000	-5.9741280999999997	0.67661033000000004
O	-1.3279491999999999	-4.6362224999999997	0.52012073000000003
H	-0.53348092999999996	-4.1568252000000001	0.20701259000000000
H	-0.99161478999999997	-5.0465358000000000	1.3821243999999999
O	3.4435207000000001	-5.6482011999999999	3.6427577000000002
H	2.8665246000000000	-4.8227028000000001	3.6429643000000000
H	2.9469348000000002	-6.1178347999999998	2.9616022000000002
O	-1.3247201000000000	-4.1739256999999998	3.9376348999999999
H	-0.37279827999999998	-4.0496004000000001	3.7205317999999998
H	-1.6046537999999999	-5.0596331000000001	3.5980297999999999
O	3.8950019000000000	-3.2597824000000002	-1.2348661999999999
H	2.9992835000000002	-3.4165353999999999	-0.85948110000000000
H	4.3547970999999999	-2.8674909999999998	-0.47188693999999998
O	0.79067038999999995	-3.2981934000000002	-2.5357710000000000
H	1.0567375000000001	-4.1077896999999997	-3.0175079000000000
H	0.86588262999999999	-3.6217088999999998	-1.6159185000000000
O	1.2347002000000000	-3.9949088000000001	6.2158796000000001
H	0.84603839999999997	-4.8312207999999996	6.2704715000000002
H	1.3569624000000000	-3.8981636000000002	5.2352055999999996
O	4.3454753999999998	-6.4108377000000001	0.94592197000000000
H	4.2004511999999998	-7.3162121999999998	1.1444794000000000
H	3.6215155000000001	-6.2838015000000000	0.26091315999999998
O	1.3035862000000000	-8.2088322999999992	3.2822648999999999
H	1.3571504000000001	-7.2996933999999998	2.8124055000000001
H	2.1723878999999999	-8.6221285000000005	3.1739766999999999
O	-0.92394630000000000	-1.5055649000000000	4.2440195999999997
H	-1.1488486000000000	-2.4497933000000001	4.1388655999999999
H	-1.1211000000000000E-002	-1.5275557000000000	4.4822407999999996
O	5.8098856000000003	-4.5090785000000002	2.5607997000000000
H	5.3574913999999998	-5.0327925999999996	1.8331229000000000
H	5.4760162000000001	-4.9609293000000001	3.3777257999999999
O	3.4562206000000000	-2.3932992000000000	6.2041776999999998
H	2.8321934999999998	-2.1687077000000001	5.5011013000000002
H	3.1169923000000002	-3.2186381000000002	6.6219796999999998

C	0.14902415E+01	-0.28378975E+01	0.24159925E+01
C	0.19724024E+01	-0.15791860E+01	0.28144617E+01
O	0.21284825E+01	-0.65897977E+00	0.17517874E+01
C	0.16431497E+01	-0.13745512E+01	0.59427060E+00
C	0.15278735E+01	-0.28219614E+01	0.99894820E+00
O	0.20922067E+01	-0.11768886E+01	0.39914516E+01
C	0.29010940E+00	-0.84882254E+00	0.12633735E+00
O	-0.71311275E+00	-0.95174405E+00	0.11234660E+01
O	1.3499378000000000	-3.6915836999999998	7.9115268000000002E-002
O	1.4018257999999999	-3.8932541000000001	3.2175669999999998
C	0.48894645E+00	0.64257892E+00	-0.50107948E-01
O	-0.71481535E+00	0.12048877E+01	-0.60175127E+00
H	0.23592309E+01	-0.11765464E+01	-0.20339491E+00
H	0.78245644E+00	0.10390014E+01	0.92395794E+00
H	0.13663404E+01	0.86885839E+00	-0.69547553E+00
H	-0.94509103E+00	0.18944518E+01	0.35598234E-01
H	0.33180623E-01	-0.13590130E+01	-0.82281986E+00
H	-0.57225183E+00	-0.17870265E+01	0.16401117E+01
O	1.2910385000000000	-6.1932710000000002	1.6537135000000001
H	1.2745329999999999	-5.2060674000000002	1.8653470999999999
H	1.3508138000000001	-6.1574656000000001	0.73154514999999998
O	-1.3611508000000001	-4.3765844999999999	0.38029759000000002
H	-0.38579988999999998	-4.2784304000000004	0.26235689000000001
H	-1.5527016000000000	-5.0766719000000000	1.0566153000000000
O	-1.1200057999999999	-4.4743696000000002	3.8605706000000000
H	-0.16890383000000000	-4.4879683999999997	3.5336560000000001
H	-1.4617321000000001	-5.1576440999999997	3.2422304999999998
O	3.5508261999999999	-5.5590586000000002	3.5460813000000000
H	2.9996548000000001	-4.7863914000000003	3.4239149000000002
H	3.3240392999999999	-5.9980117000000002	2.7212318000000000
O	4.0131138000000002	-3.4258228000000002	-1.3421863999999999
H	3.1440378000000000	-3.6048060999999998	-0.81894142999999997
H	4.5195676000000002	-2.9388803000000001	-0.63346493999999998
O	0.89303105000000005	-3.4039402999999999	-2.5772780000000002
H	0.91746316999999999	-4.3679534999999996	-2.7387489000000000
H	1.1288175000000000	-3.4194054000000000	-1.6024548000000001
O	-1.1004973000000000	-1.6572150999999999	4.3146806000000000
H	-1.3903269000000000	-2.6186596000000000	4.2849570999999997
H	-0.11885724000000000	-1.8192058000000000	4.3678150000000002
O	1.7162621000000000	-4.1856229999999996	6.2764157000000003
H	1.6214449000000000	-5.1321988000000003	6.3834125999999998
H	1.8081921000000001	-3.9280309999999998	5.3153252000000002
O	1.1674344999999999	-8.2263453999999996	3.2176265000000002
H	1.3071062000000000	-7.4206424999999996	2.7152788999999999
H	1.9794200000000000	-8.7441896999999997	3.0499348999999998
O	-2.9453035999999999	-2.0276839999999998	0.36004493999999999
H	-2.3424372000000000	-2.8452500000000001	0.26854832000000001
H	-2.2581422999999998	-1.3257886000000001	0.60676127000000002
O	4.2149729999999996	-6.9348972000000000	0.95727364000000004
H	4.2039444000000001	-7.7919755999999998	1.3749243000000000
H	3.4965041000000001	-7.0763919000000000	0.19146431000000000
O	5.8057964000000002	-4.7640361999999996	2.4278179999999998
H	5.5790496000000003	-5.4480208000000001	1.7634344000000000
H	5.0760788000000003	-5.0756714000000001	3.0871368000000001

C	0.17806139E+01	-0.28677533E+01	0.24763352E+01
C	0.20075049E+01	-0.15147588E+01	0.28999208E+01
O	0.21319307E+01	-0.64986462E+00	0.18076622E+01
C	0.16721675E+01	-0.13516335E+01	0.66914787E+00
C	0.16779187E+01	-0.28118621E+01	0.10765811E+01
O	0.21803123E+01	-0.97447249E+00	0.39838617E+01
C	0.34833733E+00	-0.71684165E+00	0.25343416E+00
O	-0.56965177E+00	-0.81947355E+00	0.13288251E+01
O	1.3913221000000000	-3.7337948999999999	0.23502991000000001
O	1.6131603000000001	-3.9048449999999999	3.3602097000000000
C	0.53198310E+00	0.73862440E+00	-0.11462869E+00
O	-0.70046386E+00	0.13355783E+01	-0.61631342E+00
H	0.23746991E+01	-0.12182113E+01	-0.14655920E+00
H	0.96229948E+00	0.13683868E+01	0.73472399E+00
H	0.12154028E+01	0.73269045E+00	-0.97798018E+00
H	-0.95004375E+00	0.20503918E+01	-0.71943082E-01
H	-0.25877840E-01	-0.12343552E+01	-0.66588084E+00
H	-0.43565089E+00	-0.16162233E+01	0.18684971E+01
O	1.5583290000000001	-6.1126908999999996	1.7456198999999999
H	1.2457898999999999	-5.5355699999999999	2.4413507000000001
H	1.6154520000000001	-5.5382645999999998	0.94460078999999997
O	-1.1677512999999999	-4.2272808999999996	0.80947977999999998
H	-0.33128559000000002	-4.2322297000000004	0.36148849999999999
H	-1.0966908000000000	-4.9974093000000002	1.3832622999999999
O	3.7151162000000002	-5.4950340999999998	3.5206423000000000
H	3.0033004000000001	-4.8027031999999998	3.4799147000000001
H	3.2282924999999998	-6.1965235999999999	2.9960567000000000
O	-1.0328997000000000	-4.4174220000000002	4.0074348000000004
H	-0.22209914000000000	-4.1347287000000001	3.5493302999999998
H	-1.2150988000000000	-5.1533104999999999	3.4884805999999999
O	3.9390703000000000	-3.4266261999999998	-1.2358864000000001
H	3.1877922999999999	-3.4990250999999999	-0.59309997000000003
H	4.5348344000000003	-2.8585202000000001	-0.75418152999999999
O	0.88396375000000005	-3.1574639000000002	-2.4751280000000002
H	0.89074399999999998	-4.0459734999999997	-2.8358945000000002
H	1.0413144999999999	-3.3805380000000000	-1.5368037000000001
O	1.6253084000000000	-3.9081362999999998	6.2695505999999996
H	1.2700157999999999	-4.7917075000000002	6.3929942000000004
H	1.5073114000000001	-3.8224885999999998	5.2559174999999998
O	4.4457646999999998	-6.8973030000000000	0.61098193000000001
H	4.3648766999999999	-7.8337272999999996	0.62176472999999999
H	3.5690390999999999	-6.6582200000000000	0.30439372999999997
O	5.7987140000000004	-4.6940599000000001	2.1168493000000002
H	5.5772880999999996	-5.3542161000000004	1.3718773000000000
H	5.0818485999999998	-4.9108688000000003	2.7354609000000001
O	1.2590444999999999	-8.5098936999999992	3.1524692999999999
H	1.3537459000000001	-7.8593726999999998	2.4396106000000000
H	2.1909201999999999	-8.7927292999999995	3.2816041000000000
O	-1.0141658000000000	-1.5141697000000001	4.7375730999999996
H	-1.0892137000000000	-2.4577379000000001	4.6703153999999998
H	-7.6297572999999994E-002	-1.4504246000000001	4.9971594000000001
O	-1.4353248999999999	-6.7068830999999998	2.4130668000000002
H	-1.1646152999999999	-7.2643468999999996	1.7008198000000001
H	-1.7616107999999999	-7.3578333000000002	3.0955482999999999

C	0.17499041E+01	-0.28835518E+01	0.25968427E+01
C	0.19026990E+01	-0.15157045E+01	0.31019972E+01
O	0.19587997E+01	-0.56899690E+00	0.20897162E+01
C	0.16807555E+01	-0.13615854E+01	0.78875384E+00
C	0.17613150E+01	-0.27843432E+01	0.11862112E+01
O	0.19369178E+01	-0.10901610E+01	0.42853442E+01

C	0.35256232E+00	-0.87414734E+00	0.21726297E+00
O	-0.59840770E+00	-0.88481182E+00	0.12813742E+01
O	1.5281545999999999	-3.6908960000000000	0.30773630000000002
O	1.5979345000000000	-3.9578924999999998	3.3127143000000001
C	0.54682435E+00	0.55901242E+00	-0.29117606E+00
O	-0.79881918E+00	0.11404188E+01	-0.57202345E+00
H	0.25102915E+01	-0.10507453E+01	0.12908948E+00
H	0.94399168E+00	0.11687869E+01	0.47576759E+00
H	0.12074402E+01	0.58878903E+00	-0.12286408E+01
H	-0.10141069E+01	0.18231834E+01	0.82892096E-01
H	-0.77699708E-01	-0.15223936E+01	-0.55518721E+00
H	-0.37520028E+00	-0.16554678E+01	0.18824124E+01
O	1.2928002999999999	-6.1214743000000000	1.6552572000000001
H	1.3496638000000001	-5.4125135999999996	2.3299775999999999
H	1.5414089000000000	-5.6000871999999999	0.81727901000000003
O	-1.1838256000000000	-4.1819965999999997	0.68833074000000005
H	-0.23887586999999999	-4.0025798000000004	0.73759085999999996
H	-1.2973110000000001	-4.8215189000000001	1.4195142999999999
O	3.4189140999999998	-5.8754356000000003	3.5410355999999998
H	2.7241038999999998	-5.1826141000000003	3.5130669000000001
H	3.2349019000000001	-6.1649696000000000	2.6009050000000000
O	-0.86294501000000001	-4.5404021999999999	4.2204984999999997
H	9.7069226999999994E-002	-4.5276114999999999	4.0783680000000002
H	-1.3428097000000001	-5.1294908000000001	3.5682380999999999
O	3.9040431000000000	-3.6736650000000002	-1.2684816000000001
H	3.0909018000000001	-3.6103740000000002	-0.77496874000000004
H	4.5780731000000001	-3.3146958999999998	-0.67137959999999997
O	0.72457645000000004	-3.2390574000000001	-2.3875378000000000
H	0.85518388999999995	-4.1349267999999997	-2.6640682000000000
H	0.92607724999999996	-3.3503986000000001	-1.4323128000000001
O	-1.4364497000000001	-6.3962332999999996	2.3632314000000001
H	-0.51238238999999997	-6.5235820999999996	2.0642990000000001
H	-1.5702734000000000	-7.3164911000000004	2.6907827000000002
O	-1.0576421000000000	-1.5848154999999999	4.4382557000000000
H	-0.81527446999999997	-2.5199227000000000	4.1494074999999997
H	-0.30513688999999999	-1.4296039000000000	5.0030104000000000
O	4.5368642000000001	-6.7191463999999996	0.76702349000000003
H	4.5403599999999997	-7.5546810999999998	0.28174935000000001
H	3.7785627000000002	-6.2751805000000003	0.28384547999999998
O	1.7931250999999999	-4.1132080999999996	6.4513059000000004
H	1.5597555999999999	-5.0354473999999998	6.4658293000000002
H	1.4761009000000000	-3.7935330999999999	5.5908085999999999
O	1.2946000000000000	-8.5575462000000009	3.2993187000000002
H	1.6247638000000000	-7.7205775000000001	3.0780989999999999
H	2.1129999000000002	-9.0862216000000000	3.2364552000000000
O	5.9292543000000002	-4.7418836000000004	2.1681572999999998
H	5.3321731000000003	-5.4666123000000004	1.7782498000000000
H	5.5925946000000000	-4.6367544000000001	3.0765680999999998

C	0.17377136E+01	-0.27984279E+01	0.25997705E+01
C	0.18527159E+01	-0.15209778E+01	0.31342084E+01
O	0.21052264E+01	-0.56318635E+00	0.20841367E+01
C	0.18006529E+01	-0.11783314E+01	0.82438271E+00
C	0.17323505E+01	-0.26638693E+01	0.12169668E+01
O	0.20264292E+01	-0.11588882E+01	0.43016431E+01
C	0.46098951E+00	-0.71922112E+00	0.34106958E+00
O	-0.53949663E+00	-0.94651588E+00	0.13139809E+01
O	1.6570092000000001	-3.5684133999999998	0.30280392000000000
O	1.5707078999999999	-3.9132939000000002	3.3615773999999998
C	0.43560536E+00	0.80762877E+00	-0.65769782E-01
O	-0.86776904E+00	0.12981598E+01	-0.58976917E+00
H	0.26984085E+01	-0.94888066E+00	0.15512941E+00
H	0.78517364E+00	0.15304649E+01	0.70131395E+00
H	0.11439848E+01	0.80795461E+00	-0.89732797E+00
H	-0.13585874E+01	0.16660523E+01	0.21244548E+00
H	0.25061905E+00	-0.12739526E+01	-0.58976783E+00
H	-0.49117161E+00	-0.19148207E+01	0.16172013E+01
O	1.2502192000000001	-5.9416121000000004	1.6088967999999999
H	1.3002602000000001	-5.2928892000000003	2.3623036000000002
H	1.5302632000000000	-5.3364883000000001	0.93108732999999999
O	-1.0266137000000000	-4.2487138000000000	0.36038619000000000
H	-8.5975222000000004E-002	-4.1299174000000001	0.38745635000000000
H	-1.1936214999999999	-4.9368753999999999	1.0118670999999999
O	3.6245664000000000	-5.7049225000000003	3.3768052000000002
H	2.9661141000000000	-4.9714102999999996	3.5152366000000002
H	3.3060279000000001	-6.0132433000000001	2.4793329000000002
O	-0.92348584000000000	-4.4349059999999998	4.1861284999999997
H	-2.3760284999999999E-002	-4.3179430999999999	3.7678856999999999
H	-1.3074977999999999	-5.1660057999999998	3.6900667000000000
O	3.7889438000000002	-3.9092600000000002	-1.3672461000000000
H	3.0156849999999999	-4.0536707999999999	-0.82382849000000002
H	4.1921732000000000	-3.1511868000000001	-0.84893258000000005
O	0.62525222999999996	-3.2291365000000001	-2.2000538000000001
H	0.98193368000000003	-4.0669408999999996	-2.5587149000000000
H	0.79195543999999995	-3.2873720000000000	-1.2208741000000001
O	1.6227475000000000	-4.0886882000000000	6.3678704000000002
H	2.2133997000000001	-4.8433124000000003	6.4506530000000000
H	1.4763953999999999	-4.0034809999999998	5.3484540000000003
O	5.8147789000000003	-4.7489170999999999	2.1211232000000000
H	5.6368638000000004	-5.2903668000000001	1.3455804000000000
H	5.0561487999999999	-4.9507108000000004	2.6539804999999999
O	-1.5514332000000000	-6.2957112999999998	2.1233765000000000
H	-0.73859258999999999	-6.6889593999999999	1.8259296000000000
H	-1.8334569000000001	-6.9807003999999999	2.7404177000000001
O	5.3286255999999996	-2.2912908000000001	0.54308840999999997
H	5.3839180999999998	-3.1207006000000002	1.0582026000000000
H	5.8045910999999997	-1.6183839000000000	1.1110454000000001
O	4.3835968000000003	-6.8010897999999997	0.55119313000000003
H	4.4246240999999999	-7.7286112999999999	0.39301593000000001
H	3.6341584999999998	-6.5523974000000003	-3.8919837999999998E-002
O	-0.94945416999999999	-1.5016791000000000	4.6750930999999998
H	-1.1460106000000001	-2.4964944999999998	4.6811999000000002
H	1.7782553000000000E-002	-1.6221563000000001	4.7706106999999998

C	0.18737193E+01	-0.28953451E+01	0.26294164E+01
C	0.18944722E+01	-0.15253158E+01	0.31014707E+01
O	0.20758541E+01	-0.64521236E+00	0.20346482E+01
C	0.18722828E+01	-0.13308195E+01	0.85063688E+00
C	0.18165511E+01	-0.27767184E+01	0.12284290E+01
O	0.17581777E+01	-0.10349883E+01	0.42360663E+01
C	0.58842928E+00	-0.76288744E+00	0.13910617E+00
O	-0.56464126E+00	-0.88651921E+00	0.10602894E+01
O	1.5676691000000000	-3.7629418000000001	0.38046261999999997
O	1.5839483000000001	-3.9289646999999999	3.4305303000000000
C	0.69037918E+00	0.72063714E+00	0.20230346E-01
O	-0.47234296E+00	0.14095365E+01	-0.40607288E+00
H	0.26969384E+01	-0.10161139E+01	0.13126222E+00
H	0.89280432E+00	0.10752304E+01	0.10457807E+01
H	0.14730470E+01	0.98745526E+00	-0.65360711E+00
H	-0.95922307E+00	0.14098743E+01	0.44311287E+00
H	0.43838215E+00	-0.12396623E+01	-0.82436661E+00
H	-0.45517005E+00	-0.15702556E+01	0.17513884E+01
O	1.1107402000000000	-6.1204891999999997	1.6577827000000001
H	1.2959390000000000	-5.2659564999999997	2.1438131000000000
H	1.4243043000000000	-5.7532595999999998	0.80461510999999997
O	-1.0674482999999999	-4.4603732000000003	0.15956276999999999
H	-0.15345887999999999	-4.1965339000000004	0.34880843000000000
H	-1.3068036000000001	-4.9780119000000003	1.0018894000000000
O	3.6964185999999999	-5.6564728999999998	3.5223176000000000
H	3.0144318999999999	-4.9137114000000004	3.5364822999999999
H	3.3065058000000001	-6.0743233999999999	2.7128106000000001
O	-1.0343058000000001	-4.2761193000000004	4.0015754000000001
H	-7.2831477000000006E-002	-4.0540263000000003	3.8742679999999998
H	-1.2026211000000000	-5.1224572999999998	3.5379410000000000
O	3.6761626999999999	-3.6460655000000002	-1.4545078000000000
H	2.8515628000000000	-3.6633439999999999	-0.91336402999999999
H	4.3817462000000003	-3.3514859000000001	-0.91133916999999998
O	0.95962417000000000	-2.9947789999999999	-2.3454820000000001
H	1.2512188000000000	-3.8862646999999999	-2.6047158000000001
H	0.90035054999999997	-3.1016420999999998	-1.3718927000000001
O	2.0620840000000000	-3.9340628000000000	6.3494975000000000
H	2.2025336000000002	-4.9318350999999998	6.3231415999999996
H	1.7451179000000001	-3.7857145999999999	5.4641706000000001
O	-1.5861628000000001	-6.3256462999999998	2.3826502999999999
H	-0.69600951000000000	-6.7032629999999997	2.1499044000000000
H	-1.9511537999999999	-7.0831895999999999	2.8043923999999998
O	-1.1391433000000000	-1.5835695000000001	4.4086698000000002
H	-1.1653989000000000	-2.5513439999999998	4.1497394999999999
H	-0.26017258999999998	-1.5061382999999999	4.6982993999999998
O	4.3873711999999996	-6.6876432000000001	0.47792868999999999
H	4.3687982999999999	-7.6453784000000002	0.42679578000000001
H	3.6467532000000000	-6.4290468000000001	-0.10941948999999999

O	1.3462164999999999	-8.7758564000000003	2.5007766999999999
H	1.3766908000000000	-7.8170225999999996	2.2828860000000000
H	2.3258595000000000	-8.9535765999999999	2.6434473000000001
O	5.8554164000000002	-4.7459519999999999	2.1095961000000001
H	5.4871293999999997	-5.2961793999999998	1.3915093999999999
H	5.4121490999999997	-5.1178955999999998	2.9119849000000002

C	0.17873337E+01	-0.29106480E+01	0.26763491E+01
C	0.18019193E+01	-0.16213183E+01	0.31911312E+01
O	0.18661718E+01	-0.69427032E+00	0.21695984E+01
C	0.18343621E+01	-0.13624619E+01	0.89942192E+00
C	0.17348356E+01	-0.28221016E+01	0.12375033E+01
O	0.17527787E+01	-0.11483731E+01	0.43484023E+01
C	0.57854139E+00	-0.81115349E+00	0.23288526E+00
O	-0.63803329E+00	-0.11182513E+01	0.96441699E+00
O	1.7937934000000000	-3.8177390999999998	0.39687688999999998
O	1.5498741000000000	-3.9985586000000000	3.4327052000000000
C	0.76368052E+00	0.67175118E+00	-0.12021165E+00
O	-0.47489885E+00	0.12398720E+01	-0.53632229E+00
H	0.26381644E+01	-0.11233720E+01	0.16039748E+00
H	0.12584529E+01	0.13114210E+01	0.61231307E+00
H	0.14306366E+01	0.59478510E+00	-0.98003669E+00
H	-0.88966809E+00	0.17374243E+01	0.19187282E+00
H	0.33882824E+00	-0.13207609E+01	-0.69585590E+00
H	-0.55794157E+00	-0.10906574E+01	0.19442811E+01
O	0.93408968999999997	-6.2891548000000004	1.6864718000000001
H	1.0973526000000000	-5.5300259000000000	2.2830716000000000
H	1.4428401000000000	-5.9506227000000003	0.96023937000000004
O	-0.93740038000000003	-4.5731491999999996	0.20116502000000000
H	-4.1161474000000003E-002	-4.1822343999999996	0.38372877999999999
H	-1.0794438000000000	-5.1445983999999996	0.97582522999999999
O	3.6410534999999999	-5.6333510999999996	3.8234905000000001
H	2.8362487000000001	-5.0288003000000003	3.7699170000000000
H	3.4581734000000002	-6.0101861999999997	2.9523532000000001
O	-1.1045448000000000	-4.4470910000000003	4.1705760999999999
H	-0.20803114000000000	-4.3921394999999999	3.7808670000000002
H	-1.4558546999999999	-5.2267250000000001	3.6905891999999998
O	3.9100407000000001	-3.4414614000000001	-1.5511695999999999
H	3.1745491000000001	-3.7298358999999999	-0.92393906000000003
H	4.3190850000000003	-2.6864366999999998	-1.1198409000000000
O	5.6394254999999998	-4.4499089999999999	2.3666866999999998
H	5.0076891000000003	-4.7585971000000002	1.6535678000000000
H	4.9952394000000000	-4.5917944999999998	3.0900848000000001
O	1.0012795999999999	-3.1743456999999999	-2.4387588000000000
H	1.0473091999999999	-4.1248816000000001	-2.7424947999999998
H	1.2649893000000001	-3.2261880999999999	-1.5568276000000001
O	-1.8662331999999999	-6.3770929000000001	2.1947302000000000
H	-0.89518892000000005	-6.4720933000000000	2.2665663000000000
H	-2.2766885000000001	-7.0708304999999996	2.7723361000000000
O	1.5139107000000001	-3.9390065000000001	6.4817507000000001
H	1.6316335000000000	-4.9144423000000002	6.5126958000000004
H	1.4029100000000001	-3.7868580999999999	5.5632890000000002
O	4.3415815000000002	-6.2647655999999996	0.64979293999999999
H	4.5663387999999996	-7.1867796999999998	0.70459744999999996
H	3.6542048000000000	-6.3334380000000001	-7.0556138000000004E-002
O	2.2189665999999999	-6.5838599000000002	-0.89568616000000001
H	2.0125521000000002	-7.4716537000000001	-1.2525955000000000
H	1.7241420000000001	-5.9316991999999997	-1.3327298999999999
O	-1.1556200999999999	-1.6288863000000000	4.0562676000000000
H	-1.2416461999999999	-2.6187342999999998	4.2505544000000004
H	-0.36019971000000001	-1.2485766000000000	4.4517065000000002

C	0.16496266E+01	-0.29802481E+01	0.26626887E+01
C	0.17281340E+01	-0.16318600E+01	0.32331404E+01
O	0.18467098E+01	-0.64658532E+00	0.22306798E+01
C	0.17544772E+01	-0.13833862E+01	0.87392140E+00
C	0.18299046E+01	-0.28495823E+01	0.12340585E+01
O	0.16441313E+01	-0.12182543E+01	0.43892110E+01
C	0.48578705E+00	-0.69542576E+00	0.28270095E+00
O	-0.63004126E+00	-0.72837699E+00	0.11508176E+01
O	1.9446494999999999	-3.7536543000000000	0.38046744999999998
O	1.5371475999999999	-4.1118899999999998	3.3292063999999999
C	0.78365362E+00	0.70772853E+00	-0.22513041E+00
O	-0.39824780E+00	0.14652019E+01	-0.48168223E+00
H	0.25939259E+01	-0.93244409E+00	0.38459649E+00
H	0.14110825E+01	0.13019338E+01	0.47160651E+00
H	0.13231791E+01	0.54651457E+00	-0.11825063E+01
H	-0.78374151E+00	0.16902483E+01	0.36991125E+00
H	0.19348718E+00	-0.12357055E+01	-0.60082951E+00
H	-0.37741874E+00	-0.42829707E+00	0.20563993E+01
O	1.1140759000000000	-6.2848464999999996	1.6317893999999999
H	1.1241961000000000	-5.5288922999999999	2.2832178000000001
H	1.6817726000000000	-5.8715862000000003	0.95078183000000005
O	3.7084269000000001	-5.6227042000000003	3.7949221999999998
H	2.9537078999999999	-4.9147391000000002	3.5543524000000000
H	3.7547171000000001	-6.1223118000000003	2.9105150000000002
O	-0.65454325999999996	-4.5735124000000003	-0.11503566000000000
H	0.34081632000000001	-4.6718983999999999	-6.3938457000000004E-002
H	-1.0543362999999999	-4.9392056999999996	0.78535948000000000
O	-1.0716344000000000	-4.3050883000000004	3.9914456999999999
H	-0.10195395000000000	-4.3199921999999997	4.0377291000000000
H	-1.1855133000000000	-4.6703549999999998	3.0968735000000001
O	4.2129963000000004	-3.4685934999999999	-1.4493810000000000
H	3.5684062000000001	-3.6350368000000000	-0.72660155999999998
H	4.7984396000000000	-2.7916211000000000	-1.0419246000000000
O	4.2937574999999999	-6.3956321000000003	0.58700573000000000
H	4.2353660000000000	-7.3616735999999996	0.48500279000000002
H	3.4026607000000002	-6.1793921999999997	0.22998490999999999
O	1.3554158000000001	-3.1738944999999998	-2.3275150000000000
H	1.3518916000000001	-4.0817880999999998	-2.7418828000000000
H	1.6551079000000000	-3.2790824000000001	-1.4133728999999999
O	-1.6115685000000000	-6.1868261000000002	1.7641376000000000
H	-0.66107099999999996	-6.2890674999999998	1.8096026999999999
H	-2.0004491999999998	-7.0154983000000000	2.0076687000000000
O	1.7424329000000001	-4.0941460000000003	6.4123444999999997
H	1.6416763000000001	-5.0561616000000003	6.4036001999999996
H	1.6611729000000000	-3.8697715000000001	5.4729255999999999
O	5.2455280000000002	-1.8121316999999999	0.28069738000000000
H	4.9733543999999998	-2.5189183000000002	0.84651577000000000
H	5.6493051000000003	-1.2556967999999999	0.95721354000000003
O	5.7070314000000000	-4.5477086000000000	2.1722204000000001
H	5.4351713999999998	-5.0035626000000004	1.4032518000000000
H	5.2490914999999996	-5.1271633999999997	2.7962522000000001
O	2.1951884000000002	-6.4304484999999998	-1.0763387000000000
H	2.2982968000000001	-7.3688832000000000	-1.4109354999999999
H	1.7215529000000001	-6.0427343999999996	-1.8293942000000001

C	0.15490616E+01	-0.28972425E+01	0.26405270E+01
C	0.18438936E+01	-0.16032464E+01	0.32007920E+01
O	0.20586184E+01	-0.65137144E+00	0.22016011E+01
C	0.19190076E+01	-0.13162422E+01	0.90708033E+00
C	0.17403936E+01	-0.28461389E+01	0.12456455E+01
O	0.17616062E+01	-0.11131947E+01	0.43282001E+01
C	0.72646958E+00	-0.73312741E+00	0.18699444E+00
O	-0.49874385E+00	-0.10159703E+01	0.88635146E+00
O	1.6573831999999999	-3.7013856999999999	0.30612129999999999
O	1.2609418999999999	-3.9087987000000002	3.4634410999999998
C	0.92419432E+00	0.83674372E+00	-0.84421029E-01
O	-0.32689977E+00	0.14350856E+01	-0.43351729E+00
H	0.28152128E+01	-0.12774361E+01	0.35336388E+00
H	0.14410165E+01	0.13322353E+01	0.77099778E+00
H	0.15158464E+01	0.95188664E+00	-0.10210269E+01
H	-0.57608112E+00	0.20163594E+01	0.30422314E+00
H	0.58424108E+00	-0.13960856E+01	-0.72146339E+00
H	-0.53055120E+00	-0.60407864E+00	0.17623463E+01
O	1.5972435000000000	-5.9936822000000003	1.7749480000000000
H	1.5087200999999999	-5.5055892000000002	2.6106017000000001
H	1.6028727000000000	-5.1923481999999996	1.1950639999999999
O	-0.75143534999999995	-4.6012868999999998	-0.36761880000000002
H	4.7849633000000003E-002	-4.1964310999999999	-5.0642312000000002E-002
H	-1.0134704999999999	-5.0205408000000000	0.50379708000000001
O	3.5416851999999999	-5.5345651000000000	3.8582746000000001
H	2.7089419000000001	-4.9757949000000004	3.9578142999999999
H	3.5990061000000000	-5.7941742999999999	2.9208402000000002
O	-0.96433840000000004	-4.9470821999999997	4.5154027000000001
H	-0.11543808000000000	-4.5873543999999997	4.2058337999999997
H	-1.3859128999999999	-5.1613341000000004	3.7013881000000000
O	3.9720480000000000	-3.5246281000000002	-1.5369037999999999
H	3.3442691000000000	-3.7761127999999999	-0.86271220000000004
H	4.5220054000000003	-2.9467189000000000	-0.96790334000000000
O	1.9365129000000001	-4.3281669000000003	6.1954044000000001
H	1.6300456999999999	-5.0942895999999998	6.5600379000000002

H	1.7089383000000000	-4.2442929999999999	5.2617493000000000
O	1.1096854000000000	-3.0631374000000000	-2.3968354000000001
H	1.2535590000000001	-3.9653955999999999	-2.6795734000000002
H	1.4686246999999999	-3.1990538000000002	-1.4860913000000000
O	-1.1804905999999999	-2.1541893000000001	4.2993927999999997
H	-1.3657944000000000	-3.1298314000000000	4.1855324999999999
H	-0.25259862000000000	-2.1006806999999998	4.4160465000000002
O	4.3040817000000002	-6.3066852999999998	1.1079363000000000
H	4.1120492000000004	-7.2116521000000002	1.2811166000000000
H	3.7439401999999999	-6.0856994000000002	0.34343240000000003
O	2.2552580999999998	-6.4751548999999997	-1.0127733999999999
H	2.2695286000000001	-7.3936130999999996	-1.2634704000000001
H	1.3249089000000001	-6.4823817999999997	-0.63485049999999998
O	-1.4551982999999999	-6.2613510000000003	1.9254494000000000
H	-0.66795274000000004	-6.7883924999999996	2.3248182000000002
H	-2.1825065000000001	-6.9114551000000004	2.0386557999999999
O	-2.7398186999999998	-2.7768193999999999	0.45250580000000001
H	-2.7786591999999999	-2.9056449999999998	-0.49195172999999998
H	-1.8354440000000001	-2.4313755000000001	0.62130485999999996

C	0.16462238E+01	-0.29146318E+01	0.25824248E+01
C	0.16997191E+01	-0.15202033E+01	0.31428029E+01
O	0.20460991E+01	-0.59704872E+00	0.21943690E+01
C	0.20711727E+01	-0.12862379E+01	0.87071765E+00
C	0.19097183E+01	-0.28098841E+01	0.12055917E+01
O	0.15727111E+01	-0.11558314E+01	0.43274870E+01
C	0.77117866E+00	-0.75224621E+00	0.20534246E+00
O	-0.25651701E+00	-0.10122955E+01	0.11565734E+01
O	2.0029515999999998	-3.7450602000000002	0.29407311000000003
O	1.2991603000000000	-3.9688498000000001	3.3839361999999999
C	0.89987630E+00	0.69033441E+00	-0.14471892E+00
O	-0.34076495E+00	0.12886711E+01	-0.52843236E+00
H	0.30464629E+01	-0.10676708E+01	0.42547355E+00
H	0.12703964E+01	0.12742227E+01	0.77476301E+00
H	0.16563894E+01	0.93162150E+00	-0.88480157E+00
H	-0.92262491E+00	0.14294354E+01	0.26825282E+00
H	0.60581647E+00	-0.13607162E+01	-0.63421165E+00
H	-0.14840628E+00	-0.42304869E+00	0.19244911E+01
O	1.2591026999999999	-5.9881665000000002	1.8186372000000000
H	1.1569288000000000	-5.3923439999999996	2.5954348000000000
H	1.7609695999999999	-5.2945061000000004	1.2873772999999999
O	3.5581296999999998	-5.4401739999999998	3.3874575000000000
H	2.6631366000000001	-5.0171688000000003	3.2804172000000000
H	3.5963558999999998	-6.0035147999999996	2.5647912000000002
O	-0.56673364000000004	-4.8462496000000002	-0.25621370999999998
H	0.22685300000000000	-4.3662932000000003	6.0577442000000002E-002
H	-0.76178794999999999	-5.3685581000000004	0.53432506999999996
O	-1.0639873000000000	-5.0684059000000001	4.3635853999999998
H	-0.20234849999999999	-4.7955430000000003	4.0092740999999998
H	-1.4706052999999999	-5.5679363000000004	3.6154939000000001
O	4.0076989999999997	-3.8004671999999999	-1.5042720999999999
H	3.4824454000000000	-3.9260399000000001	-0.65016609999999996
H	4.4082338999999999	-2.9699553000000001	-1.3271568000000000
O	-1.1418276000000001	-2.3260950999999999	4.2426186000000001
H	-1.2541475000000000	-3.2958085000000001	4.1403363000000004
H	-0.16268627999999999	-2.2503533000000000	4.1594490999999998
O	1.2258861999999999	-3.1459058000000000	-2.4532809000000002
H	1.3098234000000000	-4.0455958000000001	-2.8846948000000001
H	1.5327317000000000	-3.4217349000000000	-1.5094997000000001
O	1.6315805999999999	-4.3988426000000000	6.2209230000000000
H	1.0317107000000001	-5.0325056000000004	6.4363751999999996
H	1.5624813000000000	-4.3486140000000004	5.2600372000000002
O	5.8225252999999997	-4.2578076999999999	2.2073691000000002
H	5.5799807000000001	-4.9936099000000000	1.5764205000000000
H	4.9965320000000002	-4.2766487000000000	2.8299208000000002
O	2.7637737000000002	-6.4934167000000000	-1.1496329999999999
H	2.2646027000000002	-7.2786334000000004	-1.0031090000000000
H	2.0311799000000001	-5.8898282999999996	-1.2040687000000001
O	4.5833909999999998	-6.7086940999999998	1.0361317999999999
H	4.5238202000000003	-7.5907188999999997	0.67720420000000003
H	4.1138352999999999	-6.2749059000000003	0.29032598999999998
O	-1.4356393000000001	-6.2719810999999996	1.7193826999999999
H	-0.68884997999999997	-6.8405205999999996	2.0828326000000001
H	-2.1818628000000002	-6.8243228000000000	1.9276257000000001

C	0.14376633E+01	-0.28429876E+01	0.25942007E+01
C	0.19351772E+01	-0.16289609E+01	0.30364067E+01
O	0.22601313E+01	-0.73041774E+00	0.20019683E+01
C	0.19718931E+01	-0.14627304E+01	0.76458299E+00
C	0.16851431E+01	-0.28551446E+01	0.12004907E+01
O	0.19086730E+01	-0.12414771E+01	0.42425070E+01
C	0.60582657E+00	-0.88481131E+00	0.20341876E+00
O	-0.42384711E+00	-0.10782167E+01	0.11552740E+01
O	1.6048781000000001	-3.7916682000000002	0.31941609999999998
O	1.0707926999999999	-3.8322989999999999	3.3597964999999999
C	0.70612115E+00	0.63080173E+00	-0.68983321E-01
O	-0.49077256E+00	0.12101762E+01	-0.46926419E+00
H	0.27645271E+01	-0.14271925E+01	-0.38336303E-01
H	0.97583617E+00	0.10304143E+01	0.87961021E+00
H	0.14082250E+01	0.77680923E+00	-0.87989019E+00
H	-0.96954499E+00	0.12203198E+01	0.32092251E+00
H	0.32588386E+00	-0.13687865E+01	-0.77229000E+00
H	-0.12350424E+00	-0.17862302E+01	0.18427144E+01
O	1.3355589000000001	-6.0888923999999998	1.9366137999999999
H	1.2714334000000000	-5.1860062999999998	2.4195350000000002
H	1.6615940000000000	-5.7417261000000002	1.1083177000000000
O	-0.86595646000000004	-4.7817612000000000	-0.29685334000000002
H	1.6556998000000000E-002	-4.3804613999999997	-2.4939077000000000E-002
H	-1.2238705000000001	-4.8655416999999996	0.57118829999999998
O	3.5890477000000001	-5.2399725999999998	3.5575244000000001
H	3.3045811000000000	-4.4702203000000003	4.0975083999999997
H	2.7545220000000001	-5.5684810000000002	3.1991930000000002
O	-1.0374348000000000	-2.4805043000000002	4.6854417000000002
H	-0.41229937000000000	-3.1376824000000001	4.2725596000000001
H	-0.39420454999999999	-1.9931291000000000	5.1844887999999996
O	-0.93118347000000001	-5.4437616000000002	4.6067660999999998
H	-0.29413317999999999	-4.8782062000000002	4.2149786000000002
H	-1.4529185000000000	-5.5932931000000004	3.8119874000000000
O	-1.6549187999999999	-6.1407211000000004	2.0636944000000002
H	-0.71829933999999995	-6.3646183000000001	2.0823594000000001
H	-2.0367451000000001	-6.9189040999999998	2.4545016999999998
O	1.0807089999999999	-3.0808865999999999	-2.3531844999999998
H	1.0033272000000000	-4.0024727000000002	-2.7503491000000002
H	1.0964214999999999	-3.2996384999999999	-1.3999843000000001
O	4.0735628000000004	-3.7493099000000001	-1.5832655000000000
H	3.2743456000000002	-3.8996148000000002	-1.0872990000000000
H	4.6159536000000001	-3.2402628000000000	-0.94287701999999995
O	1.9358607999999999	-4.3477557999999998	6.1812155000000004
H	1.3366450999999999	-4.9722344999999999	6.5126511999999996
H	1.6149254000000000	-4.2670446000000002	5.3212773000000002
O	-2.5364399000000000	-2.7041791000000002	0.40128068000000000
H	-2.1058764000000001	-3.2170372999999999	-0.30441391000000001
H	-1.8461528000000000	-2.0869686000000001	0.74630991999999996
O	2.4529865000000002	-6.4198830999999998	-1.1254109000000001
H	2.0811316999999998	-7.2753236000000001	-1.2551767000000000
H	1.7405455999999999	-5.8281928000000001	-1.4388612000000001
O	0.98035746999999995	-8.6467796000000003	2.5795200999999999
H	1.1854490000000000	-7.7223163000000001	2.2652516999999999
H	1.8812930999999999	-8.9734984999999998	2.5915501000000001

C	0.16188385E+01	-0.29120408E+01	0.26655029E+01
C	0.18739273E+01	-0.15880918E+01	0.31370610E+01
O	0.22748900E+01	-0.74278800E+00	0.20991965E+01
C	0.20739155E+01	-0.14566145E+01	0.86743005E+00
C	0.18784657E+01	-0.29252301E+01	0.13022061E+01
O	0.18323907E+01	-0.11432443E+01	0.42926932E+01
C	0.79015318E+00	-0.87130398E+00	0.31188246E+00
O	-0.36556629E+00	-0.12066436E+01	0.11282198E+01
O	1.7786549000000000	-3.8241570999999999	0.38423732999999999
O	1.3485008000000001	-3.9099495000000002	3.5326529999999998
C	0.82354068E+00	0.68131298E+00	0.15510016E+00
O	-0.40510473E+00	0.12682098E+01	-0.30240674E+00
H	0.29594468E+01	-0.12933140E+01	0.25596376E+00
H	0.12112346E+01	0.13009487E+01	0.10277567E+01
H	0.15265371E+01	0.88233590E+00	-0.67831080E+00
H	-0.89632848E+00	0.16781598E+01	0.40770449E+00
H	0.59438846E+00	-0.12277969E+01	-0.68399607E+00
H	-0.25887987E+00	-0.19521748E+01	0.17362787E+01
O	1.3245575000000001	-6.1027525999999996	1.8248598000000000
H	1.1109116000000001	-5.3815622000000003	2.4770550999999998
H	1.5258451000000000	-5.5625530000000003	1.0676793000000000
O	3.6080592000000000	-5.2883490000000002	3.8488554000000001

H	2.8927941000000001	-4.6011687999999999	3.7619015999999998
H	2.9483313999999998	-6.0689190999999996	3.9073033000000001
O	-0.60127458000000000	-4.9348643000000001	-0.66821251999999998
H	0.33665663000000001	-4.7115486000000004	-0.59436586999999996
H	-0.89231623999999998	-4.7841961000000000	0.28284716999999998
O	-1.1370161000000001	-5.3576853000000000	4.4879832999999998
H	-0.42500011999999998	-4.9816310000000001	3.9458072999999998
H	-1.4965021000000001	-5.9005121999999997	3.8698369000000001
O	1.1129312000000000	-3.1420854999999999	-2.2323458000000000
H	0.92645005999999996	-4.0015992000000002	-2.6517648999999999
H	1.4834094000000000	-3.3614834000000000	-1.3185366999999999
O	-1.0248967000000000	-2.2211210000000001	4.8859807999999996
H	-0.39842083000000000	-2.8746402999999998	4.5030951999999997
H	-0.47947167000000002	-1.6685691000000000	5.4117313999999999
O	1.6196261000000001	-4.1863966000000001	6.3082662999999997
H	1.4632806000000000	-5.0475063000000002	6.5737629999999996
H	1.4285884000000000	-4.1233947000000004	5.3105764999999998
O	4.3827847000000002	-6.7896533999999997	0.94036832999999997
H	3.6833222000000001	-6.7646345999999999	1.6397493999999999
H	3.8737946000000001	-6.4757416000000001	0.15000322999999999
O	4.3749400999999999	-3.6547858000000000	-1.6285788999999999
H	3.6642166000000000	-4.0040579999999997	-1.1170947000000000
H	4.7029249999999996	-2.9178557999999999	-1.0865975999999999
O	-1.9237010999999999	-6.0655609000000004	1.8591253000000001
H	-1.1859527999999999	-6.6285261999999996	1.5155700999999999
H	-2.2325548000000000	-6.7613127999999998	2.5087595000000000
O	5.2521212999999998	-2.0749770999999999	0.36341929000000001
H	5.2725058000000002	-2.8388160999999998	0.97268555000000001
H	5.7615197000000000	-1.3970681000000000	0.97988143000000005
O	3.8166226000000001	-2.5830665000000002	5.7381622999999999
H	3.0534488999999998	-2.2388823000000002	5.2777241999999998
H	3.4982590999999998	-3.4204984000000000	6.1044546000000004

C	0.15969875E+01	-0.29345006E+01	0.26342080E+01
C	0.20306595E+01	-0.16541541E+01	0.31191330E+01
O	0.21864192E+01	-0.74230658E+00	0.21185708E+01
C	0.18821655E+01	-0.14599026E+01	0.86559388E+00
C	0.17126899E+01	-0.28943610E+01	0.12321599E+01
O	0.20928552E+01	-0.12582341E+01	0.43230998E+01
C	0.69908643E+00	-0.74174493E+00	0.29711326E+00
O	-0.52811789E+00	-0.99871819E+00	0.10690311E+01
O	1.5592728000000000	-3.8316395000000001	0.38293481000000001
O	1.3066917000000000	-3.9820538999999999	3.4802165999999999
C	0.83391935E+00	0.78580771E+00	0.28409364E+00
O	-0.29685719E+00	0.13625938E+01	-0.33405593E+00
H	0.27779188E+01	-0.12146780E+01	0.24243423E+00
H	0.85459206E+00	0.11258177E+01	0.13597825E+01
H	0.18310467E+01	0.11681937E+01	-0.10674961E+00
H	-0.72931978E+00	0.17352287E+01	0.48809096E+00
H	0.50319457E+00	-0.11281913E+01	-0.70090799E+00
H	-0.35750692E+00	-0.74590196E+00	0.19606840E+01
O	1.1820930000000001	-6.0913399000000004	1.8253710999999999
H	0.79985273999999995	-5.4863796999999996	2.5796717000000000
H	1.5205378000000001	-5.3878424999999996	1.1894340000000001
O	-0.75122230000000001	-4.8870884999999999	-0.56679029000000003
H	0.12903724000000000	-4.6194078999999997	-0.21134169000000000
H	-1.2745685000000000	-4.3019970000000001	-6.9145261000000003E-003
O	3.5997891000000002	-5.2042647000000004	4.0411275000000000
H	2.9088718999999998	-4.4982721000000003	4.2309163999999999
H	3.1322969000000001	-5.5345101000000003	3.2178019000000000
O	-1.2217829000000000	-4.5148622999999999	4.3202859000000000
H	-0.34777947999999997	-4.3818570000000001	3.8814793999999999
H	-1.7496171000000000	-4.9511165999999998	3.6024362000000001
O	4.1110315999999996	-6.2998531000000000	0.83685213999999997
H	3.1653402000000002	-6.3674764000000001	1.2154453999999999
H	3.8765092999999999	-6.1747541999999997	-9.0141120000000005E-002
O	1.4531380000000000	-3.0157878000000000	-2.1250271000000001
H	1.1801628000000000	-3.9206953000000002	-2.3686870000000000
H	1.7880739000000001	-3.1307209000000000	-1.2320762999999999
O	-1.6893129000000000	-6.0133631999999997	2.0292796000000002
H	-0.91149272999999997	-6.2397182999999998	1.5922506000000001
H	-2.1151977000000000	-6.8933147000000004	2.3623318000000002
O	1.2501650000000000	-3.8354572000000000	6.2896646000000000
H	1.3506737000000000	-4.7891684000000003	6.3860318999999999
H	1.1692207999999999	-3.7722931000000002	5.2989107999999998
O	5.4927922999999996	-4.1856762999999999	2.3328454999999999
H	5.1430156000000000	-4.9081878000000003	1.7715263000000001
H	4.9560541999999996	-4.2833418999999999	3.1400318000000000
O	1.2051289000000001	-8.6014034000000006	2.5348071999999999
H	1.1541283000000000	-7.6295468000000000	2.2716620999999999
H	2.1086230000000001	-8.8943957000000005	2.5949018000000001
O	-1.0483404000000001	-1.8411575000000000	4.3960270000000001
H	-1.2678248999999999	-2.7967070000000001	4.4264881999999997
H	-8.3256811000000000E-002	-1.7062609000000000	4.7617491999999997
O	4.4441718000000003	-3.4682572999999999	-1.5840183000000001
H	3.5787442000000000	-3.5937125000000001	-1.2645728000000001
H	4.7585889000000003	-2.7598962000000000	-1.0297430999999999

C	0.15438651E+01	-0.28375602E+01	0.26628892E+01
C	0.17679928E+01	-0.15829290E+01	0.32257970E+01
O	0.19982960E+01	-0.61385272E+00	0.22063739E+01
C	0.17940289E+01	-0.12640603E+01	0.94607819E+00
C	0.16087524E+01	-0.26931302E+01	0.12218910E+01
O	0.20107500E+01	-0.12071864E+01	0.44290809E+01
C	0.55102077E+00	-0.60855370E+00	0.25370766E+00
O	-0.67181312E+00	-0.10275979E+01	0.97845539E+00
O	1.6027420999999999	-3.6394834999999999	0.38003434000000003
O	1.2474742999999999	-3.9183932000000001	3.3677719000000002
C	0.71779888E+00	0.85508771E+00	0.27105992E+00
O	-0.48880355E+00	0.15736672E+01	-0.15081445E+00
H	0.26191715E+01	-0.10135163E+01	0.27548856E+00
H	0.99644416E+00	0.11623222E+01	0.12718908E+01
H	0.15197337E+01	0.11752444E+01	-0.36266170E+00
H	-0.72277217E+00	0.23109200E+01	0.40962258E+00
H	0.42640393E+00	-0.99085473E+00	-0.75834606E+00
H	-0.44268832E+00	-0.11602826E+01	0.19033388E+01
O	1.0238421000000000	-6.0621333999999996	1.6541056999999999
H	0.86996868999999999	-5.4207713000000002	2.3661219000000000
H	1.3962087000000001	-5.4235954000000000	0.96934494999999998
O	-0.82746615999999995	-4.5032848000000003	-0.43923551000000000
H	4.8432725000000003E-002	-4.2113497000000004	-0.12577754999999999
H	-1.2215075000000000	-4.8285998000000001	0.37214743000000000
O	3.3554737000000001	-5.1932666999999997	4.2647747999999996
H	2.6863022999999999	-4.5160174000000000	3.9916507000000001
H	2.8877207999999999	-5.8666008999999999	3.7644426000000002
O	-1.2098342000000000	-4.5608148000000002	4.4655680999999996
H	-0.29770703999999998	-4.4010980999999996	4.0242969999999998
H	-1.8138190000000001	-4.8089234000000003	3.6777096999999999
O	1.5492763000000001	-2.8788649999999998	-2.2811203999999998
H	1.5146417000000001	-3.7462993000000000	-2.6950585999999999
H	1.5332275000000000	-3.0564311000000002	-1.3198806000000001
O	5.2149266000000001	-4.1066532000000002	2.1518432999999999
H	5.2371451999999996	-4.9554137999999996	1.6387007000000000
H	4.6064290999999997	-4.3609200000000001	2.8865715999999999
O	-1.8625510000000001	-5.9426464000000001	1.7839434999999999
H	-0.96918205000000002	-6.2243570000000004	1.8790878000000000
H	-2.3000001999999999	-6.4882774999999997	2.4768164000000001
O	1.6782121000000001	-3.9310467999999998	6.2741810999999998
H	1.6772689000000001	-4.8705037999999998	6.4965004999999998
H	1.4534315000000000	-3.9282352000000000	5.3091039999999996
O	-1.0428930999999999	-1.5412739000000000	4.4574293999999997
H	-0.95417048000000004	-2.4005512000000002	4.1122790000000000
H	-0.28554891999999998	-1.5080249999999999	4.9439054000000002
O	5.0526625999999997	-1.9265040000000000	0.51839128999999995
H	5.0327641999999999	-2.6070028999999999	1.2280838000000001
H	5.7656820000000000	-1.4072827000000001	0.83339673999999997
O	4.4184038000000001	-6.3673751000000003	0.62490241000000002
H	4.0718503999999998	-6.7366095000000001	1.4886801000000001
H	3.6154937000000000	-6.4839009000000001	6.7952788000000000E-002
O	1.4128704999999999	-8.6834852999999992	2.2078788000000000
H	1.2992400000000000	-7.7503174000000001	1.9051572000000001
H	2.2843523999999999	-8.6015768000000001	2.6096583999999998

C	0.14862316E+01	-0.28878974E+01	0.24425595E+01
C	0.17117788E+01	-0.16050268E+01	0.30392992E+01
O	0.19098927E+01	-0.59783660E+00	0.21384556E+01
C	0.17763237E+01	-0.12105547E+01	0.83141495E+00
C	0.16219440E+01	-0.27055284E+01	0.10652925E+01
O	0.18836163E+01	-0.12481921E+01	0.42674631E+01
C	0.59258260E+00	-0.55420382E+00	0.18562217E+00
O	-0.64007836E+00	-0.83023880E+00	0.89228543E+00
O	1.7939943000000000	-3.6148064999999998	0.13442519999999999

O	1.3736507000000000	-4.0295183999999997	3.1156761999999998
C	0.73396789E+00	0.97107677E+00	0.29177275E+00
O	-0.38715654E+00	0.17653048E+01	-0.38509460E+00
H	0.26315151E+01	-0.98471767E+00	0.17098883E+00
H	0.90834166E+00	0.13785377E+01	0.12605499E+01
H	0.15913948E+01	0.13389897E+01	-0.27726936E+00
H	-0.11357078E+01	0.19655120E+01	0.22532629E+00
H	0.47047762E+00	-0.10332033E+01	-0.80620781E+00
H	-0.39956848E+00	-0.85069377E+00	0.18223866E+01
O	1.1636416000000001	-6.1719211999999999	1.3655940000000000
H	1.3024112999999999	-5.5617732999999996	2.1213201000000002
H	1.3321056000000000	-5.5711971000000000	0.57437762000000003
O	-0.84706029000000005	-4.6245174999999996	-0.30259788999999998
H	0.13670061999999999	-4.4899127999999999	-0.37412769000000001
H	-1.0537071000000000	-4.8406076000000002	0.64876820000000002
O	3.5528018000000001	-5.2299198000000002	4.0204601999999996
H	3.6264859000000000	-4.6380974999999999	4.8078317000000004
H	2.6746159999999999	-5.0060004999999999	3.6264560000000001
O	-0.94158392000000002	-4.4879316999999999	4.3654554000000001
H	-0.18682847999999999	-4.2327193999999997	3.8762050000000000
H	-1.4573791000000000	-4.9985936000000004	3.7631494999999999
O	4.2369253000000002	-5.8252822000000002	0.78504863999999996
H	3.9514260999999999	-6.6170134000000003	1.2617818000000001
H	3.4950975999999998	-5.6278841999999996	0.18997412000000000
O	0.95713181000000003	-3.0385469999999999	-2.3458317000000002
H	0.97013263999999999	-4.0320957000000002	-2.5447647999999998
H	1.3826478000000000	-2.9681590000000000	-1.4371194000000000
O	4.2748686999999999	-3.2286902000000000	-1.4766442000000000
H	3.5373803000000001	-3.7223752000000001	-0.99696435000000005
H	4.7426744999999997	-2.7689651999999998	-0.72598872000000003
O	5.5675943999999999	-4.1948186999999999	2.2024257000000000
H	5.2336144999999998	-4.7551601000000003	1.4879971999999999
H	4.7196524000000002	-4.1454481999999997	2.7269258000000001
O	1.7304603000000001	-3.6638772999999998	6.1754373999999999
H	1.3174515000000000	-4.5512120999999999	6.3209740999999999
H	1.4696170000000000	-3.4886680999999999	5.2437788000000003
O	-1.8153615999999999	-6.0348202999999998	2.0189430000000002
H	-0.93854104999999999	-6.4959026000000000	1.9617312000000000
H	-2.2870997000000002	-6.6267123000000003	2.6531394000000001
O	2.3680921000000001	-6.4181717999999996	-1.2079362000000000
H	2.2667006999999999	-7.4145127999999998	-1.2691732000000000
H	1.7091149999999999	-6.0993931999999997	-1.8745442000000001
O	5.3851465999999997	-1.7945473000000001	0.66967750000000004
H	5.3449843000000001	-2.4284761000000001	1.3874025000000001
H	6.2621770000000003	-1.3454140999999999	0.85751458999999997

C	0.16102816E+01	-0.28314684E+01	0.25483858E+01
C	0.20131928E+01	-0.16259773E+01	0.30830961E+01
O	0.21751013E+01	-0.64135208E+00	0.21045203E+01
C	0.18630016E+01	-0.12027365E+01	0.86498375E+00
C	0.17013273E+01	-0.27148838E+01	0.11530183E+01
O	0.21842724E+01	-0.12951637E+01	0.42997581E+01
C	0.50629520E+00	-0.51793700E+00	0.31187706E+00
O	-0.65711381E+00	-0.10330262E+01	0.97034274E+00
O	1.6733070000000001	-3.6600728000000000	0.32524252999999997
O	1.4245953000000000	-3.8443399999999999	3.3193239999999999
C	0.57293364E+00	0.10054253E+01	0.31415873E+00
O	-0.34896957E+00	0.17230739E+01	-0.47197717E+00
H	0.26348684E+01	-0.92270023E+00	0.95937237E-01
H	0.49916013E+00	0.13816797E+01	0.13450155E+01
H	0.15612553E+01	0.13997372E+01	0.47387684E-01
H	-0.43431808E+00	0.25940150E+01	-0.69474136E-01
H	0.44621577E+00	-0.70884857E+00	-0.75629941E+00
H	-0.34912056E+00	-0.14234329E+01	0.18196474E+01
O	0.95015022000000005	-6.1059675999999996	1.6375401999999999
H	1.2793513999999999	-5.3381039000000001	2.1473612000000002
H	1.0282822000000000	-5.8626062000000001	0.72691636999999998
O	-0.93803756999999999	-4.5307991999999997	-0.13539448000000001
H	-1.8899774000000001E-002	-4.3328128000000001	0.10318370000000000
H	-1.1759636000000000	-5.0193477000000000	0.69484780999999995
O	3.4210750999999999	-5.4809029999999996	4.0204668999999997
H	3.2991039999999998	-4.9113613000000003	4.8431182000000002
H	2.5447863000000002	-5.1176636000000002	3.6225052999999998
O	-1.0877827000000000	-4.3129533000000002	4.2103758999999998
H	-0.15016046999999999	-4.2164428000000003	3.7952773999999998
H	-1.4353031000000001	-5.0447857999999997	3.6325606000000001

O	0.98721020000000004	-2.8744374000000001	-2.2085067000000000
H	1.5283207000000001	-3.4616839000000001	-2.7004077000000000
H	1.2264204999999999	-3.1753133999999998	-1.2906915000000001
O	3.9956570999999999	-3.5783613999999999	-1.5826811000000001
H	3.4449103000000001	-4.1051659999999996	-0.98259936999999997
H	4.2391810000000003	-2.9049640999999999	-0.92129258000000003
O	1.7805591000000001	-3.6613948999999999	6.1721028000000002
H	1.5012489000000000	-4.5745405000000003	6.2275467999999998
H	1.6281671000000000	-3.4450064999999999	5.2235775999999996
O	4.3976305000000000	-5.8938752000000001	0.62826150999999997
H	3.7841719999999999	-6.2866514000000002	1.2053611000000000
H	3.9251206999999999	-6.0333870000000003	-0.20041679000000001
O	5.1474494999999996	-2.0541076000000000	0.73314595000000005
H	4.7569986999999996	-2.5316812999999998	1.4951585999999999
H	5.7666567000000004	-1.5071371000000000	1.2381686999999999
O	-1.8668026000000000	-6.0251979999999996	1.9239195000000000
H	-0.97337161000000005	-6.2576596999999996	2.1948048000000000
H	-2.4314268000000001	-6.6630995999999998	2.4610827999999998
O	-1.0922016999999999	-1.5858350999999999	4.2519429000000004
H	-1.1591484999999999	-2.5512839000000000	4.2006553999999996
H	-0.11830700000000000	-1.5077275999999999	4.2898942000000000
O	5.7476475000000002	-4.1796778999999997	2.7032167000000000
H	5.3646735000000003	-4.6631159000000002	1.9755341000000000
H	5.2882813999999998	-4.5210702999999999	3.5181648000000001

C	0.16251394E+01	-0.28581894E+01	0.26494446E+01
C	0.18707965E+01	-0.15949903E+01	0.32925130E+01
O	0.19895217E+01	-0.59022187E+00	0.23498426E+01
C	0.18805113E+01	-0.11537740E+01	0.10479014E+01
C	0.17613335E+01	-0.26136454E+01	0.12673386E+01
O	0.18843738E+01	-0.12632368E+01	0.44882945E+01
C	0.75305492E+00	-0.55251195E+00	0.33361360E+00
O	-0.48159576E+00	-0.99437900E+00	0.88671671E+00
O	1.8741555000000001	-3.4786782999999999	0.30743329000000003
O	1.4855155000000000	-3.9317994999999999	3.4170183999999999
C	0.70865857E+00	0.94830012E+00	0.44188360E+00
O	-0.33494484E+00	0.16169523E+01	-0.20965112E+00
H	0.27883817E+01	-0.86776946E+00	0.48407088E+00
H	0.75818217E+00	0.12184631E+01	0.14276187E+01
H	0.15693792E+01	0.14181171E+01	-0.82408885E-02
H	-0.11194276E+01	0.15733533E+01	0.40120881E+00
H	0.73657635E+00	-0.87842511E+00	-0.69801409E+00
H	-0.25268278E+00	-0.13800170E+01	0.17778187E+01
O	1.3315882999999999	-6.1705826000000004	1.5744590999999999
H	1.2373320999999999	-5.4869377999999998	2.2773325000000000
H	1.4838055999999999	-5.5126328000000004	0.84568933999999996
O	3.4855366000000001	-5.3299479999999999	4.2583276999999997
H	3.8186730999999998	-4.4297601000000002	4.5409221999999998
H	2.6254922000000001	-4.9468369000000001	3.9174601000000000
O	-0.86546140999999999	-4.9224534999999996	-0.55016991999999998
H	-2.4641830999999999E-002	-4.6410308999999996	-0.15707345000000000
H	-1.2448896000000000	-5.3298024000000002	0.26100340000000000
O	-1.0950753000000000	-4.2582848999999996	4.0772883000000002
H	-0.12748852999999999	-4.2147037999999997	3.9498516000000001
H	-1.3512131999999999	-4.9314220000000004	3.3980440999999999
O	4.5609203000000003	-5.6515009999999997	0.68174763999999999
H	4.0996354000000004	-6.4962036999999997	0.83333972000000001
H	3.8974001000000000	-5.0524811999999999	0.32901744999999999
O	0.94816350000000005	-2.9492012999999999	-2.1695815999999999
H	0.97186729000000005	-3.8205133000000000	-2.5709339999999998
H	1.0215249000000000	-3.1313319000000002	-1.2039675999999999
O	0.93971930999999997	-3.7260537999999999	6.2808402000000001
H	1.1840417999999999	-4.6406887000000001	6.1610455000000002
H	1.1274850000000001	-3.4771971000000002	5.3131181999999999
O	5.0758260999999996	-2.0816596000000001	0.62193242000000004
H	5.0050659000000000	-2.4918965000000002	1.4583240000000000
H	5.9399749999999996	-1.6288273000000000	0.74127841000000005
O	-1.3935278000000000	-6.0947332000000003	1.9601360000000001
H	-0.52052522999999995	-6.5705710000000002	1.9450780000000001
H	-1.9915160000000001	-6.7533127000000004	2.2721003000000000
O	4.1536087000000004	-2.6853549999999999	5.4807043000000002
H	3.6194896999999999	-2.2857723000000001	4.7982911000000001
H	3.4887264999999998	-2.8938839000000001	6.1406235000000002
O	-1.0275831000000000	-1.5716030999999999	4.1748830000000003
H	-1.1080399999999999	-2.5555748000000000	4.2668273000000001
H	-0.14242089999999999	-1.3698577000000001	4.4638144000000004

O	5.8094127000000002	-4.0093744999999998	2.6730838000000001
H	5.4967347000000002	-4.3258764999999997	1.8317564000000000
H	5.8085785000000003	-4.8090731000000000	3.2522706000000001

C	0.18059173E+01	-0.29069395E+01	0.26111476E+01
C	0.18229939E+01	-0.15746072E+01	0.30758342E+01
O	0.20609104E+01	-0.67662942E+00	0.20771183E+01
C	0.18424985E+01	-0.13397820E+01	0.81837544E+00
C	0.18114103E+01	-0.28266402E+01	0.12266881E+01
O	0.18212311E+01	-0.11478428E+01	0.42850916E+01
C	0.46902741E+00	-0.78707243E+00	0.19598354E+00
O	-0.73288244E+00	-0.12569243E+01	0.77620056E+00
O	1.8401472000000001	-3.7335482000000000	0.38459893000000001
O	1.7738364000000000	-3.9847883999999998	3.4353373000000000
C	0.61595570E+00	0.75478550E+00	0.26427926E+00
O	-0.55172050E+00	0.13872865E+01	-0.15713632E+00
H	0.26193066E+01	-0.12677206E+01	0.87452310E-01
H	0.77167608E+00	0.11204182E+01	0.12546579E+01
H	0.13752534E+01	0.11652906E+01	-0.41148506E+00
H	-0.12599979E+01	0.12772251E+01	0.55422342E+00
H	0.57120090E+00	-0.12323297E+01	-0.83824202E+00
H	-0.50820191E+00	-0.19422923E+01	0.13934243E+01
O	1.4357354000000000	-6.1337329000000000	1.8246765000000000
H	1.4814065000000001	-5.5415182999999999	2.5774889999999999
H	1.8400751000000000	-5.4104355000000002	1.2505052999999999
O	-0.92284926000000000	-4.2016957000000001	3.8125157999999999
H	3.1173223999999999E-002	-4.1102372999999996	3.6610160999999999
H	-1.1500847000000001	-4.8562944000000003	3.1176035999999998
O	3.7881293000000000	-5.2149565000000004	4.6492179000000000
H	4.0384251000000004	-4.5676474000000002	5.3243745999999996
H	3.1384382999999998	-4.7395906999999999	4.0506732000000003
O	4.3917764999999997	-5.4456895000000003	0.93641247000000005
H	4.1772403000000002	-6.3500012999999997	1.2644500000000001
H	3.6079482000000000	-5.1514705999999997	0.37663889000000000
O	-0.89487494000000001	-4.5912521000000002	-0.39031139999999998
H	6.0207312999999998E-002	-4.5770049999999998	-0.10874375999999999
H	-1.2151367000000000	-4.9785599999999999	0.43973247999999998
O	1.5025162000000001	-3.1353868999999999	-2.0793691000000001
H	1.1715229000000000	-4.0312824999999997	-2.4476779999999998
H	1.7414871000000001	-3.3934929999999999	-1.1821386000000000
O	-1.3990710000000000	-5.9439152000000002	1.8488937999999999
H	-0.45852133000000000	-6.1608798000000000	1.8123221000000000
H	-1.8063077999999999	-6.7633777000000004	2.2164411999999998
O	1.7198446999999999	-4.0002849999999999	6.3073610000000002
H	1.5963609000000001	-4.9515982000000003	6.5013893999999999
H	1.5211501000000001	-3.8946570999999999	5.3799219999999996
O	5.8221593000000000	-3.9871903000000000	2.7131788000000001
H	5.2636605000000003	-4.3779566000000001	2.0198018000000002
H	5.6266540000000003	-4.6116602000000002	3.4451670999999999
O	-0.98884647999999997	-1.5856106000000001	4.2121051999999999
H	-0.92201007999999995	-2.4952250999999999	3.8513039000000000
H	-4.4427003000000000E-002	-1.3165102000000000	4.2975238999999998
O	4.0676750999999998	-2.5802147000000000	5.4899437000000004
H	3.3960419000000002	-2.4239652000000000	4.8305612000000000
H	3.5558377999999999	-2.5446468000000002	6.3462972000000004
O	4.2739763999999996	-3.4304334000000001	-1.7955140000000001
H	3.4793270999999999	-3.1886356999999999	-1.3387370000000001
H	4.9270569000000002	-3.0634698000000000	-1.1800044000000001

C	0.19891018E+01	-0.30743361E+01	0.26178762E+01
C	0.21068984E+01	-0.17649864E+01	0.31941614E+01
O	0.20825925E+01	-0.81085916E+00	0.21758500E+01
C	0.19357054E+01	-0.14375389E+01	0.90597785E+00
C	0.18610834E+01	-0.29280509E+01	0.12449310E+01
O	0.19898449E+01	-0.13626141E+01	0.43964929E+01
C	0.63862999E+00	-0.91742563E+00	0.28846088E+00
O	-0.51322062E+00	-0.14053607E+01	0.94281960E+00
O	1.7012138999999999	-3.8545080000000000	0.40145723999999999
O	1.7483630999999999	-4.1811577000000000	3.3997738000000002
C	0.55174091E+00	0.59082891E+00	0.20468054E+00
O	-0.73533610E+00	0.10818731E+01	-0.24396167E+00
H	0.27536554E+01	-0.11813502E+01	0.28222142E+00
H	0.86352549E+00	0.11166724E+01	0.11171023E+01
H	0.13056229E+01	0.92797063E+00	-0.58188451E+00
H	-0.13226078E+01	0.11622174E+01	0.52004679E+00
H	0.62374301E+00	-0.13844279E+01	-0.72365207E+00
H	-0.28521497E+00	-0.14318513E+01	0.18861926E+01
O	1.5040526999999999	-6.2349914000000002	1.5082781999999999
H	1.3644049000000000	-5.5579603999999998	2.2106526000000000
H	1.8301798000000000	-5.6353572999999999	0.79161320000000002
O	-0.84814575000000003	-4.5859287999999996	4.0795629000000000
H	0.11791081000000000	-4.6040584000000004	3.8900671000000000
H	-1.1613088000000000	-4.9674202000000003	3.2492996999999999
O	-0.80926498999999996	-4.5412856000000001	-0.38322927000000001
H	9.3305026999999999E-002	-4.2325955999999998	-0.28331972000000000
H	-0.88856999999999997	-5.0975769000000000	0.39443990000000001
O	4.3356098999999997	-5.5566526999999999	1.0262027000000000
H	4.1003565000000002	-6.3809898000000000	1.5156794000000000
H	3.5764216000000002	-5.6019819000000002	0.37507245000000000
O	3.7589978999999998	-5.3519920000000001	4.7284185000000001
H	3.9930298000000000	-4.4216334000000002	4.7006237000000004
H	2.9089464999999999	-5.3371506999999996	4.3121745000000002
O	-1.1969464000000001	-6.2999280000000004	1.7987074999999999
H	-0.20897898000000001	-6.4142020999999998	1.6109203999999999
H	-1.4975977000000000	-6.9113952000000003	2.4954589000000000
O	1.8916724000000000	-3.0798722000000001	-2.1945275999999998
H	1.5071812000000000	-3.9178392999999998	-2.4502755000000001
H	1.7544050000000000	-3.2736469000000001	-1.1943832000000001
O	1.7824327000000000	-3.9415651999999999	6.4592998000000001
H	1.8889608000000000	-4.9390156999999997	6.6454231999999998
H	1.6483718000000001	-3.9143805000000000	5.4941250999999998
O	6.0484526000000001	-4.2747644999999999	2.7502651999999999
H	5.3060517000000003	-4.7152285999999997	2.2690106999999999
H	5.8794741000000004	-4.7720415000000003	3.5421347999999999
O	-0.88661159000000000	-1.7297716999999999	4.1538184999999999
H	-0.93302890000000005	-2.7304157999999998	4.1017064000000003
H	-7.9582283999999996E-004	-1.6692697000000001	4.5085205999999998
O	1.1586978999999999	-8.6848893999999994	2.4196753000000002
H	1.1909471000000000	-7.7459118000000000	2.0971977000000002
H	2.0929608000000002	-8.7355315999999998	2.6679018999999999
O	5.1193071999999997	-2.3147616000000002	0.89741884999999999
H	5.5622094000000004	-3.0126069000000002	1.3589382999999999
H	5.2169534000000004	-1.5796892000000000	1.4759268000000001

C	0.17798745E+01	-0.30190749E+01	0.25447503E+01
C	0.20209967E+01	-0.17552040E+01	0.30932571E+01
O	0.21746883E+01	-0.80904572E+00	0.21102091E+01
C	0.19335830E+01	-0.14866855E+01	0.84211068E+00
C	0.17979276E+01	-0.29544946E+01	0.11480922E+01
O	0.20266871E+01	-0.13659203E+01	0.42972558E+01
C	0.63508804E+00	-0.91561634E+00	0.27865067E+00
O	-0.42603727E+00	-0.12400778E+01	0.11826841E+01
O	1.7146034999999999	-3.9769670000000001	0.37158436000000000
O	1.7280072000000000	-4.0895935000000003	3.3276740000000000
C	0.56422637E+00	0.60806594E+00	0.18850991E+00
O	-0.66495894E+00	0.10918429E+01	-0.24186241E+00
H	0.27861576E+01	-0.13081693E+01	0.14706568E+00
H	0.83677155E+00	0.94843702E+00	0.11489756E+01
H	0.12816731E+01	0.10401653E+01	-0.49141339E+00
H	-0.13222610E+01	0.95226778E+00	0.44987442E+00
H	0.36039137E+00	-0.13690026E+01	-0.72591083E+00
H	-0.19614395E+00	-0.79932995E+00	0.20281772E+01
O	1.5078925000000001	-6.2830807000000002	1.5931242999999999
H	1.5205815000000000	-5.6219565999999999	2.3441038000000001
H	1.4689878999999999	-5.6471534999999999	0.86284322999999996
O	4.3611801000000003	-5.7722125000000002	1.1059162000000000
H	3.8778503999999998	-6.4059743999999998	1.5856363000000000
H	3.5675905999999999	-5.4064876999999996	0.69741109999999995
O	4.0309245000000002	-5.3141990999999997	4.2847879000000004
H	4.1232819000000003	-4.5437273999999999	4.9059027000000004
H	3.3136036000000000	-4.8941844999999997	3.6986150000000002
O	-0.84345141000000001	-4.5422368000000004	4.1216704000000002
H	0.14414803000000001	-4.5137223000000004	3.9368470000000002
H	-1.1423342999999999	-5.0838865999999996	3.3802805000000000
O	-1.0186499000000000	-4.7041019999999998	-0.59231164999999997
H	-0.16018046999999999	-4.5509380000000004	-0.24435602000000001
H	-1.4592536000000000	-4.9351948999999999	0.27758896999999999
O	1.8835957000000001	-3.3072783000000001	-2.2713567000000001
H	1.4755275000000001	-4.1832975000000001	-2.6389014000000000
H	1.8495352000000000	-3.4904237999999999	-1.3083678000000001
O	-1.3017584000000000	-6.2454454000000004	1.8190065000000000
H	-0.42677254999999997	-6.5462994999999999	1.7874435000000000

H	-1.7104189000000001	-7.0571834000000004	2.2650163000000001
O	5.0086550000000001	-2.1726860000000001	0.79017488999999996
H	5.0858014000000002	-2.8675206000000002	1.4357572999999999
H	5.0638679000000000	-1.4025760000000000	1.4011613000000001
O	1.9016933000000000	-4.0838821999999997	6.4897166000000004
H	1.6165233000000001	-5.0128924000000001	6.6820494000000004
H	1.8911270000000000	-4.0616887999999998	5.5102905000000000
O	-0.95268900000000001	-1.8168154000000001	4.1757460000000002
H	-1.1131161999999999	-2.7700694000000001	3.9197660000000001
H	-2.5388924000000002E-003	-1.8073413000000000	4.3323121999999996
O	1.3182343999999999	-8.7127847999999997	2.4115058000000000
H	1.6375587000000000	-7.9493825999999999	1.9553471000000000
H	2.1105255999999999	-9.2276197999999994	2.5580471999999999
O	4.2581638000000002	-2.6832609999999999	5.4600499999999998
H	3.4010395999999998	-2.3401622999999998	5.0966956000000003
H	4.0189959000000002	-2.8616494000000001	6.3847446999999997

C	0.18376995E+01	-0.31244370E+01	0.24873294E+01
C	0.19137574E+01	-0.18104579E+01	0.30616964E+01
O	0.20461331E+01	-0.84161239E+00	0.20888809E+01
C	0.18942010E+01	-0.14712227E+01	0.77298847E+00
C	0.18622574E+01	-0.29461945E+01	0.10970008E+01
O	0.17709279E+01	-0.13937619E+01	0.42126700E+01
C	0.56081707E+00	-0.89530960E+00	0.21533416E+00
O	-0.53893223E+00	-0.12679537E+01	0.10042009E+01
O	1.7538975999999999	-3.8092766000000000	0.10791096999999999
O	1.6613789999999999	-4.2378178999999996	3.2282006000000001
C	0.55231771E+00	0.58970849E+00	0.80292562E-01
O	-0.75211469E+00	0.10884975E+01	-0.31820307E+00
H	0.26800095E+01	-0.11526258E+01	-0.30955298E-02
H	0.80569316E+00	0.10389390E+01	0.10515172E+01
H	0.13136422E+01	0.86770182E+00	-0.65825373E+00
H	-0.14142582E+01	0.91616375E+00	0.42003396E+00
H	0.47358832E+00	-0.14112043E+01	-0.74277409E+00
H	-0.45449835E+00	-0.11529329E+01	0.19758217E+01
O	1.3039972000000000	-6.2259772000000000	1.2149178000000000
H	1.5163542999999999	-5.6307372999999998	1.9831365999999999
H	1.3898862000000001	-5.5312846000000002	0.55956669999999997
O	4.2737143000000000	-5.2734516999999999	0.99423804999999998
H	3.7718907000000002	-5.9754231999999998	1.3209795000000000
H	3.6387706999999998	-4.6909368999999996	0.59150530999999995
O	-0.79073194000000002	-4.7332646000000000	-0.55029240999999995
H	7.7430698000000006E-002	-4.4274151000000002	-0.27258476999999998
H	-1.0281830999999999	-5.2301538000000001	0.26371150999999998
O	-0.99942863999999998	-4.6148511000000001	4.0131698000000000
H	-8.7097292000000007E-002	-4.3818399000000001	3.8008910000000000
H	-1.1814559000000000	-5.2573183999999999	3.3175252000000000
O	3.9564528999999999	-5.2487218000000002	4.3997380000000001
H	4.2658044999999998	-4.3771563000000002	4.7062511000000002
H	3.1137982000000002	-5.1205905999999999	3.8955898000000002
O	1.9266065999999999	-3.5037937000000001	-2.4016047999999999
H	1.4122155999999999	-4.3442381000000001	-2.4137464000000000
H	1.9968048000000000	-3.4259970000000002	-1.4041215000000000
O	2.5592226000000000	-6.5591818000000002	-1.3809501000000000
H	2.1251978000000000	-6.3318593999999999	-0.51476898000000004
H	1.7297429000000000	-6.4229801000000002	-1.9813012000000001
O	-1.3855409999999999	-6.2849588000000001	1.7221508999999999
H	-0.50769133000000000	-6.6856859999999996	1.5600814999999999
H	-1.8422962000000001	-7.1135720999999998	2.0176878000000000
O	1.7378127000000001	-3.9147612999999999	6.3472930999999999
H	1.9867897999999999	-4.7251902000000001	6.7691036000000002
H	1.9158767999999999	-4.1138871000000004	5.4015715999999996
O	6.1844561000000002	-4.0129523999999996	2.5594092000000002
H	5.4840742999999996	-4.5460295999999998	2.0638030000000001
H	6.1305636000000003	-4.4038478000000003	3.4437410000000002
O	1.4529231000000000	-8.6936251999999996	2.6798947000000002
H	1.5317818000000001	-7.8587850000000001	2.1395786999999999
H	2.3459962999999999	-8.7808282000000002	3.0990229000000000
O	4.2555674999999997	-2.4593870999999998	5.2368284000000003
H	3.3634848000000002	-2.3670865999999999	4.8871244000000003
H	4.1550661000000000	-2.1028231000000002	6.1507367000000004

C	0.18849973E+01	-0.31266583E+01	0.26027870E+01
C	0.21067717E+01	-0.17878803E+01	0.31381179E+01
O	0.22179083E+01	-0.76129564E+00	0.21326528E+01
C	0.19927574E+01	-0.15007665E+01	0.84275431E+00
C	0.19413981E+01	-0.29579348E+01	0.12286550E+01
O	0.21197456E+01	-0.14492124E+01	0.42952678E+01
C	0.69644683E+00	-0.99323129E+00	0.29084299E+00
O	-0.43380498E+00	-0.13782493E+01	0.10766495E+01
O	1.9209162000000000	-3.8433218000000000	0.31163146000000003
O	1.8143966000000000	-4.2150157999999998	3.3053197000000001
C	0.64250825E+00	0.54772258E+00	0.35800261E-01
O	-0.62952699E+00	0.96511942E+00	-0.52454047E+00
H	0.27922075E+01	-0.11633513E+01	0.24026814E+00
H	0.83010525E+00	0.10812296E+01	0.10155683E+01
H	0.14732527E+01	0.78823043E+00	-0.65019383E+00
H	-0.12075637E+01	0.12590655E+01	0.25470132E+00
H	0.46250176E+00	-0.14886926E+01	-0.66093572E+00
H	-0.24693170E+00	-0.11735759E+01	0.20567078E+01
O	1.5965623000000000	-6.2611578000000003	1.4710847000000000
H	1.3760136999999999	-6.0635697000000004	2.4358711999999998
H	1.6178233000000000	-5.3308476000000002	1.1009245999999999
O	4.2304668000000003	-5.6443227000000000	1.0172601999999999
H	3.5048992000000001	-6.1137378000000000	1.4750998000000000
H	3.6554509999999998	-4.8584709000000004	0.77172308999999994
O	-0.61959213999999996	-4.9316357000000002	-0.44153734000000000
H	0.20627460000000000	-4.5222198000000002	-0.14048081000000001
H	-0.84985047000000002	-5.3855890000000004	0.35291618000000002
O	3.9144467000000001	-5.4620002999999997	4.3932729000000004
H	4.1859089000000003	-4.5775769000000004	4.7463857000000003
H	3.0247226999999999	-5.1800224999999998	4.0524202000000002
O	-0.85675323999999997	-4.7872918999999996	4.0703101000000004
H	0.12918653999999999	-4.7878448999999996	3.9156976999999999
H	-1.1696987999999999	-5.2999855000000000	3.3304288999999998
O	1.7878543000000000	-3.4872570999999999	-2.3374416999999998
H	1.5423473999999999	-4.4043961999999999	-2.4688382000000000
H	1.8766883000000001	-3.6084798000000000	-1.3437778000000000
O	1.7180572999999999	-3.8566406000000000	6.2499237000000001
H	1.8146154999999999	-4.7908141000000004	6.6429723000000003
H	1.7821400999999999	-4.0287584000000001	5.2700467000000000
O	5.9111786999999998	-3.9369215999999998	2.5381648999999999
H	5.3960701000000002	-4.5644587000000003	1.9860399000000000
H	6.2027200000000002	-4.4974324000000001	3.2861096000000001
O	2.3583715999999999	-6.8015160000000003	-1.6406050999999999
H	2.2260620000000002	-6.6076689999999996	-0.70709807999999996
H	1.4396773000000000	-6.7151334000000000	-1.9931601000000001
O	-0.78499322000000005	-2.0164053000000002	4.4016843000000003
H	-0.93225230999999997	-2.9449540999999999	4.0639365999999999
H	-2.1075696000000001E-002	-2.0046867000000002	4.9861000000000004
O	1.5158357000000000	-8.6546512999999994	2.8374560999999998
H	1.5564258000000000	-7.8927304999999999	2.2063149000000002
H	2.4725443000000000	-8.8308324999999996	3.0283121999999998
O	4.1391270999999996	-7.8057857999999998	3.3421322000000000
H	3.9674589000000000	-6.8596735000000004	3.6707874000000000
H	5.1046646999999998	-7.8250089999999997	3.4524284999999999

C	0.17778320E+01	-0.29799901E+01	0.25774516E+01
C	0.19140873E+01	-0.16901018E+01	0.32218179E+01
O	0.21506295E+01	-0.69243550E+00	0.21750956E+01
C	0.19593282E+01	-0.13509738E+01	0.81283132E+00
C	0.19702065E+01	-0.28203902E+01	0.11878717E+01
O	0.20955142E+01	-0.14020376E+01	0.43881743E+01
C	0.60780192E+00	-0.95014263E+00	0.24618305E+00
O	-0.41475622E+00	-0.11029402E+01	0.11692500E+01
O	2.0500555999999999	-3.8311066000000000	0.29960006000000000
O	1.7877023000000001	-4.1448594999999999	3.2866829000000002
C	0.61718239E+00	0.57780299E+00	-0.11874128E+00
O	-0.73192403E+00	0.90910528E+00	-0.59909790E+00
H	0.27343401E+01	-0.10533610E+01	0.12370927E+00
H	0.97904707E+00	0.12620795E+01	0.62250722E+00
H	0.13344884E+01	0.68563130E+00	-0.98557532E+00
H	-0.12247871E+01	0.12874996E+01	0.16426232E+00
H	0.44706099E+00	-0.15688951E+01	-0.64117958E+00
H	-0.22555599E+00	-0.18437782E+01	0.17369620E+01
O	1.4384950000000001	-6.2893182999999997	1.2409363000000000
H	1.4325551000000001	-5.9676859999999996	2.1254786000000001
H	1.6445434999999999	-5.4871461000000004	0.75419066999999995
O	4.3338698999999998	-5.3960239999999997	0.96992005000000003
H	3.8415105000000001	-6.0024858999999999	1.5366740999999999
H	3.6080518000000001	-4.9290101000000002	0.53772677000000002
O	-0.41845205000000002	-4.8287882000000000	-0.33381094000000000

H	0.39456424000000001	-4.3495417999999999	6.1612979999999998E-002
H	-0.79940146999999995	-5.3669864000000000	0.42797395999999999
O	-0.62120180999999997	-5.1129078999999997	3.9509873000000000
H	0.29350205000000001	-4.8708210999999997	3.6638639000000000
H	-0.76422427999999998	-5.4718457999999996	3.0732159999999999
O	4.1529962999999999	-5.3071850999999999	4.5611588000000003
H	4.5500955999999997	-4.5386167999999998	4.9775201999999998
H	3.4190820000000000	-4.9668058000000004	4.0007124999999997
O	2.0821947000000001	-3.3208833000000002	-2.2780317000000001
H	1.4092366999999999	-3.9619559999999998	-2.5762434000000001
H	2.1194383000000001	-3.5448211999999999	-1.2839153000000001
O	1.6357835000000001	-3.9974864000000001	6.0055272999999998
H	2.0431382000000000	-4.8042350999999996	6.3627887000000003
H	1.6802964000000000	-4.1490799999999997	4.9959764000000000
O	5.8959069000000000	-3.6918164999999998	2.4087222000000001
H	5.4485865000000002	-4.2769513999999997	1.7996079000000000
H	6.3451943999999996	-4.2934957999999996	2.9908104999999998
O	-0.79116861000000005	-2.2875553000000002	4.7333470000000002
H	-0.73900953999999996	-3.1386278999999999	4.3472349000000001
H	0.17276908999999999	-2.1827082000000000	4.8323001000000003
O	4.2028730999999997	-7.7080602000000003	3.1341293000000001
H	4.1598585000000003	-6.8119399999999999	3.5925449000000000
H	5.0911441999999996	-7.9926905000000001	3.3999486000000001
O	5.1518366000000002	-1.6981238000000001	0.50834811000000002
H	5.5536124999999998	-2.3899479000000001	1.0234369999999999
H	5.0439973000000000	-0.95534120000000000	1.1065495999999999
O	2.1062256000000001	-7.4973125999999999	-1.6060905999999999
H	1.5619654999999999	-7.1151485000000001	-0.87763860999999999
H	1.7939072000000000	-6.9077061000000004	-2.3192119999999998

C	0.19784485E+01	-0.30599644E+01	0.25823416E+01
C	0.19661840E+01	-0.17926298E+01	0.32260330E+01
O	0.18099115E+01	-0.81659943E+00	0.22375835E+01
C	0.18462337E+01	-0.13853940E+01	0.92748060E+00
C	0.19792006E+01	-0.28933122E+01	0.12145260E+01
O	0.19884045E+01	-0.14239969E+01	0.44150748E+01
C	0.57163423E+00	-0.93430278E+00	0.13117802E+00
O	-0.61866861E+00	-0.13082556E+01	0.89504733E+00
O	2.0808447999999999	-3.8328928000000002	0.31058097000000001
O	1.9964071999999999	-4.1990942000000002	3.2950241000000000
C	0.46833873E+00	0.57336063E+00	0.87326659E-01
O	-0.73190660E+00	0.10291057E+01	-0.57374634E+00
H	0.27062826E+01	-0.10050495E+01	0.37241589E+00
H	0.51493645E+00	0.10908921E+01	0.10421975E+01
H	0.11513559E+01	0.10686357E+01	-0.55799499E+00
H	-0.13571733E+01	0.12187998E+01	0.17553717E+00
H	0.45179523E+00	-0.13811926E+01	-0.86832495E+00
H	-0.35771875E+00	-0.20133589E+01	0.15896013E+01
O	1.4143611000000000	-6.3046052000000001	1.7611030999999999
H	1.6344997999999999	-5.8203975000000003	2.6041241999999998
H	1.5456591000000000	-5.5631987000000001	1.1509685999999999
O	4.3435186000000003	-5.3681238999999996	0.92414204000000000
H	3.8675814000000002	-5.6433850000000003	1.7553729000000000
H	3.6519202000000002	-4.8617600999999997	0.48260626000000001
O	-0.68895355000000003	-5.0096635999999998	3.7473036999999998
H	0.27037793999999998	-4.9883870999999997	3.5916782000000000
H	-0.92915614000000002	-5.4266220000000001	2.8658896000000000
O	-0.59059877999999999	-4.6496262000000002	-0.42003691999999998
H	0.10843915000000000	-4.1318809999999999	-8.7743507999999998E-002
H	-0.93001243000000000	-5.0555475999999997	0.42037382000000001
O	4.0944718000000000	-5.3999470000000001	4.5696545999999998
H	4.4380724000000003	-4.5971624999999996	5.0566804000000003
H	3.3928759999999998	-4.8674679999999997	3.9924648000000000
O	2.1037794000000001	-3.7822263999999999	-2.3283692999999999
H	1.6333972999999999	-4.6111618999999999	-2.7189461000000001
H	2.2253139000000002	-4.0341246999999996	-1.4222147999999999
O	1.3895238000000001	-4.0936583000000004	6.0433465000000002
H	1.6124058999999999	-5.0416201999999997	5.8318851000000000
H	1.4380782999999999	-3.7848769999999998	5.0860573999999996
O	5.0724548000000000	-1.7222489000000001	0.53649396999999999
H	5.1591782999999998	-2.5170902000000002	1.0386943000000000
H	4.9737492000000003	-1.0814638999999999	1.2353518000000001
O	5.8078579000000001	-3.6314972999999999	2.4585180000000002
H	5.6204466999999996	-4.3675287000000003	1.8346098000000000
H	6.3609887000000001	-4.1552211000000003	3.1127495000000001
O	2.5422853000000001	-7.1778640999999999	-1.5550337000000001
H	2.3342217999999999	-6.8204507000000003	-0.65917643000000004
H	1.7232235000000000	-7.0760078999999996	-2.0068514000000000
O	-0.65692159999999999	-2.3069063000000001	4.7010835000000002
H	-0.73793196000000005	-3.1663285999999999	4.2630575999999998
H	0.31074813000000001	-2.2238001999999999	4.6640793000000000
O	4.1239629999999998	-2.8152181999999999	5.5931176000000002
H	3.3641065000000001	-2.4350255999999999	5.1622807999999996
H	3.9856807999999999	-2.3907075999999998	6.4279862000000003

C	0.19524672E+01	-0.30067442E+01	0.26026166E+01
C	0.20091190E+01	-0.17152189E+01	0.31266408E+01
O	0.19467391E+01	-0.76563869E+00	0.21034360E+01
C	0.16825424E+01	-0.14489877E+01	0.88018457E+00
C	0.17597645E+01	-0.28830590E+01	0.12024833E+01
O	0.20955333E+01	-0.12454968E+01	0.43206392E+01
C	0.25306435E+00	-0.10812428E+01	0.32683048E+00
O	-0.71517894E+00	-0.15092775E+01	0.13120642E+01
O	1.7044030999999999	-3.8303265000000000	0.34344054000000002
O	1.9158824999999999	-4.1027946999999996	3.3214636000000000
C	0.26467064E+00	0.44547754E+00	0.15032379E+00
O	-0.91570912E+00	0.98072130E+00	-0.52199885E+00
H	0.24023398E+01	-0.10796232E+01	0.12480821E+00
H	0.34165597E+00	0.93243690E+00	0.11307589E+01
H	0.11489340E+01	0.68247227E+00	-0.45581294E+00
H	-0.13679037E+01	0.16559314E+01	0.27935995E-01
H	0.78343667E-01	-0.15969637E+01	-0.63884900E+00
H	-0.25713310E+00	-0.22117071E+01	0.17774756E+01
O	1.6796234000000001	-6.2473879999999999	1.5919964000000000
H	1.7492464000000001	-5.7441506000000002	2.4214739999999999
H	1.4665467999999999	-5.4036729000000001	1.1226448000000000
O	-0.71758438000000002	-4.4263548999999998	3.4933822999999999
H	0.27305858000000000	-4.3791225000000003	3.3844211000000000
H	-0.88533994999999999	-5.0972885999999997	2.8356229000000002
O	-0.55050473000000000	-4.8954469999999999	-0.52537792000000005
H	0.31543584000000002	-4.5296402999999996	-0.31417949000000001
H	-0.77850459000000005	-5.3238424000000002	0.33044994999999999
O	4.5551557000000003	-5.2353006999999998	0.88318823000000002
H	3.8325665999999998	-5.7117601000000002	1.2764488000000001
H	4.0763784000000003	-4.6068864999999999	0.35966724000000000
O	4.2887744000000003	-5.2320665000000002	4.2326552000000000
H	4.3317085999999998	-4.4536178000000000	4.7966432000000001
H	3.4393351999999999	-5.0658345999999996	3.7611509999999999
O	2.1126581999999998	-3.6007237000000001	-2.2825004000000000
H	1.5444012000000000	-4.3516500999999996	-2.5017083000000002
H	2.0023198999999998	-3.5874761999999998	-1.2995753000000001
O	1.1119872000000000	-4.1253909000000002	5.9572406000000004
H	1.7276256000000001	-4.6989089999999996	6.3828383999999998
H	1.5852461000000000	-4.1349463000000002	5.0718427999999998
O	-0.86653723999999999	-1.9708250000000000	4.5438872999999997
H	-0.94913289000000001	-2.8707699999999998	4.2237534999999999
H	0.10278915000000000	-1.9358200000000001	4.7105269999999999
O	1.2906882000000000	-8.6063986999999997	2.8009789000000000
H	1.4519637000000001	-7.7410413000000000	2.3522170999999998
H	2.1822134000000002	-8.9629606000000006	2.9316228999999998
O	6.3478956999999996	-3.8515385000000002	2.2860166999999998
H	5.7526919999999997	-4.5543952000000001	1.9326928999999999
H	6.9171259000000003	-4.3843521000000001	2.9089567000000001
O	-1.5034618000000000	-6.3751933999999997	1.6705738000000001
H	-1.2134598999999999	-7.1925197000000001	1.1566962999999999
H	-2.2970695999999999	-6.7570990999999996	2.1541313000000000
O	5.5809617999999999	-1.8781981999999999	0.53458580000000000
H	5.7105303000000003	-2.6586216999999999	1.0568957999999999
H	5.7675789999999996	-1.1762052000000001	1.1698705000000000

C	0.20488391E+01	-0.30105812E+01	0.25452406E+01
C	0.18711730E+01	-0.16873244E+01	0.31063182E+01
O	0.18689546E+01	-0.71646194E+00	0.21186544E+01
C	0.17272650E+01	-0.13648316E+01	0.88115431E+00
C	0.19257920E+01	-0.28449966E+01	0.11052007E+01
O	0.17291290E+01	-0.13910113E+01	0.42865796E+01
C	0.37936022E+00	-0.10758965E+01	0.29228315E+00
O	-0.70322582E+00	-0.16007716E+01	0.10323570E+01
O	1.8705995000000000	-3.6815178000000000	0.16863288000000001
O	1.9229434999999999	-4.1413020999999999	3.1942792000000000
C	0.10815830E+00	0.41203510E+00	-0.23802270E-01
O	-0.12484575E+01	0.60208890E+00	-0.51510830E+00

H	0.24942751E+01	-0.89651411E+00	0.28314972E+00
H	0.25829266E+00	0.98315040E+00	0.90285515E+00
H	0.79238980E+00	0.71818822E+00	-0.83595961E+00
H	-0.17324011E+01	0.64671069E+00	0.33233247E+00
H	0.30376280E+00	-0.14609537E+01	-0.77034050E+00
H	-0.55652115E+00	-0.18359878E+01	0.19670847E+01
O	1.7869428000000001	-6.1175392999999998	1.4206858000000000
H	1.7676706000000000	-5.5809432000000001	2.2941028000000001
H	1.7125652000000000	-5.3696541000000000	0.80551971000000000
O	4.3160211000000004	-5.2820755999999998	0.78338951999999995
H	3.7994835999999998	-5.9630141999999999	1.2921440000000000
H	3.6925889000000001	-4.6038474000000003	0.63655567000000002
O	-0.77420336000000001	-4.3498625999999998	3.4161698999999999
H	0.21222041999999999	-4.3893436000000001	3.2626769000000002
H	-1.0809169000000001	-4.9963476000000000	2.6911097000000002
O	-0.64315193000000004	-4.8837535000000001	-0.62174757000000003
H	0.16110030000000000	-4.3493943000000002	-0.34285138999999998
H	-0.82417916999999996	-5.3445286999999997	0.16753024999999999
O	4.1093191999999998	-5.3154187000000004	4.4492194999999999
H	4.4168848000000001	-4.5213057000000001	4.9722451000000003
H	3.4357617999999999	-4.9541250999999997	3.8337813000000001
O	1.4816604000000000	-4.1201854000000004	5.9173458999999999
H	1.9082020000000000	-4.9726439999999998	5.8966079000000002
H	1.4360253999999999	-4.0186038999999996	5.0168843000000001
O	1.6567156999999999	-3.5895492000000000	-2.6347361000000000
H	1.9717914999999999	-4.4483752000000001	-2.8401692999999999
H	1.6241889000000000	-3.5391211000000000	-1.6683938000000000
O	4.5490101000000003	-2.7507074999999999	-1.8728024999999999
H	3.7223036999999999	-3.1479678999999998	-1.4805816000000001
H	4.8100348999999998	-2.0501974000000001	-1.2222227000000001
O	6.4132994999999999	-3.8288972999999999	1.9529862000000000
H	5.6932543999999998	-4.3310411000000002	1.5016986999999999
H	6.5306831000000001	-4.3767896999999998	2.7641878000000002
O	1.4243163000000001	-8.7726924000000004	2.3814229000000000
H	1.6078725000000000	-7.8153652999999998	2.3140562999999998
H	2.3621911999999998	-9.1219304999999995	2.2843282000000000
O	2.7813843000000000	-7.1630317000000003	-1.7111095999999999
H	2.7659373999999999	-7.0809163000000002	-0.75826581000000004
H	1.8189789999999999	-7.2148370999999996	-1.8771876999999999
O	-0.93356439999999996	-1.8924835000000000	4.8410827000000003
H	-0.95113365999999999	-2.7539720000000001	4.3921663999999998
H	-0.10347495000000000	-1.4343543999999999	4.6641294999999996

C	0.20479927E+01	-0.31355496E+01	0.23186531E+01
C	0.20488609E+01	-0.18195053E+01	0.29371629E+01
O	0.19293460E+01	-0.79432846E+00	0.20156676E+01
C	0.17300634E+01	-0.14374759E+01	0.65523858E+00
C	0.19976889E+01	-0.28925511E+01	0.96799978E+00
O	0.21738854E+01	-0.14197811E+01	0.41235608E+01
C	0.34602389E+00	-0.10566862E+01	0.18779091E+00
O	-0.66471531E+00	-0.15435868E+01	0.10908083E+01
O	1.9984047000000000	-3.8213271999999998	1.7057847000000001E-002
O	2.0477292000000000	-4.2851657999999997	3.0551693000000002
C	0.23607047E+00	0.47668498E+00	-0.96036346E-01
O	-0.11063319E+01	0.83150606E+00	-0.45390849E+00
H	0.25573345E+01	-0.99156345E+00	0.80314533E-01
H	0.62938362E+00	0.10520768E+01	0.78536632E+00
H	0.92067660E+00	0.71923694E+00	-0.87336839E+00
H	-0.15942524E+01	0.11794949E+01	0.32820257E+00
H	0.26657782E-01	-0.15937119E+01	-0.71840823E+00
H	-0.48868290E+00	-0.13859554E+01	0.20199805E+01
O	1.6549381999999999	-6.2335390000000004	1.0514931000000001
H	1.6455544000000000	-5.8091108000000000	1.9456389000000001
H	1.6451229999999999	-5.3951171000000002	0.55679957999999996
O	4.1924196000000000	-5.4308182000000000	0.38008773000000001
H	3.5537372999999999	-5.9781557999999997	0.89812619000000005
H	3.5281962000000000	-4.7307326999999999	6.4036077999999996E-002
O	-0.90214565000000002	-4.9123628000000004	-0.29811472000000000
H	2.2338859999999999E-002	-4.7912455999999999	1.9280155000000000E-002
H	-1.1300047000000000	-5.5617634999999996	0.41037267999999999
O	-0.69221204999999997	-4.4531942999999998	3.7296969000000000
H	0.28432731999999999	-4.4541792999999998	3.6041080000000001
H	-0.85154405000000000	-5.1354591000000003	3.0314733999999999
O	4.0272921000000004	-5.3170191999999998	4.4245631000000003
H	4.3907062999999997	-4.4722505999999997	4.6919813000000001
H	3.2727301999999998	-4.9280118999999996	3.8846712000000001

O	1.3011874999999999	-3.5295347000000001	-2.5962393000000001
H	1.7975482000000000	-4.2291859000000001	-3.0205858000000001
H	1.5844410000000000	-3.7011569000000000	-1.6694061000000000
O	4.3691104000000003	-2.8931613000000000	-1.7959001000000001
H	3.4843975000000000	-2.9663963999999998	-1.3797216999999999
H	4.7996477999999998	-2.3088337000000001	-1.0492473000000000
O	1.5760399000000000	-4.0254234000000002	5.9728352999999998
H	2.2787513000000001	-4.6738423999999998	6.0120706999999998
H	1.7010825000000001	-3.6779329000000001	5.0528145000000002
O	6.2392092999999997	-3.9410512999999998	1.5068094000000001
H	5.5720659000000001	-4.6448342000000000	1.2460667000000001
H	7.0495624000000001	-4.4729934000000000	1.6454196000000001
O	1.2562875000000000	-8.5944725000000002	2.6137616000000001
H	1.3321700000000001	-7.6749710000000002	2.3076607999999998
H	2.2092459999999998	-8.8347981000000004	2.4956250000000000
O	5.0568692999999998	-1.7241560000000000	0.53164104999999995
H	5.5473179999999997	-2.4183346000000001	1.0344590000000000
H	5.1757353000000004	-1.0274639000000001	1.1519210000000000
O	4.3466952000000001	-2.7286738000000001	5.3124082000000001
H	3.5221358999999999	-2.4281337999999999	4.8377189999999999
H	4.2263261999999999	-2.2894630999999999	6.1643638000000003

C	0.17716282E+01	-0.31085317E+01	0.25080918E+01
C	0.19673800E+01	-0.17641130E+01	0.30062814E+01
O	0.18598245E+01	-0.78695702E+00	0.19076187E+01
C	0.16728326E+01	-0.15563914E+01	0.68430569E+00
C	0.17093468E+01	-0.29622094E+01	0.10958747E+01
O	0.21217331E+01	-0.13374511E+01	0.41439634E+01
C	0.24588834E+00	-0.10676955E+01	0.21858956E-01
O	-0.86760964E+00	-0.14634482E+01	0.89666156E+00
O	1.7410340000000000	-3.9669373999999999	0.25943089000000003
O	1.9105814000000001	-4.1891641000000002	3.2549427000000000
C	0.14172970E+00	0.44028593E+00	-0.97991209E-01
O	-0.11403454E+01	0.88788811E+00	-0.48458678E+00
H	0.25144845E+01	-0.13886641E+01	-0.96164742E-01
H	0.34693468E+00	0.76525488E+00	0.96670554E+00
H	0.81978531E+00	0.88179726E+00	-0.82613949E+00
H	-0.17262210E+01	0.10669025E+01	0.27851241E+00
H	0.19596107E+00	-0.16306462E+01	-0.88384067E+00
H	-0.49810241E+00	-0.17530108E+01	0.17658723E+01
O	1.3309002999999999	-6.3790623999999996	1.7124831000000000
H	1.5444027000000000	-5.5410104000000002	2.2117385999999999
H	1.6604722999999999	-5.9973817000000000	0.87498014000000002
O	4.1350027999999996	-5.3129803999999998	0.53252555999999995
H	3.7689126000000002	-5.4210215000000002	1.4061802000000001
H	3.4055537000000000	-4.8018327000000003	0.20047867000000000
O	-0.73856330999999997	-4.5760731999999997	3.7759882999999999
H	0.28195093999999998	-4.5770210000000002	3.7798604000000000
H	-0.96298371999999999	-5.2290546999999998	3.0638896999999998
O	-0.69728579000000002	-5.1599645000000001	-0.38671501000000003
H	-2.7839645999999999E-002	-4.5896631000000001	-4.3057175999999999E-005
H	-1.0534673999999999	-5.5224886000000000	0.40943695000000002
O	3.9463433000000001	-5.4419740000000001	4.6768758000000004
H	4.4187352999999998	-4.5928721000000001	4.6566565000000004
H	3.1165818999999999	-5.0958322999999996	4.2428783000000001
O	1.7062359000000000	-3.7651645999999999	5.8646665999999996
H	2.1548729999999998	-4.6236272999999999	5.9898137000000000
H	1.9036276000000001	-3.6528387000000002	4.8610813000000004
O	1.4030739000000001	-3.4177209000000000	-2.4740213000000000
H	1.6219242000000000	-4.3082297000000001	-2.8080297999999999
H	1.7718902000000001	-3.5404732000000001	-1.6288492000000001
O	4.4840821999999996	-2.6607354000000001	-1.5109319999999999
H	3.8337564000000000	-3.1951801000000000	-1.0628393000000000
H	4.8709959999999999	-2.1981234000000001	-0.72922527999999998
O	-1.2786225000000000	-6.6029415000000000	1.7542849000000000
H	-0.33781576000000002	-6.9457050999999996	1.7930069000000000
H	-1.8349553999999999	-7.3717381000000000	2.0506877000000001
O	-0.81522095000000006	-2.0633599000000000	4.4915191999999999
H	-1.2189565000000000	-2.9207470000000000	4.2209776000000003
H	0.10600479000000000	-2.3207719000000000	4.6883603999999997
O	1.2499994000000001	-8.8309286999999994	2.6817139999999999
H	1.2013342000000000	-7.8701349000000000	2.5235476999999999
H	2.1072079000000001	-9.0793847000000003	2.2141191999999998
O	4.4494549000000001	-2.7999773000000001	5.3107286000000000
H	3.5551629999999999	-2.5461732000000001	5.0634986000000000
H	4.5760857000000001	-2.3163333000000002	6.1265372999999999

C	0.19516266E+01	-0.30794290E+01	0.24351959E+01
C	0.20563588E+01	-0.17367568E+01	0.29260739E+01
O	0.20509963E+01	-0.79597002E+00	0.18869772E+01
C	0.17344035E+01	-0.15318596E+01	0.64071733E+00
C	0.18767828E+01	-0.29886294E+01	0.10146918E+01
O	0.20882927E+01	-0.13188189E+01	0.40806063E+01
C	0.27144694E+00	-0.12358737E+01	0.19267626E+00
O	-0.70832582E+00	-0.13554264E+01	0.13061203E+01
O	1.8916143999999999	-3.9128991000000002	0.13201294999999999
O	1.9418023000000000	-4.1963327000000001	3.1594299000000001
C	0.25420180E+00	0.26570556E+00	-0.22632572E+00
O	-0.10349340E+01	0.73601557E+00	-0.56937772E+00
H	0.23578728E+01	-0.11007184E+01	-0.97656675E-01
H	0.60836426E+00	0.79775386E+00	0.72139290E+00
H	0.83443342E+00	0.39329693E+00	-0.11323751E+01
H	-0.15233491E+01	0.10058546E+01	0.18209361E+00
H	0.54475260E-02	-0.19703692E+01	-0.59391682E+00
H	-0.19345351E+00	-0.17270159E+01	0.20519644E+01
O	1.7946215999999999	-6.2941259000000001	1.5432098999999999
H	1.9696199999999999	-5.6928785000000000	2.2442223000000001
H	1.7583705000000001	-5.5593165999999998	0.87520209000000004
O	4.2094434999999999	-5.4535467999999998	0.66373554000000001
H	3.5927506999999999	-6.0924867999999996	1.1212626000000001
H	3.5096631000000000	-4.8462772999999997	0.33625839000000002
O	-0.56122402000000005	-4.8819393000000000	-0.27976209000000002
H	0.35457077999999997	-4.6092931999999998	-5.5147351999999997E-002
H	-0.90982118000000001	-5.3356032999999998	0.51648987999999996
O	-0.57131536999999999	-4.6511149999999999	3.9412254000000000
H	0.41612669000000002	-4.5043914000000003	3.7516200999999998
H	-0.91421269999999999	-4.9945234999999997	3.0575993000000001
O	4.2214555000000002	-5.3875738999999996	4.2708190999999998
H	4.6660507999999998	-4.6640550999999997	4.7622378999999997
H	3.4478770999999999	-4.8467139000000001	3.9905119999999998
O	1.5443781000000001	-4.0785428000000001	6.0157359000000001
H	1.8789126000000000	-4.9536831000000001	6.3035852999999999
H	1.7774669999999999	-4.0701166000000004	5.0351486999999997
O	1.0693382000000000	-3.5074074000000000	-2.6018089000000000
H	1.5289476000000000	-4.1504143999999998	-3.1512698000000001
H	1.4798707000000000	-3.6289364000000002	-1.6948120000000000
O	4.4268422999999997	-2.8647898999999999	-1.8061275000000001
H	3.7425231999999999	-3.2749592999999999	-1.2718647999999999
H	4.8668100000000001	-2.2645114999999998	-1.0851786999999999
O	6.2698055999999998	-3.9410322999999998	1.7501960999999999
H	5.5294509999999999	-4.5328776000000000	1.3732005000000000
H	6.9568973999999999	-4.5532940999999996	1.9028590999999999
O	1.2319722000000000	-8.5602081999999999	3.0732205000000001
H	1.3583158000000000	-7.7733357999999999	2.4678882999999998
H	2.1711581000000000	-8.6718933000000007	3.1963929000000002
O	-1.1524605999999999	-6.5714661999999997	1.8822875999999999
H	-0.61300849000000002	-7.0529647999999998	1.2181713999999999
H	-1.7504245000000000	-7.2701064999999998	2.1348139000000002
O	-0.66495490999999995	-2.1330908000000002	4.7890230000000003
H	-0.92340312000000002	-3.0506576000000001	4.5840727000000001
H	0.26253240999999999	-2.2225088999999998	4.9378479000000004

C	0.20322237E+01	-0.30046114E+01	0.25833042E+01
C	0.20875884E+01	-0.17096825E+01	0.30580536E+01
O	0.19235738E+01	-0.71199813E+00	0.20453529E+01
C	0.17575753E+01	-0.14405476E+01	0.82618336E+00
C	0.19202197E+01	-0.29199039E+01	0.12139579E+01
O	0.21518361E+01	-0.12666084E+01	0.42196149E+01
C	0.47298234E+00	-0.11573362E+01	0.23408638E+00
O	-0.69778149E+00	-0.14901265E+01	0.10304734E+01
O	1.9447703000000001	-3.7672891000000002	0.24010504999999999
O	1.8448277000000000	-4.0965087999999996	3.3714430000000002
C	0.27167036E+00	0.32948719E+00	-0.29988558E+00
O	-0.10553924E+01	0.46064781E+00	-0.70973213E+00
H	0.26180283E+01	-0.10865308E+01	0.23739624E+00
H	0.52114952E+00	0.10341117E+01	0.46868571E+00
H	0.98229376E+00	0.49620361E+00	-0.11827636E+01
H	-0.15634521E+01	0.69857932E+00	0.70092811E-01
H	0.43747551E+00	-0.18218374E+01	-0.56578688E+00
H	-0.40986670E+00	-0.17123263E+01	0.19544338E+01
O	1.9310008000000001	-6.2311208999999996	1.7116141000000000
H	1.6809605000000001	-5.7842076999999996	2.5593693000000002
H	1.7569963000000000	-5.5089525999999998	1.0503226000000001
O	4.3557933000000002	-5.3586425000000002	0.73289108000000003
H	4.1043139999999996	-5.9328073000000003	1.4336894000000000
H	3.5016379999999998	-4.9495129999999996	0.64212599000000004
O	-0.37478015999999997	-5.1934184999999999	-0.12606670000000000
H	0.30535109999999999	-4.5499276000000002	7.4995529000000005E-002
H	-0.73006104999999999	-5.4697731000000003	0.75663292000000004
O	-0.85536690999999998	-4.6655392000000004	3.4726420000000000
H	0.10089115000000000	-4.4031399999999996	3.4140953999999999
H	-0.90753572000000005	-5.4084165999999998	2.9002669000000001
O	4.1084684999999999	-5.4885001999999998	3.9527530999999998
H	4.4350693999999997	-4.6318089000000002	4.2700357999999996
H	3.1746694999999998	-5.1913781999999999	3.6306845999999999
O	1.5693153000000000	-3.9732742999999999	6.0472579000000000
H	2.2016452000000002	-4.6731809000000002	6.2858074999999998
H	1.6112420000000001	-4.1008113000000002	5.0731739999999999
O	3.9937892000000002	-2.8078273999999999	-1.6748719999999999
H	3.4669105999999998	-3.2246967000000000	-0.99922162999999997
H	4.6232666000000000	-2.3399526000000002	-1.0743830999999999
O	1.2055309999999999	-3.6783484000000000	-2.4661719999999998
H	1.4446935999999999	-4.5796748999999997	-2.8190016999999998
H	1.4268054999999999	-3.6634487999999998	-1.5268459999999999
O	1.1375510000000000	-8.4536598000000005	2.8472987999999999
H	1.4911158000000000	-7.6307974999999999	2.4499187999999998
H	2.0043878999999998	-8.9461875000000006	2.7262021999999999
O	-0.63233671000000002	-2.0259510999999999	4.6473306000000001
H	-0.63641950999999997	-2.9991292000000001	4.7200129000000004
H	0.32463549000000003	-1.8432126000000000	4.7035049999999998
O	4.2926222999999997	-2.9269061000000001	5.5850815999999996
H	3.3336321999999998	-2.8265815999999999	5.2981670000000003
H	4.3580841000000001	-2.4252609000000001	6.3848001999999999
O	6.4908400999999998	-3.9061819000000000	1.7628691999999999
H	5.8379156999999999	-4.4606937000000002	1.3300517999999999
H	7.1819490000000004	-4.5494991999999996	1.7351182000000001

C	0.19481095E+01	-0.30234169E+01	0.27459011E+01
C	0.19986026E+01	-0.16396065E+01	0.31943352E+01
O	0.20465640E+01	-0.72065369E+00	0.21639429E+01
C	0.18281005E+01	-0.15042912E+01	0.88855912E+00
C	0.18895180E+01	-0.29792890E+01	0.13460817E+01
O	0.19066616E+01	-0.12019640E+01	0.43454957E+01
C	0.49161933E+00	-0.11535762E+01	0.29615846E+00
O	-0.59031868E+00	-0.14370528E+01	0.12399795E+01
O	1.9205260000000000	-3.9480748999999999	0.46420091000000002
O	1.8531563000000000	-4.0565629999999997	3.5185514000000002
C	0.30836836E+00	0.32583559E+00	-0.11901900E+00
O	-0.10293937E+01	0.59857449E+00	-0.70435568E+00
H	0.26301072E+01	-0.12567892E+01	0.19777225E+00
H	0.48470605E+00	0.89643864E+00	0.76073632E+00
H	0.92992599E+00	0.66970849E+00	-0.91842563E+00
H	-0.16657439E+01	0.33318375E+00	0.46555037E-01
H	0.42862306E+00	-0.18144294E+01	-0.51006188E+00
H	-0.12017326E+00	-0.21211234E+01	0.17712751E+01
O	1.5904166000000000	-6.2659932999999999	1.8979790999999999
H	1.7068690000000000	-5.6314694000000003	2.6499451000000001
H	1.6798611999999999	-5.5750267999999998	1.2330544999999999
O	4.2132842000000004	-5.2835330999999996	0.71045179000000003
H	3.8382455000000002	-5.9272964000000004	1.3441333000000000
H	3.4268171999999999	-4.6947162999999996	0.59968723000000002
O	-0.77508021000000005	-4.5271435000000002	3.6819657000000001
H	0.19657516999999999	-4.5765114999999996	3.7109024000000002
H	-0.94769070000000000	-5.0436730000000001	2.8806850000000002
O	4.3784603999999998	-5.4199602999999996	3.5686306999999999
H	5.1268034000000000	-4.8586195999999999	3.3668985999999999
H	3.6196202999999998	-4.8105168000000003	3.7055375000000002
O	-0.39997556000000001	-5.2546111000000000	-0.31695404999999999
H	0.37540183999999999	-4.7690099000000004	1.9708967000000001E-002
H	-0.76339310000000005	-5.5989275999999997	0.49399838000000001
O	1.5333238000000000	-4.0211750000000004	6.2399696000000002
H	1.8045838000000001	-4.9419535000000003	6.6048720000000003
H	1.5093306000000000	-4.0846448999999998	5.2274567999999997
O	0.95314752999999997	-3.7764986999999999	-2.2092901000000000
H	1.2604541000000000	-4.5853579000000000	-2.6236989999999998
H	1.5558107000000001	-3.8249224000000002	-1.3691628000000000
O	4.3882105999999999	-3.0422796999999999	-1.6261515000000000
H	3.7189272999999998	-3.4539453000000000	-1.0934834000000000

H	4.5056231999999996	-2.2545883999999998	-1.1176060999999999
O	-0.93842166999999999	-1.9509563000000001	4.6059256000000000
H	-0.85803238999999998	-2.7466927999999999	4.1635930999999999
H	-4.5125938999999997E-002	-2.0355348000000002	5.0112598000000004
O	1.0783094000000000	-8.7326104999999998	2.7286760999999999
H	1.3229795000000000	-7.8345501999999998	2.4806246999999999
H	1.9506327999999999	-9.1503619999999994	2.5436447000000002
O	6.3059959000000001	-3.7784575000000000	1.9904077000000000
H	5.8891888000000003	-4.3651846000000001	1.3495824000000001
H	7.1592428000000004	-4.2244551000000001	2.1894524000000000
O	4.3304925000000001	-3.0063102000000002	5.6951228000000000
H	3.3721787000000001	-3.1004133999999999	5.7155072000000002
H	4.3422394999999998	-2.5196939000000000	6.5139221999999997

C	0.20068558E+01	-0.29384943E+01	0.26348319E+01
C	0.19402167E+01	-0.15861835E+01	0.30835726E+01
O	0.19717161E+01	-0.64307269E+00	0.20069292E+01
C	0.17928251E+01	-0.14144289E+01	0.81264934E+00
C	0.20147548E+01	-0.28545390E+01	0.12438491E+01
O	0.17536899E+01	-0.12001153E+01	0.42441335E+01
C	0.39455958E+00	-0.12113866E+01	0.24816225E+00
O	-0.50284552E+00	-0.15225127E+01	0.13585564E+01
O	2.0343513000000000	-3.8510548999999998	0.44130511000000000
O	2.0447185999999999	-4.0202799999999996	3.4029805000000000
C	0.12036755E+00	0.24771369E+00	-0.83714470E-01
O	-0.11465871E+01	0.48285072E+00	-0.67486926E+00
H	0.25076035E+01	-0.10504213E+01	0.46901371E-01
H	0.13203711E+00	0.93260982E+00	0.83382098E+00
H	0.82540947E+00	0.70141014E+00	-0.78783752E+00
H	-0.17992403E+01	0.64175298E+00	0.42232812E-01
H	0.20188589E+00	-0.18737960E+01	-0.61425317E+00
H	-0.13640104E+00	-0.22966940E+01	0.18318667E+01
O	1.5393460000000001	-6.3702562000000000	2.0235316000000001
H	1.7602537000000000	-5.8382963999999999	2.7760438999999999
H	1.5897893999999999	-5.6775070999999997	1.3392067000000001
O	4.2285520999999999	-5.6394849000000002	0.81318011999999995
H	4.2523223999999997	-6.2055322000000004	1.6445554000000000
H	3.5023355000000000	-4.9702723000000004	1.0710554999999999
O	4.2984515999999999	-5.3829295999999998	3.6787823999999998
H	5.0162173000000001	-4.8330460000000004	3.4863411000000002
H	3.5212838000000000	-4.7841291999999997	3.4215173999999999
O	-0.70938926999999996	-4.4037477000000003	3.2636528000000000
H	0.23853062999999999	-4.3384637000000001	3.3567562000000000
H	-0.78634640000000000	-5.3452114999999996	2.9960518999999999
O	-0.58264081000000001	-5.1015215999999999	-9.3329227000000001E-002
H	0.30881625000000001	-4.7386654999999998	0.11023839000000001
H	-1.0303290000000001	-5.3120984000000000	0.75850393999999999
O	4.1361660999999996	-3.1830596000000000	-1.5770983999999999
H	3.4378511000000000	-3.5760527000000000	-0.96349056000000000
H	4.5319592000000002	-2.5685270000000000	-1.0051782000000000
O	1.4212013999999999	-4.0331307000000001	6.0728128999999997
H	1.8595664000000001	-4.8899058000000002	6.3612950000000001
H	1.7819166000000000	-3.9628207999999998	5.1499625000000000
O	0.95914374999999996	-3.7874555000000001	-2.3326688000000000
H	1.7146850000000000	-4.3094405000000000	-2.5533109999999999
H	1.2207197999999999	-3.7665758999999999	-1.3441197000000000
O	4.2924695000000002	-2.8312281000000001	5.4455913999999996
H	3.3671015000000000	-2.8927594000000001	5.0601145000000001
H	4.1404920000000001	-2.3466705000000001	6.2656267000000003
O	6.3656309999999996	-4.0902903000000004	1.6814605000000000
H	5.7693599999999998	-4.6988344000000000	1.2736132000000000
H	7.0445491000000002	-4.7161488000000000	1.7599758999999999
O	-0.97526363000000005	-2.1253182000000002	4.4380549000000000
H	-1.1138428000000000	-3.0598158999999998	4.0287132000000003
H	-4.0207645000000000E-002	-2.1407001000000001	4.6433657000000004
O	1.2697274000000001	-8.9543423000000004	2.7567986000000002
H	1.3137938000000000	-8.0964627999999994	2.3245355000000001
H	2.1618471000000001	-9.3035463000000007	2.6070319000000000

C	0.19101670E+01	-0.30900019E+01	0.26550006E+01
C	0.19230230E+01	-0.16645340E+01	0.30479069E+01
O	0.18703492E+01	-0.81576953E+00	0.19753327E+01
C	0.17957817E+01	-0.16644273E+01	0.82127392E+00
C	0.19602084E+01	-0.31054988E+01	0.12646300E+01
O	0.18815966E+01	-0.11441197E+01	0.41757234E+01
C	0.33309669E+00	-0.15353599E+01	0.23756847E+00

O	-0.68049538E+00	-0.18963480E+01	0.12217094E+01
O	2.0722984000000002	-4.0119536000000000	0.37150201999999999
O	1.8024860000000000	-4.0593940000000002	3.5212794000000001
C	0.27929262E-01	-0.12120283E+00	-0.22773396E+00
O	-0.13167263E+01	-0.60067447E-01	-0.69828779E+00
H	0.24652460E+01	-0.12200810E+01	0.11420488E+00
H	0.22725046E+00	0.65839369E+00	0.54616329E+00
H	0.66536182E+00	-0.13308227E-01	-0.11346602E+01
H	-0.18321478E+01	-0.32026961E-01	0.12793200E+00
H	0.25062718E+00	-0.23088115E+01	-0.55623521E+00
H	-0.29386009E+00	-0.25631192E+01	0.18629597E+01
O	1.8852622000000001	-6.3290620000000004	1.9445422000000001
H	2.0712581000000001	-5.8784060000000000	2.8108694999999999
H	1.9964701000000000	-5.5517652000000002	1.2724077000000000
O	4.3573697999999998	-5.5239247000000002	1.1899146000000000
H	3.6403428000000000	-6.1932023999999997	1.3703555999999999
H	3.6964996999999999	-4.9276140000000002	0.75272455999999999
O	-0.35153964999999998	-5.2195233999999999	-0.19668881999999999
H	0.55230047999999998	-4.9020253000000000	6.9373715999999997E-003
H	-0.53294573999999995	-5.7056908999999996	0.63663181999999996
O	4.2081179999999998	-5.5443046999999996	3.8938836999999999
H	4.4402825000000004	-5.4322977999999997	2.9529690000000000
H	3.3510938000000001	-5.0856402000000003	3.9101778000000000
O	-1.0496650999999999	-4.7622064000000002	3.6757493000000001
H	-7.3034797999999998E-002	-4.6615826000000000	3.6779397000000000
H	-1.1940640000000000	-5.5930431000000000	3.1790170000000000
O	4.4214368000000004	-3.3304266999999999	-1.2298258000000000
H	3.5946889000000000	-3.5643378000000001	-0.81395033999999999
H	4.4883329999999999	-2.4572862000000000	-0.85100452999999998
O	1.5612393000000000	-4.3011080000000002	6.2181446999999999
H	2.4102201999999999	-4.5717622999999996	6.5217454999999998
H	1.6930293999999999	-4.1562713999999996	5.2796165999999998
O	1.3688989000000000	-3.5118543999999998	-2.4887850000000000
H	1.5976417000000001	-4.2930843999999997	-3.0064388000000002
H	1.4218189000000001	-3.8329745000000002	-1.5721375000000000
O	-0.95219341999999996	-2.0714280999999999	4.4057731000000002
H	-1.0941816000000000	-3.0236201000000000	4.2153152000000000
H	4.1190931000000000E-002	-1.9989218000000000	4.4249435999999998
O	4.2539180999999999	-2.8303137000000000	5.5774262999999999
H	3.5630784000000002	-2.3796363999999999	5.0698903000000000
H	4.3379946000000000	-2.1884785000000000	6.3180873999999996
O	6.5403025000000001	-3.9703966999999998	1.9652877000000000
H	5.7982617999999997	-4.5241210000000001	1.4675107000000001
H	7.0915600000000003	-4.7085014999999997	2.2255161999999999
O	1.1933714000000000	-8.8624080000000003	2.7013957000000000
H	1.3730388000000000	-7.9758227000000002	2.3179813000000000
H	1.8624057000000001	-9.4078599999999994	2.2977194000000001

C	0.18981901E+01	-0.32023805E+01	0.25020825E+01
C	0.18898852E+01	-0.17921441E+01	0.28776919E+01
O	0.18613445E+01	-0.92805286E+00	0.17865132E+01
C	0.16197482E+01	-0.18053276E+01	0.64379884E+00
C	0.20042861E+01	-0.31792893E+01	0.11270923E+01
O	0.20229446E+01	-0.13322598E+01	0.40464877E+01
C	0.51084250E-01	-0.17532638E+01	0.24186598E+00
O	-0.84368563E+00	-0.19554454E+01	0.13869133E+01
O	2.0977652999999998	-4.1465163000000000	0.22586355000000000
O	1.9711155000000000	-4.1405139999999996	3.4276323000000000
C	-0.21333394E+00	-0.41999449E+00	-0.43857973E+00
O	-0.15477308E+01	-0.13876181E+00	-0.85103983E+00
H	0.22901275E+01	-0.15039771E+01	-0.21175789E+00
H	0.24098945E+00	0.36286323E+00	0.22616499E+00
H	0.35853431E+00	-0.43143232E+00	-0.14401164E+01
H	-0.21162669E+01	0.36862469E-01	-0.91657848E-01
H	-0.19590158E+00	-0.26027932E+01	-0.41803188E+00
H	-0.44372274E+00	-0.27139446E+01	0.18501751E+01
O	1.6316843999999999	-6.4754345999999998	1.8436395000000001
H	1.6574918000000001	-5.7920303999999998	2.4959190000000002
H	1.8290101999999999	-5.8618768000000001	1.0921757999999999
O	4.3134778999999996	-5.3301926000000002	0.99583999999999995
H	3.8277828999999999	-5.8780456000000001	1.7108676000000000
H	3.6583584000000000	-4.7586461000000000	0.50332984999999997
O	4.1891445999999997	-5.4912589000000001	4.0379475999999999
H	4.6503214000000002	-4.7432568000000002	4.4276483000000004
H	3.3755993000000002	-5.0217026000000002	3.7351703999999999
O	-0.81075971999999996	-5.1159207000000002	3.8403375999999998

H	-1.6188846000000000E-002	-4.7250684999999999	3.4481362000000000
H	-1.1194656000000001	-5.7512308000000001	3.1629801000000000
O	-0.85552592000000005	-5.2098443000000003	1.6077959999999999E-002
H	5.8596263000000003E-002	-5.1628128000000002	0.26959084000000000
H	-1.2216180999999999	-5.9691473000000004	0.58312288999999995
O	4.4003076999999999	-3.4580128000000001	-1.5535412000000000
H	3.6339814000000001	-3.7629046000000002	-0.98334801000000005
H	4.7358697999999997	-2.7049555999999999	-1.0186215000000001
O	1.6186898999999999	-4.1864074999999996	6.1722504000000002
H	2.5629119999999999	-4.0605756000000000	6.2182788000000002
H	1.4822191000000000	-4.0301124000000002	5.2122251999999998
O	4.2986462000000003	-2.9694626999999998	5.3613743999999999
H	3.5261298999999999	-2.7046684000000001	4.7850327999999998
H	4.3694595999999999	-2.2785720999999999	6.0994399000000001
O	0.92397324999999997	-3.4024453000000001	-2.5412911999999999
H	1.5261218999999999	-3.6823434000000002	-3.2559108999999999
H	1.3801277999999999	-3.7195151000000002	-1.7485359000000000
O	6.5560060000000000	-4.0089473000000000	2.0730960999999999
H	5.7743558999999998	-4.4478144000000004	1.6880828000000001
H	7.0981455999999996	-4.7857007999999999	2.1151981000000002
O	1.2673741999999999	-8.8939266999999997	2.8121347999999999
H	1.5250706999999999	-8.0245043999999996	2.4855662000000001
H	2.0413036000000000	-9.4485472000000001	2.7079203999999999
O	-1.2057502000000000	-2.3051222999999998	4.6718868999999996
H	-1.1928407000000001	-3.0619633999999998	4.0462021000000004
H	-0.30194986000000001	-2.4494432000000002	4.9375429999999998

C	0.16933973E+01	-0.30444333E+01	0.25124590E+01
C	0.18901861E+01	-0.16682460E+01	0.29435749E+01
O	0.19082928E+01	-0.77491948E+00	0.18564988E+01
C	0.16273143E+01	-0.15552268E+01	0.69435848E+00
C	0.18768433E+01	-0.29910964E+01	0.11186895E+01
O	0.21013355E+01	-0.12652279E+01	0.40780066E+01
C	0.23551949E+00	-0.14550257E+01	0.13792678E+00
O	-0.82241893E+00	-0.17259095E+01	0.10914454E+01
O	2.1741741000000001	-3.9471058999999999	0.24699472000000000
O	1.7442161000000000	-4.1079923999999997	3.3998401000000000
C	-0.11956429E+00	-0.37469583E-01	-0.38195887E+00
O	-0.14416353E+01	0.10586765E+00	-0.80691799E+00
H	0.23586096E+01	-0.12748490E+01	-0.13519623E+00
H	0.21808685E-01	0.70759649E+00	0.43475778E+00
H	0.47016665E+00	0.20409824E+00	-0.12904227E+01
H	-0.20392600E+01	0.24489657E+00	-0.68406087E-01
H	0.31700821E+00	-0.21611763E+01	-0.69473872E+00
H	-0.57900163E+00	-0.23095133E+01	0.18345244E+01
O	1.4567277999999999	-6.3563498000000003	1.7038390999999999
H	1.5799810000000001	-5.8665929999999999	2.5368832000000001
H	1.8286492999999999	-5.6515371999999999	1.1185607000000000
O	4.1312929000000000	-5.2457221000000001	3.5198752999999998
H	4.4915541000000001	-4.4198392999999996	3.8082600000000002
H	3.1908162999999998	-4.8919978000000004	3.4603100000000002
O	4.4760740999999999	-5.3877968000000003	0.67948242000000003
H	4.4775219000000002	-5.4978780000000000	1.6357956000000000
H	3.5113699999999999	-5.2300711000000000	0.57482683999999995
O	-0.92640233999999999	-4.8297727000000004	0.35682575000000000
H	6.5170000000000002E-003	-4.7389931000000001	0.61113472999999996
H	-1.1959689000000000	-5.6544692000000003	0.67426326000000003
O	-0.74690763000000004	-5.2180384000000002	3.6877466000000001
H	0.18536047000000000	-4.9563986000000000	3.5157208999999999
H	-0.98990944000000003	-5.7240286999999999	2.8404978000000001
O	4.0535734999999997	-3.2607295999999999	-1.5415061999999999
H	3.3798580000000000	-3.5290976999999999	-0.83627147999999996
H	4.4572702000000000	-2.5403783999999998	-1.0398414000000000
O	1.5736612000000001	-4.0879060000000003	6.1484125000000001
H	2.5661170000000002	-4.0373180000000000	6.2080291000000001
H	1.4926378000000000	-4.0484346999999996	5.1591902999999997
O	1.1672994999999999	-3.6165295999999998	-2.5980333999999998
H	1.7055674000000001	-4.2777364999999996	-3.0041091999999998
H	1.5672632000000000	-3.5701103000000001	-1.7059343000000000
O	-1.2154763000000000	-2.4602284000000001	4.3890650999999998
H	-1.7430243999999999	-3.2621329999999999	4.1018239999999997
H	-0.34408472000000001	-2.8155130000000002	4.5848880000000003
O	4.2027201999999999	-2.6638044999999999	5.5124409999999999
H	3.2738529999999999	-2.4355978000000000	5.2278150999999999
H	4.4552730000000000	-2.1433171999999998	6.3671087999999996
O	6.7303438000000000	-3.9492677999999999	1.9088822999999999
H	5.9994252000000001	-4.1790592999999996	1.2945860000000000
H	7.1998248000000000	-4.8221657000000002	2.0117756000000000
O	4.5804387000000002	-7.2682774999999999	-1.1656385000000000
H	4.3266251000000002	-6.6907205000000003	-0.35451885000000000
H	5.4137763000000003	-6.8538711000000001	-1.4025095000000001

C	0.18922933E+01	-0.30255229E+01	0.24999430E+01
C	0.19882449E+01	-0.16925406E+01	0.29506263E+01
O	0.18733102E+01	-0.74252108E+00	0.18815969E+01
C	0.16070775E+01	-0.15210042E+01	0.73873866E+00
C	0.17933349E+01	-0.29512582E+01	0.11526699E+01
O	0.21588248E+01	-0.12337171E+01	0.41165701E+01
C	0.11105657E+00	-0.14310670E+01	0.23357222E+00
O	-0.80313855E+00	-0.15729981E+01	0.13592614E+01
O	1.8265662000000000	-3.8869492000000001	0.24773123999999999
O	1.9464735000000000	-4.1084126999999997	3.2865207000000001
C	-0.18139433E-02	-0.33888842E-01	-0.42812851E+00
O	-0.13629840E+01	0.21951129E+00	-0.95066877E+00
H	0.22439657E+01	-0.12368047E+01	-0.64470844E-01
H	0.30043352E+00	0.78558974E+00	0.27359788E+00
H	0.57047654E+00	0.48550602E-01	-0.13421583E+01
H	-0.19817220E+01	0.30454842E+00	-0.20060008E+00
H	-0.13954105E+00	-0.22326701E+01	-0.50576221E+00
H	-0.46046902E+00	-0.23076602E+01	0.19021384E+01
O	1.5873835999999999	-6.6437793999999997	1.8812841000000000
H	1.4895387000000000	-5.7193877999999998	2.2611344999999998
H	2.0369377000000002	-6.3328325000000003	1.0275893000000000
O	4.2951363000000002	-5.0759746999999997	0.84696066999999997
H	4.2966765999999996	-5.3192174999999997	1.8196912999999999
H	3.4324887999999998	-4.6311456000000000	0.72093755000000004
O	-0.54856227999999996	-5.3059251999999999	3.8217705000000000
H	0.36072125999999999	-4.9362712999999996	3.5863060999999998
H	-0.84991682999999996	-5.6788487999999999	2.9134259000000000
O	-0.79916606999999995	-5.1199842000000002	-0.13155901000000000
H	8.4183630999999995E-002	-5.0044876000000000	0.24515081999999999
H	-1.2772736000000000	-5.5494602999999998	0.59380252000000000
O	4.2206913999999998	-5.5006893999999997	3.5533169000000000
H	4.8300846000000002	-4.7166632000000002	3.7083173999999999
H	3.3211246999999999	-5.1281929000000002	3.6056794999999999
O	4.2110278000000001	-3.3471147000000001	-1.4308301000000001
H	3.4821403000000002	-3.6996693000000000	-0.92542312999999998
H	4.4335186000000002	-2.6595963999999999	-0.80695444000000005
O	0.92986440000000004	-3.5795357000000001	-2.4073866000000002
H	1.4470658000000001	-4.1541490000000003	-2.9045293999999999
H	1.1896865999999999	-3.7793524999999999	-1.4544010999999999
O	1.7316815000000001	-4.0700946000000000	6.1714523999999997
H	2.6490279999999999	-3.8546713000000001	6.0483396999999997
H	1.4734248999999999	-4.1989672000000002	5.1923876000000000
O	-1.0049660000000000	-2.7057251000000000	4.5648626999999999
H	-1.2048479999999999	-3.6177391000000001	4.3988526999999999
H	-5.5661909000000002E-002	-2.8082962000000000	4.8130426999999996
O	6.4755168999999997	-3.7917573999999998	2.1773601000000000
H	5.8787501999999998	-4.1190626000000004	1.4598447000000001
H	7.0495726000000003	-4.5705872999999997	2.1513414000000002
O	4.6392711000000002	-7.0021621999999999	-1.0926225000000001
H	4.3352972999999997	-6.5021585999999996	-0.30793385000000001
H	5.3038647000000001	-6.3341270999999999	-1.5044115000000000
O	4.1642207999999998	-2.7188672000000000	5.4087638000000000
H	3.5656922999999999	-2.0221328000000001	5.0613698999999999
H	4.5627643000000004	-2.2601037000000002	6.2321410999999998

C	0.18113028E+01	-0.31072160E+01	0.25672158E+01
C	0.18107691E+01	-0.17685011E+01	0.30638617E+01
O	0.18847921E+01	-0.79618945E+00	0.19922105E+01
C	0.17754599E+01	-0.15979100E+01	0.77256737E+00
C	0.19348717E+01	-0.29955239E+01	0.12056895E+01
O	0.17343829E+01	-0.13816937E+01	0.42808088E+01
C	0.38517779E+00	-0.13613287E+01	0.15801885E+00
O	-0.65076895E+00	-0.15991815E+01	0.12078435E+01
O	2.0422932999999999	-3.9280593000000001	0.27696885999999998
O	1.7316407000000000	-4.1175464000000002	3.3976421999999999
C	0.14118259E+00	0.31124827E-01	-0.43729752E+00
O	-0.11961687E+01	0.61412535E-01	-0.93231168E+00
H	0.24166804E+01	-0.11550024E+01	-0.46564710E-01
H	0.29348039E+00	0.78845448E+00	0.27352785E+00
H	0.87788156E+00	0.19258027E+00	-0.12406377E+01

H	-0.17127251E+01	-0.28301349E+00	-0.13676599E+00
H	0.25431518E+00	-0.21358409E+01	-0.63406915E+00
H	-0.57020364E+00	-0.95850406E+00	0.19033839E+01
O	1.6826242000000000	-6.3875798000000001	1.9180151999999999
H	1.6207209000000000	-5.5574140999999999	2.5237854999999998
H	1.8205392000000000	-6.0450096000000002	1.0443958000000000
O	4.3979816999999999	-5.2851375000000003	0.96056386000000005
H	4.2563715999999996	-5.5393309000000004	1.9187988000000000
H	3.5155577999999998	-4.9707688000000001	0.71532651999999997
O	-0.66508047999999997	-5.0497658999999997	-5.5640615999999997E-002
H	0.18881128999999999	-4.6133759000000003	0.18908733999999999
H	-0.86416068999999995	-5.6292691000000001	0.66472807000000000
O	4.1964788000000004	-5.5540823000000001	3.6053777999999999
H	4.9153254000000004	-4.8778930999999996	3.7431619999999999
H	3.3872333999999999	-5.1179123000000004	3.6084711999999999
O	-0.41133420999999998	-5.8462708000000001	4.0028766999999998
H	0.47659709000000000	-5.5504540999999996	3.8336858999999999
H	-0.55252946999999997	-6.3014260000000002	3.1685807000000001
O	4.4704484000000004	-3.2160356000000001	-1.2206158000000000
H	3.6538636000000002	-3.5431580999999999	-0.77025686000000004
H	4.5186647999999998	-2.3058209999999999	-0.88854555000000002
O	1.0083578000000000	-3.6049454999999999	-2.3479009000000000
H	1.3836583000000000	-4.3650041999999996	-2.8514287999999999
H	1.2739016000000001	-3.6239753000000001	-1.3754089000000000
O	-1.1698763000000001	-3.0887516000000002	4.4932037999999999
H	-1.3453271000000000	-4.0368282000000004	4.3578662000000001
H	-0.18088185000000001	-3.1716451000000001	4.5197313000000001
O	1.8116566000000001	-4.1147194000000002	6.3078446000000001
H	2.7043020000000002	-3.8074276999999999	6.4159617999999998
H	1.7313778000000000	-3.9520461999999998	5.3441751000000002
O	6.5333882000000001	-3.6097668000000001	2.5446802000000002
H	5.7096628999999997	-3.6599753000000002	1.9158717000000001
H	6.9315154000000003	-4.5235158000000002	2.4769184000000002
O	4.1943409999999997	-2.6136857000000000	5.5128598000000002
H	3.4722371000000001	-2.2334501000000002	4.9967120999999999
H	4.4083465999999998	-1.9668420000000000	6.2424004999999996
O	1.2784245999999999	-8.9573497999999994	2.9542690999999999
H	1.3425586000000000	-8.1306805999999998	2.3939948000000002
H	2.0480181000000002	-9.4264077000000004	2.6171937999999999

C	0.19334847E+01	-0.31360828E+01	0.26076749E+01
C	0.19906093E+01	-0.17921053E+01	0.31577420E+01
O	0.19837126E+01	-0.83758552E+00	0.21157270E+01
C	0.18059672E+01	-0.15591466E+01	0.87644865E+00
C	0.19338736E+01	-0.30561720E+01	0.12162461E+01
O	0.19392664E+01	-0.13928422E+01	0.43373454E+01
C	0.40624877E+00	-0.13799329E+01	0.24905893E+00
O	-0.64470116E+00	-0.15410853E+01	0.12452134E+01
O	1.8132302000000000	-4.0213074999999998	0.37487473999999998
O	1.8590130000000000	-4.1681448000000003	3.4393961000000002
C	0.19396673E+00	0.21261845E-01	-0.43222618E+00
O	-0.10799695E+01	0.17075116E+00	-0.95464911E+00
H	0.25631695E+01	-0.12343668E+01	0.14213268E+00
H	0.38177857E+00	0.82376275E+00	0.31246541E+00
H	0.91574074E+00	0.24245524E+00	-0.12637151E+01
H	-0.17137286E+01	-0.65284579E-01	-0.28886510E+00
H	0.18211176E+00	-0.21827423E+01	-0.48404550E+00
H	-0.34611763E+00	-0.21837780E+01	0.19307316E+01
O	1.8798288999999999	-6.4830439000000002	1.8541417000000000
H	2.0542194999999999	-6.0443204000000001	2.6506064000000000
H	1.6276527999999999	-5.7404792999999996	1.2025260000000000
O	4.4232706000000004	-5.1782807000000002	0.92454575000000006
H	4.2332026999999997	-5.6539421000000001	1.8129137000000000
H	3.5412716999999998	-4.9175844000000000	0.74560037999999995
O	4.0362891999999997	-5.7136623999999996	3.6727794999999999
H	4.7817151999999998	-5.1446449999999997	3.5568168000000000
H	3.2664314999999999	-5.1014632999999998	3.5886947999999999
O	-0.63454663000000000	-4.8909244000000003	-0.16797915999999999
H	0.22413965000000000	-4.5580489000000002	0.14517421000000000
H	-0.76234535000000003	-5.6496583999999999	0.39500706000000002
O	-0.32161902999999997	-5.8638437999999997	4.0732751000000000
H	0.51591944999999995	-5.3548315000000004	3.8095287000000000
H	-0.72494877000000002	-6.1044156999999997	3.1926931999999999
O	3.9686875000000001	-3.0968618999999999	-1.3995015000000000
H	3.1420503000000002	-3.2805998000000001	-0.92290284000000000
H	4.4481275000000000	-2.5243837999999998	-0.84036058000000002
O	1.6641248000000000	-3.8918457000000002	6.1642957000000003
H	2.6456477999999999	-3.8732129000000000	6.2324846999999997
H	1.6516717999999999	-3.7989437000000001	5.2002157000000002
O	-0.86835097000000006	-3.2816869000000000	4.5947554000000004
H	-1.2151921999999999	-4.1715451999999997	4.4883017000000001
H	8.9292544000000001E-002	-3.3765375000000000	4.6363674000000001
O	1.0999060000000001	-3.2850760999999999	-2.3129482000000001
H	1.3935295000000001	-3.8153340999999998	-3.0518730000000001
H	1.3487199999999999	-3.8590743999999999	-1.5589693000000000
O	1.3342598999999999	-8.8865204000000002	3.0731071000000001
H	1.3546385999999999	-7.9803370999999999	2.7441342999999998
H	2.0162874999999998	-9.2450296999999999	2.5101996000000000
O	6.4423858999999997	-3.6612760999999998	2.3757720999999998
H	5.8200270999999999	-3.7090505999999999	1.6549714000000000
H	6.7164256000000000	-4.5821487999999997	2.3337995999999999
O	-2.8813287000000001	-2.9625971000000000	0.68050341999999997
H	-2.7356064999999998	-3.5365117000000001	-7.7149923999999995E-002
H	-1.9839443999999999	-2.6574946000000002	0.86848093999999998

C	0.18258432E+01	-0.31705324E+01	0.26749657E+01
C	0.18928697E+01	-0.18269430E+01	0.30896851E+01
O	0.16918416E+01	-0.94377040E+00	0.20458958E+01
C	0.16433004E+01	-0.16596498E+01	0.79482389E+00
C	0.18003935E+01	-0.31051389E+01	0.12544418E+01
O	0.20468237E+01	-0.13993756E+01	0.42425001E+01
C	0.20984536E+00	-0.14336864E+01	0.26735053E+00
O	-0.73559115E+00	-0.17146490E+01	0.13105167E+01
O	1.8653865000000001	-4.0255630000000000	0.33389762000000001
O	1.8837212999999999	-4.3001429000000000	3.4119370999999998
C	0.94740757E-01	-0.26085237E-01	-0.34311985E+00
O	-0.11093101E+01	0.16189354E+00	-0.10943590E+01
H	0.24990702E+01	-0.12808714E+01	0.26665869E+00
H	0.31788031E+00	0.75519483E+00	0.34226504E+00
H	0.85617334E+00	-0.18906124E-01	-0.11256795E+01
H	-0.18561522E+01	0.12044007E+00	-0.51405030E+00
H	-0.47295036E-01	-0.20818253E+01	-0.58740993E+00
H	-0.57695170E+00	-0.12991802E+01	0.21470055E+01
O	1.9099976000000001	-6.4285188000000000	1.7146538000000000
H	2.0833366000000000	-5.6648712999999997	2.3249233999999999
H	1.7392718000000000	-5.8325883999999997	0.93643354000000001
O	4.6346993999999997	-5.2052364000000004	1.0612303999999999
H	4.2960513000000002	-5.7546992000000001	1.7652276000000000
H	3.7970608000000001	-4.7589673000000001	0.76169233000000003
O	4.1077992999999999	-5.8206471999999998	3.6737883000000000
H	4.6031972000000003	-4.9953424000000002	3.6426656999999998
H	3.2240302999999999	-5.3743305000000001	3.4571562000000000
O	-0.80749636000000002	-5.0153907999999996	-0.25485562000000000
H	6.8180081000000003E-002	-4.8238731000000001	5.1234113999999997E-002
H	-1.1776614999999999	-5.5969167999999998	0.46318028999999999
O	-0.50961440000000002	-5.7197177999999997	4.1892040000000001
H	0.38276796000000002	-5.4291277999999998	3.9040149999999998
H	-0.79966841000000000	-6.1717012000000002	3.3547908000000000
O	1.7629992999999999	-3.8547668000000002	6.0787174000000004
H	2.4651375999999998	-4.5367449999999998	6.2448522999999998
H	1.7346490000000001	-3.8316240000000001	5.0941650999999997
O	4.4095366000000000	-3.2211639999999999	-1.4371035000000001
H	3.6075707000000001	-3.3104334999999998	-0.94646859000000005
H	4.6744270999999999	-2.4087977000000000	-1.0110645000000000
O	1.2260187000000000	-3.2395491999999999	-2.4055209000000000
H	1.2332277000000000	-3.9852690000000002	-3.0538028000000002
H	1.4688028000000000	-3.6003398000000000	-1.4904288000000001
O	1.3788347000000001	-8.7427767999999997	2.8279814000000001
H	1.5302832000000000	-7.8270721999999999	2.5535301000000001
H	2.1557171999999998	-9.2242876000000003	2.5132017000000002
O	6.5383310999999997	-3.5001750000000000	2.0213454000000000
H	5.7832116999999998	-3.9385043999999998	1.5968747000000001
H	7.1878428999999997	-4.1364998999999996	2.0388736999999999
O	-0.92338471000000000	-2.9715349000000000	5.3640777000000002
H	-1.0250729000000001	-3.7473831000000000	4.7605046000000000
H	1.6044249999999999E-002	-2.9248083000000000	5.5709518999999998
O	4.7785517000000004	-7.0933286999999998	-0.84057764000000001
H	4.6811053999999999	-6.6050088000000002	7.2472528999999999E-003
H	5.5265459000000003	-6.6956265999999998	-1.3101432000000000

C	0.16735306E+01	-0.32075689E+01	0.27114432E+01
C	0.15298403E+01	-0.18758704E+01	0.32132172E+01

O	0.14410989E+01	-0.90990138E+00	0.22360893E+01
C	0.14107876E+01	-0.16238831E+01	0.99624583E+00
C	0.16619734E+01	-0.31102646E+01	0.13397816E+01
O	0.16184525E+01	-0.14957336E+01	0.44156570E+01
C	0.12971253E+00	-0.13300960E+01	0.10310809E+00
O	-0.10828104E+01	-0.15292072E+01	0.86745308E+00
O	1.7949392000000000	-3.9902131000000001	0.43029684000000001
O	1.7070988000000000	-4.3185387999999998	3.4701333999999999
C	0.19261013E+00	-0.20859435E-01	-0.61256372E+00
O	-0.97358251E+00	0.28472571E+00	-0.13526568E+01
H	0.22571881E+01	-0.11384578E+01	0.47207413E+00
H	0.30163250E+00	0.76535531E+00	0.12912799E+00
H	0.10496488E+01	-0.71342266E-01	-0.13041944E+01
H	-0.18008919E+01	0.17219835E+00	-0.84641615E+00
H	0.16717133E+00	-0.21054895E+01	-0.64685816E+00
H	-0.98087045E+00	-0.10923069E+01	0.17373297E+01
O	1.6317656000000000	-6.4843498000000004	1.8103526999999999
H	1.5520388000000001	-5.7775499000000003	2.4975486999999998
H	1.7570119000000000	-5.9386991000000000	0.97059675000000001
O	3.9141455000000001	-5.6648022999999998	3.4528365000000001
H	4.0629173999999999	-4.8287518000000000	4.0043357000000004
H	2.9505102999999999	-5.4877301000000003	3.3200400999999999
O	4.5444101999999997	-4.9667899000000002	0.86499762999999996
H	4.3045711999999998	-5.3434241000000000	1.7757209000000000
H	3.7016442999999999	-4.5824033000000002	0.55222777999999995
O	-0.68479456000000005	-5.3628679999999997	4.1098084000000004
H	0.14279796000000000	-4.9486758999999996	3.7226262000000001
H	-1.0577072999999999	-5.8558022000000003	3.3510320000000000
O	-0.84768701000000002	-5.4406650000000001	-0.28537910000000000
H	-2.8529414999999999E-002	-5.1174808000000001	-2.4152593999999999E-002
H	-1.2589777000000000	-5.8370128000000001	0.55693201999999997
O	1.8238981000000001	-4.0681013999999998	6.2050764000000003
H	2.4352271999999999	-4.7218596000000002	6.4235546000000001
H	1.8307179000000000	-4.0422564999999997	5.2185078000000003
O	4.0330021000000000	-3.2875348999999998	-1.4437153000000000
H	3.1984048999999999	-3.3314089000000000	-0.97019485999999999
H	4.6342686999999998	-2.6237089000000000	-0.99361542999999997
O	1.3249135999999999	-3.3103356000000002	-2.3506323999999998
H	1.1728171000000001	-4.1508023999999999	-2.7958436999999998
H	1.3865977000000000	-3.6389779000000000	-1.4296888999999999
O	-1.0278654000000000	-2.8212136999999999	5.0813402999999999
H	-1.0811987000000001	-3.7458008999999999	4.6787555000000003
H	-0.11120756000000000	-2.7585750999999998	5.4690022000000003
O	-1.1957572999999999	-7.0291079999999999	2.0312557999999998
H	-0.39796943000000001	-7.4993508999999996	1.7750391999999999
H	-1.6696469000000000	-7.7401109000000003	2.4791219999999998
O	4.4832039000000004	-2.9013274999999998	5.6016702000000000
H	3.5385173000000001	-2.9115848999999998	5.3650335000000000
H	4.5615332000000004	-2.2907867000000000	6.3504516999999998
O	1.1344828000000000	-8.9990561000000007	2.8118892999999998
H	1.3798618000000000	-8.1650205000000007	2.4107506000000001
H	2.0042753000000002	-9.4732094000000000	2.7342919000000001

C	0.15025866E+01	-0.33091353E+01	0.25947376E+01
C	0.17600317E+01	-0.19997639E+01	0.31498269E+01
O	0.17457398E+01	-0.10210112E+01	0.21408051E+01
C	0.14467639E+01	-0.17522008E+01	0.86504618E+00
C	0.15438909E+01	-0.32205881E+01	0.12293311E+01
O	0.20439516E+01	-0.15653943E+01	0.43245616E+01
C	0.10338522E+00	-0.13085786E+01	0.23816946E+00
O	-0.10001162E+01	-0.14237939E+01	0.12036258E+01
O	1.5337019999999999	-4.1278879999999996	0.38284449999999998
O	1.4662733999999999	-4.4061367999999996	3.3751068000000002
C	0.45020629E-01	0.95797117E-01	-0.48004312E+00
O	-0.12033257E+01	0.41185885E+00	-0.11356969E+01
H	0.22713322E+01	-0.15332997E+01	0.21368265E+00
H	0.25513760E+00	0.88286235E+00	0.24384723E+00
H	0.79612175E+00	0.14287979E+00	-0.12773040E+01
H	-0.19314043E+01	0.27549939E+00	-0.50909259E+00
H	-0.52626112E-01	-0.19969149E+01	-0.57431010E+00
H	-0.64546465E+00	-0.21698619E+01	0.18691612E+01
O	1.3417616999999999	-6.7890701000000000	1.4952692999999999
H	1.3826037000000000	-6.0461131000000004	2.1169015999999998
H	1.4149432000000000	-6.2513866000000000	0.72153867999999999
O	4.0820387000000000	-5.6539035999999996	3.6110809000000001
H	4.3744972000000004	-4.9742663000000000	4.2499070000000003

H	3.1467225999999999	-5.3902121999999997	3.4055529999999998
O	4.5270961999999999	-4.9364089000000000	1.0560566000000000
H	4.3641829000000003	-5.2046982000000002	2.0077048000000000
H	3.6806918999999998	-4.7245353000000003	0.65804021000000001
O	-0.92386409999999997	-5.3613007000000001	-0.42228587000000001
H	-0.12345287000000001	-4.9058184000000002	-0.15317462000000001
H	-1.2849088000000000	-5.7488381999999998	0.39015590000000000
O	-0.99343848999999995	-5.0322836999999998	4.2060978999999996
H	-8.0350202999999995E-002	-4.7086756000000003	4.0147066000000002
H	-1.1647786000000000	-5.6831927000000002	3.5464943999999998
O	1.8606731000000001	-4.2283248000000002	6.1004883000000003
H	2.7662369999999998	-4.3990679000000004	6.2321739999999997
H	1.7710661999999999	-4.3978830000000002	5.1171803999999996
O	0.81047462999999997	-3.2438429000000002	-2.1867543000000000
H	1.0589303999999999	-3.9949981999999999	-2.7140594000000000
H	1.0558403000000001	-3.5399229999999999	-1.2837901000000000
O	3.9267580999999998	-3.2409423999999998	-1.6641234000000000
H	3.0152608000000001	-3.4434635999999998	-1.3063990000000001
H	4.2262636000000002	-2.5727856999999998	-0.98490451000000001
O	-0.89836799000000001	-2.3043703000000000	4.8767218999999997
H	-1.1125221999999999	-3.2146455000000000	4.7771455999999999
H	5.8007660000000003E-002	-2.4116247000000000	4.8056508999999998
O	-1.5252289999999999	-6.9743573999999997	2.0519908999999998
H	-1.0142788000000000	-7.7158297999999998	1.6361246000000000
H	-2.0124651000000000	-7.4676596999999996	2.7303231000000001
O	1.2141086999999999	-9.0168073000000000	3.1442445999999999
H	1.1642276000000000	-8.3108584000000008	2.4568051000000000
H	2.0027225000000000	-9.5996857999999996	2.7336369000000000
O	-3.0831156000000002	-3.0239037000000000	0.33923418999999999
H	-3.3169854999999999	-2.5686165999999999	-0.44104398000000000
H	-2.1676161000000000	-2.7495707000000000	0.55602803999999995

C	0.16579399E+01	-0.32868547E+01	0.24750892E+01
C	0.18167588E+01	-0.19163540E+01	0.29766156E+01
O	0.17568874E+01	-0.10458593E+01	0.19000986E+01
C	0.15236215E+01	-0.17460771E+01	0.67536595E+00
C	0.16416707E+01	-0.31676548E+01	0.10542024E+01
O	0.18502000E+01	-0.14534965E+01	0.41198936E+01
C	0.12578802E+00	-0.14461836E+01	0.92452470E-01
O	-0.96557278E+00	-0.14975987E+01	0.10620773E+01
O	1.6597497000000001	-4.0695274000000001	7.8781749999999998E-002
O	1.5468683000000001	-4.3338118999999997	3.3033207000000000
C	0.18782788E+00	-0.49041454E-02	-0.50609505E+00
O	-0.10326978E+01	0.31033650E+00	-0.10322346E+01
H	0.22925681E+01	-0.14362316E+01	0.29579084E-01
H	0.37537503E+00	0.65789552E+00	0.30993547E+00
H	0.96544118E+00	0.24597934E-01	-0.12911228E+01
H	-0.17583072E+01	0.20259650E+00	-0.36646624E+00
H	-0.15377074E+00	-0.21026551E+01	-0.65904997E+00
H	-0.51578517E+00	-0.14518932E+01	0.19148752E+01
O	1.2087014000000000	-6.5381407999999999	1.6420461000000000
H	1.2276467000000000	-5.9996793999999998	2.4541510000000000
H	1.5278081999999999	-5.8177301999999997	1.0468575000000000
O	4.2923251999999996	-4.6206690999999998	0.71084336000000004
H	4.1627470000000004	-4.9006055999999996	1.6041850000000000
H	3.4056402000000001	-4.2614470999999998	0.45363428000000000
O	3.9180476999999998	-5.4873941999999998	3.2929586000000000
H	4.2896846000000002	-5.1245927000000000	4.0571770999999996
H	2.9488083000000000	-5.2708899999999996	3.3158287000000000
O	-0.94437565000000001	-5.2856047999999998	-0.25728441000000002
H	-1.8685678000000001E-002	-5.1951003000000000	2.0833477000000000E-002
H	-1.1438417000000001	-6.0286805000000001	0.38262429999999997
O	-1.0884053000000000	-4.9409368000000002	3.8566636000000001
H	-0.11260129000000001	-4.8135430000000001	3.5884922000000001
H	-1.2589192000000000	-5.5840950999999999	3.0987917000000000
O	0.51941004000000002	-3.5456637000000000	-2.5336772000000001
H	0.94077507999999999	-4.3156105000000000	-2.9501037000000001
H	0.75308845000000002	-3.7245189999999999	-1.6161894999999999
O	4.1099582999999997	-3.4298986999999999	-1.7195361000000000
H	3.2393030000000000	-3.6663591000000002	-1.3872812999999999
H	4.5062816999999997	-3.0103922000000001	-0.92818409999999996
O	1.3276439000000000	-4.4495474000000002	6.1587005000000001
H	2.2888343000000000	-4.4924727999999998	6.2714805000000000
H	1.3042627000000000	-4.2826472000000004	5.2093261999999996
O	4.4962999000000003	-7.1602703999999999	-0.59780672000000001
H	4.1793034000000002	-6.4875223999999996	8.1971137999999999E-002

H	5.2627506000000004	-6.6907624999999999	-0.99813627000000005
O	6.2364075000000003	-3.6797764000000002	2.4057556999999998
H	5.6078951000000004	-3.8521314000000002	1.6939347000000000
H	6.3686322000000004	-4.5676335000000003	2.8105375000000001
O	-1.5237141000000001	-7.1238529000000002	1.6505627000000000
H	-0.67551775999999997	-7.5168600000000003	1.8490977000000000
H	-2.1929219999999998	-7.6088427999999997	2.1917216000000002
O	1.3858330000000001	-8.8711584999999999	3.2738440000000000
H	1.3996820000000001	-8.1317339000000004	2.6058490999999999
H	2.2115881000000002	-9.3193640000000002	2.9686281999999999

C	0.15105499E+01	-0.32443828E+01	0.26064736E+01
C	0.16990512E+01	-0.19408369E+01	0.31782256E+01
O	0.15975459E+01	-0.94476619E+00	0.21922342E+01
C	0.15739139E+01	-0.15728235E+01	0.88730602E+00
C	0.15940519E+01	-0.30795837E+01	0.11929434E+01
O	0.18171574E+01	-0.15051904E+01	0.43496643E+01
C	0.21905475E+00	-0.12639742E+01	0.20532846E+00
O	-0.86227089E+00	-0.15183285E+01	0.10612328E+01
O	1.6891331000000001	-3.9328723000000001	0.24200289000000000
O	1.4091681000000000	-4.3097782999999996	3.3632536000000002
C	0.15825870E+00	0.25394053E+00	-0.25821701E+00
O	-0.93420843E+00	0.40723078E+00	-0.11890956E+01
H	0.24434733E+01	-0.13542067E+01	0.26781727E+00
H	0.20336763E+00	0.10453018E+01	0.48704482E+00
H	0.10927540E+01	0.42552240E+00	-0.81324728E+00
H	-0.17548588E+01	0.12222943E+00	-0.68030928E+00
H	0.99693525E-01	-0.17452473E+01	-0.73699193E+00
H	-0.61003493E+00	-0.15269716E+01	0.20340987E+01
O	1.3120660000000000	-6.3837751000000003	1.8687206000000001
H	1.3014036000000000	-5.6244475999999999	2.5124564999999999
H	1.5734562000000001	-5.8407963000000001	1.1725660000000000
O	4.1916595000000001	-4.9420295000000003	0.87785175000000004
H	3.8874930999999999	-5.0910067999999997	1.7626596000000001
H	3.2576724000000001	-4.6114268999999997	0.62791501999999999
O	3.8285070999999999	-5.2660803999999999	3.6293785999999999
H	4.1432976000000004	-4.5198461999999999	4.2320802999999998
H	2.8456758000000000	-5.0834333000000003	3.4866780999999998
O	-1.2358579999999999	-5.2460839000000004	3.6611348000000001
H	-0.33990988000000000	-4.9260126000000000	3.3882191000000002
H	-1.4235468000000000	-5.9068157000000001	2.9898028999999999
O	-0.96260520999999999	-5.3607703999999998	-0.43147655000000001
H	-0.10177946000000000	-5.1363940000000001	-2.7034656000000001E-002
H	-1.2463120000000001	-6.0790744999999999	0.21765298000000000
O	1.3427004000000000	-4.0825506000000003	5.9578844999999996
H	2.1733248999999999	-4.5192418999999999	6.1388951000000000
H	1.3475193999999999	-4.1278094999999997	4.9868446999999998
O	3.7752555999999999	-3.2626267000000002	-1.7325771000000001
H	3.0042496000000001	-3.5444273000000002	-1.2013408999999999
H	4.3086045000000004	-2.7782461999999999	-1.1255160000000000
O	0.45116708000000000	-3.4341238000000001	-2.6355197000000001
H	1.1142010000000000	-3.8706502999999999	-3.2249154000000000
H	0.69053123000000005	-3.8595036000000000	-1.7626567000000000
O	3.9868549999999998	-3.4761926999999999	5.5225302000000003
H	3.0852762999999999	-3.3237686000000002	5.1600390999999997
H	4.2679577999999996	-2.5522783000000002	5.6116653999999997
O	-1.1349020999999999	-2.2749335999999998	4.8268483000000000
H	-1.1478347000000000	-3.2218949000000001	4.6577469999999996
H	-0.20048326000000000	-2.0774468000000001	4.6731047999999999
O	1.3256178000000001	-8.8745700999999997	3.2160462999999999
H	1.3364119999999999	-7.9780404000000003	2.8749937000000001
H	2.0180994999999999	-9.2440668000000006	2.6740629999999999
O	4.3616552999999998	-7.2556938000000004	-0.53040381999999997
H	4.1864204999999997	-6.2667954000000003	-0.35676898000000001
H	4.8740816000000002	-7.3542696999999997	0.29290156000000001

C	0.15157770E+01	-0.33074633E+01	0.26855336E+01
C	0.17023073E+01	-0.18763114E+01	0.31529444E+01
O	0.16319375E+01	-0.10254394E+01	0.20958922E+01
C	0.15684757E+01	-0.17337873E+01	0.82420036E+00
C	0.15512647E+01	-0.31830480E+01	0.12596751E+01
O	0.18313202E+01	-0.14021944E+01	0.43082776E+01
C	0.30377997E+00	-0.12578918E+01	0.96087325E-01
O	-0.91391346E+00	-0.17156838E+01	0.76957509E+00
O	1.5217159000000000	-4.1387887000000001	0.29285106000000000
O	1.5018138000000001	-4.3695995999999999	3.5306955000000002

C	0.34053405E+00	0.20428599E+00	-0.10130456E+00
O	-0.90222195E+00	0.54414672E+00	-0.79080484E+00
H	0.24394351E+01	-0.13713686E+01	0.25788775E+00
H	0.27463775E+00	0.74427732E+00	0.90473652E+00
H	0.11764860E+01	0.54724426E+00	-0.70361034E+00
H	-0.16058572E+01	0.61069964E-01	-0.34176223E+00
H	0.29350359E+00	-0.17103166E+01	-0.91068062E+00
H	-0.79774878E+00	-0.19127352E+01	0.17715092E+01
O	1.8770781000000001	-6.5244841999999998	1.7320907999999999
H	1.7960035000000001	-5.9554482000000002	2.5087000000000002
H	1.8992015000000000	-5.8239616999999999	1.0518797000000000
O	4.3784460000000003	-4.7440163999999996	1.2392192000000000
H	3.9558472999999998	-5.4926936000000000	1.7615784000000001
H	3.5812392000000002	-4.4951730000000003	0.76585862000000005
O	3.8923958000000001	-5.5248495999999996	4.0003612999999998
H	4.4269249000000004	-4.7555006999999998	4.4000135000000000
H	3.0810431000000000	-4.9965191000000004	3.7220787000000000
O	-1.0804043000000001	-5.4597302000000001	3.8287148000000002
H	-9.8712539000000002E-002	-5.3765507000000001	3.6778510999999998
H	-1.3562528000000000	-5.9497416999999997	3.0598887000000001
O	-0.97944129000000002	-5.2300373000000002	-0.41381282000000003
H	-0.10324375000000000	-5.0331948999999998	-3.1565432999999997E-002
H	-1.2836350000000001	-5.9347035000000004	0.21651751999999999
O	3.5849443999999999	-3.1123406000000000	-1.3651708000000000
H	2.9296467000000002	-3.2786878000000002	-0.68061042000000005
H	4.0267410000000003	-2.4059773999999998	-0.88578992999999995
O	-0.96708309999999997	-2.6859893000000001	4.8396338999999999
H	-0.80716549999999998	-3.5126496999999999	4.3298809000000000
H	-6.9845163000000002E-002	-2.4703051000000000	4.9915867000000000
O	1.5798582999999999	-4.4039529000000002	6.3017909999999997
H	2.1280659000000002	-5.1888529999999999	6.1128704000000003
H	1.3149493000000001	-4.2685143999999999	5.3590470999999997
O	0.86468255000000005	-3.4524172000000002	-2.5568276000000001
H	1.5998899000000000	-3.9406593999999999	-2.9062139000000000
H	0.90969213000000004	-3.7681651999999999	-1.6265889000000000
O	3.9144304000000001	-7.0756553999999996	-0.58437967000000002
H	3.3464467000000000	-6.9267159999999999	0.21176033999999999
H	4.7677671999999998	-7.2600218999999999	-0.23683429000000000
O	1.4328959999999999	-8.6290558999999991	3.4264716000000002
H	1.7541635000000000	-7.8339936000000003	2.9768189999999999
H	1.9128706000000000	-9.2655869000000006	2.8968340000000001
O	4.2687033000000003	-3.2257603000000001	5.5030231000000001
H	3.3208628999999998	-3.2210497999999999	5.4481431999999996
H	4.4126475999999997	-2.4960410000000000	6.1744326999999997

C	0.16888298E+01	-0.32565825E+01	0.25625045E+01
C	0.16794285E+01	-0.19219012E+01	0.32017489E+01
O	0.16061273E+01	-0.93546956E+00	0.22784840E+01
C	0.14525264E+01	-0.15346256E+01	0.91779883E+00
C	0.14854087E+01	-0.30791256E+01	0.11783066E+01
O	0.17168852E+01	-0.16657862E+01	0.44187640E+01
C	0.14193906E+00	-0.11477384E+01	0.29960819E+00
O	-0.98865256E+00	-0.16805685E+01	0.10025597E+01
O	1.4760275000000000	-3.9637916000000000	0.23359288000000000
O	1.6440405000000000	-4.3346584999999997	3.3601752000000000
C	0.21140425E-01	0.38622703E+00	0.16941869E+00
O	-0.97467650E+00	0.87298278E+00	-0.70783204E+00
H	0.23090388E+01	-0.12209476E+01	0.37734982E+00
H	-0.94858064E-01	0.81113715E+00	0.11523804E+01
H	0.95414825E+00	0.77423044E+00	-0.19604772E+00
H	-0.17554050E+01	0.78808920E+00	-0.24121295E+00
H	0.26374438E+00	-0.15422003E+01	-0.73509788E+00
H	-0.69798635E+00	-0.23654992E+01	0.16195664E+01
O	1.8367434000000000	-6.3992623000000002	1.6984222000000000
H	1.7904944000000000	-5.5917447999999998	2.3361402000000000
H	1.7630988999999999	-5.8366055000000001	0.92600415000000003
O	4.0054689000000003	-5.6971752000000002	3.4195538000000001
H	4.3583385999999997	-4.8513653999999997	3.8632659000000000
H	3.0075265000000000	-5.4557317000000003	3.4437063000000001
O	-0.99283420000000000	-5.1035953000000003	-0.40577348000000002
H	-0.17381693000000001	-4.8187981000000004	1.5378085000000000E-002
H	-1.3153923000000001	-5.7700304999999998	0.16592804000000000
O	-1.0533553000000000	-5.2406338000000003	3.7525921000000002
H	-0.13704864000000000	-4.8671077000000000	3.7108683999999998
H	-1.0820372000000000	-5.8726513000000002	2.9870964999999998
O	4.7807382000000000	-4.7272501000000000	0.90762810000000005

H	4.3351984000000003	-5.1132381000000002	1.7299526000000001
H	4.0284478000000004	-4.5367440999999999	0.35758063000000001
O	3.5949874999999998	-3.0831178000000001	-1.4559994999999999
H	2.8267193000000002	-3.3392138999999998	-0.95443721999999998
H	4.0768221000000002	-2.5683012000000001	-0.77393467000000005
O	1.3360040000000000	-4.6164692000000001	6.1262622999999996
H	2.1288387000000002	-5.0274912000000000	6.4746223000000001
H	1.5189733999999999	-4.6141762999999996	5.1770280000000000
O	0.86161757999999999	-3.2029437000000001	-2.4947263000000000
H	1.3162543000000000	-3.9234963999999999	-3.0084124000000001
H	0.95957161000000002	-3.5549558000000001	-1.5991865999999999
O	1.2305667000000000	-8.6456719000000000	3.3068420000000001
H	1.3497869000000000	-7.7624047999999997	2.8795123000000000
H	1.9180881999999999	-9.1020371999999998	2.8898609999999998
O	-0.97158294000000001	-2.5601509999999998	4.6059171000000001
H	-1.1614241999999999	-3.4947561000000000	4.4027962000000000
H	-4.6074316999999997E-002	-2.5336922999999998	4.9471134000000001
O	4.1371564999999997	-3.3512054000000000	5.3155747000000000
H	3.3122954999999998	-2.9007839000000000	5.0184053000000004
H	4.3179021000000004	-2.7190702000000000	6.0188122999999996
O	-1.3083313999999999	-6.9696021999999997	1.6048137000000000
H	-0.58572928999999996	-7.6268110000000000	1.4376848000000000
H	-2.0971142999999999	-7.4680206000000000	1.8712629000000001

C	0.17519745E+01	-0.32884620E+01	0.25457687E+01
C	0.17583701E+01	-0.20097804E+01	0.31312196E+01
O	0.16125219E+01	-0.96718985E+00	0.22098383E+01
C	0.15571217E+01	-0.15414050E+01	0.88700918E+00
C	0.17891280E+01	-0.30150497E+01	0.11293366E+01
O	0.18203013E+01	-0.17603879E+01	0.43489228E+01
C	0.27696503E+00	-0.11308656E+01	0.31000532E+00
O	-0.85038430E+00	-0.15127413E+01	0.11366807E+01
O	1.7581728999999999	-3.9655741999999998	0.23241253000000001
O	1.6602295000000000	-4.4318055000000003	3.1796318000000001
C	0.16904468E+00	0.32583372E+00	0.11239126E+00
O	-0.10357582E+01	0.65646108E+00	-0.62994065E+00
H	0.24061856E+01	-0.10949815E+01	0.35297667E+00
H	0.33013261E-01	0.86330857E+00	0.10688593E+01
H	0.11100243E+01	0.64950591E+00	-0.44674800E+00
H	-0.17415417E+01	0.10008816E+01	-0.56124070E-01
H	0.16397196E+00	-0.16551054E+01	-0.58269832E+00
H	-0.55441797E+00	-0.18326531E+01	0.19975519E+01
O	1.9525680000000001	-6.5301159000000002	1.3847339000000001
H	1.6579522000000000	-5.9073780999999999	2.0398399999999999
H	2.0581569000000002	-5.9624841000000002	0.60987617999999999
O	4.6657945999999999	-4.4886144000000003	1.1685460999999999
H	3.9514265000000002	-4.8702230000000002	1.7784466999999999
H	4.1721577999999999	-4.4433338999999998	0.30865196000000000
O	4.1087084999999997	-5.4979727000000000	3.6942840000000001
H	4.5514562999999999	-4.8969187999999999	4.2795896999999998
H	3.2723369999999998	-5.0215012000000003	3.4594086000000002
O	-0.86915949999999997	-5.3530914000000003	-0.25872432000000001
H	2.8990417000000001E-002	-4.9730087000000003	-0.10819328000000000
H	-1.0505183000000000	-5.7579723999999999	0.60983582999999997
O	-0.98740516000000000	-5.2913397000000000	3.5312679999999999
H	1.6204887000000001E-002	-5.3251571000000002	3.3746016000000001
H	-1.2516152000000000	-5.8456215000000000	2.8219732999999998
O	3.9107210000000001	-3.4487530000000000	-1.5193793000000000
H	3.0139298999999999	-3.4739004000000002	-1.1126822000000001
H	4.2888520000000003	-2.8256076000000001	-0.88468621000000003
O	0.70073204000000000	-3.4616514000000000	-2.4286186000000001
H	1.3294808000000000	-3.9997821999999998	-2.9819914000000001
H	1.0221330000000000	-3.6902875000000002	-1.5099199999999999
O	1.3700672000000000	-4.6046376000000002	6.0838293999999999
H	2.0462817000000002	-5.2495618000000004	6.3510149000000000
H	1.6515480000000000	-4.4853605999999999	5.1649688999999999
O	1.3178688999999999	-8.3917704000000004	3.2417562000000002
H	1.3977975000000000	-7.8092136999999999	2.4880171999999998
H	1.9037542999999999	-9.1082555000000003	3.0773313999999998
O	-1.1407255000000001	-2.8602319000000000	4.6655756000000004
H	-1.2003104000000000	-3.7544281000000002	4.2052860000000001
H	-0.20046736000000001	-2.8075190000000001	4.9532800000000003
O	4.1685812999999996	-3.2536927000000002	5.4901279000000001
H	3.5059741999999998	-2.8638005999999998	4.9253137000000002
H	4.1034214999999996	-2.6907717000000001	6.2961904999999998
O	4.2673231999999999	-7.2182399000000004	-0.21797616000000000
H	3.5040271999999999	-7.4233269999999996	0.33643120999999998
H	4.9527368000000003	-7.4393976000000004	0.39196646000000002

C	0.15483187E+01	-0.33747145E+01	0.27016728E+01
C	0.17090685E+01	-0.20596923E+01	0.34041706E+01
O	0.16359573E+01	-0.10979792E+01	0.24359403E+01
C	0.14752693E+01	-0.16427269E+01	0.11213146E+01
C	0.15544418E+01	-0.30904688E+01	0.13049437E+01
O	0.17821660E+01	-0.17745815E+01	0.46335505E+01
C	0.11440298E+00	-0.10508081E+01	0.54188130E+00
O	-0.91629883E+00	-0.14247355E+01	0.14775118E+01
O	1.6529190000000000	-3.9453198000000000	0.32888895000000001
O	1.5796804000000000	-4.5831473999999996	3.2068368999999999
C	0.18544692E-01	0.37890887E+00	0.19080207E+00
O	-0.11241884E+01	0.65162413E+00	-0.56048495E+00
H	0.22653015E+01	-0.11546879E+01	0.45292733E+00
H	0.17393530E-01	0.90500852E+00	0.11241747E+01
H	0.81357558E+00	0.72879998E+00	-0.45238415E+00
H	-0.18906237E+01	0.29434530E+00	-0.20127393E-01
H	-0.33611490E-01	-0.16906582E+01	-0.35037096E+00
H	-0.51591539E+00	-0.20047498E+01	0.21041059E+01
O	2.0803856000000001	-6.5589664000000001	1.3788828000000000
H	2.0626091999999998	-6.2764565000000001	2.3560821000000001
H	1.8663215000000000	-5.6651718000000004	1.0007275000000000
O	-0.89603668999999997	-5.2130280000000004	-8.9554815999999995E-002
H	7.9200671000000000E-002	-5.1598895000000002	0.14792701999999999
H	-1.1500946999999999	-5.7018680000000002	0.67866645999999997
O	4.0215623999999996	-5.3544388999999999	3.8251998000000000
H	4.3151295999999997	-4.6655610999999997	4.4888310999999996
H	3.0951403000000002	-5.0583584999999998	3.7001023000000002
O	4.6846182000000001	-4.1797187999999998	1.1847369000000001
H	4.4133103000000000	-4.5184593000000000	2.0313918000000002
H	3.9797009000000001	-4.4353598999999999	0.54649950999999997
O	-1.0818380999999999	-5.4298698999999999	3.6386801000000002
H	-8.3133472000000000E-002	-5.4287451000000004	3.4737670999999999
H	-1.3700068000000001	-6.0121883000000000	2.8978025000000001
O	3.5719408000000001	-3.5877997000000001	-1.5384918000000001
H	2.8925385000000001	-3.6618426999999998	-0.88645710000000000
H	4.0940021000000000	-2.9502578000000002	-0.97384444999999997
O	0.77335434999999997	-3.5492830999999998	-2.1829952000000001
H	1.3383483000000000	-4.0527319000000004	-2.7740355999999999
H	1.2587774000000000	-3.5820424000000002	-1.3763723000000001
O	1.5227434000000000	-4.2670497999999997	6.1381294000000004
H	2.1552321999999999	-4.8961857000000002	6.4426081000000002
H	1.5443568999999999	-4.4567002999999996	5.1554497000000001
O	-1.0545513000000000	-2.7867348999999999	4.8226189000000002
H	-1.0363606999999999	-3.5759726000000001	4.2215900000000000
H	-8.9231560000000001E-002	-2.6726219000000002	4.9324254999999999
O	4.4744641999999999	-7.0777448999999999	-0.43990812000000001
H	3.7149530999999998	-6.9218080000000004	0.14012622000000000
H	4.9030918999999997	-7.7458403999999996	0.10611286000000000
O	1.4723970000000000	-8.6781579000000004	2.9762751000000001
H	1.9166745000000001	-7.9814502000000003	2.4303417000000000
H	2.1380300000000001	-9.3575500999999992	2.9999794999999998
O	4.3834507000000000	-3.0880112999999998	5.5177768000000000
H	3.4095412000000000	-2.9890663000000002	5.2872966999999997
H	4.4633792999999997	-2.4184543000000001	6.2886014000000001

C	0.15506501E+01	-0.35051760E+01	0.26782111E+01
C	0.15213863E+01	-0.22124750E+01	0.33464953E+01
O	0.16148934E+01	-0.11208366E+01	0.24333501E+01
C	0.15113740E+01	-0.17192408E+01	0.11371520E+01
C	0.16044554E+01	-0.32000468E+01	0.13022617E+01
O	0.14953266E+01	-0.19623184E+01	0.45946547E+01
C	0.15344018E+00	-0.13163197E+01	0.51515910E+00
O	-0.99788032E+00	-0.16570645E+01	0.13603380E+01
O	1.7651332000000000	-3.9915403000000000	0.30470638000000000
O	1.6734142000000001	-4.6985280999999999	3.2227926999999998
C	0.12932069E+00	0.18405185E+00	0.36461593E+00
O	-0.92650081E+00	0.81438884E+00	-0.42593312E+00
H	0.22486046E+01	-0.13488583E+01	0.39033941E+00
H	0.72524018E-01	0.61732641E+00	0.13724034E+01
H	0.10452457E+01	0.40559829E+00	-0.23092940E+00
H	-0.16963994E+01	0.15081617E+00	-0.27571068E+00
H	0.96523946E-01	-0.19893633E+01	-0.35361004E+00
H	-0.85208973E+00	-0.25353273E+01	0.17930459E+01

O	1.8681633000000000	-6.4596261999999998	1.1262460999999999
H	1.8223965000000000	-5.9310793999999998	1.9450631000000000
H	1.9032794000000000	-5.7514966999999997	0.49440342999999998
O	-1.0681149999999999	-5.1168874000000004	0.28965232000000002
H	-0.14474486000000000	-5.2115980999999998	0.58510114000000002
H	-1.4746847999999999	-5.7063898999999996	0.93150937000000000
O	4.8384958999999998	-4.3683015000000003	1.3548886000000000
H	4.5256002999999998	-4.9863352000000001	2.0495123999999998
H	4.0313458000000004	-3.8350095999999998	1.1875852000000000
O	4.1181726000000003	-5.2210416999999998	3.9667048999999999
H	4.2302657000000004	-4.3078759000000000	4.2280905000000004
H	3.1673252000000001	-5.1245548000000003	3.5794291999999999
O	-1.0471600999999999	-5.2156206999999997	3.4990649000000000
H	-0.14452008000000000	-5.2770238999999997	3.1928084000000001
H	-1.4596625000000001	-5.9750952000000002	3.0850749999999998
O	3.6087319999999998	-3.2269899000000000	-1.6618278000000000
H	3.0075485999999998	-3.6941929000000000	-1.1087115000000000
H	4.1189372999999998	-2.6897872999999999	-1.0298312000000001
O	1.3976729000000001	-4.6259747000000004	6.0515990999999998
H	2.0866845999999999	-5.2622720000000003	6.1824361000000003
H	1.5030304999999999	-4.6132600999999998	5.0651380000000001
O	0.75315748000000005	-3.4202170000000001	-2.3221303999999998
H	1.2664419000000000	-3.8667945000000001	-3.0308136000000001
H	1.1564483000000001	-3.8355855000000001	-1.5267157000000000
O	1.4821005000000000	-8.5136856000000005	2.8669140999999998
H	1.3828971999999999	-7.8729442000000001	2.1533334000000002
H	2.4322187999999998	-8.8282603000000002	2.9002837000000001
O	4.2072183000000001	-7.2340695999999998	-0.53483714999999998
H	3.5495079000000000	-6.9348739999999998	7.4499976999999995E-002
H	5.0071333999999998	-7.1632036000000001	-9.6970171999999997E-003
O	-1.2690564000000000	-2.7890815999999998	4.7295642999999998
H	-1.0761896000000000	-3.4464009000000000	4.0571839000000001
H	-0.43201034999999999	-2.3035923000000000	4.8152280999999997
O	5.3523904000000000	-7.3300286999999997	2.7788455999999999
H	4.9772017999999996	-6.4885885999999999	3.1789502000000001
H	6.3146513000000004	-7.1073130000000004	2.8492324999999998

C	0.14503695E+01	-0.33998685E+01	0.26051323E+01
C	0.16150657E+01	-0.21456922E+01	0.33589209E+01
O	0.17327150E+01	-0.11081148E+01	0.24997411E+01
C	0.15350080E+01	-0.15912319E+01	0.10993454E+01
C	0.16315317E+01	-0.30865102E+01	0.12514258E+01
O	0.17796143E+01	-0.19106153E+01	0.45596217E+01
C	0.27507257E+00	-0.11351878E+01	0.45014312E+00
O	-0.82199073E+00	-0.15606417E+01	0.13066348E+01
O	1.6894828000000000	-3.8915456000000002	0.22889406000000001
O	1.3682190000000001	-4.5860291000000002	3.2250076999999999
C	0.23775259E+00	0.46285814E+00	0.37156087E+00
O	-0.94359845E+00	0.85846393E+00	-0.26188922E+00
H	0.23016497E+01	-0.11704121E+01	0.50977090E+00
H	0.33255195E+00	0.99709323E+00	0.13381281E+01
H	0.11306565E+01	0.73251766E+00	-0.25755740E+00
H	-0.16942285E+01	0.28343462E+00	-0.30060106E-01
H	0.22015150E+00	-0.15413295E+01	-0.54943516E+00
H	-0.59856934E+00	-0.12824561E+01	0.22063523E+01
O	2.1567194999999999	-6.4114599999999999	1.2888393000000000
H	1.8673181000000001	-5.8910353000000004	2.0300319999999998
H	1.9695749000000000	-5.7846736999999999	0.64144482999999997
O	-0.93554172999999996	-5.2556776999999997	0.12546811999999999
H	1.6536808000000000E-002	-4.9456357999999998	0.34990769999999999
H	-1.1042330000000000	-5.8563663000000004	0.86195922999999997
O	4.7180793000000003	-4.4666889999999997	1.3009633000000000
H	4.2402381000000000	-4.8994913000000002	0.57225908000000003
H	3.9842437999999998	-3.8869448000000002	1.6567722000000000
O	-1.1908327000000001	-5.2935302999999996	3.5805739999999999
H	-0.23092443000000001	-5.1035814000000004	3.5274820999999998
H	-1.3138755000000000	-5.9865487999999996	2.9158428999999999
O	3.7687365000000002	-5.1454560999999996	4.3900271999999996
H	3.9876109000000000	-4.2154056999999998	4.5214685000000001
H	2.9323937999999998	-5.1025998000000001	3.9133838000000001
O	0.66830871999999997	-3.8028336000000000	-2.2450614999999998
H	1.4236104000000001	-4.2845145999999996	-2.7039236000000000
H	0.94911677999999999	-3.8660494000000001	-1.2757886000000001
O	3.6253617000000000	-3.2452511999999998	-1.6121521000000001
H	2.8588624999999999	-3.4675913999999999	-0.96695582000000002
H	4.0813560000000004	-2.5898034000000001	-1.1042479999999999

O	1.2051371000000000	-4.6486394000000004	6.0674969000000001
H	2.0437422999999999	-5.1613916000000000	6.1967131000000002
H	1.0961072999999999	-4.4797447000000004	5.1445198999999997
O	1.4220071999999999	-8.4969149999999996	2.8466821000000002
H	1.7965392000000000	-7.7264103999999998	2.3908564999999999
H	2.1203542999999998	-9.1365420999999998	2.8108369000000000
O	4.1240022999999999	-7.2016071999999998	-0.30892034000000002
H	3.4257266000000000	-7.0717113999999999	0.40427764999999999
H	4.9030174999999998	-7.1014052000000003	0.23330550000000000
O	-1.1760614000000000	-2.7859026000000000	4.7973071000000003
H	-1.3153816000000000	-3.7292141000000001	4.4365062999999996
H	-0.27536291000000002	-2.8459477999999998	5.0398693000000003
O	-2.1537381000000000	-3.7098133000000000	-1.6147479000000000
H	-1.3767688000000000	-3.5375549999999998	-2.1473445000000000
H	-1.8022863000000000	-4.2695625000000001	-0.84702012000000004

C	0.15158330E+01	-0.34589808E+01	0.26396849E+01
C	0.16863780E+01	-0.22461615E+01	0.33413523E+01
O	0.18002204E+01	-0.11067104E+01	0.25120278E+01
C	0.17007551E+01	-0.16099246E+01	0.11398238E+01
C	0.16567470E+01	-0.30997499E+01	0.12513009E+01
O	0.17008524E+01	-0.19972147E+01	0.45494520E+01
C	0.30419570E+00	-0.11117393E+01	0.54076210E+00
O	-0.76758367E+00	-0.14542247E+01	0.13870272E+01
O	1.6038599000000000	-3.9453600000000000	0.23388734999999999
O	1.3538349999999999	-4.6992550000000000	3.1703006000000000
C	0.28828249E+00	0.34146934E+00	0.24283456E+00
O	-0.92372000E+00	0.74121302E+00	-0.48791258E+00
H	0.24595902E+01	-0.12385365E+01	0.49534725E+00
H	0.31259439E+00	0.92032315E+00	0.11799387E+01
H	0.11062474E+01	0.50780827E+00	-0.44627086E+00
H	-0.16422394E+01	0.34565694E+00	0.37200514E-01
H	0.18039835E+00	-0.15854332E+01	-0.42486555E+00
H	-0.42245860E+00	-0.19027129E+01	0.22105965E+01
O	2.1871029000000002	-6.4647354999999997	1.3667142999999999
H	1.8878002000000000	-5.9493806999999999	2.1271491000000000
H	1.8603348000000000	-5.8396857999999998	0.64694123999999997
O	-1.1747258000000000	-5.0546575999999996	0.13550688000000000
H	-0.33910794999999999	-4.5981747000000004	0.35756990000000000
H	-1.1333340000000001	-5.7574832999999996	0.87423814000000000
O	-1.2498600000000000	-4.8536573000000001	3.7982691000000002
H	-0.27410665000000001	-4.6712729000000000	3.5581765999999999
H	-1.3178098000000000	-5.6191775000000002	3.1862202000000002
O	4.6040413999999998	-4.4277296000000002	1.0867108999999999
H	4.0774293999999998	-5.2233967999999997	1.2999817000000000
H	3.9345549000000002	-3.7973088000000002	0.76507767999999998
O	3.8522238999999998	-5.3285682000000003	4.0992911999999997
H	4.0335169000000004	-4.4206868999999998	4.3616514999999998
H	2.9838947999999998	-5.3440329999999996	3.7490391000000001
O	0.49861767000000001	-3.5777206000000001	-2.1619055000000000
H	1.2768619999999999	-3.8069535999999999	-2.6783478999999999
H	0.89638746999999996	-3.5963376999999999	-1.2200739000000000
O	3.4385995000000000	-3.2026091999999999	-1.6265605000000001
H	2.8514599999999999	-3.4906527999999999	-0.91732033999999996
H	4.0093094000000002	-2.6696697000000000	-1.0149124000000000
O	1.1400759000000000	-4.4247924000000003	6.1228841000000003
H	1.8297063000000000	-4.8797870999999997	6.4565028000000000
H	1.0673352000000000	-4.6649199000000001	5.1883942000000003
O	4.2240555000000004	-7.4228231999999998	-0.28800229999999999
H	3.3839104999999998	-7.2729983000000002	8.0057606000000003E-002
H	4.7514529000000003	-7.2589513999999999	0.49698753000000001
O	1.2401312000000000	-8.6825796999999998	3.1128135000000001
H	1.4989992999999999	-8.1509087999999998	2.2812481999999998
H	2.1119010999999999	-8.8525250000000000	3.4264983000000000
O	-1.1771558000000000	-2.2313095000000001	4.5489573999999999
H	-1.3522008999999999	-3.1192742999999998	4.3013956000000002
H	-0.27922113999999998	-2.4171414000000002	4.7188799000000001
O	-3.0704202000000000	-2.4974454000000001	0.53250830999999998
H	-2.7659783999999998	-2.5681927999999998	-0.40420429000000002
H	-2.2315358000000001	-2.4884987999999999	0.95151743000000000

C	0.16167241E+01	-0.32651595E+01	0.25896290E+01
C	0.18584688E+01	-0.20122833E+01	0.32135015E+01
O	0.18603033E+01	-0.95091262E+00	0.22522657E+01
C	0.15567084E+01	-0.15331939E+01	0.97245961E+00
C	0.14562771E+01	-0.30507545E+01	0.12407698E+01

O	0.20696705E+01	-0.17356032E+01	0.43811755E+01
C	0.26457452E+00	-0.99713742E+00	0.37141566E+00
O	-0.83363645E+00	-0.12507020E+01	0.13230876E+01
O	1.3575699999999999	-3.8818345999999999	0.21153611999999999
O	1.5354614000000000	-4.3818913000000004	3.3622461000000001
C	0.25954511E+00	0.52851996E+00	0.10753386E+00
O	-0.10399651E+01	0.89386345E+00	-0.28724430E+00
H	0.23890528E+01	-0.12665674E+01	0.30249773E+00
H	0.65365948E+00	0.11252097E+01	0.10428614E+01
H	0.10461547E+01	0.80818720E+00	-0.68984413E+00
H	-0.16891750E+01	0.26824216E+00	-0.11420388E-01
H	-0.35567478E-01	-0.13416916E+01	-0.60092070E+00
H	-0.62999771E+00	-0.19523653E+01	0.19544041E+01
O	1.8066295999999999	-6.2736027999999999	1.3444217000000001
H	1.8988109000000000	-5.5813638000000001	2.0464839000000001
H	1.5500809000000000	-5.7117975999999997	0.61301159000000005
O	4.3423448000000002	-4.3073782999999999	1.0950238000000001
H	3.4882594000000000	-4.2410728999999998	0.59511716000000003
H	4.0367711999999996	-4.4115520000000004	2.0019049000000000
O	-1.2513112000000000	-4.7024027000000004	3.5149173999999999
H	-0.27123320000000001	-4.5725254000000000	3.4149596000000000
H	-1.5177080000000001	-5.4138780999999998	2.8613225999999998
O	3.9491567000000001	-5.2734538000000004	3.6990329000000002
H	4.2335862000000004	-4.4107184000000004	4.0591737999999999
H	2.9510141000000001	-5.0935376000000003	3.6284535999999998
O	-1.1155082000000001	-5.3221993000000003	-0.22012517000000001
H	-0.46572947999999997	-4.7072735999999997	1.9077255000000001E-002
H	-0.98877086000000003	-5.9606029999999999	0.43145257999999997
O	3.3204658999999999	-3.1067616999999998	-1.6666673999999999
H	2.6196690999999999	-3.3268377000000000	-1.1168388000000000
H	3.8645122000000001	-2.5618162000000000	-1.0247440000000001
O	0.61045316000000005	-3.3948121000000002	-2.3203792999999999
H	1.1953503000000001	-4.0303098999999998	-2.6937671000000001
H	0.81164683000000004	-3.3781287000000000	-1.3895995000000001
O	1.0160536000000000	-4.5915134000000002	6.1407286000000001
H	1.7199629999999999	-5.0369549999999998	6.5606115999999997
H	1.3994065000000000	-4.3369906000000000	5.2833107999999998
O	3.9712790999999998	-7.4590091999999997	-6.3492148999999998E-002
H	3.3131816000000001	-6.7457308999999999	0.16221416999999999
H	4.6782918000000002	-7.3278886999999999	0.53822824999999996
O	1.1835975000000001	-8.4955665000000007	2.8152970000000002
H	1.5189847000000001	-7.8477931999999999	2.2694879999999999
H	1.6234313000000000	-9.2987862999999997	2.5732189000000001
O	-1.2868539999999999	-2.1746089000000000	4.7313359000000004
H	-1.5379668000000000	-3.0344057000000002	4.2092698999999998
H	-1.1506787999999999	-2.5935600000000001	5.6057736000000000
O	4.7275860999999999	-2.9570061000000001	5.3214967000000000
H	3.8283393999999999	-2.7221069999999998	5.0247985000000002
H	4.6981466999999997	-2.4868138000000002	6.1469579000000003

C	0.16308611E+01	-0.32315515E+01	0.26305411E+01
C	0.17960257E+01	-0.20255364E+01	0.33343628E+01
O	0.16670772E+01	-0.92998803E+00	0.24725737E+01
C	0.15676646E+01	-0.14282576E+01	0.10525418E+01
C	0.14534264E+01	-0.29619065E+01	0.12435767E+01
O	0.20742300E+01	-0.17955948E+01	0.44851933E+01
C	0.32801033E+00	-0.91096675E+00	0.35485824E+00
O	-0.93488414E+00	-0.13651481E+01	0.94876585E+00
O	1.1335694000000001	-3.7083922000000000	0.22306296000000000
O	1.5742506999999999	-4.3947960999999998	3.3250552000000000
C	0.22408536E+00	0.58831630E+00	0.27834744E+00
O	-0.90520973E+00	0.90833659E+00	-0.54271857E+00
H	0.24462389E+01	-0.11065438E+01	0.52540850E+00
H	0.84940414E-01	0.95605193E+00	0.13476738E+01
H	0.11063059E+01	0.10292185E+01	-0.14864557E+00
H	-0.17108122E+01	0.36742120E+00	-0.50041943E+00
H	0.41628471E+00	-0.13397429E+01	-0.62366326E+00
H	-0.68794503E+00	-0.22740473E+01	0.11767050E+01
O	1.7886711000000000	-6.3413582000000002	1.3790243000000000
H	1.6726006000000000	-5.6199203000000004	2.0276926000000000
H	1.5732375999999999	-5.7514099999999999	0.67347036000000005
O	-1.1693469000000001	-4.8980101999999999	3.3491181000000001
H	-0.19575917000000001	-4.9492598000000001	3.3506738999999999
H	-1.3575226000000000	-5.6818989000000002	2.8120539000000000
O	-1.4098404000000000	-5.2972479000000003	0.21445987000000000
H	-0.51308821000000004	-4.9379052999999997	0.49569453000000002

H	-1.4786824999999999	-6.0057396000000001	0.94848533000000002
O	4.6370243000000002	-4.3059821999999999	1.1932474000000000
H	3.7982659000000001	-3.8740676999999999	0.94692829999999995
H	4.3457071999999997	-4.8054429000000001	1.9314378000000001
O	4.0308999999999999	-5.2369449000000001	3.7026292000000001
H	4.3816531999999997	-4.4003034999999997	4.0813060999999999
H	3.0768325000000001	-4.9525281999999997	3.7649313000000002
O	3.2143259999999998	-3.2078327000000000	-1.5869396000000000
H	2.5154003000000000	-3.4452791999999999	-0.96602104000000000
H	3.7209685000000001	-2.5385515000000001	-1.0683199999999999
O	0.83601563000000001	-4.6643207999999996	5.9211030999999998
H	1.5512077000000000	-5.2858720999999997	6.1394346000000004
H	1.0526895000000001	-4.4757321000000001	5.0171554000000000
O	0.24340637000000001	-3.5281650999999998	-2.3910233999999999
H	1.0437643999999999	-3.5895313999999998	-3.0072464999999999
H	0.61579070999999996	-3.6784924999999999	-1.4770231000000000
O	-1.2613645000000000	-2.3452323000000002	4.5006561999999999
H	-1.3399958000000001	-3.2828721000000001	4.1523360000000000
H	-0.34218955000000001	-2.4681943999999998	4.8059710999999998
O	4.0106283999999999	-7.1528450000000001	-0.14349344000000000
H	3.1895851999999998	-7.1388081999999997	0.40403855999999999
H	4.6854326000000004	-7.1909184000000002	0.52855189000000002
O	1.1294831000000001	-8.8675873000000003	2.6232020999999999
H	1.2163663000000000	-8.0456705999999993	2.1006019000000000
H	2.0599178000000000	-9.0826279999999997	2.9015624999999998
O	4.4978892000000004	-2.9617192000000001	5.1520143999999997
H	3.5304278999999998	-2.6898875000000002	4.9679975000000001
H	4.6091534999999997	-2.3930072000000000	5.9340865000000003

C	0.16237398E+01	-0.33006049E+01	0.26766736E+01
C	0.16454705E+01	-0.20419993E+01	0.33947132E+01
O	0.14830272E+01	-0.10293796E+01	0.24609800E+01
C	0.14433203E+01	-0.15438866E+01	0.11219185E+01
C	0.14291739E+01	-0.30199922E+01	0.13180370E+01
O	0.17359514E+01	-0.17550762E+01	0.45999325E+01
C	0.13929682E+00	-0.10221689E+01	0.38845020E+00
O	-0.10300576E+01	-0.16661406E+01	0.82691377E+00
O	1.4616564999999999	-3.9414278999999999	0.36291373999999998
O	1.6108350000000000	-4.5492977999999997	3.2227079000000001
C	0.60902709E-01	0.53090265E+00	0.45505446E+00
O	-0.10665979E+01	0.99827212E+00	-0.28443739E+00
H	0.23830740E+01	-0.11538286E+01	0.66162760E+00
H	0.41458392E-01	0.97307754E+00	0.14970999E+01
H	0.93910025E+00	0.89510992E+00	0.14495994E-01
H	-0.17422873E+01	0.31251792E+00	-0.10653465E+00
H	0.26770419E+00	-0.12606874E+01	-0.66428330E+00
H	-0.98097005E+00	-0.16209336E+01	0.18447515E+01
O	1.8410010999999999	-6.8027417999999997	1.6187465000000001
H	1.7714204000000000	-6.0034023000000003	2.2056936000000000
H	1.7122801999999999	-6.2792003999999997	0.80817448000000003
O	-1.1020559000000001	-4.8199757999999999	3.3566368000000000
H	-0.12555023000000001	-4.8903695000000003	3.2266477999999998
H	-1.5066698000000001	-5.6613373999999999	2.9510974000000001
O	4.0542845999999999	-5.0795868999999998	3.9333545999999999
H	4.1957284000000001	-4.0887320999999996	4.1547067000000002
H	3.0675881000000000	-4.9795606000000001	3.6193667999999999
O	4.6633509000000002	-4.3087567000000000	1.2252955000000001
H	4.1853892999999998	-3.4505699999999999	1.0455274999999999
H	4.0351138999999998	-4.7479838000000001	1.8391204000000001
O	-1.0510971000000000	-5.4017043999999999	1.2520028000000001E-002
H	-0.43069543999999998	-4.6879626999999999	0.12124169000000000
H	-0.98043166000000004	-5.9517572000000003	0.82684250999999998
O	0.87415416000000001	-4.6250201000000004	5.7232832000000000
H	1.5875117999999999	-5.2741483999999996	5.8664711000000000
H	1.1056097000000000	-4.2543473000000001	4.8280802999999999
O	3.1442792000000002	-3.2081262000000001	-1.6907279000000000
H	2.5884553000000001	-3.6217090000000001	-1.0240149999999999
H	3.7716224000000000	-2.7924834000000001	-1.1056185999999999
O	0.16835241000000001	-3.6678970999999998	-2.2685564999999999
H	0.79834437999999996	-3.4859844999999998	-2.9782454000000000
H	0.59929140999999997	-3.6132970000000002	-1.4296835999999999
O	-1.2813775000000001	-2.2942743000000001	4.2727127999999999
H	-1.4671010000000000	-3.2116400000000001	3.9400053000000002
H	-0.55617404999999998	-2.5114269000000000	4.8833647999999998
O	5.1523800000000000	-6.9791730000000003	2.1680044000000001
H	4.7441114000000004	-6.2575476999999999	2.7015047999999999

H	6.0783263999999999	-6.8101767999999998	2.3439266999999999
O	3.9581567999999998	-7.6526281999999997	-0.11863272000000000
H	3.1943823999999998	-7.6474101000000001	0.49697817000000000
H	4.5551430999999996	-7.1366658999999997	0.47600448000000001
O	1.0185888000000001	-9.3080423000000003	2.3928866000000002
H	0.98945872000000001	-8.4077075000000008	2.0977356000000000
H	1.9400124000000001	-9.2328004000000004	2.6999871999999998

C	0.15346113E+01	-0.32792958E+01	0.26500966E+01
C	0.17250216E+01	-0.19960666E+01	0.32565327E+01
O	0.17405960E+01	-0.94065595E+00	0.23437680E+01
C	0.14988705E+01	-0.15010803E+01	0.10335426E+01
C	0.14855237E+01	-0.30690815E+01	0.12659661E+01
O	0.18762447E+01	-0.18029806E+01	0.45283915E+01
C	0.24428562E+00	-0.93037853E+00	0.40774986E+00
O	-0.95493177E+00	-0.13842759E+01	0.99844098E+00
O	1.3920626000000000	-3.9178757000000002	0.33666394999999999
O	1.5557559999999999	-4.4350567999999999	3.3293680000000001
C	0.16458325E+00	0.58063612E+00	0.27044432E+00
O	-0.84139220E+00	0.92561648E+00	-0.67574801E+00
H	0.24225536E+01	-0.11875622E+01	0.48996413E+00
H	-0.92759949E-01	0.10190393E+01	0.12751946E+01
H	0.10870328E+01	0.94335933E+00	-0.17137510E+00
H	-0.16088078E+01	0.43061928E+00	-0.42050082E+00
H	0.25610823E+00	-0.12391349E+01	-0.66399276E+00
H	-0.10904077E+01	-0.14412189E+01	0.19723499E+01
O	2.0184209000000002	-6.4859539000000002	1.4645835000000000
H	1.9407182000000001	-6.0455538000000004	2.3833017000000001
H	1.6686040000000000	-5.6454428999999999	1.0160746000000000
O	4.4046618000000004	-4.2655241999999998	1.3400911000000000
H	3.5758136000000000	-3.7217387000000000	1.3502683000000000
H	4.2087256000000002	-4.9017679000000003	2.1127487000000000
O	-1.2587481000000000	-4.6302561000000004	3.4697441000000002
H	-0.29469740999999999	-4.4976608999999996	3.3549722000000002
H	-1.4024850000000000	-5.4204977999999997	2.9168213999999999
O	-1.2044637000000000	-5.3896072999999998	-3.1755282000000003E-002
H	-0.44562302999999998	-4.7997731000000003	0.22130326000000000
H	-1.2957074000000000	-5.9426195000000002	0.75408452000000004
O	4.0270564999999996	-5.1271481000000003	3.9144386999999998
H	4.3864067000000002	-4.4051549999999997	4.4480124999999999
H	3.0581999000000000	-4.8511514000000000	3.8509446000000001
O	0.37080503999999997	-3.5782761000000001	-2.0756942999999999
H	1.2099154000000001	-3.4059621000000000	-2.5482285999999998
H	0.81662555000000003	-3.7994669999999999	-1.2147882999999999
O	3.2655864000000001	-3.3758077000000002	-1.7584591000000001
H	2.7024306999999999	-3.8348412999999999	-1.1120577000000000
H	3.8819599000000000	-2.8152634999999999	-1.2080911000000001
O	0.93400159999999999	-4.6227875000000003	5.9938152999999996
H	1.8871572999999999	-4.7338619000000000	6.2543145999999998
H	0.97881116999999995	-4.7468944000000004	5.0217219000000002
O	3.9600686000000000	-7.4047485999999996	-0.46440136999999998
H	3.4394418999999998	-7.2631778999999996	0.37558480999999999
H	4.8375222000000004	-7.1487219000000000	-0.10293861000000000
O	4.3654130999999996	-2.8941862000000000	5.1559714000000003
H	3.4893654999999999	-2.5676275000000000	4.8567057000000000
H	4.4197740000000003	-2.4596008999999999	6.0480457999999997
O	-0.94524699999999995	-2.1168486000000000	4.8213005999999998
H	-1.1750659999999999	-3.0337702000000002	4.5935259000000004
H	-2.0785943000000001E-002	-2.2572573000000000	5.0486389999999997
O	1.1476253999999999	-9.0602189000000006	2.7116134000000001
H	1.1009036999999999	-8.2960784000000007	2.1433819999999999
H	2.0879362000000001	-9.3307909999999996	2.5715387999999999

C	0.15242178E+01	-0.33482575E+01	0.24865492E+01
C	0.17700501E+01	-0.21189977E+01	0.31169397E+01
O	0.16495601E+01	-0.10005662E+01	0.22895477E+01
C	0.14457841E+01	-0.15099791E+01	0.93358124E+00
C	0.12783631E+01	-0.30391604E+01	0.11431537E+01
O	0.18734163E+01	-0.17962243E+01	0.43216516E+01
C	0.13026822E+00	-0.10989120E+01	0.37170658E+00
O	-0.99425687E+00	-0.16209825E+01	0.11180891E+01
O	0.99475796000000005	-3.8574906000000002	0.24617207999999999
O	1.3573656999999999	-4.5017575000000001	3.1212042000000002
C	0.76386873E-01	0.43824180E+00	0.17872733E+00
O	-0.10033300E+01	0.85757242E+00	-0.70940715E+00
H	0.23519088E+01	-0.12181577E+01	0.30380799E+00
H	-0.40917389E-01	0.88878004E+00	0.12013910E+01
H	0.97685740E+00	0.79921219E+00	-0.26404858E+00
H	-0.18166025E+01	0.51431229E+00	-0.26500110E+00
H	0.39060881E-01	-0.15801285E+01	-0.62570212E+00
H	-0.80004832E+00	-0.16108281E+01	0.20252826E+01
O	1.4235509000000000	-6.4686934999999997	1.4397403000000000
H	1.4187592000000000	-5.6620245999999996	2.0410669000000001
H	1.3392461000000000	-5.9690325000000000	0.57937110999999997
O	-1.3590620000000000	-4.3129087000000004	3.4121589000000001
H	-0.38500566000000003	-4.4362522000000002	3.1719767000000001
H	-1.8484037000000000	-5.0078344000000001	2.9578508999999999
O	-1.2617668000000000	-5.5454473000000002	-7.7233359000000003E-003
H	-0.49801725000000002	-5.0680243000000003	0.30278575000000002
H	-1.5009269000000001	-6.0705532000000000	0.76319340000000002
O	4.3692447999999997	-3.8149460999999998	1.5806229999999999
H	3.5874012999999998	-3.2561368000000002	1.7173258000000000
H	4.4661014999999997	-4.0674450000000002	2.4489778000000002
O	3.8389136000000001	-5.5315335000000001	3.9348603999999998
H	3.9456321999999999	-4.6255553000000003	4.3344519999999997
H	2.8752621000000000	-5.4608827000000000	3.7437749000000000
O	0.33457658000000001	-3.7633633000000000	-2.3236287000000000
H	1.0875992999999999	-4.2931695999999997	-2.6353194000000002
H	0.61679908000000006	-3.6613929000000001	-1.3372322000000001
O	3.1475770999999999	-3.2350284000000000	-1.7869653999999999
H	2.5586777000000001	-3.5440437000000000	-1.0871526000000000
H	3.6640834999999998	-2.5432849000000002	-1.2219933000000001
O	1.0064932000000000	-4.5798505000000000	5.9234415000000000
H	1.5833355000000000	-5.2951464000000001	6.1250818000000002
H	1.0672572000000000	-4.4413071000000004	4.9238346999999996
O	3.5145420999999999	-7.4287789999999996	-0.25861339000000000
H	2.7660971999999999	-7.0445365000000004	0.29249681999999999
H	4.1895724000000003	-7.4084231000000003	0.48558594999999999
O	-1.1731946000000000	-1.8783034000000001	4.6022556000000003
H	-1.3452687000000001	-2.7996425999999999	4.2427494000000001
H	-0.23342935000000001	-1.9657309000000001	4.9775479999999996
O	1.0388397000000000	-9.0340624999999992	2.7049669000000001
H	1.1015995999999999	-8.1714921000000000	2.2638780999999999
H	1.9664271000000000	-9.2578227999999996	2.7848445000000002
O	0.13593568000000000	-7.1159299999999996	-1.9223786000000000
H	-0.30078611999999999	-6.6621858999999996	-1.1467870000000000
H	0.62303145000000004	-6.3677124999999997	-2.3417132999999999

C	0.14448477E+01	-0.31846865E+01	0.25827372E+01
C	0.16651311E+01	-0.19634750E+01	0.33100503E+01
O	0.16611525E+01	-0.86245173E+00	0.24169297E+01
C	0.15192537E+01	-0.14490427E+01	0.10924548E+01
C	0.14304997E+01	-0.28920115E+01	0.12205404E+01
O	0.17180648E+01	-0.17235498E+01	0.45313310E+01
C	0.14550683E+00	-0.93183107E+00	0.41938445E+00
O	-0.99133041E+00	-0.12384489E+01	0.12313531E+01
O	1.3007223000000001	-3.7682093000000001	0.24029139999999999
O	1.2689150000000000	-4.4099484999999996	3.1208534999999999
C	0.28795704E+00	0.53802970E+00	0.71951228E-01
O	-0.70881029E+00	0.91321674E+00	-0.81896113E+00
H	0.23192582E+01	-0.10323943E+01	0.44353092E+00
H	0.28164863E+00	0.12009453E+01	0.10127394E+01
H	0.12171940E+01	0.73965970E+00	-0.49384006E+00
H	-0.15619787E+01	0.51370725E+00	-0.56611933E+00
H	0.10033066E+00	-0.15070215E+01	-0.51993537E+00
H	-0.65439398E+00	-0.15212027E+01	0.21343826E+01
O	1.6607456000000000	-6.2975545000000004	0.95439419000000003
H	1.4884130000000000	-5.8909095999999996	1.8604084999999999
H	1.8156314000000000	-5.3780763000000000	0.57204918000000005
O	4.3192981000000001	-3.6649720000000001	1.5130296999999999
H	3.3487380000000000	-3.6401417000000000	1.1760676999999999
H	4.2578591000000001	-4.3082149000000003	2.2397486000000000
O	-1.2007188000000000	-5.4281718999999997	-1.1054108000000000E-002
H	-0.54724152000000004	-4.8098194999999997	0.27547423999999998
H	-1.0633497999999999	-6.1117016000000000	0.66369964000000004
O	-1.7590250999999999	-4.4547596000000000	3.2855935999999999
H	-0.75254966999999995	-4.6096985999999998	3.4056413000000001
H	-2.0313156999999999	-5.1929220000000003	2.6503665000000001
O	3.7613219999999998	-5.1303557000000000	4.1663573999999999
H	3.9108049000000000	-4.3446873000000004	4.6916069000000000
H	2.7952658000000001	-5.1051674000000000	3.9567394000000000
O	3.2054923000000000	-3.3459656999999998	-1.8162313000000001

H	2.5488268999999999	-3.4334783000000000	-1.0828481000000001
H	3.7575126999999999	-2.6559930000000000	-1.3614236000000000
O	-0.12270389000000000	-3.8107147000000001	-2.2449360000000000
H	0.67374500000000004	-4.1104361999999997	-2.6122163999999999
H	0.14518281999999999	-3.6501598999999998	-1.3060006000000000
O	1.0660470000000000	-4.6314750000000000	5.9584118000000004
H	1.6149610000000001	-5.4192822999999999	6.1535076000000002
H	1.2489459999999999	-4.4256469999999997	5.0010881999999999
O	3.5015196000000000	-7.6380609000000002	-0.28467703999999999
H	2.6918749000000002	-7.1327106999999996	3.7080180999999997E-002
H	4.0339184000000001	-7.4117753000000004	0.45573814000000001
O	-3.3034330999999999	-2.9804971999999998	0.80336255000000001
H	-3.3082460999999999	-2.9393394000000002	-0.18443562999999999
H	-2.3968756999999998	-2.6440907000000000	1.0405009000000001
O	1.0822887999999999	-8.7159355000000005	3.1855232000000000
H	0.94638467999999998	-8.0973641999999995	2.4596507999999999
H	2.0125611999999999	-8.8314872999999992	3.2876596000000000
O	-1.3052589000000001	-2.0940249999999998	4.5192794000000003
H	-1.8513991000000001	-2.8012288000000001	4.1004752000000000
H	-0.98374950000000005	-2.5486130000000000	5.3103677999999999

C	0.13260192E+01	-0.34153959E+01	0.26991770E+01
C	0.17466525E+01	-0.21514813E+01	0.33473631E+01
O	0.16821593E+01	-0.11319245E+01	0.24064307E+01
C	0.15193873E+01	-0.16389557E+01	0.11144455E+01
C	0.13550417E+01	-0.31364821E+01	0.13314529E+01
O	0.21042992E+01	-0.18761121E+01	0.44877674E+01
C	0.25917748E+00	-0.10144338E+01	0.45449235E+00
O	-0.90175594E+00	-0.11729058E+01	0.12907069E+01
O	1.2237644999999999	-3.9191698000000001	0.31046918000000001
O	1.2873915000000000	-4.6265761999999997	3.2614212999999999
C	0.39587173E+00	0.46327217E+00	0.16694140E+00
O	-0.63591422E+00	0.10416628E+01	-0.61901060E+00
H	0.24643563E+01	-0.14542219E+01	0.56619322E+00
H	0.45478040E+00	0.10033288E+01	0.11104543E+01
H	0.13320397E+01	0.64533090E+00	-0.43229467E+00
H	-0.14857636E+01	0.63762804E+00	-0.40172708E+00
H	0.11491458E+00	-0.16481136E+01	-0.41536013E+00
H	-0.57202164E+00	-0.90453149E+00	0.21453185E+01
O	1.5362213000000000	-6.4153342999999996	1.1607422000000001
H	1.3511229000000000	-5.9821477999999999	2.0402211000000001
H	1.7778702000000000	-5.5917092000000004	0.67775836000000000
O	-1.2471018000000000	-5.2730091999999997	9.2183888000000005E-002
H	-0.38823291999999998	-4.9197218999999999	0.42521563000000001
H	-1.5080507000000001	-5.8152473000000002	0.87257554999999998
O	4.5686568000000003	-3.8717304000000001	1.3746271000000001
H	4.6272824999999997	-3.1183971000000001	2.0077056999999998
H	3.7466140999999999	-4.1634509000000000	1.7554828000000000
O	3.7758194999999999	-5.0239744000000002	3.9032822000000000
H	3.8646368999999998	-4.1422891000000002	4.3608468000000000
H	2.7983254999999998	-4.9637840000000004	3.6299739999999998
O	-1.6804338999999999	-4.5973750999999998	3.5678448000000000
H	-0.73064395000000004	-4.6657057999999996	3.4472599000000002
H	-1.8947927000000000	-5.3299269999999996	2.9089285999999999
O	2.9598599999999999	-3.0706381999999999	-1.6764996000000001
H	2.3902166999999999	-3.2826914999999999	-0.90007607000000001
H	3.7851431999999998	-2.9092498999999998	-1.1724926000000000
O	0.81625117999999997	-4.5601437000000002	5.8006732000000003
H	1.4062593999999999	-5.2608109000000001	6.0842492000000004
H	1.1824625000000000	-4.4511096999999999	4.8869674999999999
O	4.7992545999999997E-002	-3.9334223000000001	-2.3305946000000000
H	0.92296458000000003	-4.0298527000000002	-2.7847251000000002
H	0.40161300000000000	-3.9243082000000000	-1.3950427999999999
O	4.5579986000000003	-7.0971415000000002	1.9415929999999999
H	4.1486501000000002	-6.3104449999999996	2.3812628999999998
H	5.5058465999999999	-6.8461828999999996	1.8622889000000000
O	1.3202479000000000	-8.3489336000000005	3.3100377000000001
H	1.5139841999999999	-7.7767605000000000	2.5645308000000000
H	2.1885351000000002	-8.7660850999999997	3.4883346999999998
O	3.5067233999999998	-7.7329309999999998	-0.38597152000000001
H	2.6518994000000000	-7.3577624000000004	-1.9577424000000000E-002
H	4.0795750999999996	-7.4692765999999997	0.35761124000000000
O	-1.1455914000000000	-1.8945495000000001	4.8101279000000003
H	-1.2763732000000001	-2.8282289000000000	4.5941796999999998
H	-0.93264248999999999	-1.9235903999999999	5.7471524000000000

C	0.14146099E+01	-0.34071596E+01	0.26390927E+01
C	0.16790736E+01	-0.21903960E+01	0.32973441E+01
O	0.18591705E+01	-0.11484028E+01	0.23712010E+01
C	0.15451510E+01	-0.16576957E+01	0.10304130E+01
C	0.14325951E+01	-0.32010648E+01	0.12297545E+01
O	0.19647488E+01	-0.19754924E+01	0.45062777E+01
C	0.35773707E+00	-0.94869610E+00	0.40705125E+00
O	-0.85231984E+00	-0.11974701E+01	0.12210833E+01
O	1.2887092000000000	-4.0941669000000003	0.30682141000000002
O	1.3276876000000000	-4.5937036000000004	3.2390983000000002
C	0.49429049E+00	0.59237660E+00	0.21948239E+00
O	-0.59273321E+00	0.10771503E+01	-0.61125943E+00
H	0.24279097E+01	-0.14305856E+01	0.37228102E+00
H	0.41429002E+00	0.11190985E+01	0.11224470E+01
H	0.14400254E+01	0.89094313E+00	-0.22647495E+00
H	-0.13938184E+01	0.10135844E+01	-0.10203007E+00
H	0.21191596E+00	-0.13559838E+01	-0.60055048E+00
H	-0.61357923E+00	-0.17720572E+01	0.19216871E+01
O	1.2303785000000000	-6.5802024000000001	1.2915934000000000
H	1.1137123000000000	-6.1025288000000000	2.1419074000000000
H	1.4309426000000001	-5.7061818999999998	0.90947893999999996
O	-1.4458225000000000	-4.8156768000000003	3.5184513000000002
H	-0.51000798000000003	-4.7738725000000004	3.2516213000000000
H	-1.8454904999999999	-5.4583354000000002	2.8204889000000000
O	-1.2070916000000000	-5.5237625000000001	-0.18692022000000000
H	-0.42470407999999998	-5.0227976999999999	0.11453099999999999
H	-1.5307527000000001	-6.0844136999999998	0.57912220999999997
O	3.8848052000000002	-5.2166952000000002	3.7112017000000002
H	4.0829908000000001	-4.3581070999999998	4.1895699999999998
H	2.8888321000000001	-5.1293803999999996	3.6958457000000000
O	4.6701762000000002	-3.9792293000000001	1.2944363999999999
H	4.1741406999999997	-4.2514963000000003	2.0876209000000001
H	4.1249804000000001	-3.2033323999999999	1.1009028000000001
O	2.9297342999999998	-2.8707365999999999	-1.7237107000000000
H	2.4651188999999998	-3.4910814999999999	-1.1139827000000000
H	3.6879637999999999	-2.6273898000000000	-1.1287020000000001
O	1.0228105000000001	-4.7214393000000001	5.9264608000000001
H	1.7076990000000001	-5.3588221000000003	6.0804425999999996
H	1.0520389999999999	-4.5751822000000004	4.9887287000000002
O	0.20100978000000000	-3.6430418000000002	-2.1787635000000001
H	0.81463821999999997	-4.2987526999999996	-2.4707219000000000
H	0.39107121000000000	-3.6371812000000001	-1.2562472000000000
O	3.2612165000000002	-7.4359802999999998	-0.35611555000000000
H	2.3414126999999998	-7.4492858000000002	-1.2477401000000001E-002
H	3.7730448999999999	-7.1199165000000004	0.36195154000000002
O	1.1576417000000001	-8.8601030999999999	3.2281487000000002
H	1.1609721000000000	-8.0132282999999997	2.6837023000000002
H	1.9410741000000000	-9.3148458999999999	2.8525684000000000
O	4.6019268000000002	-7.1784140000000001	2.0620235000000000
H	4.4906796000000000	-6.2843619999999998	2.4316518999999999
H	5.5884480999999999	-7.1366759000000002	1.8405130000000001
O	-1.3023845000000001	-2.1964901000000001	4.5282283000000003
H	-1.8256186000000001	-2.9872429999999999	4.2339054000000003
H	-0.71560279999999998	-2.6203379000000000	5.2310127000000000

C	0.15107713E+01	-0.33491638E+01	0.27139394E+01
C	0.17610736E+01	-0.20531694E+01	0.32342887E+01
O	0.18689340E+01	-0.10618562E+01	0.23372738E+01
C	0.15613790E+01	-0.16884598E+01	0.10047942E+01
C	0.15165973E+01	-0.32000799E+01	0.13376109E+01
O	0.19183672E+01	-0.17117382E+01	0.44686841E+01
C	0.26814784E+00	-0.10242815E+01	0.42183047E+00
O	-0.92986213E+00	-0.12255276E+01	0.12823213E+01
O	1.5552931000000001	-4.1002691999999996	0.40758105000000000
O	1.3287051000000001	-4.5314629000000002	3.3870854000000001
C	0.49399444E+00	0.45470527E+00	0.31865045E+00
O	-0.35813549E+00	0.11729370E+01	-0.52935337E+00
H	0.23623535E+01	-0.13913683E+01	0.26873045E+00
H	0.46447220E+00	0.88186440E+00	0.13302932E+01
H	0.14894145E+01	0.54607779E+00	-0.13787054E+00
H	-0.12179654E+01	0.75326254E+00	-0.37386812E+00
H	0.17987829E+00	-0.14898691E+01	-0.53126090E+00
H	-0.73577737E+00	-0.14633063E+01	0.22036432E+01
O	1.3197357000000001	-6.5794772999999998	1.8202691000000000
H	1.2316638000000000	-5.7787082999999999	2.4146789000000002
H	1.0948891999999999	-6.0947658999999996	1.0028459000000001

O	4.6578669000000001	-4.3729880999999997	1.3960136999999999
H	4.6627080999999997	-4.7253515000000004	2.3032430000000002
H	3.7212524000000000	-4.3886573999999996	1.1831670999999999
O	3.7165564999999998	-5.3573126999999996	4.0799572000000000
H	3.7743916000000000	-4.4652399999999997	4.3852614000000001
H	2.8001296999999998	-5.2387641000000000	3.8333267000000002
O	-1.4086183000000001	-5.4951943999999999	0.41478631999999999
H	-0.80537062999999998	-4.8940020999999998	0.83459068000000003
H	-1.6554721999999999	-6.2000804000000000	1.1196254999999999
O	-1.4761207999999999	-4.6761869999999996	3.7603049000000000
H	-0.48990168000000001	-4.7722918999999999	3.6966733000000001
H	-1.7878076000000001	-5.3978361000000001	3.2508181000000000
O	3.1758875000000000	-2.9516339999999999	-1.6079583000000000
H	2.6824102000000001	-3.3525795000000000	-0.86000164000000001
H	3.9114719000000000	-2.4631093000000002	-1.1815245000000001
O	1.5858863000000001	-4.4648893999999997	6.1039608000000003
H	1.9944748999999999	-5.3225620999999999	6.1872818000000001
H	1.5083260999999999	-4.3471560000000000	5.1250821999999996
O	0.25021670000000001	-3.7369924000000001	-2.0641259999999999
H	0.90088478000000005	-4.2964973999999998	-2.5908419999999999
H	0.70088150000000005	-3.8269986999999999	-1.2159424000000001
O	3.4110543000000000	-7.5973474999999997	-0.13991523000000000
H	2.7277650000000002	-7.0728748000000001	0.25403358999999998
H	4.1659253999999999	-7.5746951999999999	0.44705080000000003
O	1.1656545000000000	-8.8759157000000002	3.3048008000000002
H	1.0815904999999999	-8.0555556999999993	2.7521469999999999
H	2.0583895999999999	-8.7807610999999994	3.6266421000000002
O	5.0825104000000003	-7.1802444000000003	2.4166156999999999
H	4.6199056000000001	-6.5449713000000003	2.9646865999999998
H	6.0241576999999999	-6.9530088000000001	2.3791186999999998
O	-1.5355553000000000	-1.9164806999999999	4.3539288999999997
H	-1.7591308000000001	-2.7986374999999999	4.0579023000000003
H	-0.88874874000000004	-2.1593881000000001	5.0036493000000002

C	0.15420413E+01	-0.32992388E+01	0.28628421E+01
C	0.18455660E+01	-0.19385852E+01	0.33765991E+01
O	0.16512537E+01	-0.98413994E+00	0.24009563E+01
C	0.14834779E+01	-0.16652073E+01	0.11223807E+01
C	0.14588676E+01	-0.31520533E+01	0.14342599E+01
O	0.20158193E+01	-0.15200422E+01	0.45441532E+01
C	0.23279254E+00	-0.10368133E+01	0.48720270E+00
O	-0.10567438E+01	-0.12919636E+01	0.12489658E+01
O	1.3689783000000000	-4.1031009999999997	0.55161384000000002
O	1.5204340999999999	-4.3909311000000004	3.5915070000000000
C	0.37736027E+00	0.57736991E+00	0.50549342E+00
O	-0.66524883E+00	0.12516520E+01	-0.13774253E+00
H	0.23993964E+01	-0.13601423E+01	0.54732017E+00
H	0.49950125E+00	0.98879029E+00	0.15089717E+01
H	0.12517535E+01	0.72904281E+00	-0.87992330E-01
H	-0.15219849E+01	0.78870459E+00	-0.65126087E-01
H	0.50057647E-01	-0.13610230E+01	-0.55653975E+00
H	-0.71701072E+00	-0.15137139E+01	0.21583624E+01
O	1.7911096000000000	-6.3912275999999997	1.7714951999999999
H	1.7946228000000000	-5.8749703000000002	2.6361530000000002
H	1.6390146000000001	-5.6321611000000003	1.2222184000000000
O	4.4923222999999997	-4.3995927000000004	1.2700864000000001
H	4.1240442000000002	-5.2355304000000000	1.5991166999999999
H	3.6497077999999998	-3.8865547999999999	1.2085352000000000
O	3.9488772999999999	-5.3910888000000003	4.2015997000000000
H	4.1445857999999998	-4.6514042000000000	4.7983924000000000
H	3.0401047999999999	-5.0346634000000003	3.8403228999999999
O	-1.3333573000000001	-4.9949734000000001	3.9816962999999999
H	-0.41222992000000003	-4.7707546000000001	3.7070853000000001
H	-1.4836373000000000	-5.8834926999999997	3.5610184999999999
O	-1.0595958000000001	-5.7658132000000002	0.13627425000000001
H	-0.35968512000000002	-4.9896243000000000	0.12835715000000000
H	-1.1826026999999999	-5.9427880999999996	1.1149973000000000
O	1.1750449000000001	-4.2674130999999997	6.2147753999999997
H	1.6087648999999999	-5.0459261000000000	6.4533186999999996
H	1.4258036000000001	-4.1335670000000002	5.2816938999999996
O	2.9603310000000000	-3.0928338000000002	-1.6265571999999999
H	2.5715070999999998	-3.7027554000000000	-1.0365160000000000
H	3.6236446000000000	-2.6513194000000002	-1.0429169000000000
O	5.7944606000000003E-002	-3.9045928000000001	-2.3138431000000002
H	0.77893986000000004	-4.1719638999999997	-2.9248360999999998
H	0.49734411000000001	-3.8820458000000002	-1.4363272000000000

O	-1.6014725000000001	-1.9898705000000001	4.5347299999999997
H	-1.1600189000000001	-2.8921481999999998	4.3688986999999999
H	-1.4530478000000000	-1.9780924000000000	5.4793816000000000
O	3.4530183999999999	-7.7354722999999996	0.14981201000000000
H	2.5958689000000001	-7.5189269000000003	0.62149639000000001
H	4.1280137999999997	-7.3175093999999996	0.77725367000000001
O	1.1839386000000001	-8.7410069000000004	3.4247817999999999
H	1.2674547000000000	-8.1426546000000002	2.7119260000000001
H	2.0542329000000001	-9.1068633000000005	3.3475465000000000
O	5.0603612000000000	-7.1775038999999996	2.1726394000000000
H	4.8505558000000004	-6.5350057000000001	2.8751655999999999
H	6.0205817000000001	-6.9396374999999999	2.1071925999999999

C	0.14637178E+01	-0.33108193E+01	0.27032038E+01
C	0.16130472E+01	-0.20641054E+01	0.34687659E+01
O	0.16355267E+01	-0.99049409E+00	0.26250927E+01
C	0.15931201E+01	-0.14291550E+01	0.12074299E+01
C	0.15480209E+01	-0.30005933E+01	0.13641240E+01
O	0.18318554E+01	-0.18080914E+01	0.46674736E+01
C	0.24502519E+00	-0.84922783E+00	0.69905130E+00
O	-0.86455697E+00	-0.13235903E+01	0.14833096E+01
O	1.3195276000000000	-3.7383712999999998	0.29578868000000003
O	1.2994068000000001	-4.4375602000000001	3.4029902999999999
C	0.28795044E+00	0.70847587E+00	0.61990620E+00
O	-0.29609334E+00	0.12056875E+01	-0.55317032E+00
H	0.24576940E+01	-0.95133889E+00	0.67554159E+00
H	-0.19422889E+00	0.11023646E+01	0.15584121E+01
H	0.12781281E+01	0.11057969E+01	0.48535160E+00
H	-0.12622495E+01	0.13522239E+01	-0.34278050E+00
H	0.13415224E+00	-0.11488256E+01	-0.33602452E+00
H	-0.57013576E+00	-0.14921455E+01	0.23671399E+01
O	1.6065507000000001	-6.3379317000000004	1.6295766000000000
H	1.5218346000000000	-5.8170118000000004	2.5161821999999998
H	1.3355592000000001	-5.5894944999999998	1.0908666000000000
O	-1.1131557000000001	-5.3500882000000001	0.18589172000000001
H	-0.38938178000000001	-4.7300377999999998	0.45252827000000001
H	-1.5896125000000001	-5.4659725000000003	1.0544123000000001
O	4.5204516000000003	-4.8700711999999999	1.0676555999999999
H	4.6683981000000001	-5.7769475999999997	1.4136854999999999
H	3.5855513000000001	-4.6348085000000001	1.1891198000000001
O	-1.4441583000000000	-4.8504896000000004	4.0392188000000004
H	-0.46335295999999998	-4.7137859000000004	3.8748464999999999
H	-1.6634921000000000	-5.5944118999999999	3.4549262999999999
O	3.7726264000000000	-5.5787778000000001	4.3328436000000004
H	3.8305107000000000	-4.8141194000000000	4.8057670000000003
H	2.8363209000000000	-5.4880165999999999	4.0706762000000003
O	3.0064570000000002	-2.8192865999999999	-1.6930334000000000
H	2.3519777999999998	-3.4128071000000002	-1.2862651000000001
H	3.4728607999999999	-2.3802743999999998	-0.94023836999999999
O	0.78836976999999997	-4.1674293999999996	6.2222990999999999
H	1.1064373999999999	-5.0749338000000002	6.4155578000000002
H	0.99973049000000003	-4.0263061999999996	5.2735384999999999
O	3.3270109999999999E-003	-3.5638431000000002	-2.3309821999999998
H	0.61564646999999995	-3.9846135999999999	-2.9625430000000001
H	0.41858574999999998	-3.5298824999999998	-1.4469669999999999
O	3.3938969000000001	-7.5524282999999999	0.15131642000000001
H	2.6324773000000001	-6.9791284999999998	0.45633040000000002
H	3.8552301000000000	-7.5355146000000000	0.99452876999999995
O	0.87453004999999995	-8.5520134999999993	3.3032645000000000
H	1.1249974000000000	-7.8413874000000003	2.6275529999999998
H	1.6540663000000000	-9.0879680999999994	3.1119838000000000
O	-0.32351174999999999	-6.7479078000000001	-2.1006046000000000
H	-0.32576547000000000	-6.2598267999999999	-1.2608024000000000
H	0.55340367999999995	-6.4395465999999999	-2.4538635000000002
O	-1.5729652999999999	-2.0334143000000000	4.5586279000000003
H	-1.7270823000000000	-2.9679617000000000	4.4221763000000003
H	-1.4875631000000000	-1.9549437999999999	5.5029474000000000

C	0.12544972E+01	-0.33575950E+01	0.27369362E+01
C	0.15682297E+01	-0.21173269E+01	0.34348418E+01
O	0.17257318E+01	-0.11154225E+01	0.25181044E+01
C	0.15793398E+01	-0.16544654E+01	0.11949770E+01
C	0.13822307E+01	-0.31332158E+01	0.13884221E+01
O	0.17918861E+01	-0.17156856E+01	0.45802312E+01
C	0.38190971E+00	-0.82449629E+00	0.57265537E+00
O	-0.87830854E+00	-0.92707839E+00	0.12813508E+01

O	1.5160452000000000	-3.9156894000000002	0.36795605999999997
O	1.0100009000000001	-4.5294800999999998	3.3790363000000001
C	0.69618811E+00	0.62101239E+00	0.55144131E+00
O	-0.31076938E+00	0.12811291E+01	-0.35084934E+00
H	0.24791468E+01	-0.14104013E+01	0.67463645E+00
H	0.55441355E+00	0.92585104E+00	0.16169187E+01
H	0.16827721E+01	0.87949752E+00	-0.19287036E-01
H	-0.12611245E+01	0.12094771E+01	-0.63847202E-01
H	0.70814938E-01	-0.12021285E+01	-0.42185959E+00
H	-0.65050162E+00	-0.12499952E+01	0.22144423E+01
O	1.6412458000000001	-6.4121883000000004	1.4589544999999999
H	1.4734023999999999	-5.9589970000000001	2.3707601000000000
H	1.7463465000000000	-5.5772868000000004	0.98884274999999999
O	-1.0884727999999999	-5.9483870999999997	0.42332998999999999
H	-0.36822080000000001	-5.3334415000000002	0.64645779000000003
H	-1.2895112000000000	-6.3573779999999998	1.3043648000000001
O	4.2021784000000002	-5.0270054999999996	0.99398894000000004
H	3.7033448999999998	-5.4393162999999998	1.6706287000000000
H	3.6352099999999998	-4.3644774000000002	0.65034380000000003
O	3.4282534000000000	-5.4505416999999996	4.4080952000000000
H	3.7021068000000001	-4.6498993999999998	4.9409403999999997
H	2.5857418999999999	-5.1637556000000000	4.1029552000000002
O	-1.7740467000000000	-4.7211622000000002	3.9032456999999998
H	-0.82958646000000003	-4.7947126999999998	3.6560388000000001
H	-2.1042025999999998	-5.5293444000000003	3.5068136999999999
O	0.28073083999999998	-3.5138639000000000	-1.9734520000000000
H	0.88469529999999996	-4.0575159000000003	-2.5455345999999999
H	0.78028520000000001	-3.5176677000000001	-1.1236614000000000
O	3.1165707000000000	-3.0664551000000002	-1.5841491000000001
H	2.5237153000000001	-3.5691532000000001	-0.93433215000000003
H	3.8402731000000001	-2.7827373000000000	-1.0418099000000001
O	0.74484492000000002	-4.3387688999999998	6.1276378999999999
H	1.4156032000000001	-4.8977314999999999	6.4148835999999996
H	0.74642140000000001	-4.2469086000000003	5.1897754000000003
O	3.6371701000000001	-7.5215886999999997	-6.2637210999999998E-002
H	2.9835935000000000	-7.1534738000000004	0.61814232000000002
H	4.4176539999999997	-7.1990015999999999	0.33910643000000001
O	0.81043653000000004	-8.6532932000000002	2.8185839000000001
H	0.81556892999999997	-7.9254334000000002	2.2310696999999999
H	1.7585645999999999	-8.8899260000000009	2.7106240000000001
O	-1.8718657999999999	-1.8675264000000000	4.2115764999999996
H	-1.7716113000000000	-2.7848570000000001	3.8827913999999999
H	-2.4248435000000002	-2.0509746999999998	4.9760418000000000
O	4.0070677000000003	-3.0315175000000001	5.5555895000000000
H	3.2045701000000002	-2.7909730000000001	5.0522755000000004
H	4.4289679000000000	-2.2162266000000002	5.8086460000000004

C	0.13434138E+01	-0.33335752E+01	0.27672288E+01
C	0.15982676E+01	-0.20466316E+01	0.33740080E+01
O	0.16813559E+01	-0.10859654E+01	0.23792148E+01
C	0.14998226E+01	-0.16348386E+01	0.11353840E+01
C	0.13243565E+01	-0.31287635E+01	0.13871206E+01
O	0.18287509E+01	-0.16877204E+01	0.45354827E+01
C	0.26668634E+00	-0.94350167E+00	0.54819227E+00
O	-0.89258277E+00	-0.12521199E+01	0.13883142E+01
O	1.2340008000000000	-4.0414234999999996	0.44477815999999998
O	1.1026080000000000	-4.5023758000000003	3.4009083000000002
C	0.57554932E+00	0.52698811E+00	0.36630426E+00
O	-0.54711010E+00	0.12453417E+01	-0.22655484E+00
H	0.23344254E+01	-0.14326358E+01	0.45394825E+00
H	0.91687123E+00	0.10204660E+01	0.12152744E+01
H	0.13825000E+01	0.80470922E+00	-0.29766564E+00
H	-0.13828052E+01	0.92501141E+00	0.17907624E+00
H	0.18978207E-01	-0.13640371E+01	-0.44030696E+00
H	-0.65439673E+00	-0.15542456E+01	0.22333736E+01
O	1.9784264000000000	-6.4369478000000004	1.7992052999999999
H	1.6346890999999999	-5.9811978000000003	2.6606733000000000
H	1.6381228999999999	-5.7619080000000000	1.1971257000000000
O	-0.81940268000000005	-6.0623791000000002	0.53468985000000002
H	-0.24086102000000001	-5.2724548999999996	0.53144716999999997
H	-1.0548706000000001	-6.2109781000000002	1.4495822000000000
O	4.2853887000000004	-4.9520334999999998	1.1322291000000000
H	4.1935789999999997	-5.3002212000000002	2.0395476000000001
H	3.3444091000000000	-4.7392056000000000	1.0176788000000001
O	-1.7563998999999999	-4.7775904000000002	3.5719690000000002
H	-0.76890628000000005	-4.7717403999999997	3.4653182999999999
H	-1.9731112000000000	-5.7036246999999998	3.3376925000000002
O	3.3861987000000000	-5.3905194999999999	4.4216107999999998
H	3.6198359000000000	-4.6024681000000003	4.9488035999999997
H	2.4093545999999999	-5.2162601000000004	4.1953126000000003
O	0.52362635999999996	-4.5438963000000001	5.9555347000000003
H	1.0114563999999999	-5.3090355000000002	6.2507524999999999
H	0.64718885999999998	-4.5564504000000001	4.9551287000000004
O	3.1151502000000000	-3.0888103000000000	-1.4904968000000001
H	2.5381863000000000	-3.6490008999999999	-0.82133639999999997
H	3.5903214000000001	-2.4701303999999999	-0.94727183999999998
O	0.15649969999999999	-3.5706003000000002	-2.1889797000000000
H	0.79803290000000005	-3.9409496000000002	-2.9000799000000002
H	0.61072978000000000	-3.7266276999999999	-1.3557117000000001
O	0.80616326999999999	-8.7039495999999996	2.6519278000000002
H	1.1779826000000000	-7.9089191999999997	2.1840758000000000
H	1.6176037000000001	-9.1970279999999995	2.6270728999999999
O	3.6730000000000000	-7.6304740999999998	-0.23272749000000001
H	3.1204326000000000	-7.0517740000000000	0.30329992000000000
H	4.5110912000000001	-7.6568889000000002	0.28393688000000000
O	-3.0690632999999998	-2.9979095000000000	0.61401269999999997
H	-2.9881063000000001	-3.2692144999999999	-0.30701811000000001
H	-2.2532562000000000	-2.5576759999999998	0.75477592000000004
O	4.4353638999999996	-7.5594067000000003	2.9501859000000001
H	3.8604134999999999	-6.9778086999999998	3.4980693999999999
H	5.3274863999999997	-7.0930520000000001	3.0076801000000000

C	0.14526639E+01	-0.34432261E+01	0.28498919E+01
C	0.17211146E+01	-0.21369190E+01	0.34723102E+01
O	0.18459034E+01	-0.11184004E+01	0.25814670E+01
C	0.16472122E+01	-0.16731060E+01	0.12462494E+01
C	0.14966897E+01	-0.32071166E+01	0.14770255E+01
O	0.18001810E+01	-0.18499307E+01	0.47397130E+01
C	0.27853206E+00	-0.10446790E+01	0.68626494E+00
O	-0.92974993E+00	-0.13178084E+01	0.14068543E+01
O	1.3523434999999999	-4.0425749000000000	0.46229863999999998
O	0.98705366000000005	-4.5263286999999996	3.5238418999999999
C	0.42150581E+00	0.45950515E+00	0.69145854E+00
O	-0.58265792E+00	0.11594656E+01	0.39458126E-02
H	0.24798222E+01	-0.14950800E+01	0.61990512E+00
H	0.39529729E+00	0.78413607E+00	0.17970620E+01
H	0.13885539E+01	0.81391078E+00	0.25223600E+00
H	-0.15150324E+01	0.10942630E+01	0.34122269E+00
H	0.77304031E-01	-0.14897180E+01	-0.30816815E+00
H	-0.72887957E+00	-0.18280084E+01	0.22399945E+01
O	1.8433898000000000	-6.4295466000000001	1.7964613000000000
H	1.5598977000000001	-6.0408527999999997	2.6509166999999998
H	1.6828689999999999	-5.6227375000000004	1.2420848000000000
O	-0.55389306000000005	-6.2311002000000002	0.59187855000000000
H	-2.8524727000000000E-002	-5.4413000000000000	0.62317920000000004
H	-0.21635531000000000	-6.7663617000000000	1.2918183000000001
O	-1.5792922000000000	-5.0174099999999999	3.9158293999999998
H	-0.63609572000000003	-4.8430966000000000	3.7592826000000001
H	-1.7111771000000000	-5.9139790000000003	3.4162658000000001
O	3.4868952000000002	-5.2045132000000001	4.4947229000000002
H	3.5032383999999999	-4.4252815999999999	5.1174400999999996
H	2.5969050000000000	-5.1074476999999998	4.1562796999999998
O	4.3925770999999996	-4.8882021000000000	1.2544310999999999
H	4.0605362999999999	-4.0431289000000001	1.5256168999999999
H	3.6665288000000000	-5.4152844000000000	1.5181271000000001
O	3.2415033000000002	-3.5532604999999999	-1.4575855000000000
H	2.5371725999999999	-3.8676168000000000	-0.79909406000000005
H	3.7771096000000002	-3.0823154000000001	-0.87046661000000003
O	0.12062535000000001	-3.5777551000000001	-1.9786690000000000
H	0.73803114999999997	-4.1478833000000002	-2.5762944999999999
H	0.52640816999999995	-3.7356221000000001	-1.1278725000000001
O	0.39288222000000000	-4.4582860000000002	6.1337090999999999
H	0.81678912999999997	-5.3939792000000004	6.0292269000000003
H	0.56833076999999999	-4.1854943000000002	5.1820864000000002
O	0.54040845000000004	-8.5518994999999993	2.7033179000000001
H	1.1306920000000000	-7.7851812999999996	2.4854528999999999
H	1.1164398000000000	-9.0929304000000002	3.2941216999999998
O	3.5290626999999999	-7.7015022999999996	0.17362906000000000
H	2.8198322000000000	-7.1293806000000002	0.52957427999999995
H	4.2749968999999997	-7.7080479999999998	0.78734006000000001
O	-1.7999745000000000	-2.3690047999999999	4.6658027000000004
H	-1.9134386999999999	-3.2439657999999998	4.1444114000000001

H	-1.1442230000000000	-2.7383586000000002	5.2694364000000000
O	-3.0829198999999998	-2.8171501000000001	0.43380243000000002
H	-2.8089312000000000	-3.3349766000000001	-0.27088899000000000
H	-2.2168231999999999	-2.4754187999999999	0.74487152999999995

C	0.13501424E+01	-0.33051602E+01	0.28641344E+01
C	0.18344268E+01	-0.20889042E+01	0.34218565E+01
O	0.19325007E+01	-0.10270280E+01	0.24891957E+01
C	0.15994428E+01	-0.15789906E+01	0.11862378E+01
C	0.13970174E+01	-0.31019271E+01	0.14849324E+01
O	0.19494882E+01	-0.16277995E+01	0.45753246E+01
C	0.30060712E+00	-0.89327390E+00	0.66951033E+00
O	-0.94819974E+00	-0.11201129E+01	0.14262020E+01
O	1.1743889999999999	-4.0442907000000003	0.60539463000000004
O	1.1141919000000000	-4.4122697000000004	3.5026586000000002
C	0.45849808E+00	0.62988194E+00	0.72255321E+00
O	-0.54178686E+00	0.12947794E+01	-0.23090229E-01
H	0.24257259E+01	-0.12211661E+01	0.54254565E+00
H	0.34576885E+00	0.92307393E+00	0.17352769E+01
H	0.14593573E+01	0.95927494E+00	0.43069254E+00
H	-0.14382975E+01	0.91910409E+00	0.14084429E+00
H	0.21283747E+00	-0.11615157E+01	-0.33557626E+00
H	-0.63470042E+00	-0.15256817E+01	0.22594552E+01
O	1.9236778000000001	-6.5198258999999998	1.7963971000000001
H	1.7002550000000001	-5.9381012000000002	2.5531093000000000
H	1.9208889000000000	-5.7761535999999998	1.1544337000000000
O	-0.59936734000000003	-6.2014620000000003	0.46928275000000003
H	-1.8599211000000001E-002	-5.4500137000000004	0.58217184000000000
H	-0.21560660000000001	-6.8732889000000004	1.0459346000000000
O	-1.6460467999999999	-4.9302542999999996	3.9081198000000001
H	-0.67618102999999996	-4.6994774000000001	3.7572583000000002
H	-1.7181010000000001	-5.5909807000000002	3.1821823000000000
O	3.4880475000000000	-5.3957182000000001	4.5592902000000004
H	3.8342953000000000	-4.5131635000000001	4.9044321000000002
H	2.5794149000000002	-5.1309167999999996	4.2089226000000002
O	4.4959353999999996	-4.8263397000000001	1.6054462000000000
H	4.2578063000000004	-3.8619186999999999	1.5155858000000000
H	4.0748277000000002	-5.1012243000000002	2.4220473999999999
O	0.57541255999999996	-4.5126796000000002	6.1324759000000002
H	1.2385048000000001	-5.2035584000000004	6.3760912999999997
H	0.72331668000000005	-4.5133444999999996	5.1564740999999996
O	0.12202262000000000	-3.4943746000000000	-1.8547138999999999
H	0.72800960000000003	-4.0181300999999996	-2.3985365999999999
H	0.45436104999999999	-3.5353243000000001	-0.94272562999999998
O	3.5196371000000002	-3.6946642999999999	-1.1844572000000000
H	2.7214626000000002	-3.9256053999999998	-0.73750019000000000
H	4.1063727999999999	-3.2792110999999999	-0.59001190000000003
O	3.6850754000000001	-7.4347567000000003	-0.16474753000000000
H	2.9162938000000000	-6.8687503000000003	0.23670883000000001
H	4.3468385999999999	-7.2642861999999999	0.51753976999999995
O	0.81520901999999995	-8.7458437999999994	3.1531964000000001
H	0.93684007999999996	-7.8402893000000002	2.9504310000000000
H	1.6035435000000000	-9.1738143999999995	2.8121939999999999
O	-1.4642177000000001	-2.1737072999999998	4.6824612999999999
H	-2.0568369999999998	-2.8408864000000000	4.2441632000000000
H	-0.87143212999999997	-2.8020706999999998	5.0411142000000000
O	-3.0381027999999999	-2.9554301999999999	0.45703521000000003
H	-2.5513397000000002	-3.6300430000000001	-5.8299220999999998E-002
H	-2.2570863999999999	-2.4068947000000001	0.69445530000000000

C	0.12849221E+01	-0.34216006E+01	0.27696341E+01
C	0.17614761E+01	-0.21672592E+01	0.33045682E+01
O	0.19032614E+01	-0.11762066E+01	0.24167659E+01
C	0.14387332E+01	-0.16934910E+01	0.10807656E+01
C	0.13227643E+01	-0.32312528E+01	0.14294698E+01
O	0.21368328E+01	-0.18866838E+01	0.44862559E+01
C	0.23007241E+00	-0.86356717E+00	0.66782752E+00
O	-0.89846412E+00	-0.10507897E+01	0.15540012E+01
O	1.1323517000000001	-4.0866623999999998	0.47979040000000001
O	1.1075611999999999	-4.5768190000000004	3.5010606000000002
C	0.54130873E+00	0.64999714E+00	0.55338024E+00
O	-0.35929943E+00	0.13796066E+01	-0.27447529E+00
H	0.22807958E+01	-0.14998624E+01	0.42124551E+00
H	0.61949592E+00	0.11357394E+01	0.15074007E+01
H	0.15241922E+01	0.73437604E+00	0.10288120E+00
H	-0.12566712E+01	0.10883285E+01	0.25440453E-02
H	-0.75364012E-01	-0.12442200E+01	-0.36562322E+00
H	-0.56833391E+00	-0.16095103E+01	0.22889065E+01
O	2.1111304000000000	-6.4660482999999997	1.9632080999999999
H	1.7933380000000001	-5.9129054999999999	2.7694412000000002
H	2.0607123999999999	-5.8012088000000004	1.2927382000000001
O	-0.41056388999999999	-6.2505940000000004	0.33332679999999998
H	0.24888410000000000	-5.5321539000000000	0.44162130999999999
H	-0.57115804999999997	-6.4987493000000001	1.2775042999999999
O	3.6418664999999999	-4.9293278000000003	4.1453094000000004
H	3.7009059000000000	-4.5418504999999998	5.0002376000000002
H	2.6479659999999998	-4.7181062000000002	3.9899585000000002
O	-1.7963380000000000	-4.8542386000000004	3.8097598000000001
H	-0.77424857999999996	-4.7936396999999999	3.7431372999999999
H	-1.8839762000000000	-5.6124900000000002	3.2004890000000001
O	3.3174285999999999	-3.3019078999999998	-1.1119490999999999
H	2.5668080000000000	-3.5795743000000000	-0.55239004999999997
H	3.9088601999999999	-2.9890148999999999	-0.40600880000000000
O	0.33999329000000000	-3.3766387000000000	-1.9140009000000000
H	1.1748266999999999	-3.8243725999999998	-2.2153212999999998
H	0.42620881999999999	-3.5105602000000000	-0.98377987000000000
O	4.6813390999999998	-4.7112578999999997	1.7228380000000001
H	5.0474851999999997	-3.8092614999999999	1.5428329000000001
H	4.2626157999999999	-4.7025306999999996	2.6207943999999999
O	0.69041107000000002	-4.5427628999999996	6.3862009000000004
H	1.2032905000000000	-5.3129871000000000	6.7153470999999998
H	0.84105509000000001	-4.6015817999999999	5.4032517999999996
O	0.93655778999999995	-8.6486061999999997	3.1002100000000001
H	1.1993845999999999	-7.7864152000000004	2.6219853000000000
H	1.5113558000000000	-9.2567544000000002	2.6616206000000000
O	4.6268234000000001	-7.3495336000000000	2.6232237000000000
H	3.7128969000000001	-7.0148793999999999	2.5602961000000000
H	5.0981239000000000	-6.4943102000000001	2.7187171999999999
O	3.6755000000000000	-7.4742895000000003	-8.9476186999999999E-002
H	2.8919236000000001	-7.3781176999999998	0.48083936999999999
H	4.3337351000000002	-7.3340030000000000	0.66999595000000001
O	-1.1274341999999999	-2.3511370000000000	4.7439106999999998
H	-1.6402239999999999	-3.1450580000000001	4.4954453000000001
H	-0.38234101999999998	-2.7282150000000001	5.1937857999999997

C	0.12739277E+01	-0.32871721E+01	0.27407748E+01
C	0.16576097E+01	-0.20001430E+01	0.32748647E+01
O	0.18733521E+01	-0.10940615E+01	0.22866174E+01
C	0.15944258E+01	-0.17891065E+01	0.10297440E+01
C	0.13261697E+01	-0.32548874E+01	0.13758415E+01
O	0.18791802E+01	-0.17292350E+01	0.44803904E+01
C	0.34377965E+00	-0.11029811E+01	0.43778160E+00
O	-0.85198274E+00	-0.13798078E+01	0.12815121E+01
O	1.3965870000000000	-4.1998607999999997	0.52268245000000002
O	1.0889310999999999	-4.4398980000000003	3.5021198000000000
C	0.57200965E+00	0.39076728E+00	0.31685233E+00
O	-0.39936967E+00	0.11263051E+01	-0.48157668E+00
H	0.24548863E+01	-0.15137533E+01	0.38065964E+00
H	0.52863346E+00	0.81522425E+00	0.13434511E+01
H	0.15371168E+01	0.63221574E+00	-0.44343745E-01
H	-0.13229631E+01	0.87040183E+00	-0.27738325E+00
H	0.13207563E+00	-0.14905979E+01	-0.57967461E+00
H	-0.56266302E+00	-0.15647942E+01	0.22043193E+01
O	1.6137056000000001	-6.6691244000000003	1.9294016000000000
H	1.3723285999999999	-6.0427898000000004	2.6209673000000002
H	1.8230705000000000	-5.9475870000000004	1.2955167000000001
O	-0.66159076000000006	-6.0279097999999998	0.30249176999999999
H	-6.4442868000000000E-002	-5.2439714999999998	0.44617088999999999
H	-0.25980961000000002	-6.5984578000000003	0.98022704000000005
O	3.5966857999999999	-5.1166723000000003	4.2781472999999997
H	3.5594125999999999	-4.3462548999999999	4.9500621000000002
H	2.6931542999999998	-5.0582846999999997	3.8868450000000001
O	-1.5824658000000000	-4.9739887999999999	3.9826183999999998
H	-0.60611521999999995	-4.7462285000000000	3.8685067000000002
H	-1.6708407000000001	-5.6140176000000004	3.2671758999999998
O	4.5994522000000000	-4.2979786999999998	1.7097164000000000
H	4.8126992000000001	-3.3280460000000001	1.6734171000000000
H	4.1238625000000004	-4.2980292000000002	2.5926919000000002
O	0.26715265999999999	-3.8003999999999998	-1.9873167000000000
H	1.0468904999999999	-4.2121063000000003	-2.3955590000000000
H	0.41345714000000000	-3.9917199999999999	-1.0091755000000000
O	0.80451793999999999	-4.2958232000000001	6.1213902999999998

H	1.2870621000000000	-5.1455342000000002	6.2880849999999997
H	0.97047022000000005	-4.2452471000000003	5.1602793000000000
O	3.7499698000000001	-3.5822482000000000	-1.0590529000000000
H	3.0069707999999999	-3.9635951000000000	-0.62783827999999997
H	3.8906398000000002	-2.8786505999999998	-0.38936549999999998
O	-1.4326781000000000	-2.1871383999999998	4.9442513999999997
H	-1.8982794999999999	-3.0215233000000001	4.8945648000000004
H	-0.56839236000000004	-2.5943016000000001	5.0494824999999999
O	4.6143209000000001	-7.3318477000000000	2.4396296999999998
H	4.1007331000000002	-6.8192534000000000	3.1611609000000001
H	5.3592896000000003	-6.7863140000000000	2.2277955000000000
O	-2.8929635000000000	-3.0787957000000001	0.51961491999999998
H	-2.7957111000000001	-3.0711141000000000	-0.42826434000000002
H	-2.0876579000000000	-2.5600291999999998	0.76440785000000000
O	0.85756306000000004	-9.0702984000000004	3.0522165000000001
H	1.3053684999999999	-8.2282843999999997	2.8712754000000000
H	1.5841489000000000	-9.7576084999999999	2.9898764999999998

C	0.13992176E+01	-0.33646787E+01	0.27481709E+01
C	0.18438073E+01	-0.21240672E+01	0.32393651E+01
O	0.21068793E+01	-0.11985395E+01	0.22376780E+01
C	0.16620098E+01	-0.17512470E+01	0.10005456E+01
C	0.14225821E+01	-0.32621555E+01	0.13275015E+01
O	0.21330305E+01	-0.16063213E+01	0.43496219E+01
C	0.39108655E+00	-0.11110938E+01	0.43838629E+00
O	-0.70581355E+00	-0.13169897E+01	0.12808979E+01
O	1.2220131000000001	-4.1711516000000000	0.44469408999999999
O	1.2124016000000000	-4.4221994000000002	3.5232323999999999
C	0.52940465E+00	0.40958228E+00	0.33126890E+00
O	-0.55931473E+00	0.10707587E+01	-0.41460362E+00
H	0.25077744E+01	-0.16277313E+01	0.22234792E+00
H	0.62450058E+00	0.84111081E+00	0.13187586E+01
H	0.14997032E+01	0.63901978E+00	-0.22624059E+00
H	-0.14217193E+01	0.69601678E+00	-0.87016147E-01
H	0.20609766E+00	-0.16219759E+01	-0.55527704E+00
H	-0.41756231E+00	-0.18974458E+01	0.20506024E+01
O	1.6169054000000000	-6.6511402999999998	1.7799765999999999
H	1.6984716000000000	-5.9467195999999998	2.4015564999999999
H	1.3745148000000000	-6.0702784000000003	1.0096722000000000
O	3.7616529999999999	-5.0274524999999999	4.1740424000000003
H	3.5592676000000001	-4.5586422999999998	4.9942190000000002
H	2.8671217000000002	-4.7769678999999998	3.7801426999999999
O	-0.82127170000000005	-6.0216900999999998	0.15385330000000000
H	-0.21050132999999999	-5.2390869999999996	0.17820723999999999
H	-0.57247583999999996	-6.4165584999999998	0.98335368000000001
O	-1.4424391000000001	-4.8267737000000004	4.0249823999999998
H	-0.45348347000000000	-4.8042670000000003	3.9735775000000002
H	-1.6394629000000001	-5.4305933000000000	3.3187533999999999
O	8.6225906000000005E-002	-3.8275066999999998	-1.9214211999999999
H	0.61217529000000004	-4.4088095999999997	-2.4600415999999998
H	0.47382595000000000	-3.9540541000000000	-1.0908462000000001
O	0.60534723000000001	-4.2952821999999999	6.2306882999999997
H	1.0599280000000000	-5.1141284000000002	6.4765924000000004
H	0.93024074000000001	-4.2872633999999996	5.3039914000000001
O	3.8739686000000000	-3.5468098000000001	-1.1387252999999999
H	2.9886100000000000	-3.8273826999999998	-0.95183746000000002
H	4.0938490999999999	-2.9556239999999998	-0.33335506999999998
O	4.7494328000000001	-4.6149091000000002	1.5997755000000000
H	5.0979761999999997	-3.7312373000000001	1.4191705999999999
H	4.6310490000000000	-4.5220146999999997	2.5429203000000000
O	3.8807488999999999	-7.1651575999999997	-0.16318788000000001
H	2.9526995000000000	-7.1406485000000002	0.14035107999999999
H	4.3009104999999996	-6.5854436999999999	0.46317227000000000
O	-2.8935613999999998	-2.9900701000000001	0.74275005000000005
H	-2.8596138999999998	-3.3384713000000001	-0.15225482000000001
H	-1.9866813000000001	-2.5669059000000001	0.80397048999999998
O	0.85801936999999995	-9.1273511000000003	2.6968695999999999
H	0.81449066999999997	-8.2299462999999999	2.3593212000000001
H	1.8495592999999999	-9.1424140000000005	2.6265105000000002
O	-1.0960970000000001	-2.2352175999999999	5.4364505000000003
H	-1.4220027000000000	-2.8069350000000002	4.7246467000000001
H	-0.48657960000000000	-2.9044710000000000	5.8138202999999997

C	0.14395272E+01	-0.32740384E+01	0.27426258E+01
C	0.18035445E+01	-0.20213813E+01	0.32456897E+01
O	0.20281644E+01	-0.10801368E+01	0.22599319E+01
C	0.16461734E+01	-0.17688466E+01	0.10817010E+01
C	0.15022681E+01	-0.32447639E+01	0.13574831E+01
O	0.18446402E+01	-0.15978704E+01	0.44353691E+01
C	0.41312425E+00	-0.11038407E+01	0.48162084E+00
O	-0.77533666E+00	-0.13578761E+01	0.12751812E+01
O	1.4887242000000001	-4.1431152000000004	0.51088361000000004
O	1.2812116000000000	-4.4256542000000003	3.4457670000000000
C	0.57687707E+00	0.32352497E+00	0.30731417E+00
O	-0.44369623E+00	0.96244324E+00	-0.47356504E+00
H	0.24408319E+01	-0.15449885E+01	0.32185093E+00
H	0.55601626E+00	0.80587056E+00	0.12775492E+01
H	0.15507487E+01	0.49277117E+00	-0.24020995E+00
H	-0.12682720E+01	0.52578320E+00	-0.40933270E+00
H	0.28677464E+00	-0.15748979E+01	-0.45637235E+00
H	-0.53397976E+00	-0.20566903E+01	0.18823821E+01
O	1.4677338000000000	-6.6609482000000000	1.8608332999999999
H	1.4654581000000000	-5.9411388000000001	2.6117634000000001
H	1.6590973000000000	-6.0641243999999999	1.0849747999999999
O	3.8223417999999998	-5.1796870000000004	4.0775642000000003
H	3.7594102999999999	-4.5294403000000001	4.8332043999999996
H	2.9473758999999999	-5.0118881999999996	3.6135868000000002
O	-0.82859917999999999	-6.1579525999999998	0.25134351999999999
H	-4.1326537000000003E-002	-5.7215841999999997	0.63531088000000002
H	-1.2503146000000001	-6.5775907000000000	1.0644216000000000
O	-1.6003261000000000	-4.6409884999999997	3.8767284000000002
H	-0.66824600000000001	-4.8302322999999996	3.6678888999999999
H	-2.0750437000000002	-5.3214411000000004	3.3004424000000001
O	0.36488008999999999	-3.7742064000000002	-1.9862841000000000
H	0.76302448000000000	-4.3943399999999997	-2.5979250000000000
H	0.69040603000000000	-4.0341101000000004	-1.1117770000000000
O	4.5160026000000002	-4.5314490000000003	1.4679498000000000
H	4.8373830000000000	-3.5807148999999998	1.3049778000000001
H	4.6025631999999996	-4.6397852000000004	2.4395720000000001
O	0.88415431000000000	-4.0835122999999998	6.0625457999999997
H	1.3462955999999999	-4.9416566000000000	6.2294248000000003
H	1.1526156999999999	-3.9448702999999998	5.1830612000000000
O	3.4620392999999998	-7.1352934000000001	-4.3318601999999998E-002
H	2.6116552999999998	-7.4458241999999997	0.30141351999999999
H	3.5106066999999999	-6.3252427000000004	0.49884612000000000
O	3.9791338000000001	-3.8293544000000002	-1.1240095000000001
H	3.1166782999999998	-4.1440261999999999	-0.87202791999999996
H	4.1770889999999996	-3.2572874000000001	-0.44010629000000001
O	4.8673450999999996	-7.2437664000000002	2.6260576000000002
H	4.1827443000000004	-6.6019182000000001	2.8598219000000000
H	5.6686304999999999	-6.7308003999999997	2.4578215000000001
O	1.0905609000000001	-9.0554442000000002	3.1800508000000001
H	1.0420113000000000	-8.1741545999999996	2.7352916000000000
H	1.8094313000000000	-9.5042542000000001	2.6501960000000002
O	-3.0466693000000000	-3.1022308999999999	0.34633129000000001
H	-2.8330394000000001	-3.5103751999999999	-0.49079024999999998
H	-2.1851438999999999	-2.6791448000000000	0.69770178999999999

C	0.15662633E+01	-0.32526762E+01	0.27469064E+01
C	0.16159668E+01	-0.19490276E+01	0.32832259E+01
O	0.17948473E+01	-0.93777689E+00	0.23962709E+01
C	0.17071634E+01	-0.15821177E+01	0.11239615E+01
C	0.16313408E+01	-0.30935094E+01	0.13716045E+01
O	0.16136869E+01	-0.16440159E+01	0.45215580E+01
C	0.47080737E+00	-0.10413938E+01	0.38700870E+00
O	-0.71327381E+00	-0.13642610E+01	0.10781492E+01
O	1.5533375000000000	-4.0284350000000000	0.52828251999999998
O	1.4551318000000000	-4.3944004000000003	3.3530180999999999
C	0.51593517E+00	0.50955071E+00	0.20355520E+00
O	-0.60506640E+00	0.11378480E+01	-0.47979540E+00
H	0.25819638E+01	-0.13990035E+01	0.49860205E+00
H	0.72302112E+00	0.98902322E+00	0.12072936E+01
H	0.14766160E+01	0.66104160E+00	-0.41419090E+00
H	-0.13460317E+01	0.72745749E+00	0.13111015E-01
H	0.49808391E+00	-0.16331999E+01	-0.56809302E+00
H	-0.51863525E+00	-0.19346547E+01	0.17988567E+01
O	1.2544194000000000	-6.6443409999999998	1.7854219000000000
H	1.2938274000000001	-6.4083313999999998	2.7417677999999999
H	1.4665615999999999	-5.7851907999999996	1.4683351000000000
O	3.7301104000000000	-5.1491902999999999	4.2735988999999996
H	3.9365283000000000	-4.5285696000000000	4.9984599999999997
H	2.8490250000000001	-4.8194322999999999	3.9634960000000001

O	-1.4145194000000001	-4.9529934999999998	4.0387472000000004
H	-0.50965612000000005	-4.9262690999999998	3.6053386999999999
H	-1.8737893999999999	-5.6991087000000000	3.6710009000000001
O	-0.86669251000000003	-6.4330768999999997	3.1341985000000003E-002
H	-0.10713817000000000	-6.3797246999999997	0.65336844999999999
H	-1.5404670000000000	-6.4715179999999997	0.75786573999999995
O	4.7658499000000001	-4.6209750999999999	1.5973531000000001
H	4.7996306000000004	-3.6640804000000000	1.7988966000000000
H	4.4099475999999997	-4.9614294000000001	2.3861786000000000
O	3.5967180000000001	-7.1201502000000003	0.12959055999999999
H	2.7873294999999998	-6.7908379999999999	0.46093926000000002
H	4.0900531999999998	-7.2312709000000002	0.98539560000000004
O	0.14258353000000001	-4.1613005999999997	-2.0826069999999999
H	0.74733105000000000	-4.7773294000000002	-2.5763113999999998
H	0.56812289999999999	-4.0222217000000002	-1.2549653999999999
O	0.76012703999999998	-4.0498190000000003	6.1504477000000000
H	1.2829782000000001	-4.9059705999999998	6.3210278000000004
H	1.0341416000000001	-3.9213881000000002	5.2305197999999997
O	4.3195728000000004	-3.3936269000000001	-1.0894356000000001
H	3.6293636999999999	-3.9258266000000002	-0.64737661999999996
H	4.6399422000000001	-2.8080843000000000	-0.31252414000000001
O	4.4323426000000001	-7.1757472000000000	2.6771512000000000
H	3.8312303999999999	-6.5628986999999999	3.2083146999999999
H	5.2500717000000003	-6.6651702999999998	2.4551826000000001
O	3.7339473000000001	-2.8928932000000001	5.9950302000000004
H	2.9231368000000000	-2.7754500000000002	5.5076935999999996
H	4.1434851000000004	-1.9918947000000000	5.9871274999999997
O	1.0257885000000000	-9.1567483999999997	3.3174828000000001
H	0.92797848999999999	-8.4757438999999994	2.5932966999999998
H	1.9242163000000001	-9.5558209999999999	3.0513469999999998

C	0.15549846E+01	-0.33313730E+01	0.27446831E+01
C	0.19700836E+01	-0.20443995E+01	0.31675962E+01
O	0.20983832E+01	-0.11084801E+01	0.21005525E+01
C	0.17080653E+01	-0.18829267E+01	0.93119380E+00
C	0.15709492E+01	-0.33221313E+01	0.13365417E+01
O	0.20779266E+01	-0.16194025E+01	0.43156635E+01
C	0.38426206E+00	-0.12958987E+01	0.35811888E+00
O	-0.82368889E+00	-0.16731345E+01	0.10666312E+01
O	1.3725825000000000	-4.2775325000000004	0.49279817999999997
O	1.3442118000000001	-4.3015492999999996	3.6058078999999998
C	0.48865137E+00	0.19250805E+00	0.19794568E+00
O	-0.46118937E+00	0.82541701E+00	-0.76954670E+00
H	0.25103469E+01	-0.16186008E+01	0.21708024E+00
H	0.32834517E+00	0.50609069E+00	0.12388261E+01
H	0.14609629E+01	0.55961711E+00	-0.20805109E+00
H	-0.13241784E+01	0.41063063E+00	-0.46851108E+00
H	0.33757168E+00	-0.17161265E+01	-0.68760754E+00
H	-0.52849048E+00	-0.21899701E+01	0.18331370E+01
O	1.4940055999999999	-6.5507942999999997	2.0713199000000002
H	1.4732320000000001	-5.9669463000000000	2.8575959000000002
H	1.3417173000000000	-5.9368707000000001	1.4140623000000001
O	3.8447653000000002	-5.2194668000000002	4.3158652999999996
H	3.8785123000000001	-4.4150637000000001	4.8549429000000002
H	2.9539846000000001	-5.0249326999999999	3.9121277999999999
O	-1.4315485999999999	-4.5481924999999999	4.0481463000000000
H	-0.44947963000000002	-4.5402747999999997	4.0773149000000002
H	-1.5614389000000000	-5.2564446000000000	3.3669972000000001
O	-1.0050669999999999	-6.2388814999999997	-9.5481626000000000E-002
H	-0.42367116999999999	-5.5759580000000000	0.28047767000000001
H	-1.3749520000000000	-6.5207929000000000	0.76569293000000005
O	0.19700086000000000	-3.8724851999999998	-1.8630807000000000
H	0.61392846000000001	-4.6225351999999997	-2.3772593000000000
H	0.62438747000000006	-4.0154513999999999	-0.94420183999999996
O	4.4690010999999998	-4.8604402999999996	1.5712168000000000
H	4.5909678999999999	-3.9199763000000001	1.5931024000000000
H	4.3432263999999998	-5.1059798000000001	2.5473246999999999
O	4.0844787000000000	-3.5465556999999999	-1.0232720000000000
H	3.1448223999999998	-3.6491942000000002	-0.60705260000000005
H	4.6061790000000000	-3.0502992999999998	-0.35584655999999998
O	3.3073720999999998	-6.7849554000000003	-0.14548367000000001
H	2.6061861999999998	-6.9822923000000001	0.46160065000000000
H	3.5827265000000001	-6.0176109999999996	0.32222609000000002
O	1.0063926999999999	-4.3198566999999999	6.3059165000000004
H	1.5270353000000001	-5.1066511999999999	6.6427334000000000
H	1.2592989000000001	-4.1793893000000004	5.3693819999999999

O	-0.75065068999999995	-1.9265559999999999	4.8818818999999998
H	-1.3973802000000000	-2.6642458000000002	4.9013333000000001
H	5.3966972000000002E-002	-2.3912007000000002	4.9857519999999997
O	4.3348905000000002	-7.6233835000000001	2.8754721999999999
H	3.8991676000000002	-6.8699648000000000	3.3328126999999999
H	5.1416259999999996	-7.2035952999999999	2.5818827999999998
O	0.71722801000000003	-9.0840250000000005	2.8800867999999999
H	0.78166764000000000	-8.3116641999999992	2.3009255999999998
H	1.4723250999999999	-9.5815728999999994	2.5632024000000002

C	0.15045093E+01	-0.32110083E+01	0.26680412E+01
C	0.19915883E+01	-0.19540609E+01	0.31279319E+01
O	0.20754057E+01	-0.98571582E+00	0.20727055E+01
C	0.18212021E+01	-0.17360099E+01	0.79264380E+00
C	0.15467484E+01	-0.32303695E+01	0.12351678E+01
O	0.22764863E+01	-0.16308411E+01	0.43078686E+01
C	0.61402758E+00	-0.11825241E+01	0.14585221E+00
O	-0.55018457E+00	-0.13784118E+01	0.99489632E+00
O	1.2393445000000001	-4.1870488000000003	0.41799901000000000
O	1.3247897000000000	-4.1852635999999999	3.5555226000000002
C	0.71614697E+00	0.35320917E+00	-0.14300564E+00
O	-0.46488432E+00	0.83417625E+00	-0.72616085E+00
H	0.27048404E+01	-0.16598402E+01	0.23370974E+00
H	0.92545755E+00	0.92317455E+00	0.78399623E+00
H	0.15297809E+01	0.60621016E+00	-0.84280544E+00
H	-0.12335801E+01	0.63700557E+00	-0.17589174E+00
H	0.38124960E+00	-0.17216535E+01	-0.79535805E+00
H	-0.31848074E+00	-0.18064722E+01	0.18453509E+01
O	1.3643818999999999	-6.5273838000000000	1.8992663999999999
H	1.1072484000000000	-5.8563964000000004	2.5826033000000002
H	1.6383483000000001	-5.7835017000000004	1.2726401000000001
O	-1.3588563000000000	-4.6252332999999997	3.6273624000000000
H	-0.37308331000000000	-4.5677779999999997	3.6207425000000000
H	-1.4932799999999999	-5.3713097999999997	3.0448491000000000
O	3.7412079999999999	-5.1836774999999999	4.1401579000000002
H	3.8033573000000001	-4.4700081000000003	4.7506453000000004
H	2.7805352000000001	-5.0963535999999996	3.8632211000000001
O	4.6058811999999998	-4.8008350999999996	1.7418369000000000
H	4.7236016999999997	-3.8803793999999998	1.4930976000000000
H	4.3037276000000002	-4.6390599000000003	2.6350658999999998
O	2.6135815000000000E-003	-4.2079838000000001	-1.9530400999999999
H	0.47795613999999997	-4.9760429000000004	-2.3099999000000002
H	0.55573360000000005	-4.1530756000000002	-1.1280006000000000
O	-0.79882123999999999	-6.7325729000000001	-0.12764745999999999
H	-4.5704119000000001E-002	-6.7747466999999997	0.45743665999999999
H	-1.5254326000000000	-6.7469489999999999	0.54478643000000004
O	0.92118568999999995	-4.4630576000000000	6.2764445999999996
H	1.4782093999999999	-5.2440135999999997	6.4079101999999999
H	1.2206288999999999	-4.1840446000000000	5.3742552000000003
O	3.5307436000000001	-6.7036176999999997	-0.14507653000000001
H	2.8733080000000002	-7.2577444000000000	0.33265404999999998
H	3.8710011000000000	-6.2277823000000003	0.60535092000000001
O	4.2153321999999998	-3.7886153999999999	-1.2601925000000000
H	3.3433424000000000	-4.0539021999999996	-0.97333842999999998
H	4.5778876999999998	-3.3648842999999999	-0.49139829000000002
O	-0.99099696999999998	-2.1157493999999999	4.6551099999999996
H	-1.4807349999999999	-2.8233613000000002	4.1339264000000000
H	-0.21566647000000000	-2.6752204000000002	4.9436890000000000
O	4.4052851999999998	-7.4331236000000001	2.7433337999999998
H	3.8842211999999998	-6.7490788000000004	3.1560963000000002
H	5.2006451000000000	-7.0106973000000004	2.4887888000000000
O	0.92062597000000002	-8.8650000999999996	3.0220411000000000
H	1.1884725000000000	-7.9833039000000001	2.6415571000000000
H	1.5159833000000000	-9.4022924999999997	2.5108614999999999

C	0.15228785E+01	-0.34224646E+01	0.26940265E+01
C	0.18759526E+01	-0.20742920E+01	0.31207802E+01
O	0.19475394E+01	-0.12559390E+01	0.20255322E+01
C	0.16632525E+01	-0.19911156E+01	0.87673486E+00
C	0.13760896E+01	-0.33900021E+01	0.13319735E+01
O	0.20338255E+01	-0.15416814E+01	0.42171007E+01
C	0.39199041E+00	-0.13181199E+01	0.10596964E+00
O	-0.80515249E+00	-0.15650301E+01	0.91124416E+00
O	1.1520070000000000	-4.3638937999999996	0.49424407999999997
O	1.3781014000000000	-4.4244415000000004	3.5677770000000000
C	0.51784809E+00	0.11250931E+00	-0.44425030E-01

O	-0.59360697E+00	0.77599476E+00	-0.76639235E+00
H	0.25484351E+01	-0.19308239E+01	0.22617045E+00
H	0.69902658E+00	0.65952472E+00	0.92243190E+00
H	0.15133610E+01	0.37565155E+00	-0.56918538E+00
H	-0.13243149E+01	0.15872208E+00	-0.61764082E+00
H	0.19029769E+00	-0.19267650E+01	-0.79820279E+00
H	-0.65572586E+00	-0.22598915E+01	0.15346451E+01
O	1.5775300999999999	-6.5439572999999998	2.0429735999999998
H	1.4373404000000001	-5.8092385999999996	2.6605804000000002
H	1.4160896000000001	-5.9500413999999999	1.2459941000000001
O	-1.1977224000000000	-4.8488781999999997	3.8632637000000001
H	-0.25784657999999999	-4.7338003000000004	3.8272656999999999
H	-1.2948070000000000	-5.4408925999999997	3.1513105000000001
O	-0.70918323999999999	-6.4587763999999996	-3.2828314999999997E-002
H	-2.0368417999999999E-002	-5.8145522999999999	0.19839688000000000
H	-1.0600754999999999	-6.6947947000000001	0.89131322999999996
O	3.9483356000000001	-5.1497117000000001	4.2887040000000001
H	4.1053018000000003	-4.4134583999999997	4.9975548999999999
H	3.0325902000000000	-4.8397509000000003	4.1194078999999997
O	0.82695929000000001	-4.5718481999999998	6.1673220999999998
H	1.2822283000000001	-5.4777260999999999	6.1549994999999997
H	1.0773356999999999	-4.3935013999999999	5.2399448000000000
O	3.4288368000000000	-6.5804967999999997	-8.3707816000000004E-002
H	2.7119490000000002	-6.8763218999999998	0.54634488000000003
H	4.0325274000000002	-6.1854129000000002	0.57890516000000003
O	-2.3159616000000001E-002	-4.2043312999999998	-2.0627585000000002
H	0.42601364000000003	-4.7160672000000003	-2.7351470999999998
H	0.53385064999999998	-4.3145221999999999	-1.2597693000000001
O	4.9201743999999996	-4.8124402999999996	1.7455153000000001
H	4.9525544999999997	-3.8183139000000001	1.6232013000000001
H	4.5627122000000000	-4.8691722999999998	2.7061226999999999
O	4.2928261000000001	-3.7976847000000000	-1.0411542000000000
H	3.5657462999999998	-4.3389581000000002	-1.2364180000000000
H	4.0983014999999998	-3.5549637999999999	-0.11799930000000000
O	-0.87298587000000005	-2.2396913999999999	4.7667165000000002
H	-1.0965549000000001	-3.1508199000000001	4.7895415999999997
H	9.5095872999999997E-002	-2.4126878999999999	4.8897659999999998
O	0.80572675999999999	-8.8880704999999995	3.3734886999999998
H	0.93932888000000003	-8.0466519999999999	2.8694861000000000
H	1.5736521999999999	-9.3529496000000005	2.9292932999999999
O	4.8250956000000000	-2.2882394000000001	1.1925105000000000
H	4.5547591000000001	-1.4837562000000000	1.7471010000000000
H	5.6785215999999998	-1.9294072000000000	0.82668543999999999

C	0.17573252E+01	-0.34471008E+01	0.27094534E+01
C	0.20337527E+01	-0.20850401E+01	0.32308399E+01
O	0.19686685E+01	-0.11505246E+01	0.22099043E+01
C	0.17331119E+01	-0.19106598E+01	0.97764294E+00
C	0.15956051E+01	-0.33860256E+01	0.13313413E+01
O	0.22267031E+01	-0.16199571E+01	0.44109715E+01
C	0.52782008E+00	-0.13863539E+01	0.22666593E+00
O	-0.67628262E+00	-0.17303058E+01	0.95954814E+00
O	1.3833679999999999	-4.3254403999999997	0.43643728999999998
O	1.7634190999999999	-4.4651931999999999	3.5856083000000001
C	0.44875389E+00	0.14615207E+00	0.21686090E-01
O	-0.66654917E+00	0.69612625E+00	-0.70557988E+00
H	0.26774671E+01	-0.17037300E+01	0.39445756E+00
H	0.66685604E+00	0.63616163E+00	0.98997403E+00
H	0.13605108E+01	0.35401588E+00	-0.57149506E+00
H	-0.15728201E+01	0.59219987E+00	-0.38721144E+00
H	0.48977558E+00	-0.18289890E+01	-0.75566899E+00
H	-0.56732258E+00	-0.25326971E+01	0.15061248E+01
O	1.3700870000000001	-6.5931632000000002	1.7898202000000001
H	1.5134027000000001	-5.9621056000000001	2.4735046999999999
H	1.4014470999999999	-5.9657837999999996	1.0264891000000000
O	-1.2664901000000000	-4.7630046000000004	3.7653075999999999
H	-0.29573680000000002	-4.8955589000000002	3.7385286000000000
H	-1.6644664000000000	-5.3319742000000003	3.0589803000000000
O	4.2157039999999997	-5.2213329000000002	4.4368027999999997
H	4.3575261000000003	-4.3930759000000004	5.0072343999999998
H	3.3487621999999999	-5.0434502999999999	4.0246452000000001
O	3.7357566000000002	-3.9866299999999999	-1.1426002000000000
H	2.8822600999999999	-4.1038122000000001	-0.63084505000000002
H	4.3876264999999997	-3.7380418999999998	-0.46885157999999999
O	-0.71712814000000003	-6.6655547999999998	-0.32215480000000002
H	-0.10169015000000001	-5.9420232000000004	1.8807896000000001E-002
H	-1.0975984999999999	-6.9609272999999998	0.54616942999999996
O	4.6993736999999998	-5.0059239000000000	1.6447961000000000
H	4.5745475999999998	-4.0260714000000002	1.4953780000000001
H	4.9448958000000003	-5.0088233999999998	2.6007636000000001
O	3.3212136000000001	-6.9045380999999999	-0.12782504000000000
H	2.5689703000000002	-7.3029716000000002	0.33714988000000001
H	3.6618906999999998	-6.2793951999999997	0.51916242999999995
O	0.84929511000000002	-4.5463588000000001	6.1882352000000003
H	1.2977358000000001	-5.3846958999999996	6.4485241999999996
H	1.1849483000000001	-4.3628390000000001	5.2689466999999999
O	0.15620993000000000	-4.0067972000000003	-2.0712180999999998
H	0.85414948999999996	-4.3979287999999999	-2.6007668000000002
H	0.60387332000000005	-4.0379794000000002	-1.1770073999999999
O	0.68816290000000002	-8.7185337000000001	3.4855898999999999
H	1.0155391000000000	-7.9748516000000000	2.9997384999999999
H	1.3347198000000000	-9.3415645000000005	3.1355746999999998
O	4.5777175999999997	-7.6851696000000000	3.1369932999999999
H	4.2026348999999996	-6.8728864999999999	3.6225052999999998
H	5.4883094999999997	-7.3657108999999998	2.9447796999999998
O	4.8218052000000000	-2.2604706000000001	0.97129178000000005
H	4.6542164000000001	-1.5328349999999999	1.5848156000000000
H	5.7569971000000004	-2.0085088000000000	0.61491006000000004

C	0.16255096E+01	-0.34316977E+01	0.26317527E+01
C	0.19432184E+01	-0.20769633E+01	0.31141704E+01
O	0.18880464E+01	-0.10897577E+01	0.20677335E+01
C	0.16110429E+01	-0.18585977E+01	0.87826413E+00
C	0.14998020E+01	-0.33207002E+01	0.12291224E+01
O	0.22786455E+01	-0.16849696E+01	0.42462837E+01
C	0.33580513E+00	-0.13823395E+01	0.11245411E+00
O	-0.90161077E+00	-0.17006226E+01	0.72716302E+00
O	1.2300660000000001	-4.2487985999999998	0.34326060000000003
O	1.4575172999999999	-4.5158431999999999	3.3711970999999998
C	0.37353966E+00	0.99952620E-01	-0.10218593E+00
O	-0.68198961E+00	0.70957797E+00	-0.88761873E+00
H	0.24103436E+01	-0.17690953E+01	0.17385482E+00
H	0.24679155E+00	0.54333840E+00	0.81694244E+00
H	0.13389293E+01	0.40822345E+00	-0.64705085E+00
H	-0.15450947E+01	0.67634522E+00	-0.31923849E+00
H	0.25342077E+00	-0.18808489E+01	-0.86550518E+00
H	-0.74177949E+00	-0.26046862E+01	0.10611269E+01
O	1.1194001000000000	-6.6605403000000001	1.6632690000000001
H	1.1443525999999999	-6.2068725999999996	2.5599929000000001
H	1.3239150000000000	-5.9153485999999997	1.0724879000000000
O	-1.2678548999999999	-4.8128671000000001	4.0269162999999999
H	-0.36645823000000000	-4.7392620000000001	3.6865011000000001
H	-1.7514426999999999	-5.4999007000000004	3.4897274000000000
O	4.5644168000000001	-4.8262387000000002	1.5044525000000000
H	4.7049631999999999	-3.9124840999999999	1.3296517000000001
H	4.0100933999999997	-4.6161722999999997	2.2404046000000002
O	-0.42930496000000001	-6.3381369000000003	-0.56327969000000000
H	-0.28886172999999998	-5.4459415000000000	-0.21308856000000001
H	-0.14581717999999999	-6.7606026000000004	0.23122593000000000
O	3.8967181000000002	-5.3011054000000000	4.3815233999999998
H	4.1072733000000001	-4.4603969000000001	4.8646409000000004
H	2.9867454000000002	-5.1826827000000000	4.0492983000000002
O	3.6825561000000002	-3.8486674000000001	-1.2481214000000000
H	2.7782464000000000	-4.0805062000000003	-0.89510548999999995
H	4.1463739000000004	-3.6019405999999998	-0.41110588999999997
O	0.19604494000000000	-4.0924589999999998	-2.2316493999999998
H	0.83791420999999999	-4.6643306999999998	-2.6932874999999998
H	0.64680983000000003	-4.1177244999999996	-1.3155553000000000
O	0.80434753000000003	-4.3605910999999997	6.0682529000000001
H	1.3783285999999999	-5.0623072999999996	6.2752518999999998
H	0.91022051000000004	-4.3973012000000002	5.1083413999999996
O	3.5544585000000000	-6.7830887999999998	-9.1765997000000002E-002
H	2.6290648000000001	-6.8785162000000000	0.11246510999999999
H	3.9278430000000002	-6.1643961000000003	0.58636801000000005
O	-0.98414365999999998	-2.4983732999999999	5.3410399000000002
H	-1.1510254000000000	-3.3369599000000001	4.8126344000000003
H	-0.18352663999999999	-2.7659332999999999	5.7829028999999998
O	4.8854642000000004	-2.2791895000000002	0.88713781999999997
H	4.8532083999999998	-1.4424093000000000	1.3799387000000001
H	5.8494804000000000	-2.1215411000000000	0.61236809000000003
O	4.5609169999999999	-7.6731945000000001	2.9307118000000001
H	4.3092791000000004	-6.9558086000000001	3.4918589000000000

H	5.2337939999999996	-7.2027926999999998	2.4317049000000002
